# TnpPred: A Web Service for the Robust Prediction of Prokaryotic Transposases

**DOI:** 10.1155/2012/678761

**Published:** 2012-11-18

**Authors:** Gonzalo Riadi, Cristobal Medina-Moenne, David S. Holmes

**Affiliations:** ^1^Center for Bioinformatics and Genome Biology, Fundación Ciencia para la Vida Y Facultad de Ciencias Biologicas, Universidad Andres Bello, Santiago, Chile; ^2^Centro de Bioinformática y Simulación Molecular, Universidad de Talca, Talca, Chile

## Abstract

Transposases (Tnps) are enzymes that participate in the movement of insertion sequences (ISs) within and between genomes. Genes that encode Tnps are amongst the most abundant and widely distributed genes in nature. However, they are difficult to predict bioinformatically and given the increasing availability of prokaryotic genomes and metagenomes, it is incumbent to develop rapid, high quality automatic annotation of ISs. This need prompted us to develop a web service, termed TnpPred for Tnp discovery. It provides better sensitivity and specificity for Tnp predictions than given by currently available programs as determined by ROC analysis. TnpPred should be useful for improving genome annotation. The TnpPred web service is freely available for noncommercial use.

## 1. Introduction

Insertion sequences (ISs) are small, mobile DNA elements that usually contain a gene encoding a transposase that catalyzes the movement of the ISs from one part of the genome to another. ISs are found in nearly all prokaryotes [1, 2], sometimes at very high frequency per genome and are among the most abundant genes in nature [3]. They play a major role in lateral gene transfer, genome organization, and genome evolution [4]. Many ISs are bounded by short terminal inverted repeats (IRs) and some generate short direct repeats (DRs) when they integrate into the genome. ISs are classified into 19 families based on amino acid sequence similarity of the transposases, DNA sequence similarity including respective IRs and DRs and, in some cases, supported by phylogenetic profiling [5, 6].

Given the increasing availability of prokaryotic genomes and metagenomes, it is incumbent to develop rapid, high quality automatic annotation of ISs. Unfortunately, currently transposases of many ISs are incorrectly annotated as having other functions or are identified as “hypothetical.” In addition, their annotation is exacerbated by the presence of numerous partial ISs scattered in most genomes, representing the remains of once active ISs. 

Recently, the web application ISsaga was released, providing high quality ISs annotation [7], based on information available from curated ISs families present in the ISfinder database [5]. One advantage of the ISsaga pipeline is that it combines IS (DNA) and transposase (protein) sequence searches for the prediction of complete and partial ISs. The DNA and protein sequence searches are based on a suite of BLAST programs (BLASTN, BLASTX, and BLASTP) [8, 9]. IScan is another application that makes use of BLAST to scan whole genomes for ISs and includes in its prediction pipeline searches for transposases and inverted and direct repeats [10]. However, it is widely acknowledged that sequence-sequence comparison as carried out by this BLAST suite is inferior to profile-sequence comparisons such as Profile Hidden Markov Models (HMMs) when searching for remote homologies [11]. Recognizing this advantage, HMMs have been generated for transposases belonging to 19 of the 23 characterized families of ISs (PFAM database [12] and ACLAME database [13]). An additional bioinformatic resource for IS prediction is the Superfamily database [14] of structural and functional annotation of genomes based on a library of HMM profiles derived from structural domains in SCOP database [15]. Currently, Superfamily hosts 6 HMM profiles from domains belonging to two prokaryotic families of transposases, mu bacteriophage transposase, and IS200. A third HMM profile in Superfamily recognizes the eukaryotic Hermes transposase.

Since existing bioinformatic resources for predicting transposases via HMMs are limited to less than 60% of the IS families, we have developed a web service, termed TnpPred, that provides HMM profiles for transposases of the remaining ~40% of the IS families. In addition, newly available sequence information and manual curation allowed us to generate new HMM profiles for the ~60% of IS families for which HMM profiles already exist, that, with the exception of two cases, are as sensitive or in some cases more sensitive than those currently available in the PFAM database.

## 2. Materials and Methods

Transposase sequences were obtained from the ISFinder website [5]. The sequences were then manually curated using Blastp against RefSeq database [16], and several HMM profiles were developed for each IS family using multiple sequence alignments generated by ClustalW [17] and HMMer software [18], version 2.3.2. TnpPred was programmed in HTML [19] and Cascading Style Sheets, CSS [20], complying with the World Wide Web Consortium, W3C (http://www.w3.org/), guidelines. Compliance with these guidelines facilitates the accessibility of Mobilomics from any browser in any available operative system. Evaluation of the sensitivity and specificity of the HMM models was done by ROC analysis [21].

## 3. Results and Discussion

### 3.1. Validation of TnpPred HMM Profiles

In order to evaluate the sensitivity and selectivity of the TnpPred HMM profiles for predicting transposases, the HMMs were subjected to ROC curve analysis [21]. This analysis compared the performance of TnpPred HMMs derived from our study with those derived from Pfam and assessed their ability to identify transposases in a database of known, curated transposases (known positives database) versus a database of sequences devoid of known transposases (known negatives database). The known positives transposase database was constructed by amalgamating the database of ISfinder [5] with transposases from RefSeq. The known negatives transposase database was made with all sequences from Swiss-Prot Database [22, 23] after the elimination of all entries tagged as transposases, insertion sequences, resolvases, recombinases, and integrases. The ROC curves for 19 IS families are provided in supplementary file 1, see Supplementary Material available online at doi:10.1155/2012/678761 and are available for downloading at http://www.mobilomics.cl/.

TnpPRED HMMs have equal or superior selectivity, sensitivity, and cutoff *e*-value scores compared to those derived from Pfam HMM profiles for the prediction of Tnps belonging to 17 families of ISs (Table 1, marked with an asterick). In the two remaining cases, namely, Transposase mut of IS Family IS256 and Transposase 7 of IS Family Tn3, Pfam HMM profile outperforms TnpPRED HMMs in at least one performance criterion. In the case of IS family IS256, Pfam's Transposase mut outperforms TnpPRED HMM Profile in its selectivity and sensitivity. In the case of Tn3, Pfam's Profile Transposase 7 outperforms TnpPRED in selectivity but not sensitivity (Table 1). For these reasons, the Pfam HMM profile for predicting IS256 transposase members has been incorporated into the profiles used for the TnpPRED prediction web service, whereas both the Pfam HMM profiles and TnpPRED HMM profiles are used to predict Tn3 IS family members (Table 1, marked with asterisk). 

### 3.2. Comparison of TnpPred with ISsaga

To assess the predictive power of ISsaga, it was used to predict Tnps in the genomes of *Acaryochloris marina* MBIC11017 and *Stenotrophomonas maltophilia* K279a [7]. We have also annotated these two genomes in order to compare the Tnps predictions of TnpPred with those of ISsaga and to evaluate the types of additional IS predictions offered by TnpPred. In the genome of *Acaryochloris marina* MBIC11017, ISsaga predicts a total of 272 Tnps for 17 IS families or subfamilies, whereas TnpPred HMM profiles predict a total of 266 Tnps for the same 17 IS families (Table 2). Summing the predictions for both ISsaga and TnpPred gives a total of 293 unique Tnps. In the case of *S. maltophilia* K279a, ISsaga predicts a total of 39 Tnps from 9 IS families, whereas TnpPred predicts a total of 47 Tnps for 10 IS families (Table 2) summing to 53 unique Tnps.

**Figure 1:**
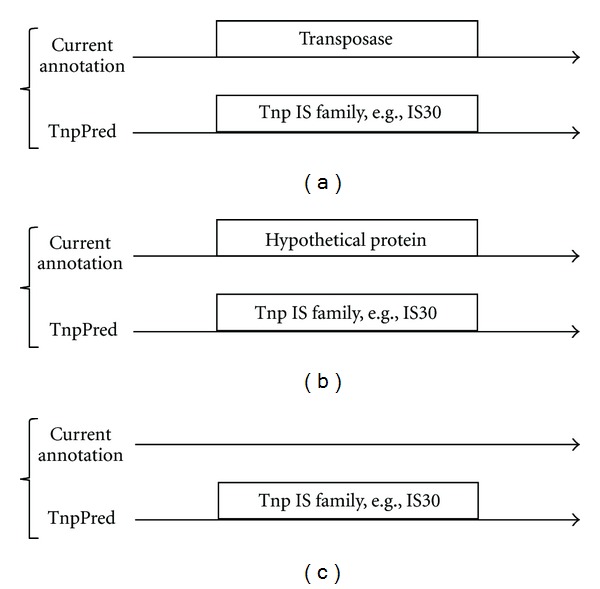
Classes of improvement of gene annotation using TnpPred. (a) Additional information such as “family classifiaction” is provided for a previously annotated transposase, (b) prediction of a transposase where a previously hypothetical gene had been annotated, (c) prediction of a transposase where no prior annotation existed.

Inspection of the Tnps predicted by TnpPred but not by ISsaga revealed three broad classes of novel predictions as outlined in Figure 1. In class (a), TnpPred provides a family prediction for a gene previously annotated only as “transposase”; in class (b), TnpPred adds information to a gene previously annotated as “hypothetical” or with “no known function” and in class (c), TnpPred predicts a Tnp in a DNA sequence where no gene had previously been annotated.

In *A. marina* MBIC11017, an example of a class (a) annotation improvement is YP_001515477.1, annotated in ISsaga as “transposase” and in TnpPred as “transposase Family IS630”; a class (b) annotation improvement is YP_001516695.1, annotated as “hypothetical protein” in ISsaga and as “IS5 sub-family ISL2” in TnpPred, and a class (c) annotation improvement is a sequence not annotated in ISsaga (coordinates 5666475..5666933 +strand) and as “IS200/IS605” in TnpPred (supplementary files 2 and 4). Similar examples exist for *S. maltophilia* K279a (supplementary files 3 and 5).

However, TnpPred failed to detect 27 out of 293 predicted Tnps in *A. marina* MBIC11017 and 6 Tnps out of 53 in *S. maltophilia* K279a. There are several possible reasons for this: (i) some sequences in ISsaga fall below the accepted e value cutoff for TnpPred, (ii) some sequences are incorrectly annotated in ISsaga because it uses the NCBI nr data base via BLAST to predict Tnps and some of these Tnps are incorrectly annotated in NCBI, and (iii) ISsaga has predictions for four new families of Tnps [5] that were not available when TnpPred was developed. These new families will be incorporated into a future update of TnpPred.

### 3.3. Additional Discussion

TnpPred is able to detect fragments or pseudogenes of Tnps if the relevant sequence has an e-value lower than the accepted evalue cutoff score specified by the HMM. It is often useful to be able to detect such “molecular fossils” because they can aid in the prediction of genes and gene islands, including pathogenicity islands, that may have been horizontally transferred [24, 25].

## 4. Conclusions

TnpPred is a web service that supplements and extends currently available programs and HMM Profiles for the prediction of 19 prokaryotic transposase families. In a comparison of the sensitivity and selectivity by ROC analysis of the HMMs used by TnpPred versus those used by Pfam HMMs, the TnpPred predictions of the 19 families outperformed Pfam in all but two cases. The ability of TnpPred to predict Tnps in whole genomes was compared to the currently available ISsaga annotations for *A. marina* MBIC11017 and *S. maltophilia* K279a. TnpPred successfully predicted 266 Tnps out of 293 for *A. marina* and 47 Tnps out of 53 for *S. maltophilia.* In addition, TnPred predicted additional loci for Tnps in both genomes that were not recognized by ISsaga and improved the prediction of several Tnps by the assignment of a Family designation to Tnps that were only identified by the general term “Tnps” in ISsaga. Therefore, it is proposed that TnPred could be a useful aid to predict Tnps in microbial genomes.

## 5. Website and FTP

The TnpPred web service of Tnp IS family HMM prediction for aminoacid sequences and the HMM Profiles for 19 Tnp IS families can be accessed at http://www.mobilomics.cl/.

## Supplementary Material

The Supplementary Materials include the following files: Excel file showing each IS amily HMM profile ROC curve, sensitivity and selectivity. *Acaryochloris marina* MBIC11017 uid58167 TnpPred annotation in genbank format (.gbk). *Stenotrophomonas maltophilia* K279a TnpPred annotation in genbank format (.gbk). *Acaryochloris marina* MBIC11017 uid58167 full report of TnpPred predictions. NC_009925.gbk Chromosome. *Stenotrophomonas maltophilia* K279a full report of TnpPred predictions. NC_010943.gbk Chromosome.

## Figures and Tables

**Table 1:** A comparison of the selectivities, sensitivities, and cutoff *e*-values derived from TnpPred versus the corresponding Pfam HMM profiles for 19 IS families.

	IS family	Pfam				TnpPred			
		HMM	Selectivity	Sensitivity	Cutoff^1^	HMM	Selectivity	Sensitivity	Cutoff^1^
		Transposase_27	96.4%	83.2%	1.2*E* + 04	Combined*	100.0%	95.4%	2.0*E* − 06
1	IS1	—	—	—	—	ORF1*	99.9%	100.0%	32*E* − 34
		—	—	—	—	ORF2*	99.4%	100.0%	3.9*E* − 40

2	IS110	Transposase_9	95.3%	94.2%	7.0*E* + 03	ORF1*	100.0%	100.0%	1.3*E* − 63
		Transposase_20	100.0%	99.1%	1.2*E* − 06				

3	IS1380	—	—	—	—	ORF1*	100.0%	100.0%	2.3*E* − 224

4	IS200/IS605	Transposase_17	100.0%	100.0%	3.9*E* − 21	ORF1*	100.0%	100.0%	4.2*E* − 79

		—	—	—	—	Combined*	93.9%	96.0%	*l*.8*E* − 09
5	IS21	—	—	—	—	ORF1*	100.0%	93.7%	84*E* − 10
		IstB_N	72.8%	79.3%	6.7*E* + 04	ORF2*	100.0%	100.0%	2.0*E* − 06
		IstB	76.6%	79.5%	2.6*E* + 04		100.0%	100.0%	2.0*E* − 06

6	IS256	Transposase_mut*	100.0%	100.0%	8.7*E* − 01	ORF1	99.4%	98.8%	3.1*E* − 55

		—	—	—	—	Combined*	99.5%	81.8%	2.7*E* + 02
	IS3_IS150	—	—	—	—	ORF1*	90.3%	69.7%	5.4*E* + 03
		—	—	—	—	ORF2	100.0%	100.0%	2.4*E* − 114
		Transposase_8	80.1%	78.8%	2.5*E* + 04	Combined*	100.0%	100.0%	5.7*E* − 06
	IS3_IS2	—	—	—	—	ORF1*	100.0%	90.0%	1.2*E* − 83
		—	—	—	—	ORF2*	100.0%	100.0%	1.3*E* − 223
		—	—	—	—	Combined*	98.6%	89.6%	3.3*E* + 02
7	IS3_IS3	—	—	—	—	ORF1*	93.6%	76.5%	2.5*E* − 11
		—	—	—	—	ORF2*	100.0%	100.0%	7.9*E* − 140
		—	—	—	—	Combined*	99.9%	100.0%	3.9*E* − 04
	IS3_IS407	—	—	—	—	ORF1*	99.7%	95.8%	3.0*E* − 44
		—	—	—	—	ORF2*	100.0%	100.0%	3.5*E* − 135
		—	—	—	—	Combined*	100.0%	91.4%	5.4*E* − 98
	IS3_IS51	—	—	—	—	ORF1*	87.4%	74.3%	5.5*E* − 24
		—	—	—	—	ORF2	100.0%	100.0%	8.1*E* − 205

8	IS30	—	—	—	—	ORF1*	100.0%	100.0%	1.7*E* − 127

9	IS4	Transposase_11	99.0%	96.0%	9.5*E* + 02	ORF1*	100.0%	96.1%	1.3*E* − 01
		Transposase_Tn5	51.8%	58.9%	1.1*E* + 05				

10	IS481	Mu-transpos_C	67.7%	54.0%	6.7*E* + 04	ORF1*	99.9%	100.0%	4.0*E* − 84

	IS5_IS1031	—	—	—	—	ORF1*	100.0%	100.0%	1.2*E* − 162
	IS5_IS427	—	—	—	—	Combined*	99.7%	97.7%	6.7*E* − 11
11	IS5_IS5	Transposase_33	54.6%	60.4%	1.1*E* + 05	ORF1*	100.0%	100.0%	4.2*E* − 48
	IS5_IS903	—	—	—	—	ORF1*	100.0%	100.0%	7.3*E* − 155
	IS5_ISH1	—	—	—	—	ORF1*	100.0%	100.0%	1.5*E* − 216
	IS5_ISL2		—	—	—	ORF1*	100.0%	100.0%	2.6*E* − 129

12	IS6	—	—	—	—	ORF1*	100.0%	100.0%	4.1*E* − 65

13	IS630	Transposase_14	52.8%	68.2%	9.8*E* + 04	ORF1*	98.4%	97.7%	2.7*E* − 93

		Transposase_34	89.9%	73.1%	2.4*E* − 35	Combined	85.6%	79.0%	1.5*E* + 04
14	IS66	—	—	—	—	ORF1*	97.8%	82.6%	1.4*E* − 32
		—	—	—	—	ORF2*	94.0%	88.4%	3.4*E* + 02
		—	—	—	—	ORF3*	100.0%	88.8%	1.7*E* − 299

15	IS91	Transposase_32	100.0%	100.0%	7.9*E* − 16	ORF1*	100.0%	100.0%	4.1*E* − 216

16	IS982	—	—	—	—	ORF1*	99.3%	99.2%	3.5*E* − 102

17	ISAs1	—	—	—	—	ORF1*	100.0%	100.0%	4.1*E* − 205

18	ISL3	Transposase_12	100.0%	99.0%	4.7*E* − 31	ORF1*	100.0%	99.0%	5.0*E* − 95

19	Tn3	Transposase_7*	100.0%	63.3%	1.7*E* − 156	ORF1*	94.8%	68.3%	0.0*E* + 00

^1^Cutoff *e*-values are derived from ROC charts for each model (Supplementary File 1); *indicates the HMM that was selected for incorporation into the TnpPred web service.

**Table 2:** Summary of Tnp predictions by TnpPred compared to ISsaga.

Organism	*Acaryochloris marina* MBIC11017	*Stenotrophomonas maltophilia* K279a
Kingdom	Bacteria	Bacteria
Class	Acaryochloris	Gammaproteobacteria
Date	May 27, 10	July 9, 10
Accession number	NC_009925	NC_010943
% G + C	47.3%	66.3%
Length (Mbp)	6.5	4.9
Confirmed total	244	46
Class A*	214	42
Class B*	30	4
Class C*	22	1
Not found by TnpPred	27	6
Number of IS families TnpPred	17	10
Total: not found + TnpPred	293	53
Number of IS families ISsaga	17	9
Total TnpPred	266	47
Total ISsaga	272	39

^∗^See Figure 1 for the definition of classes.
