# Supplementary material for: TnpPred: A Web Service for the Robust Prediction of Prokaryotic Transposases
Source: Comp Funct Genomics. 2012 Nov 18;2012:678761. doi: 10.1155/2012/678761 (PMC3506888; doi:10.1155/2012/678761)
Supplement: Supplementary file 2 [file 678761.f2.pdf]

Supplementary file 2
Acaryochloris marina MBIC11017 uid58167 TnpPred annotation in genbank format (.gbk)

```
LOCUS       NC_009925                 0 bp    DNA     linear   UNK
ACCESSION   unknown
COMMENT     This looks like a good IS fragment prediction
FEATURES             Location/Qualifiers
     source          1..6503724
                     /mol_type="genomic DNA"
                     /db_xref="taxon:329726"
                     /organism="Acaryochloris marina MBIC11017"
     CDS             19129..19968
                     /colour="255 0 0"
                     /evidence=predicted
                     /translation="TGVLIT-KIFTNLVSXTSEDYRFIINTKNRXICHFYFYLASKQY
                     PQXSLFMNRtIDYCLSKIIYskqfkiVSIERRSGxsrkqQNHSQFAQLFLXLETLGPG
                     VQRLFEKXVKLSRYXsVNTQTLDRTXRAMPTAYDSDLTTLQWELLEPLIPAAKP-GGR
                     PRT-TDMLSVLNAIFYLVVTGCQWRQLPHDFPCWSTVYSYFRRWRDDGTWVHINEHLR
                     MQERVSE----------------------------DRHPSPSAAICDAQSVKVGNPR
                     CHSIGFDGgkmvKGRKRHVLVDTLGLVL-MVMVTAAN"
                     /note="IS5_IS427   e-value= 4.4e-06 complete sequence hit
                     coverage= 100%, between model( 296 aa) positions 1; 296
                     length is 280aa with 33 gaps, 7 stops, absolute frame=
                     Plus1"
     CDS             19486..20337
                     /colour="255 0 0"
                     /evidence=predicted
                     /translation="TQTLDRTXRAM-----PTAYDSDLTTLQWELLEPLIPAAK----
                     ---PGGRPRTTDMLSVLNAIFYLVVTGCQWRQLPHDFPCWSTVYSYFRRWRDDGTWVH
                     INEHLRMQERVSEDRHPSPSAAICDAQSVKVGNPRCH-SIGFDGGKMVKGRKRHVLVD
                     TLGLVLMVMVTAANISDQR-GAKILFWKARRQGaslSRLVRIWADAGYQGQALMKWVM
                     DRFQYVLEVVKRSDNLAGFQVVSKRWIVERTFGWLLWSRRLNKDYEVLTRTAEALAYV
                     AMIRLMVRRLAQEHXNFSNSLS"
                     /note="IS5_IS1031  e-value= 1.9e-108 complete sequence hit
                     coverage= 100%, between model( 295 aa) positions 1; 295
                     length is 284aa with 14 gaps, 2 stops, absolute frame=
                     Plus1"
     CDS             19528..20337
                     /colour="255 0 0"
                     /evidence=predicted
                     /translation="YDSDLTTLQWELLEPLIPAAK-------PGGRPRTTDMLSVLNA
                     IFYLVVTGCQWRQLPHDFPCWSTVYSYFRRWRDDGTWVHINEHLRMQERVSEDRHPSP
                     SAAICDAQSVKVGNPRCH-SIGFDGGKMVKGRKRHVLVDTLGLVLMVMVTAANISDQR
                     -GAKILFWKARRQGaslSRLVRIWADAGYQGQALMKWVMDRFQYVLEVVKRSDNLAGF
                     QVVSKRWIVERTFGWLLWSRRLNKDYEVLTRTAEALAYVAMIRLMVRRLAQEHXNFSN
                     SLS"
                     /note="IS5_IS1031  e-value= 1.3e-110 fragment hit
                     coverage= 93.56%, between model( 295 aa) positions 20;
                     295 length is 270aa with 9 gaps, 1 stops, absolute frame=
                     Plus1"
     CDS             19537..19776
                     /colour="255 0 0"
                     /evidence=predicted
                     /translation="DLTTLQWELLEPLIPAAKP-GGRPRT-TDMLSVLNAIFYLVVTG
                     CQWRQLPHDFPCWSTVYSYFRRWRDDGTWVHINEHLRM"
                     /note="IS5_IS427   e-value= 3.3e-18 fragment hit coverage=
                     27.70%, between model( 296 aa) positions 125; 206 length
                     is 80aa with 2 gaps, 0 stops, absolute frame= Plus1"
     CDS             57724..58665
```

```
                     /colour="255 0 0"
                     /evidence=predicted
                     /translation="TKNRXPMSYQIRNWSEYNAGLKQRGSVTFWLEQS--AIVHWLET
                     TPRQKRGASLTYSDTAISTFETVKCIYGLAGRQTEGFLNSLFELMGIELPVCDHSTVS
                     RRKGQLSISLPVIPKQGAIHVVIDSTGIKVYG------------EGEWKTRQHGVS-K
                     RRTWRKLHLSVDESTGEILTGVVTTNDVQDGEVFEDLLEGIDD-----EIEQVSAD-G
                     AYDQSHCYDALMERNATAAIPPRKNA-------------KIWQHGNC--NApphpRDQ
                     NLRAIRKQGRKRWKKQAHYHRRSLAETTMFRFKTIFGGQVRSRNFDNQATELLLQCAA
                     LN-RMIQIAKPETVWVE"
                     /note="IS5_IS903   e-value= 9.4e-98 complete sequence hit
                     coverage= 100%, between model( 347 aa) positions 1; 347
                     length is 314aa with 37 gaps, 1 stops, absolute frame=
                     Plus1"
     CDS             57724..58665
                     /colour="255 0 0"
                     /evidence=predicted
                     /translation="TKNRXPMSYQIRNWSEYNAGLKQRGSVTFWLEQS--AIVHWLET
                     TPRQKRGASLTYSDTAISTFETVKCIYGLAGRQTEGFLNSLFELMGIELPVCDHSTVS
                     RRKGQLSISLPVIPKQGAIHVVIDSTGIKVYG------------EGEWKTRQHGVS-K
                     RRTWRKLHLSVDESTGEILTGVVTTNDVQDGEVFEDLLEGIDD-----EIEQVSAD-G
                     AYDQSHCYDALMERNATAAIPPRKNA-------------KIWQHGNC--NApphpRDQ
                     NLRAIRKQGRKRWKKQAHYHRRSLAETTMFRFKTIFGGQVRSRNFDNQATELLLQCAA
                     LN-RMIQIAKPETVWVE"
                     /note="IS5_IS903   e-value= 1.7e-97 complete sequence hit
                     coverage= 100%, between model( 347 aa) positions 1; 347
                     length is 314aa with 37 gaps, 1 stops, absolute frame=
                     Plus1"
     CDS             322582..322968
                     /colour="255 0 0"
                     /evidence=predicted
                     /translation="YADESGMDHRDEYDY-AYGPKGE-----------RVYA-LKTG-
                     RRSGRVNMIAALRAKQL--MAPFTIEGACNRTVFELwLERCLIPvlKPGQKLVI--DN
                     ATFHKGGQIQELVEKAG--CEVWYLPPYSPDLNKIERSWSWIKSRIR"
                     /note="IS630  e-value= 1.1e-13 fragment hit coverage=
                     38.83%, between model( 376 aa) positions 188; 333 length
                     is 129aa with 20 gaps, 0 stops, absolute frame= Plus1"
     CDS             439435..439710
                     /colour="255 0 0"
                     /evidence=predicted
                     /translation="KQQLQLP-VTLARIDRFDLLFLDNVGDVKKLDFETSVLFEXIAH
                     QYKRCSFLIIANYPFSEWDIIFR-DSMMMVVAVDRLVHHALIIEIKAESY"
                     /note="IS21_ORF2   e-value= 4.1e-16 fragment hit coverage=
                     36.43%, between model( 258 aa) positions 148; 241 length
                     is 92aa with 2 gaps, 1 stops, absolute frame= Plus1"
     CDS             470638..470901
                     /colour="255 0 0"
                     /evidence=predicted
                     /translation="FSYLLIQCPLCGHPK-THKHGKTSKGSQRYRCPHCQQTFSATLD
                     TLYYRRqiSSETIQTILQSHAEGSSLRGLSRITGVAFNTCVSVIR"
                     /note="IS1_ORF1    e-value= 4.6e-14 fragment hit coverage=
                     36.40%, between model( 239 aa) positions 1; 87 length is
                     88aa with 1 gaps, 0 stops, absolute frame= Plus1"
     CDS             689482..689622
                     /colour="255 0 0"
                     /evidence=predicted
                     /translation="SEYIHKSHNVSVLLYHLVFPAKYRRALF----DESVDEVLR-NI
                     CMDIENRF"
                     /note="IS200  e-value= 3.9e-06 fragment hit coverage=
                     33.77%, between model( 154 aa) positions 1; 52 length is
                     47aa with 5 gaps, 0 stops, absolute frame= Plus1"
```

```
CDS             689482..689604
                /colour="255 0 0"
                /evidence=predicted
                /translation="SEYIHKSHNVSVLLYHLVFPAKYRRALFDES-----VDEVLRNI
                CM"
                /note="IS200_IS605 e-value= 3.1e-06 fragment hit
                coverage= 30.07%, between model( 153 aa) positions 1; 46
                length is 41aa with 5 gaps, 0 stops, absolute frame=
                Plus1"
CDS             689875..690138
                /colour="255 0 0"
                /evidence=predicted
                /translation="LFYFLMQCPLCGHPK-THKHGKTSKGSQRYRCPHCQQTFS-Etf
                DTLCYRRQiSPETIQTILQAHVEGSSLRGLSRITGVAYNTCVSVVR"
                /note="IS1_ORF1    e-value= 1.7e-15 fragment hit coverage=
                36.40%, between model( 239 aa) positions 1; 87 length is
                88aa with 2 gaps, 0 stops, absolute frame= Plus1"
CDS             850231..851292
                /colour="255 0 0"
                /evidence=predicted
                /translation="IVKRXKN------EQCIVSHpYPALXPPQPLAQG---ISSA---
                --RQXHFXESEQRRQRXSENCSVRASSRPIDAAIRDRKKSLNVLTP--NQKRQMVRQL
                QQDYSIRQICQVLNYPRSQVYYHARGQP-----DESELKAAIAGVAGAYP-TYGYRRI
                TAQLQ---RQGYCVNHKRVARLMRQIGIMAKTKVK-RKRTTNSEHSfpRYGNRVLNLS
                --IDHPEQVWVADITYIRLQ-QEFVYLAVVMDVFTRAIRGWHLSRHIDQQLTLRALNK
                ALERAT---PEIHHS-----DQGVQYAAAAYMQLLQQHQVQISMAEVGQAWQNGYAER
                LMRTIKEEEVD--LSDYRNFTEAYEHIEQFLEdVYMHKRIHSSLGYLTPCEYEQQWRQ
                Q"
                /note="IS3_IS150_ORF2    e-value= 1.7e-22 complete sequence
                hit coverage= 100%, between model( 389 aa) positions 1;
                389 length is 354aa with 39 gaps, 5 stops, absolute
                frame= Plus1"
CDS             850270..851316
                /colour="255 0 0"
                /evidence=predicted
                /translation="HPYPALXPPQ--PLAQGI--SSARQXHFXESEQRRQRXSENCSV
                RASSRPIDAAiRDRKKslnVLTPNQKRQMVRQLQQDY--SIRQICQVLNYPRSQVYYH
                ARGQPDESELKAAIAGVAGAYPTYGYRRITAQLQRQGYCVNHKRVARLMRQIGIMAKT
                KV-KRKRTtnsehsfprYGNRVLNLSIDHPEQVWVADITYIRLQ--QEFVYLAVVmDV
                FTRAIRGWHLSRHIDQQLTLRALNKALE-RATPEIHHSDQGVQYAAAAYMQLLQQHQV
                QISMAEVGQAWQNGYAERLMRTIKEEEVDLSDYRNFTEAYEHIEQFLEDvYMHKRIHS
                SLGYLTPCEYEQQWRQQNNHYCMNK"
                /note="IS3_IS407_ORF2    e-value= 6.3e-13 complete sequence
                hit coverage= 100%, between model( 344 aa) positions 1;
                344 length is 349aa with 10 gaps, 4 stops, absolute
                frame= Plus1"
CDS             850405..851289
                /colour="255 0 0"
                /evidence=predicted
                /translation="PIDAAIRDRKKSLnVLTPNQKRQMVRQLQQDYSIRQICQVLNYP
                RSQVYYHARGQPDESELKAAIAGV-AGAYPTYGYRRITAQLQRQGYCVNHKRVARLMR
                QIGIMAKTKVK----RKRTTNSEHSF-PRYGNRvlNLSIDHPEQVWVADITYIRL--Q
                QEFVYLAVVMDVFTRAIRGWHLSRHIDQQLTLRALNKALER----ATPEIHHSDQGVQ
                YAAAAYMQLLQQHQVQISMAEVGQAWQNGYAERLMRTIKEEEVDLSDYRNFT--EAYE
                HIEQFLEDVYMHKRIHSSLGYLTPCEYEQQWRQ"
                /note="IS3_IS150_ORF1    e-value= 4.3e-49 complete sequence
                hit coverage= 100%, between model( 306 aa) positions 1;
                306 length is 295aa with 14 gaps, 0 stops, absolute
                frame= Plus1"
CDS             850441..851325
```

```
                     /colour="255 0 0"
                     /evidence=predicted
                     /translation="LNVLTPNQKRQMVRQLQQD-YSIRQICQVLNYPRSQVVYYHARGQ
                     PD--------------------ESELKAAIAGVAGAYPTYGYRRITAQLQRQGYC---
                     VNHKRVARLMRQIGIMAKTKVKRKR-TTNSEHSFPRYGnRVLNLSIDHPEQVWVADIT
                     YI-------------RLQQ--------EFVYLAVVMDVFTRAIRGWHLSRH----IDQ
                     QLTLRALNKALERAT-PEIHHSDQGVQYAAA----AYMQLLQQHQVQISMAEVGQAWQ
                     NGYAERLMRTIKEEEVDLSD---YRNFTEAYEHIEQFLEDvYMHKRIHSSLGYLTPCE
                     YEQQWRQQNNHYCMNKEDS"
                     /note="IS481 e-value= 2.9e-08 complete sequence hit
                     coverage= 100%, between model( 351 aa) positions 1; 351
                     length is 295aa with 58 gaps, 0 stops, absolute frame=
                     Plus1"
     CDS             850441..851325
                     /colour="255 0 0"
                     /evidence=predicted
                     /translation="LNVLTPNQK-RQMVRQLQqdYSIRQICQVLNYP---RSQVVYYHA
                     RgQPDESELKA--AIAGV-------AGA---YPTYGYRRITAQLQRQGYCVNHKRVAR
                     LMRQIGIMAKTKVKRKRTTNSEHSFPRYGNRVL-NLSIDHPEQVWVADITYIRLQQEF
                     VYLAVVMDVFTRAIRGWHLSRHIDQQLTLRALNKALERATPE--------IHHSDQGV
                     QYAAAAYMQLLQQHQVQISMAEVGQAWQNGYAERLMRTIKEEEVDLSDYrnFTEAYEH
                     IEQFLEDV--YMHKRIHSSLGYLTPCEYEQQWRQQNNHYCMNKEDS"
                     /note="IS3_IS51_ORF2     e-value= 1.6e-26 complete sequence
                     hit coverage= 100%, between model( 317 aa) positions 1;
                     317 length is 295aa with 27 gaps, 0 stops, absolute
                     frame= Plus1"
     CDS             850447..850701
                     /colour="255 0 0"
                     /evidence=predicted
                     /translation="VLTPNQKRQMVRQLQQDY--SIRQICQVLNYPRSQVVYYHARGQP
                     DESELKAAIAGVAGAYPTYGYRRITAQLQRQGYCVNHKRVARL"
                     /note="IS3_IS407_ORF2    e-value= 1.2e-17 fragment hit
                     coverage= 25.29%, between model( 344 aa) positions 60;
                     146 length is 85aa with 2 gaps, 0 stops, absolute frame=
                     Plus1"
     CDS             850450..851301
                     /colour="255 0 0"
                     /evidence=predicted
                     /translation="LTPNQKRQMVRQL-QQDYSIRQICQVLNYPRSQVVYYHARG----
                     -------QPDESELKAAIAGVAGAYPTYGYRRITAQLQRQGY-----CVNHKRVARLM
                     RQIGIM--AKTKVKRK-RttnsehsfpRYGNRVLnlsIDHPEQVWVAD-ITYIRLQQE
                     FVYLAVVMDVFTRAIRGWH------LSRHIDQQLTLRALNK---ALERATPEIHH-SD
                     QGVQYAAAAYMQLLQQHQVQISMAEVGQAWQNGYAERLMRTIKEEEVDLSDYRNFTEA
                     YEHIEQFLEDvYMHKRIHSSLGYLTPCEYEQQWRQQNNH"
                     /note="IS3_IS2_ORF2      e-value= 2.2e-08 complete sequence
                     hit coverage= 100%, between model( 302 aa) positions 1;
                     302 length is 284aa with 31 gaps, 0 stops, absolute
                     frame= Plus1"
     CDS             850465..851292
                     /colour="255 0 0"
                     /evidence=predicted
                     /translation="KRQMVRQLQQDYSIRQICQVLNYPRSQVVYYHARGQP-----DES
                     ELKAAIAGVAGAYP-TYGYRRITAQLQ---RQGYCVNHKRVARLMRQIGIMAKTKVK-
                     RKRTTNSEHSfpRYGNRVLNLS--IDHPEQVWVADITYIRLQ-QEFVYLAVVMDVFTR
                     AIRGWHLSRHIDQQLTLRALNKALERAT---PEIHHS-----DQGVQYAAAAYMQLLQ
                     QHQVQISMAEVGQAWQNGYAERLMRTIKEEEVD--LSDYRNFTEAYEHIEQFLEdVYM
                     HKRIHSSLGYLTPCEYEQQWRQQ"
                     /note="IS3_IS150_ORF2    e-value= 2.2e-47 fragment hit
                     coverage= 76.09%, between model( 389 aa) positions 94;
                     389 length is 276aa with 23 gaps, 0 stops, absolute
```

frame= Plus1"
CDS 850468..851316
/colour="255 0 0"
/evidence=predicted
/translation="RQMVRQLQQDYSIRQICQVLNYPRSQVYYHA-----------RG
QPDEsELKAAIAGV-AGAYPTYGYRRITAQLQRQGYCVNHKRVARLMRQIGIMAKTKV
KRKRTTNSEHSFPRYGNRVL-NLSIDHPEQVWVADITYIRLQQEFVYLAVVMDVFTRA
IRGWHLSRHIDQQLT-LRALNKALERATPE-------IHHSDQGVQYAAAAYMQLLQQ
HQVQISMAEVGQAWQNGYAERLMRTIKEEEVDL---S-DYRNFTEAYEHIEQFLEDVY
MHKRIHSSLGYLTPCEYEQQWRQQNNHYCMNK"
/note="IS3_IS51_ORF1 e-value= 6.3e-51 complete sequence
hit coverage= 100%, between model(307 aa) positions 1;
307 length is 283aa with 25 gaps, 0 stops, absolute
frame= Plus1"
CDS 850468..851316
/colour="255 0 0"
/evidence=predicted
/translation="RQMVRQLQQDYSIRQICQVLNYPRSQVYYHA-----------RG
QPDEsELKAAIAGV-AGAYPTYGYRRITAQLQRQGYCVNHKRVARLMRQIGIMAKTKV
KRKRTTNSEHSFPRYGNRVL-NLSIDHPEQVWVADITYIRLQQEFVYLAVVMDVFTRA
IRGWHLSRHIDQQLT-LRALNKALERATPE-------IHHSDQGVQYAAAAYMQLLQQ
HQVQISMAEVGQAWQNGYAERLMRTIKEEEVDL---S-DYRNFTEAYEHIEQFLEDVY
MHKRIHSSLGYLTPCEYEQQWRQQNNHYCMNK"
/note="IS3_IS51_ORF1 e-value= 8.1e-53 complete sequence
hit coverage= 100%, between model(307 aa) positions 1;
307 length is 283aa with 25 gaps, 0 stops, absolute
frame= Plus1"
CDS 850471..851286
/colour="255 0 0"
/evidence=predicted
/translation="QMVRQLQQDYSIRQICQVLNYPRSQVYYHARG--QPD-----ES
ELKAAI-AGVAGAYPTYGYRRITAQLQ-RQGYCVNHKRVARLMRQIGIMAKTKVKRKR
TTNSEHSfPRYGNRVLNLS-IDHPEQVWVADITYIRLQQEFVYLAVVMDVFTRAIRGW
HLSRHIDQQLTLRALNKALERA-TPE--IHHSDQGVQYAAAAYMQLLQQHQVQISMAE
VGQAWQNGYAERLMRTIK-EEEVDL-SDYRNFTEAYEHIEQFLEDVYMHKRIHSSLGY
LTPCEYEQQWR"
/note="IS3_IS3_ORF1 e-value= 9.5e-54 complete sequence
hit coverage= 100%, between model(286 aa) positions 1;
286 length is 272aa with 15 gaps, 0 stops, absolute
frame= Plus1"
CDS 850471..851286
/colour="255 0 0"
/evidence=predicted
/translation="QMVRQLQQDYSIRQICQVLNYPRSQVYYHARG--QPD-----ES
ELKAAI-AGVAGAYPTYGYRRITAQLQ-RQGYCVNHKRVARLMRQIGIMAKTKVKRKR
TTNSEHSfPRYGNRVLNLS-IDHPEQVWVADITYIRLQQEFVYLAVVMDVFTRAIRGW
HLSRHIDQQLTLRALNKALERA-TPE--IHHSDQGVQYAAAAYMQLLQQHQVQISMAE
VGQAWQNGYAERLMRTIK-EEEVDL-SDYRNFTEAYEHIEQFLEDVYMHKRIHSSLGY
LTPCEYEQQWR"
/note="IS3_IS3_ORF1 e-value= 2.5e-54 complete sequence
hit coverage= 100%, between model(286 aa) positions 1;
286 length is 272aa with 15 gaps, 0 stops, absolute
frame= Plus1"
CDS 850474..851289
/colour="255 0 0"
/evidence=predicted
/translation="MVRQLQQDYSIRQICQVLNYPRSQVYYHARGQPDESELKAAIAG
V-AGAYPTYGYRRITAQLQRQGYCVNHKRVARLMRQIGIMAKTKVK----RKRTTNSE
HSF-PRYGNRvlNLSIDHPEQVWVADITYIRL--QQEFVYLAVVMDVFTRAIRGWHLS
RHIDQQLTLRALNKALER----ATPEIHHSDQGVQYAAAAYMQLLQQHQVQISMAEVG

QAWQNGYAERLMRTIKEEEVDLSDYRNFT--EAYEHIEQFLEDVYMHKRIHSSLGYLT
PCEYEQQWRQ"
/note="IS3_IS150_ORF1   e-value= 6.3e-50 fragment hit
coverage= 92.81%, between model(306 aa) positions 23;
306 length is 272aa with 14 gaps, 0 stops, absolute
frame= Plus1"

CDS 850483..851298
/colour="255 0 0"
/evidence=predicted
/translation="QLQQDYSIRQICQVLNYPRSQVYYHARGQPDE---------SEL
KAAIAGVAGaypTYGYRRITAQLQRQGYCVNHKRVARLMRQIGIMAKTKVKRKRTTNS
EHSFPRY---GNRVLNLSIDHPEQVWVADITYIRLQQEFVYLAVVMDVFTRAIRGWHL
SRHIDQQLTLRALNKALER----ATPE---IHHSDQGVQYAAAAYMQLLQQHQVQISM
AEVGQAWQNGYAERLMRTIKEEEVDLSDYRNFTEAYEHIEQFLEDVYMHKRIHSSLGY
LTPCEYEQQWRQQNN"
/note="IS3_IS3_ORF2   e-value= 1.2e-64 complete sequence
hit coverage= 100%, between model(288 aa) positions 1;
288 length is 272aa with 19 gaps, 0 stops, absolute
frame= Plus1"

CDS 850483..851298
/colour="255 0 0"
/evidence=predicted
/translation="QLQQDYSIRQICQVLNYPRSQVYYHARGQPDE---------SEL
KAAIAGVAGaypTYGYRRITAQLQRQGYCVNHKRVARLMRQIGIMAKTKVKRKRTTNS
EHSFPRY---GNRVLNLSIDHPEQVWVADITYIRLQQEFVYLAVVMDVFTRAIRGWHL
SRHIDQQLTLRALNKALER----ATPE---IHHSDQGVQYAAAAYMQLLQQHQVQISM
AEVGQAWQNGYAERLMRTIKEEEVDLSDYRNFTEAYEHIEQFLEDVYMHKRIHSSLGY
LTPCEYEQQWRQQNN"
/note="IS3_IS3_ORF2   e-value= 3.3e-65 complete sequence
hit coverage= 100%, between model(288 aa) positions 1;
288 length is 272aa with 19 gaps, 0 stops, absolute
frame= Plus1"

CDS 850552..850710
/colour="255 0 0"
/evidence=predicted
/translation="YHARgQPDESELKAAIAGVAGAYPTYGYRRITAQLQRQGY----
-CVNHKRVARLMRQ"
/note="IS3_IS2_ORF2   e-value= 9.1e-09 fragment hit
coverage= 18.87%, between model(302 aa) positions 48;
104 length is 53aa with 5 gaps, 0 stops, absolute frame=
Plus1"

CDS 850618..851301
/colour="255 0 0"
/evidence=predicted
/translation="YPTYGYRRITAQLQRQGYCVNHKRVARLMRQIGIMAKTKVKRKR
TTNSEHSFPRYGNRVL-NLSIDHPEQVWVADITYIRLQQEFVYLAVVMDVFTRAIRGW
HLSRHIDQQLTLRALNKALERATPE--------IHHSDQGVQYAAAAYMQLLQQHQVQ
ISMAEVGQAWQNGYAERLMRTIKEEEVDLSDYrnFTEAYEHIEQFLEDV--YMHKRIH
SSLGYLTPCEYEQQWRQQNNH"
/note="IS3_IS51_ORF2   e-value= 1.9e-38 fragment hit
coverage= 74.76%, between model(317 aa) positions 73;
309 length is 228aa with 11 gaps, 0 stops, absolute
frame= Plus1"

CDS 943111..944052
/colour="255 0 0"
/evidence=predicted
/translation="TKNRXPMSYQIRNWSEYNAGLKQRGSVTFWLEQS--AIVHWLET
TPRQKRGASLTYSDTAISTFETVKCIYGLAGRQTEGFLNSLFELMGIELPVCDHSTVS
RRKGQLSISLPVIPKQGAIHVVIDSTGIKVYG------------EGEWKTRQHGVS-K
RRTWRKLHLSVDESTGEILTGVVTTNDVQDGEVFEDLLEGIDD-----EIEQVSAD-G

```
                     AYDQSHCYDALMERNATAAIPPRKNA-------------KIWQHGNC--NApphpRDQ
                     NLRAIRKQGRKRWKKQAHYHRRSLAETTMFRFKTIFGGQVRSRNFDNQATELLLQCAA
                     LN-RMIQIAKPETVWVE"
                     /note="IS5_IS903   e-value= 9.4e-98 complete sequence hit
                     coverage= 100%, between model( 347 aa) positions 1; 347
                     length is 314aa with 37 gaps, 1 stops, absolute frame=
                     Plus1"
     CDS             943111..944052
                     /colour="255 0 0"
                     /evidence=predicted
                     /translation="TKNRXPMSYQIRNWSEYNAGLKQRGSVTFWLEQS--AIVHWLET
                     TPRQKRGASLTYSDTAISTFETVKCIYGLAGRQTEGFLNSLFELMGIELPVCDHSTVS
                     RRKGQLSISLPVIPKQGAIHVVIDSTGIKVYG------------EGEWKTRQHGVS-K
                     RRTWRKLHLSVDESTGEILTGVVTTNDVQDGEVFEDLLEGIDD-----EIEQVSAD-G
                     AYDQSHCYDALMERNATAAIPPRKNA-------------KIWQHGNC--NApphpRDQ
                     NLRAIRKQGRKRWKKQAHYHRRSLAETTMFRFKTIFGGQVRSRNFDNQATELLLQCAA
                     LN-RMIQIAKPETVWVE"
                     /note="IS5_IS903   e-value= 1.7e-97 complete sequence hit
                     coverage= 100%, between model( 347 aa) positions 1; 347
                     length is 314aa with 37 gaps, 1 stops, absolute frame=
                     Plus1"
     CDS             1300873..1301259
                     /colour="255 0 0"
                     /evidence=predicted
                     /translation="YADESGMDHRDEYDY-AYGPKGE-----------RVYA-LKTG-
                     RRSGRVNMIAALRAKQL--MAPFTIEGACNRTVFELwLERCLIPvlKPGQKLVI--DN
                     ATFHKGGQIQELVEKAG--CEVWYLPPYSPDLNKIERSWSWIKSRIR"
                     /note="IS630  e-value= 1.1e-13 fragment hit coverage=
                     38.83%, between model( 376 aa) positions 188; 333 length
                     is 129aa with 20 gaps, 0 stops, absolute frame= Plus1"
     CDS             1334545..1335405
                     /colour="255 0 0"
                     /evidence=predicted
                     /translation="IQVELRGSIHXplNLKMVRHPYDTDLTLDQWELLRPLLPKAK--
                     -----SGGRPRRVNLLAVVNAIFYLLRSGCPWRLIPHDFPAWQTVYTYFRQWEADGTW
                     ERLNRALREKLRIQAGRHRHPSAACVDSQSVKTAGAAQE--KGFDGGKKVKGRKRTIL
                     VDTMGLLIGATVHSARRSDHQ-GMILLGLWFSAFWQSIQLIWTDQTFGGQAFTAWFQK
                     TFGWTMEVVKRPEEQQGFEVLPRRWVVERTFAWFGHYRRLSKDYEYLPTTSEMMLYAA
                     MVNLMLKRLAXIFSNTLLEAV"
                     /note="IS5_IS1031  e-value= 2.7e-109 complete sequence hit
                     coverage= 100%, between model( 295 aa) positions 1; 295
                     length is 287aa with 10 gaps, 2 stops, absolute frame=
                     Plus1"
     CDS             1334545..1335384
                     /colour="255 0 0"
                     /evidence=predicted
                     /translation="IQVELRGSIHXPLNLKMVRHPYDTDLTLDQWELLRPLLPKAKSG
                     ------------------------GRPRRVNLLAVVNAIFYLLRSGCPWRL--IPHD
                     FPAWQTVYTYFRQWEADGTWERLNRALREKLRIQAG---RHRHPSAACVDSQSVKTAG
                     A---------------------------------------------------------
                     ----------------------------------------AQEKGFDGGKKVKGRKRT
                     ILVDTMGLLIGA---------------TVHSARRSDHQGMILLGLWFSAFWQSIQLIW
                     TDQTFGGQAFTAWFQKTFGWTMEVVKRPEEQQGFEVLPRRWVVERTFAWFGHYRRLSK
                     DYEYLPTTSEMMLYAAMVNLMLKRLAXIFS"
                     /note="IS5_IS5     e-value= 6.1e-15 complete sequence hit
                     coverage= 100%, between model( 422 aa) positions 1; 422
                     length is 280aa with 142 gaps, 2 stops, absolute frame=
                     Plus1"
     CDS             1334599..1335378
                     /colour="255 0 0"
```

```
                     /evidence=predicted
                     /translation="RHPYDTDLTLDQWELLRPLLPKAK-------SGGRPRRVNLLAV
                     VNAIFYLLRSGCPWRLIPHDFPAWQTVYTYFRQWEADGTWERLNRALREKLRIQAGRH
                     RHPSAACVDSQSVKTAGAAQE--KGFDGGKKVKGRKRTILVDTMGLLIGATVHSARRS
                     DHQ-GMILLGLWFSAFWQSIQLIWTDQTFGGQAFTAWFQKTFGWTMEVVKRPEEQQGF
                     EVLPRRWVVERTFAWFGHYRRLSKDYEYLPTTSEMMLYAAMVNLMLKRLAXI"
                     /note="IS5_IS1031  e-value= 3.5e-110 fragment hit
                     coverage= 91.53%, between model( 295 aa) positions 17;
                     286 length is 260aa with 10 gaps, 1 stops, absolute
                     frame= Plus1"
     CDS             1334629..1334862
                     /colour="255 0 0"
                     /evidence=predicted
                     /translation="DQWELLRPLLP--KAKSGGRPRRVNLLAVVNAIFYLLRSGCPWR
                     LIPHDFPAWQTVYTYFRQWEADGTWERLNRALREKL"
                     /note="IS5_IS427   e-value= 7.9e-16 fragment hit coverage=
                     27.03%, between model( 296 aa) positions 129; 208 length
                     is 78aa with 2 gaps, 0 stops, absolute frame= Plus1"
     CDS             1334704..1334931
                     /colour="255 0 0"
                     /evidence=predicted
                     /translation="AVVNAIFYLLRSGCPWRL--IPHDFPAWQTVYTYFRQWEADGTW
                     ERLNRALREKLRIQAG---RHRHPSAACVDSQSVKTA"
                     /note="IS5_IS5     e-value= 7.9e-09 fragment hit coverage=
                     19.19%, between model( 422 aa) positions 79; 159 length
                     is 76aa with 5 gaps, 0 stops, absolute frame= Plus1"
     CDS             1334932..1335384
                     /colour="255 0 0"
                     /evidence=predicted
                     /translation="GAAQEKGFDGGKKVKGRKRTILVDTMGLLIGA------------
                     ---TVHSARRSDHQGMILLGLWFSAFWQSIQLIWTDQTFGGQAFTAWFQKTFGWTMEV
                     VKRPEEQQGFEVLPRRWVVERTFAWFGHYRRLSKDYEYLPTTSEMMLYAAMVNLMLKR
                     LAXIFS"
                     /note="IS5_IS5     e-value= 1.2e-15 fragment hit coverage=
                     39.34%, between model( 422 aa) positions 257; 422 length
                     is 151aa with 15 gaps, 1 stops, absolute frame= Plus1"
     CDS             1474789..1475553
                     /colour="255 0 0"
                     /evidence=predicted
                     /translation="-----TGVYKQTFkrMLHAWHTHHLGRSN--AGRPPKLCRPDQL
                     LVALQYWREYRTYFHIAGDWEVSESTVCRIVHQVETALMNSGLFRLPGQKSLLQGFER
                     PDVVVMDVTETPIERPQKRQKAYYSGKKRDIPSNARLSLTAT--LXRLSV--------
                     XALAQAVGMIFrssrfqvSISIQIPRVCKIADIKGLQPT--TPTAM----FLSRSHNT
                     VNXLPCSESITVlxvknEWAlntlNRSLKIFRILSERYRNRRRRYTLRCNLIAALYNH
                     EL"
                     /note="IS5_ISL2    e-value= 2.2e-49 complete sequence hit
                     coverage= 100%, between model( 260 aa) positions 1; 260
                     length is 255aa with 23 gaps, 3 stops, absolute frame=
                     Plus1"
     CDS             1474852..1475160
                     /colour="255 0 0"
                     /evidence=predicted
                     /translation="RSNAGRPPKLCRPDQLLVALQYWREYRTYFHIAGDWEVSESTVC
                     RIVHQVETALMNSGLFRLPGQKSLLQGFERPDVVVMDVTETPIERPQKRQKAYYSGKK
                     R"
                     /note="IS5_ISL2    e-value= 1.5e-44 fragment hit coverage=
                     39.62%, between model( 260 aa) positions 27; 129 length
                     is 103aa with 0 gaps, 0 stops, absolute frame= Plus1"
     CDS             1475446..1475553
                     /colour="255 0 0"
```

```
                     /evidence=predicted
                     /translation="NRSLKIFRILSERYRNRRRRYTLRCNLIAALYNHEL"
                     /note="IS5_ISL2    e-value= 1.1e-16 fragment hit coverage=
                     13.85%, between model( 260 aa) positions 225; 260 length
                     is 36aa with 0 gaps, 0 stops, absolute frame= Plus1"
     CDS             1499707..1499970
                     /colour="255 0 0"
                     /evidence=predicted
                     /translation="LFYFLMQCPLCGHPK-THKHGKTSKGSQRYRCPHCQQTFS-Etf
                     DTLCYRRQiSPETIQTILQAHVEGSSLRGLSRITGVAYNTCVSVVR"
                     /note="IS1_ORF1    e-value= 1.7e-15 fragment hit coverage=
                     36.40%, between model( 239 aa) positions 1; 87 length is
                     88aa with 2 gaps, 0 stops, absolute frame= Plus1"
     CDS             1585042..1585257
                     /colour="255 0 0"
                     /evidence=predicted
                     /translation="REIHEAIGVSIRTIERVRMRFVEEG---LEAAINQRsGAGRKRK
                     IQGEQEAHLIALRCSEPPVG----HARWTLRLLAD"
                     /note="IS630  e-value= 9.9e-08 fragment hit coverage=
                     20.74%, between model( 376 aa) positions 44; 121 length
                     is 72aa with 7 gaps, 0 stops, absolute frame= Plus1"
     CDS             1602370..1602585
                     /colour="255 0 0"
                     /evidence=predicted
                     /translation="REIHEAIGVSIRTIERVRMRFVEEG---LEAAINQRsGAGRKRK
                     IQGEQEAHLIALRCSEPPVG----HARWTLRLLAD"
                     /note="IS630  e-value= 9.9e-08 fragment hit coverage=
                     20.74%, between model( 376 aa) positions 44; 121 length
                     is 72aa with 7 gaps, 0 stops, absolute frame= Plus1"
     CDS             2373475..2374293
                     /colour="255 0 0"
                     /evidence=predicted
                     /translation="-IEYPQFLQLLEQAELKHNERKaeiexhksRVNAK-GGGRKPLL
                     SVAEEVCLCLFYLRHYPTFEVLGLQFGVSKSEANDTVHYWLSMLRVLL-----PASLL
                     EQVDSNPsdyAIVqewltqfqlIVDTFEQARERPIdnDEQRSYFSGKKQQHTFKSQIV
                     TLPGGKDIVDAIAGkKGPTSDISVFREHQSQFAPGQGFDGDKAYVGAQNVQ----TPH
                     KKPRGKELTPKQKAQNKKfSSTRRIFVEHTIRLVRIFRVAQERFRLRHDTYEQVILTV
                     CGLVRLRL"
                     /note="IS5_ISL2    e-value= 1.6e-11 complete sequence hit
                     coverage= 100%, between model( 260 aa) positions 1; 260
                     length is 273aa with 11 gaps, 0 stops, absolute frame=
                     Plus1"
     CDS             2373877..2374236
                     /colour="255 0 0"
                     /evidence=predicted
                     /translation="QRSYFSGKKQQHTFKSQIVTLPGGKDIVDAIAGkKGPTSDISVF
                     REHQSQFAPGQGFDGDKAYVGAQNVQ----TPHKKPRGKELTPKQKAQNKKfSSTRRI
                     FVEHTIRLVRIFRVAQERFRLR"
                     /note="IS5_ISL2    e-value= 8.7e-11 fragment hit coverage=
                     46.92%, between model( 260 aa) positions 120; 241 length
                     is 120aa with 4 gaps, 0 stops, absolute frame= Plus1"
     CDS             2559835..2560116
                     /colour="255 0 0"
                     /evidence=predicted
                     /translation="SMECPYCQSEK-ILKRGFDSlqdgTLVQRYQCKDCNRRFN-ERT
                     GTPMARLRtasSVVSYAIKARTEGMGVRSAGRTFGKSHTTIMRWEKRLADQA"
                     /note="IS1_ORF1    e-value= 3.2e-11 fragment hit coverage=
                     37.24%, between model( 239 aa) positions 5; 93 length is
                     94aa with 2 gaps, 0 stops, absolute frame= Plus1"
     CDS             2630473..2630631
```

```
                     /colour="255 0 0"
                     /evidence=predicted
                     /translation="HHPHPRVQRRMEVLYLKSQGLPHAQICQLCQISRPTLAKTLRLY
                     QQGGIEGLK"
                     /note="IS481 e-value= 1.1e-08 fragment hit coverage=
                     15.10%, between model( 351 aa) positions 1; 53 length is
                     53aa with 0 gaps, 0 stops, absolute frame= Plus1"
     CDS             2982304..2983041
                     /colour="255 0 0"
                     /evidence=predicted
                     /translation="-----TGVYKQTF-KRMLHAWHTYHlSHSNAERPPKLCRPDQLL
                     VALQYWREYRTYFHIAGDWDVSESTVCRIVQQVESALMNSGLFRLPGKKHLIQGFERP
                     DVVVMDVTETPIERPQKGQKAYYSGKKRDIPSNARLSLTVALXRLSALT---------
                     -LAKVVGMIFrssrfqvSTSIQIprVCKIADiKGLQPI-----TPIAM----FLSRSH
                     NTVNXLPSSESTTVLXVRNEWALNTLIAAXRFSEFCRSAIVIVVVATRCGVTX"
                     /note="IS5_ISL2    e-value= 2.8e-20 complete sequence hit
                     coverage= 100%, between model( 260 aa) positions 1; 260
                     length is 246aa with 25 gaps, 5 stops, absolute frame=
                     Plus1"
     CDS             2982364..2982675
                     /colour="255 0 0"
                     /evidence=predicted
                     /translation="SHSNAERPPKLCRPDQLLVALQYWREYRTYFHIAGDWDVSESTV
                     CRIVQQVESALMNSGLFRLPGKKHLIQGFERPDVVVMDVTETPIERPQKGQKAYYSGK
                     KR"
                     /note="IS5_ISL2    e-value= 1.3e-41 fragment hit coverage=
                     40%, between model( 260 aa) positions 26; 129 length is
                     104aa with 0 gaps, 0 stops, absolute frame= Plus1"
     CDS             3974686..3975072
                     /colour="255 0 0"
                     /evidence=predicted
                     /translation="YADESGMDHRDEYDY-AYGPKGE-----------RVYA-LKTG-
                     RRSGRVNMIAALRAKQL--MAPFTIEGACNRTVFELwLERCLIPvlKPGQKLVI--DN
                     ATFHKGGQIQELVEKAG--CEVWYLPPYSPDLNKIERSWSWIKSRIR"
                     /note="IS630 e-value= 1.1e-13 fragment hit coverage=
                     38.83%, between model( 376 aa) positions 188; 333 length
                     is 129aa with 20 gaps, 0 stops, absolute frame= Plus1"
     CDS             3975325..3976020
                     /colour="255 0 0"
                     /evidence=predicted
                     /translation="FKHLHLGMIsDIKRKSLPAIARAVGLENHQSLHHFLSESPWVAS
                     hLSDkRLDIILKILDGRRLILLID------------------ETGDCKKGKSTDYVKR
                     QYIGNVGKKENGIvAVTAYGLFQGMILPLSFEVYKPR---------ErLKEEDEYLSK
                     PQIAAGMIRQLQGMGFCFELVLADSLYGEAQTNFVNVLEELKLPYILAIRSNHAVWLP
                     AEQAVFAEPWQSFERTFSNGTTETRYrQEIIYGqrhhQKRY"
                     /note="IS4   e-value= 4.7e-14 fragment hit coverage=
                     51.23%, between model( 486 aa) positions 1; 249 length is
                     232aa with 27 gaps, 0 stops, absolute frame= Plus1"
     CDS             3975325..3976452
                     /colour="255 0 0"
                     /evidence=predicted
                     /translation="FKHLHLGMIsDIKRKSLPAIARAVGLENHQSLHHFLSESPWVAS
                     hLSDkRLDIILKILDGRRLILLID------------------ETGDCKKGKSTDYVKR
                     QYIGNVGKKENGIvAVTAYGLFQGMILPLSFEVYKPR---------ErLKEEDEYLSK
                     PQIAAGMIRQLQGMGFCFELVLADSLYGEAQTNFVNVLEELKLPYILAIRSNHAVWLP
                     AEQAVFAEPWQSFERTFSNGT--------------------------------TETRYR
                     QEIIYGQRHHQKRyWLLTTDPQTLPENSTS-----------------------YVMAA
                     APE----IKLDEIGDCYGFRTWIEYGLKQSKD-TLGWADFRMTHYEQIEKWWEIVMSA
                     FLMVSLFADVFNDS-------------------------CPVAHQHFAQHPwWDNQNG
                     WKNLLNNVR--LIIQPLIS----WNLLKRWLEVFPSRALKKGF"
```

/note="IS4   e-value= 9.9e-07 complete sequence hit coverage= 100%, between model(486 aa) positions 1; 486 length is 376aa with 117 gaps, 0 stops, absolute frame= Plus1"
CDS 4217206..4217592
/colour="255 0 0"
/evidence=predicted
/translation="YADESGMDHRDEYDY-AYGPKGE-----------RVYA-LKTG-RRSGRVNMIAALRAKQL--MAPFTIEGACNRTVFELwLERCLIPvlKPGQKLVI--DNATFHKGGQIQELVEKAG--CEVWYLPPYSPDLNKIERSWSWIKSRIR"
/note="IS630 e-value= 1.1e-13 fragment hit coverage= 38.83%, between model(376 aa) positions 188; 333 length is 129aa with 20 gaps, 0 stops, absolute frame= Plus1"
CDS 4225801..4226796
/colour="255 0 0"
/evidence=predicted
/translation="CIAVAILRRTXTREVFHSFCSVMPKIYSYdlRCKVIDAIeLDGMRPSEASELFHIRRNTINQWQHLKAETG-----DLHPKP-VHCPDHSHKIK--------DWDKFRAFAYKHRHKTQAQMAQLWDGE----ISERTISRALQNIGFT-RKKTYGYR--ERD--EHKR----AAFIKRLSTVDP-DDIVYADESGMDHRDEYDY-AYGPKGE-----------RVYA-LKSG-TRKGRVNMIAALRAKQL--MAPFTIEGACNRTVFEvwlERCLIPMLKPGQKLVI--DNATFHKAGRIQELVEKAG--CEVWYLPPYSPDLNKIERSWSWIK--SRIRHQLDHFGSLREAMEHVLHLTSXIEWRLLXNKLCNNLKRIIL"
/note="IS630 e-value= 1.2e-10 complete sequence hit coverage= 100%, between model(376 aa) positions 1; 376 length is 332aa with 50 gaps, 3 stops, absolute frame= Plus1"
CDS 4226083..4226673
/colour="255 0 0"
/evidence=predicted
/translation="AFAYKHRHKTQAQMAQLWDGEISERTISRALQNIGFT-RKKTYGYR--ERD--EHKR----AAFIKRLSTVDP-DDIVYADESGMDHRDEYDY-AYGPKGE-----------RVYA-LKSG-TRKGRVNMIAALRAKQL--MAPFTIEGACNRTVFEvwlERCLIPMLKPGQKLVI--DNATFHKAGRIQELVEKAG--CEVWYLPPYSPDLNKIERSWSWIKSRIR"
/note="IS630 e-value= 1.2e-14 fragment hit coverage= 59.57%, between model(376 aa) positions 110; 333 length is 197aa with 30 gaps, 0 stops, absolute frame= Plus1"
CDS 4227730..4228050
/colour="255 0 0"
/evidence=predicted
/translation="FPDAQQVHVVLDNLNTHtpaalyKTFKPDEALRILS-RIQFHYTPKHGSWLNMVEFEFSALSRQCLNRRIP-DIEKLRHEVTAWEQRRNWDKAMVNWLFTVDDARTKLS"
/note="IS630 e-value= 3.3e-07 fragment hit coverage= 27.39%, between model(376 aa) positions 274; 376 length is 107aa with 2 gaps, 0 stops, absolute frame= Plus1"
CDS 5176546..5176935
/colour="255 0 0"
/evidence=predicted
/translation="FFMDESGFSLVPCIPY-GWQPIG-------------TYL-EIPTRSSKRLNVLGFLSRRQGL--HAYTSEQTITSEVVShCIDTFFADV--ELPTVIVVDQAPIHTSQsiyEMKAEWAERG--ITLFELPSYSPHLNLIERLWQFMKYQWI"
/note="IS630 e-value= 6.5e-11 fragment hit coverage= 39.10%, between model(376 aa) positions 187; 333 length is 130aa with 21 gaps, 0 stops, absolute frame= Plus1"
CDS 5240533..5240919
/colour="255 0 0"
/evidence=predicted
/translation="YADESGMEHRDEYDY-AYGPKGE-----------RAYA-LKSG-

RRSGRVNMIAALRAKQL--MAPFTIEGACNRTVFEIWLercLIPMLKPGQKLVI--DN
ATFHKGGQIQELVEAAG--CEVWYLPPYSPDLNKIERSWSWIKSRIR"
/note="IS630 e-value= 4e-14 fragment hit coverage= 38.83%, between model(376 aa) positions 188; 333 length is 129aa with 20 gaps, 0 stops, absolute frame= Plus1"

CDS 5297623..5297775
/colour="255 0 0"
/evidence=predicted
/translation="QLCLILDNNPTHKG-KMRSQLAIHLEqmgltqsiQVEFLYLPSY
SPKLNLVE"
/note="IS630 e-value= 4.3e-06 fragment hit coverage= 11.70%, between model(376 aa) positions 279; 322 length is 51aa with 1 gaps, 0 stops, absolute frame= Plus1"

CDS 5359111..5359329
/colour="255 0 0"
/evidence=predicted
/translation="RIRFVYIPKHTSWLNQIECWFSILVRRLIRRGNFTSKDDLQQRI
LEFIEYFNHtMAKPFQWQFKGFQPRLXLM"
/note="IS630 e-value= 4e-10 fragment hit coverage= 19.15%, between model(376 aa) positions 305; 376 length is 73aa with 0 gaps, 1 stops, absolute frame= Plus1"

CDS 5359159..5359269
/colour="255 0 0"
/evidence=predicted
/translation="IECWFSILVRRLIR--RGNFTSKDDLQQRILEFIEYFNH"
/note="IS3_IS150_ORF2 e-value= 2e-08 fragment hit coverage= 10.03%, between model(389 aa) positions 329; 367 length is 37aa with 2 gaps, 0 stops, absolute frame= Plus1"

CDS 5666503..5666847
/colour="255 0 0"
/evidence=predicted
/translation="CGNANIMXYLY--------------RNIEKGEYMVELGgfrRYF
YELARHKESVIEEGHLMPDHVHKLLSIPPKYSVSNVVGYVKGKVPFISPESXA-----
--------------IVGMRQAI------SFGLEDILC-QQSAKMRKXSVDISG"
/note="IS605 e-value= 9.1e-06 complete sequence hit coverage= 100%, between model(152 aa) positions 1; 152 length is 115aa with 40 gaps, 3 stops, absolute frame= Plus1"

CDS 5666590..5666721
/colour="255 0 0"
/evidence=predicted
/translation="FYELARHKESVIEEGHLMPDHVHKLLSIPPKYSVSNVVGYVKGK
"
/note="IS200 e-value= 1.3e-14 fragment hit coverage= 28.57%, between model(154 aa) positions 41; 84 length is 44aa with 0 gaps, 0 stops, absolute frame= Plus1"

CDS 5666590..5666721
/colour="255 0 0"
/evidence=predicted
/translation="FYELARHKESVIEEGHLMPDHVHKLLSIPPKYSVSNVVGYVKGK
"
/note="IS200_IS605 e-value= 1.4e-14 fragment hit coverage= 28.76%, between model(153 aa) positions 41; 84 length is 44aa with 0 gaps, 0 stops, absolute frame= Plus1"

CDS 5666629..5666721
/colour="255 0 0"
/evidence=predicted
/translation="EGHLMPDHVHKLLSIPPKYSVSNVVGYVKGK"

/note="IS605 e-value= 2.2e-10 fragment hit coverage= 20.39%, between model(152 aa) positions 54; 84 length is 31aa with 0 gaps, 0 stops, absolute frame= Plus1"
CDS 5747743..5747880
/colour="255 0 0"
/evidence=predicted
/translation="WQTDGWEGYARQLPDE---VVHEVSKALTQRLERTNGIVRQQTG
RWHRR"
/note="IS1_ORF2 e-value= 1.8e-11 fragment hit coverage= 20.68%, between model(237 aa) positions 164; 212 length is 46aa with 3 gaps, 0 stops, absolute frame= Plus1"
CDS 5903662..5904156
/colour="255 0 0"
/evidence=predicted
/translation="LEKAGIESKKKTYGYR--ERD--ETQR---QEFIERLKTKHA--
HQIVYVDEAGIDNRADYPY-GYGPIG-----------QRFYD-LKSG-KRTERVSFIA
ALKEGQL--FSPMTFEGSCNWLLFEAWLQQSLIPQLQPgdIIVI-DNASFHHGLSIEE
IVAEAG--CEIWYLPSYSPDLNKIERWWFVLKN"
/note="IS630 e-value= 4e-13 fragment hit coverage= 50.80%, between model(376 aa) positions 140; 330 length is 165aa with 28 gaps, 0 stops, absolute frame= Plus1"
CDS 6055453..6056127
/colour="255 0 0"
/evidence=predicted
/translation="SXGICMKCPECGGQH-IHKNGHRR-GKQNHICVTCGRQFL--ST
YSKRGYSDWTKRLCLRMYVNGMGLKGIERVIGVAHTTVIHWVQQVGVLLPDAYDPDD-
-----IP---QVGELDELETFVGKKRNKVWIW-TVVDHFHPG--ILGWVVGDHSAETF
RPLWqaIVCWQCFFWVSDGNPVYpgFIPEgDQIVSKTYMTRVEGENTRLRHYLARLHR
KTLCYSKSVDMLKHSIRLL---IHYL"
/note="IS1_ORF1 e-value= 5.8e-42 complete sequence hit coverage= 100%, between model(239 aa) positions 1; 239 length is 225aa with 19 gaps, 1 stops, absolute frame= Plus1"
CDS 6055453..6056127
/colour="255 0 0"
/evidence=predicted
/translation="SXGICMKCPECGGQH-IHKNGHRR-GKQNHICVTCGRQFL--ST
YSKRGYSDWTKRLCLRMYVNGMGLKGIERVIGVAHTTVIHWVQQVGVLLPDAYDPDD-
-----IP---QVGELDELETFVGKKRNKVWIW-TVVDHFHPG--ILGWVVGDHSAETF
RPLWqaIVCWQCFFWVSDGNPVYpgFIPEgDQIVSKTYMTRVEGENTRLRHYLARLHR
KTLCYSKSVDMLKHSIRLL---IHYL"
/note="IS1_ORF1 e-value= 3.3e-41 complete sequence hit coverage= 100%, between model(239 aa) positions 1; 239 length is 225aa with 19 gaps, 1 stops, absolute frame= Plus1"
CDS 6055453..6056133
/colour="255 0 0"
/evidence=predicted
/translation="SXGICMKCPECGGQH-IHKNGHRR-GKQNHICVTCGR----QFL
STYSKRGysdwTKRLCLRMYVNGMGLKGIERVIGVAHTTVIHWVQQV--GVLLPDAYD
PDDIPQVGELDELETFVGKKRNKVWIWTVVDHFHPGILGWVVGDHSAETFRPLWQAIV
CWQ-CFFWVSDGNPVYPGFIPEG----DQIVSKTYMTRVEGENTRLRHYLARLHRKTL
CYSKSVDMLKHSIRLLI-HYLKF"
/note="IS1_ORF2 e-value= 3.9e-29 complete sequence hit coverage= 100%, between model(237 aa) positions 1; 237 length is 227aa with 14 gaps, 1 stops, absolute frame= Plus1"
CDS 6055732..6056133
/colour="255 0 0"
/evidence=predicted

```
                     /translation="DPDDIPQVGELDELETFVGKKRNKVWIWTVVDHFHPGILGWVVG
                     DHSAETFRPLWQAIVCWQ-CFFWVSDGNPVYPGFIPEG----DQIVSKTYMTRVEGEN
                     TRLRHYLARLHRKTLCYSKSVDMLKHSIRLLI-HYLKF"
                     /note="IS1_ORF2    e-value= 8.3e-32 fragment hit coverage=
                     59.07%, between model( 237 aa) positions 98; 237 length
                     is 134aa with 6 gaps, 0 stops, absolute frame= Plus1"
     CDS             6108877..6109140
                     /colour="255 0 0"
                     /evidence=predicted
                     /translation="LFYFLMQCPLCGHPK-THKHGKTSKGSQRYRCPHCQQTFS-Etf
                     DTLCYRRQiSPETIQTILQAHVEGSSLRGLSRITGVAYNTCVSVVR"
                     /note="IS1_ORF1    e-value= 1.7e-15 fragment hit coverage=
                     36.40%, between model( 239 aa) positions 1; 87 length is
                     88aa with 2 gaps, 0 stops, absolute frame= Plus1"
     CDS             6307891..6308655
                     /colour="255 0 0"
                     /evidence=predicted
                     /translation="SPYQHLSLYLKKLRLSHMLTHWESIESQA-MQENWSYAEFLLAL
                     CETKAQRREQARLKRALTEARLPNA-KSFTNFDFSHCPQLNPAPLMQLAAdPGWLERA
                     ENCLILGPSGVGKTHLATGVSKKMLEFGKRVKFFAANALVQHLQQAKLQLQLH-PMLK
                     KLDRYDLLVLDDLGYCKKSEAETSVLFELIAHRYERKSLLITANQPFSQWDDIFT-DS
                     MMAVAAIDRLIHHGLIIKIQADSYRRKSATQRTAQTQSPPQ"
                     /note="IS21_ORF2   e-value= 7.2e-80 complete sequence hit
                     coverage= 100%, between model( 258 aa) positions 1; 258
                     length is 255aa with 4 gaps, 0 stops, absolute frame=
                     Plus1"
     CDS             6307891..6308655
                     /colour="255 0 0"
                     /evidence=predicted
                     /translation="SPYQHLSLYLKKLRLSHMLTHWESIESQA-MQENWSYAEFLLAL
                     CETKAQRREQARLKRALTEARLPNA-KSFTNFDFSHCPQLNPAPLMQLAAdPGWLERA
                     ENCLILGPSGVGKTHLATGVSKKMLEFGKRVKFFAANALVQHLQQAKLQLQLH-PMLK
                     KLDRYDLLVLDDLGYCKKSEAETSVLFELIAHRYERKSLLITANQPFSQWDDIFT-DS
                     MMAVAAIDRLIHHGLIIKIQADSYRRKSATQRTAQTQSPPQ"
                     /note="IS21_ORF2   e-value= 1.9e-80 complete sequence hit
                     coverage= 100%, between model( 258 aa) positions 1; 258
                     length is 255aa with 4 gaps, 0 stops, absolute frame=
                     Plus1"
     CDS             6335869..6336906
                     /colour="255 0 0"
                     /evidence=predicted
                     /translation="PMIKIDFTAEEIQQLNYERYHHPHPRVQRRMEVLYLKSQGLSHT
                     QICQLCQISRPTLAKTLRQYQQGG---IEGLKTLEYKGQPSLLNAHS-DSVRAYFEQH
                     PP---------RTSAEAQAVIEQLTGIKRSPTQIKAFLKRIGCRYRKVGYVPGKSSLP
                     -EKIEEQEQFRHTRLEPLleeAQRQERLVFFVDAAHFVHRAYLGF-VWCITRI-----
                     ---------FIPSPS-GRQRFNVLGALDAITKDLVSVTNHTYINSHSMCLLLAKLALL
                     DPV-IPISVILDNARYQKCQLVTDFAEIVD--IELVYLPSYSPHLNLIERLWRFVRKE
                     CLYSKYYADFPAFKGAIQQCIDQCNGEHKAKLTTLLSLKFQSFKK"
                     /note="IS630 e-value= 4.9e-24 complete sequence hit
                     coverage= 100%, between model( 376 aa) positions 1; 376
                     length is 346aa with 33 gaps, 0 stops, absolute frame=
                     Plus1"
     CDS             6335869..6336906
                     /colour="255 0 0"
                     /evidence=predicted
                     /translation="PMIKIDFTAEEIQQLNYERYHHPHPRVQRRMEVLYLKSQGLSHT
                     QICQLCQISRPTLAKTLRQYQQGG---IEGLKTLEYKGQPSLLNAHS-DSVRAYFEQH
                     PP---------RTSAEAQAVIEQLTGIKRSPTQIKAFLKRIGCRYRKVGYVPGKSSLP
                     -EKIEEQEQFRHTRLEPLleeAQRQERLVFFVDAAHFVHRAYLGF-VWCITRI-----
                     ---------FIPSPS-GRQRFNVLGALDAITKDLVSVTNHTYINSHSMCLLLAKLALL
```

```
                     DPV-IPISVILDNARYQKCQLVTDFAEIVD--IELVYLPSYSPHLNLIERLWRFVRKE
                     CLYSKYYADFPAFKGAIQQCIDQCNGEHKAKLTTLLSLKFQSFKK"
                     /note="IS630 e-value= 6.7e-25 complete sequence hit
                     coverage= 100%, between model( 376 aa) positions 1; 376
                     length is 346aa with 33 gaps, 0 stops, absolute frame=
                     Plus1"
     CDS             6335929..6336087
                     /colour="255 0 0"
                     /evidence=predicted
                     /translation="HHPHPRVQRRMEVLYLKSQGLSHTQICQLCQISRPTLAKTLRQY
                     QQGGIEGLK"
                     /note="IS481 e-value= 3.7e-09 fragment hit coverage=
                     15.10%, between model( 351 aa) positions 1; 53 length is
                     53aa with 0 gaps, 0 stops, absolute frame= Plus1"
     CDS             6483169..6483501
                     /colour="255 0 0"
                     /evidence=predicted
                     /translation="QRVKDRAAMIRLSHQGMYVEKIAALFQCNVRTARQTFHRWQQKG
                     ---LGGLWDAPHPGAQRRWHPED-IEYLETCLRQEERTY-------NSQQLSRKLAKE
                     RNVHLSADRIRQILKKRGXS"
                     /note="IS630 e-value= 6e-07 fragment hit coverage=
                     32.45%, between model( 376 aa) positions 25; 146 length
                     is 111aa with 11 gaps, 1 stops, absolute frame= Plus1"
     CDS             59159..60220
                     /colour="255 0 0"
                     /evidence=predicted
                     /translation="IVKRXKN------EQCIVSHpYPALXPPQPLAQG---ISSA---
                     --RQXHFXESEQRRQRXSENCSVRASSRPIDAAIRDRKKSLNVLTP--NQKRQMVRQL
                     QQDYSIRQICQVLNYPRSQVYYHARGQP-----DESELKAAIAGVAGAYP-TYGYRRI
                     TAQLQ---RQGYCVNHKRVARLMRQIGIMAKTKVK-RKRTTNSEHSfpRYGNRVLNLS
                     --IDHPEQVWVADITYIRLQ-QEFVYLAVVMDVFTRAIRGWHLSRHIDQQLTLRALNK
                     ALERAT---PEIHHS-----DQGVQYAAAAYMQLLQQHQVQISMAEVGQAWQNGYAER
                     LMRTIKEEEVD--LSDYRNFTEAYEHIEQFLEdVYMHKRIHSSLGYLTPCEYEQQWRQ
                     Q"
                     /note="IS3_IS150_ORF2    e-value= 1.7e-22 complete sequence
                     hit coverage= 100%, between model( 389 aa) positions 1;
                     389 length is 354aa with 39 gaps, 5 stops, absolute
                     frame= Plus2"
     CDS             59198..60244
                     /colour="255 0 0"
                     /evidence=predicted
                     /translation="HPYPALXPPQ--PLAQGI--SSARQXHFXESEQRRQRXSENCSV
                     RASSRPIDAAiRDRKKslnVLTPNQKRQMVRQLQQDY--SIRQICQVLNYPRSQVYYH
                     ARGQPDESELKAAIAGVAGAYPTYGYRRITAQLQRQGYCVNHKRVARLMRQIGIMAKT
                     KV-KRKRTtnsehsfprYGNRVLNLSIDHPEQVWVADITYIRLQ--QEFVYLAVVmDV
                     FTRAIRGWHLSRHIDQQLTLRALNKALE-RATPEIHHSDQGVQYAAAAYMQLLQQHQV
                     QISMAEVGQAWQNGYAERLMRTIKEEEVDLSDYRNFTEAYEHIEQFLEDvYMHKRIHS
                     SLGYLTPCEYEQQWRQQNNHYCMNK"
                     /note="IS3_IS407_ORF2    e-value= 6.3e-13 complete sequence
                     hit coverage= 100%, between model( 344 aa) positions 1;
                     344 length is 349aa with 10 gaps, 4 stops, absolute
                     frame= Plus2"
     CDS             59333..60217
                     /colour="255 0 0"
                     /evidence=predicted
                     /translation="PIDAAIRDRKKSLnVLTPNQKRQMVRQLQQDYSIRQICQVLNYP
                     RSQVYYHARGQPDESELKAAIAGV-AGAYPTYGYRRITAQLQRQGYCVNHKRVARLMR
                     QIGIMAKTKVK----RKRTTNSEHSF-PRYGNRvlNLSIDHPEQVWVADITYIRL--Q
                     QEFVYLAVVMDVFTRAIRGWHLSRHIDQQLTLRALNKALER----ATPEIHHSDQGVQ
                     YAAAAYMQLLQQHQVQISMAEVGQAWQNGYAERLMRTIKEEEVDLSDYRNFT--EAYE
```

HIEQFLEDVYMHKRIHSSLGYLTPCEYEQQWRQ"
/note="IS3_IS150_ORF1   e-value= 4.3e-49 complete sequence hit coverage= 100%, between model(306 aa) positions 1; 306 length is 295aa with 14 gaps, 0 stops, absolute frame= Plus2"

CDS 59369..60253
/colour="255 0 0"
/evidence=predicted
/translation="LNVLTPNQKRQMVRQLQQD-YSIRQICQVLNYPRSQVYYHARGQ
PD--------------------ESELKAAIAGVAGAYPTYGYRRITAQLQRQGYC---
VNHKRVARLMRQIGIMAKTKVKRKR-TTNSEHSFPRYGnRVLNLSIDHPEQVWVADIT
YI-------------RLQQ---------EFVYLAVVMDVFTRAIRGWHLSRH----IDQ
QLTLRALNKALERAT-PEIHHSDQGVQYAAA----AYMQLLQQHQVQISMAEVGQAWQ
NGYAERLMRTIKEEEVDLSD---YRNFTEAYEHIEQFLEDvYMHKRIHSSLGYLTPCE
YEQQWRQQNNHYCMNKEDS"
/note="IS481 e-value= 2.9e-08 complete sequence hit coverage= 100%, between model(351 aa) positions 1; 351 length is 295aa with 58 gaps, 0 stops, absolute frame= Plus2"

CDS 59369..60253
/colour="255 0 0"
/evidence=predicted
/translation="LNVLTPNQK-RQMVRQLQqdYSIRQICQVLNYP---RSQVYYHA
RgQPDESELKA--AIAGV-------AGA---YPTYGYRRITAQLQRQGYCVNHKRVAR
LMRQIGIMAKTKVKRKRTTNSEHSFPRYGNRVL-NLSIDHPEQVWVADITYIRLQQEF
VYLAVVMDVFTRAIRGWHLSRHIDQQLTLRALNKALERATPE--------IHHSDQGV
QYAAAAYMQLLQQHQVQISMAEVGQAWQNGYAERLMRTIKEEEVDLSDYrnFTEAYEH
IEQFLEDV--YMHKRIHSSLGYLTPCEYEQQWRQQNNHYCMNKEDS"
/note="IS3_IS51_ORF2   e-value= 1.6e-26 complete sequence hit coverage= 100%, between model(317 aa) positions 1; 317 length is 295aa with 27 gaps, 0 stops, absolute frame= Plus2"

CDS 59375..59629
/colour="255 0 0"
/evidence=predicted
/translation="VLTPNQKRQMVRQLQQDY--SIRQICQVLNYPRSQVYYHARGQP
DESELKAAIAGVAGAYPTYGYRRITAQLQRQGYCVNHKRVARL"
/note="IS3_IS407_ORF2   e-value= 1.2e-17 fragment hit coverage= 25.29%, between model(344 aa) positions 60; 146 length is 85aa with 2 gaps, 0 stops, absolute frame= Plus2"

CDS 59378..60229
/colour="255 0 0"
/evidence=predicted
/translation="LTPNQKRQMVRQL-QQDYSIRQICQVLNYPRSQVYYHARG----
-------QPDESELKAAIAGVAGAYPTYGYRRITAQLQRQGY-----CVNHKRVARLM
RQIGIM--AKTKVKRK-RttnsehsfpRYGNRVLnlsIDHPEQVWVAD-ITYIRLQQE
FVYLAVVMDVFTRAIRGWH------LSRHIDQQLTLRALNK---ALERATPEIHH-SD
QGVQYAAAAYMQLLQQHQVQISMAEVGQAWQNGYAERLMRTIKEEEVDLSDYRNFTEA
YEHIEQFLEDvYMHKRIHSSLGYLTPCEYEQQWRQQNNH"
/note="IS3_IS2_ORF2   e-value= 2.2e-08 complete sequence hit coverage= 100%, between model(302 aa) positions 1; 302 length is 284aa with 31 gaps, 0 stops, absolute frame= Plus2"

CDS 59393..60220
/colour="255 0 0"
/evidence=predicted
/translation="KRQMVRQLQQDYSIRQICQVLNYPRSQVYYHARGQP-----DES
ELKAAIAGVAGAYP-TYGYRRITAQLQ---RQGYCVNHKRVARLMRQIGIMAKTKVK-
RKRTTNSEHSfpRYGNRVLNLS--IDHPEQVWVADITYIRLQ-QEFVYLAVVMDVFTR

AIRGWHLSRHIDQQLTLRALNKALERAT---PEIHHS-----DQGVQYAAAAYMQLLQ
QHQVQISMAEVGQAWQNGYAERLMRTIKEEEVD--LSDYRNFTEAYEHIEQFLEdVYM
HKRIHSSLGYLTPCEYEQQWRQQ"
/note="IS3_IS150_ORF2 e-value= 2.2e-47 fragment hit
coverage= 76.09%, between model(389 aa) positions 94;
389 length is 276aa with 23 gaps, 0 stops, absolute
frame= Plus2"
CDS 59396..60244
/colour="255 0 0"
/evidence=predicted
/translation="RQMVRQLQQDYSIRQICQVLNYPRSQVYYHA-----------RG
QPDEsELKAAIAGV-AGAYPTYGYRRITAQLQRQGYCVNHKRVARLMRQIGIMAKTKV
KRKRTTNSEHSFPRYGNRVL-NLSIDHPEQVWVADITYIRLQQEFVYLAVVMDVFTRA
IRGWHLSRHIDQQLT-LRALNKALERATPE-------IHHSDQGVQYAAAAYMQLLQQ
HQVQISMAEVGQAWQNGYAERLMRTIKEEEVDL---S-DYRNFTEAYEHIEQFLEDVY
MHKRIHSSLGYLTPCEYEQQWRQQNNHYCMNK"
/note="IS3_IS51_ORF1 e-value= 8.1e-53 complete sequence
hit coverage= 100%, between model(307 aa) positions 1;
307 length is 283aa with 25 gaps, 0 stops, absolute
frame= Plus2"
CDS 59396..60244
/colour="255 0 0"
/evidence=predicted
/translation="RQMVRQLQQDYSIRQICQVLNYPRSQVYYHA-----------RG
QPDEsELKAAIAGV-AGAYPTYGYRRITAQLQRQGYCVNHKRVARLMRQIGIMAKTKV
KRKRTTNSEHSFPRYGNRVL-NLSIDHPEQVWVADITYIRLQQEFVYLAVVMDVFTRA
IRGWHLSRHIDQQLT-LRALNKALERATPE-------IHHSDQGVQYAAAAYMQLLQQ
HQVQISMAEVGQAWQNGYAERLMRTIKEEEVDL---S-DYRNFTEAYEHIEQFLEDVY
MHKRIHSSLGYLTPCEYEQQWRQQNNHYCMNK"
/note="IS3_IS51_ORF1 e-value= 6.3e-51 complete sequence
hit coverage= 100%, between model(307 aa) positions 1;
307 length is 283aa with 25 gaps, 0 stops, absolute
frame= Plus2"
CDS 59399..60214
/colour="255 0 0"
/evidence=predicted
/translation="QMVRQLQQDYSIRQICQVLNYPRSQVYYHARG--QPD-----ES
ELKAAI-AGVAGAYPTYGYRRITAQLQ-RQGYCVNHKRVARLMRQIGIMAKTKVKRKR
TTNSEHSfPRYGNRVLNLS-IDHPEQVWVADITYIRLQQEFVYLAVVMDVFTRAIRGW
HLSRHIDQQLTLRALNKALERA-TPE--IHHSDQGVQYAAAAYMQLLQQHQVQISMAE
VGQAWQNGYAERLMRTIK-EEEVDL-SDYRNFTEAYEHIEQFLEDVYMHKRIHSSLGY
LTPCEYEQQWR"
/note="IS3_IS3_ORF1 e-value= 9.5e-54 complete sequence
hit coverage= 100%, between model(286 aa) positions 1;
286 length is 272aa with 15 gaps, 0 stops, absolute
frame= Plus2"
CDS 59399..60214
/colour="255 0 0"
/evidence=predicted
/translation="QMVRQLQQDYSIRQICQVLNYPRSQVYYHARG--QPD-----ES
ELKAAI-AGVAGAYPTYGYRRITAQLQ-RQGYCVNHKRVARLMRQIGIMAKTKVKRKR
TTNSEHSfPRYGNRVLNLS-IDHPEQVWVADITYIRLQQEFVYLAVVMDVFTRAIRGW
HLSRHIDQQLTLRALNKALERA-TPE--IHHSDQGVQYAAAAYMQLLQQHQVQISMAE
VGQAWQNGYAERLMRTIK-EEEVDL-SDYRNFTEAYEHIEQFLEDVYMHKRIHSSLGY
LTPCEYEQQWR"
/note="IS3_IS3_ORF1 e-value= 2.5e-54 complete sequence
hit coverage= 100%, between model(286 aa) positions 1;
286 length is 272aa with 15 gaps, 0 stops, absolute
frame= Plus2"
CDS 59402..60217

```
                     /colour="255 0 0"
                     /evidence=predicted
                     /translation="MVRQLQQDYSIRQICQVLNYPRSQVYYHARGQPDESELKAAIAG
                     V-AGAYPTYGYRRITAQLQRQGYCVNHKRVARLMRQIGIMAKTKVK----RKRTTNSE
                     HSF-PRYGNRvlNLSIDHPEQVWVADITYIRL--QQEFVYLAVVMDVFTRAIRGWHLS
                     RHIDQQLTLRALNKALER----ATPEIHHSDQGVQYAAAAYMQLLQQHQVQISMAEVG
                     QAWQNGYAERLMRTIKEEEVDLSDYRNFT--EAYEHIEQFLEDVYMHKRIHSSLGYLT
                     PCEYEQQWRQ"
                     /note="IS3_IS150_ORF1    e-value= 6.3e-50 fragment hit
                     coverage= 92.81%, between model( 306 aa) positions 23;
                     306 length is 272aa with 14 gaps, 0 stops, absolute
                     frame= Plus2"
CDS                  59411..60226
                     /colour="255 0 0"
                     /evidence=predicted
                     /translation="QLQQDYSIRQICQVLNYPRSQVYYHARGQPDE---------SEL
                     KAAIAGVAGaypTYGYRRITAQLQRQGYCVNHKRVARLMRQIGIMAKTKVKRKRTTNS
                     EHSFPRY---GNRVLNLSIDHPEQVWVADITYIRLQQEFVYLAVVMDVFTRAIRGWHL
                     SRHIDQQLTLRALNKALER----ATPE---IHHSDQGVQYAAAAYMQLLQQHQVQISM
                     AEVGQAWQNGYAERLMRTIKEEEVDLSDYRNFTEAYEHIEQFLEDVYMHKRIHSSLGY
                     LTPCEYEQQWRQQNN"
                     /note="IS3_IS3_ORF2      e-value= 1.2e-64 complete sequence
                     hit coverage= 100%, between model( 288 aa) positions 1;
                     288 length is 272aa with 19 gaps, 0 stops, absolute
                     frame= Plus2"
CDS                  59411..60226
                     /colour="255 0 0"
                     /evidence=predicted
                     /translation="QLQQDYSIRQICQVLNYPRSQVYYHARGQPDE---------SEL
                     KAAIAGVAGaypTYGYRRITAQLQRQGYCVNHKRVARLMRQIGIMAKTKVKRKRTTNS
                     EHSFPRY---GNRVLNLSIDHPEQVWVADITYIRLQQEFVYLAVVMDVFTRAIRGWHL
                     SRHIDQQLTLRALNKALER----ATPE---IHHSDQGVQYAAAAYMQLLQQHQVQISM
                     AEVGQAWQNGYAERLMRTIKEEEVDLSDYRNFTEAYEHIEQFLEDVYMHKRIHSSLGY
                     LTPCEYEQQWRQQNN"
                     /note="IS3_IS3_ORF2      e-value= 3.3e-65 complete sequence
                     hit coverage= 100%, between model( 288 aa) positions 1;
                     288 length is 272aa with 19 gaps, 0 stops, absolute
                     frame= Plus2"
CDS                  59480..59638
                     /colour="255 0 0"
                     /evidence=predicted
                     /translation="YHARgQPDESELKAAIAGVAGAYPTYGYRRITAQLQRQGY----
                     -CVNHKRVARLMRQ"
                     /note="IS3_IS2_ORF2      e-value= 9.1e-09 fragment hit
                     coverage= 18.87%, between model( 302 aa) positions 48;
                     104 length is 53aa with 5 gaps, 0 stops, absolute frame=
                     Plus2"
CDS                  59546..60229
                     /colour="255 0 0"
                     /evidence=predicted
                     /translation="YPTYGYRRITAQLQRQGYCVNHKRVARLMRQIGIMAKTKVKRKR
                     TTNSEHSFPRYGNRVL-NLSIDHPEQVWVADITYIRLQQEFVYLAVVMDVFTRAIRGW
                     HLSRHIDQQLTLRALNKALERATPE--------IHHSDQGVQYAAAAYMQLLQQHQVQ
                     ISMAEVGQAWQNGYAERLMRTIKEEEVDLSDYrnFTEAYEHIEQFLEDV--YMHKRIH
                     SSLGYLTPCEYEQQWRQQNNH"
                     /note="IS3_IS51_ORF2     e-value= 1.9e-38 fragment hit
                     coverage= 74.76%, between model( 317 aa) positions 73;
                     309 length is 228aa with 11 gaps, 0 stops, absolute
                     frame= Plus2"
CDS                  86624..87475
```

/colour="255 0 0"
/evidence=predicted
/translation="TQTLDRTXRAM-----PTAYDSDLTTLQWELLEPLIPAAK-------PGGRPRTTDMLSVLNAIFYLVVTGCQWRQLPHDFPCWSTVYSYFRRWRDDGTWVHINEHLRMQERVSEDRHPSPSAAICDAQSVKVGNPRCH-SIGFDGGKMVKGRKRHVLVDTLGLVLMVMVTAANISDQR-GAKILFWKARRQGaslSRLVRIWADAGYQGQALMKWVMDRFQYVLEVVKRSDNLAGFQVVSKRWIVERTFGWLLWSRRLNKDYEVLTRTAEALAYVAMIRLMVRRLAQEHXNFSNSLS"
/note="IS5_IS1031 e-value= 1.9e-108 complete sequence hit coverage= 100%, between model(295 aa) positions 1; 295 length is 284aa with 14 gaps, 2 stops, absolute frame= Plus2"

CDS 86666..87475
/colour="255 0 0"
/evidence=predicted
/translation="YDSDLTTLQWELLEPLIPAAK-------PGGRPRTTDMLSVLNAIFYLVVTGCQWRQLPHDFPCWSTVYSYFRRWRDDGTWVHINEHLRMQERVSEDRHPSPSAAICDAQSVKVGNPRCH-SIGFDGGKMVKGRKRHVLVDTLGLVLMVMVTAANISDQR-GAKILFWKARRQGaslSRLVRIWADAGYQGQALMKWVMDRFQYVLEVVKRSDNLAGFQVVSKRWIVERTFGWLLWSRRLNKDYEVLTRTAEALAYVAMIRLMVRRLAQEHXNFSNSLS"
/note="IS5_IS1031 e-value= 1.3e-110 fragment hit coverage= 93.56%, between model(295 aa) positions 20; 295 length is 270aa with 9 gaps, 1 stops, absolute frame= Plus2"

CDS 86675..86914
/colour="255 0 0"
/evidence=predicted
/translation="DLTTLQWELLEPLIPAAKP-GGRPRT-TDMLSVLNAIFYLVVTGCQWRQLPHDFPCWSTVYSYFRRWRDDGTWVHINEHLRM"
/note="IS5_IS427   e-value= 3.3e-18 fragment hit coverage= 27.70%, between model(296 aa) positions 125; 206 length is 80aa with 2 gaps, 0 stops, absolute frame= Plus2"

CDS 104411..105622
/colour="255 0 0"
/evidence=predicted
/translation="DELIGENPQPEdiLGESGLLKRLSKRLVERALAGELTHHLQQSSNDQS-GDeNSNGPRNSRNGYSKKTVQSEQG-EMDLSIPRDRCG-EFEPVLVPKGQRRIA-GLDEKIIALYARGMTTRDIRAQLVELYG--ANISEALISDVTNSVMEEVKDWRSRPLDEV--YPIVYLDALYVNIKVNGQVSKRAVYVALAVTVEGNKELLGLWIGAAerEGAKFWLSVLTDLKNRGTQDILIACCDGLKGFPQAIESVYPQTQVQVCIVHLIRNSLRHVPWKESRAVAADLKPIYQAATLE-ESEAALDAFAH-KWDETYPGISQIWIRHWDNVAPLFQYPMAIRKVIYTTNAIESLNRSLRKVIKTKAVFPNEESVYKLMFLAMRNISKR-WTRPILNWKAALSHFAILFPTRFNYXIH"
/note="IS256 e-value= 1.4e-166 complete sequence hit coverage= 100%, between model(410 aa) positions 1; 410 length is 404aa with 11 gaps, 1 stops, absolute frame= Plus2"

CDS 104411..105622
/colour="255 0 0"
/evidence=predicted
/translation="DELIGENPQPEdiLGESGLLKRLSKRLVERALAGELTHHLQQSSNDQS-GDeNSNGPRNSRNGYSKKTVQSEQG-EMDLSIPRDRCG-EFEPVLVPKGQRRIA-GLDEKIIALYARGMTTRDIRAQLVELYG--ANISEALISDVTNSVMEEVKDWRSRPLDEV--YPIVYLDALYVNIKVNGQVSKRAVYVALAVTVEGNKELLGLWIGAAerEGAKFWLSVLTDLKNRGTQDILIACCDGLKGFPQAIESVYPQTQVQVCIVHLIRNSLRHVPWKESRAVAADLKPIYQAATLE-ESEAALDAFAH-KWDETYPGISQIWIRHWDNVAPLFQYPMAIRKVIYTTNAIESLNRSLRKVIKTKAVFPNEESVYKLMFLAMRNISKR-WTRPILNWKAALSHFAILFPTRFNYXIH"
/note="IS256 e-value= 3.7e-167 complete sequence hit

```
                     coverage= 100%, between model( 410 aa) positions 1; 410
                     length is 404aa with 11 gaps, 1 stops, absolute frame=
                     Plus2"
     CDS             159026..160063
                     /colour="255 0 0"
                     /evidence=predicted
                     /translation="PMIKIDFTAEEIQQLNYERYHHPHPRVQRRMEVLYLKSQGLSHT
                     QICQLCQISRPTLAKTLRQYQQGG---IEGLKTLEYKGQPSLLNAHS-DSVRAYFEQH
                     PP---------RTSAEAQAVIEQLTGIKRSPTQIKAFLKRIGCRYRKVGYVPGKSSLP
                     -EKIEEQEQFRHTRLEPLleeAQRQERLVFFVDAAHFVHRAYLGF-VWCITRI-----
                     ---------FIPSPS-GRQRFNVLGALDAITKDLVSVTNHTYINSHSMCLLLAKLALL
                     DPV-IPISVILDNARYQKCQLVTDFAEIVD--IELVYLPSYSPHLNLIERLWRFVRKE
                     CLYSKYYADFPAFKGAIQQCIDQCNGEHKAKLTTLLSLKFQSFKK"
                     /note="IS630 e-value= 6.7e-25 complete sequence hit
                     coverage= 100%, between model( 376 aa) positions 1; 376
                     length is 346aa with 33 gaps, 0 stops, absolute frame=
                     Plus2"
     CDS             159026..160063
                     /colour="255 0 0"
                     /evidence=predicted
                     /translation="PMIKIDFTAEEIQQLNYERYHHPHPRVQRRMEVLYLKSQGLSHT
                     QICQLCQISRPTLAKTLRQYQQGG---IEGLKTLEYKGQPSLLNAHS-DSVRAYFEQH
                     PP---------RTSAEAQAVIEQLTGIKRSPTQIKAFLKRIGCRYRKVGYVPGKSSLP
                     -EKIEEQEQFRHTRLEPLleeAQRQERLVFFVDAAHFVHRAYLGF-VWCITRI-----
                     ---------FIPSPS-GRQRFNVLGALDAITKDLVSVTNHTYINSHSMCLLLAKLALL
                     DPV-IPISVILDNARYQKCQLVTDFAEIVD--IELVYLPSYSPHLNLIERLWRFVRKE
                     CLYSKYYADFPAFKGAIQQCIDQCNGEHKAKLTTLLSLKFQSFKK"
                     /note="IS630 e-value= 4.9e-24 complete sequence hit
                     coverage= 100%, between model( 376 aa) positions 1; 376
                     length is 346aa with 33 gaps, 0 stops, absolute frame=
                     Plus2"
     CDS             159086..159244
                     /colour="255 0 0"
                     /evidence=predicted
                     /translation="HHPHPRVQRRMEVLYLKSQGLSHTQICQLCQISRPTLAKTLRQY
                     QQGGIEGLK"
                     /note="IS481 e-value= 3.7e-09 fragment hit coverage=
                     15.10%, between model( 351 aa) positions 1; 53 length is
                     53aa with 0 gaps, 0 stops, absolute frame= Plus2"
     CDS             249134..249991
                     /colour="255 0 0"
                     /evidence=predicted
                     /translation="CRISDRNLRAM-----STAYDSDLTAEQWELLEPLIPAAK----
                     ---PGGRPRTTDMLSVVNAIFYLVVTGCQWRQLPHDFHCWSTVYSYFRKWRDDGTWRQ
                     INEHLRMQVRVSEDRHPSPSAAICDAQSVKVGNPRCQ-SIGFDGGKLIKGRKRHVLVD
                     TLGLILMVVVTAANISDQR-GAKILFWKARRQGAslgRLVRIWADAGYQGEALMQWVM
                     DRFRYILEVIKRSDNLAGFKVVPKRWIVERTFGWLLWSRRLNKDYEVLTRTAEALVYV
                     AMIRLMVRRLAQEyxTFQTASKMW"
                     /note="IS5_IS1031  e-value= 6.8e-110 complete sequence hit
                     coverage= 100%, between model( 295 aa) positions 1; 295
                     length is 286aa with 14 gaps, 0 stops, absolute frame=
                     Plus2"
     CDS             249164..249424
                     /colour="255 0 0"
                     /evidence=predicted
                     /translation="MSTAYDSDLTAEQWELLEPLIPAAKP-GGRPRT-TDMLSVVNAI
                     FYLVVTGCQWRQLPHDFHCWSTVYSYFRKWRDDGTWRQINEHLRM"
                     /note="IS5_IS427   e-value= 1e-22 fragment hit coverage=
                     30.07%, between model( 296 aa) positions 118; 206 length
                     is 87aa with 2 gaps, 0 stops, absolute frame= Plus2"
```

```
CDS             249176..249991
                /colour="255 0 0"
                /evidence=predicted
                /translation="YDSDLTAEQWELLEPLIPAAK-------PGGRPRTTDMLSVVNA
                IFYLVVTGCQWRQLPHDFHCWSTVYSYFRKWRDDGTWRQINEHLRMQVRVSEDRHPSP
                SAAICDAQSVKVGNPRCQ-SIGFDGGKLIKGRKRHVLVDTLGLILMVVVTAANISDQR
                -GAKILFWKARRQGAslgRLVRIWADAGYQGEALMQWVMDRFRYILEVIKRSDNLAGF
                KVVPKRWIVERTFGWLLWSRRLNKDYEVLTRTAEALVYVAMIRLMVRRLAQEyxTFQT
                ASKMW"
                /note="IS5_IS1031  e-value= 1.4e-111 fragment hit
                coverage= 93.56%, between model( 295 aa) positions 20;
                295 length is 272aa with 9 gaps, 0 stops, absolute frame=
                Plus2"
CDS             440219..441070
                /colour="255 0 0"
                /evidence=predicted
                /translation="TQTLDRTXRAM-----PTAYDSDLTTLQWELLEPLIPAAK----
                ---PGGRPRTTDMLSVLNAIFYLVVTGCQWRQLPHDFPCWSTVYSYFRRWRDDGTWSQ
                INEHLRMQVRVSEDRHPSPSAAICDAQSVKVGNPRCH-LIGFDGGKMVKGRKRHVLVD
                TLGLVLMVMVTAANISDQR-GAKILFWKARRQGaslSRLVRIWADAGYQGQALMKWVM
                DRSQYVLEVIKRSDNLAGFQVVSKRWIVERTFGWLLWSRRLNKDYEVLTRTAEALVYV
                AMIRLMVRRLAQEHXNFSNSLS"
                /note="IS5_IS1031  e-value= 2.3e-109 complete sequence hit
                coverage= 100%, between model( 295 aa) positions 1; 295
                length is 284aa with 14 gaps, 2 stops, absolute frame=
                Plus2"
CDS             440261..441070
                /colour="255 0 0"
                /evidence=predicted
                /translation="YDSDLTTLQWELLEPLIPAAK-------PGGRPRTTDMLSVLNA
                IFYLVVTGCQWRQLPHDFPCWSTVYSYFRRWRDDGTWSQINEHLRMQVRVSEDRHPSP
                SAAICDAQSVKVGNPRCH-LIGFDGGKMVKGRKRHVLVDTLGLVLMVMVTAANISDQR
                -GAKILFWKARRQGaslSRLVRIWADAGYQGQALMKWVMDRSQYVLEVIKRSDNLAGF
                QVVSKRWIVERTFGWLLWSRRLNKDYEVLTRTAEALVYVAMIRLMVRRLAQEHXNFSN
                SLS"
                /note="IS5_IS1031  e-value= 1.5e-111 fragment hit
                coverage= 93.56%, between model( 295 aa) positions 20;
                295 length is 270aa with 9 gaps, 1 stops, absolute frame=
                Plus2"
CDS             440270..440509
                /colour="255 0 0"
                /evidence=predicted
                /translation="DLTTLQWELLEPLIPAAKP-GGRPRT-TDMLSVLNAIFYLVVTG
                CQWRQLPHDFPCWSTVYSYFRRWRDDGTWSQINEHLRM"
                /note="IS5_IS427   e-value= 2e-19 fragment hit coverage=
                27.70%, between model( 296 aa) positions 125; 206 length
                is 80aa with 2 gaps, 0 stops, absolute frame= Plus2"
CDS             720377..721051
                /colour="255 0 0"
                /evidence=predicted
                /translation="SXGICMKCPECGGQH-IHKNGHRR-GKQNHICVTCGRQFL--ST
                YSKRGYSDWTKRLCLRMYVNGMGLKGIERVIGVAHTTVIHWVQQVGVLLPDAYDPDD-
                -----IP---QVGELDELETFVGKKRNKVWIW-TVVDHFHPG--ILGWVVGNHSAETF
                RPLWqaIVCWQCFFWVSDGNPVYpgFIPEgDQIVSKTYMTRVEGENTRLRHYLARLHR
                KTLCYSKSVDMLKHSIRLL---IHYL"
                /note="IS1_ORF1    e-value= 4e-41 complete sequence hit
                coverage= 100%, between model( 239 aa) positions 1; 239
                length is 225aa with 19 gaps, 1 stops, absolute frame=
                Plus2"
CDS             720377..721051
```

```
                     /colour="255 0 0"
                     /evidence=predicted
                     /translation="SXGICMKCPECGGQH-IHKNGHRR-GKQNHICVTCGRQFL--ST
                     YSKRGYSDWTKRLCLRMYVNGMGLKGIERVIGVAHTTVIHWVQQVGVLLPDAYDPDD-
                     -----IP---QVGELDELETFVGKKRNKVWIW-TVVDHFHPG--ILGWVVGNHSAETF
                     RPLWqaIVCWQCFFWVSDGNPVYpgFIPEgDQIVSKTYMTRVEGENTRLRHYLARLHR
                     KTLCYSKSVDMLKHSIRLL---IHYL"
                     /note="IS1_ORF1    e-value= 2.4e-40 complete sequence hit
                     coverage= 100%, between model( 239 aa) positions 1; 239
                     length is 225aa with 19 gaps, 1 stops, absolute frame=
                     Plus2"
     CDS             720377..721057
                     /colour="255 0 0"
                     /evidence=predicted
                     /translation="SXGICMKCPECGGQH-IHKNGHRR-GKQNHICVTCGR----QFL
                     STYSKRGysdwTKRLCLRMYVNGMGLKGIERVIGVAHTTVIHWVQQV--GVLLPDAYD
                     PDDIPQVGELDELETFVGKKRNKVWIWTVVDHFHPGILGWVVGNHSAETFRPLWQAIV
                     CWQ-CFFWVSDGNPVYPGFIPEG----DQIVSKTYMTRVEGENTRLRHYLARLHRKTL
                     CYSKSVDMLKHSIRLLI-HYLKF"
                     /note="IS1_ORF2    e-value= 1.5e-28 complete sequence hit
                     coverage= 100%, between model( 237 aa) positions 1; 237
                     length is 227aa with 14 gaps, 1 stops, absolute frame=
                     Plus2"
     CDS             720656..721057
                     /colour="255 0 0"
                     /evidence=predicted
                     /translation="DPDDIPQVGELDELETFVGKKRNKVWIWTVVDHFHPGILGWVVG
                     NHSAETFRPLWQAIVCWQ-CFFWVSDGNPVYPGFIPEG----DQIVSKTYMTRVEGEN
                     TRLRHYLARLHRKTLCYSKSVDMLKHSIRLLI-HYLKF"
                     /note="IS1_ORF2    e-value= 3e-31 fragment hit coverage=
                     59.07%, between model( 237 aa) positions 98; 237 length
                     is 134aa with 6 gaps, 0 stops, absolute frame= Plus2"
     CDS             748838..749635
                     /colour="255 0 0"
                     /evidence=predicted
                     /translation="IRVSCVVAQGDGSTALQAIHQAFGLEGxlfGCKLHPPLYCKKEN
                     DLVIKRGHSRICIWIXVRTAVVVLT-QKQWXTHSDYTLSAVTSKRLFEK-------XV
                     KLSRYXsVNTQTLDRTXRAMPTAYDSDLTTLQWELLEPLIPAAKP-GGRPRT-TDMLS
                     VLNAIFYLVVTGCQWRQLPHDFPCWSTVYSYFRRWRDDGTWSQINEHLRMQVRVSE--
                     ---------------------------DRHPSPSAAICDAQSVkvgnpRCHLIGFDGG
                     KMVKGRKRHVLVDTLGLVLMVMVTAANIS"
                     /note="IS5_IS427   e-value= 2.8e-07 complete sequence hit
                     coverage= 100%, between model( 296 aa) positions 1; 296
                     length is 266aa with 39 gaps, 5 stops, absolute frame=
                     Plus2"
     CDS             749108..749977
                     /colour="255 0 0"
                     /evidence=predicted
                     /translation="EKXVKLSRYXSVNTQTLDRTXramptaYDSDLTTLQWELLEPLI
                     PAAKPG-----GRPRTTDML--------------------SVLNAIFYLVVTGCQWRQ
                     --LPHDFPCWSTVYSYFRRWRDDGTWSQINEHLRMQVRVSED---RHPSPSAAICDAQ
                     SVK------------------------------VGNpRcHLIGFD--------GGKM-
                     ----------------------------------------------VKGRKRHVLVD-TL
                     GLVLMVMVTAANISDQRGAK----------------------ILFWKAQRQGASLSRL
                     VR----IWADAGYQGQALMKWVMDRFQYVLEVVKRSDNLAGFQVIPKRWIVERTFGWL
                     LWSRRLNKDYEVLTRTAEALVYYAMIRLMVRRLAQEHX"
                     /note="IS5_IS5     e-value= 1.8e-09 complete sequence hit
                     coverage= 100%, between model( 422 aa) positions 1; 422
                     length is 290aa with 140 gaps, 4 stops, absolute frame=
                     Plus2"
```

```
CDS             749147..749998
                /colour="255 0 0"
                /evidence=predicted
                /translation="TQTLDRTXRAM-----PTAYDSDLTTLQWELLEPLIPAAK----
                ---PGGRPRTTDMLSVLNAIFYLVVTGCQWRQLPHDFPCWSTVYSYFRRWRDDGTWSQ
                INEHLRMQVRVSEDRHPSPSAAICDAQSVKVGNPRCH-LIGFDGGKMVKGRKRHVLVD
                TLGLVLMVMVTAANISDQR-GAKILFWKAQRQGaslSRLVRIWADAGYQGQALMKWVM
                DRFQYVLEVVKRSDNLAGFQVIPKRWIVERTFGWLLWSRRLNKDYEVLTRTAEALVYV
                AMIRLMVRRLAQEHXNFSNSLX"
                /note="IS5_IS1031  e-value= 1.8e-111 complete sequence hit
                coverage= 100%, between model( 295 aa) positions 1; 295
                length is 284aa with 14 gaps, 3 stops, absolute frame=
                Plus2"
CDS             749189..749998
                /colour="255 0 0"
                /evidence=predicted
                /translation="YDSDLTTLQWELLEPLIPAAK-------PGGRPRTTDMLSVLNA
                IFYLVVTGCQWRQLPHDFPCWSTVYSYFRRWRDDGTWSQINEHLRMQVRVSEDRHPSP
                SAAICDAQSVKVGNPRCH-LIGFDGGKMVKGRKRHVLVDTLGLVLMVMVTAANISDQR
                -GAKILFWKAQRQGaslSRLVRIWADAGYQGQALMKWVMDRFQYVLEVVKRSDNLAGF
                QVIPKRWIVERTFGWLLWSRRLNKDYEVLTRTAEALVYVAMIRLMVRRLAQEHXNFSN
                SLX"
                /note="IS5_IS1031  e-value= 1.2e-113 fragment hit
                coverage= 93.56%, between model( 295 aa) positions 20;
                295 length is 270aa with 9 gaps, 2 stops, absolute frame=
                Plus2"
CDS             749198..749437
                /colour="255 0 0"
                /evidence=predicted
                /translation="DLTTLQWELLEPLIPAAKP-GGRPRT-TDMLSVLNAIFYLVVTG
                CQWRQLPHDFPCWSTVYSYFRRWRDDGTWSQINEHLRM"
                /note="IS5_IS427   e-value= 2e-19 fragment hit coverage=
                27.70%, between model( 296 aa) positions 125; 206 length
                is 80aa with 2 gaps, 0 stops, absolute frame= Plus2"
CDS             791804..792046
                /colour="255 0 0"
                /evidence=predicted
                /translation="VKLAELIPWESFESEYADQFSQ---TMGAPAKPFRVALGTLIIK
                EKLGISDAETVEQIRENPYLQYFLGFSEYRESAPFDASML"
                /note="IS5_IS5     e-value= 2.6e-06 fragment hit coverage=
                19.91%, between model( 422 aa) positions 23; 106 length
                is 81aa with 3 gaps, 0 stops, absolute frame= Plus2"
CDS             940328..941023
                /colour="255 0 0"
                /evidence=predicted
                /translation="FKHLHLGMIsDIKRKSLPAIARAVGLENHQSLHHFLSESPWVAS
                hLSDkRLDIILKILDGRRLILLID------------------ETGDCKKGKSTDYVKR
                QYIGNVGKKENGIvAVTAYGLFQGMILPLSFEVYKPR---------ErLKEEDEYLSK
                PQIAAGMIRQLQGMGFCFELVLADSLYGEAQTNFVNVLEELKLPYILAIRSNHAVWLP
                AEQAVFAEPWQSFERTFSNGTTETRYrQEIIYGqrhhQKRY"
                /note="IS4   e-value= 4.7e-14 fragment hit coverage=
                51.23%, between model( 486 aa) positions 1; 249 length is
                232aa with 27 gaps, 0 stops, absolute frame= Plus2"
CDS             940328..941455
                /colour="255 0 0"
                /evidence=predicted
                /translation="FKHLHLGMIsDIKRKSLPAIARAVGLENHQSLHHFLSESPWVAS
                hLSDkRLDIILKILDGRRLILLID------------------ETGDCKKGKSTDYVKR
                QYIGNVGKKENGIvAVTAYGLFQGMILPLSFEVYKPR---------ErLKEEDEYLSK
                PQIAAGMIRQLQGMGFCFELVLADSLYGEAQTNFVNVLEELKLPYILAIRSNHAVWLP
```

```
                     AEQAVFAEPWQSFERTFSNGT-------------------------------TETRYR
                     QEIIYGQRHHQKRyWLLTTDPQTLPENSTS----------------------YVMAA
                     APE----IKLDEIGDCYGFRTWIEYGLKQSKD-TLGWADFRMTHYEQIEKWWEIVMSA
                     FLMVSLFADVFNDS------------------------CPVAHQHFAQHPwWDNQNG
                     WKNLLNNVR--LIIQPLIS----WNLLKRWLEVFPSRALKKGF"
                     /note="IS4   e-value= 9.9e-07 complete sequence hit
                     coverage= 100%, between model( 486 aa) positions 1; 486
                     length is 376aa with 117 gaps, 0 stops, absolute frame=
                     Plus2"
CDS                  942041..942427
                     /colour="255 0 0"
                     /evidence=predicted
                     /translation="YADESGMDHRDEYDY-AYGPKGE-----------RVYA-LKTG-
                     RRSGRVNMIAALRAKQL--MAPFTIEGACNRTVFELwLERCLIPvlKPGQKLVI--DN
                     ATFHKGGQIQELVEKAG--CEVWYLPPYSPDLNKIERSWSWIKSRIR"
                     /note="IS630 e-value= 1.1e-13 fragment hit coverage=
                     38.83%, between model( 376 aa) positions 188; 333 length
                     is 129aa with 20 gaps, 0 stops, absolute frame= Plus2"
CDS                  1009808..1010014
                     /colour="255 0 0"
                     /evidence=predicted
                     /translation="IKANILKGQLIQADETPIKMVK---RE--TGYVWVFTTTD----
                     ---------AVLYFFKSTRKAFFLKELLKDFTGVLISDFYAGY"
                     /note="IS66_ORF2   e-value= 1e-08 fragment hit coverage=
                     16.26%, between model( 535 aa) positions 243; 329 length
                     is 69aa with 18 gaps, 0 stops, absolute frame= Plus2"
CDS                  1009844..1010014
                     /colour="255 0 0"
                     /evidence=predicted
                     /translation="ADETPIKMV-----KRETGYVWVFTTTD---------AVLYFFK
                     STRKAFFLKELLKDFTGVLISDFYAGY"
                     /note="IS66_ORF3   e-value= 3.6e-07 fragment hit coverage=
                     12.72%, between model( 558 aa) positions 287; 357 length
                     is 57aa with 14 gaps, 0 stops, absolute frame= Plus2"
CDS                  1313054..1314091
                     /colour="255 0 0"
                     /evidence=predicted
                     /translation="PMIKIDFTAEEIQQLNYERYHHPHPRVQRRMEVLYLKSQGLSHT
                     QICQLCQISRPTLAKTLRQYQQGG---IEGLKTLEYKGQPSLLNAHS-DSVRAYFEQH
                     PP---------RTSAEAQAVIEQLTGIKRSPTQIKAFLKRIGCRYRKVGYVPGKSSLP
                     -EKIEEQEQFRHTRLEPLleeAQRQERLVFFVDAAHFVHRAYLGF-VWCITRI-----
                     ---------FIPSPS-GRQRFNVLGALDAITKDLVSVTNHTYINSHSMCLLLAKLALL
                     DPV-IPISVILDNARYQKCQLVTDFAEIVD--IELVYLPSYSPHLNLIERLWRFVRKE
                     CLYSKYYADFPAFKGAIQQCIDQCNGEHKAKLTTLLSLKFQSFKK"
                     /note="IS630 e-value= 6.7e-25 complete sequence hit
                     coverage= 100%, between model( 376 aa) positions 1; 376
                     length is 346aa with 33 gaps, 0 stops, absolute frame=
                     Plus2"
CDS                  1313054..1314091
                     /colour="255 0 0"
                     /evidence=predicted
                     /translation="PMIKIDFTAEEIQQLNYERYHHPHPRVQRRMEVLYLKSQGLSHT
                     QICQLCQISRPTLAKTLRQYQQGG---IEGLKTLEYKGQPSLLNAHS-DSVRAYFEQH
                     PP---------RTSAEAQAVIEQLTGIKRSPTQIKAFLKRIGCRYRKVGYVPGKSSLP
                     -EKIEEQEQFRHTRLEPLleeAQRQERLVFFVDAAHFVHRAYLGF-VWCITRI-----
                     ---------FIPSPS-GRQRFNVLGALDAITKDLVSVTNHTYINSHSMCLLLAKLALL
                     DPV-IPISVILDNARYQKCQLVTDFAEIVD--IELVYLPSYSPHLNLIERLWRFVRKE
                     CLYSKYYADFPAFKGAIQQCIDQCNGEHKAKLTTLLSLKFQSFKK"
                     /note="IS630 e-value= 4.9e-24 complete sequence hit
                     coverage= 100%, between model( 376 aa) positions 1; 376
```

```
                     length is 346aa with 33 gaps, 0 stops, absolute frame=
                     Plus2"
     CDS             1313114..1313272
                     /colour="255 0 0"
                     /evidence=predicted
                     /translation="HHPHPRVQRRMEVLYLKSQGLSHTQICQLCQISRPTLAKTLRQY
                     QQGGIEGLK"
                     /note="IS481 e-value= 3.7e-09 fragment hit coverage=
                     15.10%, between model( 351 aa) positions 1; 53 length is
                     53aa with 0 gaps, 0 stops, absolute frame= Plus2"
     CDS             1525481..1525684
                     /colour="255 0 0"
                     /evidence=predicted
                     /translation="QRYQCKDCNRRFN-ERTGTPMARLRtasSVVSYAIKARTEGMGV
                     RAAGRTFGKSHTTIMRWEKRLADQA"
                     /note="IS1_ORF1    e-value= 2.6e-10 fragment hit coverage=
                     27.62%, between model( 239 aa) positions 28; 93 length is
                     68aa with 1 gaps, 0 stops, absolute frame= Plus2"
     CDS             1529090..1529305
                     /colour="255 0 0"
                     /evidence=predicted
                     /translation="REIHEAIGVSIRTIERVRMRFVEEG---LEAAINQRsGAGRKRK
                     IQGEQEAHLIALRCSEPPVG----HARWTLRLLAD"
                     /note="IS630 e-value= 9.9e-08 fragment hit coverage=
                     20.74%, between model( 376 aa) positions 44; 121 length
                     is 72aa with 7 gaps, 0 stops, absolute frame= Plus2"
     CDS             1580969..1581355
                     /colour="255 0 0"
                     /evidence=predicted
                     /translation="YADESGMDHRDEYDY-AYGPKGE-----------RVYA-LKTG-
                     RRSGRVNMIAALRAKQL--MAPFTIEGACNRTVFEIWLercLIPMLKPGQKLVI--DN
                     ATFHKGGRIQELVEKAG--CEVWYLPPYSPDLNKIERSWSWIKSRIR"
                     /note="IS630 e-value= 6.9e-14 fragment hit coverage=
                     38.83%, between model( 376 aa) positions 188; 333 length
                     is 129aa with 20 gaps, 0 stops, absolute frame= Plus2"
     CDS             1587323..1587460
                     /colour="255 0 0"
                     /evidence=predicted
                     /translation="WQTDGWEGYARQLPDE---VVHEVSKALTQRLERTNGIVRQQTG
                     RWHRR"
                     /note="IS1_ORF2    e-value= 1.8e-11 fragment hit coverage=
                     20.68%, between model( 237 aa) positions 164; 212 length
                     is 46aa with 3 gaps, 0 stops, absolute frame= Plus2"
     CDS             1631051..1631893
                     /colour="255 0 0"
                     /evidence=predicted
                     /translation="LDRTLRAMPTA--------YDSDLTTLQWELLEPLIPAAK----
                     ---PGGRPRTTDMRSVLNAILYLVVTGCQWRQLPHDFPCWSTVYSYFRRWRDDGTWVH
                     INEHLRMQERVSEDRHPSPRAAICDAQSVKVGNPRCH-SIGFDGGKQIKGRKRHVLVD
                     TLGLVLMVIVTAANISDQR-GAKILFWKARRQGaslSRLVRIWADAGYQGQAFMKWVM
                     DRFQYVLEVIKRSDNLAGFQVIPKRWIVERTFGWLLWSRRLNKDYEVLTRTAEALVYV
                     AMIRLMVRRLTEEAXNFSNSLL"
                     /note="IS5_IS1031  e-value= 2.8e-110 complete sequence hit
                     coverage= 100%, between model( 295 aa) positions 1; 295
                     length is 281aa with 17 gaps, 1 stops, absolute frame=
                     Plus2"
     CDS             1631084..1631893
                     /colour="255 0 0"
                     /evidence=predicted
                     /translation="YDSDLTTLQWELLEPLIPAAK-------PGGRPRTTDMRSVLNA
```

```
                     ILYLVVTGCQWRQLPHDFPCWSTVYSYFRRWRDDGTWVHINEHLRMQERVSEDRHPSP
                     RAAICDAQSVKVGNPRCH-SIGFDGGKQIKGRKRHVLVDTLGLVLMVIVTAANISDQR
                     -GAKILFWKARRQGaslSRLVRIWADAGYQGQAFMKWVMDRFQYVLEVIKRSDNLAGF
                     QVIPKRWIVERTFGWLLWSRRLNKDYEVLTRTAEALVYYVAMIRLMVRRLTEEAXNFSN
                     SLL"
                     /note="IS5_IS1031  e-value= 1.4e-112 fragment hit
                     coverage= 93.56%, between model( 295 aa) positions 20;
                     295 length is 270aa with 9 gaps, 1 stops, absolute frame=
                     Plus2"
CDS                  1631093..1631332
                     /colour="255 0 0"
                     /evidence=predicted
                     /translation="DLTTLQWELLEPLIPAAKP-GGRPRT-TDMRSVLNAILYLVVTG
                     CQWRQLPHDFPCWSTVYSYFRRWRDDGTWVHINEHLRM"
                     /note="IS5_IS427   e-value= 1.2e-18 fragment hit coverage=
                     27.70%, between model( 296 aa) positions 125; 206 length
                     is 80aa with 2 gaps, 0 stops, absolute frame= Plus2"
CDS                  2086388..2086531
                     /colour="255 0 0"
                     /evidence=predicted
                     /translation="VIVIDNASFHHGQRIEEIVAEAG--CEIWYLPSYSPDLNKIERW
                     WFVLKN"
                     /note="IS630 e-value= 1.7e-14 fragment hit coverage=
                     13.30%, between model( 376 aa) positions 281; 330 length
                     is 48aa with 2 gaps, 0 stops, absolute frame= Plus2"
CDS                  2216453..2216695
                     /colour="255 0 0"
                     /evidence=predicted
                     /translation="VKLAELIPWESFESEYADQFSQ---TMGAPAKPFRVALGTLIIK
                     EKLGISDAETVEQIRENPYLQYFLGFSEYRESAPFDASML"
                     /note="IS5_IS5     e-value= 2.6e-06 fragment hit coverage=
                     19.91%, between model( 422 aa) positions 23; 106 length
                     is 81aa with 3 gaps, 0 stops, absolute frame= Plus2"
CDS                  2274167..2274631
                     /colour="255 0 0"
                     /evidence=predicted
                     /translation="LSIYYPKHASPKLR---TFSA--RLXLLHISnagXRSSIKKSIV
                     ENHLGNYAVtrlTILKGMVSKDHDHTLIEYPPSKSISDIAKRLKGFTSRRLQQEYREL
                     ERRYWDKYLWALGYGAWSTGNISEQMVEEYLEHHRLVSNNDINTFMLEXIKTLVLRRI
                     "
                     /note="IS200 e-value= 1.5e-09 complete sequence hit
                     coverage= 100%, between model( 154 aa) positions 1; 154
                     length is 155aa with 5 gaps, 3 stops, absolute frame=
                     Plus2"
CDS                  2274167..2274628
                     /colour="255 0 0"
                     /evidence=predicted
                     /translation="LSIYYPKHASPKLR---TFSARLXLLHISNAGXRSSIKKSIVEn
                     hLGNYAvtRLTILKGMVSKDHDHTLIEYPPSKSISDIAKRLKGFTSRRLQQEYRELER
                     RYWDKYLWALGYGAWSTGNISEQMVEEYLEHHRLVSNNDINTFMLEXIKTLVLRR"
                     /note="IS200_IS605 e-value= 3.6e-09 complete sequence hit
                     coverage= 100%, between model( 153 aa) positions 1; 153
                     length is 154aa with 3 gaps, 3 stops, absolute frame=
                     Plus2"
CDS                  2274320..2274559
                     /colour="255 0 0"
                     /evidence=predicted
                     /translation="TILKGMVSKDHDHTLIEYPPSKSISDIAKRLKGFTSRRLQQEYR
                     ELERRYWDKYLWALGYGAWSTGNISEQMVEEYLEHH"
                     /note="IS200 e-value= 3.2e-19 fragment hit coverage=
```

51.95%, between model(154 aa) positions 51; 130 length
is 80aa with 0 gaps, 0 stops, absolute frame= Plus2"

```
CDS             2274320..2274559
                /colour="255 0 0"
                /evidence=predicted
                /translation="TILKGMVSKDHDHTLIEYPPSKSISDIAKRLKGFTSRRLQQEYR
                ELERRYWDKYLWALGYGAWSTGNISEQMVEEYLEHH"
                /note="IS200_IS605 e-value= 7.4e-15 fragment hit
                coverage= 52.29%, between model( 153 aa) positions 51;
                130 length is 80aa with 0 gaps, 0 stops, absolute frame=
                Plus2"
CDS             2409029..2410090
                /colour="255 0 0"
                /evidence=predicted
                /translation="IVKRXKN------EQCIVSHpYPALXPPQPLAQG---ISSA---
                --RQXHFXESEQRRQRXSENCSVRASSRPIDAAIRDRKKSLNVLTP--NQKRQMVRQL
                QQDYSIRQICQVLNYPRSQVYYHARGQP-----DESELKAAIAGVAGAYP-TYGYRRI
                TAQLQ---RQGYCVNHKRVARLMRQIGIMAKTKVK-RKRTTNSEHSfpRYGNRVLNLS
                --IDHPEQVWVADITYIRLQ-QEFVYLAVVMDVFTRAIRGWHLSRHIDQQLTLRALNK
                ALERAT---PEIHHS-----DQGVQYAAAAYMQLLQQHQVQISMAEVGQAWQNGYAER
                LMRTIKEEEVD--LSDYRNFTEAYEHIEQFLEdVYMHKRIHSSLGYLTPCEYEQQWRQ
                Q"
                /note="IS3_IS150_ORF2    e-value= 1.7e-22 complete sequence
                hit coverage= 100%, between model( 389 aa) positions 1;
                389 length is 354aa with 39 gaps, 5 stops, absolute
                frame= Plus2"
CDS             2409203..2410087
                /colour="255 0 0"
                /evidence=predicted
                /translation="PIDAAIRDRKKSLnVLTPNQKRQMVRQLQQDYSIRQICQVLNYP
                RSQVYYHARGQPDESELKAAIAGV-AGAYPTYGYRRITAQLQRQGYCVNHKRVARLMR
                QIGIMAKTKVK----RKRTTNSEHSF-PRYGNRvlNLSIDHPEQVWVADITYIRL--Q
                QEFVYLAVVMDVFTRAIRGWHLSRHIDQQLTLRALNKALER----ATPEIHHSDQGVQ
                YAAAAYMQLLQQHQVQISMAEVGQAWQNGYAERLMRTIKEEEVDLSDYRNFT--EAYE
                HIEQFLEDVYMHKRIHSSLGYLTPCEYEQQWRQ"
                /note="IS3_IS150_ORF1    e-value= 4.3e-49 complete sequence
                hit coverage= 100%, between model( 306 aa) positions 1;
                306 length is 295aa with 14 gaps, 0 stops, absolute
                frame= Plus2"
CDS             2409239..2410123
                /colour="255 0 0"
                /evidence=predicted
                /translation="LNVLTPNQK-RQMVRQLQqdYSIRQICQVLNYP---RSQVYYHA
                RgQPDESELKA--AIAGV-------AGA---YPTYGYRRITAQLQRQGYCVNHKRVAR
                LMRQIGIMAKTKVKRKRTTNSEHSFPRYGNRVL-NLSIDHPEQVWVADITYIRLQQEF
                VYLAVVMDVFTRAIRGWHLSRHIDQQLTLRALNKALERATPE--------IHHSDQGV
                QYAAAAYMQLLQQHQVQISMAEVGQAWQNGYAERLMRTIKEEEVDLSDYrnFTEAYEH
                IEQFLEDV--YMHKRIHSSLGYLTPCEYEQQWRQQNNHYCMNKEDS"
                /note="IS3_IS51_ORF2     e-value= 1.6e-26 complete sequence
                hit coverage= 100%, between model( 317 aa) positions 1;
                317 length is 295aa with 27 gaps, 0 stops, absolute
                frame= Plus2"
CDS             2409245..2409499
                /colour="255 0 0"
                /evidence=predicted
                /translation="VLTPNQKRQMVRQLQQDY--SIRQICQVLNYPRSQVYYHARGQP
                DESELKAAIAGVAGAYPTYGYRRITAQLQRQGYCVNHKRVARL"
                /note="IS3_IS407_ORF2    e-value= 1.2e-17 fragment hit
                coverage= 25.29%, between model( 344 aa) positions 60;
                146 length is 85aa with 2 gaps, 0 stops, absolute frame=
```

```
                     Plus2"
CDS                  2409263..2410090
                     /colour="255 0 0"
                     /evidence=predicted
                     /translation="KRQMVRQLQQDYSIRQICQVLNYPRSQVYYHARGQP-----DES
                     ELKAAIAGVAGAYP-TYGYRRITAQLQ---RQGYCVNHKRVARLMRQIGIMAKTKVK-
                     RKRTTNSEHSfpRYGNRVLNLS--IDHPEQVWVADITYIRLQ-QEFVYLAVVMDVFTR
                     AIRGWHLSRHIDQQLTLRALNKALERAT---PEIHHS-----DQGVQYAAAAYMQLLQ
                     QHQVQISMAEVGQAWQNGYAERLMRTIKEEEVD--LSDYRNFTEAYEHIEQFLEdVYM
                     HKRIHSSLGYLTPCEYEQQWRQQ"
                     /note="IS3_IS150_ORF2    e-value= 2.2e-47 fragment hit
                     coverage= 76.09%, between model( 389 aa) positions 94;
                     389 length is 276aa with 23 gaps, 0 stops, absolute
                     frame= Plus2"
CDS                  2409266..2410114
                     /colour="255 0 0"
                     /evidence=predicted
                     /translation="RQMVRQLQQDYSIRQICQVLNYPRSQVYYHA-----------RG
                     QPDEsELKAAIAGV-AGAYPTYGYRRITAQLQRQGYCVNHKRVARLMRQIGIMAKTKV
                     KRKRTTNSEHSFPRYGNRVL-NLSIDHPEQVWVADITYIRLQQEFVYLAVVMDVFTRA
                     IRGWHLSRHIDQQLT-LRALNKALERATPE-------IHHSDQGVQYAAAAYMQLLQQ
                     HQVQISMAEVGQAWQNGYAERLMRTIKEEEVDL---S-DYRNFTEAYEHIEQFLEDVY
                     MHKRIHSSLGYLTPCEYEQQWRQQNNHYCMNK"
                     /note="IS3_IS51_ORF1     e-value= 8.1e-53 complete sequence
                     hit coverage= 100%, between model( 307 aa) positions 1;
                     307 length is 283aa with 25 gaps, 0 stops, absolute
                     frame= Plus2"
CDS                  2409266..2410114
                     /colour="255 0 0"
                     /evidence=predicted
                     /translation="RQMVRQLQQDYSIRQICQVLNYPRSQVYYHA-----------RG
                     QPDEsELKAAIAGV-AGAYPTYGYRRITAQLQRQGYCVNHKRVARLMRQIGIMAKTKV
                     KRKRTTNSEHSFPRYGNRVL-NLSIDHPEQVWVADITYIRLQQEFVYLAVVMDVFTRA
                     IRGWHLSRHIDQQLT-LRALNKALERATPE-------IHHSDQGVQYAAAAYMQLLQQ
                     HQVQISMAEVGQAWQNGYAERLMRTIKEEEVDL---S-DYRNFTEAYEHIEQFLEDVY
                     MHKRIHSSLGYLTPCEYEQQWRQQNNHYCMNK"
                     /note="IS3_IS51_ORF1     e-value= 6.3e-51 complete sequence
                     hit coverage= 100%, between model( 307 aa) positions 1;
                     307 length is 283aa with 25 gaps, 0 stops, absolute
                     frame= Plus2"
CDS                  2409269..2410084
                     /colour="255 0 0"
                     /evidence=predicted
                     /translation="QMVRQLQQDYSIRQICQVLNYPRSQVYYHARG--QPD-----ES
                     ELKAAI-AGVAGAYPTYGYRRITAQLQ-RQGYCVNHKRVARLMRQIGIMAKTKVKRKR
                     TTNSEHSfPRYGNRVLNLS-IDHPEQVWVADITYIRLQQEFVYLAVVMDVFTRAIRGW
                     HLSRHIDQQLTLRALNKALERA-TPE--IHHSDQGVQYAAAAYMQLLQQHQVQISMAE
                     VGQAWQNGYAERLMRTIK-EEEVDL-SDYRNFTEAYEHIEQFLEDVYMHKRIHSSLGY
                     LTPCEYEQQWR"
                     /note="IS3_IS3_ORF1      e-value= 9.5e-54 complete sequence
                     hit coverage= 100%, between model( 286 aa) positions 1;
                     286 length is 272aa with 15 gaps, 0 stops, absolute
                     frame= Plus2"
CDS                  2409269..2410084
                     /colour="255 0 0"
                     /evidence=predicted
                     /translation="QMVRQLQQDYSIRQICQVLNYPRSQVYYHARG--QPD-----ES
                     ELKAAI-AGVAGAYPTYGYRRITAQLQ-RQGYCVNHKRVARLMRQIGIMAKTKVKRKR
                     TTNSEHSfPRYGNRVLNLS-IDHPEQVWVADITYIRLQQEFVYLAVVMDVFTRAIRGW
                     HLSRHIDQQLTLRALNKALERA-TPE--IHHSDQGVQYAAAAYMQLLQQHQVQISMAE
```

```
                     VGQAWQNGYAERLMRTIK-EEEVDL-SDYRNFTEAYEHIEQFLEDVYMHKRIHSSLGY
                     LTPCEYEQQWR"
                     /note="IS3_IS3_ORF1      e-value= 2.5e-54 complete sequence
                     hit coverage= 100%, between model( 286 aa) positions 1;
                     286 length is 272aa with 15 gaps, 0 stops, absolute
                     frame= Plus2"
CDS                  2409272..2410087
                     /colour="255 0 0"
                     /evidence=predicted
                     /translation="MVRQLQQDYSIRQICQVLNYPRSQVYYHARGQPDESELKAAIAG
                     V-AGAYPTYGYRRITAQLQRQGYCVNHKRVARLMRQIGIMAKTKVK----RKRTTNSE
                     HSF-PRYGNRvlNLSIDHPEQVWVADITYIRL--QQEFVYLAVVMDVFTRAIRGWHLS
                     RHIDQQLTLRALNKALER----ATPEIHHSDQGVQYAAAAYMQLLQQHQVQISMAEVG
                     QAWQNGYAERLMRTIKEEEVDLSDYRNFT--EAYEHIEQFLEDVYMHKRIHSSLGYLT
                     PCEYEQQWRQ"
                     /note="IS3_IS150_ORF1    e-value= 6.3e-50 fragment hit
                     coverage= 92.81%, between model( 306 aa) positions 23;
                     306 length is 272aa with 14 gaps, 0 stops, absolute
                     frame= Plus2"
CDS                  2409281..2410096
                     /colour="255 0 0"
                     /evidence=predicted
                     /translation="QLQQDYSIRQICQVLNYPRSQVYYHARGQPDE---------SEL
                     KAAIAGVAGaypTYGYRRITAQLQRQGYCVNHKRVARLMRQIGIMAKTKVKRKRTTNS
                     EHSFPRY---GNRVLNLSIDHPEQVWVADITYIRLQQEFVYLAVVMDVFTRAIRGWHL
                     SRHIDQQLTLRALNKALER----ATPE---IHHSDQGVQYAAAAYMQLLQQHQVQISM
                     AEVGQAWQNGYAERLMRTIKEEEVDLSDYRNFTEAYEHIEQFLEDVYMHKRIHSSLGY
                     LTPCEYEQQWRQQNN"
                     /note="IS3_IS3_ORF2      e-value= 1.2e-64 complete sequence
                     hit coverage= 100%, between model( 288 aa) positions 1;
                     288 length is 272aa with 19 gaps, 0 stops, absolute
                     frame= Plus2"
CDS                  2409281..2410096
                     /colour="255 0 0"
                     /evidence=predicted
                     /translation="QLQQDYSIRQICQVLNYPRSQVYYHARGQPDE---------SEL
                     KAAIAGVAGaypTYGYRRITAQLQRQGYCVNHKRVARLMRQIGIMAKTKVKRKRTTNS
                     EHSFPRY---GNRVLNLSIDHPEQVWVADITYIRLQQEFVYLAVVMDVFTRAIRGWHL
                     SRHIDQQLTLRALNKALER----ATPE---IHHSDQGVQYAAAAYMQLLQQHQVQISM
                     AEVGQAWQNGYAERLMRTIKEEEVDLSDYRNFTEAYEHIEQFLEDVYMHKRIHSSLGY
                     LTPCEYEQQWRQQNN"
                     /note="IS3_IS3_ORF2      e-value= 3.3e-65 complete sequence
                     hit coverage= 100%, between model( 288 aa) positions 1;
                     288 length is 272aa with 19 gaps, 0 stops, absolute
                     frame= Plus2"
CDS                  2409350..2409508
                     /colour="255 0 0"
                     /evidence=predicted
                     /translation="YHARgQPDESELKAAIAGVAGAYPTYGYRRITAQLQRQGY----
                     -CVNHKRVARLMRQ"
                     /note="IS3_IS2_ORF2      e-value= 9.1e-09 fragment hit
                     coverage= 18.87%, between model( 302 aa) positions 48;
                     104 length is 53aa with 5 gaps, 0 stops, absolute frame=
                     Plus2"
CDS                  2409416..2410099
                     /colour="255 0 0"
                     /evidence=predicted
                     /translation="YPTYGYRRITAQLQRQGYCVNHKRVARLMRQIGIMAKTKVKRKR
                     TTNSEHSFPRYGNRVL-NLSIDHPEQVWVADITYIRLQQEFVYLAVVMDVFTRAIRGW
                     HLSRHIDQQLTLRALNKALERATPE--------IHHSDQGVQYAAAAYMQLLQQHQVQ
```

```
                ISMAEVGQAWQNGYAERLMRTIKEEEVDLSDYrnFTEAYEHIEQFLEDV--YMHKRIH
                SSLGYLTPCEYEQQWRQQNNH"
                /note="IS3_IS51_ORF2    e-value= 1.9e-38 fragment hit
                coverage= 74.76%, between model( 317 aa) positions 73;
                309 length is 228aa with 11 gaps, 0 stops, absolute
                frame= Plus2"
CDS             2500838..2501899
                /colour="255 0 0"
                /evidence=predicted
                /translation="IVKRXKN------EQCIVSHpYPALXPPQPLAQG---ISSA---
                --RQXHFXESEQRRQRXSENCSVRASSRPIDAAIRDRKKSLNVLTP--NQKRQMVRQL
                QQDYSIRQICQVLNYPRSQVYYHARGQP-----DESELKAAIAGVAGAYP-TYGYRRI
                TAQLQ---RQGYCVNHKRVARLMRQIGIMAKTKVK-RKRTTNSEHSfpRYGNRVLNLS
                --IDHPEQVWVADITYIRLQ-QEFVYLAVVMDVFTRAIRGWHLSRHIDQQLTLRALNK
                ALERAT---PEIHHS-----DQGVQYAAAAYMQLLQQHQVQISMAEVGQAWQNGYAER
                LMRTIKEEEVD--LSDYRNFTEAYEHIEQFLEdVYMHKRIHSSLGYLTPCEYEQQWRQ
                Q"
                /note="IS3_IS150_ORF2   e-value= 1.7e-22 complete sequence
                hit coverage= 100%, between model( 389 aa) positions 1;
                389 length is 354aa with 39 gaps, 5 stops, absolute
                frame= Plus2"
CDS             2501012..2501896
                /colour="255 0 0"
                /evidence=predicted
                /translation="PIDAAIRDRKKSLnVLTPNQKRQMVRQLQQDYSIRQICQVLNYP
                RSQVYYHARGQPDESELKAAIAGV-AGAYPTYGYRRITAQLQRQGYCVNHKRVARLMR
                QIGIMAKTKVK----RKRTTNSEHSF-PRYGNRvlNLSIDHPEQVWVADITYIRL--Q
                QEFVYLAVVMDVFTRAIRGWHLSRHIDQQLTLRALNKALER----ATPEIHHSDQGVQ
                YAAAAYMQLLQQHQVQISMAEVGQAWQNGYAERLMRTIKEEEVDLSDYRNFT--EAYE
                HIEQFLEDVYMHKRIHSSLGYLTPCEYEQQWRQ"
                /note="IS3_IS150_ORF1   e-value= 4.3e-49 complete sequence
                hit coverage= 100%, between model( 306 aa) positions 1;
                306 length is 295aa with 14 gaps, 0 stops, absolute
                frame= Plus2"
CDS             2501048..2501932
                /colour="255 0 0"
                /evidence=predicted
                /translation="LNVLTPNQK-RQMVRQLQqdYSIRQICQVLNYP---RSQVYYHA
                RgQPDESELKA--AIAGV-------AGA---YPTYGYRRITAQLQRQGYCVNHKRVAR
                LMRQIGIMAKTKVKRKRTTNSEHSFPRYGNRVL-NLSIDHPEQVWVADITYIRLQQEF
                VYLAVVMDVFTRAIRGWHLSRHIDQQLTLRALNKALERATPE--------IHHSDQGV
                QYAAAAYMQLLQQHQVQISMAEVGQAWQNGYAERLMRTIKEEEVDLSDYrnFTEAYEH
                IEQFLEDV--YMHKRIHSSLGYLTPCEYEQQWRQQNNHYCMNKEDS"
                /note="IS3_IS51_ORF2    e-value= 1.6e-26 complete sequence
                hit coverage= 100%, between model( 317 aa) positions 1;
                317 length is 295aa with 27 gaps, 0 stops, absolute
                frame= Plus2"
CDS             2501054..2501308
                /colour="255 0 0"
                /evidence=predicted
                /translation="VLTPNQKRQMVRQLQQDY--SIRQICQVLNYPRSQVYYHARGQP
                DESELKAAIAGVAGAYPTYGYRRITAQLQRQGYCVNHKRVARL"
                /note="IS3_IS407_ORF2   e-value= 1.2e-17 fragment hit
                coverage= 25.29%, between model( 344 aa) positions 60;
                146 length is 85aa with 2 gaps, 0 stops, absolute frame=
                Plus2"
CDS             2501072..2501899
                /colour="255 0 0"
                /evidence=predicted
                /translation="KRQMVRQLQQDYSIRQICQVLNYPRSQVYYHARGQP-----DES
```

```
                     ELKAAIAGVAGAYP-TYGYRRITAQLQ---RQGYCVNHKRVARLMRQIGIMAKTKVK-
                     RKRTTNSEHSfpRYGNRVLNLS--IDHPEQVWVADITYIRLQ-QEFVYLAVVMDVFTR
                     AIRGWHLSRHIDQQLTLRALNKALERAT---PEIHHS-----DQGVQYAAAAYMQLLQ
                     QHQVQISMAEVGQAWQNGYAERLMRTIKEEEVD--LSDYRNFTEAYEHIEQFLEdVYM
                     HKRIHSSLGYLTPCEYEQQWRQQ"
                     /note="IS3_IS150_ORF2    e-value= 2.2e-47 fragment hit
                     coverage= 76.09%, between model( 389 aa) positions 94;
                     389 length is 276aa with 23 gaps, 0 stops, absolute
                     frame= Plus2"
     CDS             2501075..2501923
                     /colour="255 0 0"
                     /evidence=predicted
                     /translation="RQMVRQLQQDYSIRQICQVLNYPRSQVYYHA-----------RG
                     QPDEsELKAAIAGV-AGAYPTYGYRRITAQLQRQGYCVNHKRVARLMRQIGIMAKTKV
                     KRKRTTNSEHSFPRYGNRVL-NLSIDHPEQVWVADITYIRLQQEFVYLAVVMDVFTRA
                     IRGWHLSRHIDQQLT-LRALNKALERATPE-------IHHSDQGVQYAAAAYMQLLQQ
                     HQVQISMAEVGQAWQNGYAERLMRTIKEEEVDL---S-DYRNFTEAYEHIEQFLEDVY
                     MHKRIHSSLGYLTPCEYEQQWRQQNNHYCMNK"
                     /note="IS3_IS51_ORF1     e-value= 8.1e-53 complete sequence
                     hit coverage= 100%, between model( 307 aa) positions 1;
                     307 length is 283aa with 25 gaps, 0 stops, absolute
                     frame= Plus2"
     CDS             2501075..2501923
                     /colour="255 0 0"
                     /evidence=predicted
                     /translation="RQMVRQLQQDYSIRQICQVLNYPRSQVYYHA-----------RG
                     QPDEsELKAAIAGV-AGAYPTYGYRRITAQLQRQGYCVNHKRVARLMRQIGIMAKTKV
                     KRKRTTNSEHSFPRYGNRVL-NLSIDHPEQVWVADITYIRLQQEFVYLAVVMDVFTRA
                     IRGWHLSRHIDQQLT-LRALNKALERATPE-------IHHSDQGVQYAAAAYMQLLQQ
                     HQVQISMAEVGQAWQNGYAERLMRTIKEEEVDL---S-DYRNFTEAYEHIEQFLEDVY
                     MHKRIHSSLGYLTPCEYEQQWRQQNNHYCMNK"
                     /note="IS3_IS51_ORF1     e-value= 6.3e-51 complete sequence
                     hit coverage= 100%, between model( 307 aa) positions 1;
                     307 length is 283aa with 25 gaps, 0 stops, absolute
                     frame= Plus2"
     CDS             2501078..2501893
                     /colour="255 0 0"
                     /evidence=predicted
                     /translation="QMVRQLQQDYSIRQICQVLNYPRSQVYYHARG--QPD-----ES
                     ELKAAI-AGVAGAYPTYGYRRITAQLQ-RQGYCVNHKRVARLMRQIGIMAKTKVKRKR
                     TTNSEHSfPRYGNRVLNLS-IDHPEQVWVADITYIRLQQEFVYLAVVMDVFTRAIRGW
                     HLSRHIDQQLTLRALNKALERA-TPE--IHHSDQGVQYAAAAYMQLLQQHQVQISMAE
                     VGQAWQNGYAERLMRTIK-EEEVDL-SDYRNFTEAYEHIEQFLEDVYMHKRIHSSLGY
                     LTPCEYEQQWR"
                     /note="IS3_IS3_ORF1      e-value= 9.5e-54 complete sequence
                     hit coverage= 100%, between model( 286 aa) positions 1;
                     286 length is 272aa with 15 gaps, 0 stops, absolute
                     frame= Plus2"
     CDS             2501078..2501893
                     /colour="255 0 0"
                     /evidence=predicted
                     /translation="QMVRQLQQDYSIRQICQVLNYPRSQVYYHARG--QPD-----ES
                     ELKAAI-AGVAGAYPTYGYRRITAQLQ-RQGYCVNHKRVARLMRQIGIMAKTKVKRKR
                     TTNSEHSfPRYGNRVLNLS-IDHPEQVWVADITYIRLQQEFVYLAVVMDVFTRAIRGW
                     HLSRHIDQQLTLRALNKALERA-TPE--IHHSDQGVQYAAAAYMQLLQQHQVQISMAE
                     VGQAWQNGYAERLMRTIK-EEEVDL-SDYRNFTEAYEHIEQFLEDVYMHKRIHSSLGY
                     LTPCEYEQQWR"
                     /note="IS3_IS3_ORF1      e-value= 2.5e-54 complete sequence
                     hit coverage= 100%, between model( 286 aa) positions 1;
                     286 length is 272aa with 15 gaps, 0 stops, absolute
```

```
                     frame= Plus2"
     CDS             2501081..2501896
                     /colour="255 0 0"
                     /evidence=predicted
                     /translation="MVRQLQQDYSIRQICQVLNYPRSQVYYHARGQPDESELKAAIAG
                     V-AGAYPTYGYRRITAQLQRQGYCVNHKRVARLMRQIGIMAKTKVK----RKRTTNSE
                     HSF-PRYGNRvlNLSIDHPEQVWVADITYIRL--QQEFVYLAVVMDVFTRAIRGWHLS
                     RHIDQQLTLRALNKALER----ATPEIHHSDQGVQYAAAAYMQLLQQHQVQISMAEVG
                     QAWQNGYAERLMRTIKEEEVDLSDYRNFT--EAYEHIEQFLEDVYMHKRIHSSLGYLT
                     PCEYEQQWRQ"
                     /note="IS3_IS150_ORF1    e-value= 6.3e-50 fragment hit
                     coverage= 92.81%, between model( 306 aa) positions 23;
                     306 length is 272aa with 14 gaps, 0 stops, absolute
                     frame= Plus2"
     CDS             2501090..2501905
                     /colour="255 0 0"
                     /evidence=predicted
                     /translation="QLQQDYSIRQICQVLNYPRSQVYYHARGQPDE---------SEL
                     KAAIAGVAGaypTYGYRRITAQLQRQGYCVNHKRVARLMRQIGIMAKTKVKRKRTTNS
                     EHSFPRY---GNRVLNLSIDHPEQVWVADITYIRLQQEFVYLAVVMDVFTRAIRGWHL
                     SRHIDQQLTLRALNKALER----ATPE---IHHSDQGVQYAAAAYMQLLQQHQVQISM
                     AEVGQAWQNGYAERLMRTIKEEEVDLSDYRNFTEAYEHIEQFLEDVYMHKRIHSSLGY
                     LTPCEYEQQWRQQNN"
                     /note="IS3_IS3_ORF2      e-value= 1.2e-64 complete sequence
                     hit coverage= 100%, between model( 288 aa) positions 1;
                     288 length is 272aa with 19 gaps, 0 stops, absolute
                     frame= Plus2"
     CDS             2501090..2501905
                     /colour="255 0 0"
                     /evidence=predicted
                     /translation="QLQQDYSIRQICQVLNYPRSQVYYHARGQPDE---------SEL
                     KAAIAGVAGaypTYGYRRITAQLQRQGYCVNHKRVARLMRQIGIMAKTKVKRKRTTNS
                     EHSFPRY---GNRVLNLSIDHPEQVWVADITYIRLQQEFVYLAVVMDVFTRAIRGWHL
                     SRHIDQQLTLRALNKALER----ATPE---IHHSDQGVQYAAAAYMQLLQQHQVQISM
                     AEVGQAWQNGYAERLMRTIKEEEVDLSDYRNFTEAYEHIEQFLEDVYMHKRIHSSLGY
                     LTPCEYEQQWRQQNN"
                     /note="IS3_IS3_ORF2      e-value= 3.3e-65 complete sequence
                     hit coverage= 100%, between model( 288 aa) positions 1;
                     288 length is 272aa with 19 gaps, 0 stops, absolute
                     frame= Plus2"
     CDS             2501159..2501317
                     /colour="255 0 0"
                     /evidence=predicted
                     /translation="YHARgQPDESELKAAIAGVAGAYPTYGYRRITAQLQRQGY----
                     -CVNHKRVARLMRQ"
                     /note="IS3_IS2_ORF2      e-value= 9.1e-09 fragment hit
                     coverage= 18.87%, between model( 302 aa) positions 48;
                     104 length is 53aa with 5 gaps, 0 stops, absolute frame=
                     Plus2"
     CDS             2501225..2501908
                     /colour="255 0 0"
                     /evidence=predicted
                     /translation="YPTYGYRRITAQLQRQGYCVNHKRVARLMRQIGIMAKTKVKRKR
                     TTNSEHSFPRYGNRVL-NLSIDHPEQVWVADITYIRLQQEFVYLAVVMDVFTRAIRGW
                     HLSRHIDQQLTLRALNKALERATPE--------IHHSDQGVQYAAAAYMQLLQQHQVQ
                     ISMAEVGQAWQNGYAERLMRTIKEEEVDLSDYrnFTEAYEHIEQFLEDV--YMHKRIH
                     SSLGYLTPCEYEQQWRQQNNH"
                     /note="IS3_IS51_ORF2     e-value= 1.9e-38 fragment hit
                     coverage= 74.76%, between model( 317 aa) positions 73;
                     309 length is 228aa with 11 gaps, 0 stops, absolute
```

frame= Plus2"
CDS 2629103..2629798
/colour="255 0 0"
/evidence=predicted
/translation="FKHLHLGMIsDIKRKSLPAIARAVGLENHQSLHHFLSESPWVAS
hLSDkRLDIILKILDGRRLILLID------------------ETGDCKKGKSTDYVKR
QYIGNVGKKENGIvAVTAYGLFQGMILPLSFEVYKPR---------ErLKEEDEYLSK
PQIAAGMIRQLQGMGFCFELVLADSLYGEAQTNFVNVLEELKLPYILAIRSNHAVWLP
AEQAVFAEPWQSFERTFSNGTTETRYrQEIIYGqrhhQKRY"
/note="IS4 e-value= 4.7e-14 fragment hit coverage=
51.23%, between model(486 aa) positions 1; 249 length is
232aa with 27 gaps, 0 stops, absolute frame= Plus2"
CDS 2629103..2630230
/colour="255 0 0"
/evidence=predicted
/translation="FKHLHLGMIsDIKRKSLPAIARAVGLENHQSLHHFLSESPWVAS
hLSDkRLDIILKILDGRRLILLID------------------ETGDCKKGKSTDYVKR
QYIGNVGKKENGIvAVTAYGLFQGMILPLSFEVYKPR---------ErLKEEDEYLSK
PQIAAGMIRQLQGMGFCFELVLADSLYGEAQTNFVNVLEELKLPYILAIRSNHAVWLP
AEQAVFAEPWQSFERTFSNGT--------------------------------TETRYR
QEIIYGQRHHQKRyWLLTTDPQTLPENSTS------------------------YVMAA
APE----IKLDEIGDCYGFRTWIEYGLKQSKD-TLGWADFRMTHYEQIEKWWEIVMSA
FLMVSLFADVFNDS-------------------------CPVAHQHFAQHPwWDNQNG
WKNLLNNVR--LIIQPLIS----WNLLKRWLEVFPSRALKKGF"
/note="IS4 e-value= 9.9e-07 complete sequence hit
coverage= 100%, between model(486 aa) positions 1; 486
length is 376aa with 117 gaps, 0 stops, absolute frame=
Plus2"
CDS 2631026..2631439
/colour="255 0 0"
/evidence=predicted
/translation="GRQRFNVLGALNAVTS---ITNHTYINSHSMCLLLAKLALLDPV
I--PISVILDNARYQKCQLVTDFAEIVD--IELVYLPSYSPHLNLIERLWRFVRKECL
YSKYYSDFHSFKGAIQQCIDQCNTEHKAKLTSLLSLKFQSFQK"
/note="IS630 e-value= 1.1e-17 fragment hit coverage=
38.56%, between model(376 aa) positions 232; 376 length
is 138aa with 7 gaps, 0 stops, absolute frame= Plus2"
CDS 2714843..2715040
/colour="255 0 0"
/evidence=predicted
/translation="GKHKQILLVLDGAGWHTC---KNRVVPPG--IHLKILPPYSPEL
QPAERLWRLADEPLAN-QCFETLDDLED"
/note="IS630 e-value= 1.4e-09 fragment hit coverage=
19.15%, between model(376 aa) positions 275; 346 length
is 66aa with 6 gaps, 0 stops, absolute frame= Plus2"
CDS 2845484..2846386
/colour="255 0 0"
/evidence=predicted
/translation="QRFIQGLSPETIHLLSRIHRHSYHHQVRQRAHCILLSFEGFNVT
ELMSIFAVTRKTVYTWLDDWDNHC---LVGLYDQPGRGRKPKLNDVQ-KEQIRAWAKM
TP---------HNLNAVLAKIKEAWNIAVSKTTLKRILKSCSMSWRRLRRVVAGQPDP
VEYETK-----RHQLEVLKRQEEKGELdlrYLDESGFCLVPYVPY-AWQEKG------
--------ETLGLPSQRSSRFNVLGLMNRHNDL-TSYVFDKSITSAVVVACIDDFSRT
C--DQHTVVVMDQASVHKNAEIEEKIEDWKAkNVEIFWLPTYSPHLNLIEIFWRFMKY
EWI"
/note="IS630 e-value= 1.1e-24 fragment hit coverage=
88.56%, between model(376 aa) positions 1; 333 length is
301aa with 36 gaps, 0 stops, absolute frame= Plus2"
CDS 2845484..2846518
/colour="255 0 0"

/evidence=predicted
/translation="QRFIQGLSPETIHLLSRIHRHSYHHQVRQRAHCILLSFEGFNVT
ELMSIFAVTRKTVYTWLDDWDNHC---LVGLYDQPGRGRKPKLNDVQ-KEQIRAWAKM
TP---------HNLNAVLAKIKEAWNIAVSKTTLKRILKSCSMSWRRLRRVVAGQPDP
VEYETK-----RHQLEVLKRQEEKGELdlrYLDESGFCLVPYVPY-AWQEKG------
--------ETLGLPSQRSSRFNVLGLMNRHNDL-TSYVFDKSITSAVVVACIDDFSRT
C--DQHTVVVMDQASVHKNAEIEEKIEDWKAkNVEIFWLPTYSPHLNLIEIFWRFMKY
EWIEFAAYKCLGSLSLYIDKILKGFgKDYVIDFGXVLINFXLTTQLL"
/note="IS630 e-value= 5.3e-21 complete sequence hit
coverage= 100%, between model(376 aa) positions 1; 376
length is 345aa with 36 gaps, 2 stops, absolute frame=
Plus2"
CDS 3258380..3259537
/colour="255 0 0"
/evidence=predicted
/translation="SSNSFRAKTPSIXKVG-ILNSNHQIT---------FHVSSTQVF
ACCPVCNQITHRVHSRYERTLRDLPCVDFCLTILLQVCKFFCRNeaCKRCIFTErlpk
vaVPWARKTIRFAEHLTSIGLALGGAAAARLSYQINYGSSRNT----MLRSIAKLEPP
-PAPTPKILGVDDFAFQRG--HHYGTILVDLEHH----RTIALLPDRDAKTLVAWLEE
HP------GVEILSRDRSKTYKSAISEGAPNAIQVADRFHLLKNLQEVLEKVF-----
------------------HSNYPALKSVDAALLKS-EVPEPHAPESNEKSKVP-PDE
QgPRSRAH-----RLNNYQQTHALKQQGYXVTDIAHHLGIAKRTAYQ-YLSHPFFPEH
QPYTRKVPTVIEP-YKDY---------LYQEWMAGRQNSYCTK------YRdALIGHK
SIKCVKTNDLD"
/note="ISL3 e-value= 4e-07 complete sequence hit
coverage= 100%, between model(451 aa) positions 1; 451
length is 386aa with 75 gaps, 2 stops, absolute frame=
Plus2"
CDS 3258827..3259108
/colour="255 0 0"
/evidence=predicted
/translation="TPKILGVDDFAFQRG--HHYGTILVDLEHH----RTIALLPDRD
AKTLVAWLEEHP------GVEILSRDRSKTYKSAISEGAPNAIQVADRFHLLKNLQEV
LEKV"
/note="ISL3 e-value= 1.4e-18 fragment hit coverage=
23.50%, between model(451 aa) positions 157; 262 length
is 94aa with 12 gaps, 0 stops, absolute frame= Plus2"
CDS 3307874..3309085
/colour="255 0 0"
/evidence=predicted
/translation="DELIGENPQPEdiLGESGLLKRLSKRLVERALAGELTHHLQQSS
NDQS-GDeNSNGPRNSRNGYSKKTVQSEQG-EMDLSIPRDRCG-EFEPVLVPKGQRRI
A-GLDEKIIALYARGMTTRDIRAQLVELYG--ANISEALISDVTNSVMEEVKDWRSRP
LDEV--YPIVYLDALYVNIKVNGQVSKRAVYVALAVTVEGNKELLGLWIGAAerEGAK
FWLSVLTDLKNRGTQDILIACCDGLKGFPQAIESVYPQTQVQVCIVHLIRNSLRHVPW
KESRAVAADLKPIYQAATLE-ESEAALDAFAH-KWDETYPGISQIWIRHWDNVAPLFQ
YPMAIRKVIYTTNAIESLNRSLRKVIKTKAVFPNEESVYKLMFLAMRNISKR-WTRPI
LNWKAALSHFAILFPTRFNYXIH"
/note="IS256 e-value= 1.4e-166 complete sequence hit
coverage= 100%, between model(410 aa) positions 1; 410
length is 404aa with 11 gaps, 1 stops, absolute frame=
Plus2"
CDS 3307874..3309085
/colour="255 0 0"
/evidence=predicted
/translation="DELIGENPQPEdiLGESGLLKRLSKRLVERALAGELTHHLQQSS
NDQS-GDeNSNGPRNSRNGYSKKTVQSEQG-EMDLSIPRDRCG-EFEPVLVPKGQRRI
A-GLDEKIIALYARGMTTRDIRAQLVELYG--ANISEALISDVTNSVMEEVKDWRSRP
LDEV--YPIVYLDALYVNIKVNGQVSKRAVYVALAVTVEGNKELLGLWIGAAerEGAK
FWLSVLTDLKNRGTQDILIACCDGLKGFPQAIESVYPQTQVQVCIVHLIRNSLRHVPW

```
                     KESRAVAADLKPIYQAATLE-ESEAALDAFAH-KWDETYPGISQIWIRHWDNVAPLFQ
                     YPMAIRKVIYTTNAIESLNRSLRKVIKTKAVFPNEESVYKLMFLAMRNISKR-WTRPI
                     LNWKAALSHFAILFPTRFNYXIH"
                     /note="IS256 e-value= 3.7e-167 complete sequence hit
                     coverage= 100%, between model( 410 aa) positions 1; 410
                     length is 404aa with 11 gaps, 1 stops, absolute frame=
                     Plus2"
     CDS             3753371..3754582
                     /colour="255 0 0"
                     /evidence=predicted
                     /translation="DELIGENPQPEdiLGESGLLKRLSKRLVERALAGELTHHLQQSS
                     NDQS-GDeNSNGPRNSRNGYSKKTVQSEQG-EMDLSIPRDRCG-EFEPVLVPKGQRRI
                     A-GLDEKIIALYARGMTTRDIRAQLVELYG--ANISEALISDVTNSVMEEVKDWRSRP
                     LDEV--YPIVYLDALYVNIKVNGQVSKRAVYVALAVTVEGNKELLGLWIGAAerEGAK
                     FWLSVLTDLKNRGTQDILIACCDGLKGFPQAIESVYPQTQVQVCIVHLIRNSLRHVPW
                     KESRAVAADLKPIYQAATLE-ESEAALDAFAH-KWDETYPGISQIWIRHWDNVAPLFQ
                     YPMAIRKVIYTTNAIESLNRSLRKVIKTKAVFPNEESVYKLMFLAMRNISKR-WTRPI
                     LNWKAALSHFAILFPTRFNYXIH"
                     /note="IS256 e-value= 1.4e-166 complete sequence hit
                     coverage= 100%, between model( 410 aa) positions 1; 410
                     length is 404aa with 11 gaps, 1 stops, absolute frame=
                     Plus2"
     CDS             3753371..3754582
                     /colour="255 0 0"
                     /evidence=predicted
                     /translation="DELIGENPQPEdiLGESGLLKRLSKRLVERALAGELTHHLQQSS
                     NDQS-GDeNSNGPRNSRNGYSKKTVQSEQG-EMDLSIPRDRCG-EFEPVLVPKGQRRI
                     A-GLDEKIIALYARGMTTRDIRAQLVELYG--ANISEALISDVTNSVMEEVKDWRSRP
                     LDEV--YPIVYLDALYVNIKVNGQVSKRAVYVALAVTVEGNKELLGLWIGAAerEGAK
                     FWLSVLTDLKNRGTQDILIACCDGLKGFPQAIESVYPQTQVQVCIVHLIRNSLRHVPW
                     KESRAVAADLKPIYQAATLE-ESEAALDAFAH-KWDETYPGISQIWIRHWDNVAPLFQ
                     YPMAIRKVIYTTNAIESLNRSLRKVIKTKAVFPNEESVYKLMFLAMRNISKR-WTRPI
                     LNWKAALSHFAILFPTRFNYXIH"
                     /note="IS256 e-value= 3.7e-167 complete sequence hit
                     coverage= 100%, between model( 410 aa) positions 1; 410
                     length is 404aa with 11 gaps, 1 stops, absolute frame=
                     Plus2"
     CDS             3911570..3912511
                     /colour="255 0 0"
                     /evidence=predicted
                     /translation="TKNRXPMSYQIRNWSEYNAGLKQRGSVTFWLEQS--AIVHWLET
                     TPRQKRGASLTYSDTAISTFETVKCIYGLAGRQTEGFLNSLFELMGIELPVCDHSTVS
                     RRKGQLSISLPVIPKQGAIHVVIDSTGVKVYG------------EGEWKTRQHGVS-K
                     RRTWRKLHLSVDESTGEILTGVVTTNDVQDGEVFEDLLEGIDD-----EIEQVSAD-G
                     AYDQSHCYDALMERNATAAIPPRKNA-------------KIWQHGNC--NApphpRDQ
                     NLRAIRKQGRKRWKKQAHYHRRSLAETTMFRFKTIFGGQVRSRNFDNQATELLLQCAA
                     LN-RMIQIAKPETVWVE"
                     /note="IS5_IS903   e-value= 1.1e-96 complete sequence hit
                     coverage= 100%, between model( 347 aa) positions 1; 347
                     length is 314aa with 37 gaps, 1 stops, absolute frame=
                     Plus2"
     CDS             3911570..3912511
                     /colour="255 0 0"
                     /evidence=predicted
                     /translation="TKNRXPMSYQIRNWSEYNAGLKQRGSVTFWLEQS--AIVHWLET
                     TPRQKRGASLTYSDTAISTFETVKCIYGLAGRQTEGFLNSLFELMGIELPVCDHSTVS
                     RRKGQLSISLPVIPKQGAIHVVIDSTGVKVYG------------EGEWKTRQHGVS-K
                     RRTWRKLHLSVDESTGEILTGVVTTNDVQDGEVFEDLLEGIDD-----EIEQVSAD-G
                     AYDQSHCYDALMERNATAAIPPRKNA-------------KIWQHGNC--NApphpRDQ
                     NLRAIRKQGRKRWKKQAHYHRRSLAETTMFRFKTIFGGQVRSRNFDNQATELLLQCAA
```

```
                     LN-RMIQIAKPETVWVE"
                     /note="IS5_IS903   e-value= 2.2e-96 complete sequence hit
                     coverage= 100%, between model( 347 aa) positions 1; 347
                     length is 314aa with 37 gaps, 1 stops, absolute frame=
                     Plus2"
     CDS             4126139..4127350
                     /colour="255 0 0"
                     /evidence=predicted
                     /translation="DELIGENPQPEdiLGESGLLKRLSKRLVERALAGELTHHLQQSS
                     NDQS-GDeNSNGPRNSRNGYSKKTVQSEQG-EMDLSIPRDRCG-EFEPVLVPKGQRRI
                     A-GLDEKIIALYARGMTTRDIRAQLVELYG--ANISEALISDVTNSVMEEVKDWRSRP
                     LDEV--YPIVYLDALYVNIKVNGQVSKRAVYVALAVTVEGNKELLGLWIGAAerEGAK
                     FWLSVLTDLKNRGTQDILIACCDGLKGFPQAIESVYPQTQVQVCIVHLIRNSLRHVPW
                     KESRAVAADLKPIYQAATLE-ESEAALDAFAH-KWDETYPGISQIWIRHWDNVAPLFQ
                     YPMAIRKVIYTTNAIESLNRSLRKVIKTKAVFPNEESVYKLMFLAMRNISKR-WTRPI
                     LNWKAALSHFAILFPTRFNYXIH"
                     /note="IS256  e-value= 1.4e-166 complete sequence hit
                     coverage= 100%, between model( 410 aa) positions 1; 410
                     length is 404aa with 11 gaps, 1 stops, absolute frame=
                     Plus2"
     CDS             4126139..4127350
                     /colour="255 0 0"
                     /evidence=predicted
                     /translation="DELIGENPQPEdiLGESGLLKRLSKRLVERALAGELTHHLQQSS
                     NDQS-GDeNSNGPRNSRNGYSKKTVQSEQG-EMDLSIPRDRCG-EFEPVLVPKGQRRI
                     A-GLDEKIIALYARGMTTRDIRAQLVELYG--ANISEALISDVTNSVMEEVKDWRSRP
                     LDEV--YPIVYLDALYVNIKVNGQVSKRAVYVALAVTVEGNKELLGLWIGAAerEGAK
                     FWLSVLTDLKNRGTQDILIACCDGLKGFPQAIESVYPQTQVQVCIVHLIRNSLRHVPW
                     KESRAVAADLKPIYQAATLE-ESEAALDAFAH-KWDETYPGISQIWIRHWDNVAPLFQ
                     YPMAIRKVIYTTNAIESLNRSLRKVIKTKAVFPNEESVYKLMFLAMRNISKR-WTRPI
                     LNWKAALSHFAILFPTRFNYXIH"
                     /note="IS256  e-value= 3.7e-167 complete sequence hit
                     coverage= 100%, between model( 410 aa) positions 1; 410
                     length is 404aa with 11 gaps, 1 stops, absolute frame=
                     Plus2"
     CDS             4199279..4199470
                     /colour="255 0 0"
                     /evidence=predicted
                     /translation="ATPIELSQQQHHCLLQIVRQTINPYRLVRRASIILSAASGKSNT
                     QISRQWQLDRNQVRYWRQRW"
                     /note="IS630  e-value= 8.9e-08 fragment hit coverage=
                     17.02%, between model( 376 aa) positions 1; 64 length is
                     64aa with 0 gaps, 0 stops, absolute frame= Plus2"
     CDS             4226972..4227316
                     /colour="255 0 0"
                     /evidence=predicted
                     /translation="KYIVTLTPEERSELIQLTRRRTLSARKMKRAQILMLADEGHKDD
                     TITQMLNAGISTVHRTRQKFVEGG---VEfALNERPRPGGQKKLDSKAEALLIATACS
                     DPPTG----CCRWTMQLLAE"
                     /note="IS630  e-value= 1.2e-15 fragment hit coverage=
                     32.18%, between model( 376 aa) positions 1; 121 length is
                     115aa with 7 gaps, 0 stops, absolute frame= Plus2"
     CDS             4926788..4927030
                     /colour="255 0 0"
                     /evidence=predicted
                     /translation="VKLAELIPWESFESEYADQFSQ---TMGAPAKPFRVALGTLIIK
                     EKLGISDAETVEQIRENPYLQYFLGFSEYRESAPFDASML"
                     /note="IS5_IS5     e-value= 2.6e-06 fragment hit coverage=
                     19.91%, between model( 422 aa) positions 23; 106 length
                     is 81aa with 3 gaps, 0 stops, absolute frame= Plus2"
```

```
CDS             5053775..5054902
                /colour="255 0 0"
                /evidence=predicted
                /translation="PFASIIEHFSDLDDPRAAHRIEYSLEDIIFITLCAVLCGADNWV
                EVANYGCSKAQWLKQWIALPNG------IPSHDTFEWVFARLKPQQLQQCFLNWTQAI
                YHLSAGA-LIAIDGKTLRGAIATGEQRSLIHMVSAWASHNRLVLGQRTVEEKSNEITA
                IPELLKILELEG-ALVSIDAMGCQTAIAETIIEGQG-DYVLALKGNQGDLYNDVVQLF
                dhaCQTQFQGIEHDSYQTVEKGHGRIEHRTYWTMGQTDYLLGAE-RWAQLKSIGCVES
                CRRQPGHPG---TLQRRYYLLSIESDAQR---FADAVRSHWGIENQLHWILDVGFRED
                KLRACQGYSAQNLSVIRHIAANLLQQESTAKCGVKAKRLKAGWDDDYLVKILSAAAKG
                "
                /note="ISAs1 e-value= 1.9e-150 complete sequence hit
                coverage= 100%, between model( 389 aa) positions 1; 389
                length is 376aa with 16 gaps, 0 stops, absolute frame=
                Plus2"
CDS             5053775..5054902
                /colour="255 0 0"
                /evidence=predicted
                /translation="PFASIIEHFSDLDDPRAAHRIEYSLEDIIFITLCAVLCGADNWV
                EVANYGCSKAQWLKQWIALPNG------IPSHDTFEWVFARLKPQQLQQCFLNWTQAI
                YHLSAGA-LIAIDGKTLRGAIATGEQRSLIHMVSAWASHNRLVLGQRTVEEKSNEITA
                IPELLKILELEG-ALVSIDAMGCQTAIAETIIEGQG-DYVLALKGNQGDLYNDVVQLF
                dhaCQTQFQGIEHDSYQTVEKGHGRIEHRTYWTMGQTDYLLGAE-RWAQLKSIGCVES
                CRRQPGHPG---TLQRRYYLLSIESDAQR---FADAVRSHWGIENQLHWILDVGFRED
                KLRACQGYSAQNLSVIRHIAANLLQQESTAKCGVKAKRLKAGWDDDYLVKILSAAAKG
                "
                /note="ISAs1 e-value= 1.5e-144 complete sequence hit
                coverage= 100%, between model( 389 aa) positions 1; 389
                length is 376aa with 16 gaps, 0 stops, absolute frame=
                Plus2"
CDS             5055743..5055940
                /colour="255 0 0"
                /evidence=predicted
                /translation="ASPIELSQQQHHCLTQIVRQTTNPYRLVRRASIILSAASGESNT
                QISRQWQLDRNQVRYWRQRWLD"
                /note="IS630 e-value= 4e-07 fragment hit coverage=
                17.55%, between model( 376 aa) positions 1; 66 length is
                66aa with 0 gaps, 0 stops, absolute frame= Plus2"
CDS             5176034..5176441
                /colour="255 0 0"
                /evidence=predicted
                /translation="MLLVRPISTESLRLLHRIYHSSRHHQVRQRAHCLILFAQGWPPY
                TLASLFSVSPKTVYNWLKAWNNRG---FAGLYNHPGRGRKPMFNPDQQQQIYEWT-QA
                SPIQ---------LNQVLAQIEQQWSVRVSKATVKRVLKQMDMSWHR"
                /note="IS630 e-value= 8.7e-15 fragment hit coverage=
                39.63%, between model( 376 aa) positions 1; 149 length is
                136aa with 13 gaps, 0 stops, absolute frame= Plus2"
CDS             5176094..5176432
                /colour="255 0 0"
                /evidence=predicted
                /translation="SSRHHQVRQRAHCLILFAQGWPPYTLASLFSVSPKTVYNWLKAW
                NNRGFAGLYNH---PGRGRKPMfNPDQQQQIYEWTQASPIQLNQVLAQIEQQWSVR--
                -VSKATVKRVLKQMDMS"
                /note="IS481 e-value= 3.5e-09 fragment hit coverage=
                33.62%, between model( 351 aa) positions 1; 118 length is
                113aa with 6 gaps, 0 stops, absolute frame= Plus2"
CDS             5341466..5341705
                /colour="255 0 0"
                /evidence=predicted
                /translation="RNQQHYIRQRLTAIKLLNEGHSRTQVSEQVGCSYDTLTRWMDKY
```

```
                     LDGGLQGLVQPI--RHQKPSRLSPEEQQQLKEMVLTQR"
                     /note="IS481 e-value= 1.2e-07 fragment hit coverage=
                     23.36%, between model( 351 aa) positions 1; 82 length is
                     80aa with 2 gaps, 0 stops, absolute frame= Plus2"
     CDS             5341493..5341900
                     /colour="255 0 0"
                     /evidence=predicted
                     /translation="RLTAIKLLNEGHSRTQVSEQVGCSYDTLTRWMDKYLDGG---LQ
                     GLVQPIRHQKPSRLSPEEQQQLKEMVLTQRPTDYGIDRNMWTGAILAVVIEQRFEVQL
                     KDSRIYELLSELGLSYQRAHRDYA--NADLNAQKEWVAA"
                     /note="IS630 e-value= 1.9e-29 fragment hit coverage=
                     37.50%, between model( 376 aa) positions 30; 170 length
                     is 136aa with 5 gaps, 0 stops, absolute frame= Plus2"
     CDS             5545037..5545732
                     /colour="255 0 0"
                     /evidence=predicted
                     /translation="FKHLHLGMInDIKRKSLPAIARAVGLENHQSLHHFLSESPWVAS
                     hLSDkSLDIILKILDGRRLILLID------------------ETGDCKKGKSTDYVKR
                     QYIGNVGKKENGIvAVTAYGLFQGMILPLSFEVYKPR---------ErLKEGDEYLSK
                     PQIAAGMIRQLQGMGFCFELVLADSLYGAAQTNFVNVLEELKLPYILAIRSNHAVWLP
                     AEQAVFAEPWQSFERTFSNGTTETRYrQEIIYGqrhhQKRY"
                     /note="IS4   e-value= 2.3e-13 fragment hit coverage=
                     51.23%, between model( 486 aa) positions 1; 249 length is
                     232aa with 27 gaps, 0 stops, absolute frame= Plus2"
     CDS             5545037..5546200
                     /colour="255 0 0"
                     /evidence=predicted
                     /translation="FKHLHLGMInDIKRKSLPAIARAVGLENHQSLHHFLSESPWVAS
                     hLSDkSLDIILKILDGRRLILLID------------------ETGDCKKGKSTDYVKR
                     QYIGNVGKKENGIvAVTAYGLFQGMILPLSFEVYKPR---------ErLKEGDEYLSK
                     PQIAAGMIRQLQGMGFCFELVLADSLYGAAQTNFVNVLEELKLPYILAIRSNHAVWLP
                     AEQAVFAEPWQSFERTFSNGT--------------------------------TETRYR
                     QEIIYGQRHHQKRyWLLTTDPQTLPENSTS-----------------------YVMAA
                     APE----IKLDEIGDCYGFRTWIEYGLKQSKD-TLGWADFRMTHYEQIEKWWEIVMSA
                     FLMVSLFADVFNDSCP-----------------VANQHFaQHPWWDNQNGWKNLLNNV
                     RLIIQPLISWNLLKSWLE--VFPIRALKKGFEQLTSKMEAFCC"
                     /note="IS4   e-value= 1.2e-06 complete sequence hit
                     coverage= 100%, between model( 486 aa) positions 1; 486
                     length is 388aa with 105 gaps, 0 stops, absolute frame=
                     Plus2"
     CDS             5653406..5653864
                     /colour="255 0 0"
                     /evidence=predicted
                     /translation="MTHRKLSHSVWECKYHVVFVPKYRKGRIYGQIRXNSGDIFH-EL
                     ARHKKNVIEEGHLMPDHVHMLLSIPLKYSVSNVVGYVKGKSAIYIARELGNSRNA-TG
                     HKFWSRGYFVSTVG-RDEAMIRRYIRHQGQEEQKLEQLNLFraXPKSPLRGSNS"
                     /note="IS200 e-value= 6.3e-51 complete sequence hit
                     coverage= 100%, between model( 154 aa) positions 1; 154
                     length is 153aa with 3 gaps, 2 stops, absolute frame=
                     Plus2"
     CDS             5653406..5653864
                     /colour="255 0 0"
                     /evidence=predicted
                     /translation="MTHRKLSHSVWECKYHVVFVPKYRKGRIYGQIRXNSGDIFH-EL
                     ARHKKNVIEEGHLMPDHVHMLLSIPLKYSVSNVVGYVKGKSAIYIARELGNSRNA-TG
                     HKFWSRGYFVSTVG-RDEAMIRRYIRHQGQEEQKLEQLNLFraXPKSPLRGSNS"
                     /note="IS200 e-value= 5.1e-50 complete sequence hit
                     coverage= 100%, between model( 154 aa) positions 1; 154
                     length is 153aa with 3 gaps, 2 stops, absolute frame=
                     Plus2"
```

```
CDS             5653406..5653861
                /colour="255 0 0"
                /evidence=predicted
                /translation="MTHRKLSHSVWECKYHVVFVPKYRKGRIYGQ-IRXNSGDIFHEL
                ARHKKNVIEEGHLMPDHVHMLLSIPLKYSVSNVVGYVKGKSAIYIARELGNSRNA-TG
                HKFWSRGYFVSTVG-RDEAMIRRYIRHQGQEEQKLEQLNLFraXPKSPLRGSN"
                /note="IS200_IS605 e-value= 2.2e-50 complete sequence hit
                coverage= 100%, between model( 153 aa) positions 1; 153
                length is 152aa with 3 gaps, 2 stops, absolute frame=
                Plus2"
CDS             5653406..5653861
                /colour="255 0 0"
                /evidence=predicted
                /translation="MTHRKLSHSVWECKYHVVFVPKYRKGRIYGQ-IRXNSGDIFHEL
                ARHKKNVIEEGHLMPDHVHMLLSIPLKYSVSNVVGYVKGKSAIYIARELGNSRNA-TG
                HKFWSRGYFVSTVG-RDEAMIRRYIRHQGQEEQKLEQLNLFraXPKSPLRGSN"
                /note="IS200_IS605 e-value= 5.6e-51 complete sequence hit
                coverage= 100%, between model( 153 aa) positions 1; 153
                length is 152aa with 3 gaps, 2 stops, absolute frame=
                Plus2"
CDS             5653406..5653861
                /colour="255 0 0"
                /evidence=predicted
                /translation="-MTHRKLSHSVWECKYHVVFVPKYRKGRIYGQIRXNSGDIFHEL
                ARHKKNVIEEGHLMPDHVHMLLSIPLKYSVSNVVGYVKGKSAIYIARELGNSRN-ATG
                HKFWSRGYFVSTVGRDEAMIRRYIRHQGQEEQKLEQLNLfrAXPKSPLRGSN"
                /note="IS605 e-value= 6.9e-45 complete sequence hit
                coverage= 100%, between model( 152 aa) positions 1; 152
                length is 152aa with 2 gaps, 2 stops, absolute frame=
                Plus2"
CDS             5653406..5653861
                /colour="255 0 0"
                /evidence=predicted
                /translation="-MTHRKLSHSVWECKYHVVFVPKYRKGRIYGQIRXNSGDIFHEL
                ARHKKNVIEEGHLMPDHVHMLLSIPLKYSVSNVVGYVKGKSAIYIARELGNSRN-ATG
                HKFWSRGYFVSTVGRDEAMIRRYIRHQGQEEQKLEQLNLfrAXPKSPLRGSN"
                /note="IS605 e-value= 8.2e-45 complete sequence hit
                coverage= 100%, between model( 152 aa) positions 1; 152
                length is 152aa with 2 gaps, 2 stops, absolute frame=
                Plus2"
CDS             5747213..5747476
                /colour="255 0 0"
                /evidence=predicted
                /translation="LFYFLMQCPLCGHPK-THKHGKTSKGSQRYRCPHCQQTFS-Etf
                DTLCYRRQiSPETIQTILQAHVEGSSLRGLSRITGVAYNTCVSVVR"
                /note="IS1_ORF1    e-value= 1.7e-15 fragment hit coverage=
                36.40%, between model( 239 aa) positions 1; 87 length is
                88aa with 2 gaps, 0 stops, absolute frame= Plus2"
CDS             5896091..5896333
                /colour="255 0 0"
                /evidence=predicted
                /translation="VKLAELIPWESFESEYADQFSQ---TMGAPAKPFRVALGTLIIK
                EKLGISDAETVEQIRENPYLQYFLGFSEYRESAPFDASML"
                /note="IS5_IS5     e-value= 2.6e-06 fragment hit coverage=
                19.91%, between model( 422 aa) positions 23; 106 length
                is 81aa with 3 gaps, 0 stops, absolute frame= Plus2"
CDS             5908769..5909158
                /colour="255 0 0"
                /evidence=predicted
                /translation="FFMDESGFSLVPCIPY-GWQPIG-------------TYL-EIPT
```

RSSKRLNVLGFLSRRQGL--HAYTSEQTITSEVVShCIDTFFADV--ELPTVIVVDQA
PIHTSQsiyEMKAEWAERG--ITLFELPSYSPHLNLIERLWQFMKYQWI"
/note="IS630 e-value= 6.5e-11 fragment hit coverage= 39.10%, between model(376 aa) positions 187; 333 length is 130aa with 21 gaps, 0 stops, absolute frame= Plus2"

CDS 5942117..5942839
/colour="255 0 0"
/evidence=predicted
/translation="----MAHLSPESFXCRATSEVVPSRST---------ISGPSILa
risdLFPHCWRLGSLXIN---GLPYSPPGRDRS-----DELRTLP----PTRQETSNT
RIXAAXCCRDGCDXDTDXTSpKRTKSVLFRKKKRHTLKCQIIADRNTLEVICLSFGPG
RRHDFQIFKGSGIHIHPNTESLQDSGYQGIAAYHANSYVPFKKPQHGELTSLQREYNC
ALSQERMGIEHINRSLKISRILSERYRNRRCRYALRCNLIAALYNHEL"
/note="IS5_ISL2 e-value= 1e-38 complete sequence hit coverage= 100%, between model(260 aa) positions 1; 260 length is 241aa with 25 gaps, 6 stops, absolute frame= Plus2"

CDS 5942438..5942839
/colour="255 0 0"
/evidence=predicted
/translation="KKKRHTLKCQIIADRNTLEVICLSFGPGRRHDFQIFKGSGIHIH
PNTESLQDSGYQGIAAYHANSYVPFKKPQHGELTSLQREYNCALSQERMGIEHINRSL
KISRILSERYRNRRCRYALRCNLIAALYNHEL"
/note="IS5_ISL2 e-value= 2.1e-57 fragment hit coverage= 51.54%, between model(260 aa) positions 127; 260 length is 134aa with 0 gaps, 0 stops, absolute frame= Plus2"

CDS 6104879..6105265
/colour="255 0 0"
/evidence=predicted
/translation="YADESGMDHRDEYDY-AYGPKGE-----------RVYA-LKTG-
RRSGRVNMIAALRAKQL--MAPFTIEGACNRTVFEIWLercLIPMLKPGQKLVI--DN
ATFHKGGRIQELVEKAG--CEVWYLPPYSPDLNKIERSWSWIKSRIR"
/note="IS630 e-value= 6.9e-14 fragment hit coverage= 38.83%, between model(376 aa) positions 188; 333 length is 129aa with 20 gaps, 0 stops, absolute frame= Plus2"

CDS 6243806..6244048
/colour="255 0 0"
/evidence=predicted
/translation="VKLAELIPWESFESEYADQFSQ---TMGAPAKPFRVALGTLIIK
EKLGISDAETVEQIRENPYLQYFLGFSEYRESAPFDASML"
/note="IS5_IS5 e-value= 2.6e-06 fragment hit coverage= 19.91%, between model(422 aa) positions 23; 106 length is 81aa with 3 gaps, 0 stops, absolute frame= Plus2"

CDS 6501464..6501685
/colour="255 0 0"
/evidence=predicted
/translation="YASDLTAEQWELLEPLIPAAK-------PGGRPRTTDMLSVLNA
ILYLVVTGCQWRQLPHDFPCWSTVYSYFRRWRDDGTW"
/note="IS5_IS1031 e-value= 3.3e-39 fragment hit coverage= 27.46%, between model(295 aa) positions 20; 100 length is 74aa with 7 gaps, 0 stops, absolute frame= Plus2"

CDS 6501473..6501685
/colour="255 0 0"
/evidence=predicted
/translation="DLTAEQWELLEPLIPAAKP-GGRPRT-TDMLSVLNAILYLVVTG
CQWRQLPHDFPCWSTVYSYFRRWRDDGTW"
/note="IS5_IS427 e-value= 7.3e-19 fragment hit coverage= 24.66%, between model(296 aa) positions 125; 197 length is 71aa with 2 gaps, 0 stops, absolute frame= Plus2"

CDS 316002..316943

```
                     /colour="255 0 0"
                     /evidence=predicted
                     /translation="TKNRXPMSYQIRNWSEYNAGLKQRGSVTFWLEQS--AIVHWLET
                     TPRQKRGASLTYSDTAISTFETVKCIYGLAGRQTEGFLNSLFELMGIELPVCDHSTVS
                     RRKGQLSISLPVIPKQGAIHVVIDSTGVKVYG------------EGEWKTRQHGVS-K
                     RRTWRKLHLSVDESTGEILTGVVTTNDVQDGEVFEDLLEGIDD-----EIEQVSAD-G
                     AYDQSHCYDALMERNATAAIPPRKNA-------------KIWQHGNC--NApphpRDQ
                     NLRAIRKQGRKRWKKQAHYHRRSLAETTMFRFKTIFGGQVRSRNFDNQATELLLQCAA
                     LN-RMIQIAKPETVWVE"
                     /note="IS5_IS903   e-value= 1.1e-96 complete sequence hit
                     coverage= 100%, between model( 347 aa) positions 1; 347
                     length is 314aa with 37 gaps, 1 stops, absolute frame=
                     Plus3"
     CDS             316002..316943
                     /colour="255 0 0"
                     /evidence=predicted
                     /translation="TKNRXPMSYQIRNWSEYNAGLKQRGSVTFWLEQS--AIVHWLET
                     TPRQKRGASLTYSDTAISTFETVKCIYGLAGRQTEGFLNSLFELMGIELPVCDHSTVS
                     RRKGQLSISLPVIPKQGAIHVVIDSTGVKVYG------------EGEWKTRQHGVS-K
                     RRTWRKLHLSVDESTGEILTGVVTTNDVQDGEVFEDLLEGIDD-----EIEQVSAD-G
                     AYDQSHCYDALMERNATAAIPPRKNA-------------KIWQHGNC--NApphpRDQ
                     NLRAIRKQGRKRWKKQAHYHRRSLAETTMFRFKTIFGGQVRSRNFDNQATELLLQCAA
                     LN-RMIQIAKPETVWVE"
                     /note="IS5_IS903   e-value= 2.2e-96 complete sequence hit
                     coverage= 100%, between model( 347 aa) positions 1; 347
                     length is 314aa with 37 gaps, 1 stops, absolute frame=
                     Plus3"
     CDS             469320..469814
                     /colour="255 0 0"
                     /evidence=predicted
                     /translation="LEKTGIESKKKTYGYR--ERD--ETQR---QAFIERLQTKHP--
                     HQIVYVDEAGIDNRADYPY-GYCPVG-----------QRFYD-LKSG-KRTERVSFIA
                     ALKEGQL--FSPMTFEGSCNRLLFEAWLQQSLI-SQLQLgdVIVIDNASFHHGQRIEE
                     IVAEAG--CEIWYLPSYSPDLNKIERWWFVLKN"
                     /note="IS630  e-value= 3e-16 fragment hit coverage=
                     50.80%, between model( 376 aa) positions 140; 330 length
                     is 165aa with 28 gaps, 0 stops, absolute frame= Plus3"
     CDS             471168..471305
                     /colour="255 0 0"
                     /evidence=predicted
                     /translation="WQTDGWEGYSRQLPNE---TIHHVSKALTQRLERTNGILRQQAG
                     RWHRR"
                     /note="IS1_ORF2    e-value= 3.3e-13 fragment hit coverage=
                     20.68%, between model( 237 aa) positions 164; 212 length
                     is 46aa with 3 gaps, 0 stops, absolute frame= Plus3"
     CDS             516039..516734
                     /colour="255 0 0"
                     /evidence=predicted
                     /translation="FKHLHLGMIsDIKRKSLPAIARAVGLENHQSLHHFLSESPWVAS
                     hLSDkRLDIILKILDGRRLILLID------------------ETGDCKKGKSTDYVKR
                     QYIGNVGKKENGIvAVTAYGLFQGMILPLSFEVYKPR---------ErLKEEDEYLSK
                     PQIAAGMIRQLQGMGFCFELVLADSLYGEAQTNFVNVLEELKLPYILAIRSNHAVWLP
                     AEQAVFAEPWQSFERTFSNGTTETRYrQEIIYGqrhhQKRY"
                     /note="IS4   e-value= 4.7e-14 fragment hit coverage=
                     51.23%, between model( 486 aa) positions 1; 249 length is
                     232aa with 27 gaps, 0 stops, absolute frame= Plus3"
     CDS             516039..517166
                     /colour="255 0 0"
                     /evidence=predicted
                     /translation="FKHLHLGMIsDIKRKSLPAIARAVGLENHQSLHHFLSESPWVAS
```

```
                     hLSDkRLDIILKILDGRRLILLID------------------ETGDCKKGKSTDYVKR
                     QYIGNVGKKENGIvAVTAYGLFQGMILPLSFEVYKPR---------ErLKEEDEYLSK
                     PQIAAGMIRQLQGMGFCFELVLADSLYGEAQTNFVNVLEELKLPYILAIRSNHAVWLP
                     AEQAVFAEPWQSFERTFSNGT--------------------------------TETRYR
                     QEIIYGQRHHQKRyWLLTTDPQTLPENSTS-----------------------YVMAA
                     APE----IKLDEIGDCYGFRTWIEYGLKQSKD-TLGWADFRMTHYEQIEKWWEIVMSA
                     FLMVSLFADVFNDS-------------------------CPVAHQHFAQHPwWDNQNG
                     WKNLLNNVR--LIIQPLIS----WNLLKRWLEVFPSRALKKGF"
                     /note="IS4   e-value= 9.9e-07 complete sequence hit
                     coverage= 100%, between model( 486 aa) positions 1; 486
                     length is 376aa with 117 gaps, 0 stops, absolute frame=
                     Plus3"
     CDS             666744..667439
                     /colour="255 0 0"
                     /evidence=predicted
                     /translation="FKHLHLGMIsDIKRKSLPAIARAVGLENHQSLHHFLSESPWVAS
                     hLSDkRLDIILKILDGRRLILLID------------------ETGDCKKGKSTDYVKR
                     QYIGNVGKKENGIvAVTAYGLFQGMILPLSFEVYKPR---------ErLKEEDEYLSK
                     PQIAAGMIRQLQGMGFCFELVLADSLYGEAQTNFVNVLEELKLPYILAIRSNHAVWLP
                     AEQAVFAEPWQSFERTFSNGTTETRYrQEIIYGqrhhQKRY"
                     /note="IS4   e-value= 4.7e-14 fragment hit coverage=
                     51.23%, between model( 486 aa) positions 1; 249 length is
                     232aa with 27 gaps, 0 stops, absolute frame= Plus3"
     CDS             666744..667871
                     /colour="255 0 0"
                     /evidence=predicted
                     /translation="FKHLHLGMIsDIKRKSLPAIARAVGLENHQSLHHFLSESPWVAS
                     hLSDkRLDIILKILDGRRLILLID------------------ETGDCKKGKSTDYVKR
                     QYIGNVGKKENGIvAVTAYGLFQGMILPLSFEVYKPR---------ErLKEEDEYLSK
                     PQIAAGMIRQLQGMGFCFELVLADSLYGEAQTNFVNVLEELKLPYILAIRSNHAVWLP
                     AEQAVFAEPWQSFERTFSNGT--------------------------------TETRYR
                     QEIIYGQRHHQKRyWLLTTDPQTLPENSTS-----------------------YVMAA
                     APE----IKLDEIGDCYGFRTWIEYGLKQSKD-TLGWADFRMTHYEQIEKWWEIVMSA
                     FLMVSLFADVFNDS-------------------------CPVAHQHFAQHPwWDNQNG
                     WKNLLNNVR--LIIQPLIS----WNLLKRWLEVFPSRALKKGF"
                     /note="IS4   e-value= 9.9e-07 complete sequence hit
                     coverage= 100%, between model( 486 aa) positions 1; 486
                     length is 376aa with 117 gaps, 0 stops, absolute frame=
                     Plus3"
     CDS             690405..690542
                     /colour="255 0 0"
                     /evidence=predicted
                     /translation="WQTDGWEGYARQLPDE---VVHEVSKALTQRLERTNGIVRQQTG
                     RWHRR"
                     /note="IS1_ORF2    e-value= 1.8e-11 fragment hit coverage=
                     20.68%, between model( 237 aa) positions 164; 212 length
                     is 46aa with 3 gaps, 0 stops, absolute frame= Plus3"
     CDS             972294..973070
                     /colour="255 0 0"
                     /evidence=predicted
                     /translation="PYXXXEKAXISLVLEASKNAXISLLSXGwVQAPTTIRIAGLARL
                     IAWA--VCFANPASSXEL--PLYSGSFSLSI---------SKYRTGCLERLFEKXVKL
                     SRYXsVNTQTLDRTXRAMPTAYDSDLTTLQWELLEPLIPAAKP-GGRPRT-TDMLSVL
                     NAIFYLVVTGCQWRQLPHDFPCWSTVYSYFRRWRDDGTWSQINEHLRMQVRVSE----
                     -------------------------DRHPSPSAAICDAQSVkvgnpRCHLIGFDGGKM
                     VKGRKRHVLVDTLGLVLMVMVTAANIS"
                     /note="IS5_IS427   e-value= 8.6e-07 complete sequence hit
                     coverage= 100%, between model( 296 aa) positions 1; 296
                     length is 259aa with 44 gaps, 10 stops, absolute frame=
                     Plus3"
```

```
CDS             972543..973412
                /colour="255 0 0"
                /evidence=predicted
                /translation="EKXVKLSRYXSVNTQTLDRTXramptaYDSDLTTLQWELLEPLI
                PAAKPG-----GRPRTTDML--------------------SVLNAIFYLVVTGCQWRQ
                --LPHDFPCWSTVYSYFRRWRDDGTWSQINEHLRMQVRVSED---RHPSPSAAICDAQ
                SVK------------------------------VGNpRcHLIGFD--------GGKM-
                ------------------------------------------VKGRKRHVLVD-TL
                GLVLMVMVTAANISDQRGAK----------------------ILFWKAQRQGASLSRL
                VR----IWADAGYQGQALMKWVMDRFQYVLEVVKRSDNLAGFQVIPKRWIVERTFGWL
                LWSRRLNKDYEVLTRTAEALVYVAMIRLMVRRLAQEHX"
                /note="IS5_IS5     e-value= 1.8e-09 complete sequence hit
                coverage= 100%, between model( 422 aa) positions 1; 422
                length is 290aa with 140 gaps, 4 stops, absolute frame=
                Plus3"
CDS             972582..973433
                /colour="255 0 0"
                /evidence=predicted
                /translation="TQTLDRTXRAM-----PTAYDSDLTTLQWELLEPLIPAAK----
                ---PGGRPRTTDMLSVLNAIFYLVVTGCQWRQLPHDFPCWSTVYSYFRRWRDDGTWSQ
                INEHLRMQVRVSEDRHPSPSAAICDAQSVKVGNPRCH-LIGFDGGKMVKGRKRHVLVD
                TLGLVLMVMVTAANISDQR-GAKILFWKAQRQGaslSRLVRIWADAGYQGQALMKWVM
                DRFQYVLEVVKRSDNLAGFQVIPKRWIVERTFGWLLWSRRLNKDYEVLTRTAEALVYV
                AMIRLMVRRLAQEHXNFSNSLX"
                /note="IS5_IS1031  e-value= 1.8e-111 complete sequence hit
                coverage= 100%, between model( 295 aa) positions 1; 295
                length is 284aa with 14 gaps, 3 stops, absolute frame=
                Plus3"
CDS             972624..973433
                /colour="255 0 0"
                /evidence=predicted
                /translation="YDSDLTTLQWELLEPLIPAAK-------PGGRPRTTDMLSVLNA
                IFYLVVTGCQWRQLPHDFPCWSTVYSYFRRWRDDGTWSQINEHLRMQVRVSEDRHPSP
                SAAICDAQSVKVGNPRCH-LIGFDGGKMVKGRKRHVLVDTLGLVLMVMVTAANISDQR
                -GAKILFWKAQRQGaslSRLVRIWADAGYQGQALMKWVMDRFQYVLEVVKRSDNLAGF
                QVIPKRWIVERTFGWLLWSRRLNKDYEVLTRTAEALVYVAMIRLMVRRLAQEHXNFSN
                SLX"
                /note="IS5_IS1031  e-value= 1.2e-113 fragment hit
                coverage= 93.56%, between model( 295 aa) positions 20;
                295 length is 270aa with 9 gaps, 2 stops, absolute frame=
                Plus3"
CDS             972633..972872
                /colour="255 0 0"
                /evidence=predicted
                /translation="DLTTLQWELLEPLIPAAKP-GGRPRT-TDMLSVLNAIFYLVVTG
                CQWRQLPHDFPCWSTVYSYFRRWRDDGTWSQINEHLRM"
                /note="IS5_IS427   e-value= 2e-19 fragment hit coverage=
                27.70%, between model( 296 aa) positions 125; 206 length
                is 80aa with 2 gaps, 0 stops, absolute frame= Plus3"
CDS             1016775..1017812
                /colour="255 0 0"
                /evidence=predicted
                /translation="PMIKIDFTAEEIQQLNYERYHHPHPRVQRRMEVLYLKSQGLSHT
                QICQLCQISRPTLAKTLRQYQQGG---IEGLKTLEYKGQPSLLNAHS-DSVRAYFEQH
                PP---------RTSAEAQAVIEQLTGIKRSPTQIKAFLKRIGCRYRKVGYVPGKSSLP
                -EKIEEQEQFRHTRLEPLleeAQRQERLVFFVDAAHFVHRAYLGF-VWCITRI-----
                ---------FIPSPS-GRQRFNVLGALDAITKDLVSVTNHTYINSHSMCLLLAKLALL
                DPV-IPISVILDNARYQKCQLVTDFAEIVD--IELVYLPSYSRHLNLIERLWRFVRKE
                CLYSKYYADFPAFKGAIQQCIDQCNGEHKAKLTTLLSLKFQSFKK"
                /note="IS630  e-value= 3.1e-22 complete sequence hit
```

```
                     coverage= 100%, between model( 376 aa) positions 1; 376
                     length is 346aa with 33 gaps, 0 stops, absolute frame=
                     Plus3"
     CDS             1016775..1017812
                     /colour="255 0 0"
                     /evidence=predicted
                     /translation="PMIKIDFTAEEIQQLNYERYHHPHPRVQRRMEVLYLKSQGLSHT
                     QICQLCQISRPTLAKTLRQYQQGG---IEGLKTLEYKGQPSLLNAHS-DSVRAYFEQH
                     PP---------RTSAEAQAVIEQLTGIKRSPTQIKAFLKRIGCRYRKVGYVPGKSSLP
                     -EKIEEQEQFRHTRLEPLleeAQRQERLVFFVDAAHFVHRAYLGF-VWCITRI-----
                     ---------FIPSPS-GRQRFNVLGALDAITKDLVSVTNHTYINSHSMCLLLAKLALL
                     DPV-IPISVILDNARYQKCQLVTDFAEIVD--IELVYLPSYSRHLNLIERLWRFVRKE
                     CLYSKYYADFPAFKGAIQQCIDQCNGEHKAKLTTLLSLKFQSFKK"
                     /note="IS630 e-value= 3.5e-21 complete sequence hit
                     coverage= 100%, between model( 376 aa) positions 1; 376
                     length is 346aa with 33 gaps, 0 stops, absolute frame=
                     Plus3"
     CDS             1016835..1016993
                     /colour="255 0 0"
                     /evidence=predicted
                     /translation="HHPHPRVQRRMEVLYLKSQGLSHTQICQLCQISRPTLAKTLRQY
                     QQGGIEGLK"
                     /note="IS481 e-value= 3.7e-09 fragment hit coverage=
                     15.10%, between model( 351 aa) positions 1; 53 length is
                     53aa with 0 gaps, 0 stops, absolute frame= Plus3"
     CDS             1099857..1100243
                     /colour="255 0 0"
                     /evidence=predicted
                     /translation="YADESGMDHRDEYDY-AYGPKGE-----------RVYA-LKTG-
                     RRSGRVNMIAALRAKQL--MAPFTIEGACNRTVFELwLERCLIPvlKPGQKLVI--DN
                     ATFHKGGQIQELVEKAG--CEVWYLPPYSPDLNKIERSWSWIKSRIR"
                     /note="IS630 e-value= 1.1e-13 fragment hit coverage=
                     38.83%, between model( 376 aa) positions 188; 333 length
                     is 129aa with 20 gaps, 0 stops, absolute frame= Plus3"
     CDS             1100496..1101191
                     /colour="255 0 0"
                     /evidence=predicted
                     /translation="FKHLHLGMIsDIKRKSLPAIARAVGLENHQSLHHFLSESPWVAS
                     hLSDkRLDIILKILDGRRLILLID------------------ETGDCKKGKSTDYVKR
                     QYIGNVGKKENGIvAVTAYGLFQGMILPLSFEVYKPR---------ErLKEEDEYLSK
                     PQIAAGMIRQLQGMGFCFELVLADSLYGEAQTNFVNVLEELKLPYILAIRSNHAVWLP
                     AEQAVFAEPWQSFERTFSNGTTETRYrQEIIYGqrhhQKRY"
                     /note="IS4   e-value= 4.7e-14 fragment hit coverage=
                     51.23%, between model( 486 aa) positions 1; 249 length is
                     232aa with 27 gaps, 0 stops, absolute frame= Plus3"
     CDS             1100496..1101623
                     /colour="255 0 0"
                     /evidence=predicted
                     /translation="FKHLHLGMIsDIKRKSLPAIARAVGLENHQSLHHFLSESPWVAS
                     hLSDkRLDIILKILDGRRLILLID------------------ETGDCKKGKSTDYVKR
                     QYIGNVGKKENGIvAVTAYGLFQGMILPLSFEVYKPR---------ErLKEEDEYLSK
                     PQIAAGMIRQLQGMGFCFELVLADSLYGEAQTNFVNVLEELKLPYILAIRSNHAVWLP
                     AEQAVFAEPWQSFERTFSNGT--------------------------------TETRYR
                     QEIIYGQRHHQKRyWLLTTDPQTLPENSTS-----------------------YVMAA
                     APE----IKLDEIGDCYGFRTWIEYGLKQSKD-TLGWADFRMTHYEQIEKWWEIVMSA
                     FLMVSLFADVFNDS------------------------CPVAHQHFAQHPwWDNQNG
                     WKNLLNNVR--LIIQPLIS----WNLLKRWLEVFPSRALKKGF"
                     /note="IS4   e-value= 9.9e-07 complete sequence hit
                     coverage= 100%, between model( 486 aa) positions 1; 486
                     length is 376aa with 117 gaps, 0 stops, absolute frame=
```

```
                     Plus3"
     CDS             1211556..1212239
                     /colour="255 0 0"
                     /evidence=predicted
                     /translation="LHLXYFRRQYQCGGQHIHKNGHRR-GKQNHICVACGR----QFL
                     STYSKRGysdwTKRLCLRMYVNGMGLQGIERVIGVAHTTVIRWVQQV--GVLLPDAYD
                     PDDIPQVGELDELETFVGQKRNKVWIWTVVDHFHPGILGWVVGDHSAETFRPLWQAIV
                     CWQ-CFFWVSDGNPVYPGFIPEG----DQIVSKTYMTRVEGENTRLRHYLARLHRKTL
                     CYSKSVDMLKHSIRLLI-HYLKF"
                     /note="IS1_ORF2    e-value= 4.1e-24 complete sequence hit
                     coverage= 100%, between model( 237 aa) positions 1; 237
                     length is 228aa with 13 gaps, 1 stops, absolute frame=
                     Plus3"
     CDS             1211586..1212233
                     /colour="255 0 0"
                     /evidence=predicted
                     /translation="QCGGQH-IHKNGHRR-GKQNHICVACGRQFL--STYSKRGYSDW
                     TKRLCLRMYVNGMGLQGIERVIGVAHTTVIRWVQQVGVLLPDAYDPDD------IP--
                     -QVGELDELETFVGQKRNKVWIW-TVVDHFHPG--ILGWVVGDHSAETFRPLWqaIVC
                     WQCFFWVSDGNPVYpgFIPEgDQIVSKTYMTRVEGENTRLRHYLARLHRKTLCYSKSV
                     DMLKHSIRLL---IHYL"
                     /note="IS1_ORF1    e-value= 5.5e-39 fragment hit coverage=
                     96.23%, between model( 239 aa) positions 10; 239 length
                     is 216aa with 19 gaps, 0 stops, absolute frame= Plus3"
     CDS             1211586..1212233
                     /colour="255 0 0"
                     /evidence=predicted
                     /translation="---------QCGGQH-IHKNGHRR-GKQNHICVACGRQFL--ST
                     YSKRGYSDWTKRLCLRMYVNGMGLQGIERVIGVAHTTVIRWVQQVGVLLPDAYDPDD-
                     -----IP---QVGELDELETFVGQKRNKVWIW-TVVDHFHPG--ILGWVVGDHSAETF
                     RPLWqaIVCWQCFFWVSDGNPVYpgFIPEgDQIVSKTYMTRVEGENTRLRHYLARLHR
                     KTLCYSKSVDMLKHSIRLL---IHYL"
                     /note="IS1_ORF1    e-value= 5.6e-36 complete sequence hit
                     coverage= 100%, between model( 239 aa) positions 1; 239
                     length is 216aa with 28 gaps, 0 stops, absolute frame=
                     Plus3"
     CDS             1211838..1212239
                     /colour="255 0 0"
                     /evidence=predicted
                     /translation="DPDDIPQVGELDELETFVGQKRNKVWIWTVVDHFHPGILGWVVG
                     DHSAETFRPLWQAIVCWQ-CFFWVSDGNPVYPGFIPEG----DQIVSKTYMTRVEGEN
                     TRLRHYLARLHRKTLCYSKSVDMLKHSIRLLI-HYLKF"
                     /note="IS1_ORF2    e-value= 1.9e-31 fragment hit coverage=
                     59.07%, between model( 237 aa) positions 98; 237 length
                     is 134aa with 6 gaps, 0 stops, absolute frame= Plus3"
     CDS             1474827..1475525
                     /colour="255 0 0"
                     /evidence=predicted
                     /translation="RMAYPSsrSLKCRASSKVVSPXSTIGGSS---------ILAGIS
                     DLFPYCWRLGSLXINCLPYCppGRNRSDELRTLSLTWSEISATR---------VXATG
                     CRCDGCDXNAHXTSPEKTKSVLF----REKKRHTLKCQIIADRNTLEIICLSFGPGRR
                     HDFQIFKVSGIHIHPNTESLQDSGYQGIAAYHANSYVPFKKSQHSELTSLQREYNRAL
                     SQERMGIEHIK---------SQPEDFQNSVGALPXSSSSLHAAVXL"
                     /note="IS5_ISL2    e-value= 5e-10 complete sequence hit
                     coverage= 100%, between model( 260 aa) positions 1; 260
                     length is 233aa with 31 gaps, 7 stops, absolute frame=
                     Plus3"
     CDS             1475154..1475444
                     /colour="255 0 0"
                     /evidence=predicted
```

```
                     /translation="KKRHTLKCQIIADRNTLEIICLSFGPGRRHDFQIFKVSGIHIHP
                     NTESLQDSGYQGIAAYHANSYVPFKKSQHSELTSLQREYNRALSQERMGIEHI"
                     /note="IS5_ISL2    e-value= 4.6e-35 fragment hit coverage=
                     37.31%, between model( 260 aa) positions 128; 224 length
                     is 97aa with 0 gaps, 0 stops, absolute frame= Plus3"
     CDS             1500237..1500374
                     /colour="255 0 0"
                     /evidence=predicted
                     /translation="WQTDGWEGYARQLPDE---VVHEVSKALTQRLERTNGIVRQQTG
                     RWHRR"
                     /note="IS1_ORF2    e-value= 1.8e-11 fragment hit coverage=
                     20.68%, between model( 237 aa) positions 164; 212 length
                     is 46aa with 3 gaps, 0 stops, absolute frame= Plus3"
     CDS             1586793..1587056
                     /colour="255 0 0"
                     /evidence=predicted
                     /translation="LFYFLMQCPLCGHPK-THKHGKTSKGSQRYRCPHCQQTFS-Etf
                     DTLCYRRQiSPETIQTILQAHVEGSSLRGLSRITGVAYNTCVSVVR"
                     /note="IS1_ORF1    e-value= 1.7e-15 fragment hit coverage=
                     36.40%, between model( 239 aa) positions 1; 87 length is
                     88aa with 2 gaps, 0 stops, absolute frame= Plus3"
     CDS             2912997..2913938
                     /colour="255 0 0"
                     /evidence=predicted
                     /translation="TKNRXPMSYQIRNWSEYNAGLKQRGSVTFWLEQS--AIVHWLET
                     TPRQKRGASLTYSDTAISTFETVKCIYGLAGRQTEGFLNSLFELMGIELPVCDHSTVS
                     RRKGQLSISLPVIPKQGAIHVVIDSTGVKVYG------------EGEWKTRQHGVS-K
                     RRTWRKLHLSVDESTGEILTGVVTTNDVQDGEVFEDLLEGIDD-----EIEQVSAD-G
                     AYDQSHCYDALMERNATAAIPPRKNA-------------KIWQHGNC--NApphpRDQ
                     NLRAIRKQGRKRWKKQAHYHRRSLAETTMFRFKTIFGGQVRSRNFDNQATELLLQCAA
                     LN-RMIQIAKPETVWVE"
                     /note="IS5_IS903   e-value= 1.1e-96 complete sequence hit
                     coverage= 100%, between model( 347 aa) positions 1; 347
                     length is 314aa with 37 gaps, 1 stops, absolute frame=
                     Plus3"
     CDS             2912997..2913938
                     /colour="255 0 0"
                     /evidence=predicted
                     /translation="TKNRXPMSYQIRNWSEYNAGLKQRGSVTFWLEQS--AIVHWLET
                     TPRQKRGASLTYSDTAISTFETVKCIYGLAGRQTEGFLNSLFELMGIELPVCDHSTVS
                     RRKGQLSISLPVIPKQGAIHVVIDSTGVKVYG------------EGEWKTRQHGVS-K
                     RRTWRKLHLSVDESTGEILTGVVTTNDVQDGEVFEDLLEGIDD-----EIEQVSAD-G
                     AYDQSHCYDALMERNATAAIPPRKNA-------------KIWQHGNC--NApphpRDQ
                     NLRAIRKQGRKRWKKQAHYHRRSLAETTMFRFKTIFGGQVRSRNFDNQATELLLQCAA
                     LN-RMIQIAKPETVWVE"
                     /note="IS5_IS903   e-value= 2.2e-96 complete sequence hit
                     coverage= 100%, between model( 347 aa) positions 1; 347
                     length is 314aa with 37 gaps, 1 stops, absolute frame=
                     Plus3"
     CDS             2982312..2983067
                     /colour="255 0 0"
                     /evidence=predicted
                     /translation="LQANLQTHAPRMAHLSPKSLXCRATSKVVPPRSTIsgpSILARI
                     SDLFPHCWRLGRFXIN-----GLPYSPAGR---ECSDELRTLP----PTRQETSNTRI
                     XATXCCRDGCDXDTDXTsSKRTKSVLFRKKKRHTLKCQIIANRSTLEIICLNFGKGRR
                     HDFQIFKVSGIHIHPNTESLQDSGYQGITAYHSNSYVPFKKPQHGELTSLQREYNRAL
                     SQERMGIEHINRSLKIFRILSERYRNRRRRYALRCNLIAALYNHEL"
                     /note="IS5_ISL2    e-value= 4.5e-45 complete sequence hit
                     coverage= 100%, between model( 260 aa) positions 1; 260
                     length is 252aa with 12 gaps, 6 stops, absolute frame=
```

```
                     Plus3"
     CDS             2982666..2983067
                     /colour="255 0 0"
                     /evidence=predicted
                     /translation="KKKRHTLKCQIIANRSTLEIICLNFGKGRRHDFQIFKVSGIHIH
                     PNTESLQDSGYQGITAYHSNSYVPFKKPQHGELTSLQREYNRALSQERMGIEHINRSL
                     KIFRILSERYRNRRRRYALRCNLIAALYNHEL"
                     /note="IS5_ISL2    e-value= 5.1e-63 fragment hit coverage=
                     51.54%, between model( 260 aa) positions 127; 260 length
                     is 134aa with 0 gaps, 0 stops, absolute frame= Plus3"
     CDS             3307083..3307769
                     /colour="255 0 0"
                     /evidence=predicted
                     /translation="DELIGENPQPEdiLGESGLLKRLSKRLVERALAGELTHHLQQSS
                     NDQS-GDeNSNGPRNSRNGYSKKTVQSEQG-EMDLSIPRDRCG-EFEPVLVPKGQRRI
                     A-GLDEKIIALYARGMTTRDIRAQLVELYG--ANISEALISDVTNSVMEEVKDWRSRP
                     LDEV--YPIVYLDALYVNIKVNGQVSKRAVYVALAVTVEGNKELLGLWIGAAerEGAK
                     FWLSVLTDLKNRGTQDILM"
                     /note="IS256 e-value= 1.9e-79 fragment hit coverage=
                     56.59%, between model( 410 aa) positions 1; 232 length is
                     229aa with 8 gaps, 0 stops, absolute frame= Plus3"
     CDS             3307083..3309647
                     /colour="255 0 0"
                     /evidence=predicted
                     /translation="DELIGENPQPEdiLGESGLLKRLSKRLVERALAGELTHHLQQSS
                     NDQS-GDeNSNGPRNSRNGYSKKTVQSEQG-EMDLSIPRDRCG-EFEPVLVPKGQRRI
                     A-GLDEKIIALYARGMTTRDIRAQLVELYG--ANISEALISDVTNSVMEEVKDWRSRP
                     LDEV--YPIVYLDALYVNIKVNGQVSKRAVYVALAVTVEGNKELLGLWIGAAerEGAK
                     FWLSVLTDLKNRGTQDilmglfrvlckrsriqthqetvrclerkklpiesmncxmnss
                     ertlnrkifwanqdclndsanaxwneplqvnxlitcsnpptisqvmrilmapeivamv
                     trkrpsslnkerwtyrflvtvaaslslswclkasdasqgsmrksspymlgaxppvife
                     pnwwscmgpislklxlvtspiaswkrsktgvqgrwmrcirlsilmrcmxtsksmvksa
                     selsmslwqxlwratrncwdygxeplnereqnsgfrcsqtlkieelrisxllavmdsk
                     vflkrlsrstlrprcrfvlsilfviafamcrgrkvepxlqtsspftkrplwkslrlrw
                     mhlpingtrrilalaksgydigimlprcfntqwpsvrlstppmplsrstahxerxlrp
                     krfslmkslstnxcfxrxgifrsdgpdqfxigrrrcpillfcsqhdstieytaaytki
                     xtlSLIACCDGLKGFPQAIESVYPQTQVQVCIVHLIRNSLRHVPWKESRAVAADLKPI
                     YQAATLE-ESEAALDAFAH-KWDETYPGISQIWIRHWDNVAPLFQYPMAIRKVIYTTN
                     AIESLNRSLRKVIKTKAVFPNEESVYKLMFLAMRNISKR-WTRPILNWKAALSHFAIL
                     FPTRFNYXIH"
                     /note="IS256 e-value= 4.1e-83 complete sequence hit
                     coverage= 100%, between model( 410 aa) positions 1; 410
                     length is 855aa with 11 gaps, 1 stops, absolute frame=
                     Plus3"
     CDS             3309117..3309647
                     /colour="255 0 0"
                     /evidence=predicted
                     /translation="LIACCDGLKGFPQAIESVYPQTQVQVCIVHLIRNSLRHVPWKES
                     RAVAADLKPIYQAATLE-ESEAALDAFAH-KWDETYPGISQIWIRHWDNVAPLFQYPM
                     AIRKVIYTTNAIESLNRSLRKVIKTKAVFPNEESVYKLMFLAMRNISKR-WTRPILNW
                     KAALSHFAILFPTRFNYXIH"
                     /note="IS256 e-value= 5.1e-77 fragment hit coverage=
                     43.90%, between model( 410 aa) positions 231; 410 length
                     is 177aa with 3 gaps, 1 stops, absolute frame= Plus3"
     CDS             3315393..3316334
                     /colour="255 0 0"
                     /evidence=predicted
                     /translation="TKNRXPMSYQIRNWSEYNAGLKQRGSVTFWLEQS--AIVHWLET
                     TPRQKRGASLTYSDTAISTFETVKCIYGLAGRQTEGFLNSLFELMGIELPVCDHSTVS
                     RRKGQLSISLPVIPKQGAIHVVIDSTGIKVYG------------EGEWKTRQHGVS-K
```

```
                     RRTWRKLHLSVDESTGEILTGVVTTNDVQDGEVFEDLLEGIDD-----EIEQVSAD-G
                     AYDQSHCYDALMERNATAAIPPRKNA-------------KIWQHGNC--NApphpRDQ
                     NLRAIRKQGRKRWKKQAHYHRRSLAETTMFRFKTIFGGQVRSRNFDNQATELLLQCAA
                     LN-RMIQIAKPETVWVE"
                     /note="IS5_IS903   e-value= 9.4e-98 complete sequence hit
                     coverage= 100%, between model( 347 aa) positions 1; 347
                     length is 314aa with 37 gaps, 1 stops, absolute frame=
                     Plus3"
     CDS             3315393..3316334
                     /colour="255 0 0"
                     /evidence=predicted
                     /translation="TKNRXPMSYQIRNWSEYNAGLKQRGSVTFWLEQS--AIVHWLET
                     TPRQKRGASLTYSDTAISTFETVKCIYGLAGRQTEGFLNSLFELMGIELPVCDHSTVS
                     RRKGQLSISLPVIPKQGAIHVVIDSTGIKVYG------------EGEWKTRQHGVS-K
                     RRTWRKLHLSVDESTGEILTGVVTTNDVQDGEVFEDLLEGIDD-----EIEQVSAD-G
                     AYDQSHCYDALMERNATAAIPPRKNA--------------KIWQHGNC--NApphpRDQ
                     NLRAIRKQGRKRWKKQAHYHRRSLAETTMFRFKTIFGGQVRSRNFDNQATELLLQCAA
                     LN-RMIQIAKPETVWVE"
                     /note="IS5_IS903   e-value= 1.7e-97 complete sequence hit
                     coverage= 100%, between model( 347 aa) positions 1; 347
                     length is 314aa with 37 gaps, 1 stops, absolute frame=
                     Plus3"
     CDS             3758064..3758450
                     /colour="255 0 0"
                     /evidence=predicted
                     /translation="YADESGMDHRDEYDY-AYGPKGE-----------RVYA-LKTG-
                     RRSGRVNMIAALRAKQL--MAPFTIEGACNRTVFELwLERCLIPvlKPGQKLVI--DN
                     ATFHKGGQIQELVEKAG--CEVWYLPPYSPDLNKIERSWSWIKSRIR"
                     /note="IS630  e-value= 1.1e-13 fragment hit coverage=
                     38.83%, between model( 376 aa) positions 188; 333 length
                     is 129aa with 20 gaps, 0 stops, absolute frame= Plus3"
     CDS             4200300..4200518
                     /colour="255 0 0"
                     /evidence=predicted
                     /translation="RIRFVYIPKHTSXLNQIECWFSILVRRLIRRGNFTSTDDLQQRI
                     LDFIEYFNHtMAKPFQWQFKGFTPHGXLM"
                     /note="IS630  e-value= 5.4e-08 fragment hit coverage=
                     19.15%, between model( 376 aa) positions 305; 376 length
                     is 73aa with 0 gaps, 2 stops, absolute frame= Plus3"
     CDS             4200348..4200458
                     /colour="255 0 0"
                     /evidence=predicted
                     /translation="IECWFSILVRRLIR--RGNFTSTDDLQQRILDFIEYFNH"
                     /note="IS3_IS150_ORF2    e-value= 4.8e-09 fragment hit
                     coverage= 10.03%, between model( 389 aa) positions 329;
                     367 length is 37aa with 2 gaps, 0 stops, absolute frame=
                     Plus3"
     CDS             4250727..4251422
                     /colour="255 0 0"
                     /evidence=predicted
                     /translation="FKHLHLGMIsDIKRKSLPAIARAVGLENHQSLHHFLSESPWVAS
                     hLSDkRLDIILKILDGRRLILLID------------------ETGDCKKGKSTDYVKR
                     QYIGNVGKKENGIvAVTAYGLFQGMILPLSFEVYKPR---------ErLKEEDEYLSK
                     PQIAAGMIRQLQGMGFCFELVLADSLYGEAQTNFVNVLEELKLPYILAIRSNHAVWLP
                     AEQAVFAEPWQSFERTFSNGTTETRYrQEIIYGqrhhQKRY"
                     /note="IS4   e-value= 4.7e-14 fragment hit coverage=
                     51.23%, between model( 486 aa) positions 1; 249 length is
                     232aa with 27 gaps, 0 stops, absolute frame= Plus3"
     CDS             4250727..4251854
                     /colour="255 0 0"
```

```
                     /evidence=predicted
                     /translation="FKHLHLGMIsDIKRKSLPAIARAVGLENHQSLHHFLSESPWVAS
                     hLSDkRLDIILKILDGRRLILLID------------------ETGDCKKGKSTDYVKR
                     QYIGNVGKKENGIvAVTAYGLFQGMILPLSFEVYKPR---------ErLKEEDEYLSK
                     PQIAAGMIRQLQGMGFCFELVLADSLYGEAQTNFVNVLEELKLPYILAIRSNHAVWLP
                     AEQAVFAEPWQSFERTFSNGT-------------------------------TETRYR
                     QEIIYGQRHHQKRyWLLTTDPQTLPENSTS----------------------YVMAA
                     APE----IKLDEIGDCYGFRTWIEYGLKQSKD-TLGWADFRMTHYEQIEKWWEIVMSA
                     FLMVSLFADVFNDS-------------------------CPVAHQHFAQHPwWDNQNG
                     WKNLLNNVR--LIIQPLIS----WNLLKRWLEVFPSRALKKGF"
                     /note="IS4   e-value= 9.9e-07 complete sequence hit
                     coverage= 100%, between model( 486 aa) positions 1; 486
                     length is 376aa with 117 gaps, 0 stops, absolute frame=
                     Plus3"
     CDS             4281402..4281956
                     /colour="255 0 0"
                     /evidence=predicted
                     /translation="HLSKTTQVNPKNQGLNINKVACSSQSW---------NDCLLMPV
                     SLKFFPLSLXFLLISEYMNSGL------ISLMPKHMKHLR----------LFFTVYPE
                     XspACVKQHDTKQRPHEPRQA------VLLLSLRXQIKITLX----------------
                     ----------------------------RLLYVPLKKPQQGELTPLEXEYNRALSQ
                     ERMGIEHINRSLKIFRILSERYRSRSRRYALRCNLIAAIYNYEL"
                     /note="IS5_ISL2    e-value= 3.9e-10 complete sequence hit
                     coverage= 100%, between model( 260 aa) positions 1; 260
                     length is 185aa with 77 gaps, 5 stops, absolute frame=
                     Plus3"
     CDS             4281750..4281956
                     /colour="255 0 0"
                     /evidence=predicted
                     /translation="YVPLKKPQQGELTPLEXEYNRALSQERMGIEHINRSLKIFRILS
                     ERYRSRSRRYALRCNLIAAIYNYEL"
                     /note="IS5_ISL2    e-value= 3.1e-31 fragment hit coverage=
                     26.54%, between model( 260 aa) positions 192; 260 length
                     is 69aa with 0 gaps, 1 stops, absolute frame= Plus3"
     CDS             5056764..5056982
                     /colour="255 0 0"
                     /evidence=predicted
                     /translation="RIRFVYIPKHTSWLNQIECWFSILVRRLIRRGNFTSKDDLQQRI
                     LEFIEYFNHtMAKPFQWQFKGFQPRLXLM"
                     /note="IS630  e-value= 4e-10 fragment hit coverage=
                     19.15%, between model( 376 aa) positions 305; 376 length
                     is 73aa with 0 gaps, 1 stops, absolute frame= Plus3"
     CDS             5056812..5056922
                     /colour="255 0 0"
                     /evidence=predicted
                     /translation="IECWFSILVRRLIR--RGNFTSKDDLQQRILEFIEYFNH"
                     /note="IS3_IS150_ORF2    e-value= 2e-08 fragment hit
                     coverage= 10.03%, between model( 389 aa) positions 329;
                     367 length is 37aa with 2 gaps, 0 stops, absolute frame=
                     Plus3"
     CDS             5147340..5148377
                     /colour="255 0 0"
                     /evidence=predicted
                     /translation="PMIKIDFTAEEIQQLNYERYHHPHPRVQRRMEVLYLKSQGLSHT
                     QICQLCQISRPTLAKTLRQYQQGG---IEGLKTLEYKGQPSLLNAHS-DSVRAYFEQH
                     PP---------RTSAEAQAVIEQLTGIKRSPTQIKAFLKRIGCRYRKVGYVPGKSSLP
                     -EKIEEQEQFRHTRLEPLleeAQRQERLVFFVDAAHFVHRAYLGF-VWCITRI-----
                     ---------FIPSPS-GRQRFNVLGALDAITKDLVSVTNHTYINSHSMCLLLAKLALL
                     DPV-IPISVILDNARYQKCQLVTDFAEIVD--IELVYLPSYSPHLNLIERLWRFVRKE
                     CLYSKYYADFPAFKGAIQQCIDQCNGEHKAKLTTLLSLKFQSFKK"
```

/note="IS630 e-value= 6.7e-25 complete sequence hit
coverage= 100%, between model(376 aa) positions 1; 376
length is 346aa with 33 gaps, 0 stops, absolute frame=
Plus3"
CDS 5147340..5148377
/colour="255 0 0"
/evidence=predicted
/translation="PMIKIDFTAEEIQQLNYERYHHPHPRVQRRMEVLYLKSQGLSHT
QICQLCQISRPTLAKTLRQYQQGG---IEGLKTLEYKGQPSLLNAHS-DSVRAYFEQH
PP---------RTSAEAQAVIEQLTGIKRSPTQIKAFLKRIGCRYRKVGYVPGKSSLP
-EKIEEQEQFRHTRLEPLleeAQRQERLVFFVDAAHFVHRAYLGF-VWCITRI-----
---------FIPSPS-GRQRFNVLGALDAITKDLVSVTNHTYINSHSMCLLLAKLALL
DPV-IPISVILDNARYQKCQLVTDFAEIVD--IELVYLPSYSPHLNLIERLWRFVRKE
CLYSKYYADFPAFKGAIQQCIDQCNGEHKAKLTTLLSLKFQSFKK"
/note="IS630 e-value= 4.9e-24 complete sequence hit
coverage= 100%, between model(376 aa) positions 1; 376
length is 346aa with 33 gaps, 0 stops, absolute frame=
Plus3"
CDS 5147400..5147558
/colour="255 0 0"
/evidence=predicted
/translation="HHPHPRVQRRMEVLYLKSQGLSHTQICQLCQISRPTLAKTLRQY
QQGGIEGLK"
/note="IS481 e-value= 3.7e-09 fragment hit coverage=
15.10%, between model(351 aa) positions 1; 53 length is
53aa with 0 gaps, 0 stops, absolute frame= Plus3"
CDS 5209563..5210690
/colour="255 0 0"
/evidence=predicted
/translation="PFASIIEHFSDLDDPRAAHRIEYSLEDIIIITLCAVLCGADNWV
EVANYGRSKAQWLKQWIALPNG------VPSHDTFEWVFARLKPQQLQQCFLNWTQAI
YQLSAGE-LIAIDGKTLRGAIAPGEQCSLIHMVSAWASHNRLVLGQRTVDEKSNEITA
IPELLKVLELEG-ALVSIDAMGCQTAIAETIIEGQG-DYVLALKGNQGDLYNDVVQLF
dhaCQTQFQGIEHDSYQTVEKGHGRIEHRTYWTMGQTDYLLGAE-RWAQLKSIGCVES
CRRQPGHPG---TLQRRYYLLSIESDAQR---FADAVRSHWGIENQLHWILDVGFRED
KLRACQGCSAQNLSVIRHIAANLLQQESTAKCGVKAKRLKAGWDDNYLVKILSVAAKG
"
/note="ISAs1 e-value= 2.4e-150 complete sequence hit
coverage= 100%, between model(389 aa) positions 1; 389
length is 376aa with 16 gaps, 0 stops, absolute frame=
Plus3"
CDS 5209563..5210690
/colour="255 0 0"
/evidence=predicted
/translation="PFASIIEHFSDLDDPRAAHRIEYSLEDIIIITLCAVLCGADNWV
EVANYGRSKAQWLKQWIALPNG------VPSHDTFEWVFARLKPQQLQQCFLNWTQAI
YQLSAGE-LIAIDGKTLRGAIAPGEQCSLIHMVSAWASHNRLVLGQRTVDEKSNEITA
IPELLKVLELEG-ALVSIDAMGCQTAIAETIIEGQG-DYVLALKGNQGDLYNDVVQLF
dhaCQTQFQGIEHDSYQTVEKGHGRIEHRTYWTMGQTDYLLGAE-RWAQLKSIGCVES
CRRQPGHPG---TLQRRYYLLSIESDAQR---FADAVRSHWGIENQLHWILDVGFRED
KLRACQGCSAQNLSVIRHIAANLLQQESTAKCGVKAKRLKAGWDDNYLVKILSVAAKG
"
/note="ISAs1 e-value= 1.8e-144 complete sequence hit
coverage= 100%, between model(389 aa) positions 1; 389
length is 376aa with 16 gaps, 0 stops, absolute frame=
Plus3"
CDS 5296908..5297147
/colour="255 0 0"
/evidence=predicted
/translation="RNQQHYIRQRLTAIKLLNEGHSRTQVSEQVGCSYDTLTRWMDKY

```
                     LDGGLQGLVQPI--RHQKPSRLSPEEQQQLKEMVLTQR"
                     /note="IS481 e-value= 1.2e-07 fragment hit coverage=
                     23.36%, between model( 351 aa) positions 1; 82 length is
                     80aa with 2 gaps, 0 stops, absolute frame= Plus3"
     CDS             5296935..5297342
                     /colour="255 0 0"
                     /evidence=predicted
                     /translation="RLTAIKLLNEGHSRTQVSEQVGCSYDTLTRWMDKYLDGG---LQ
                     GLVQPIRHQKPSRLSPEEQQQLKEMVLTQRPTDYGIDRNMWTGAILAVVIEQRFEVQL
                     KDSRIYELLSELGLSYQRAHRDYA--NADLNAQKEWVAA"
                     /note="IS630 e-value= 1.9e-29 fragment hit coverage=
                     37.50%, between model( 376 aa) positions 30; 170 length
                     is 136aa with 5 gaps, 0 stops, absolute frame= Plus3"
     CDS             5342181..5342333
                     /colour="255 0 0"
                     /evidence=predicted
                     /translation="QLCLILDNNPTHKG-KMRSQLAIHLEqmgltqsiQVEFLYLPSY
                     SPKLNLVE"
                     /note="IS630 e-value= 4.3e-06 fragment hit coverage=
                     11.70%, between model( 376 aa) positions 279; 322 length
                     is 51aa with 1 gaps, 0 stops, absolute frame= Plus3"
     CDS             5352645..5353340
                     /colour="255 0 0"
                     /evidence=predicted
                     /translation="FKHLHLGMIsDIKRKSLPAIARAVGLENHQSLHHFLSESPWVAS
                     hLSDkRLDIILKILDGRRLILLID------------------ETGDCKKGKSTDYVKR
                     QYIGNVGKKENGIvAVTAYGLFQGMILPLSFEVYKPR---------ErLKEEDEYLSK
                     PQIAAGMIRQLQGMGFCFELVLADSLYGEAQTNFVNVLEELKLPYILAIRSNHAVWLP
                     AEQAVFAEPWQSFERTFSNGTTETRYrQEIIYGqrhhQKRY"
                     /note="IS4   e-value= 4.7e-14 fragment hit coverage=
                     51.23%, between model( 486 aa) positions 1; 249 length is
                     232aa with 27 gaps, 0 stops, absolute frame= Plus3"
     CDS             5352645..5353772
                     /colour="255 0 0"
                     /evidence=predicted
                     /translation="FKHLHLGMIsDIKRKSLPAIARAVGLENHQSLHHFLSESPWVAS
                     hLSDkRLDIILKILDGRRLILLID------------------ETGDCKKGKSTDYVKR
                     QYIGNVGKKENGIvAVTAYGLFQGMILPLSFEVYKPR---------ErLKEEDEYLSK
                     PQIAAGMIRQLQGMGFCFELVLADSLYGEAQTNFVNVLEELKLPYILAIRSNHAVWLP
                     AEQAVFAEPWQSFERTFSNGT-------------------------------TETRYR
                     QEIIYGQRHHQKRyWLLTTDPQTLPENSTS-----------------------YVMAA
                     APE----IKLDEIGDCYGFRTWIEYGLKQSKD-TLGWADFRMTHYEQIEKWWEIVMSA
                     FLMVSLFADVFNDS-------------------------CPVAHQHFAQHPwWDNQNG
                     WKNLLNNVR--LIIQPLIS----WNLLKRWLEVFPSRALKKGF"
                     /note="IS4   e-value= 9.9e-07 complete sequence hit
                     coverage= 100%, between model( 486 aa) positions 1; 486
                     length is 376aa with 117 gaps, 0 stops, absolute frame=
                     Plus3"
     CDS             5358090..5358287
                     /colour="255 0 0"
                     /evidence=predicted
                     /translation="ASPIELSQQQHHCLTQIVRQTTNPYRLVRRASIILSAASGESNT
                     QISRQWQLDRNQVRYWRQRWLD"
                     /note="IS630 e-value= 4e-07 fragment hit coverage=
                     17.55%, between model( 376 aa) positions 1; 66 length is
                     66aa with 0 gaps, 0 stops, absolute frame= Plus3"
     CDS             5653053..5653250
                     /colour="255 0 0"
                     /evidence=predicted
                     /translation="GKHKQILLVLDGAGWHTC---KNRVVPPG--IHLKILPPYSPEL
```

QPAERLWRLADEPLAN-QCFETLDDLED"
/note="IS630 e-value= 1.4e-09 fragment hit coverage= 19.15%, between model(376 aa) positions 275; 346 length is 66aa with 6 gaps, 0 stops, absolute frame= Plus3"

CDS 5666475..5666576
/colour="255 0 0"
/evidence=predicted
/translation="MTYRKLSHSVWECKYHVVFVPKHRKGRIYGRIRR"
/note="IS200 e-value= 1.1e-12 fragment hit coverage= 22.08%, between model(154 aa) positions 1; 34 length is 34aa with 0 gaps, 0 stops, absolute frame= Plus3"

CDS 5666475..5666933
/colour="255 0 0"
/evidence=predicted
/translation="MTYRKLSHSVWECKYHVVFVPKHRKGRIYGRIRRIXAIFLXAGT
SXRECHRrrsFNARSCSX-----VVEYSAEILRVQCGGLRKRKSAIYIARELGNSRNA
-TGHKFWSRGYFVSTVG-KDEEMICXYIRpgiRSKKSKSXSNXTCFGRSPSPLXGVPI
"
/note="IS200 e-value= 3.1e-10 complete sequence hit coverage= 100%, between model(154 aa) positions 1; 154 length is 153aa with 7 gaps, 8 stops, absolute frame= Plus3"

CDS 5666475..5666576
/colour="255 0 0"
/evidence=predicted
/translation="MTYRKLSHSVWECKYHVVFVPKHRKGRIYGR-IRR"
/note="IS200_IS605 e-value= 1.5e-13 fragment hit coverage= 22.88%, between model(153 aa) positions 1; 35 length is 34aa with 1 gaps, 0 stops, absolute frame= Plus3"

CDS 5666475..5666906
/colour="255 0 0"
/evidence=predicted
/translation="MTYRKLSHSVWECKYHVVFVPKHRKGRIYGR--IRRIXAIFLXA
GTsxRECHRrrsFNARSCSX-----VVEYSAEILRVQCGGLRKRKSAIYIARELGNSR
NA-TGHKFWSRGYFVSTVG-KDEEMICXYIR-----PGIRSKKSKSXSNXTCFGRS"
/note="IS200_IS605 e-value= 5.4e-10 complete sequence hit coverage= 100%, between model(153 aa) positions 1; 153 length is 144aa with 14 gaps, 6 stops, absolute frame= Plus3"

CDS 5666475..5666576
/colour="255 0 0"
/evidence=predicted
/translation="-MTYRKLSHSVWECKYHVVFVPKHRKGRIYGRIRR"
/note="IS605 e-value= 1.1e-11 fragment hit coverage= 23.03%, between model(152 aa) positions 1; 35 length is 34aa with 1 gaps, 0 stops, absolute frame= Plus3"

CDS 5666475..5666906
/colour="255 0 0"
/evidence=predicted
/translation="-MTYRKLSHSVWECKYHVVFVPKHRKGRIYGRIRRIXAIFLXAG
TSXREChrrRSFNARSCSXVVEYSAEI---LRVQC-GGLRKRKSAIYIARELGNSRN-
ATGHKFWSRGYFVSTVGKDEEMICXYIRPGIRSKK-----SKSXSNXTCFGRS"
/note="IS605 e-value= 1.7e-10 complete sequence hit coverage= 100%, between model(152 aa) positions 1; 152 length is 144aa with 11 gaps, 7 stops, absolute frame= Plus3"

CDS 5666769..5666846
/colour="255 0 0"
/evidence=predicted

```
                     /translation="GHKFWSRGYFVSTVG-KDEEMICXYIR"
                     /note="IS200 e-value= 3.8e-10 fragment hit coverage=
                     17.53%, between model( 154 aa) positions 102; 128 length
                     is 26aa with 1 gaps, 1 stops, absolute frame= Plus3"
     CDS             5666769..5666846
                     /colour="255 0 0"
                     /evidence=predicted
                     /translation="GHKFWSRGYFVSTVG-KDEEMICXYIR"
                     /note="IS200_IS605 e-value= 1.3e-09 fragment hit
                     coverage= 17.65%, between model( 153 aa) positions 102;
                     128 length is 26aa with 1 gaps, 1 stops, absolute frame=
                     Plus3"
     CDS             5666769..5666846
                     /colour="255 0 0"
                     /evidence=predicted
                     /translation="GHKFWSRGYFVSTVGKDEEMICXYIR"
                     /note="IS605 e-value= 5.7e-09 fragment hit coverage=
                     17.11%, between model( 152 aa) positions 102; 127 length
                     is 26aa with 0 gaps, 1 stops, absolute frame= Plus3"
     CDS             5908257..5908664
                     /colour="255 0 0"
                     /evidence=predicted
                     /translation="MLLVRPISTESLRLLHRIYHSSRHHQVRQRAHCLILFAQGWPPY
                     TLASLFSVSPKTVYNWLKAWNNRG---FAGLYNHPGRGRKPMFNPDQQQQIYEWT-QA
                     SPIQ---------LNQVLAQIEQQWSVRVSKATVKRVLKQMDMSWHR"
                     /note="IS630 e-value= 8.7e-15 fragment hit coverage=
                     39.63%, between model( 376 aa) positions 1; 149 length is
                     136aa with 13 gaps, 0 stops, absolute frame= Plus3"
     CDS             5908317..5908655
                     /colour="255 0 0"
                     /evidence=predicted
                     /translation="SSRHHQVRQRAHCLILFAQGWPPYTLASLFSVSPKTVYNWLKAW
                     NNRGFAGLYNH---PGRGRKPMfNPDQQQQIYEWTQASPIQLNQVLAQIEQQWSVR--
                     -VSKATVKRVLKQMDMS"
                     /note="IS481 e-value= 3.5e-09 fragment hit coverage=
                     33.62%, between model( 351 aa) positions 1; 118 length is
                     113aa with 6 gaps, 0 stops, absolute frame= Plus3"
     CDS             5916063..5917301
                     /colour="255 0 0"
                     /evidence=predicted
                     /translation="IRIPLNLPDI--RVLELSKTERGDWL------IKIESTLQGTTC
                     HQCGREITDL-HCHDQ-PFRIRHLPLFEVPVYLEIRPKRYRCKYCDDhpTTTQHLEWH
                     EPRSPNTKAYERWLLRILIN-STVSDVARKLGVSEDIVSGIIDRWIAAQVDWSEYADL
                     KVMGMDEISLKRG--HR---DFVVLITIPTTdgVDILAVLADRKQQTVANFLQSIP-I
                     DLRQTIERVCTDMYQGFVSAVREQLPQAKIVIDRFHVTKAYRNCADTVRKREVKRLRR
                     ELS------------KQEYD-----------SIKGAMWPFRKRPENLKES-EQQLLER
                     LFA--YSPQLKQAYNLREKLTQIFEGRYTKHGAKcAIRAWCKQVLKSDiKEFDCFLTT
                     INNWMDEMTNYFLEG-WTSGFVEGFNNRVKVLKRRCYGIFDIERLFQRISLDLNGYQT
                     FAVTXTL"
                     /note="ISL3  e-value= 1.8e-43 complete sequence hit
                     coverage= 100%, between model( 451 aa) positions 1; 451
                     length is 413aa with 44 gaps, 1 stops, absolute frame=
                     Plus3"
     CDS             5916222..5917301
                     /colour="255 0 0"
                     /evidence=predicted
                     /translation="RIRHLPLFEVPVYLEIRPKRYRCKYCDDhpTTTQHLEWHEPRSP
                     NTKAYERWLLRILIN-STVSDVARKLGVSEDIVSGIIDRWIAAQVDWSEYADLKVMGM
                     DEISLKRG--HR---DFVVLITIPTTdgVDILAVLADRKQQTVANFLQSIP-IDLRQT
                     IERVCTDMYQGFVSAVREQLPQAKIVIDRFHVTKAYRNCADTVRKREVKRLRRELS--
```

```
                     ----------KQEYD-----------SIKGAMWPFRKRPENLKES-EQQLLERLFA--
                     YSPQLKQAYNLREKLTQIFEGRYTKHGAKcAIRAWCKQVLKSDiKEFDCFLTTINNWM
                     DEMTNYFLEG-WTSGFVEGFNNRVKVLKRRCYGIFDIERLFQRISLDLNGYQTFAVTX
                     TL"
                     /note="ISL3  e-value= 1e-49 fragment hit coverage= 86.03%,
                     between model( 451 aa) positions 64; 451 length is 360aa
                     with 34 gaps, 1 stops, absolute frame= Plus3"
     CDS             5942076..5942741
                     /colour="255 0 0"
                     /evidence=predicted
                     /translation="-----TGVYKQTF-KRMLHAWHTYHlSHSNAGRPPKLCRPDQLL
                     VALQYWREYRTYFHIAGDWEVSESTVCRIVHQVETALMNSGLFRLPGKKHLIQGFERP
                     DVVVMDVTETPIERPQKGQKAYYSGKKRDIPSNARLLLTAT--LXRLSV--------X
                     ALAQAVGMIFR-------------YSRVQVSIS--IQIPRVCKIADIKGLQPTTPTAM
                     FLS--------RSHNMVNXLPCSESITVHXVRNEWALNTLIAA"
                     /note="IS5_ISL2    e-value= 1.8e-23 complete sequence hit
                     coverage= 100%, between model( 260 aa) positions 1; 260
                     length is 222aa with 39 gaps, 4 stops, absolute frame=
                     Plus3"
     CDS             5942136..5942447
                     /colour="255 0 0"
                     /evidence=predicted
                     /translation="SHSNAGRPPKLCRPDQLLVALQYWREYRTYFHIAGDWEVSESTV
                     CRIVHQVETALMNSGLFRLPGKKHLIQGFERPDVVVMDVTETPIERPQKGQKAYYSGK
                     KR"
                     /note="IS5_ISL2    e-value= 5e-44 fragment hit coverage=
                     40%, between model( 260 aa) positions 26; 129 length is
                     104aa with 0 gaps, 0 stops, absolute frame= Plus3"
     CDS             5992275..5993312
                     /colour="255 0 0"
                     /evidence=predicted
                     /translation="PMIKIDFTAEEIQQLNYERYHHPHPRVQRRMEVLYLKSQGLSHT
                     QICQLCQISRPTLAKTLRQYQQGG---IEGLKTLEYKGQPSLLNAHS-DSVRAYFEQH
                     PP---------RTSAEAQAVIEQLTGIKRSPTQIKAFLKRIGCRYRKVGYVPGKSSLP
                     -EKIEEQEQFRHTRLEPLleeAQRQERLVFFVDAAHFVHRAYLGF-VWCITRI-----
                     ---------FIPSPS-GRQRFNVLGALDAITKDLVSVTNHTYINSHSMCLLLAKLALL
                     DPV-IPISVILDNARYQKCQLVTDFAEIVD--IELVYLPSYSPHLNLIERLWRFVRKE
                     CLYSKYYADFPAFKGAIQQCIDQCNGEHKAKLTTLLSLKFQSFKK"
                     /note="IS630  e-value= 6.7e-25 complete sequence hit
                     coverage= 100%, between model( 376 aa) positions 1; 376
                     length is 346aa with 33 gaps, 0 stops, absolute frame=
                     Plus3"
     CDS             5992275..5993312
                     /colour="255 0 0"
                     /evidence=predicted
                     /translation="PMIKIDFTAEEIQQLNYERYHHPHPRVQRRMEVLYLKSQGLSHT
                     QICQLCQISRPTLAKTLRQYQQGG---IEGLKTLEYKGQPSLLNAHS-DSVRAYFEQH
                     PP---------RTSAEAQAVIEQLTGIKRSPTQIKAFLKRIGCRYRKVGYVPGKSSLP
                     -EKIEEQEQFRHTRLEPLleeAQRQERLVFFVDAAHFVHRAYLGF-VWCITRI-----
                     ---------FIPSPS-GRQRFNVLGALDAITKDLVSVTNHTYINSHSMCLLLAKLALL
                     DPV-IPISVILDNARYQKCQLVTDFAEIVD--IELVYLPSYSPHLNLIERLWRFVRKE
                     CLYSKYYADFPAFKGAIQQCIDQCNGEHKAKLTTLLSLKFQSFKK"
                     /note="IS630  e-value= 4.9e-24 complete sequence hit
                     coverage= 100%, between model( 376 aa) positions 1; 376
                     length is 346aa with 33 gaps, 0 stops, absolute frame=
                     Plus3"
     CDS             5992335..5992493
                     /colour="255 0 0"
                     /evidence=predicted
                     /translation="HHPHPRVQRRMEVLYLKSQGLSHTQICQLCQISRPTLAKTLRQY
```

QQGGIEGLK"
/note="IS481 e-value= 3.7e-09 fragment hit coverage= 15.10%, between model(351 aa) positions 1; 53 length is 53aa with 0 gaps, 0 stops, absolute frame= Plus3"
CDS 6109407..6109544
/colour="255 0 0"
/evidence=predicted
/translation="WQTDGWEGYARQLPDE---VVHEVSKALTQRLERTNGIVRQQTG
RWHRR"
/note="IS1_ORF2 e-value= 1.8e-11 fragment hit coverage= 20.68%, between model(237 aa) positions 164; 212 length is 46aa with 3 gaps, 0 stops, absolute frame= Plus3"
CDS 6263616..6264854
/colour="255 0 0"
/evidence=predicted
/translation="IRIPLNLPDI--RVLELSKTERGDWL------IKIESTLQGTTC
HQCGREITDL-HCHDQ-PFRIRHLPLFEVPVYLEIRPKRYRCKYCDDhpTTTQHLEWH
EPRSPNTKAYERWLLRILIN-STVSDVARKLGVSEDIVSGIIDRWIAAQVDWSEYADL
KVMGMDEISLKRG--HR---DFVVLITIPTTdgVDILAVLADRKQQTVANFLQSIP-I
DLRQTIERVCTDMYQGFVSAVREQLPQAKIVIDRFHVTKAYRNCADTVRKREVKRLRR
ELS------------KQEYD-----------SIKGAMWPFRKRPENLKES-EQQLLER
LFA--YSPQLKQAYNLREKLTQIFEGRYTKHGAKcAIRAWCKQVLKSDiKEFDCFLTT
INNWMDEMTNYFLEG-WTSGFVEGFNNRVKVLKRRCYGIFDIERLFQRISLDLNGYQT
FAVTXTL"
/note="ISL3 e-value= 1.8e-43 complete sequence hit coverage= 100%, between model(451 aa) positions 1; 451 length is 413aa with 44 gaps, 1 stops, absolute frame= Plus3"
CDS 6263775..6264854
/colour="255 0 0"
/evidence=predicted
/translation="RIRHLPLFEVPVYLEIRPKRYRCKYCDDhpTTTQHLEWHEPRSP
NTKAYERWLLRILIN-STVSDVARKLGVSEDIVSGIIDRWIAAQVDWSEYADLKVMGM
DEISLKRG--HR---DFVVLITIPTTdgVDILAVLADRKQQTVANFLQSIP-IDLRQT
IERVCTDMYQGFVSAVREQLPQAKIVIDRFHVTKAYRNCADTVRKREVKRLRRELS--
----------KQEYD-----------SIKGAMWPFRKRPENLKES-EQQLLERLFA--
YSPQLKQAYNLREKLTQIFEGRYTKHGAKcAIRAWCKQVLKSDiKEFDCFLTTINNWM
DEMTNYFLEG-WTSGFVEGFNNRVKVLKRRCYGIFDIERLFQRISLDLNGYQTFAVTX
TL"
/note="ISL3 e-value= 1e-49 fragment hit coverage= 86.03%, between model(451 aa) positions 64; 451 length is 360aa with 34 gaps, 1 stops, absolute frame= Plus3"
CDS 6300402..6300794
/colour="255 0 0"
/evidence=predicted
/translation="VDTHGQSEVGFAFCYLLNFQLMPRFKGINKQKLYLPMsGQKKKY
VYLKAIVKRPMRWDLVREQYEPMIQFTTAMKQGTAEPEAILSRFTRNNIKHPTYLALA
ELGRAIKTIFLCQYLHDEALRQEINEGLN"
/note="Tn3 e-value= 7.4e-17 fragment hit coverage= 12.95%, between model(1004 aa) positions 764; 893 length is 131aa with 0 gaps, 0 stops, absolute frame= Plus3"
CDS 6306396..6307874
/colour="255 0 0"
/evidence=predicted
/translation="DSYHTRVYMNGRDLGLKQAEAAYIAEISTRTGQRIEAGTHQPNR
-GRLQD-------QRTVPDPLADVWE---DELEPMLRRDPRLKPMTLYEYLQD-KYPG
QYPQVlrtlQRRVRTWKALHGPS-----PEVMFELRHEPGVQGFSDFTELKGITITIA
GQPFEHLIYHYRLGYSGWRYAQIIQGgESFVALSEGLQNAFAACGGVPQQHRTDSLSA
AYRNMGGRRS-KNLTRLYDELCDHYRLEPTRNNKG--IAHENGSIESPHGHLKNRIKQ
AIYLRGSAD-----FTSVAEYQALIDAQVAKLNQQCQTK------YEQEKDHLQPLPK

```
                     YRTPDYEVLTA----KVSKRSTIDVRCILYTVPSRLIGRQLELHLYHDRIVGYLERHP
                     VVELPRKRVSGkGKRRDRCINYRHVIGSMRLKPRAFIYCTWQSDLLPNPEYRQIWEQL
                     KAQFDLEQAAKIIVEALYIAAVQDKEQAVAVYLQQQLQASSLTLNRLKKQFEPPQMKQ
                     VPDLSIeQHS----LELYDKLLPS"
                     /note="IS21_ORF1   e-value= 1.3e-21 complete sequence hit
                     coverage= 100%, between model( 525 aa) positions 1; 525
                     length is 493aa with 39 gaps, 0 stops, absolute frame=
                     Plus3"
     CDS             6306666..6307496
                     /colour="255 0 0"
                     /evidence=predicted
                     /translation="QYPQVLRTLQRRVRTWKALHGPS-----PEVMFELRHEPGVQGF
                     SDFTELKGITITIAGQPFEHLIYHYRLGYSGWRYAQIIQGgESFVALSEGLQNAFAAC
                     GGVPQQHRTDSLSAAYRNMGGRRS-KNLTRLYDELCDHYRLEPTRNNKG--IAHENGS
                     IESPHGHLKNRIKQAIYLRGSAD-----FTSVAEYQALIDAQVAKLNQQCQTK-----
                     -YEQEKDHLQPLPKYRTPDYEVLTA----KVSKRSTIDVRCILYTVPSRLIGRQLELH
                     LYHDRIVGY-----LERHPVVELPRKRVS"
                     /note="IS21_ORF1   e-value= 1e-29 fragment hit coverage=
                     57.90%, between model( 525 aa) positions 99; 402 length
                     is 277aa with 28 gaps, 0 stops, absolute frame= Plus3"
     CDS             complement(6395489..6394549)
                     /colour="255 0 0"
                     /evidence=predicted
                     /translation="TKNRXPMSYQIRNWSEYNAGLKQRGSVTFWLEQS--AIVHWLET
                     TPRQKRGASLTYSDTAISTFETVKCIYGLAGRQTEGFLNSLFELMGIELPVCDHSTVS
                     RRKGQLSISLPVIPKQGAIHVVIDSTGVKVYG------------EGEWKTRQHGVS-K
                     RRTWRKLHLSVDESTGEILTGVVTTNDVQDGEVFEDLLEGIDD-----EIEQVSAD-G
                     AYDQSHCYDALMERNATAAIPPRKNA-------------KIWQHGNC--NApphpRDQ
                     NLRAIRKQGRKRWKKQAHYHRRSLAETTMFRFKTIFGGQVRSRNFDNQATELLLQCAA
                     LN-RMIQIAKPETVWVE"
                     /note="IS5_IS903   e-value= 1.1e-96 complete sequence hit
                     coverage= 100%, between model( 347 aa) positions 1; 347
                     length is 314aa with 37 gaps, 1 stops, absolute frame=
                     Minus1"
     CDS             complement(6395489..6394549)
                     /colour="255 0 0"
                     /evidence=predicted
                     /translation="TKNRXPMSYQIRNWSEYNAGLKQRGSVTFWLEQS--AIVHWLET
                     TPRQKRGASLTYSDTAISTFETVKCIYGLAGRQTEGFLNSLFELMGIELPVCDHSTVS
                     RRKGQLSISLPVIPKQGAIHVVIDSTGVKVYG------------EGEWKTRQHGVS-K
                     RRTWRKLHLSVDESTGEILTGVVTTNDVQDGEVFEDLLEGIDD-----EIEQVSAD-G
                     AYDQSHCYDALMERNATAAIPPRKNA-------------KIWQHGNC--NApphpRDQ
                     NLRAIRKQGRKRWKKQAHYHRRSLAETTMFRFKTIFGGQVRSRNFDNQATELLLQCAA
                     LN-RMIQIAKPETVWVE"
                     /note="IS5_IS903   e-value= 2.2e-96 complete sequence hit
                     coverage= 100%, between model( 347 aa) positions 1; 347
                     length is 314aa with 37 gaps, 1 stops, absolute frame=
                     Minus1"
     CDS             complement(6348974..6348694)
                     /colour="255 0 0"
                     /evidence=predicted
                     /translation="SISFRaMQCIHCQSEN-VVKNGTKT-LKtaqvvQYFLCKDCGRR
                     FN-ERSGTPMARLRtpvETISMAINARTEGLGIRAAGRVLGKSPTSILLWEKR"
                     /note="IS1_ORF1    e-value= 9.8e-10 fragment hit coverage=
                     36.82%, between model( 239 aa) positions 1; 88 length is
                     94aa with 3 gaps, 0 stops, absolute frame= Minus1"
     CDS             complement(6343880..6343381)
                     /colour="255 0 0"
                     /evidence=predicted
                     /translation="QVTLIEAAVETTQQRFGWRVYVTNAPLEELSFEEAVLTVRDAWI
```

```
                     QESGFSRLKGNPLGASPLFVQRDDHAKGLMHLLSLGLRILTLIEFVVQRRLKQ-----
                     --------------HKEkLFGLFPGNPKRATTRPTTERILRAFKDISLTILGVKDeey
                     ghVSPLTSLQQRILELLGLAPDIYSS"
                     /note="IS4   e-value= 3e-08 fragment hit coverage= 37.04%,
                     between model( 486 aa) positions 307; 486 length is 167aa
                     with 19 gaps, 0 stops, absolute frame= Minus1"
     CDS             complement(6314132..6313894)
                     /colour="255 0 0"
                     /evidence=predicted
                     /translation="TLAKLDHFELFILDEIGYDIKTDDETSIQSELIVHRYERRSFKI
                     TANHPFSTRXCIFC-DSSMTVAAIDRFVYHAFIIEIK"
                     /note="IS21_ORF2   e-value= 1.3e-09 fragment hit coverage=
                     31.40%, between model( 258 aa) positions 157; 237 length
                     is 80aa with 1 gaps, 1 stops, absolute frame= Minus1"
     CDS             complement(6234089..6232612)
                     /colour="255 0 0"
                     /evidence=predicted
                     /translation="DSYHTRVYMNGRDLGLKQAEAAYIAEISTRTGQRIEAGTHQPNR
                     -GRLQD-------QRTVPDPLADVWE---DELEPMLRRDPRLKPMTLYEYLQD-KYPG
                     QYPQVlrtlQRRVRTWKALHGPS-----PEVMFELRHEPGVQGFSDFTELKGITITIA
                     GKPFEHLIYHYRLGYSGWRYAQIIQGgESFVALSEGLQNAFAACGGVPTQHRTDSLSA
                     AYRNMGGRRS-KNLTRLYDELCDHYRLEPTRNNKG--VAHENGSIESPHGHLKNRIKQ
                     AIYLRGSAD-----FTSVGEYQALIDAQVA---KLNQQC---QAKYEQEKEHLQPLPK
                     YRTPDYEVLTA----KVSKRSTIDVRCILYTVPSRLIGRQLELHLYHDRIVGYLERHP
                     VVELPRKRVSGkGKRRDRCINYRHVIGSMRLKPRAFIYCTWQSDLLPNPEYRQIWEQL
                     KAQFDLEQAAKIIVEALYIAAVQDKEQAVAVYLQQQLRSSSLTLNRLKKQFEPPQMKQ
                     VPDLSIeQHS----LELYDKLLPS"
                     /note="IS21_ORF1   e-value= 3.3e-24 complete sequence hit
                     coverage= 100%, between model( 525 aa) positions 1; 525
                     length is 493aa with 39 gaps, 0 stops, absolute frame=
                     Minus1"
     CDS             complement(6233819..6232990)
                     /colour="255 0 0"
                     /evidence=predicted
                     /translation="QYPQVLRTLQRRVRTWKALHGPS-----PEVMFELRHEPGVQGF
                     SDFTELKGITITIAGKPFEHLIYHYRLGYSGWRYAQIIQGgESFVALSEGLQNAFAAC
                     GGVPTQHRTDSLSAAYRNMGGRRS-KNLTRLYDELCDHYRLEPTRNNKG--VAHENGS
                     IESPHGHLKNRIKQAIYLRGSAD-----FTSVGEYQALIDAQVA---KLNQQC---QA
                     KYEQEKEHLQPLPKYRTPDYEVLTA----KVSKRSTIDVRCILYTVPSRLIGRQLELH
                     LYHDRIVGY-----LERHPVVELPRKRVS"
                     /note="IS21_ORF1   e-value= 4.2e-32 fragment hit coverage=
                     57.90%, between model( 525 aa) positions 99; 402 length
                     is 277aa with 28 gaps, 0 stops, absolute frame= Minus1"
     CDS             complement(6059630..6059137)
                     /colour="255 0 0"
                     /evidence=predicted
                     /translation="LEKTGIESKKKTYGYR--ERD--ETQR---QAFIERLQTKHP--
                     HQIVYVDEAGIDNRADYPY-GYCPVG-----------QRFYD-LKSG-KRTERVSFIA
                     ALKEGQL--FSPMTFEGSCNRLLFEAWLQQSLI-SQLQLgdVIVIDNASFHHGQRIEE
                     IVAEAG--CEIWYLPSYSPDLNKIERWWFVLKN"
                     /note="IS630  e-value= 3e-16 fragment hit coverage=
                     50.80%, between model( 376 aa) positions 140; 330 length
                     is 165aa with 28 gaps, 0 stops, absolute frame= Minus1"
     CDS             complement(6014810..6014614)
                     /colour="255 0 0"
                     /evidence=predicted
                     /translation="ASPIELSQQQHHCLTQIVRQTTNPYRLVRRASIILSAASGESNT
                     QISRQWQLDRNQVRYWRQRWLD"
                     /note="IS630  e-value= 4e-07 fragment hit coverage=
                     17.55%, between model( 376 aa) positions 1; 66 length is
```

66aa with 0 gaps, 0 stops, absolute frame= Minus1"
CDS complement(5728283..5728174)
/colour="255 0 0"
/evidence=predicted
/translation="VMKVMI-CPHCQSDR-LSKNGKRR-NQQCYVCKDCRKQFV"
/note="IS1_ORF1 e-value= 4.1e-06 fragment hit coverage= 16.74%, between model(239 aa) positions 1; 40 length is 37aa with 3 gaps, 0 stops, absolute frame= Minus1"
CDS complement(5719823..5719186)
/colour="255 0 0"
/evidence=predicted
/translation="MASKTTNVkPVVVSLWKLLSKSELIHPLKAXSISYCLKRYLWQV
L-----PGS------VMFLKAGYKSTXTVsMKQCLVLXTYHQKKGR------------
---LTLQCDEMWSFVNDKSNKQWIWLALDVITREIVGVYVGARSKQGARQLWNSLPGI
YRQCAVaYTDFWDAYGCVFPKQ-RHQAVGKETGQTCYIERFNCTMRQRVSRLVRKTLS
FSKKLENHIGAIWMFVHHYNAS"
/note="IS1_ORF2 e-value= 4.6e-13 complete sequence hit coverage= 100%, between model(237 aa) positions 1; 237 length is 213aa with 27 gaps, 3 stops, absolute frame= Minus1"
CDS complement(5719571..5719195)
/colour="255 0 0"
/evidence=predicted
/translation="DEMWSFVNDKSNKQWIWLALDVITREIVGVYVGA----RSKQGA
RQLwNSLPGIYRQCAVAYTDFWDAYgCVFPKQRHQAVGKEtgQTCYIERFNCTMRQRV
SRLVRKTLSFSKKLENHIGAIWMF---VHHY"
/note="IS1_ORF1 e-value= 4.6e-14 fragment hit coverage= 53.97%, between model(239 aa) positions 111; 239 length is 126aa with 7 gaps, 0 stops, absolute frame= Minus1"
CDS complement(5719571..5719186)
/colour="255 0 0"
/evidence=predicted
/translation="DEMWSFVNDKSNKQWIWLALDVITREIVGVYVGARSKQGARQLW
NSLPGIYRQCAVaYTDFWDAYGCVFPKQ-RHQAVGKETGQTCYIERFNCTMRQRVSRL
VRKTLSFSKKLENHIGAIWMFVHHYNAS"
/note="IS1_ORF2 e-value= 6.2e-32 fragment hit coverage= 54.43%, between model(237 aa) positions 109; 237 length is 129aa with 1 gaps, 0 stops, absolute frame= Minus1"
CDS complement(5718014..5717818)
/colour="255 0 0"
/evidence=predicted
/translation="ASPIELSQQQHHCLTQIVRQTTNPYRLVRRASIILSAASGESNT
QISRQWQLDRNQVRYWRQRWLD"
/note="IS630 e-value= 4e-07 fragment hit coverage= 17.55%, between model(376 aa) positions 1; 66 length is 66aa with 0 gaps, 0 stops, absolute frame= Minus1"
CDS complement(5509199..5509063)
/colour="255 0 0"
/evidence=predicted
/translation="WQTDGWEGYARQLPDE---VVHEVSKALTQRLERTNGIVRQQTG
RWHRR"
/note="IS1_ORF2 e-value= 1.8e-11 fragment hit coverage= 20.68%, between model(237 aa) positions 164; 212 length is 46aa with 3 gaps, 0 stops, absolute frame= Minus1"
CDS complement(5354885..5354170)
/colour="255 0 0"
/evidence=predicted
/translation="YLSPES-LXCRASSKVVSMXSTVGSPS---------ILARISDL
FPHCWRLGSLXINCLPYCppGRDRSDELRTLSLTRSEISATR---------VRATGCR
CDGCDXNAHXTSPDKTKSVLF----RKKKRHTFKCQIIANRNTLEIICLNVGPGRRHD

FQISKVSGIHIHPDTESLQDSGYQGIAAYHANSYVPLKKPQHGELTSLQREYNRALSQ
ERMGIEHINRSLKIFRILSERYRNRRRRYALRCNLIAAIYNYEL"
/note="IS5_ISL2 e-value= 8.9e-38 complete sequence hit
coverage= 100%, between model(260 aa) positions 1; 260
length is 239aa with 23 gaps, 5 stops, absolute frame=
Minus1"

CDS complement(5354570..5354170)
/colour="255 0 0"
/evidence=predicted
/translation="KKKRHTFKCQIIANRNTLEIICLNVGPGRRHDFQISKVSGIHIH
PDTESLQDSGYQGIAAYHANSYVPLKKPQHGELTSLQREYNRALSQERMGIEHINRSL
KIFRILSERYRNRRRRYALRCNLIAAIYNYEL"
/note="IS5_ISL2 e-value= 4.7e-61 fragment hit coverage=
51.54%, between model(260 aa) positions 127; 260 length
is 134aa with 0 gaps, 0 stops, absolute frame= Minus1"

CDS complement(5242232..5241538)
/colour="255 0 0"
/evidence=predicted
/translation="FKHLHLGMIsDIKRKSLPAIARAVGLENHQSLHHFLSESPWVAS
hLSDkRLDIILKILDGRRLILLID------------------ETGDCKKGKSTDYVKR
QYIGNVGKKENGIvAVTAYGLFQGMILPLSFEVYKPR---------ErLKEEDEYLSK
PQIAAGMIRQLQGMGFCFELVLADSLYGEAQTNFVNVLEELKLPYILAIRSNHAVWLP
AEQAVFAEPWQSFERTFSNGTTETRYrQEIIYGqrhhQKRY"
/note="IS4 e-value= 4.7e-14 fragment hit coverage=
51.23%, between model(486 aa) positions 1; 249 length is
232aa with 27 gaps, 0 stops, absolute frame= Minus1"

CDS complement(5242232..5241106)
/colour="255 0 0"
/evidence=predicted
/translation="FKHLHLGMIsDIKRKSLPAIARAVGLENHQSLHHFLSESPWVAS
hLSDkRLDIILKILDGRRLILLID------------------ETGDCKKGKSTDYVKR
QYIGNVGKKENGIvAVTAYGLFQGMILPLSFEVYKPR---------ErLKEEDEYLSK
PQIAAGMIRQLQGMGFCFELVLADSLYGEAQTNFVNVLEELKLPYILAIRSNHAVWLP
AEQAVFAEPWQSFERTFSNGT--------------------------------TETRYR
QEIIYGQRHHQKRyWLLTTDPQTLPENSTS-----------------------YVMAA
APE----IKLDEIGDCYGFRTWIEYGLKQSKD-TLGWADFRMTHYEQIEKWWEIVMSA
FLMVSLFADVFNDS-------------------------CPVAHQHFAQHPwWDNQNG
WKNLLNNVR--LIIQPLIS----WNLLKRWLEVFPSRALKKGF"
/note="IS4 e-value= 9.9e-07 complete sequence hit
coverage= 100%, between model(486 aa) positions 1; 486
length is 376aa with 117 gaps, 0 stops, absolute frame=
Minus1"

CDS complement(5175581..5174845)
/colour="255 0 0"
/evidence=predicted
/translation="-----TGVYKRTFkrMLHAWHTYHLSRSN--AGRPPKLCQCDQL
LVTLQYWREYRTYFHIAGDWEVSESTVCRIVHQVETALMNSGLFRLPGQKSLLQGFER
PDVVVMDVTETPIERPQTRQKAYYSGKKRDIPSNARLSLTATLXrlsaltLVQVVG--
-------MIFRSSRVQVSISIQIPRVCKIADIKGLQ-----PIMP-TAMFLSRSHNTV
NXLPCSESITVLXVRNEWALNTLIAAXRFSEFCRSAIAIVVVATRCGVTX"
/note="IS5_ISL2 e-value= 3.2e-21 complete sequence hit
coverage= 100%, between model(260 aa) positions 1; 260
length is 246aa with 22 gaps, 5 stops, absolute frame=
Minus1"

CDS complement(5175521..5175211)
/colour="255 0 0"
/evidence=predicted
/translation="SRSNAGRPPKLCQCDQLLVTLQYWREYRTYFHIAGDWEVSESTV
CRIVHQVETALMNSGLFRLPGQKSLLQGFERPDVVVMDVTETPIERPQTRQKAYYSGK
KR"

```
                     /note="IS5_ISL2    e-value= 7.6e-44 fragment hit coverage=
                     40%, between model( 260 aa) positions 26; 129 length is
                     104aa with 0 gaps, 0 stops, absolute frame= Minus1"
     CDS             complement(5069105..5069026)
                     /colour="255 0 0"
                     /evidence=predicted
                     /translation="TFSCLNRYRRLGKDYELYPETSEAMIY"
                     /note="IS5_IS1031  e-value= 5.8e-07 fragment hit coverage=
                     9.15%, between model( 295 aa) positions 246; 272 length
                     is 27aa with 0 gaps, 0 stops, absolute frame= Minus1"
     CDS             complement(4933280..4932616)
                     /colour="255 0 0"
                     /evidence=predicted
                     /translation="FKYLHLGMMsDIKHKSLPAIARICGLANEQGLLHFLTESPWRPS
                     aLEQaRLNLILQVLSGRPLTLIID------------------ETEDRKKGKQTDYVQR
                     QYLGNLGKVDNGIvAVTAYGVVEHMTLPLMFRVYKPKsRLQSGD---------VYHSK
                     PEIAVSMIDELLAHGCQFDLVLADSLYGESGSTFVSHLQALQLPYVVAIRSNHGLWLP
                     KEQRVRCNRWRAFEHVFSDGS---------------CETRYIREVI"
                     /note="IS4   e-value= 4.5e-12 fragment hit coverage=
                     53.29%, between model( 486 aa) positions 1; 259 length is
                     222aa with 42 gaps, 0 stops, absolute frame= Minus1"
     CDS             complement(4933280..4932184)
                     /colour="255 0 0"
                     /evidence=predicted
                     /translation="FKYLHLGMMsDIKHKSLPAIARICGLANEQGLLHFLTESPWRPS
                     aLEQaRLNLILQVLSGRPLTLIID------------------ETEDRKKGKQTDYVQR
                     QYLGNLGKVDNGIvAVTAYGVVEHMTLPLMFRVYKPKsRLQSGD---------VYHSK
                     PEIAVSMIDELLAHGCQFDLVLADSLYGESGSTFVSHLQALQLPYVVAIRSNHGLWLP
                     KEQRVRCNRWRAFEHVFSDGS-----------------------------CETRYIREV
                     IYGQR----LAQQfWDITTDPQNLPKASTW------------------------YVMS
                     EIE---GVKYHQIGNFYGLRNWVEYGLKQSKN-ELGWADFRVTDYDQIERWWQVVMSA
                     YLMVSLHSDRFEL-----------------------------------PTGLSMKQQVP
                     PVIETFSQ-----HRYWQTGKGWKHLLNNLRLVIIQSLILFD"
                     /note="IS4   e-value= 1.2e-06 complete sequence hit
                     coverage= 100%, between model( 486 aa) positions 1; 486
                     length is 366aa with 126 gaps, 0 stops, absolute frame=
                     Minus1"
     CDS             complement(4908698..4908313)
                     /colour="255 0 0"
                     /evidence=predicted
                     /translation="YADESGMDHRDEYDY-AYGPKGE-----------RVYA-LKTG-
                     RRSGRVNMIAALRAKQL--MAPFTIEGACNRTVFELwLERCLIPvlKPGQKLVI--DN
                     ATFHKGGQIQELVEKAG--CEVWYLPPYSPDLNKIERSWSWIKSRIR"
                     /note="IS630 e-value= 1.1e-13 fragment hit coverage=
                     38.83%, between model( 376 aa) positions 188; 333 length
                     is 129aa with 20 gaps, 0 stops, absolute frame= Minus1"
     CDS             complement(4716020..4715806)
                     /colour="255 0 0"
                     /evidence=predicted
                     /translation="REIHEAIGVSIRTIERVRMRFVEEG---LEAAINQRsGAGRKRK
                     IQGEQEAHLIALRCSEPPVG----HARWTLRLLAD"
                     /note="IS630 e-value= 9.9e-08 fragment hit coverage=
                     20.74%, between model( 376 aa) positions 44; 121 length
                     is 72aa with 7 gaps, 0 stops, absolute frame= Minus1"
     CDS             complement(4612568..4612411)
                     /colour="255 0 0"
                     /evidence=predicted
                     /translation="HHPHPRVQRRMEVLYLKSQGLPHAQICQLCQISRPTLAKTLRLY
                     QQGGIEGLK"
                     /note="IS481 e-value= 1.1e-08 fragment hit coverage=
```

15.10%, between model(351 aa) positions 1; 53 length is 53aa with 0 gaps, 0 stops, absolute frame= Minus1"

CDS complement(4319990..4319854)
/colour="255 0 0"
/evidence=predicted
/translation="WQTDGWEGYARQLPDE---VVHEVSKALTQRLERTNGIVRQQTG
RWHRR"
/note="IS1_ORF2 e-value= 1.8e-11 fragment hit coverage= 20.68%, between model(237 aa) positions 164; 212 length is 46aa with 3 gaps, 0 stops, absolute frame= Minus1"

CDS complement(4252406..4251982)
/colour="255 0 0"
/evidence=predicted
/translation="GRQRFNVLGALNAVTKEvTSITNHTYINSHSMCLLLAKLALLDP
VI--PISVILDNARYQKCQLVTDFAEIVD--IELVYLPSYSPHLNLIERLWRFVRKEC
LYSKYYSDFHSFKGAIQQCIDQCNTEHKAKLTSLLSLKFQSFQK"
/note="IS630 e-value= 6.2e-18 fragment hit coverage= 38.56%, between model(376 aa) positions 232; 376 length is 142aa with 4 gaps, 0 stops, absolute frame= Minus1"

CDS complement(4222007..4221850)
/colour="255 0 0"
/evidence=predicted
/translation="HHPHPRVQRRMEVLYLKSQGLPHAQICQLCQISRPTLAKTLRLY
QQGGIEGLK"
/note="IS481 e-value= 1.1e-08 fragment hit coverage= 15.10%, between model(351 aa) positions 1; 53 length is 53aa with 0 gaps, 0 stops, absolute frame= Minus1"

CDS complement(4221497..4221091)
/colour="255 0 0"
/evidence=predicted
/translation="MLLVRPISTESLRLLHRIYHSSRHHQVRQRAHCLILFAQGWPPY
TLASLFSVSPKTVYNWLKAWNNRG---FAGLYNHPGRGRKPMFNPDQQQQIYEWT-QA
SPIQ---------LNQVLAQIEQQWSVRVSKATVKRVLKQMDMSWHR"
/note="IS630 e-value= 8.7e-15 fragment hit coverage= 39.63%, between model(376 aa) positions 1; 149 length is 136aa with 13 gaps, 0 stops, absolute frame= Minus1"

CDS complement(4221437..4221100)
/colour="255 0 0"
/evidence=predicted
/translation="SSRHHQVRQRAHCLILFAQGWPPYTLASLFSVSPKTVYNWLKAW
NNRGFAGLYNH---PGRGRKPMfNPDQQQQIYEWTQASPIQLNQVLAQIEQQWSVR--
-VSKATVKRVLKQMDMS"
/note="IS481 e-value= 3.5e-09 fragment hit coverage= 33.62%, between model(351 aa) positions 1; 118 length is 113aa with 6 gaps, 0 stops, absolute frame= Minus1"

CDS complement(4218878..4218454)
/colour="255 0 0"
/evidence=predicted
/translation="GRQRFNVLGALNAVTKEvTSITNHTYINSHSMCLLLAKLALLDP
VI--PISVILDNARYQKCQLVTDFAEIVD--IELVYLPSYSPHLNLIERLWRFVRKEC
LYSKYYSDFHSFKGAIQQCIDQCNTEHKAKLTSLLSLKFQSFQK"
/note="IS630 e-value= 6.2e-18 fragment hit coverage= 38.56%, between model(376 aa) positions 232; 376 length is 142aa with 4 gaps, 0 stops, absolute frame= Minus1"

CDS complement(4135019..4134772)
/colour="255 0 0"
/evidence=predicted
/translation="MQCPLCGHPT-THXHGKTSKGSQRYRCLHCXRTFSEtfDTLYYH
RQISPQTLQTILQSHAEGSSLRGLARITGVAYNTCVSVVR"
/note="IS1_ORF1 e-value= 2.5e-12 fragment hit coverage=

```
                     34.31%, between model( 239 aa) positions 6; 87 length is
                     83aa with 1 gaps, 2 stops, absolute frame= Minus1"
     CDS             complement(4135019..4134334)
                     /colour="255 0 0"
                     /evidence=predicted
                     /translation="-----MQCPLCGHPT-THXHGKTSKGSQRYRCLHCXRTFSEtfD
                     TLYYHRQISPQTLQTILQSHAEGSSLRGLARITGVAYNTCVSVVRSA--SHKAQMIHN
                     Q-------EGQAVSTDVinaDALWSFVKKSKsTVNRKNXASAIAGXRX-------VLP
                     KTVAXCS--VVVLANIPTSSLKHXLRILKAKPPVtIGKRMVETVTHANYPMRXSItxV
                     RPXRNAXSgpmeyyGNRPGAGIDDKTNLV-KSGNRV"
                     /note="IS1_ORF1    e-value= 1.6e-06 complete sequence hit
                     coverage= 100%, between model( 239 aa) positions 1; 239
                     length is 229aa with 25 gaps, 10 stops, absolute frame=
                     Minus1"
     CDS             complement(4134977..4134790)
                     /colour="255 0 0"
                     /evidence=predicted
                     /translation="GKTSKGSQRYRCLHCXRTFSETFdtLYYHRQisPQTLQTILQSH
                     AEGSSLRGLARITGVAYNT"
                     /note="IS1_ORF2    e-value= 4.3e-06 fragment hit coverage=
                     24.89%, between model( 237 aa) positions 21; 79 length is
                     63aa with 0 gaps, 1 stops, absolute frame= Minus1"
     CDS             complement(3406541..3406153)
                     /colour="255 0 0"
                     /evidence=predicted
                     /translation="FFMDESGFSLVPCIPY-GWQPIG-------------TYL-EIPT
                     RSSKRLNVLGFLSRRQGL--HAYTSEQTITSEVVShCIDTFFADV--ELPTVIVVDQA
                     PIHTSQsiyEMKAEWAERG--ITLFELPSYSPHLNLIERLWQFMKYQWI"
                     /note="IS630  e-value= 6.5e-11 fragment hit coverage=
                     39.10%, between model( 376 aa) positions 187; 333 length
                     is 130aa with 21 gaps, 0 stops, absolute frame= Minus1"
     CDS             complement(2707001..2705968)
                     /colour="255 0 0"
                     /evidence=predicted
                     /translation="QRFIQGLSPETIHLLSRIHRHSYHHQVRQRAHCILLSFEGFNVT
                     ELMSIFAVTRKTVYTWLDDWDNHC---LVGLYDQPGRGRKPKLNDVQ-KEQIRAWAKM
                     TP---------HNLNAVLAKIKEAWNIAVSKTTLKRILKSCSMSWRRLRRVVAGQPDP
                     VEYETK-----RHQLEVLKRQEEKGELdlrYLDESGFCLVPYVPY-AWQEKG------
                     --------ETLGLPSQRSSRFNVLGLMNRHNDL-TSYVFDKSITSAVVVACIDDFSRT
                     C--DQHTVVVMDQASVHKNAEIEEKIEDWKAkNVEIFWLPTYSPHLNLIEIFWRFMKY
                     EWIEFAAYKCLGSLSLYIDKILKGFgKDYVIDFGXVLTKAVFISDIY"
                     /note="IS630  e-value= 1.1e-19 complete sequence hit
                     coverage= 100%, between model( 376 aa) positions 1; 376
                     length is 345aa with 36 gaps, 1 stops, absolute frame=
                     Minus1"
     CDS             complement(2707001..2706100)
                     /colour="255 0 0"
                     /evidence=predicted
                     /translation="QRFIQGLSPETIHLLSRIHRHSYHHQVRQRAHCILLSFEGFNVT
                     ELMSIFAVTRKTVYTWLDDWDNHC---LVGLYDQPGRGRKPKLNDVQ-KEQIRAWAKM
                     TP---------HNLNAVLAKIKEAWNIAVSKTTLKRILKSCSMSWRRLRRVVAGQPDP
                     VEYETK-----RHQLEVLKRQEEKGELdlrYLDESGFCLVPYVPY-AWQEKG------
                     --------ETLGLPSQRSSRFNVLGLMNRHNDL-TSYVFDKSITSAVVVACIDDFSRT
                     C--DQHTVVVMDQASVHKNAEIEEKIEDWKAkNVEIFWLPTYSPHLNLIEIFWRFMKY
                     EWI"
                     /note="IS630  e-value= 1.1e-24 fragment hit coverage=
                     88.56%, between model( 376 aa) positions 1; 333 length is
                     301aa with 36 gaps, 0 stops, absolute frame= Minus1"
     CDS             complement(2587106..2586868)
                     /colour="255 0 0"
```

```
                     /evidence=predicted
                     /translation="RNQQHYIRQRLTAIKLLNEGHSRTQVSEQVGCSYDTLTRWMDKY
                     LDGGLQGLVQPI--RHQKPSRLSPEEQQQLKEMVLTQR"
                     /note="IS481 e-value= 1.2e-07 fragment hit coverage=
                     23.36%, between model( 351 aa) positions 1; 82 length is
                     80aa with 2 gaps, 0 stops, absolute frame= Minus1"
     CDS             complement(2587079..2586673)
                     /colour="255 0 0"
                     /evidence=predicted
                     /translation="RLTAIKLLNEGHSRTQVSEQVGCSYDTLTRWMDKYLDGG---LQ
                     GLVQPIRHQKPSRLSPEEQQQLKEMVLTQRPTDYGIDRNMWTGAILAVVIEQRFEVQL
                     KDSRIYELLSELGLSYQRAHRDYA--NADLNAQKEWVAA"
                     /note="IS630 e-value= 1.9e-29 fragment hit coverage=
                     37.50%, between model( 376 aa) positions 30; 170 length
                     is 136aa with 5 gaps, 0 stops, absolute frame= Minus1"
     CDS             complement(2498918..2498134)
                     /colour="255 0 0"
                     /evidence=predicted
                     /translation="CLAAXPNATLSHFQQSMKCLYCVIM--EHNSTHCLPLMGGRHQI
                     KALSQSNRRLKMGLIGFTRSWISPNELNLHRWHQEVEXAASSKRLFEK-----XVKLS
                     RYXsVNTQTLDRTXRAMPTAYDSDLTTLQWELLEPLIPAAKP-GGRPRT-TDMLSVLN
                     AIFYLVVTGCQWRQLPHDFPCWSTVYSYFRRWRDDGTWVHINEHLRMQERVSE-----
                     -----------------------DRHPSPSAAICDAQSVKVGNPRCHSIGFDGgkmv
                     KGRKRHVLVDTLGLVL-MVMVTAAN"
                     /note="IS5_IS427   e-value= 7.3e-07 complete sequence hit
                     coverage= 100%, between model( 296 aa) positions 1; 296
                     length is 262aa with 39 gaps, 5 stops, absolute frame=
                     Minus1"
     CDS             complement(2498615..2497765)
                     /colour="255 0 0"
                     /evidence=predicted
                     /translation="TQTLDRTXRAM-----PTAYDSDLTTLQWELLEPLIPAAK----
                     ---PGGRPRTTDMLSVLNAIFYLVVTGCQWRQLPHDFPCWSTVYSYFRRWRDDGTWVH
                     INEHLRMQERVSEDRHPSPSAAICDAQSVKVGNPRCH-SIGFDGGKMVKGRKRHVLVD
                     TLGLVLMVMVTAANISDQR-GAKILFWKARRQGaslSRLVRIWADAGYQGQALMKWVM
                     DRSQYVLEVIKRSDNLAGFQVVSKRWIVERTFGWLLWSRRLNKDYEVLTRTAEALVYV
                     AMIRLMVRRLAQEHXNFSNSLX"
                     /note="IS5_IS1031  e-value= 3.2e-108 complete sequence hit
                     coverage= 100%, between model( 295 aa) positions 1; 295
                     length is 284aa with 14 gaps, 3 stops, absolute frame=
                     Minus1"
     CDS             complement(2498573..2497765)
                     /colour="255 0 0"
                     /evidence=predicted
                     /translation="YDSDLTTLQWELLEPLIPAAK-------PGGRPRTTDMLSVLNA
                     IFYLVVTGCQWRQLPHDFPCWSTVYSYFRRWRDDGTWVHINEHLRMQERVSEDRHPSP
                     SAAICDAQSVKVGNPRCH-SIGFDGGKMVKGRKRHVLVDTLGLVLMVMVTAANISDQR
                     -GAKILFWKARRQGaslSRLVRIWADAGYQGQALMKWVMDRSQYVLEVIKRSDNLAGF
                     QVVSKRWIVERTFGWLLWSRRLNKDYEVLTRTAEALVYVAMIRLMVRRLAQEHXNFSN
                     SLX"
                     /note="IS5_IS1031  e-value= 2.1e-110 fragment hit
                     coverage= 93.56%, between model( 295 aa) positions 20;
                     295 length is 270aa with 9 gaps, 2 stops, absolute frame=
                     Minus1"
     CDS             complement(2498564..2498326)
                     /colour="255 0 0"
                     /evidence=predicted
                     /translation="DLTTLQWELLEPLIPAAKP-GGRPRT-TDMLSVLNAIFYLVVTG
                     CQWRQLPHDFPCWSTVYSYFRRWRDDGTWVHINEHLRM"
                     /note="IS5_IS427   e-value= 3.3e-18 fragment hit coverage=
```

```
                     27.70%, between model( 296 aa) positions 125; 206 length
                     is 80aa with 2 gaps, 0 stops, absolute frame= Minus1"
     CDS             complement(2405255..2405098)
                     /colour="255 0 0"
                     /evidence=predicted
                     /translation="HHPHPRVQRRMEVLYLKSQGLPHAQICQLCQISRPTLAKTLRLY
                     QQGGIEGLK"
                     /note="IS481 e-value= 1.1e-08 fragment hit coverage=
                     15.10%, between model( 351 aa) positions 1; 53 length is
                     53aa with 0 gaps, 0 stops, absolute frame= Minus1"
     CDS             complement(2383214..2382808)
                     /colour="255 0 0"
                     /evidence=predicted
                     /translation="MLLVRPISTESLRLLHRIYHSSRHHQVRQRAHCLILFAQGWPPY
                     TLASLFSVSPKTVYNWLKAWNNRG---FAGLYNHPGRGRKPMFNPDQQQQIYEWT-QA
                     SPIQ---------LNQVLAQIEQQWSVRVSKATVKRVLKQMDMSWHR"
                     /note="IS630 e-value= 8.7e-15 fragment hit coverage=
                     39.63%, between model( 376 aa) positions 1; 149 length is
                     136aa with 13 gaps, 0 stops, absolute frame= Minus1"
     CDS             complement(2383154..2382817)
                     /colour="255 0 0"
                     /evidence=predicted
                     /translation="SSRHHQVRQRAHCLILFAQGWPPYTLASLFSVSPKTVYNWLKAW
                     NNRGFAGLYNH---PGRGRKPMfNPDQQQQIYEWTQASPIQLNQVLAQIEQQWSVR--
                     -VSKATVKRVLKQMDMS"
                     /note="IS481 e-value= 3.5e-09 fragment hit coverage=
                     33.62%, between model( 351 aa) positions 1; 118 length is
                     113aa with 6 gaps, 0 stops, absolute frame= Minus1"
     CDS             complement(2285066..2284843)
                     /colour="255 0 0"
                     /evidence=predicted
                     /translation="SQMSTLTCPNCRSQN-VVKNGRIHNGKQNHKCKTCGRQFV--EA
                     PQQKRIDSSTKGLIDKLLLEKIPLAGIARVCDVS"
                     /note="IS1_ORF1    e-value= 3.6e-14 fragment hit coverage=
                     32.64%, between model( 239 aa) positions 1; 78 length is
                     75aa with 3 gaps, 0 stops, absolute frame= Minus1"
     CDS             complement(1924193..1923499)
                     /colour="255 0 0"
                     /evidence=predicted
                     /translation="FKHLHLGMIsDIKRKSLPAIARAVGLENHQSLHHFLSESPWVAS
                     hLSDkRLDIILKILDGRRLILLID------------------ETGDCKKGKSTDYVKR
                     QYIGNVGKKENGIvAVTAYGLFQGMILPLSFEVYKPR---------ErLKEEDEYLSK
                     PQIAAGMIRQLQGMGFCFELVLADSLYGEAQTNFVNVLEELKLPYILAIRSNHAVWLP
                     AEQAVFAEPWQSFERTFSNGTTETRYrQEIIYGqrhhQKRY"
                     /note="IS4   e-value= 4.7e-14 fragment hit coverage=
                     51.23%, between model( 486 aa) positions 1; 249 length is
                     232aa with 27 gaps, 0 stops, absolute frame= Minus1"
     CDS             complement(1924193..1923067)
                     /colour="255 0 0"
                     /evidence=predicted
                     /translation="FKHLHLGMIsDIKRKSLPAIARAVGLENHQSLHHFLSESPWVAS
                     hLSDkRLDIILKILDGRRLILLID------------------ETGDCKKGKSTDYVKR
                     QYIGNVGKKENGIvAVTAYGLFQGMILPLSFEVYKPR---------ErLKEEDEYLSK
                     PQIAAGMIRQLQGMGFCFELVLADSLYGEAQTNFVNVLEELKLPYILAIRSNHAVWLP
                     AEQAVFAEPWQSFERTFSNGT--------------------------------TETRYR
                     QEIIYGQRHHQKRyWLLTTDPQTLPENSTS-----------------------YVMAA
                     APE----IKLDEIGDCYGFRTWIEYGLKQSKD-TLGWADFRMTHYEQIEKWWEIVMSA
                     FLMVSLFADVFNDS-------------------------CPVAHQHFAQHPwWDNQNG
                     WKNLLNNVR--LIIQPLIS----WNLLKRWLEVFPSRALKKGF"
                     /note="IS4   e-value= 9.9e-07 complete sequence hit
```

coverage= 100%, between model(486 aa) positions 1; 486 length is 376aa with 117 gaps, 0 stops, absolute frame= Minus1"
CDS complement(1895579..1895383)
/colour="255 0 0"
/evidence=predicted
/translation="ASPIELSQQQHHCLTQIVRQTTNPYRLVRRASIILSAASGESNT
QISRQWQLDRNQVRYWRQRWLD"
/note="IS630 e-value= 4e-07 fragment hit coverage= 17.55%, between model(376 aa) positions 1; 66 length is 66aa with 0 gaps, 0 stops, absolute frame= Minus1"
CDS complement(1336244..1335394)
/colour="255 0 0"
/evidence=predicted
/translation="TQTLDRTXRAM-----PTAYDSDLTAEQWELLEPLIPAAK----
---PGGRPRTTDMRSVLNAILYLVVTGCQWRQLPHDFPCWSTVYSYFRRWRDDGTWVH
INEHLRMQERVSEDRHPSPRAAICDAQSVKVGNPRCH-SIGFDGGKQIKGRKRHVLVD
TLGLVLMVIVTAANISDQR-GAKILFWKARRQGaslSRLVRIWADAGYQGQAFMKWVM
DRFQYVLEVIKRSDNLAGFQVVPKRWIVERTFGWLLWSRRLNKDYELLTRTAEALAYV
AMIRLMVRRLAQEYXNFSNSLX"
/note="IS5_IS1031 e-value= 3e-112 complete sequence hit coverage= 100%, between model(295 aa) positions 1; 295 length is 284aa with 14 gaps, 3 stops, absolute frame= Minus1"
CDS complement(1336202..1335394)
/colour="255 0 0"
/evidence=predicted
/translation="YDSDLTAEQWELLEPLIPAAK-------PGGRPRTTDMRSVLNA
ILYLVVTGCQWRQLPHDFPCWSTVYSYFRRWRDDGTWVHINEHLRMQERVSEDRHPSP
RAAICDAQSVKVGNPRCH-SIGFDGGKQIKGRKRHVLVDTLGLVLMVIVTAANISDQR
-GAKILFWKARRQGaslSRLVRIWADAGYQGQAFMKWVMDRFQYVLEVIKRSDNLAGF
QVVPKRWIVERTFGWLLWSRRLNKDYELLTRTAEALAYVAMIRLMVRRLAQEYXNFSN
SLX"
/note="IS5_IS1031 e-value= 2.4e-114 fragment hit coverage= 93.56%, between model(295 aa) positions 20; 295 length is 270aa with 9 gaps, 2 stops, absolute frame= Minus1"
CDS complement(1336193..1335955)
/colour="255 0 0"
/evidence=predicted
/translation="DLTAEQWELLEPLIPAAKP-GGRPRT-TDMRSVLNAILYLVVTG
CQWRQLPHDFPCWSTVYSYFRRWRDDGTWVHINEHLRM"
/note="IS5_IS427 e-value= 1.2e-20 fragment hit coverage= 27.70%, between model(296 aa) positions 125; 206 length is 80aa with 2 gaps, 0 stops, absolute frame= Minus1"
CDS complement(1225547..1225171)
/colour="255 0 0"
/evidence=predicted
/translation="EHRKSEDFALCMQYLVDGLFPQADKLHVVLDNLNTHspaalyKT
FNPDEALRILK-RIQFHYTPKHGSWLNMIEFEFSALSRQCLNRRIP-DIEQLRYEVTA
WEKRRNHDKATVNWLFTVDDARTKLS"
/note="IS630 e-value= 2.3e-07 fragment hit coverage= 32.45%, between model(376 aa) positions 255; 376 length is 126aa with 2 gaps, 0 stops, absolute frame= Minus1"
CDS complement(1184315..1183279)
/colour="255 0 0"
/evidence=predicted
/translation="PMIKIDFTAEEIQQLNYERYHHPHPRVQRRMEVLYLKSQGLSHT
QICQLCQISRPTLAKTLRQYQQGG---IEGLKTLEYKGQPSLLNAHS-DSVRAYFEQH
PP---------RTSAEAQAVIEQLTGIKRSPTQIKAFLKRIGCRYRKVGYVPGKSSLP

```
                     -EKIEEQEQFRHTRLEPLleeAQRQERLVFFVDAAHFVHRAYLGF-VWCITRI-----
                     ---------FIPSPS-GRQRFNVLGALDAITKDLVSVTNHTYINSHSMCLLLAKLALL
                     DPV-IPISVILDNARYQKCQLVTDFAEIVD--IELVYLPSYSRHLNLIERLWRFVRKE
                     CLYSKYYADFPAFKGAIQQCIDQCNGEHKAKLTTLLSLKFQSFKK"
                     /note="IS630 e-value= 3.5e-21 complete sequence hit
                     coverage= 100%, between model( 376 aa) positions 1; 376
                     length is 346aa with 33 gaps, 0 stops, absolute frame=
                     Minus1"
     CDS             complement(1184315..1183279)
                     /colour="255 0 0"
                     /evidence=predicted
                     /translation="PMIKIDFTAEEIQQLNYERYHHPHPRVQRRMEVLYLKSQGLSHT
                     QICQLCQISRPTLAKTLRQYQQGG---IEGLKTLEYKGQPSLLNAHS-DSVRAYFEQH
                     PP---------RTSAEAQAVIEQLTGIKRSPTQIKAFLKRIGCRYRKVGYVPGKSSLP
                     -EKIEEQEQFRHTRLEPLleeAQRQERLVFFVDAAHFVHRAYLGF-VWCITRI-----
                     ---------FIPSPS-GRQRFNVLGALDAITKDLVSVTNHTYINSHSMCLLLAKLALL
                     DPV-IPISVILDNARYQKCQLVTDFAEIVD--IELVYLPSYSRHLNLIERLWRFVRKE
                     CLYSKYYADFPAFKGAIQQCIDQCNGEHKAKLTTLLSLKFQSFKK"
                     /note="IS630 e-value= 3.1e-22 complete sequence hit
                     coverage= 100%, between model( 376 aa) positions 1; 376
                     length is 346aa with 33 gaps, 0 stops, absolute frame=
                     Minus1"
     CDS             complement(1184255..1184098)
                     /colour="255 0 0"
                     /evidence=predicted
                     /translation="HHPHPRVQRRMEVLYLKSQGLSHTQICQLCQISRPTLAKTLRQY
                     QQGGIEGLK"
                     /note="IS481 e-value= 3.7e-09 fragment hit coverage=
                     15.10%, between model( 351 aa) positions 1; 53 length is
                     53aa with 0 gaps, 0 stops, absolute frame= Minus1"
     CDS             complement(1119713..1118947)
                     /colour="255 0 0"
                     /evidence=predicted
                     /translation="--IYPSFITLGRXKNRLLDLLHnxvcdMVLSHSNAGRPPKLCRP
                     DQLLVALQDWREYRTYFHIAGDWGVSESTVCRIVQQVEtaHELRTLP----PTRQETS
                     NTRIXAAXCCRDGCDXDTDXTSpKRTKSVLCRKKKRHTLKCQIIADRNTLEIICLSFG
                     KGRRHDFQVFKVSGVHIHPDTESLLDSGYQG------NSYVPLKKPQQGELTSLEREY
                     NHALSQERIGIEHINRSLKMFRILSERYCNRRRRYSLRCNLIAAIYNYEL"
                     /note="IS5_ISL2    e-value= 6.5e-76 complete sequence hit
                     coverage= 100%, between model( 260 aa) positions 1; 260
                     length is 256aa with 12 gaps, 5 stops, absolute frame=
                     Minus1"
     CDS             complement(1119629..1118947)
                     /colour="255 0 0"
                     /evidence=predicted
                     /translation="SHSNAGRPPKLCRPDQLLVALQDWREYRTYFHIAGDWGVSESTV
                     CRIVQQVEtaHELRTLP----PTRQETSNTRIXAAXCCRDGCDXDTDXTSpKRTKSVL
                     CRKKKRHTLKCQIIADRNTLEIICLSFGKGRRHDFQVFKVSGVHIHPDTESLLDSGYQ
                     G------NSYVPLKKPQQGELTSLEREYNHALSQERIGIEHINRSLKMFRILSERYCN
                     RRRRYSLRCNLIAAIYNYEL"
                     /note="IS5_ISL2    e-value= 1.1e-77 fragment hit coverage=
                     90.38%, between model( 260 aa) positions 26; 260 length
                     is 228aa with 10 gaps, 4 stops, absolute frame= Minus1"
     CDS             complement(826598..826267)
                     /colour="255 0 0"
                     /evidence=predicted
                     /translation="QRVKDRAAMIRLSHQGMYVEKIAALFQCNVRTVRQTLHRWQQKG
                     ---LGGLWDAPHPGAQRRWNPED-IEYLETCLRQEERTY-------NSQQLSQKLAKE
                     RNVHLSADRIRQILKKRGXS"
                     /note="IS630 e-value= 8.8e-10 fragment hit coverage=
```

32.45%, between model(376 aa) positions 25; 146 length
is 111aa with 11 gaps, 1 stops, absolute frame= Minus1"
CDS complement(741935..741721)
/colour="255 0 0"
/evidence=predicted
/translation="REIHEAIGVSIRTIERVRMRFVEEG---LEAAINQRsGAGRKRK
IQEEQEAHLIALRCSEPPVG----HARWTLRLLAD"
/note="IS630 e-value= 2.5e-08 fragment hit coverage=
20.74%, between model(376 aa) positions 44; 121 length
is 72aa with 7 gaps, 0 stops, absolute frame= Minus1"
CDS complement(669422..669199)
/colour="255 0 0"
/evidence=predicted
/translation="SQMSTLTCPNCRSQN-VVKNGRIHNGKQNHKCKTCGRQFV--EA
PQQKRIDSSTKGLIDKLLLEKIPLAGIARVCDVS"
/note="IS1_ORF1 e-value= 3.6e-14 fragment hit coverage=
32.64%, between model(239 aa) positions 1; 78 length is
75aa with 3 gaps, 0 stops, absolute frame= Minus1"
CDS complement(517589..517393)
/colour="255 0 0"
/evidence=predicted
/translation="GKHKQILLVLDGAGWHTC---KNRVVPPG--IHLKILPPYSPEL
QPAERLWRLADEPLAN-QCFETLDDLED"
/note="IS630 e-value= 1.4e-09 fragment hit coverage=
19.15%, between model(376 aa) positions 275; 346 length
is 66aa with 6 gaps, 0 stops, absolute frame= Minus1"
CDS complement(481079..480364)
/colour="255 0 0"
/evidence=predicted
/translation="YLSPES-LXCRASSKVVSMXSTVGSPS---------ILARISDL
FPHCWRLGSLXINRLPYCppGRDRSDELRTLSLTRSEISATR---------VRATGCR
CDGCDXNAHXTSPDKTKSVLF----RKKKRHTFKYQIIANRNTLEIICLNVGPGRRHD
FQIFKVSGIHIHPDTESLQDSGYQGIAAYHTNSYVPLKKPQHGELTSLQREYNRALSQ
ERMGIEHINRSLKIFRILSERYRNRRRRYALRCNLIAAIYNYEL"
/note="IS5_ISL2 e-value= 1e-38 complete sequence hit
coverage= 100%, between model(260 aa) positions 1; 260
length is 239aa with 23 gaps, 5 stops, absolute frame=
Minus1"
CDS complement(480764..480364)
/colour="255 0 0"
/evidence=predicted
/translation="KKKRHTFKYQIIANRNTLEIICLNVGPGRRHDFQIFKVSGIHIH
PDTESLQDSGYQGIAAYHTNSYVPLKKPQHGELTSLQREYNRALSQERMGIEHINRSL
KIFRILSERYRNRRRRYALRCNLIAAIYNYEL"
/note="IS5_ISL2 e-value= 2.5e-62 fragment hit coverage=
51.54%, between model(260 aa) positions 127; 260 length
is 134aa with 0 gaps, 0 stops, absolute frame= Minus1"
CDS complement(476648..475171)
/colour="255 0 0"
/evidence=predicted
/translation="DSYHTRVYMNGRDLGLKQAEAAYIAEISTRTGQRIEAGTHQPNR
-GRLQD-------QRTVPDPLADVWE---DELEPMLRRDPRLKPMTLYEYLQD-KYPG
QYPQVlrtlQRRVRTWKALHGPS-----PEVMFELRHEPGVQGFSGFTELKGITITIA
GQPFEHLIYHYRLGYSGWRYAQIIQGgESFVALSEGLQNAFAACGGVPQQHRTDSLSA
AYRNMGGRRS-KNLTRLYDELCDHYRLEPTRNNKG--IAHENGSIESPHGHLKNRIKQ
AIYLRGSAD-----FTSVAEYQALIDAQVAKLNQQCQTK------YEQEKDHLQPLPK
YRTPDYEVLTA----KVSKRSTIDVRCILYTVPSRLIGRQLELHLYHDRIVGYLERHP
VVELPRKRVSGkGKRRDRCINYRHVIGSMRLKPRAFIYCTWQSDLLPNPEYRQIWEQL
KAQFDLEQAAKIIVEALYIAAVQDKEQAVAVYLQQQLQASSLTLNRLKKQFEPPQMKQ
VPDLSIeQHS----LELYDKLLPS"

/note="IS21_ORF1   e-value= 5e-20 complete sequence hit coverage= 100%, between model(525 aa) positions 1; 525 length is 493aa with 39 gaps, 0 stops, absolute frame= Minus1"

CDS complement(476378..475549)
/colour="255 0 0"
/evidence=predicted
/translation="QYPQVLRTLQRRVRTWKALHGPS-----PEVMFELRHEPGVQGF
SGFTELKGITITIAGQPFEHLIYHYRLGYSGWRYAQIIQGgESFVALSEGLQNAFAAC
GGVPQQHRTDSLSAAYRNMGGRRS-KNLTRLYDELCDHYRLEPTRNNKG--IAHENGS
IESPHGHLKNRIKQAIYLRGSAD-----FTSVAEYQALIDAQVAKLNQQCQTK-----
-YEQEKDHLQPLPKYRTPDYEVLTA----KVSKRSTIDVRCILYTVPSRLIGRQLELH
LYHDRIVGY-----LERHPVVELPRKRVS"
/note="IS21_ORF1   e-value= 3.2e-28 fragment hit coverage= 57.90%, between model(525 aa) positions 99; 402 length is 277aa with 28 gaps, 0 stops, absolute frame= Minus1"

CDS complement(408932..408064)
/colour="255 0 0"
/evidence=predicted
/translation="EKXVKLSRYXSVNTQTLDRTXramptaYDSDLTTLQWELLEPLI
PAAKPG-----GRPRTTDML--------------------SVLNAIFYLVVTGCQWRQ
--LPHDFPCWSTVYSYFRRWRDDGTWSQINEHLRMQVRVSED---RHPSPSAAICDAQ
SVK------------------------------VGNpRcHLIGFD--------GGKM-
---VKGRKRHVLVD-TL
GLVLMVMVTAANISDQRGAK----------------------ILFWKAQRQGASLSRL
VR----IWADAGYQGQALMKWVMDRFQYVLEVIKRSDNLAGFQVVPKRWIVERTFGWL
LWSRRLNKDYEVLTRTAEALVYVAMIRLMVRRLAQEHX"
/note="IS5_IS5   e-value= 1.3e-09 complete sequence hit coverage= 100%, between model(422 aa) positions 1; 422 length is 290aa with 140 gaps, 4 stops, absolute frame= Minus1"

CDS complement(408893..408043)
/colour="255 0 0"
/evidence=predicted
/translation="TQTLDRTXRAM-----PTAYDSDLTTLQWELLEPLIPAAK----
---PGGRPRTTDMLSVLNAIFYLVVTGCQWRQLPHDFPCWSTVYSYFRRWRDDGTWSQ
INEHLRMQVRVSEDRHPSPSAAICDAQSVKVGNPRCH-LIGFDGGKMVKGRKRHVLVD
TLGLVLMVMVTAANISDQR-GAKILFWKAQRQGaslSRLVRIWADAGYQGQALMKWVM
DRFQYVLEVIKRSDNLAGFQVVPKRWIVERTFGWLLWSRRLNKDYEVLTRTAEALVYV
AMIRLMVRRLAQEHXNFSNSLL"
/note="IS5_IS1031 e-value= 4.2e-111 complete sequence hit coverage= 100%, between model(295 aa) positions 1; 295 length is 284aa with 14 gaps, 2 stops, absolute frame= Minus1"

CDS complement(408851..408043)
/colour="255 0 0"
/evidence=predicted
/translation="YDSDLTTLQWELLEPLIPAAK-------PGGRPRTTDMLSVLNA
IFYLVVTGCQWRQLPHDFPCWSTVYSYFRRWRDDGTWSQINEHLRMQVRVSEDRHPSP
SAAICDAQSVKVGNPRCH-LIGFDGGKMVKGRKRHVLVDTLGLVLMVMVTAANISDQR
-GAKILFWKAQRQGaslSRLVRIWADAGYQGQALMKWVMDRFQYVLEVIKRSDNLAGF
QVVPKRWIVERTFGWLLWSRRLNKDYEVLTRTAEALVYVAMIRLMVRRLAQEHXNFSN
SLL"
/note="IS5_IS1031 e-value= 2.8e-113 fragment hit coverage= 93.56%, between model(295 aa) positions 20; 295 length is 270aa with 9 gaps, 1 stops, absolute frame= Minus1"

CDS complement(408842..408604)
/colour="255 0 0"
/evidence=predicted

```
                     /translation="DLTTLQWELLEPLIPAAKP-GGRPRT-TDMLSVLNAIFYLVVTG
                     CQWRQLPHDFPCWSTVYSYFRRWRDDGTWSQINEHLRM"
                     /note="IS5_IS427   e-value= 2e-19 fragment hit coverage=
                     27.70%, between model( 296 aa) positions 125; 206 length
                     is 80aa with 2 gaps, 0 stops, absolute frame= Minus1"
     CDS             complement(129620..128584)
                     /colour="255 0 0"
                     /evidence=predicted
                     /translation="PMIKIDFTAEEIQQLNYERYHHPHPRVQRRMEVLYLKSQGLSHT
                     QICQLCQISRPTLAKTLRQYQQGG---IEGLKTLEYKGQPSLLNAHS-DSVRAYFEQH
                     PP---------RTSAEAQAVIEQLTGIKRSPTQIKAFLKRIGCRYRKVGYVPGKSSLP
                     -EKIEEQEQFRHTRLEPLleeAQRQERLVFFVDAAHFVHRAYLGF-VWCITRI-----
                     ---------FIPSPS-GRQRFNVLGALDAITKDLVSVTNHTYINSHSMCLLLAKLALL
                     DPV-IPISVILDNARYQKCQLVTDFAEIVD--IELVYLPSYSPHLNLIERLWRFVRKE
                     CLYSKYYADFPAFKGAIQQCIDQCNGEHKAKLTTLLSLKFQSFKK"
                     /note="IS630  e-value= 4.9e-24 complete sequence hit
                     coverage= 100%, between model( 376 aa) positions 1; 376
                     length is 346aa with 33 gaps, 0 stops, absolute frame=
                     Minus1"
     CDS             complement(129620..128584)
                     /colour="255 0 0"
                     /evidence=predicted
                     /translation="PMIKIDFTAEEIQQLNYERYHHPHPRVQRRMEVLYLKSQGLSHT
                     QICQLCQISRPTLAKTLRQYQQGG---IEGLKTLEYKGQPSLLNAHS-DSVRAYFEQH
                     PP---------RTSAEAQAVIEQLTGIKRSPTQIKAFLKRIGCRYRKVGYVPGKSSLP
                     -EKIEEQEQFRHTRLEPLleeAQRQERLVFFVDAAHFVHRAYLGF-VWCITRI-----
                     ---------FIPSPS-GRQRFNVLGALDAITKDLVSVTNHTYINSHSMCLLLAKLALL
                     DPV-IPISVILDNARYQKCQLVTDFAEIVD--IELVYLPSYSPHLNLIERLWRFVRKE
                     CLYSKYYADFPAFKGAIQQCIDQCNGEHKAKLTTLLSLKFQSFKK"
                     /note="IS630  e-value= 6.7e-25 complete sequence hit
                     coverage= 100%, between model( 376 aa) positions 1; 376
                     length is 346aa with 33 gaps, 0 stops, absolute frame=
                     Minus1"
     CDS             complement(129560..129403)
                     /colour="255 0 0"
                     /evidence=predicted
                     /translation="HHPHPRVQRRMEVLYLKSQGLSHTQICQLCQISRPTLAKTLRQY
                     QQGGIEGLK"
                     /note="IS481  e-value= 3.7e-09 fragment hit coverage=
                     15.10%, between model( 351 aa) positions 1; 53 length is
                     53aa with 0 gaps, 0 stops, absolute frame= Minus1"
     CDS             complement(105725..105655)
                     /colour="255 0 0"
                     /evidence=predicted
                     /translation="HVHMLISIPPKYSVLNVMGYIKGK"
                     /note="IS200  e-value= 5.3e-11 fragment hit coverage=
                     15.58%, between model( 154 aa) positions 61; 84 length is
                     24aa with 0 gaps, 0 stops, absolute frame= Minus1"
     CDS             complement(105725..105655)
                     /colour="255 0 0"
                     /evidence=predicted
                     /translation="HVHMLISIPPKYSVLNVMGYIKGK"
                     /note="IS200_IS605 e-value= 2.1e-10 fragment hit
                     coverage= 15.69%, between model( 153 aa) positions 61; 84
                     length is 24aa with 0 gaps, 0 stops, absolute frame=
                     Minus1"
     CDS             complement(105725..105655)
                     /colour="255 0 0"
                     /evidence=predicted
                     /translation="HVHMLISIPPKYSVLNVMGYIKGK"
```

/note="IS605 e-value= 2.8e-08 fragment hit coverage= 15.79%, between model(152 aa) positions 61; 84 length is 24aa with 0 gaps, 0 stops, absolute frame= Minus1"

CDS complement(100253..99016)
/colour="255 0 0"
/evidence=predicted
/translation="IRIPLNLPDI--RVLELSKTERGDWL------IKIESTLQGTTC
HQCGREITDL-HCHDQ-PFRIRHLPLFEVPVYLEIRPKRYRCKYCDDhpTTTQHLEWH
EPRSPNTKAYERWLLRILIN-STVSDVARKLGVSEDIVSGIIDRWIAAQVDWSEYADL
KVMGMDEISLKRG--HR---DFVVLITIPTTdgVDILAVLADRKQQTVANFLQSIP-I
DLRQTIERVCTDMYQGFVSAVREQLPQAKIVIDRFHVTKAYRNCADTVRKREVKRLRR
ELS------------KQEYD-----------SIKGAMWPFRKRPENLKES-EQQLLER
LFA--YSPQLKQAYNLREKLTQIFEGRYTKHGAKcAIRAWCKQVLKSDiKEFDCFLTT
INNWMDEMTNYFLEG-WTSGFVEGFNNRVKVLKRRCYGIFDIERLFQRISLDLNGYQT
FAVTXTL"
/note="ISL3 e-value= 1.8e-43 complete sequence hit coverage= 100%, between model(451 aa) positions 1; 451 length is 413aa with 44 gaps, 1 stops, absolute frame= Minus1"

CDS complement(100094..99016)
/colour="255 0 0"
/evidence=predicted
/translation="RIRHLPLFEVPVYLEIRPKRYRCKYCDDhpTTTQHLEWHEPRSP
NTKAYERWLLRILIN-STVSDVARKLGVSEDIVSGIIDRWIAAQVDWSEYADLKVMGM
DEISLKRG--HR---DFVVLITIPTTdgVDILAVLADRKQQTVANFLQSIP-IDLRQT
IERVCTDMYQGFVSAVREQLPQAKIVIDRFHVTKAYRNCADTVRKREVKRLRRELS--
----------KQEYD-----------SIKGAMWPFRKRPENLKES-EQQLLERLFA--
YSPQLKQAYNLREKLTQIFEGRYTKHGAKcAIRAWCKQVLKSDiKEFDCFLTTINNWM
DEMTNYFLEG-WTSGFVEGFNNRVKVLKRRCYGIFDIERLFQRISLDLNGYQTFAVTX
TL"
/note="ISL3 e-value= 1e-49 fragment hit coverage= 86.03%, between model(451 aa) positions 64; 451 length is 360aa with 34 gaps, 1 stops, absolute frame= Minus1"

CDS complement(6333589..6332766)
/colour="255 0 0"
/evidence=predicted
/translation="XRIKXKNYILINSNXXKINIKLNYKLKIRRXAF------IGNEV
PIXLKXLNYXQLDKLKXRLMQWLKKKLVKQRFKPTQLVXSIVEVVtlrtfpklsaITX
RLFEKXVKLSRYXsVNTQTLDRTXRAMPTAYDSDLTTLQWELLEPLIPAAKP-GGRPR
T-TDMLSVLNAIFYLVVTGCQWRQLPHDFPCWSTVYSYFRRWRDDGTWSQINEHLRMQ
VRVSE-----------------------------DRHPSPSAAICDAQSVkvgnpRCH
LIGFDGGKMVKGRKRHVLVDTLGLVLMVMVTAANIS"
/note="IS5_IS427 e-value= 2.1e-06 complete sequence hit coverage= 100%, between model(296 aa) positions 1; 296 length is 275aa with 37 gaps, 14 stops, absolute frame= Minus2"

CDS complement(6333292..6332424)
/colour="255 0 0"
/evidence=predicted
/translation="EKXVKLSRYXSVNTQTLDRTXramptaYDSDLTTLQWELLEPLI
PAAKPG-----GRPRTTDML--------------------SVLNAIFYLVVTGCQWRQ
--LPHDFPCWSTVYSYFRRWRDDGTWSQINEHLRMQVRVSED---RHPSPSAAICDAQ
SVK------------------------------VGNpRcHLIGFD--------GGKM-
--VKGRKRHVLVD-TL
GLVLMVMVTAANISDQRGAK----------------------ILFWKAQRQGASLSRL
VR----IWADAGYQGQALMKWVMDRFQYVLEVVKRSDNLAGFQVIPKRWIVERTFGWL
LWSRRLNKDYEVLTRTAEALVYYVAMIRLMVRRLAQEHX"
/note="IS5_IS5 e-value= 1.8e-09 complete sequence hit coverage= 100%, between model(422 aa) positions 1; 422 length is 290aa with 140 gaps, 4 stops, absolute frame=

```
                     Minus2"
CDS                  complement(6333253..6332403)
                     /colour="255 0 0"
                     /evidence=predicted
                     /translation="TQTLDRTXRAM-----PTAYDSDLTTLQWELLEPLIPAAK----
                     ---PGGRPRTTDMLSVLNAIFYLVVTGCQWRQLPHDFPCWSTVYSYFRRWRDDGTWSQ
                     INEHLRMQVRVSEDRHPSPSAAICDAQSVKVGNPRCH-LIGFDGGKMVKGRKRHVLVD
                     TLGLVLMVMVTAANISDQR-GAKILFWKAQRQGaslSRLVRIWADAGYQGQALMKWVM
                     DRFQYVLEVVKRSDNLAGFQVIPKRWIVERTFGWLLWSRRLNKDYEVLTRTAEALVYV
                     AMIRLMVRRLAQEHXNFSNSLL"
                     /note="IS5_IS1031  e-value= 3.6e-111 complete sequence hit
                     coverage= 100%, between model( 295 aa) positions 1; 295
                     length is 284aa with 14 gaps, 2 stops, absolute frame=
                     Minus2"
CDS                  complement(6333211..6332403)
                     /colour="255 0 0"
                     /evidence=predicted
                     /translation="YDSDLTTLQWELLEPLIPAAK-------PGGRPRTTDMLSVLNA
                     IFYLVVTGCQWRQLPHDFPCWSTVYSYFRRWRDDGTWSQINEHLRMQVRVSEDRHPSP
                     SAAICDAQSVKVGNPRCH-LIGFDGGKMVKGRKRHVLVDTLGLVLMVMVTAANISDQR
                     -GAKILFWKAQRQGaslSRLVRIWADAGYQGQALMKWVMDRFQYVLEVVKRSDNLAGF
                     QVIPKRWIVERTFGWLLWSRRLNKDYEVLTRTAEALVYVAMIRLMVRRLAQEHXNFSN
                     SLL"
                     /note="IS5_IS1031  e-value= 2.4e-113 fragment hit
                     coverage= 93.56%, between model( 295 aa) positions 20;
                     295 length is 270aa with 9 gaps, 1 stops, absolute frame=
                     Minus2"
CDS                  complement(6333202..6332964)
                     /colour="255 0 0"
                     /evidence=predicted
                     /translation="DLTTLQWELLEPLIPAAKP-GGRPRT-TDMLSVLNAIFYLVVTG
                     CQWRQLPHDFPCWSTVYSYFRRWRDDGTWSQINEHLRM"
                     /note="IS5_IS427   e-value= 2e-19 fragment hit coverage=
                     27.70%, between model( 296 aa) positions 125; 206 length
                     is 80aa with 2 gaps, 0 stops, absolute frame= Minus2"
CDS                  complement(6280795..6279558)
                     /colour="255 0 0"
                     /evidence=predicted
                     /translation="IRIPLNLPDI--RVLELSKTERGDWL------IKIESTLQGTTC
                     HQCGREITDL-HCHDQ-PFRIRHLPLFEVPVYLEIRPKRYRCKYCDDhpTTTQHLEWH
                     EPRSPNTKAYERWLLRILIN-STVSDVARKLGVSEDIVSGIIDRWIAAQVDWSEYADL
                     KVMGMDEISLKRG--HR---DFVVLITIPTTdgVDILAVLADRKQQTVANFLQSIP-I
                     DLRQTIERVCTDMYQGFVSAVREQLPQAKIVIDRFHVTKAYRNCADTVRKREVKRLRR
                     ELS------------KQEYD-----------SIKGAMWPFRKRPENLKES-EQQLLER
                     LFA--YSPQLKQAYNLREKLTQIFEGRYTKHGAKcAIRAWCKQVLKSDiKEFDCFLTT
                     INNWMDEMTNYFLEG-WTSGFVEGFNNRVKVLKRRCYGIFDIERLFQRISLDLNGYQT
                     FAVTXTL"
                     /note="ISL3  e-value= 1.8e-43 complete sequence hit
                     coverage= 100%, between model( 451 aa) positions 1; 451
                     length is 413aa with 44 gaps, 1 stops, absolute frame=
                     Minus2"
CDS                  complement(6280636..6279558)
                     /colour="255 0 0"
                     /evidence=predicted
                     /translation="RIRHLPLFEVPVYLEIRPKRYRCKYCDDhpTTTQHLEWHEPRSP
                     NTKAYERWLLRILIN-STVSDVARKLGVSEDIVSGIIDRWIAAQVDWSEYADLKVMGM
                     DEISLKRG--HR---DFVVLITIPTTdgVDILAVLADRKQQTVANFLQSIP-IDLRQT
                     IERVCTDMYQGFVSAVREQLPQAKIVIDRFHVTKAYRNCADTVRKREVKRLRRELS--
                     ----------KQEYD-----------SIKGAMWPFRKRPENLKES-EQQLLERLFA--
                     YSPQLKQAYNLREKLTQIFEGRYTKHGAKcAIRAWCKQVLKSDiKEFDCFLTTINNWM
```

```
                     DEMTNYFLEG-WTSGFVEGFNNRVKVLKRRCYGIFDIERLFQRISLDLNGYQTFAVTX
                     TL"
                     /note="ISL3  e-value= 1e-49 fragment hit coverage= 86.03%,
                     between model( 451 aa) positions 64; 451 length is 360aa
                     with 34 gaps, 1 stops, absolute frame= Minus2"
     CDS             complement(6250273..6249804)
                     /colour="255 0 0"
                     /evidence=predicted
                     /translation="EAVYILDGLLKN--TSEIQPDTIHGDTQSQSCTVFALAFLLGIT
                     LMPRIRGWQNLAFYRPsRGTRYK--HLDSLFTEVTDWDLIETHLPDMLRVALSIKDGK
                     VQASTLLRKLGTNSRKNKLFQAFHELGGVLRTIFLLQYINDPQMQETIHAETNKCEAF
                     N"
                     /note="Tn3   e-value= 2.6e-14 fragment hit coverage=
                     15.94%, between model( 1004 aa) positions 740; 899 length
                     is 157aa with 4 gaps, 0 stops, absolute frame= Minus2"
     CDS             complement(6238489..6238101)
                     /colour="255 0 0"
                     /evidence=predicted
                     /translation="FFMDESGFSLVPCIPY-GWQPIG-------------TYL-EIPT
                     RSSKRLNVLGFLSRRQGL--HAYTSEQTITSEVVShCIDTFFADV--ELPTVIVVDQA
                     PIHTSQsiyEMKAEWAERG--ITLFELPSYSPHLNLIERLWQFMKYQWI"
                     /note="IS630  e-value= 6.5e-11 fragment hit coverage=
                     39.10%, between model( 376 aa) positions 187; 333 length
                     is 130aa with 21 gaps, 0 stops, absolute frame= Minus2"
     CDS             complement(6232594..6231831)
                     /colour="255 0 0"
                     /evidence=predicted
                     /translation="SPYQHLSLYLKKLRLSHMLTHWESIESQA-MQENWSYAEFLLAL
                     CETEAQRREQARLKRALTEARLPNA-KSFTNFDFSHCPQLNPAPLMQLAAdPGWLERA
                     ENCLILGPSGVGKTHLATGVSKKMLEFGKRVKFFAANALVQHLQQAKLQLQLH-PMLK
                     KLDRYDLLVLDDLGYCKKSEAETSVLFELIAHRYERKSLLITANQPFSQWDDIFT-DS
                     MMAVAAIDRLIHHGLIIKIQADSYRRKSATQRTAQTQSPPQ"
                     /note="IS21_ORF2   e-value= 1.3e-82 complete sequence hit
                     coverage= 100%, between model( 258 aa) positions 1; 258
                     length is 255aa with 4 gaps, 0 stops, absolute frame=
                     Minus2"
     CDS             complement(6232594..6231831)
                     /colour="255 0 0"
                     /evidence=predicted
                     /translation="SPYQHLSLYLKKLRLSHMLTHWESIESQA-MQENWSYAEFLLAL
                     CETEAQRREQARLKRALTEARLPNA-KSFTNFDFSHCPQLNPAPLMQLAAdPGWLERA
                     ENCLILGPSGVGKTHLATGVSKKMLEFGKRVKFFAANALVQHLQQAKLQLQLH-PMLK
                     KLDRYDLLVLDDLGYCKKSEAETSVLFELIAHRYERKSLLITANQPFSQWDDIFT-DS
                     MMAVAAIDRLIHHGLIIKIQADSYRRKSATQRTAQTQSPPQ"
                     /note="IS21_ORF2   e-value= 3.4e-83 complete sequence hit
                     coverage= 100%, between model( 258 aa) positions 1; 258
                     length is 255aa with 4 gaps, 0 stops, absolute frame=
                     Minus2"
     CDS             complement(6209392..6208452)
                     /colour="255 0 0"
                     /evidence=predicted
                     /translation="TKNRXPMSYQIRNWSEYNAGLKQRGSVTFWLEQS--AIVHWLET
                     TPRQKRGASLTYSDTAISTFETVKCIYGLAGRQTEGFLNSLFELMGIELPVCDHSTVS
                     RRKGQLSISLPVIPKQGAIHVVIDSTGVKVYG------------EGEWKTRQHGVS-K
                     RRTWRKLHLSVDESTGEILTGVVTTNDVQDGEVFEDLLEGIDD-----EIEQVSAD-G
                     AYDQSHCYDALMERNATAAIPPRKNA-------------KIWQHGNC--NApphpRDQ
                     NLRAIRKQGRKRWKKQAHYHRRSLAETTMFRFKTIFGGQVRSRNFDNQATELLLQCAA
                     LN-RMIQIAKPETVWVE"
                     /note="IS5_IS903   e-value= 1.1e-96 complete sequence hit
                     coverage= 100%, between model( 347 aa) positions 1; 347
```

```
                     length is 314aa with 37 gaps, 1 stops, absolute frame=
                     Minus2"
     CDS             complement(6209392..6208452)
                     /colour="255 0 0"
                     /evidence=predicted
                     /translation="TKNRXPMSYQIRNWSEYNAGLKQRGSVTFWLEQS--AIVHWLET
                     TPRQKRGASLTYSDTAISTFETVKCIYGLAGRQTEGFLNSLFELMGIELPVCDHSTVS
                     RRKGQLSISLPVIPKQGAIHVVIDSTGVKVYG------------EGEWKTRQHGVS-K
                     RRTWRKLHLSVDESTGEILTGVVTTNDVQDGEVFEDLLEGIDD-----EIEQVSAD-G
                     AYDQSHCYDALMERNATAAIPPRKNA-------------KIWQHGNC--NApphpRDQ
                     NLRAIRKQGRKRWKKQAHYHRRSLAETTMFRFKTIFGGQVRSRNFDNQATELLLQCAA
                     LN-RMIQIAKPETVWVE"
                     /note="IS5_IS903   e-value= 2.2e-96 complete sequence hit
                     coverage= 100%, between model( 347 aa) positions 1; 347
                     length is 314aa with 37 gaps, 1 stops, absolute frame=
                     Minus2"
     CDS             complement(6167842..6167601)
                     /colour="255 0 0"
                     /evidence=predicted
                     /translation="VKLAELIPWESFESEYADQFSQ---TMGAPAKPFRVALGTLIIK
                     EKLGISDAETVEQIRENPYLQYFLGFSEYRESAPFDASML"
                     /note="IS5_IS5     e-value= 2.6e-06 fragment hit coverage=
                     19.91%, between model( 422 aa) positions 23; 106 length
                     is 81aa with 3 gaps, 0 stops, absolute frame= Minus2"
     CDS             complement(6120718..6119784)
                     /colour="255 0 0"
                     /evidence=predicted
                     /translation="CWVGIDVSKQNLDIHIRPSGH-----IFQHPNDKDGISALVASL
                     ESVS-----IERIVLEATGGMELPAAVALSQA---GMAVAVVNPRQARDFA-KATGQI
                     AKTDTIDAAILAHFA--EAIRPETRPLASEESQLLGELVTRRHQIVDMITAEKNRLAA
                     MHGPM--KEHIEQHIE------------------------------------------
                     ---------------------------WLKTQLNELDEQLQRTIRQTPVWCEQAKLL
                     KS---------VPGVGDVLSSVLLVDLP-ELGKLDSR-KIACLVGLAPLNRDSG-KFR
                     -GKRMIWGGRGRVRTALYMPTLVAIRYNPVLKAFYERLVGQGK---PKKVALTACMRK
                     LLIILNAMVKNNQQWNPEMARGAX"
                     /note="IS110 e-value= 6.8e-68 complete sequence hit
                     coverage= 100%, between model( 416 aa) positions 1; 416
                     length is 312aa with 104 gaps, 1 stops, absolute frame=
                     Minus2"
     CDS             complement(6120718..6119784)
                     /colour="255 0 0"
                     /evidence=predicted
                     /translation="CWVGIDVSKQNLDIHIRPSGH-----IFQHPNDKDGISALVASL
                     ESVS-----IERIVLEATGGMELPAAVALSQA---GMAVAVVNPRQARDFA-KATGQI
                     AKTDTIDAAILAHFA--EAIRPETRPLASEESQLLGELVTRRHQIVDMITAEKNRLAA
                     MHGPM--KEHIEQHIE------------------------------------------
                     ---------------------------WLKTQLNELDEQLQRTIRQTPVWCEQAKLL
                     KS---------VPGVGDVLSSVLLVDLP-ELGKLDSR-KIACLVGLAPLNRDSG-KFR
                     -GKRMIWGGRGRVRTALYMPTLVAIRYNPVLKAFYERLVGQGK---PKKVALTACMRK
                     LLIILNAMVKNNQQWNPEMARGAX"
                     /note="IS110 e-value= 2.2e-68 complete sequence hit
                     coverage= 100%, between model( 416 aa) positions 1; 416
                     length is 312aa with 104 gaps, 1 stops, absolute frame=
                     Minus2"
     CDS             complement(6013789..6013572)
                     /colour="255 0 0"
                     /evidence=predicted
                     /translation="RIRFVYIPKHTSWLNQIECWFSILVRRLIRRGNFTSKDDLQQRI
                     LEFIEYFNHtMAKPFQWQFKGFQPRLXLM"
                     /note="IS630 e-value= 4e-10 fragment hit coverage=
```

```
                 19.15%, between model( 376 aa) positions 305; 376 length
                 is 73aa with 0 gaps, 1 stops, absolute frame= Minus2"
CDS              complement(6013741..6013632)
                 /colour="255 0 0"
                 /evidence=predicted
                 /translation="IECWFSILVRRLIR--RGNFTSKDDLQQRILEFIEYFNH"
                 /note="IS3_IS150_ORF2    e-value= 2e-08 fragment hit
                 coverage= 10.03%, between model( 389 aa) positions 329;
                 367 length is 37aa with 2 gaps, 0 stops, absolute frame=
                 Minus2"
CDS              complement(5927854..5927166)
                 /colour="255 0 0"
                 /evidence=predicted
                 /translation="TDLFKWRHYQFEIILLNVRWYCRYTLSYRNLEEMMG-RGFKVDH
                 ITIHRWVFKYSSELDKR-CRHLKP-THASSRVDETYVKIHXQWRYLYRAVDSE-SYTL
                 DFLLTAKRDTKAAKRFLTKALNTXDSWVXISSIQCGRHPAYPPAVEQLQADERIPEKE
                 KVRQVKYLNNRVEQDRIGIKKLVNPGMGFGSCNSARRTLKGFESMNMICKVQIKNVGK
                 DDVIG-QIYFINQIFGL"
                 /note="IS6   e-value= 1.5e-77 complete sequence hit
                 coverage= 100%, between model( 235 aa) positions 1; 235
                 length is 230aa with 5 gaps, 3 stops, absolute frame=
                 Minus2"
CDS              complement(5927854..5927166)
                 /colour="255 0 0"
                 /evidence=predicted
                 /translation="TDLFKWRHYQFEIILLNVRWYCRYTLSYRNLEEMMG-RGFKVDH
                 ITIHRWVFKYSSELDKR-CRHLKP-THASSRVDETYVKIHXQWRYLYRAVDSE-SYTL
                 DFLLTAKRDTKAAKRFLTKALNTXDSWVXISSIQCGRHPAYPPAVEQLQADERIPEKE
                 KVRQVKYLNNRVEQDRIGIKKLVNPGMGFGSCNSARRTLKGFESMNMICKVQIKNVGK
                 DDVIG-QIYFINQIFGL"
                 /note="IS6   e-value= 6.2e-80 complete sequence hit
                 coverage= 100%, between model( 235 aa) positions 1; 235
                 length is 230aa with 5 gaps, 3 stops, absolute frame=
                 Minus2"
CDS              complement(5746405..5745369)
                 /colour="255 0 0"
                 /evidence=predicted
                 /translation="CMIKIDFTAEEIQHLNYERYHHPHPRVQRRMEVLYLKSQGLPHA
                 QICQLCQISRPTLAKTLRLYQQGG---IEGLKTLEYKGQSSSLNAHS-DSVRAYFEQH
                 PP---------RTSAEAQAVIEQLTGIKRSPTQIKAFLKRIGCRSRKVGYVPGKSSLP
                 -EKIEEQEQFRQTRLEPLleeAQRQERLVFFVDAAHFVHRAYLGF-LWCLRRI-----
                 ---------FIPSPS-GRQRFNVLGALNAVTKEvTSITNHTYINSHSMCLLLAKLALL
                 DPVI--PISVILDNARYQKCQLVTDFAEIVD--IELVYLPSYSPHLNLIERLWRFVRK
                 ECLYSKYYSDFHSFKGAIQQCIDQCNTEHKAKLTSLLSLKFQSFQK"
                 /note="IS630  e-value= 4e-22 complete sequence hit
                 coverage= 100%, between model( 376 aa) positions 1; 376
                 length is 346aa with 34 gaps, 0 stops, absolute frame=
                 Minus2"
CDS              complement(5746345..5746188)
                 /colour="255 0 0"
                 /evidence=predicted
                 /translation="HHPHPRVQRRMEVLYLKSQGLPHAQICQLCQISRPTLAKTLRLY
                 QQGGIEGLK"
                 /note="IS481  e-value= 1.1e-08 fragment hit coverage=
                 15.10%, between model( 351 aa) positions 1; 53 length is
                 53aa with 0 gaps, 0 stops, absolute frame= Minus2"
CDS              complement(5746339..5745369)
                 /colour="255 0 0"
                 /evidence=predicted
                 /translation="PHPRVQRRMEVLYLKSQGLPHAQICQLCQISRPTLAKTLRLYQQ
```

```
                     GG---IEGLKTLEYKGQSSSLNAHS-DSVRAYFEQHPP---------RTSAEAQAVIE
                     QLTGIKRSPTQIKAFLKRIGCRSRKVGYVPGKSSLP-EKIEEQEQFRQTRLEPLleeA
                     QRQERLVFFVDAAHFVHRAYLGF-LWCLRRI--------------FIPSPS-GRQRFN
                     VLGALNAVTKEvTSITNHTYINSHSMCLLLAKLALLDPVI--PISVILDNARYQKCQL
                     VTDFAEIVD--IELVYLPSYSPHLNLIERLWRFVRKECLYSKYYSDFHSFKGAIQQCI
                     DQCNTEHKAKLTSLLSLKFQSFQK"
                     /note="IS630  e-value= 9.5e-24 fragment hit coverage=
                     94.15%, between model( 376 aa) positions 23; 376 length
                     is 324aa with 34 gaps, 0 stops, absolute frame= Minus2"
     CDS             complement(5716993..5716776)
                     /colour="255 0 0"
                     /evidence=predicted
                     /translation="RIRFVYIPKHTSWLNQIECWFSILVRRLIRRGNFTSKDDLQQRI
                     LEFIEYFNHtMAKPFQWQFKGFQPRLXLM"
                     /note="IS630  e-value= 4e-10 fragment hit coverage=
                     19.15%, between model( 376 aa) positions 305; 376 length
                     is 73aa with 0 gaps, 1 stops, absolute frame= Minus2"
     CDS             complement(5716945..5716836)
                     /colour="255 0 0"
                     /evidence=predicted
                     /translation="IECWFSILVRRLIR--RGNFTSKDDLQQRILEFIEYFNH"
                     /note="IS3_IS150_ORF2    e-value= 2e-08 fragment hit
                     coverage= 10.03%, between model( 389 aa) positions 329;
                     367 length is 37aa with 2 gaps, 0 stops, absolute frame=
                     Minus2"
     CDS             complement(5652220..5652039)
                     /colour="255 0 0"
                     /evidence=predicted
                     /translation="AVAESFFGTLKTELIYSTTFANRA--MAKTAIAEWIEVFYNRQR
                     LHSTIGHCAPVQFEENYWR"
                     /note="IS3_IS150_ORF1    e-value= 1.1e-07 fragment hit
                     coverage= 20.59%, between model( 306 aa) positions 244;
                     306 length is 61aa with 2 gaps, 0 stops, absolute frame=
                     Minus2"
     CDS             complement(5652220..5652060)
                     /colour="255 0 0"
                     /evidence=predicted
                     /translation="AVAESFFGTLKTELIY--STTFANRAMAKTAIAEWIEvFYNRQR
                     LHSTIGHCAPVQ"
                     /note="IS3_IS150_ORF2    e-value= 3.7e-12 fragment hit
                     coverage= 14.14%, between model( 389 aa) positions 327;
                     381 length is 54aa with 2 gaps, 0 stops, absolute frame=
                     Minus2"
     CDS             complement(5652220..5652042)
                     /colour="255 0 0"
                     /evidence=predicted
                     /translation="AVAESFFGTLK--TELIYSTTFANRAMAKTAIAEWIEVFYNRQR
                     LHSTIGHCAPVQFEENYW"
                     /note="IS3_IS3_ORF1      e-value= 3.3e-23 fragment hit
                     coverage= 21.68%, between model( 286 aa) positions 225;
                     286 length is 60aa with 2 gaps, 0 stops, absolute frame=
                     Minus2"
     CDS             complement(5652220..5652030)
                     /colour="255 0 0"
                     /evidence=predicted
                     /translation="AVAESFFGTLKTELIYSTTFANRAMAKTAIAEWIEVFYNRQRLH
                     STIGHCAPVQFEENYWRTRH"
                     /note="IS3_IS3_ORF2      e-value= 1.9e-21 fragment hit
                     coverage= 22.22%, between model( 288 aa) positions 225;
                     288 length is 64aa with 0 gaps, 0 stops, absolute frame=
```

```
                     Minus2"
     CDS             complement(5652220..5652012)
                     /colour="255 0 0"
                     /evidence=predicted
                     /translation="AVAESFFGTLKTELIYS----TTFANRAMAKTAIAEWIEVFYNR
                     QRLHSTIGHCAPVQFEENYWRTRHQTLVAX"
                     /note="IS3_IS51_ORF1     e-value= 1.5e-22 fragment hit
                     coverage= 24.10%, between model( 307 aa) positions 234;
                     307 length is 70aa with 4 gaps, 1 stops, absolute frame=
                     Minus2"
     CDS             complement(5652220..5652030)
                     /colour="255 0 0"
                     /evidence=predicted
                     /translation="AVAESFFGTLKTELIYSTTFANRAMAKTAIAEWIEVFYNRQRLH
                     STIGHCAPVQFEENYW-RTRH"
                     /note="IS3_IS51_ORF2     e-value= 2.3e-14 fragment hit
                     coverage= 20.50%, between model( 317 aa) positions 245;
                     309 length is 64aa with 1 gaps, 0 stops, absolute frame=
                     Minus2"
     CDS             complement(5522092..5521956)
                     /colour="255 0 0"
                     /evidence=predicted
                     /translation="WQTDGWEGYSRQLADE---VIHHVSKALTQRLERTNGILRQQTG
                     RWHRR"
                     /note="IS1_ORF2    e-value= 1.6e-12 fragment hit coverage=
                     20.68%, between model( 237 aa) positions 164; 212 length
                     is 46aa with 3 gaps, 0 stops, absolute frame= Minus2"
     CDS             complement(5510998..5509761)
                     /colour="255 0 0"
                     /evidence=predicted
                     /translation="IRIPLNLPDI--RVLELSKTERGDWL------IKIESTLQGTTC
                     HQCGREITDL-HCHDQ-PFRIRHLPLFEVPVYLEIRPKRYRCKYCDDhpTTTQHLEWH
                     EPRSPNTKAYERWLLRILIN-STVSDVARKLGVSEDIVSGIIDRWIAAQVDWSEYADL
                     KVMGMDEISLKRG--HR---DFVVLITIPTTdgVDILAVLADRKQQTVANFLQSIP-I
                     DLRQTIERVCTDMYQGFVSAVREQLPQAKIVIDRFHVTKAYRNCADTVRKREVKRLRR
                     ELS------------KQEYD-----------SIKGAMWPFRKRPENLKES-EQQLLER
                     LFA--YSPQLKQAYNLREKLTQIFEGRYTKHGAKcAIRAWCKQVLKSDiKEFDCFLTT
                     INNWMDEMTNYFLEG-WTSGFVEGFNNRVKVLKRRCYGIFDIERLFQRISLDLNGYQT
                     LVVHQRS"
                     /note="ISL3  e-value= 4.5e-44 complete sequence hit
                     coverage= 100%, between model( 451 aa) positions 1; 451
                     length is 413aa with 44 gaps, 0 stops, absolute frame=
                     Minus2"
     CDS             complement(5510839..5509761)
                     /colour="255 0 0"
                     /evidence=predicted
                     /translation="RIRHLPLFEVPVYLEIRPKRYRCKYCDDhpTTTQHLEWHEPRSP
                     NTKAYERWLLRILIN-STVSDVARKLGVSEDIVSGIIDRWIAAQVDWSEYADLKVMGM
                     DEISLKRG--HR---DFVVLITIPTTdgVDILAVLADRKQQTVANFLQSIP-IDLRQT
                     IERVCTDMYQGFVSAVREQLPQAKIVIDRFHVTKAYRNCADTVRKREVKRLRRELS--
                     ----------KQEYD-----------SIKGAMWPFRKRPENLKES-EQQLLERLFA--
                     YSPQLKQAYNLREKLTQIFEGRYTKHGAKcAIRAWCKQVLKSDiKEFDCFLTTINNWM
                     DEMTNYFLEG-WTSGFVEGFNNRVKVLKRRCYGIFDIERLFQRISLDLNGYQTLVVHQ
                     RS"
                     /note="ISL3  e-value= 2.6e-50 fragment hit coverage=
                     86.03%, between model( 451 aa) positions 64; 451 length
                     is 360aa with 34 gaps, 0 stops, absolute frame= Minus2"
     CDS             complement(5509729..5509467)
                     /colour="255 0 0"
                     /evidence=predicted
```

```
                     /translation="LFYFLMQCPLCGHPK-THKHGKTSKGSQRYRCPHCQQTFS-Etf
                     DTLCYRRQiSPETIQTILQAHVEGSSLRGLSRITGVAYNTCVSVVR"
                     /note="IS1_ORF1    e-value= 1.7e-15 fragment hit coverage=
                     36.40%, between model( 239 aa) positions 1; 87 length is
                     88aa with 2 gaps, 0 stops, absolute frame= Minus2"
     CDS             complement(5475031..5474574)
                     /colour="255 0 0"
                     /evidence=predicted
                     /translation="MTYRKLSHSVWECKYHVVFVPKYRKRRIYGRIRQNLGDIFH-EL
                     ARHKESAIEEGHLMLDHVHMLLSIPPKYSVSNVVGYVKGKSAIYIARELGNSRNA-TG
                     HKFWSRGYFVSTVG-RDEEVIRRYIRHQEQEEQKLEQLNLFraXPKSPLRGSNS"
                     /note="IS200  e-value= 9.6e-56 complete sequence hit
                     coverage= 100%, between model( 154 aa) positions 1; 154
                     length is 153aa with 3 gaps, 1 stops, absolute frame=
                     Minus2"
     CDS             complement(5475031..5474574)
                     /colour="255 0 0"
                     /evidence=predicted
                     /translation="MTYRKLSHSVWECKYHVVFVPKYRKRRIYGRIRQNLGDIFH-EL
                     ARHKESAIEEGHLMLDHVHMLLSIPPKYSVSNVVGYVKGKSAIYIARELGNSRNA-TG
                     HKFWSRGYFVSTVG-RDEEVIRRYIRHQEQEEQKLEQLNLFraXPKSPLRGSNS"
                     /note="IS200  e-value= 5.7e-55 complete sequence hit
                     coverage= 100%, between model( 154 aa) positions 1; 154
                     length is 153aa with 3 gaps, 1 stops, absolute frame=
                     Minus2"
     CDS             complement(5475031..5474577)
                     /colour="255 0 0"
                     /evidence=predicted
                     /translation="MTYRKLSHSVWECKYHVVFVPKYRKRRIYGR-IRQNLGDIFHEL
                     ARHKESAIEEGHLMLDHVHMLLSIPPKYSVSNVVGYVKGKSAIYIARELGNSRNA-TG
                     HKFWSRGYFVSTVG-RDEEVIRRYIRHQEQEEQKLEQLNLFraXPKSPLRGSN"
                     /note="IS200_IS605 e-value= 8.6e-58 complete sequence hit
                     coverage= 100%, between model( 153 aa) positions 1; 153
                     length is 152aa with 3 gaps, 1 stops, absolute frame=
                     Minus2"
     CDS             complement(5475031..5474577)
                     /colour="255 0 0"
                     /evidence=predicted
                     /translation="MTYRKLSHSVWECKYHVVFVPKYRKRRIYGR-IRQNLGDIFHEL
                     ARHKESAIEEGHLMLDHVHMLLSIPPKYSVSNVVGYVKGKSAIYIARELGNSRNA-TG
                     HKFWSRGYFVSTVG-RDEEVIRRYIRHQEQEEQKLEQLNLFraXPKSPLRGSN"
                     /note="IS200_IS605 e-value= 2.2e-58 complete sequence hit
                     coverage= 100%, between model( 153 aa) positions 1; 153
                     length is 152aa with 3 gaps, 1 stops, absolute frame=
                     Minus2"
     CDS             complement(5475031..5474577)
                     /colour="255 0 0"
                     /evidence=predicted
                     /translation="-MTYRKLSHSVWECKYHVVFVPKYRKRRIYGRIRQNLGDIFHEL
                     ARHKESAIEEGHLMLDHVHMLLSIPPKYSVSNVVGYVKGKSAIYIARELGNSRN-ATG
                     HKFWSRGYFVSTVGRDEEVIRRYIRHQEQEEQKLEQLNLfrAXPKSPLRGSN"
                     /note="IS605  e-value= 1e-51 complete sequence hit
                     coverage= 100%, between model( 152 aa) positions 1; 152
                     length is 152aa with 2 gaps, 1 stops, absolute frame=
                     Minus2"
     CDS             complement(5475031..5474577)
                     /colour="255 0 0"
                     /evidence=predicted
                     /translation="-MTYRKLSHSVWECKYHVVFVPKYRKRRIYGRIRQNLGDIFHEL
                     ARHKESAIEEGHLMLDHVHMLLSIPPKYSVSNVVGYVKGKSAIYIARELGNSRN-ATG
```

```
                     HKFWSRGYFVSTVGRDEEVIRRYIRHQEQEEQKLEQLNLfrAXPKSPLRGSN"
                     /note="IS605 e-value= 5.5e-52 complete sequence hit
                     coverage= 100%, between model( 152 aa) positions 1; 152
                     length is 152aa with 2 gaps, 1 stops, absolute frame=
                     Minus2"
     CDS             complement(5354932..5354196)
                     /colour="255 0 0"
                     /evidence=predicted
                     /translation="-----TGVYKRTFkpMLHAWHTYHLSRSN--AGRPPKLCRCDQL
                     LVALQYWREYRTYFHIAGDWEVSESTVCRIVHQVETALMNSGLFRLPGQKSLLQGFER
                     PDVVVMDVTETPIERPQTRQKAYYSGKKRDIPSNARLSLTATLXRLSALT--------
                     --LVQVVGMIFrsprfqvSISIQIPRVCKIADIKGLQ-----PIMP-TAMFLSRSHNT
                     VNXLPCSESITVLXVRNEWALNTLIAAXRFSEFCRSAIAIVVVATRCGVTX"
                     /note="IS5_ISL2    e-value= 9.6e-21 complete sequence hit
                     coverage= 100%, between model( 260 aa) positions 1; 260
                     length is 246aa with 23 gaps, 5 stops, absolute frame=
                     Minus2"
     CDS             complement(5354872..5354562)
                     /colour="255 0 0"
                     /evidence=predicted
                     /translation="SRSNAGRPPKLCRCDQLLVALQYWREYRTYFHIAGDWEVSESTV
                     CRIVHQVETALMNSGLFRLPGQKSLLQGFERPDVVVMDVTETPIERPQTRQKAYYSGK
                     KR"
                     /note="IS5_ISL2    e-value= 9.3e-44 fragment hit coverage=
                     40%, between model( 260 aa) positions 26; 129 length is
                     104aa with 0 gaps, 0 stops, absolute frame= Minus2"
     CDS             complement(5338738..5338023)
                     /colour="255 0 0"
                     /evidence=predicted
                     /translation="YLSPES-LQCRASSKVVSMXSTVGSPS---------ILARISDL
                     FPHCWRLGSLXINCLPYCppGRDRSDELRTLSLTRSEISATR---------VRATGCR
                     CDGCDXNAHXTSPDKTKSVLF----RKKKRHTFKCQIIANRNTLEIICLNVGPGRRHD
                     FQIFKGSGIHIHPDTESLQDSGYQGIAAYHANSYVPLKKPQHGELTSLQREYNRALSQ
                     ERMGIEHINRSLKIFRILSERYRNRRRRYALRCNLIAAIYNYEL"
                     /note="IS5_ISL2    e-value= 6.9e-39 complete sequence hit
                     coverage= 100%, between model( 260 aa) positions 1; 260
                     length is 239aa with 23 gaps, 4 stops, absolute frame=
                     Minus2"
     CDS             complement(5338423..5338023)
                     /colour="255 0 0"
                     /evidence=predicted
                     /translation="KKKRHTFKCQIIANRNTLEIICLNVGPGRRHDFQIFKGSGIHIH
                     PDTESLQDSGYQGIAAYHANSYVPLKKPQHGELTSLQREYNRALSQERMGIEHINRSL
                     KIFRILSERYRNRRRRYALRCNLIAAIYNYEL"
                     /note="IS5_ISL2    e-value= 6.5e-62 fragment hit coverage=
                     51.54%, between model( 260 aa) positions 127; 260 length
                     is 134aa with 0 gaps, 0 stops, absolute frame= Minus2"
     CDS             complement(4930255..4929975)
                     /colour="255 0 0"
                     /evidence=predicted
                     /translation="SMECPYCQSEK-ILKRGFDSlqdgTLVQRYQCKDCNRRFN-ERT
                     GTPMARLRtasSVVSYAIKARTEGMGVRSAGRTFGKSHTTIMRWEKRLADQA"
                     /note="IS1_ORF1    e-value= 3.2e-11 fragment hit coverage=
                     37.24%, between model( 239 aa) positions 5; 93 length is
                     94aa with 2 gaps, 0 stops, absolute frame= Minus2"
     CDS             complement(4612015..4611591)
                     /colour="255 0 0"
                     /evidence=predicted
                     /translation="GRQRFNVLGALNAVTKEvTSITNHTYINSHSMCLLLAKLALLDP
                     VI--PISVILDNARYQKCQLVTDFAEIVD--IELVYLPSYSPHLNLIERLWRFVRKEC
```

```
                     LYSKYYSDFHSFKGAIQQCIDQCNTEHKAKLTSLLSLKFQSFQK"
                     /note="IS630 e-value= 6.2e-18 fragment hit coverage=
                     38.56%, between model( 376 aa) positions 232; 376 length
                     is 142aa with 4 gaps, 0 stops, absolute frame= Minus2"
     CDS             complement(4461124..4460748)
                     /colour="255 0 0"
                     /evidence=predicted
                     /translation="EHRKSEDFALCMQYLVDGLFPQADKLHVVLDNLNTHspaalyKT
                     FNPDEALRILK-RIQFHYTPKHGSWLNMIEFEFSALSRQCLNRRIP-DIEQLRYEVTA
                     WEKRRNHDKATVNWLFTVDDARTKLS"
                     /note="IS630 e-value= 2.3e-07 fragment hit coverage=
                     32.45%, between model( 376 aa) positions 255; 376 length
                     is 126aa with 2 gaps, 0 stops, absolute frame= Minus2"
     CDS             complement(4320520..4320258)
                     /colour="255 0 0"
                     /evidence=predicted
                     /translation="LFYFLMQCPLCGHPK-THKHGKTSKGSQRYRCPHCQQTFS-Etf
                     DTLCYRRQiSPETIQTILQAHVEGSSLRGLSRITGVAYNTCVSVVR"
                     /note="IS1_ORF1    e-value= 1.7e-15 fragment hit coverage=
                     36.40%, between model( 239 aa) positions 1; 87 length is
                     88aa with 2 gaps, 0 stops, absolute frame= Minus2"
     CDS             complement(4297825..4296789)
                     /colour="255 0 0"
                     /evidence=predicted
                     /translation="PMIKIDFTAEEIQQLNYERYHHPHPRVQRRMEVLYLKSQGLSHT
                     QICQLCQISRPTLAKTLRQYQQGG---IEGLKTLEYKGQPSLLNAHS-DSVRAYFEQH
                     PP---------RTSAEAQAVIEQLTGIKRSPTQIKAFLKRIGCRYRKVGYVPGKSSLP
                     -EKIEEQEQFRHTRLEPLleeAQRQERLVFFVDAAHFVHRAYLGF-VWCITRI-----
                     ---------FIPSPS-GRQRFNVLGALDAITKDLVSVTNHTYINSHSMCLLLAKLALL
                     DPV-IPISVILDNARYQKCQLVTDFAEIVD--IELVYLPSYSPHLNLIERLWRFVRKE
                     CLYSKYYADFPAFKGAIQQCIDQCNGEHKAKLTTLLSLKFQSFKK"
                     /note="IS630 e-value= 6.7e-25 complete sequence hit
                     coverage= 100%, between model( 376 aa) positions 1; 376
                     length is 346aa with 33 gaps, 0 stops, absolute frame=
                     Minus2"
     CDS             complement(4297825..4296789)
                     /colour="255 0 0"
                     /evidence=predicted
                     /translation="PMIKIDFTAEEIQQLNYERYHHPHPRVQRRMEVLYLKSQGLSHT
                     QICQLCQISRPTLAKTLRQYQQGG---IEGLKTLEYKGQPSLLNAHS-DSVRAYFEQH
                     PP---------RTSAEAQAVIEQLTGIKRSPTQIKAFLKRIGCRYRKVGYVPGKSSLP
                     -EKIEEQEQFRHTRLEPLleeAQRQERLVFFVDAAHFVHRAYLGF-VWCITRI-----
                     ---------FIPSPS-GRQRFNVLGALDAITKDLVSVTNHTYINSHSMCLLLAKLALL
                     DPV-IPISVILDNARYQKCQLVTDFAEIVD--IELVYLPSYSPHLNLIERLWRFVRKE
                     CLYSKYYADFPAFKGAIQQCIDQCNGEHKAKLTTLLSLKFQSFKK"
                     /note="IS630 e-value= 4.9e-24 complete sequence hit
                     coverage= 100%, between model( 376 aa) positions 1; 376
                     length is 346aa with 33 gaps, 0 stops, absolute frame=
                     Minus2"
     CDS             complement(4297765..4297608)
                     /colour="255 0 0"
                     /evidence=predicted
                     /translation="HHPHPRVQRRMEVLYLKSQGLSHTQICQLCQISRPTLAKTLRQY
                     QQGGIEGLK"
                     /note="IS481 e-value= 3.7e-09 fragment hit coverage=
                     15.10%, between model( 351 aa) positions 1; 53 length is
                     53aa with 0 gaps, 0 stops, absolute frame= Minus2"
     CDS             complement(4135960..4135503)
                     /colour="255 0 0"
                     /evidence=predicted
```

```
                     /translation="MTYRKLSHSVWECKYHVVFVPKYRKRRIYGRIRQNLGDIFH-EL
                     ARHKESAIEEGHLMLDHVHMLLSIPPKYSVSNVVGYVKGKSAIYIARELGNSRNA-TG
                     HKFWSRGYFVSTVG-RDEEVIRRYIRHQEQEEQKLEQLNLFraXPKSPLRGSNS"
                     /note="IS200 e-value= 9.6e-56 complete sequence hit
                     coverage= 100%, between model( 154 aa) positions 1; 154
                     length is 153aa with 3 gaps, 1 stops, absolute frame=
                     Minus2"
     CDS             complement(4135960..4135503)
                     /colour="255 0 0"
                     /evidence=predicted
                     /translation="MTYRKLSHSVWECKYHVVFVPKYRKRRIYGRIRQNLGDIFH-EL
                     ARHKESAIEEGHLMLDHVHMLLSIPPKYSVSNVVGYVKGKSAIYIARELGNSRNA-TG
                     HKFWSRGYFVSTVG-RDEEVIRRYIRHQEQEEQKLEQLNLFraXPKSPLRGSNS"
                     /note="IS200 e-value= 5.7e-55 complete sequence hit
                     coverage= 100%, between model( 154 aa) positions 1; 154
                     length is 153aa with 3 gaps, 1 stops, absolute frame=
                     Minus2"
     CDS             complement(4135960..4135506)
                     /colour="255 0 0"
                     /evidence=predicted
                     /translation="MTYRKLSHSVWECKYHVVFVPKYRKRRIYGR-IRQNLGDIFHEL
                     ARHKESAIEEGHLMLDHVHMLLSIPPKYSVSNVVGYVKGKSAIYIARELGNSRNA-TG
                     HKFWSRGYFVSTVG-RDEEVIRRYIRHQEQEEQKLEQLNLFraXPKSPLRGSN"
                     /note="IS200_IS605 e-value= 8.6e-58 complete sequence hit
                     coverage= 100%, between model( 153 aa) positions 1; 153
                     length is 152aa with 3 gaps, 1 stops, absolute frame=
                     Minus2"
     CDS             complement(4135960..4135506)
                     /colour="255 0 0"
                     /evidence=predicted
                     /translation="MTYRKLSHSVWECKYHVVFVPKYRKRRIYGR-IRQNLGDIFHEL
                     ARHKESAIEEGHLMLDHVHMLLSIPPKYSVSNVVGYVKGKSAIYIARELGNSRNA-TG
                     HKFWSRGYFVSTVG-RDEEVIRRYIRHQEQEEQKLEQLNLFraXPKSPLRGSN"
                     /note="IS200_IS605 e-value= 2.2e-58 complete sequence hit
                     coverage= 100%, between model( 153 aa) positions 1; 153
                     length is 152aa with 3 gaps, 1 stops, absolute frame=
                     Minus2"
     CDS             complement(4135960..4135506)
                     /colour="255 0 0"
                     /evidence=predicted
                     /translation="-MTYRKLSHSVWECKYHVVFVPKYRKRRIYGRIRQNLGDIFHEL
                     ARHKESAIEEGHLMLDHVHMLLSIPPKYSVSNVVGYVKGKSAIYIARELGNSRN-ATG
                     HKFWSRGYFVSTVGRDEEVIRRYIRHQEQEEQKLEQLNLfrAXPKSPLRGSN"
                     /note="IS605 e-value= 1e-51 complete sequence hit
                     coverage= 100%, between model( 152 aa) positions 1; 152
                     length is 152aa with 2 gaps, 1 stops, absolute frame=
                     Minus2"
     CDS             complement(4135960..4135506)
                     /colour="255 0 0"
                     /evidence=predicted
                     /translation="-MTYRKLSHSVWECKYHVVFVPKYRKRRIYGRIRQNLGDIFHEL
                     ARHKESAIEEGHLMLDHVHMLLSIPPKYSVSNVVGYVKGKSAIYIARELGNSRN-ATG
                     HKFWSRGYFVSTVGRDEEVIRRYIRHQEQEEQKLEQLNLfrAXPKSPLRGSN"
                     /note="IS605 e-value= 5.5e-52 complete sequence hit
                     coverage= 100%, between model( 152 aa) positions 1; 152
                     length is 152aa with 2 gaps, 1 stops, absolute frame=
                     Minus2"
     CDS             complement(3914230..3913173)
                     /colour="255 0 0"
                     /evidence=predicted
```

```
                     /translation="ACSRCRHTFT-DXSSQSEDIEGSQKPHGCMGSESSCTT------
                     ---WRISGASSQH---TDGDSSALE---RMGXVKSPSRGLAVSDPgDLAGKVRVRQ-L
                     QQDYSIRQICQVLNYPRSQVYYHARGQP-----DESELKAAIAGVAGAYP-TYGYRRI
                     TAQLQ---RQGYCVNHKRVARLMRQIGIMAKTKVK-RKRTTNSEHSfpRYGNRVLNLS
                     --IDHPEQVWVADITYIRLQ-QEFVYLAVVMDVFTRAIRGWHLSRHIDQQLTLRALNK
                     ALERAT---PEIHHS-----DQGVQYAAAAYMQLLQQHQVQISMAEVGQAWQNGYAER
                     LMRTIKEEEVD--LSDYRNFTEAYEHIEQFLEdVYMHKRIHSSLGYLTPCEYEQQWRQ
                     Q"
                     /note="IS3_IS150_ORF2    e-value= 5e-28 complete sequence
                     hit coverage= 100%, between model( 389 aa) positions 1;
                     389 length is 353aa with 40 gaps, 2 stops, absolute
                     frame= Minus2"
     CDS             complement(3914146..3913149)
                     /colour="255 0 0"
                     /evidence=predicted
                     /translation="MGSESSCTTWRISGASSQHTDGDS--SALERMGXVK-----SPS
                     RGLAVSDPGDLAGKVRVRQLQQ-----------DYSIRQICQVLNYPRSQVYYHARGQ
                     PDESELKAAIAGVAGAYPTYGYRRITAQLQRQGYCVNHKRVARLMRQIGIMAKTKV-K
                     RKRTtnsehsfprYGNRVLNLSIDHPEQVWVADITYIRLQ--QEFVYLAVVmDVFTRA
                     IRGWHLSRHIDQQLTLRALNKALE-RATPEIHHSDQGVQYAAAAYMQLLQQHQVQISM
                     AEVGQAWQNGYAERLMRTIKEEEVDLSDYRNFTEAYEHIEQFLEDvYMHKRIHSSLGY
                     LTPCEYEQQWRQQNNHYCMNK"
                     /note="IS3_IS407_ORF2    e-value= 1.2e-11 complete sequence
                     hit coverage= 100%, between model( 344 aa) positions 1;
                     344 length is 333aa with 22 gaps, 1 stops, absolute
                     frame= Minus2"
     CDS             complement(3914050..3913176)
                     /colour="255 0 0"
                     /evidence=predicted
                     /translation="VKSPSRGLAVSDPgdLAGKVR----VRQLQQDYSIRQICQVLNY
                     PRSQVYYHARGQPDESELKAAIAGV-AGAYPTYGYRRITAQLQRQGYCVNHKRVARLM
                     RQIGIMAKTKVK----RKRTTNSEHSF-PRYGNRvlNLSIDHPEQVWVADITYIRL--
                     QQEFVYLAVVMDVFTRAIRGWHLSRHIDQQLTLRALNKALER----ATPEIHHSDQGV
                     QYAAAAYMQLLQQHQVQISMAEVGQAWQNGYAERLMRTIKEEEVDLSDYRNFT--EAY
                     EHIEQFLEDVYMHKRIHSSLGYLTPCEYEQQWRQ"
                     /note="IS3_IS150_ORF1    e-value= 2.2e-46 complete sequence
                     hit coverage= 100%, between model( 306 aa) positions 1;
                     306 length is 292aa with 18 gaps, 0 stops, absolute
                     frame= Minus2"
     CDS             complement(3914026..3913140)
                     /colour="255 0 0"
                     /evidence=predicted
                     /translation="AVSDPGDLAGKVRVRQLQQDYSIRQICQVLNYPRSQVYYHARGQ
                     PD--------------------ESELKAAIAGVAGAYPTYGYRRITAQLQRQGYC---
                     VNHKRVARLMRQIGIMAKTKVKRKR-TTNSEHSFPRYGnRVLNLSIDHPEQVWVADIT
                     YI-------------RLQQ--------EFVYLAVVMDVFTRAIRGWHLSRH----IDQ
                     QLTLRALNKALERAT-PEIHHSDQGVQYAAA----AYMQLLQQHQVQISMAEVGQAWQ
                     NGYAERLMRTIKEEEVDLSD---YRNFTEAYEHIEQFLEDvYMHKRIHSSLGYLTPCE
                     YEQQWRQQNNHYCMNKEDS"
                     /note="IS481 e-value= 2.7e-07 complete sequence hit
                     coverage= 100%, between model( 351 aa) positions 1; 351
                     length is 296aa with 57 gaps, 0 stops, absolute frame=
                     Minus2"
     CDS             complement(3914008..3913140)
                     /colour="255 0 0"
                     /evidence=predicted
                     /translation="----DLAGKVRVRQLQQDYSIRQICQVLNYP---RSQVYYHARg
                     QPDESELKA--AIAGV-------AGA---YPTYGYRRITAQLQRQGYCVNHKRVARLM
                     RQIGIMAKTKVKRKRTTNSEHSFPRYGNRVL-NLSIDHPEQVWVADITYIRLQQEFVY
                     LAVVMDVFTRAIRGWHLSRHIDQQLTLRALNKALERATPE--------IHHSDQGVQY
```

```
                     AAAAYMQLLQQHQVQISMAEVGQAWQNGYAERLMRTIKEEEVDLSDYrnFTEAYEHIE
                     QFLEDV--YMHKRIHSSLGYLTPCEYEQQWRQQNNHYCMNKEDS"
                     /note="IS3_IS51_ORF2    e-value= 3.7e-25 complete sequence
                     hit coverage= 100%, between model( 317 aa) positions 1;
                     317 length is 290aa with 30 gaps, 0 stops, absolute
                     frame= Minus2"
     CDS             complement(3914005..3913164)
                     /colour="255 0 0"
                     /evidence=predicted
                     /translation="-LAGKV--RVRQL-QQDYSIRQICQVLNYPRSQVYYHARG----
                     -------QPDESELKAAIAGVAGAYPTYGYRRITAQLQRQGY-----CVNHKRVARLM
                     RQIGIM--AKTKVKRK-RttnsehsfpRYGNRVLnlsIDHPEQVWVAD-ITYIRLQQE
                     FVYLAVVMDVFTRAIRGWH------LSRHIDQQLTLRALNK---ALERATPEIHH-SD
                     QGVQYAAAAYMQLLQQHQVQISMAEVGQAWQNGYAERLMRTIKEEEVDLSDYRNFTEA
                     YEHIEQFLEDvYMHKRIHSSLGYLTPCEYEQQWRQQNNH"
                     /note="IS3_IS2_ORF2     e-value= 3.7e-08 complete sequence
                     hit coverage= 100%, between model( 302 aa) positions 1;
                     302 length is 281aa with 34 gaps, 0 stops, absolute
                     frame= Minus2"
     CDS             complement(3913996..3913149)
                     /colour="255 0 0"
                     /evidence=predicted
                     /translation="KVRVRQLQQDYSIRQICQVLNYPRSQVYYHA-----------RG
                     QPDEsELKAAIAGV-AGAYPTYGYRRITAQLQRQGYCVNHKRVARLMRQIGIMAKTKV
                     KRKRTTNSEHSFPRYGNRVL-NLSIDHPEQVWVADITYIRLQQEFVYLAVVMDVFTRA
                     IRGWHLSRHIDQQLT-LRALNKALERATPE-------IHHSDQGVQYAAAAYMQLLQQ
                     HQVQISMAEVGQAWQNGYAERLMRTIKEEEVDL---S-DYRNFTEAYEHIEQFLEDVY
                     MHKRIHSSLGYLTPCEYEQQWRQQNNHYCMNK"
                     /note="IS3_IS51_ORF1    e-value= 3e-51 complete sequence
hit
                     coverage= 100%, between model( 307 aa) positions 1; 307
                     length is 283aa with 25 gaps, 0 stops, absolute frame=
                     Minus2"
     CDS             complement(3913996..3913149)
                     /colour="255 0 0"
                     /evidence=predicted
                     /translation="KVRVRQLQQDYSIRQICQVLNYPRSQVYYHA-----------RG
                     QPDEsELKAAIAGV-AGAYPTYGYRRITAQLQRQGYCVNHKRVARLMRQIGIMAKTKV
                     KRKRTTNSEHSFPRYGNRVL-NLSIDHPEQVWVADITYIRLQQEFVYLAVVMDVFTRA
                     IRGWHLSRHIDQQLT-LRALNKALERATPE-------IHHSDQGVQYAAAAYMQLLQQ
                     HQVQISMAEVGQAWQNGYAERLMRTIKEEEVDL---S-DYRNFTEAYEHIEQFLEDVY
                     MHKRIHSSLGYLTPCEYEQQWRQQNNHYCMNK"
                     /note="IS3_IS51_ORF1    e-value= 3.9e-53 complete sequence
                     hit coverage= 100%, between model( 307 aa) positions 1;
                     307 length is 283aa with 25 gaps, 0 stops, absolute
                     frame= Minus2"
     CDS             complement(3913993..3913179)
                     /colour="255 0 0"
                     /evidence=predicted
                     /translation="VRVRQLQQDYSIRQICQVLNYPRSQVYYHARG--QPD-----ES
                     ELKAAI-AGVAGAYPTYGYRRITAQLQ-RQGYCVNHKRVARLMRQIGIMAKTKVKRKR
                     TTNSEHSfPRYGNRVLNLS-IDHPEQVWVADITYIRLQQEFVYLAVVMDVFTRAIRGW
                     HLSRHIDQQLTLRALNKALERA-TPE--IHHSDQGVQYAAAAYMQLLQQHQVQISMAE
                     VGQAWQNGYAERLMRTIK-EEEVDL-SDYRNFTEAYEHIEQFLEDVYMHKRIHSSLGY
                     LTPCEYEQQWR"
                     /note="IS3_IS3_ORF1     e-value= 7.6e-54 complete sequence
                     hit coverage= 100%, between model( 286 aa) positions 1;
                     286 length is 272aa with 15 gaps, 0 stops, absolute
                     frame= Minus2"
     CDS             complement(3913993..3913179)
```

```
                     /colour="255 0 0"
                     /evidence=predicted
                     /translation="VRVRQLQQDYSIRQICQVLNYPRSQVYYHARG--QPD-----ES
                     ELKAAI-AGVAGAYPTYGYRRITAQLQ-RQGYCVNHKRVARLMRQIGIMAKTKVKRKR
                     TTNSEHSfPRYGNRVLNLS-IDHPEQVWVADITYIRLQQEFVYLAVVMDVFTRAIRGW
                     HLSRHIDQQLTLRALNKALERA-TPE--IHHSDQGVQYAAAAYMQLLQQHQVQISMAE
                     VGQAWQNGYAERLMRTIK-EEEVDL-SDYRNFTEAYEHIEQFLEDVYMHKRIHSSLGY
                     LTPCEYEQQWR"
                     /note="IS3_IS3_ORF1      e-value= 2e-54 complete sequence
hit
                     coverage= 100%, between model( 286 aa) positions 1; 286
                     length is 272aa with 15 gaps, 0 stops, absolute frame=
                     Minus2"
     CDS             complement(3913981..3913167)
                     /colour="255 0 0"
                     /evidence=predicted
                     /translation="QLQQDYSIRQICQVLNYPRSQVYYHARGQPDE---------SEL
                     KAAIAGVAGaypTYGYRRITAQLQRQGYCVNHKRVARLMRQIGIMAKTKVKRKRTTNS
                     EHSFPRY---GNRVLNLSIDHPEQVWVADITYIRLQQEFVYLAVVMDVFTRAIRGWHL
                     SRHIDQQLTLRALNKALER----ATPE---IHHSDQGVQYAAAAYMQLLQQHQVQISM
                     AEVGQAWQNGYAERLMRTIKEEEVDLSDYRNFTEAYEHIEQFLEDVYMHKRIHSSLGY
                     LTPCEYEQQWRQQNN"
                     /note="IS3_IS3_ORF2      e-value= 3.3e-65 complete sequence
                     hit coverage= 100%, between model( 288 aa) positions 1;
                     288 length is 272aa with 19 gaps, 0 stops, absolute
                     frame= Minus2"
     CDS             complement(3913981..3913167)
                     /colour="255 0 0"
                     /evidence=predicted
                     /translation="QLQQDYSIRQICQVLNYPRSQVYYHARGQPDE---------SEL
                     KAAIAGVAGaypTYGYRRITAQLQRQGYCVNHKRVARLMRQIGIMAKTKVKRKRTTNS
                     EHSFPRY---GNRVLNLSIDHPEQVWVADITYIRLQQEFVYLAVVMDVFTRAIRGWHL
                     SRHIDQQLTLRALNKALER----ATPE---IHHSDQGVQYAAAAYMQLLQQHQVQISM
                     AEVGQAWQNGYAERLMRTIKEEEVDLSDYRNFTEAYEHIEQFLEDVYMHKRIHSSLGY
                     LTPCEYEQQWRQQNN"
                     /note="IS3_IS3_ORF2      e-value= 1.2e-64 complete sequence
                     hit coverage= 100%, between model( 288 aa) positions 1;
                     288 length is 272aa with 19 gaps, 0 stops, absolute
                     frame= Minus2"
     CDS             complement(3913978..3913173)
                     /colour="255 0 0"
                     /evidence=predicted
                     /translation="LQQDYSIRQICQVLNYPRSQVYYHARGQP-----DESELKAAIA
                     GVAGAYP-TYGYRRITAQLQ---RQGYCVNHKRVARLMRQIGIMAKTKVK-RKRTTNS
                     EHSfpRYGNRVLNLS--IDHPEQVWVADITYIRLQ-QEFVYLAVVMDVFTRAIRGWHL
                     SRHIDQQLTLRALNKALERAT---PEIHHS-----DQGVQYAAAAYMQLLQQHQVQIS
                     MAEVGQAWQNGYAERLMRTIKEEEVD--LSDYRNFTEAYEHIEQFLEdVYMHKRIHSS
                     LGYLTPCEYEQQWRQQ"
                     /note="IS3_IS150_ORF2    e-value= 2.2e-46 fragment hit
                     coverage= 74.29%, between model( 389 aa) positions 101;
                     389 length is 269aa with 23 gaps, 0 stops, absolute
                     frame= Minus2"
     CDS             complement(3913963..3913176)
                     /colour="255 0 0"
                     /evidence=predicted
                     /translation="SIRQICQVLNYPRSQVYYHARGQPDESELKAAIAGV-AGAYPTY
                     GYRRITAQLQRQGYCVNHKRVARLMRQIGIMAKTKVK----RKRTTNSEHSF-PRYGN
                     RvlNLSIDHPEQVWVADITYIRL--QQEFVYLAVVMDVFTRAIRGWHLSRHIDQQLTL
                     RALNKALER----ATPEIHHSDQGVQYAAAAYMQLLQQHQVQISMAEVGQAWQNGYAE
                     RLMRTIKEEEVDLSDYRNFT--EAYEHIEQFLEDVYMHKRIHSSLGYLTPCEYEQQWR
```

Q"
/note="IS3_IS150_ORF1   e-value= 1.4e-49 fragment hit coverage= 89.87%, between model(306 aa) positions 32; 306 length is 263aa with 14 gaps, 0 stops, absolute frame= Minus2"

CDS   complement(3913963..3913764)
/colour="255 0 0"
/evidence=predicted
/translation="SIRQICQVLNYPRSQVYYHARGQPDESELKAAIAGVAGAYPTYG
YRRITAQLQRQGYCVNHKRVARL"
/note="IS3_IS407_ORF2   e-value= 2e-15 fragment hit coverage= 19.48%, between model(344 aa) positions 80; 146 length is 67aa with 0 gaps, 0 stops, absolute frame= Minus2"

CDS   complement(3913912..3913755)
/colour="255 0 0"
/evidence=predicted
/translation="YHARgQPDESELKAAIAGVAGAYPTYGYRRITAQLQRQGY----
-CVNHKRVARLMRQ"
/note="IS3_IS2_ORF2   e-value= 9.1e-09 fragment hit coverage= 18.87%, between model(302 aa) positions 48; 104 length is 53aa with 5 gaps, 0 stops, absolute frame= Minus2"

CDS   complement(3913846..3913164)
/colour="255 0 0"
/evidence=predicted
/translation="YPTYGYRRITAQLQRQGYCVNHKRVARLMRQIGIMAKTKVKRKR
TTNSEHSFPRYGNRVL-NLSIDHPEQVWVADITYIRLQQEFVYLAVVMDVFTRAIRGW
HLSRHIDQQLTLRALNKALERATPE--------IHHSDQGVQYAAAAYMQLLQQHQVQ
ISMAEVGQAWQNGYAERLMRTIKEEEVDLSDYrnFTEAYEHIEQFLEDV--YMHKRIH
SSLGYLTPCEYEQQWRQQNNH"
/note="IS3_IS51_ORF2   e-value= 1.9e-38 fragment hit coverage= 74.76%, between model(317 aa) positions 73; 309 length is 228aa with 11 gaps, 0 stops, absolute frame= Minus2"

CDS   complement(3759763..3758637)
/colour="255 0 0"
/evidence=predicted
/translation="FKHLHLGMIsDIKRKSLPAIARAVGLENHQSLHHFLSESPWVAS
hLSDkRLDIILKILDGRRLILLID------------------ETGDCKKGKSTDYVKR
QYIGNVGKKENGIvAVTAYGLFQGMILPLSFEVYKPR---------ErLKEEDEYLSK
PQIAAGMIRQLQGMGFCFELVLADSLYGEAQTNFVNVLEELKLPYILAIRSNHAVWLP
AEQAVFAEPWQSFERTFSNGT--------------------------------TETRYR
QEIIYGQRHHQKRyWLLTTDPQTLPENSTS------------------------YVMAA
APE----IKLDEIGDCYGFRTWIEYGLKQSKD-TLGWADFRMTHYEQIEKWWEIVMSA
FLMVSLFADVFNDS-------------------------CPVAHQHFAQHPwWDNQNG
WKNLLNNVR--LIIQPLIS----WNLLKRWLEVFPSRALKKGF"
/note="IS4   e-value= 9.9e-07 complete sequence hit coverage= 100%, between model(486 aa) positions 1; 486 length is 376aa with 117 gaps, 0 stops, absolute frame= Minus2"

CDS   complement(3759763..3759069)
/colour="255 0 0"
/evidence=predicted
/translation="FKHLHLGMIsDIKRKSLPAIARAVGLENHQSLHHFLSESPWVAS
hLSDkRLDIILKILDGRRLILLID------------------ETGDCKKGKSTDYVKR
QYIGNVGKKENGIvAVTAYGLFQGMILPLSFEVYKPR---------ErLKEEDEYLSK
PQIAAGMIRQLQGMGFCFELVLADSLYGEAQTNFVNVLEELKLPYILAIRSNHAVWLP
AEQAVFAEPWQSFERTFSNGTTETRYrQEIIYGqrhhQKRY"
/note="IS4   e-value= 4.7e-14 fragment hit coverage=

51.23%, between model(486 aa) positions 1; 249 length is 232aa with 27 gaps, 0 stops, absolute frame= Minus2"
CDS complement(3688744..3687618)
/colour="255 0 0"
/evidence=predicted
/translation="FKHLHLGMIsDIKRKSLPAIARAVGLENHQSLHHFLSESPWVAS
hLSDkRLDIILKILDGRRLILLID------------------ETGDCKKGKSTDYVKR
QYIGNVGKKENGIvAVTAYGLFQGMILPLSFEVYKPR---------ErLKEEDEYLSK
PQIAAGMIRQLQGMGFCFELVLADSLYGEAQTNFVNVLEELKLPYILAIRSNHAVWLP
AEQAVFAEPWQSFERTFSNGT--------------------------------TETRYR
QEIIYGQRHHQKRyWLLTTDPQTLPENSTS-----------------------YVMAA
APE----IKLDEIGDCYGFRTWIEYGLKQSKD-TLGWADFRMTHYEQIEKWWEIVMSA
FLMVSLFADVFNDS-------------------------CPVAHQHFAQHPwWDNQNG
WKNLLNNVR--LIIQPLIS----WNLLKRWLEVFPSRALKKGF"
/note="IS4 e-value= 9.9e-07 complete sequence hit coverage= 100%, between model(486 aa) positions 1; 486 length is 376aa with 117 gaps, 0 stops, absolute frame= Minus2"
CDS complement(3688744..3688050)
/colour="255 0 0"
/evidence=predicted
/translation="FKHLHLGMIsDIKRKSLPAIARAVGLENHQSLHHFLSESPWVAS
hLSDkRLDIILKILDGRRLILLID------------------ETGDCKKGKSTDYVKR
QYIGNVGKKENGIvAVTAYGLFQGMILPLSFEVYKPR---------ErLKEEDEYLSK
PQIAAGMIRQLQGMGFCFELVLADSLYGEAQTNFVNVLEELKLPYILAIRSNHAVWLP
AEQAVFAEPWQSFERTFSNGTTETRYrQEIIYGqrhhQKRY"
/note="IS4 e-value= 4.7e-14 fragment hit coverage= 51.23%, between model(486 aa) positions 1; 249 length is 232aa with 27 gaps, 0 stops, absolute frame= Minus2"
CDS complement(3511168..3510495)
/colour="255 0 0"
/evidence=predicted
/translation="SXGICMKCPECGGQH-IHKNGHRR-GKQNHICVTCGRQFL--ST
YSKRGYSDWTKRLCLRMYVNGMGLKGIERVIGVAHTTVIHWVQQVGvllPNAYDPDDI
PQ------------VGELDELETFVGKKRNKVWIW-TVVDHFHPG--ILGWVVGDHSA
ETFRPLWqaIVCWQCFFWVSDGNPVYpgFIPEgDQIVSKTYMTRVEGENTRLRHYLAR
LHRKTLCYSKSVDMLKHSIRLL---IHYL"
/note="IS1_ORF1 e-value= 3.5e-42 complete sequence hit coverage= 100%, between model(239 aa) positions 1; 239 length is 225aa with 22 gaps, 1 stops, absolute frame= Minus2"
CDS complement(3511168..3510495)
/colour="255 0 0"
/evidence=predicted
/translation="SXGICMKCPECGGQH-IHKNGHRR-GKQNHICVTCGRQFL--ST
YSKRGYSDWTKRLCLRMYVNGMGLKGIERVIGVAHTTVIHWVQQVGvllPNAYDPDDI
PQ------------VGELDELETFVGKKRNKVWIW-TVVDHFHPG--ILGWVVGDHSA
ETFRPLWqaIVCWQCFFWVSDGNPVYpgFIPEgDQIVSKTYMTRVEGENTRLRHYLAR
LHRKTLCYSKSVDMLKHSIRLL---IHYL"
/note="IS1_ORF1 e-value= 1.9e-41 complete sequence hit coverage= 100%, between model(239 aa) positions 1; 239 length is 225aa with 22 gaps, 1 stops, absolute frame= Minus2"
CDS complement(3511168..3510489)
/colour="255 0 0"
/evidence=predicted
/translation="SXGICMKCPECGGQH-IHKNGHRR-GKQNHICVTCGR----QFL
STYSKRGysdwTKRLCLRMYVNGMGLKGIERVIGVAHTTVIHWVQQV--GVLLPNAYD
PDDIPQVGELDELETFVGKKRNKVWIWTVVDHFHPGILGWVVGDHSAETFRPLWQAIV
CWQ-CFFWVSDGNPVYPGFIPEG----DQIVSKTYMTRVEGENTRLRHYLARLHRKTL

```
                     CYSKSVDMLKHSIRLLI-HYLKF"
                     /note="IS1_ORF2    e-value= 3.2e-29 complete sequence hit
                     coverage= 100%, between model( 237 aa) positions 1; 237
                     length is 227aa with 14 gaps, 1 stops, absolute frame=
                     Minus2"
     CDS             complement(3510889..3510489)
                     /colour="255 0 0"
                     /evidence=predicted
                     /translation="DPDDIPQVGELDELETFVGKKRNKVWIWTVVDHFHPGILGWVVG
                     DHSAETFRPLWQAIVCWQ-CFFWVSDGNPVYPGFIPEG----DQIVSKTYMTRVEGEN
                     TRLRHYLARLHRKTLCYSKSVDMLKHSIRLLI-HYLKF"
                     /note="IS1_ORF2    e-value= 8.3e-32 fragment hit coverage=
                     59.07%, between model( 237 aa) positions 98; 237 length
                     is 134aa with 6 gaps, 0 stops, absolute frame= Minus2"
     CDS             complement(3407053..3406647)
                     /colour="255 0 0"
                     /evidence=predicted
                     /translation="MLLVRPISTESLRLLHRIYHSSRHHQVRQRAHCLILFAQGWPPY
                     TLASLFSVSPKTVYNWLKAWNNRG---FAGLYNHPGRGRKPMFNPDQQQQIYEWT-QA
                     SPIQ---------LNQVLAQIEQQWSVRVSKATVKRVLKQMDMSWHR"
                     /note="IS630 e-value= 8.7e-15 fragment hit coverage=
                     39.63%, between model( 376 aa) positions 1; 149 length is
                     136aa with 13 gaps, 0 stops, absolute frame= Minus2"
     CDS             complement(3406993..3406656)
                     /colour="255 0 0"
                     /evidence=predicted
                     /translation="SSRHHQVRQRAHCLILFAQGWPPYTLASLFSVSPKTVYNWLKAW
                     NNRGFAGLYNH---PGRGRKPMfNPDQQQQIYEWTQASPIQLNQVLAQIEQQWSVR--
                     -VSKATVKRVLKQMDMS"
                     /note="IS481 e-value= 3.5e-09 fragment hit coverage=
                     33.62%, between model( 351 aa) positions 1; 118 length is
                     113aa with 6 gaps, 0 stops, absolute frame= Minus2"
     CDS             complement(2632693..2631567)
                     /colour="255 0 0"
                     /evidence=predicted
                     /translation="FKHLHLGMIsDIKRKSLPAIARAVGLENHQSLHHFLSESPWVAS
                     hLSDkRLDIILKILDGRRLILLID------------------ETGDCKKGKSTDYVKR
                     QYIGNVGKKENGIvAVTAYGLFQGMILPLSFEVYKPR---------ErLKEEDEYLSK
                     PQIAAGMIRQLQGMGFCFELVLADSLYGEAQTNFVNVLEELKLPYILAIRSNHAVWLP
                     AEQAVFAEPWQSFERTFSNGT-------------------------------TETRYR
                     QEIIYGQRHHQKRyWLLTTDPQTLPENSTS-----------------------YVMAA
                     APE----IKLDEIGDCYGFRTWIEYGLKQSKD-TLGWADFRMTHYEQIEKWWEIVMSA
                     FLMVSLFADVFNDS-------------------------CPVAHQHFAQHPwWDNQNG
                     WKNLLNNVR--LIIQPLIS----WNLLKRWLEVFPSRALKKGF"
                     /note="IS4   e-value= 9.9e-07 complete sequence hit
                     coverage= 100%, between model( 486 aa) positions 1; 486
                     length is 376aa with 117 gaps, 0 stops, absolute frame=
                     Minus2"
     CDS             complement(2632693..2631999)
                     /colour="255 0 0"
                     /evidence=predicted
                     /translation="FKHLHLGMIsDIKRKSLPAIARAVGLENHQSLHHFLSESPWVAS
                     hLSDkRLDIILKILDGRRLILLID------------------ETGDCKKGKSTDYVKR
                     QYIGNVGKKENGIvAVTAYGLFQGMILPLSFEVYKPR---------ErLKEEDEYLSK
                     PQIAAGMIRQLQGMGFCFELVLADSLYGEAQTNFVNVLEELKLPYILAIRSNHAVWLP
                     AEQAVFAEPWQSFERTFSNGTTETRYrQEIIYGqrhhQKRY"
                     /note="IS4   e-value= 4.7e-14 fragment hit coverage=
                     51.23%, between model( 486 aa) positions 1; 249 length is
                     232aa with 27 gaps, 0 stops, absolute frame= Minus2"
     CDS             complement(2586391..2586240)
```

/colour="255 0 0"
/evidence=predicted
/translation="QLCLILDNNPTHKG-KMRSQLAIHLEqmgltqsiQVEFLYLPSY
SPKLNLVE"
/note="IS630 e-value= 4.3e-06 fragment hit coverage=
11.70%, between model(376 aa) positions 279; 322 length
is 51aa with 1 gaps, 0 stops, absolute frame= Minus2"

CDS complement(2459947..2458710)
/colour="255 0 0"
/evidence=predicted
/translation="IRIPLNLPDI--RVLELSKTERGDWL------IKIESTLQGTTC
HQCGREITDL-HCHDQ-PFRIRHLPLFEVPVYLEIRPKRYRCKYCDDhpTTTQHLEWH
EPRSPNTKAYERWLLRILIN-STVSDVARKLGVSEDIVSGIIDRWIAAQVDWSEYADL
KVMGMDEISLKRG--HR---DFVVLITIPTTdgVDILAVLADRKQQTVANFLQSIP-I
DLRQTIERVCTDMYQGFVSAVREQLPQAKIVIDRFHVTKAYRNCADTVRKREVKRLRR
ELS------------KQEYD-----------SIKGAMWPFRKRPENLKES-EQQLLER
LFA--YSPQLKQAYNLREKLTQIFEGRYTKHGAKcAIRAWCKQVLKSDiKEFDCFLTT
INNWMDEMTNYFLEG-WTSGFVEGFNNRVKVLKRRCYGIFDIERLFQRISLDLNGYQT
FAVTXTL"
/note="ISL3 e-value= 1.8e-43 complete sequence hit
coverage= 100%, between model(451 aa) positions 1; 451
length is 413aa with 44 gaps, 1 stops, absolute frame=
Minus2"

CDS complement(2459788..2458710)
/colour="255 0 0"
/evidence=predicted
/translation="RIRHLPLFEVPVYLEIRPKRYRCKYCDDhpTTTQHLEWHEPRSP
NTKAYERWLLRILIN-STVSDVARKLGVSEDIVSGIIDRWIAAQVDWSEYADLKVMGM
DEISLKRG--HR---DFVVLITIPTTdgVDILAVLADRKQQTVANFLQSIP-IDLRQT
IERVCTDMYQGFVSAVREQLPQAKIVIDRFHVTKAYRNCADTVRKREVKRLRRELS--
----------KQEYD-----------SIKGAMWPFRKRPENLKES-EQQLLERLFA--
YSPQLKQAYNLREKLTQIFEGRYTKHGAKcAIRAWCKQVLKSDiKEFDCFLTTINNWM
DEMTNYFLEG-WTSGFVEGFNNRVKVLKRRCYGIFDIERLFQRISLDLNGYQTFAVTX
TL"
/note="ISL3 e-value= 1e-49 fragment hit coverage= 86.03%,
between model(451 aa) positions 64; 451 length is 360aa
with 34 gaps, 1 stops, absolute frame= Minus2"

CDS complement(2413129..2412849)
/colour="255 0 0"
/evidence=predicted
/translation="PMSHLIDTLKQVPDFRSARGRTHPLWLLLLLMVMGMLAGYQGYC
PLETFTSDYQQPLCELLGLENF-----QVPSHCTFRRVMKGLDFQSLSHQFEAWM"
/note="ISAs1 e-value= 1.2e-14 fragment hit coverage=
25.45%, between model(389 aa) positions 1; 99 length is
94aa with 5 gaps, 0 stops, absolute frame= Minus2"

CDS complement(2413129..2412096)
/colour="255 0 0"
/evidence=predicted
/translation="PMSHLIDTLKQVPDFRSARGRTHPLWLLLLLMVMGMLAGYQGYC
PLETFTSDYQQPLCELLGLENF-----QVPSHCTFRRVMKGLDFQSLSHQFEAWMlsk
aqthspDNYAASMDGK-RIC---QGLTDENGKQRFVG---LVSIFAVEAGITLKLEAL
TQEDNsEIKVVQALLETLQLDG-LLITMDALHAQKNTSAS-CGFGX------------
XLSYRRXAQSGPSLRPPPDLL-XVSXTHGxayplHTEXRTRGTSVYPGLXACRHSPTR
-VGSHSLGALCPAMGYSSRQG----VSQH-----GLLHQFS-----------CHLTPS
LAISGPRALGHXKS-----------VALAEGCGLWXRXLSTRGXTSTAQLVSAXNHCD
XYSASKRLSIPQ"
/note="ISAs1 e-value= 7e-07 complete sequence hit
coverage= 100%, between model(389 aa) positions 1; 389
length is 345aa with 59 gaps, 13 stops, absolute frame=
Minus2"

```
     CDS             complement(2404702..2404278)
                     /colour="255 0 0"
                     /evidence=predicted
                     /translation="GRQRFNVLGALNAVTKEvTSITNHTYINSHSMCLLLAKLALLDP
                     VI--PISVILDNARYQKCQLVTDFAEIVD--IELVYLPSYSPHLNLIERLWRFVRKEC
                     LYSKYYSDFHSFKGAIQQCIDQCNTEHKAKLTSLLSLKFQSFQK"
                     /note="IS630 e-value= 6.2e-18 fragment hit coverage=
                     38.56%, between model( 376 aa) positions 232; 376 length
                     is 142aa with 4 gaps, 0 stops, absolute frame= Minus2"
     CDS             complement(2284996..2284359)
                     /colour="255 0 0"
                     /evidence=predicted
                     /translation="MASKTTNVkPVVVSLWKLLSKSELIHPLKAXSISYCLKRYLWQV
                     L-----PGS------VMFLKAGYKSTXTVsMKQCLVLXTYHQKKGR------------
                     ---LTLQCDEMWSFVNDKSNKQWIWLALDVITREIVGVYVGARSKQGARQLWNSLPGI
                     YRQCAVaYTDFWDAYGCVFPKQ-RHQAVGKETGQTCYIERFNCTMRQRVSRLVRKTLS
                     FSKKLENHIGAIWMFVHHYNAS"
                     /note="IS1_ORF2    e-value= 4.6e-13 complete sequence hit
                     coverage= 100%, between model( 237 aa) positions 1; 237
                     length is 213aa with 27 gaps, 3 stops, absolute frame=
                     Minus2"
     CDS             complement(2284744..2284368)
                     /colour="255 0 0"
                     /evidence=predicted
                     /translation="DEMWSFVNDKSNKQWIWLALDVITREIVGVYVGA----RSKQGA
                     RQLwNSLPGIYRQCAVAYTDFWDAYgCVFPKQRHQAVGKEtgQTCYIERFNCTMRQRV
                     SRLVRKTLSFSKKLENHIGAIWMF---VHHY"
                     /note="IS1_ORF1    e-value= 4.6e-14 fragment hit coverage=
                     53.97%, between model( 239 aa) positions 111; 239 length
                     is 126aa with 7 gaps, 0 stops, absolute frame= Minus2"
     CDS             complement(2284744..2284359)
                     /colour="255 0 0"
                     /evidence=predicted
                     /translation="DEMWSFVNDKSNKQWIWLALDVITREIVGVYVGARSKQGARQLW
                     NSLPGIYRQCAVaYTDFWDAYGCVFPKQ-RHQAVGKETGQTCYIERFNCTMRQRVSRL
                     VRKTLSFSKKLENHIGAIWMFVHHYNAS"
                     /note="IS1_ORF2    e-value= 6.2e-32 fragment hit coverage=
                     54.43%, between model( 237 aa) positions 109; 237 length
                     is 129aa with 1 gaps, 0 stops, absolute frame= Minus2"
     CDS             complement(2088655..2088024)
                     /colour="255 0 0"
                     /evidence=predicted
                     /translation="TQTLDRTXRAM-----PTAYDSDLTTLQWELLEPLIPAAK----
                     ---PGGRPRTTDMRSVLNAIFYLVVTGCQWRQLPHDFPCWSTVYSYFRRWRDDGTWVH
                     INEHLRMQERVSEDRHPSPSAAICDAQSVKVG--NPR---------------------
                     --------------CHTNS----LIEXPLXSKHPR---------YKNIPLEKPPPSGR
                     GYXYAQIKRSP-------VPQ-----------------XNQSFCLXKAFLGPAIIALP
                     DXVLdyETAVQSATHSQAIEP"
                     /note="IS5_IS1031  e-value= 3.4e-16 complete sequence hit
                     coverage= 100%, between model( 295 aa) positions 1; 295
                     length is 211aa with 86 gaps, 7 stops, absolute frame=
                     Minus2"
     CDS             complement(2088613..2088297)
                     /colour="255 0 0"
                     /evidence=predicted
                     /translation="YDSDLTTLQWELLEPLIPAAK-------PGGRPRTTDMRSVLNA
                     IFYLVVTGCQWRQLPHDFPCWSTVYSYFRRWRDDGTWVHINEHLRMQERVSEDRHPSP
                     SAAICDAQSVK"
                     /note="IS5_IS1031  e-value= 7.2e-51 fragment hit coverage=
                     38.31%, between model( 295 aa) positions 20; 132 length
```

is 106aa with 7 gaps, 0 stops, absolute frame= Minus2"
CDS complement(2088604..2088366)
/colour="255 0 0"
/evidence=predicted
/translation="DLTTLQWELLEPLIPAAKP-GGRPRT-TDMRSVLNAIFYLVVTG
CQWRQLPHDFPCWSTVYSYFRRWRDDGTWVHINEHLRM"
/note="IS5_IS427 e-value= 9.2e-19 fragment hit coverage=
27.70%, between model(296 aa) positions 125; 206 length
is 80aa with 2 gaps, 0 stops, absolute frame= Minus2"
CDS complement(2087236..2086668)
/colour="255 0 0"
/evidence=predicted
/translation="SQISIRKGLGM-----RDKY-------------------Q----
-------QRATP-------IIQVTH-----------------IWNWY----------
---------------------------------HSI----GFDGGKMVKGRKRHVLVD
TLGLVLMVMVTAANISDQR-GAKMLFWKARRQGaslSRLVRIWADAGYQGQAFMKWVM
DRFQYVLEVIKRSDNLAGFQVVPKRWIVERTFGWLLWSRRLNKDYEVLTRTTEALAYV
AMIRLMVRRLAQEYXNFSNSLX"
/note="IS5_IS1031 e-value= 2.9e-19 complete sequence hit
coverage= 100%, between model(295 aa) positions 1; 295
length is 190aa with 108 gaps, 2 stops, absolute frame=
Minus2"
CDS complement(2087131..2086668)
/colour="255 0 0"
/evidence=predicted
/translation="GFDGGKMVKGRKRHVLVDTLGLVLMVMVTAANISDQR-GAKMLF
WKARRQGaslSRLVRIWADAGYQGQAFMKWVMDRFQYVLEVIKRSDNLAGFQVVPKRW
IVERTFGWLLWSRRLNKDYEVLTRTTEALAYVAMIRLMVRRLAQEYXNFSNSLX"
/note="IS5_IS1031 e-value= 2.2e-60 fragment hit coverage=
51.86%, between model(295 aa) positions 143; 295 length
is 155aa with 1 gaps, 2 stops, absolute frame= Minus2"
CDS complement(1894558..1894341)
/colour="255 0 0"
/evidence=predicted
/translation="RIRFVYIPKHTSWLNQIECWFSILVRRLIRRGNFTSKDDLQQRI
LEFIEYFNHtMAKPFQWQFKGFQPRLXLM"
/note="IS630 e-value= 4e-10 fragment hit coverage=
19.15%, between model(376 aa) positions 305; 376 length
is 73aa with 0 gaps, 1 stops, absolute frame= Minus2"
CDS complement(1894510..1894401)
/colour="255 0 0"
/evidence=predicted
/translation="IECWFSILVRRLIR--RGNFTSKDDLQQRILEFIEYFNH"
/note="IS3_IS150_ORF2 e-value= 2e-08 fragment hit
coverage= 10.03%, between model(389 aa) positions 329;
367 length is 37aa with 2 gaps, 0 stops, absolute frame=
Minus2"
CDS complement(1226248..1225905)
/colour="255 0 0"
/evidence=predicted
/translation="KYIVTLTPEERSELIQLTRRRTLSARKMKRAQILMLADEGHKDD
TITQMLNAGISTVHRTRQKFVEGG---VEfALNERPRPGGQKKLDSKAEALLIATACS
DPPTG----CCRWTMQLLAE"
/note="IS630 e-value= 1.2e-15 fragment hit coverage=
32.18%, between model(376 aa) positions 1; 121 length is
115aa with 7 gaps, 0 stops, absolute frame= Minus2"
CDS complement(1119469..1119321)
/colour="255 0 0"
/evidence=predicted
/translation="LMNSGLFRLPGKKHLIQGFERPDVVVMDVTETPIERPQKGQKAY

```
                     YAGKKR"
                     /note="IS5_ISL2    e-value= 1.8e-17 fragment hit coverage=
                     19.23%, between model( 260 aa) positions 80; 129 length
                     is 50aa with 0 gaps, 0 stops, absolute frame= Minus2"
     CDS             complement(669352..668715)
                     /colour="255 0 0"
                     /evidence=predicted
                     /translation="MASKTTNVkPVVVSLWKLLSKSELIHPLKAXSISYCLKRYLWQV
                     L-----PGS------VMFLKAGYKSTXTVsMKQCLVLXTYHQKKGR------------
                     ---LTLQCDEMWSFVNDKSNKQWIWLALDVITREIVGVYVGARSKQGARQLWNSLPGI
                     YRQCAVaYTDFWDAYGCVFPKQ-RHQAVGKETGQTCYIERFNCTMRQRVSRLVRKTLS
                     FSKKLENHIGAIWMFVHHYNAS"
                     /note="IS1_ORF2    e-value= 4.6e-13 complete sequence hit
                     coverage= 100%, between model( 237 aa) positions 1; 237
                     length is 213aa with 27 gaps, 3 stops, absolute frame=
                     Minus2"
     CDS             complement(669100..668724)
                     /colour="255 0 0"
                     /evidence=predicted
                     /translation="DEMWSFVNDKSNKQWIWLALDVITREIVGVYVGA----RSKQGA
                     RQLwNSLPGIYRQCAVAYTDFWDAYgCVFPKQRHQAVGKEtgQTCYIERFNCTMRQRV
                     SRLVRKTLSFSKKLENHIGAIWMF---VHHY"
                     /note="IS1_ORF1    e-value= 4.6e-14 fragment hit coverage=
                     53.97%, between model( 239 aa) positions 111; 239 length
                     is 126aa with 7 gaps, 0 stops, absolute frame= Minus2"
     CDS             complement(669100..668715)
                     /colour="255 0 0"
                     /evidence=predicted
                     /translation="DEMWSFVNDKSNKQWIWLALDVITREIVGVYVGARSKQGARQLW
                     NSLPGIYRQCAVaYTDFWDAYGCVFPKQ-RHQAVGKETGQTCYIERFNCTMRQRVSRL
                     VRKTLSFSKKLENHIGAIWMFVHHYNAS"
                     /note="IS1_ORF2    e-value= 6.2e-32 fragment hit coverage=
                     54.43%, between model( 237 aa) positions 109; 237 length
                     is 129aa with 1 gaps, 0 stops, absolute frame= Minus2"
     CDS             complement(557656..557520)
                     /colour="255 0 0"
                     /evidence=predicted
                     /translation="WQTDGWEGYSRQLANE---VIHHVSKALTQRLERTNGILRQQTS
                     RWHRR"
                     /note="IS1_ORF2    e-value= 1.2e-12 fragment hit coverage=
                     20.68%, between model( 237 aa) positions 164; 212 length
                     is 46aa with 3 gaps, 0 stops, absolute frame= Minus2"
     CDS             complement(522460..522219)
                     /colour="255 0 0"
                     /evidence=predicted
                     /translation="VKLAELIPWESFESEYADQFSQ---TMGAPAKPFRVALGTLIIK
                     EKLGISDAETVEQIRENPYLQYFLGFSEYRESAPFDASML"
                     /note="IS5_IS5     e-value= 2.6e-06 fragment hit coverage=
                     19.91%, between model( 422 aa) positions 23; 106 length
                     is 81aa with 3 gaps, 0 stops, absolute frame= Minus2"
     CDS             complement(481126..480390)
                     /colour="255 0 0"
                     /evidence=predicted
                     /translation="-----TGVYKRTFkpMLHAWHTYHLSRSN--AGRPPKLCRCDQL
                     LVALQYWREYRTYFHIAGDWEVSESTVCRIVHQVETALMNSGLFRLPGQKSLLQGFER
                     PDVVVMDVTETPIERPQTRQKAYYSGKKRDIPSNTRLSLTATLXRLSALT--------
                     --LVQVVGMIFrssrfqvSISIQIPRVCKIADIKGLQPII--PTAM----FLSRSHNT
                     VNXLPCSESITVLXVRNEWALNTLIAAXRFSEFCRSAIAIVVVATRCGVTX"
                     /note="IS5_ISL2    e-value= 1.9e-21 complete sequence hit
                     coverage= 100%, between model( 260 aa) positions 1; 260
```

```
                     length is 246aa with 23 gaps, 5 stops, absolute frame=
                     Minus2"
     CDS             complement(481066..480756)
                     /colour="255 0 0"
                     /evidence=predicted
                     /translation="SRSNAGRPPKLCRCDQLLVALQYWREYRTYFHIAGDWEVSESTV
                     CRIVHQVETALMNSGLFRLPGQKSLLQGFERPDVVVMDVTETPIERPQTRQKAYYSGK
                     KR"
                     /note="IS5_ISL2    e-value= 9.3e-44 fragment hit coverage=
                     40%, between model( 260 aa) positions 26; 129 length is
                     104aa with 0 gaps, 0 stops, absolute frame= Minus2"
     CDS             complement(475153..474390)
                     /colour="255 0 0"
                     /evidence=predicted
                     /translation="SPYQHLSLYLKKLRLSHMLTHWESIESQA-MQENWSYAEFLLAL
                     CETKAQRREQARLKRALTEARLPNA-KSFTNFDFSHCPQLNPAPLMQLAAdPGWLERA
                     ENCLILGPSGVGKTHLATGVSKKMLEFGKRVKFFAANALVQHLQQAKLQLQLH-PMLK
                     KLDRYDLLVLDDLGYCKKSEAETSVLFELIAHRYERKSLLITANQPFSQWDDIFT-DS
                     MMAVAAIDRLIHHGLIIKIQADSYRRKSATQRTAQTQSPPQ"
                     /note="IS21_ORF2   e-value= 7.2e-80 complete sequence hit
                     coverage= 100%, between model( 258 aa) positions 1; 258
                     length is 255aa with 4 gaps, 0 stops, absolute frame=
                     Minus2"
     CDS             complement(475153..474390)
                     /colour="255 0 0"
                     /evidence=predicted
                     /translation="SPYQHLSLYLKKLRLSHMLTHWESIESQA-MQENWSYAEFLLAL
                     CETKAQRREQARLKRALTEARLPNA-KSFTNFDFSHCPQLNPAPLMQLAAdPGWLERA
                     ENCLILGPSGVGKTHLATGVSKKMLEFGKRVKFFAANALVQHLQQAKLQLQLH-PMLK
                     KLDRYDLLVLDDLGYCKKSEAETSVLFELIAHRYERKSLLITANQPFSQWDDIFT-DS
                     MMAVAAIDRLIHHGLIIKIQADSYRRKSATQRTAQTQSPPQ"
                     /note="IS21_ORF2   e-value= 1.9e-80 complete sequence hit
                     coverage= 100%, between model( 258 aa) positions 1; 258
                     length is 255aa with 4 gaps, 0 stops, absolute frame=
                     Minus2"
     CDS             complement(466177..466083)
                     /colour="255 0 0"
                     /evidence=predicted
                     /translation="AERG--ITLVELPSYSPHLNLIERLWQFMKYQWI"
                     /note="IS630 e-value= 1.7e-09 fragment hit coverage=
                     9.04%, between model( 376 aa) positions 300; 333 length
                     is 32aa with 2 gaps, 0 stops, absolute frame= Minus2"
     CDS             complement(339451..339123)
                     /colour="255 0 0"
                     /evidence=predicted
                     /translation="MVENRRGNHTVTRLTVHIVWVTKYRYQVLKGEVQKRCRELLIQI
                     CDAEDIRILKGVVSKDHVHMLIEYPPSKSISDIVKCLKGRTSRRLQQEYREXEKRYWG
                     KHLWAIGY"
                     /note="IS605 e-value= 3e-25 fragment hit coverage=
                     72.37%, between model( 152 aa) positions 1; 110 length is
                     110aa with 0 gaps, 1 stops, absolute frame= Minus2"
     CDS             complement(339451..338973)
                     /colour="255 0 0"
                     /evidence=predicted
                     /translation="MVENRRGNHTVTRLTVHIVWVTKYRYQVLKGEVQKRCRELLIQI
                     CDAEDIRILKGVVSKDHVHMLIEYPPSKSISDIVKCLKGRTSRRLQQEYREXEKRYWG
                     KHLWAIGYGAWSTGnISEQMVEEYLEHHrlvsnnDLDTFMLEXIrTLVLRGTGLSVDK
                     "
                     /note="IS605 e-value= 2.6e-17 complete sequence hit
                     coverage= 100%, between model( 152 aa) positions 1; 152
```

```
                     length is 160aa with 0 gaps, 2 stops, absolute frame=
                     Minus2"
     CDS             complement(339448..339063)
                     /colour="255 0 0"
                     /evidence=predicted
                     /translation="VENRRGNHTVTRLTVHIVWVTKYRYQVLKGEVQKRCRELLI-QI
                     CDAEDIRILKGVVSKDHVHMLIEYPPSKSISDIVKCLKGRTSRRLQQEYREXEKRYWG
                     KHLWAIGYGAWSTGNISEQMVEEYLEHH"
                     /note="IS200 e-value= 1.2e-47 fragment hit coverage=
                     84.42%, between model( 154 aa) positions 1; 130 length is
                     129aa with 1 gaps, 1 stops, absolute frame= Minus2"
     CDS             complement(339448..339015)
                     /colour="255 0 0"
                     /evidence=predicted
                     /translation="VENRRGNHTVTRLTVHIVWVTKYRYQVLKGEVQKRCRELLI-QI
                     CDAEDIRILKGVVSKDHVHMLIEYPPSKSISDIVKCLKGRTSRRLQQEYREXEKRYWG
                     KHLWAIGYGAWSTGNISEQMVEEYLEHHRLVSNNDL--------DTFMLEXI"
                     /note="IS200 e-value= 1.5e-46 complete sequence hit
                     coverage= 100%, between model( 154 aa) positions 1; 154
                     length is 145aa with 9 gaps, 2 stops, absolute frame=
                     Minus2"
     CDS             complement(339448..339063)
                     /colour="255 0 0"
                     /evidence=predicted
                     /translation="VENRRGNHTVTRLTVHIVWVTKYRYQVLKGE-VQKRCRELLIQI
                     CDAEDIRILKGVVSKDHVHMLIEYPPSKSISDIVKCLKGRTSRRLQQEYREXEKRYWG
                     KHLWAIGYGAWSTGNISEQMVEEYLEHH"
                     /note="IS200_IS605 e-value= 1.3e-45 fragment hit
                     coverage= 84.97%, between model( 153 aa) positions 1; 130
                     length is 129aa with 1 gaps, 1 stops, absolute frame=
                     Minus2"
     CDS             complement(339448..338994)
                     /colour="255 0 0"
                     /evidence=predicted
                     /translation="VENRRGNHTVTRLTVHIVWVTKYRYQVLKGE-VQKRCRELLIQI
                     CDAEDIRILKGVVSKDHVHMLIEYPPSKSISDIVKCLKGRTSRRLQQEYREXEKRYWG
                     KHLWAIGYGAWSTGNISEQMVEEYLEHHRLVSNNDLDTFMLEXIRTLVLRG"
                     /note="IS200_IS605 e-value= 4.2e-45 complete sequence hit
                     coverage= 100%, between model( 153 aa) positions 1; 153
                     length is 152aa with 1 gaps, 2 stops, absolute frame=
                     Minus2"
     CDS             complement(323713..323475)
                     /colour="255 0 0"
                     /evidence=predicted
                     /translation="RNQQHYIRQRLTAIKLLNEGHSRTQVSEQVGCSYDTLTRWMDKY
                     LDGGLQGLVQSI--RHQKPSRLSPEEQQQLKEMVLTQR"
                     /note="IS481 e-value= 6.3e-08 fragment hit coverage=
                     23.36%, between model( 351 aa) positions 1; 82 length is
                     80aa with 2 gaps, 0 stops, absolute frame= Minus2"
     CDS             complement(323686..323280)
                     /colour="255 0 0"
                     /evidence=predicted
                     /translation="RLTAIKLLNEGHSRTQVSEQVGCSYDTLTRWMDKYLDGG---LQ
                     GLVQSIRHQKPSRLSPEEQQQLKEMVLTQRPTDYGIDRNMWTGAILAVVIEQRFEVQL
                     KDSRIYELLSELGLSYQRAHRDYA--NADLNAQKEWVAA"
                     /note="IS630 e-value= 9.2e-30 fragment hit coverage=
                     37.50%, between model( 376 aa) positions 30; 170 length
                     is 136aa with 5 gaps, 0 stops, absolute frame= Minus2"
     CDS             complement(322042..321891)
                     /colour="255 0 0"
```

/evidence=predicted
/translation="QLCLILDNNPTHKG-KMRSQLAIHLEqmgltqsiQVEFLYLPSY
SPKLNLVE"
/note="IS630 e-value= 4.3e-06 fragment hit coverage=
11.70%, between model(376 aa) positions 279; 322 length
is 51aa with 1 gaps, 0 stops, absolute frame= Minus2"
CDS complement(259486..259350)
/colour="255 0 0"
/evidence=predicted
/translation="WQTDGWEGYARQLPDE---VVHEVSKALTQRLERTNGIVRQQTG
RWHRR"
/note="IS1_ORF2 e-value= 1.8e-11 fragment hit coverage=
20.68%, between model(237 aa) positions 164; 212 length
is 46aa with 3 gaps, 0 stops, absolute frame= Minus2"
CDS complement(127654..127374)
/colour="255 0 0"
/evidence=predicted
/translation="PMSHLIDTLKQVPDFRSARGRTHPLWLLLLLMVMGMLAGYQGYC
PLETFTSDYQQPLCELLGLENF-----QVPSHCTFRRVMKGLDFQSLSHQFEAWM"
/note="ISAs1 e-value= 1.2e-14 fragment hit coverage=
25.45%, between model(389 aa) positions 1; 99 length is
94aa with 5 gaps, 0 stops, absolute frame= Minus2"
CDS complement(105898..105813)
/colour="255 0 0"
/evidence=predicted
/translation="ITYHKLFHTEWECKYRVVFIPKYCQKCIY"
/note="IS200 e-value= 2.1e-06 fragment hit coverage=
18.83%, between model(154 aa) positions 1; 29 length is
29aa with 0 gaps, 0 stops, absolute frame= Minus2"
CDS complement(105898..105807)
/colour="255 0 0"
/evidence=predicted
/translation="ITYHKLFHTEWECKYRVVFIPKYCQKCIYSN"
/note="IS200_IS605 e-value= 4.7e-06 fragment hit
coverage= 20.26%, between model(153 aa) positions 1; 31
length is 31aa with 0 gaps, 0 stops, absolute frame=
Minus2"
CDS complement(105892..105807)
/colour="255 0 0"
/evidence=predicted
/translation="---YHKLFHTEWECKYRVVFIPKYCQKCIYSN"
/note="IS605 e-value= 9.3e-06 fragment hit coverage=
21.05%, between model(152 aa) positions 1; 32 length is
29aa with 3 gaps, 0 stops, absolute frame= Minus2"
CDS complement(62245..61035)
/colour="255 0 0"
/evidence=predicted
/translation="DELIGENPQPEdiLGESGLLKRLSKRLVERALAGELTHHLQQSS
NDQS-GDeNSNGPRNSRNGYSKKTVQSEQG-EMDLSIPRDRCG-EFEPVLVPKGQRRI
A-GLDEKIIALYARGMTTRDIRAQLVELYG--ANISEALISDVTNSVMEEVKDWRSRP
LDEV--YPIVYLDALYVNIKVNGQVSKRAVYVALAVTVEGNKELLGLWIGAAerEGAK
FWLSVLTDLKNRGTQDILIACCDGLKGFPQAIESVYPQTQVQVCIVHLIRNSLRHVPW
KESRAVAADLKPIYQAATLE-ESEAALDAFAH-KWDETYPGISQIWIRHWDNVAPLFQ
YPMAIRKVIYTTNAIESLNRSLRKVIKTKAVFPNEESVYKLMFLAMRNISKR-WTRPI
LNWKAALSHFAILFPTRFNYXIH"
/note="IS256 e-value= 1.4e-166 complete sequence hit
coverage= 100%, between model(410 aa) positions 1; 410
length is 404aa with 11 gaps, 1 stops, absolute frame=
Minus2"
CDS complement(62245..61035)

```
                     /colour="255 0 0"
                     /evidence=predicted
                     /translation="DELIGENPQPEdiLGESGLLKRLSKRLVERALAGELTHHLQQSS
                     NDQS-GDeNSNGPRNSRNGYSKKTVQSEQG-EMDLSIPRDRCG-EFEPVLVPKGQRRI
                     A-GLDEKIIALYARGMTTRDIRAQLVELYG--ANISEALISDVTNSVMEEVKDWRSRP
                     LDEV--YPIVYLDALYVNIKVNGQVSKRAVYVALAVTVEGNKELLGLWIGAAerEGAK
                     FWLSVLTDLKNRGTQDILIACCDGLKGFPQAIESVYPQTQVQVCIVHLIRNSLRHVPW
                     KESRAVAADLKPIYQAATLE-ESEAALDAFAH-KWDETYPGISQIWIRHWDNVAPLFQ
                     YPMAIRKVIYTTNAIESLNRSLRKVIKTKAVFPNEESVYKLMFLAMRNISKR-WTRPI
                     LNWKAALSHFAILFPTRFNYXIH"
                     /note="IS256 e-value= 3.7e-167 complete sequence hit
                     coverage= 100%, between model( 410 aa) positions 1; 410
                     length is 404aa with 11 gaps, 1 stops, absolute frame=
                     Minus2"
     CDS             complement(6379401..6378635)
                     /colour="255 0 0"
                     /evidence=predicted
                     /translation="PCLGRFILYFTKLQLPPQKKYCDLEXWIISA---IILGYNGGEF
                     RXLIQPIIwLVLMPVAFRIALLSDIKNLV---------FPSILSXRLFEKXV----KL
                     SRYXsVNTQTLDRTXRAMPTAYDSDLTTLQWELLEPLIPAAKP-GGRPRT-TDMLSVL
                     NAIFYLVVTGCQWRQLPHDFPCWSTVYSYFRRWRDDGTWSQINEHLRMQVRVSE----
                     -------------------------DRHPSPSAAICDAQSVkvgnpRCHLIGFDGGKM
                     VKGRKRHVLVDTLGLVLMVMVTAANIS"
                     /note="IS5_IS427   e-value= 4.4e-06 complete sequence hit
                     coverage= 100%, between model( 296 aa) positions 1; 296
                     length is 256aa with 47 gaps, 6 stops, absolute frame=
                     Minus3"
     CDS             complement(6379161..6378293)
                     /colour="255 0 0"
                     /evidence=predicted
                     /translation="EKXVKLSRYXSVNTQTLDRTXramptaYDSDLTTLQWELLEPLI
                     PAAKPG-----GRPRTTDML--------------------SVLNAIFYLVVTGCQWRQ
                     --LPHDFPCWSTVYSYFRRWRDDGTWSQINEHLRMQVRVSED---RHPSPSAAICDAQ
                     SVK------------------------------VGNpRcHLIGFD--------GGKM-
                     --------------------------------------------VKGRKRHVLVD-TL
                     GLVLMVMVTAANISDQRGAK----------------------ILFWKAQRQGASLSRL
                     VR----IWADAGYQGQALMKWVMDRFQYVLEVVKRSDNLAGFQVIPKRWIVERTFGWL
                     LWSRRLNKDYEVLTRTAEALVYYVAMIRLMVRRLAQEHX"
                     /note="IS5_IS5     e-value= 1.8e-09 complete sequence hit
                     coverage= 100%, between model( 422 aa) positions 1; 422
                     length is 290aa with 140 gaps, 4 stops, absolute frame=
                     Minus3"
     CDS             complement(6379122..6378272)
                     /colour="255 0 0"
                     /evidence=predicted
                     /translation="TQTLDRTXRAM-----PTAYDSDLTTLQWELLEPLIPAAK----
                     ---PGGRPRTTDMLSVLNAIFYLVVTGCQWRQLPHDFPCWSTVYSYFRRWRDDGTWSQ
                     INEHLRMQVRVSEDRHPSPSAAICDAQSVKVGNPRCH-LIGFDGGKMVKGRKRHVLVD
                     TLGLVLMVMVTAANISDQR-GAKILFWKAQRQGaslSRLVRIWADAGYQGQALMKWVM
                     DRFQYVLEVVKRSDNLAGFQVIPKRWIVERTFGWLLWSRRLNKDYEVLTRTAEALVYV
                     AMIRLMVRRLAQEHXNFSNSLL"
                     /note="IS5_IS1031  e-value= 3.6e-111 complete sequence hit
                     coverage= 100%, between model( 295 aa) positions 1; 295
                     length is 284aa with 14 gaps, 2 stops, absolute frame=
                     Minus3"
     CDS             complement(6379080..6378272)
                     /colour="255 0 0"
                     /evidence=predicted
                     /translation="YDSDLTTLQWELLEPLIPAAK-------PGGRPRTTDMLSVLNA
                     IFYLVVTGCQWRQLPHDFPCWSTVYSYFRRWRDDGTWSQINEHLRMQVRVSEDRHPSP
```

```
                     SAAICDAQSVKVGNPRCH-LIGFDGGKMVKGRKRHVLVDTLGLVLMVMVTAANISDQR
                     -GAKILFWKAQRQGaslSRLVRIWADAGYQGQALMKWVMDRFQYVLEVVKRSDNLAGF
                     QVIPKRWIVERTFGWLLWSRRLNKDYEVLTRTAEALVYVAMIRLMVRRLAQEHXNFSN
                     SLL"
                     /note="IS5_IS1031  e-value= 2.4e-113 fragment hit
                     coverage= 93.56%, between model( 295 aa) positions 20;
                     295 length is 270aa with 9 gaps, 1 stops, absolute frame=
                     Minus3"
     CDS             complement(6379071..6378833)
                     /colour="255 0 0"
                     /evidence=predicted
                     /translation="DLTTLQWELLEPLIPAAKP-GGRPRT-TDMLSVLNAIFYLVVTG
                     CQWRQLPHDFPCWSTVYSYFRRWRDDGTWSQINEHLRM"
                     /note="IS5_IS427   e-value= 2e-19 fragment hit coverage=
                     27.70%, between model( 296 aa) positions 125; 206 length
                     is 80aa with 2 gaps, 0 stops, absolute frame= Minus3"
     CDS             complement(6361275..6360608)
                     /colour="255 0 0"
                     /evidence=predicted
                     /translation="QRFIQGLSPETIHLLSRIHRHSHHHQVRQRAHCILLSFEGFNVT
                     ELMSIFAVTRKTVYTWLDAWDNHC---LVGLYDQPGRGRKPKLRDVQ-KEQIREWAKM
                     TP---------HNLNVVLAKIKEAWNIEVSKTTLKRILKSCSMSWRRLRRVVAGQPDP
                     VEYATK-----RHQLEVLKRQEEKGELdlrYLDESGFCLVPYVPY-AWQEKG-ETLGL
                     PSQRSGRFNVLGLMNRHNDLTSYVF"
                     /note="IS630  e-value= 4.6e-12 fragment hit coverage=
                     63.83%, between model( 376 aa) positions 1; 240 length is
                     223aa with 20 gaps, 0 stops, absolute frame= Minus3"
     CDS             complement(6361275..6360251)
                     /colour="255 0 0"
                     /evidence=predicted
                     /translation="QRFIQGLSPETIHLLSRIHRHSHHHQVRQRAHCILLSFEGFNVT
                     ELMSIFAVTRKTVYTWLDAWDNHC---LVGLYDQPGRGRKPKLRDVQ-KEQIREWAKM
                     TP---------HNLNVVLAKIKEAWNIEVSKTTLKRILKSCSMSWRRLRRVVAGQPDP
                     VEYATK-----RHQLEVLKRQEEKGELdlrYLDESGFCLVPYVPY-AWQEKG------
                     --------ETLGLPSQRSGRFNVLGLMNRHNDLtSYVFDKSITSAVVVACIDDFSQTC
                     DQHT----VVVMDQASIHKNTEIEENIEDWKAkNVEIFWLPTYSPHLNLIEIFWRFMK
                     YEWLEFDAYKCLGSLSLYIDKILKGFGR--DYVIDFGXVLSVLLFGY"
                     /note="IS630  e-value= 3.7e-16 complete sequence hit
                     coverage= 100%, between model( 376 aa) positions 1; 376
                     length is 342aa with 39 gaps, 1 stops, absolute frame=
                     Minus3"
     CDS             complement(6360546..6360377)
                     /colour="255 0 0"
                     /evidence=predicted
                     /translation="DQHTVVVMDQASIHKNTEIEENIEDWKAkNVEIFWLPTYSPHLN
                     LIEIFWRFMKYEW"
                     /note="IS630  e-value= 1.9e-12 fragment hit coverage=
                     14.89%, between model( 376 aa) positions 277; 332 length
                     is 57aa with 0 gaps, 0 stops, absolute frame= Minus3"
     CDS             complement(6350184..6349745)
                     /colour="255 0 0"
                     /evidence=predicted
                     /translation="-RNVTXEAKSYDMGLHHIVFIPKRRRRVIYGSLRIRLGVLFRDL
                     AEQRDVEILEGHLLPDHVHMCMSIPPKHAVSGVVGFMKGKSAIAIARQFMGKRRNYSG
                     ESFWAXGYYVSTVDLYEGMVRAYIRNQELEDARLEQLSLPLGX----CXR"
                     /note="IS605  e-value= 1e-36 complete sequence hit
                     coverage= 100%, between model( 152 aa) positions 1; 152
                     length is 147aa with 5 gaps, 4 stops, absolute frame=
                     Minus3"
     CDS             complement(6350172..6349730)
```

```
                     /colour="255 0 0"
                     /evidence=predicted
                     /translation="XEAKSYDMGLH----HIVFIPKRRRRVIYGSLRIRLGVLFR-DL
                     AEQRDVEILEGHLLPDHVHMCMSIPPKHAVSGVVGFMKGKSAIAIARQFMGKRRNYSG
                     ESFWAXGYYVSTVD-LYEGMVRAYIRNQELEDARLEQLSLPLGXCXRVAPWG"
                     /note="IS200 e-value= 2.5e-35 complete sequence hit
                     coverage= 100%, between model( 154 aa) positions 1; 154
                     length is 148aa with 6 gaps, 4 stops, absolute frame=
                     Minus3"
     CDS             complement(6350172..6349733)
                     /colour="255 0 0"
                     /evidence=predicted
                     /translation="XEAKSYDMGLH----HIVFIPKRRRRVIYGS-LRIRLGVLFRDL
                     AEQRDVEILEGHLLPDHVHMCMSIPPKHAVSGVVGFMKGKSAIAIARQFMGKRRNYSG
                     ESFWAXGYYVSTVD-LYEGMVRAYIRNQELEDARLEQLSLPLGXCXRVAPW"
                     /note="IS200_IS605 e-value= 8.1e-37 complete sequence hit
                     coverage= 100%, between model( 153 aa) positions 1; 153
                     length is 147aa with 6 gaps, 4 stops, absolute frame=
                     Minus3"
     CDS             complement(6350139..6349763)
                     /colour="255 0 0"
                     /evidence=predicted
                     /translation="HIVFIPKRRRRVIYGSLRIRLGVLFR-DLAEQRDVEILEGHLLP
                     DHVHMCMSIPPKHAVSGVVGFMKGKSAIAIARQFMGKRRNYSGESFWAXGYYVSTVD-
                     LYEGMVRAYIRNQELEDARLEQLSLP"
                     /note="IS200 e-value= 1.2e-40 fragment hit coverage=
                     83.12%, between model( 154 aa) positions 16; 143 length
                     is 126aa with 2 gaps, 1 stops, absolute frame= Minus3"
     CDS             complement(6350139..6349760)
                     /colour="255 0 0"
                     /evidence=predicted
                     /translation="HIVFIPKRRRRVIYGS-LRIRLGVLFRDLAEQRDVEILEGHLLP
                     DHVHMCMSIPPKHAVSGVVGFMKGKSAIAIARQFMGKRRNYSGESFWAXGYYVSTVD-
                     LYEGMVRAYIRNQELEDARLEQLSLPL"
                     /note="IS200_IS605 e-value= 7.4e-40 fragment hit
                     coverage= 84.31%, between model( 153 aa) positions 16;
                     144 length is 127aa with 2 gaps, 1 stops, absolute frame=
                     Minus3"
     CDS             complement(6350139..6349760)
                     /colour="255 0 0"
                     /evidence=predicted
                     /translation="HIVFIPKRRRRVIYGSLRIRLGVLFRDLAEQRDVEILEGHLLPD
                     HVHMCMSIPPKHAVSGVVGFMKGKSAIAIARQFMGKRRNYSGESFWAXGYYVSTVDLY
                     EGMVRAYIRNQELEDARLEQLSLPL"
                     /note="IS605 e-value= 2.1e-41 fragment hit coverage=
                     83.55%, between model( 152 aa) positions 17; 143 length
                     is 127aa with 0 gaps, 1 stops, absolute frame= Minus3"
     CDS             complement(6347595..6346655)
                     /colour="255 0 0"
                     /evidence=predicted
                     /translation="TKNRXPMSYQIRNWSEYNAGLKQRGSVTFWLEQS--AIVHWLET
                     TPRQKRGASLTYSDTAISTFETVKCIYGLAGRQTEGFLNSLFELMGIELPVCDHSTVS
                     RRKGQLSISLPVIPKQGAIHVVIDSTGVKVYG------------EGEWKTRQHGVS-K
                     RRTWRKLHLSVDESTGEILTGVVTTNDVQDGEVFEDLLEGIDD-----EIEQVSAD-G
                     AYDQSHCYDALMERNATAAIPPRKNA-------------KIWQHGNC--NApphpRDQ
                     NLRAIRKQGRKRWKKQAHYHRRSLAETTMFRFKTIFGGQVRSRNFDNQATELLLQCAA
                     LN-RMIQIAKPETVWVE"
                     /note="IS5_IS903   e-value= 1.1e-96 complete sequence hit
                     coverage= 100%, between model( 347 aa) positions 1; 347
                     length is 314aa with 37 gaps, 1 stops, absolute frame=
```

Minus3"
CDS complement(6347595..6346655)
/colour="255 0 0"
/evidence=predicted
/translation="TKNRXPMSYQIRNWSEYNAGLKQRGSVTFWLEQS--AIVHWLET
TPRQKRGASLTYSDTAISTFETVKCIYGLAGRQTEGFLNSLFELMGIELPVCDHSTVS
RRKGQLSISLPVIPKQGAIHVVIDSTGVKVYG------------EGEWKTRQHGVS-K
RRTWRKLHLSVDESTGEILTGVVTTNDVQDGEVFEDLLEGIDD-----EIEQVSAD-G
AYDQSHCYDALMERNATAAIPPRKNA-------------KIWQHGNC--NApphpRDQ
NLRAIRKQGRKRWKKQAHYHRRSLAETTMFRFKTIFGGQVRSRNFDNQATELLLQCAA
LN-RMIQIAKPETVWVE"
/note="IS5_IS903 e-value= 2.2e-96 complete sequence hit
coverage= 100%, between model(347 aa) positions 1; 347
length is 314aa with 37 gaps, 1 stops, absolute frame=
Minus3"
CDS complement(6338160..6337466)
/colour="255 0 0"
/evidence=predicted
/translation="FKHLHLGMIsDIKRKSLPAIARAVGLENHQSLHHFLSESPWVAS
hLSDkRLDIILKILDGRRLILLID-----------------ETGDCKKGKSTDYVKR
QYIGNVGKKENGIvAVTAYGLFQGMILPLSFEVYKPR---------ErLKEEDEYLSK
PQIAAGMIRQLQGMGFCFELVLADSLYGEAQTNFVNVLEELKLPYILAIRSNHAVWLP
AEQAVFAEPWQSFERTFSNGTTETRYrQEIIYGqrhhQKRY"
/note="IS4 e-value= 4.7e-14 fragment hit coverage=
51.23%, between model(486 aa) positions 1; 249 length is
232aa with 27 gaps, 0 stops, absolute frame= Minus3"
CDS complement(6338160..6337034)
/colour="255 0 0"
/evidence=predicted
/translation="FKHLHLGMIsDIKRKSLPAIARAVGLENHQSLHHFLSESPWVAS
hLSDkRLDIILKILDGRRLILLID-----------------ETGDCKKGKSTDYVKR
QYIGNVGKKENGIvAVTAYGLFQGMILPLSFEVYKPR---------ErLKEEDEYLSK
PQIAAGMIRQLQGMGFCFELVLADSLYGEAQTNFVNVLEELKLPYILAIRSNHAVWLP
AEQAVFAEPWQSFERTFSNGT-------------------------------TETRYR
QEIIYGQRHHQKRyWLLTTDPQTLPENSTS----------------------YVMAA
APE----IKLDEIGDCYGFRTWIEYGLKQSKD-TLGWADFRMTHYEQIEKWWEIVMSA
FLMVSLFADVFNDS-------------------------CPVAHQHFAQHPwWDNQNG
WKNLLNNVR--LIIQPLIS----WNLLKRWLEVFPSRALKKGF"
/note="IS4 e-value= 9.9e-07 complete sequence hit
coverage= 100%, between model(486 aa) positions 1; 486
length is 376aa with 117 gaps, 0 stops, absolute frame=
Minus3"
CDS complement(6335550..6334313)
/colour="255 0 0"
/evidence=predicted
/translation="IRIPLNLPDI--RVLELSKTERGDWL------IKIESTLQGTTC
HQCGREITDL-HCHDQ-PFRIRHLPLFEVPVYLEIRPKRYRCKYCDDhpTTTQHLEWH
EPRSPNTKAYERWLLRILIN-STVSDVARKLGVSEDIVSGIIDRWIAAQVDWSEYADL
KVMGMDEISLKRG--HR---DFVVLITIPTTdgVDILAVLADRKQQTVANFLQSIP-I
DLRQTIERVCTDMYQGFVSAVREQLPQAKIVIDRFHVTKAYRNCADTVRKREVKRLRR
ELS------------KQEYD-----------SIKGAMWPFRKRPENLKES-EQQLLER
LFA--YSPQLKQAYNLREKLTQIFEGRYTKHGAKcAIRAWCKQVlKSGIKEFDSFLTT
INNWMDEMTNYFLEG-WTSGFVEGFNNRVKVLKRRCYGIFDIERLFQRISLDLNGYQT
FAVTXTL"
/note="ISL3 e-value= 1.2e-45 complete sequence hit
coverage= 100%, between model(451 aa) positions 1; 451
length is 413aa with 44 gaps, 1 stops, absolute frame=
Minus3"
CDS complement(6335391..6334313)
/colour="255 0 0"

```
                     /evidence=predicted
                     /translation="RIRHLPLFEVPVYLEIRPKRYRCKYCDDhpTTTQHLEWHEPRSP
                     NTKAYERWLLRILIN-STVSDVARKLGVSEDIVSGIIDRWIAAQVDWSEYADLKVMGM
                     DEISLKRG--HR---DFVVLITIPTTdgVDILAVLADRKQQTVANFLQSIP-IDLRQT
                     IERVCTDMYQGFVSAVREQLPQAKIVIDRFHVTKAYRNCADTVRKREVKRLRRELS--
                     ----------KQEYD-----------SIKGAMWPFRKRPENLKES-EQQLLERLFA--
                     YSPQLKQAYNLREKLTQIFEGRYTKHGAKcAIRAWCKQVlKSGIKEFDSFLTTINNWM
                     DEMTNYFLEG-WTSGFVEGFNNRVKVLKRRCYGIFDIERLFQRISLDLNGYQTFAVTX
                     TL"
                     /note="ISL3  e-value= 8.8e-52 fragment hit coverage=
                     86.03%, between model( 451 aa) positions 64; 451 length
                     is 360aa with 34 gaps, 1 stops, absolute frame= Minus3"
     CDS             complement(6315477..6314273)
                     /colour="255 0 0"
                     /evidence=predicted
                     /translation="TCSFFLTSXVSAIESGLLKRLSKRLVERALAGELTHHLQQSSND
                     QS-GDeNSNGPRNSRNGYSKKTVQSEQG-EMDLSIPRDRCG-EFEPVLVPKGQRRIA-
                     GLDEKIIALYARGMTTRDIRAQLVELYG--ANISEALISDVINSVMEEVKDWRSRPLD
                     EV--YPIVYLDALYVNIKVNGQVSKRAVYVALAVTVEGNKELLGLWIGAAerEGAKFW
                     LSVLTDLKNRGTQDILIACCDGLKGFPQAIESVYPQTQVQVCIVHLIRNSLRHVPWKE
                     SRAVAADLKPIYQAATLE-ESEAALDAFAH-KWDETYPGISQIWIRHWDNVAPLFQYP
                     MAIRKVIYTTNAIESLNRSLRKVIKTKAVFPNEESVYKLMFLAMRNISKR-WTRPILN
                     WKAALSHFAILFPTRFNYXIH"
                     /note="IS256  e-value= 6.6e-165 complete sequence hit
                     coverage= 100%, between model( 410 aa) positions 1; 410
                     length is 402aa with 11 gaps, 2 stops, absolute frame=
                     Minus3"
     CDS             complement(6315435..6314273)
                     /colour="255 0 0"
                     /evidence=predicted
                     /translation="SGLLKRLSKRLVERALAGELTHHLQQSSNDQS-GDeNSNGPRNS
                     RNGYSKKTVQSEQG-EMDLSIPRDRCG-EFEPVLVPKGQRRIA-GLDEKIIALYARGM
                     TTRDIRAQLVELYG--ANISEALISDVINSVMEEVKDWRSRPLDEV--YPIVYLDALY
                     VNIKVNGQVSKRAVYVALAVTVEGNKELLGLWIGAAerEGAKFWLSVLTDLKNRGTQD
                     ILIACCDGLKGFPQAIESVYPQTQVQVCIVHLIRNSLRHVPWKESRAVAADLKPIYQA
                     ATLE-ESEAALDAFAH-KWDETYPGISQIWIRHWDNVAPLFQYPMAIRKVIYTTNAIE
                     SLNRSLRKVIKTKAVFPNEESVYKLMFLAMRNISKR-WTRPILNWKAALSHFAILFPT
                     RFNYXIH"
                     /note="IS256  e-value= 1.1e-164 fragment hit coverage=
                     96.59%, between model( 410 aa) positions 15; 410 length
                     is 388aa with 11 gaps, 1 stops, absolute frame= Minus3"
     CDS             complement(6239001..6238595)
                     /colour="255 0 0"
                     /evidence=predicted
                     /translation="MLLVRPISTESLRLLHRIYHSSRHHQVRQRAHCLILFAQGWPPY
                     TLASLFSVSPKTVYNWLKAWNNRG---FAGLYNHPGRGRKPMFNPDQQQQIYEWT-QA
                     SPIQ---------LNQVLAQIEQQWSVRVSKATVKRVLKQMDMSWHR"
                     /note="IS630  e-value= 8.7e-15 fragment hit coverage=
                     39.63%, between model( 376 aa) positions 1; 149 length is
                     136aa with 13 gaps, 0 stops, absolute frame= Minus3"
     CDS             complement(6238941..6238604)
                     /colour="255 0 0"
                     /evidence=predicted
                     /translation="SSRHHQVRQRAHCLILFAQGWPPYTLASLFSVSPKTVYNWLKAW
                     NNRGFAGLYNH---PGRGRKPMfNPDQQQQIYEWTQASPIQLNQVLAQIEQQWSVR--
                     -VSKATVKRVLKQMDMS"
                     /note="IS481  e-value= 3.5e-09 fragment hit coverage=
                     33.62%, between model( 351 aa) positions 1; 118 length is
                     113aa with 6 gaps, 0 stops, absolute frame= Minus3"
     CDS             complement(6207357..6205703)
```

```
                     /colour="255 0 0"
                     /evidence=predicted
                     /translation="FQQFLPCQDTLHLDSWDLDSTNHQMT---------FHVSSTQAL
                     ACCPVCHQTSHRVHSRYERTLRDLPCVEFCLTILLQVCKFFCsnKTCKRRIfterlpQ
                     VAVPWARRTVRFAEHLSSIGLALGGAAAARLSYQINYGSSRNT----MLRALFKLPLP
                     -SVTTPKILGVDDFALRRG--HEYGTILVDLEKH----QPIVLLTDRKADTLADWLNE
                     HP------GVEVISRDRSKAYKSGASEGAPDALQVADRFHLLQNLEEILENVF-----
                     -------------------SSHSQ----VIKTVEYTLLKATLAEHQDTEAMSQPvvkp
                     qvdpnsrkaqnrarrlekyeqthalrkqgylikdiahhlgigkrtvytylaadtfqey
                     kthprrgwsglnpykaylleqwnkgrqnskqllaeiqqqgfkgsytmvvrythklrqs
                     lppqpprdslnelpgrgpvpeaqeliqkpltvqraawlvmrkvenlTEEDETILE-QL
                     SSQPELSKAIDLAQSLLFIVR-KRLPQHLDPWLDRAKN--SALKPFQSFAKGLLDDYE
                     AVKA-ALTLEVSNGQVEGQNNRLK--------------MVKRQMYGRAGLDLLNKRLV
                     L"
                     /note="ISL3  e-value= 9.2e-10 complete sequence hit
                     coverage= 100%, between model( 451 aa) positions 1; 451
                     length is 552aa with 73 gaps, 0 stops, absolute frame=
                     Minus3"
     CDS             complement(6206910..6206654)
                     /colour="255 0 0"
                     /evidence=predicted
                     /translation="TTPKILGVDDFALRRG--HEYGTILVDLEKH----QPIVLLTDR
                     KADTLADWLNEHP------GVEVISRDRSKAYKSGASEGAPDALQVADRFHLLQ"
                     /note="ISL3  e-value= 1.5e-21 fragment hit coverage=
                     21.73%, between model( 451 aa) positions 156; 253 length
                     is 86aa with 12 gaps, 0 stops, absolute frame= Minus3"
     CDS             complement(6206475..6206177)
                     /colour="255 0 0"
                     /evidence=predicted
                     /translation="RLEKYEQTHALRKQGYLIKDIAHHLGIGKRTVYTYLAADTFQEY
                     KTHPRR-------GWSGLNPYKAYLLEQWNKGRQNSKQ--------LLAEIQQQGFKG
                     SYTMVVRYTHKLR"
                     /note="IS21_ORF1   e-value= 2.7e-14 fragment hit coverage=
                     21.90%, between model( 525 aa) positions 1; 115 length is
                     100aa with 15 gaps, 0 stops, absolute frame= Minus3"
     CDS             complement(6206010..6205742)
                     /colour="255 0 0"
                     /evidence=predicted
                     /translation="LEQLSSQPELSKAIDLAQSLLFIVR-KRLPQHLDPWLDRAKN--
                     SALKPFQSFAKGLLDDYEAVKA-ALTLEVSNGQVEGQNNRLKMVKRQMYG"
                     /note="ISL3  e-value= 1.6e-06 fragment hit coverage=
                     20.84%, between model( 451 aa) positions 331; 424 length
                     is 90aa with 4 gaps, 0 stops, absolute frame= Minus3"
     CDS             complement(6205539..6203885)
                     /colour="255 0 0"
                     /evidence=predicted
                     /translation="FQQFLPCQDTLHLDSWDLDSTNHQMT---------FHVSSTQAL
                     ACCPVCHQTSHRVHSRYERTLRDLPCVEFCLTILLQVCKFFCsnKTCKRRIfterlpQ
                     VAVPWARRTVRFAEHLSSIGLALGGAAAARLSYQINYGSSRNT----MLRALFKLPLP
                     -SVTTPKILGVDDFALRRG--HEYGTILVDLEKH----QPIVLLTDRKADTLADWLNE
                     HP------GVEVISRDRSKAYKSGASEGAPDALQVADRFHLLQNLEEILENVF-----
                     -------------------SSHSQ----VIKTVEYTLLKATLAEHQDTEAMSQPvvkp
                     qvdpnsrkaqnrarrlekyeqthalrkqgylikdiahhlgigkrtvytylaadtfqey
                     kthprrgwsglnpykaylleqwnkgrqnskqllaeiqqqgfkgsytmvvrythklrqs
                     lppqpprdslnelpgrgpvpeaqeliqkpltvqraawlvmrkvenlTEEDETILE-QL
                     SSQPELSKAIDLAQSLLFIVR-KRLPQHLDPWLDRAKN--SALKPFQSFAKGLLDDYE
                     AVKA-ALTLEVSNGQVEGQNNRLK--------------MVKRQMYGRAGLDLLNKRLV
                     L"
                     /note="ISL3  e-value= 9.2e-10 complete sequence hit
                     coverage= 100%, between model( 451 aa) positions 1; 451
```

```
                     length is 552aa with 73 gaps, 0 stops, absolute frame=
                     Minus3"
     CDS             complement(6205092..6204836)
                     /colour="255 0 0"
                     /evidence=predicted
                     /translation="TTPKILGVDDFALRRG--HEYGTILVDLEKH----QPIVLLTDR
                     KADTLADWLNEHP------GVEVISRDRSKAYKSGASEGAPDALQVADRFHLLQ"
                     /note="ISL3  e-value= 1.5e-21 fragment hit coverage=
                     21.73%, between model( 451 aa) positions 156; 253 length
                     is 86aa with 12 gaps, 0 stops, absolute frame= Minus3"
     CDS             complement(6204657..6204359)
                     /colour="255 0 0"
                     /evidence=predicted
                     /translation="RLEKYEQTHALRKQGYLIKDIAHHLGIGKRTVYTYLAADTFQEY
                     KTHPRR-------GWSGLNPYKAYLLEQWNKGRQNSKQ--------LLAEIQQQGFKG
                     SYTMVVRYTHKLR"
                     /note="IS21_ORF1   e-value= 2.7e-14 fragment hit coverage=
                     21.90%, between model( 525 aa) positions 1; 115 length is
                     100aa with 15 gaps, 0 stops, absolute frame= Minus3"
     CDS             complement(6204192..6203924)
                     /colour="255 0 0"
                     /evidence=predicted
                     /translation="LEQLSSQPELSKAIDLAQSLLFIVR-KRLPQHLDPWLDRAKN--
                     SALKPFQSFAKGLLDDYEAVKA-ALTLEVSNGQVEGQNNRLKMVKRQMYG"
                     /note="ISL3  e-value= 1.6e-06 fragment hit coverage=
                     20.84%, between model( 451 aa) positions 331; 424 length
                     is 90aa with 4 gaps, 0 stops, absolute frame= Minus3"
     CDS             complement(6061491..6061148)
                     /colour="255 0 0"
                     /evidence=predicted
                     /translation="KYIVTLTPEERSELIQLTRRRTLSARKMKRAQILMLADEGHKDD
                     TITQMLNAGISTVHRTRQKFVEGG---VEfALNERPRPGGQKKLDSKAEALLIATACS
                     DPPTG----CCRWTMQLLAE"
                     /note="IS630  e-value= 1.2e-15 fragment hit coverage=
                     32.18%, between model( 376 aa) positions 1; 121 length is
                     115aa with 7 gaps, 0 stops, absolute frame= Minus3"
     CDS             complement(5745174..5744480)
                     /colour="255 0 0"
                     /evidence=predicted
                     /translation="FKHLHLGMIsDIKRKSLPAIARAVGLENHQSLHHFLSESPWVAS
                     hLSDkRLDIILKILDGRRLILLID------------------ETGDCKKGKSTDYVKR
                     QYIGNVGKKENGIvAVTAYGLFQGMILPLSFEVYKPR---------ErLKEEDEYLSK
                     PQIAAGMIRQLQGMGFCFELVLADSLYGEAQTNFVNVLEELKLPYILAIRSNHAVWLP
                     AEQAVFAEPWQSFERTFSNGTTETRYrQEIIYGqrhhQKRY"
                     /note="IS4   e-value= 4.7e-14 fragment hit coverage=
                     51.23%, between model( 486 aa) positions 1; 249 length is
                     232aa with 27 gaps, 0 stops, absolute frame= Minus3"
     CDS             complement(5745174..5744048)
                     /colour="255 0 0"
                     /evidence=predicted
                     /translation="FKHLHLGMIsDIKRKSLPAIARAVGLENHQSLHHFLSESPWVAS
                     hLSDkRLDIILKILDGRRLILLID------------------ETGDCKKGKSTDYVKR
                     QYIGNVGKKENGIvAVTAYGLFQGMILPLSFEVYKPR---------ErLKEEDEYLSK
                     PQIAAGMIRQLQGMGFCFELVLADSLYGEAQTNFVNVLEELKLPYILAIRSNHAVWLP
                     AEQAVFAEPWQSFERTFSNGT------------------------------TETRYR
                     QEIIYGQRHHQKRyWLLTTDPQTLPENSTS-----------------------YVMAA
                     APE----IKLDEIGDCYGFRTWIEYGLKQSKD-TLGWADFRMTHYEQIEKWWEIVMSA
                     FLMVSLFADVFNDS------------------------CPVAHQHFAQHPwWDNQNG
                     WKNLLNNVR--LIIQPLIS----WNLLKRWLEVFPSRALKKGF"
                     /note="IS4   e-value= 9.9e-07 complete sequence hit
```

coverage= 100%, between model(486 aa) positions 1; 486
length is 376aa with 117 gaps, 0 stops, absolute frame=
Minus3"
CDS complement(5719893..5719670)
/colour="255 0 0"
/evidence=predicted
/translation="SQMSTLTCPNCRSQN-VVKNGRIHNGKQNHKCKTCGRQFV--EA
PQQKRIDSSTKGLIDKLLLEKIPLAGIARVCDVS"
/note="IS1_ORF1 e-value= 3.6e-14 fragment hit coverage=
32.64%, between model(239 aa) positions 1; 78 length is
75aa with 3 gaps, 0 stops, absolute frame= Minus3"
CDS complement(5522628..5522375)
/colour="255 0 0"
/evidence=predicted
/translation="HIEFSyplMQCPLCGHPK-IYKHGKTSKGSQRXRCPHCHQTFS-
DtfDTLYYRRQiSPQTIQVILQSHAEGSSFRGLPRITGVAYNT"
/note="IS1_ORF1 e-value= 9e-13 fragment hit coverage=
33.89%, between model(239 aa) positions 1; 81 length is
85aa with 2 gaps, 1 stops, absolute frame= Minus3"
CDS complement(5391534..5391422)
/colour="255 0 0"
/evidence=predicted
/translation="KDYERXTDSTEVMIYICMIRLMVRRVAXTLRRWSLFTH"
/note="IS5_IS1031 e-value= 2.7e-08 fragment hit coverage=
12.88%, between model(295 aa) positions 258; 295 length
is 38aa with 0 gaps, 2 stops, absolute frame= Minus3"
CDS complement(5338785..5338049)
/colour="255 0 0"
/evidence=predicted
/translation="-----TGVYKRTFkpMLHAWHTYHLSRSN--AGRPPKLCRCDQL
LVALQYWREYRTYFHIAGDWEVSESTVCRIVHQVETALMNSGLFRLPGQKSLLQGFER
PDVVVMDVTETPIERPQTRQKAYYSGKKRDIPSNARLSLTATLXrlsaltLVQVVG--
-------MIFRSSRVQVSISIQIPRVCKIADIKGLQ-----PIMP-TAMFLSRSHNTV
NXLPCSESITVLXVRNEWALNTLIAAXRFSEFCRSAIAIVVVATRCGVTX"
/note="IS5_ISL2 e-value= 3.1e-21 complete sequence hit
coverage= 100%, between model(260 aa) positions 1; 260
length is 246aa with 22 gaps, 5 stops, absolute frame=
Minus3"
CDS complement(5338725..5338415)
/colour="255 0 0"
/evidence=predicted
/translation="SRSNAGRPPKLCRCDQLLVALQYWREYRTYFHIAGDWEVSESTV
CRIVHQVETALMNSGLFRLPGQKSLLQGFERPDVVVMDVTETPIERPQTRQKAYYSGK
KR"
/note="IS5_ISL2 e-value= 9.3e-44 fragment hit coverage=
40%, between model(260 aa) positions 26; 129 length is
104aa with 0 gaps, 0 stops, absolute frame= Minus3"
CDS complement(5175534..5174819)
/colour="255 0 0"
/evidence=predicted
/translation="YLSPES-LQCRASSKVVSMXSTVGNPS---------ILARISDL
FPHCWRLGSLXINRLPYCppGRDRSDELRTLSLTRSEISATR---------VRATGCR
CDGCDXNAHXTSPDKTKSVLF----RKKKRHTFKCQIIANRNTLEIICLNVGPGRRHD
FQIFKGSGIHIHPDTESLQDSGYQGIAAYHANSYVPLKKPQHGELTSLQREYNRALSQ
ERMGIEHINRSLKIFRILSERYRNRRRRYALRCNLIAAIYNYEL"
/note="IS5_ISL2 e-value= 1.5e-39 complete sequence hit
coverage= 100%, between model(260 aa) positions 1; 260
length is 239aa with 23 gaps, 4 stops, absolute frame=
Minus3"
CDS complement(5175219..5174819)

```
                     /colour="255 0 0"
                     /evidence=predicted
                     /translation="KKKRHTFKCQIIANRNTLEIICLNVGPGRRHDFQIFKGSGIHIH
                     PDTESLQDSGYQGIAAYHANSYVPLKKPQHGELTSLQREYNRALSQERMGIEHINRSL
                     KIFRILSERYRNRRRRYALRCNLIAAIYNYEL"
                     /note="IS5_ISL2    e-value= 6.5e-62 fragment hit coverage=
                     51.54%, between model( 260 aa) positions 127; 260 length
                     is 134aa with 0 gaps, 0 stops, absolute frame= Minus3"
     CDS             complement(5122416..5122250)
                     /colour="255 0 0"
                     /evidence=predicted
                     /translation="SRRRAYPNDMSDTEWDVLRPLLPPANGFR-------RPRTVNLR
                     EILNAIFYVQRSGYQWEML"
                     /note="IS5_IS1031  e-value= 1.2e-20 fragment hit coverage=
                     21.36%, between model( 295 aa) positions 15; 77 length is
                     56aa with 7 gaps, 0 stops, absolute frame= Minus3"
     CDS             complement(4461825..4461482)
                     /colour="255 0 0"
                     /evidence=predicted
                     /translation="KYIVTLTPEERSELIQLTRRRTLSARKMKRAQILMLADEGHKDD
                     TITQMLNAGISTVHRTRQKFVEGG---VEfALNERPRPGGQKKLDSKAEALLIATACS
                     DPPTG----CCRWTMQLLAE"
                     /note="IS630  e-value= 1.2e-15 fragment hit coverage=
                     32.18%, between model( 376 aa) positions 1; 121 length is
                     115aa with 7 gaps, 0 stops, absolute frame= Minus3"
     CDS             complement(4252959..4252802)
                     /colour="255 0 0"
                     /evidence=predicted
                     /translation="HHPHPRVQRRMEVLYLKSQGLPHAQICQLCQISRPTLAKTLRLY
                     QQGGIEGLK"
                     /note="IS481  e-value= 1.1e-08 fragment hit coverage=
                     15.10%, between model( 351 aa) positions 1; 53 length is
                     53aa with 0 gaps, 0 stops, absolute frame= Minus3"
     CDS             complement(4219197..4219103)
                     /colour="255 0 0"
                     /evidence=predicted
                     /translation="AERG--ITLFELPSYSPHLNLIERLWQFMKYQWI"
                     /note="IS630  e-value= 1.8e-09 fragment hit coverage=
                     9.04%, between model( 376 aa) positions 300; 333 length
                     is 32aa with 2 gaps, 0 stops, absolute frame= Minus3"
     CDS             complement(4134504..4134368)
                     /colour="255 0 0"
                     /evidence=predicted
                     /translation="WQTDGXDGYSRQLPDE---VIHHVSKALTQRLERTNGILWQQTG
                     RWHRR"
                     /note="IS1_ORF2    e-value= 3.9e-08 fragment hit coverage=
                     20.68%, between model( 237 aa) positions 164; 212 length
                     is 46aa with 3 gaps, 1 stops, absolute frame= Minus3"
     CDS             complement(3873963..3873578)
                     /colour="255 0 0"
                     /evidence=predicted
                     /translation="YADESGMDHRDEYDY-AYGPKGE-----------RVYA-LKTG-
                     RRSGRVNMIAALRAKQL--MAPFTIEGACNRTVFELwLERCLIPvlKPGQKLVI--DN
                     ATFHKGGQIQELVEKAG--CEVWYLPPYSPDLNKIERSWSWIKSRIR"
                     /note="IS630  e-value= 1.1e-13 fragment hit coverage=
                     38.83%, between model( 376 aa) positions 188; 333 length
                     is 129aa with 20 gaps, 0 stops, absolute frame= Minus3"
     CDS             complement(3585738..3585497)
                     /colour="255 0 0"
                     /evidence=predicted
```

```
                     /translation="VKLAELIPWESFESEYADQFSQ---TMGAPAKPFRVALGTLIIK
                     EKLGISDAETVEQIRENPYLQYFLGFSEYRESAPFDASML"
                     /note="IS5_IS5     e-value= 2.6e-06 fragment hit coverage=
                     19.91%, between model( 422 aa) positions 23; 106 length
                     is 81aa with 3 gaps, 0 stops, absolute frame= Minus3"
CDS                  complement(3216468..3216083)
                     /colour="255 0 0"
                     /evidence=predicted
                     /translation="YADESGMDHRDEYDY-AYGPKGE-----------RVYA-LKTG-
                     RRSGRVNMIAALRAKQL--MAPFTIEGACNRTVFELwLERCLIPvlKPGQKLVI--DN
                     ATFHKGGQIQELVEKAG--CEVWYLPPYSPDLNKIERSWSWIKSRIR"
                     /note="IS630 e-value= 1.1e-13 fragment hit coverage=
                     38.83%, between model( 376 aa) positions 188; 333 length
                     is 129aa with 20 gaps, 0 stops, absolute frame= Minus3"
CDS                  complement(2709669..2709254)
                     /colour="255 0 0"
                     /evidence=predicted
                     /translation="IERLQTKHP--HQIVYVDEAGIDNRADYPY-GYCPVG-------
                     ----QRFYD-LKSG-KRTERVSFIAALKEGQL--FSPMIFEGSCNRLLFEAWLQQSLI
                     -SQLQLgdVIVIDNASFHHGQRIEEIVAEAG--CEIWYLPSYSPDLNKIERWWFVLKN
                     "
                     /note="IS630 e-value= 7.3e-15 fragment hit coverage=
                     42.02%, between model( 376 aa) positions 173; 330 length
                     is 139aa with 21 gaps, 0 stops, absolute frame= Minus3"
CDS                  complement(2412987..2412026)
                     /colour="255 0 0"
                     /evidence=predicted
                     /translation="LLRAIISSLyVSCWDWR-TSKFR------LTVPFDAXXRGLTSN
                     RXATNLKLGCSRKLRLTLPIIMQHPWMangFVRGSpMKTASSVLXDWXV------YSQ
                     WKQASPSSSKPS---------LRRIIAKS-KSSRLywkHYNSMACXSPWMP----YTP
                     KKT---------------L----------------QQVVASVN-DYLIAVKRNQGRL
                     YDHLQTYFeCLKPMAEHTHS-----IQSRGREEHRCIQVYEPVGI---ALQEWEAIRS
                     VLCVQRWGTRQGKAY----HNTAYYISSAATSPHH---WQSLVREHWGIENRLHWPKD
                     VVFGEDDYRLEDEQALLNWSVLRTIVINILRLNG--YQSLKTAMTKLANRVDIIFSLL
                     TXNSPA"
                     /note="ISAs1 e-value= 1.9e-08 complete sequence hit
                     coverage= 100%, between model( 389 aa) positions 1; 389
                     length is 321aa with 77 gaps, 7 stops, absolute frame=
                     Minus3"
CDS                  complement(2412552..2412110)
                     /colour="255 0 0"
                     /evidence=predicted
                     /translation="DYLIAVKRNQGRLYDHLQTYFeCLKPMAEHTHS-----IQSRGR
                     EEHRCIQVYEPVGI---ALQEWEAIRSVLCVQRWGTRQGKAY----HNTAYYISSAAT
                     SPHH---WQSLVREHWGIENRLHWPKDVVFGEDDYRLEDEQALLNWSVLRTIVINILR
                     LNG"
                     /note="ISAs1 e-value= 2.4e-27 fragment hit coverage=
                     41.65%, between model( 389 aa) positions 198; 359 length
                     is 148aa with 15 gaps, 0 stops, absolute frame= Minus3"
CDS                  complement(2404083..2403389)
                     /colour="255 0 0"
                     /evidence=predicted
                     /translation="FKHLHLGMIsDIKRKSLPAIARAVGLENHQSLHHFLSESPWVAS
                     hLSDkRLDIILKILDGRRLILLID------------------ETGDCKKGKSTDYVKR
                     QYIGNVGKKENGIvAVTAYGLFQGMILPLSFEVYKPR---------ErLKEEDEYLSK
                     PQIAAGMIRQLQGMGFCFELVLADSLYGEAQTNFVNVLEELKLPYILAIRSNHAVWLP
                     AEQAVFAEPWQSFERTFSNGTTETRYrQEIIYGqrhhQKRY"
                     /note="IS4   e-value= 4.7e-14 fragment hit coverage=
                     51.23%, between model( 486 aa) positions 1; 249 length is
                     232aa with 27 gaps, 0 stops, absolute frame= Minus3"
```

```
CDS             complement(2404083..2402957)
                /colour="255 0 0"
                /evidence=predicted
                /translation="FKHLHLGMIsDIKRKSLPAIARAVGLENHQSLHHFLSESPWVAS
                hLSDkRLDIILKILDGRRLILLID------------------ETGDCKKGKSTDYVKR
                QYIGNVGKKENGIvAVTAYGLFQGMILPLSFEVYKPR---------ErLKEEDEYLSK
                PQIAAGMIRQLQGMGFCFELVLADSLYGEAQTNFVNVLEELKLPYILAIRSNHAVWLP
                AEQAVFAEPWQSFERTFSNGT-------------------------------TETRYR
                QEIIYGQRHHQKRyWLLTTDPQTLPENSTS----------------------YVMAA
                APE----IKLDEIGDCYGFRTWIEYGLKQSKD-TLGWADFRMTHYEQIEKWWEIVMSA
                FLMVSLFADVFNDS------------------------CPVAHQHFAQHPwWDNQNG
                WKNLLNNVR--LIIQPLIS----WNLLKRWLEVFPSRALKKGF"
                /note="IS4   e-value= 9.9e-07 complete sequence hit
                coverage= 100%, between model( 486 aa) positions 1; 486
                length is 376aa with 117 gaps, 0 stops, absolute frame=
                Minus3"
CDS             complement(2382702..2382314)
                /colour="255 0 0"
                /evidence=predicted
                /translation="FFMDESGFSLVPCIPY-GWQPIG-------------TYL-EIPT
                RSSKRLNVLGFLSRRQGL--HAYTSEQTITSEVVShCIDTFFADV--ELPTVIVVDQA
                PIHTSQsiyEMKAEWAERG--ITLFELPSYSPHLNLIERLWQFMKYQWI"
                /note="IS630  e-value= 6.5e-11 fragment hit coverage=
                39.10%, between model( 376 aa) positions 187; 333 length
                is 130aa with 21 gaps, 0 stops, absolute frame= Minus3"
CDS             complement(2279970..2279714)
                /colour="255 0 0"
                /evidence=predicted
                /translation="SMECPCCQSQK-IHKRGFDSlqdgTLVQRYQCEDCNRRFK-ERT
                GNPMARLRTtslVVSYAIKARTKGMGVRATGRTFGTSHTTIMRW"
                /note="IS1_ORF1    e-value= 8.4e-08 fragment hit coverage=
                33.89%, between model( 239 aa) positions 5; 85 length is
                86aa with 2 gaps, 0 stops, absolute frame= Minus3"
CDS             complement(1504611..1503287)
                /colour="255 0 0"
                /evidence=predicted
                /translation="CDSDVTLDF----YQDHPLCVQFSDLDLSSDVGILLAAQAESRI
                HICQDIADCIEEWRDPDKLTHSLPQLVAQRVYQLIGGYEDANDSDSLRHDPIFKIACE
                YLPIPESnVLASQLTISRLENQVTTDQTAAMRRQFIDRFIA-----SYPHPPSTIVLD
                IDGWDDPTHGDQEGSAFHGYYGQHMYFPVLINE----AQSGFPLVCQLRRGNSHPGKA
                VAGILRWLFWRLKRAWPGVT-------------IVLRADAGFSLPEILRVCERSGIHY
                AIGFSSNAVLKRKISNV-LEQARLQYCRTQQKARL-----FDDVYYAAATWDYPRRLV
                MKAEYLPKGANPRFVLTD-MMLSPQQLYDTFYVQRGGDSEHpIKELKRGIQADRLSCH
                RFTANQFRLLLA-------------QAAYLLMITLRXAAQ-GTELATAQVERLRCALI
                KGAARVRVSVRRVLVELATFCPFEKEIRLIAQRLCDPMP"
                /note="IS1380 e-value= 2.1e-64 complete sequence hit
                coverage= 100%, between model( 487 aa) positions 1; 487
                length is 442aa with 47 gaps, 1 stops, absolute frame=
                Minus3"
CDS             complement(1504542..1503287)
                /colour="255 0 0"
                /evidence=predicted
                /translation="LSSDVGILLAAQAESRIHICQDIADCIEEWRDPDKLTHSLPQLV
                AQRVYQLIGGYEDANDSDSLRHDPIFKIACEYLPIPESnVLASQLTISRLENQVTTDQ
                TAAMRRQFIDRFIA-----SYPHPPSTIVLDIDGWDDPTHGDQEGSAFHGYYGQHMYF
                PVLINE----AQSGFPLVCQLRRGNSHPGKAVAGILRWLFWRLKRAWPGVT-------
                ------IVLRADAGFSLPEILRVCERSGIHYAIGFSSNAVLKRKISNV-LEQARLQYC
                RTQQKARL-----FDDVYYAAATWDYPRRLVMKAEYLPKGANPRFVLTD-MMLSPQQL
                YDTFYVQRGGDSEHpIKELKRGIQADRLSCHRFTANQFRLLLA-------------QA
                AYLLMITLRXAAQ-GTELATAQVERLRCALIKGAARVRVSVRRVLVELATFCPFEKEI
```

```
                     RLIAQRLCDPMP"
                     /note="IS1380 e-value= 1.5e-67 fragment hit coverage=
                     94.46%, between model( 487 aa) positions 28; 487 length
                     is 419aa with 43 gaps, 1 stops, absolute frame= Minus3"
     CDS             complement(691935..690725)
                     /colour="255 0 0"
                     /evidence=predicted
                     /translation="DELIGENPQPEdiLGESGLLKRLSKRLVERALAGELTHHLQQSS
                     NDQS-GDeNSNGPRNSRNGYSKKTVQSEQG-EMDLSIPRDRCG-EFEPVLVPKGQRRI
                     A-GLDEKIIALYARGMTTRDIRAQLVELYG--ANISEALISDVINSVMEEVKDWRSRP
                     LDEV--YPIVYLDALYVNIKVNGQVSKRAVYVALAVTVEGNKELLGLWIGAAerEGAK
                     FWLSVLTDLKNRGTQDILIACCDGLKGFPQAIESVYPQTQVQVCIVHLIRNSLRHVPW
                     KESRAVAADLKPIYQAATLE-ESEAALDAFAH-KWDETYPGISQIWIRHWDNVAPLFQ
                     YPMAIRKVIYTTNAIESLNRSLRKVIKTKAVFPNEESVYKLMFLAMRNISKR-WTRPI
                     LNWKAALSHFAILFPTRFNYXIH"
                     /note="IS256 e-value= 3.8e-165 complete sequence hit
                     coverage= 100%, between model( 410 aa) positions 1; 410
                     length is 404aa with 11 gaps, 1 stops, absolute frame=
                     Minus3"
     CDS             complement(691935..690725)
                     /colour="255 0 0"
                     /evidence=predicted
                     /translation="DELIGENPQPEdiLGESGLLKRLSKRLVERALAGELTHHLQQSS
                     NDQS-GDeNSNGPRNSRNGYSKKTVQSEQG-EMDLSIPRDRCG-EFEPVLVPKGQRRI
                     A-GLDEKIIALYARGMTTRDIRAQLVELYG--ANISEALISDVINSVMEEVKDWRSRP
                     LDEV--YPIVYLDALYVNIKVNGQVSKRAVYVALAVTVEGNKELLGLWIGAAerEGAK
                     FWLSVLTDLKNRGTQDILIACCDGLKGFPQAIESVYPQTQVQVCIVHLIRNSLRHVPW
                     KESRAVAADLKPIYQAATLE-ESEAALDAFAH-KWDETYPGISQIWIRHWDNVAPLFQ
                     YPMAIRKVIYTTNAIESLNRSLRKVIKTKAVFPNEESVYKLMFLAMRNISKR-WTRPI
                     LNWKAALSHFAILFPTRFNYXIH"
                     /note="IS256 e-value= 9.9e-166 complete sequence hit
                     coverage= 100%, between model( 410 aa) positions 1; 410
                     length is 404aa with 11 gaps, 1 stops, absolute frame=
                     Minus3"
     CDS             complement(558171..557924)
                     /colour="255 0 0"
                     /evidence=predicted
                     /translation="MQCPLCGHPK-THKHGKTSKGSQRYRCPHCHQTFS--ETFDTLH
                     YRRQISSEtiqtILQSHAEGSSLRGLSRITGVAYNTCVSVIR"
                     /note="IS1_ORF1    e-value= 2.1e-12 fragment hit coverage=
                     34.31%, between model( 239 aa) positions 6; 87 length is
                     83aa with 3 gaps, 0 stops, absolute frame= Minus3"
     CDS             complement(483540..483299)
                     /colour="255 0 0"
                     /evidence=predicted
                     /translation="VKLAELIPWESFESEYADQFSQ---TMGAPAKPFRVALGTLIIK
                     EKLGISDAETVEQIRENPYLQYFLGFSEYRESAPFDASML"
                     /note="IS5_IS5     e-value= 2.6e-06 fragment hit coverage=
                     19.91%, between model( 422 aa) positions 23; 106 length
                     is 81aa with 3 gaps, 0 stops, absolute frame= Minus3"
     CDS             complement(477669..477176)
                     /colour="255 0 0"
                     /evidence=predicted
                     /translation="LEKTGIESKKKTYGYR--ERD--ETQR---QAFIERLQTKHP--
                     HQIVYVDEAGIDNRADYPY-GYCPVG-----------QRFYD-LKSG-KRTERVSFIA
                     ALKEGQL--FSPMTFEGSCNRLLFEAWLQQSLI-SQLQLgdVIVIDNASFHHGQRIEE
                     IVAEAG--CEIWYLPSYSPDLNKIERWWFVLKN"
                     /note="IS630 e-value= 3e-16 fragment hit coverage=
                     50.80%, between model( 376 aa) positions 140; 330 length
                     is 165aa with 28 gaps, 0 stops, absolute frame= Minus3"
```

```
CDS             complement(474207..474032)
                /colour="255 0 0"
                /evidence=predicted
                /translation="GXHPIAQICQVLNYPRSQVYYHARGQP-----DESELKAAIAGV
                AGAYP-TYGYRRITAQLQ---RQG"
                /note="IS3_IS150_ORF2   e-value= 2.1e-06 fragment hit
                coverage= 17.48%, between model( 389 aa) positions 103;
                170 length is 59aa with 9 gaps, 1 stops, absolute frame=
                Minus3"
CDS             complement(474183..474032)
                /colour="255 0 0"
                /evidence=predicted
                /translation="CQVLNYPRSQVYYHARGQPDESELKAAIAGVAGAYPTYGYRRIT
                AQLQRQG"
                /note="IS3_IS407_ORF2   e-value= 1.2e-07 fragment hit
                coverage= 14.83%, between model( 344 aa) positions 85;
                135 length is 51aa with 0 gaps, 0 stops, absolute frame=
                Minus3"
CDS             complement(474147..474035)
                /colour="255 0 0"
                /evidence=predicted
                /translation="YHARgQPDESELKAAIAGVAGAYPTYGYRRITAQLQRQ"
                /note="IS3_IS2_ORF2     e-value= 9.4e-07 fragment hit
                coverage= 12.25%, between model( 302 aa) positions 48; 84
                length is 38aa with 0 gaps, 0 stops, absolute frame=
                Minus3"
CDS             complement(472965..471914)
                /colour="255 0 0"
                /evidence=predicted
                /translation="MSPLMMKTSVFE------GLGLGTQPRTTISHEERLVLSGNlsN
                XISVKFGTMDTTIMQGXigtrpLTTEDRMLRQTHKXTQARTHQYRIhxLKXRVFTGAK
                -----AAIQSLAELTQMVE------------NHKG--NECDDSLTLKQSVYFI-----
                -DCFSRLLXSKLK-----GYCVNHKRVARLMRQIGIMAKTKVK-RKRTTNSEHSfpRY
                GNRVLNLS--IDHPEQVWVADITYIRLQ-QEFVYLAVVMDVFTRAIRGWHLSRHIDQQ
                LTLRALNKALERAT---PEIHHS-----DQGVQYAAAAYMQLLQQHQVQISMAEVGQA
                WQNGYAERLMRTIKEEEVD--LSDYRNFTEAYEHIEQFLEdVYMHKRIHSSLGYLTPC
                EYEQQWRQQ"
                /note="IS3_IS150_ORF2   e-value= 4.3e-13 complete sequence
                hit coverage= 100%, between model( 389 aa) positions 1;
                389 length is 351aa with 50 gaps, 5 stops, absolute
                frame= Minus3"
CDS             complement(472827..471917)
                /colour="255 0 0"
                /evidence=predicted
                /translation="MDTTIMQGXIGTRplTTEDRMLRQTHKXTQARTHQYRIHXLKXR
                VFTGAKAAIQSLAELTQMVENhkgneCDDSLTLKQSVYFIDCFSRLLXSKlkGYCVNH
                KRVARLMRQIGIMAKTKVK----RKRTTNSEHSF-PRYGNRVlNLSIDHPEQVWVADI
                TYIRL--QQEFVYLAVVMDVFTRAIRGWHLSRHIDQQLTLRALNKALER----ATPEI
                HHSDQGVQYAAAAYMQLLQQHQVQISMAEVGQAWQNGYAERLMRTIKEEEVDLSDYRN
                FT--EAYEHIEQFLEDVYMHKRIHSSLGYLTPCEYEQQWRQ"
                /note="IS3_IS150_ORF1   e-value= 6.7e-29 complete sequence
                hit coverage= 100%, between model( 306 aa) positions 1;
                306 length is 304aa with 13 gaps, 5 stops, absolute
                frame= Minus3"
CDS             complement(472827..471890)
                /colour="255 0 0"
                /evidence=predicted
                /translation="MDTTIMQGXI------------GTRPLTTEDRMLRQTHKXT---
                QARTHQYRIHXLKXRVFTGAKA--AIQSLAELTQMVE--------------NHKGNEC
                DDSLTLKQSVYFI----DCF-SRLLXSKL--KGYCVNHKRVARLMRQIGIMAKTKV-K
```

```
                     RKRTtnsehsfprYGNRVLNLSIDHPEQVWVADITYIRLQ--QEFVYLAVVmDVFTRA
                     IRGWHLSRHIDQQLTLRALNKALE-RATPEIHHSDQGVQYAAAAYMQLLQQHQVQISM
                     AEVGQAWQNGYAERLMRTIKEEEVDLSDYRNFTEAYEHIEQFLEDvYMHKRIHSSLGY
                     LTPCEYEQQWRQQNNHYCMNK"
                     /note="IS3_IS407_ORF2    e-value= 9.2e-07 complete sequence
                     hit coverage= 100%, between model( 344 aa) positions 1;
                     344 length is 313aa with 42 gaps, 5 stops, absolute
                     frame= Minus3"
     CDS             complement(472785..471890)
                     /colour="255 0 0"
                     /evidence=predicted
                     /translation="LTTEDRmLRQTH------KXTQARTHQYRIHXLKXRVFTGAKAA
                     IQSLAELTQMVEN-HKGNECddsltlkqsVYFIDCFSRLLXSklKGYCVNHKRVARLM
                     RQIGIMAKTKVKRKRTTNSEHSFPRYGNRVL-NLSIDHPEQVWVADITYIRLQQEFVY
                     LAVVMDVFTRAIRGWHLSRHIDQQLT-LRALNKALERATPE-------IHHSDQGVQY
                     AAAAYMQLLQQHQVQISMAEVGQAWQNGYAERLMRTIKEEEVDL---S-DYRNFTEAY
                     EHIEQFLEDVYMHKRIHSSLGYLTPCEYEQQWRQQNNHYCMNK"
                     /note="IS3_IS51_ORF1     e-value= 2.6e-25 complete sequence
                     hit coverage= 100%, between model( 307 aa) positions 1;
                     307 length is 299aa with 20 gaps, 4 stops, absolute
                     frame= Minus3"
     CDS             complement(472752..471881)
                     /colour="255 0 0"
                     /evidence=predicted
                     /translation="HKXTQARTH-QYRIHXLKX------RVFTGA---KAAIQSLAEL
                     TQMvENHKGNECDDSL-----TLKQS-----VYFIDCFSRLLxsKLKGYCVNHKRVAR
                     LMRQIGIMAKTKVKRKRTTNSEHSFPRYGNRVL-NLSIDHPEQVWVADITYIRLQQEF
                     VYLAVVMDVFTRAIRGWHLSRHIDQQLTLRALNKALERATPE--------IHHSDQGV
                     QYAAAAYMQLLQQHQVQISMAEVGQAWQNGYAERLMRTIKEEEVDLSDYrnFTEAYEH
                     IEQFLEDV--YMHKRIHSSLGYLTPCEYEQQWRQQNNHYCMNKEDS"
                     /note="IS3_IS51_ORF2     e-value= 1.6e-14 complete sequence
                     hit coverage= 100%, between model( 317 aa) positions 1;
                     317 length is 291aa with 31 gaps, 3 stops, absolute
                     frame= Minus3"
     CDS             complement(472737..471908)
                     /colour="255 0 0"
                     /evidence=predicted
                     /translation="ARTHQYRIHXLkXRVFT----GAKAAIQSLAELTQMVENHKGNE
                     CDDSLTLKQSVYFIDCFSRLLXSklKGYCVNHKRVARLMRQIGIMAKTKVKRKRTTNS
                     EHSFPRY---GNRVLNLSIDHPEQVWVADITYIRLQQEFVYLAVVMDVFTRAIRGWHL
                     SRHIDQQLTLRALNKALER----ATPE---IHHSDQGVQYAAAAYMQLLQQHQVQISM
                     AEVGQAWQNGYAERLMRTIKEEEVDLSDYRNFTEAYEHIEQFLEDVYMHKRIHSSLGY
                     LTPCEYEQQWRQQNN"
                     /note="IS3_IS3_ORF2      e-value= 7.9e-44 complete sequence
                     hit coverage= 100%, between model( 288 aa) positions 1;
                     288 length is 277aa with 14 gaps, 3 stops, absolute
                     frame= Minus3"
     CDS             complement(472722..471920)
                     /colour="255 0 0"
                     /evidence=predicted
                     /translation="YRIHXLKXRVFTGAKAAIQSLAELT--QMVEN--HKG--NECDD
                     SLTLKQSVYFID-----CFSRLLXSKL--KGYCVNHKRVARLMRQIGIMAKTKVKRKR
                     TTNSEHSfPRYGNRVLNLS-IDHPEQVWVADITYIRLQQEFVYLAVVMDVFTRAIRGW
                     HLSRHIDQQLTLRALNKALERA-TPE--IHHSDQGVQYAAAAYMQLLQQHQVQISMAE
                     VGQAWQNGYAERLMRTIK-EEEVDL-SDYRNFTEAYEHIEQFLEDVYMHKRIHSSLGY
                     LTPCEYEQQWR"
                     /note="IS3_IS3_ORF1      e-value= 1.8e-35 complete sequence
                     hit coverage= 100%, between model( 286 aa) positions 1;
                     286 length is 268aa with 19 gaps, 3 stops, absolute
                     frame= Minus3"
```

```
CDS             complement(472551..471914)
                /colour="255 0 0"
                /evidence=predicted
                /translation="SKLKGYCVNHKRVARLMRQIGIMAKTKVK-RKRTTNSEHSfpRY
                GNRVLNLS--IDHPEQVWVADITYIRLQ-QEFVYLAVVMDVFTRAIRGWHLSRHIDQQ
                LTLRALNKALERAT---PEIHHS-----DQGVQYAAAAYMQLLQQHQVQISMAEVGQA
                WQNGYAERLMRTIKEEEVD--LSDYRNFTEAYEHIEQFLEdVYMHKRIHSSLGYLTPC
                EYEQQWRQQ"
                /note="IS3_IS150_ORF2    e-value= 3.3e-35 fragment hit
                coverage= 57.58%, between model( 389 aa) positions 166;
                389 length is 213aa with 14 gaps, 0 stops, absolute
                frame= Minus3"
CDS             complement(472548..471917)
                /colour="255 0 0"
                /evidence=predicted
                /translation="KLKGYCVNHKRVARLMRQIGIMAKTKVK----RKRTTNSEHSF-
                PRYGNRVlNLSIDHPEQVWVADITYIRL--QQEFVYLAVVMDVFTRAIRGWHLSRHID
                QQLTLRALNKALER----ATPEIHHSDQGVQYAAAAYMQLLQQHQVQISMAEVGQAWQ
                NGYAERLMRTIKEEEVDLSDYRNFT--EAYEHIEQFLEDVYMHKRIHSSLGYLTPCEY
                EQQWRQ"
                /note="IS3_IS150_ORF1    e-value= 2.3e-40 fragment hit
                coverage= 72.55%, between model( 306 aa) positions 85;
                306 length is 211aa with 13 gaps, 0 stops, absolute
                frame= Minus3"
CDS             complement(472548..471920)
                /colour="255 0 0"
                /evidence=predicted
                /translation="K-LKGYCVNHKRVARLMRQIGIMAKTKVKRKRKRTTNSEHSfPRYG
                NRVLNLS-IDHPEQVWVADITYIRLQQEFVYLAVVMDVFTRAIRGWHLSRHIDQQLTL
                RALNKALERA-TPE--IHHSDQGVQYAAAAYMQLLQQHQVQISMAEVGQAWQNGYAER
                LMRTIK-EEEVDL-SDYRNFTEAYEHIEQFLEDVYMHKRIHSSLGYLTPCEYEQQWR"
                /note="IS3_IS3_ORF1      e-value= 2.3e-45 fragment hit
                coverage= 75.52%, between model( 286 aa) positions 71;
                286 length is 210aa with 7 gaps, 0 stops, absolute frame=
                Minus3"
CDS             complement(472539..471908)
                /colour="255 0 0"
                /evidence=predicted
                /translation="GYCVNHKRVARLMRQIGIMAKTKVKRKRKRTTNSEHSFPRY---GN
                RVLNLSIDHPEQVWVADITYIRLQQEFVYLAVVMDVFTRAIRGWHLSRHIDQQLTLRA
                LNKALER----ATPE---IHHSDQGVQYAAAAYMQLLQQHQVQISMAEVGQAWQNGYA
                ERLMRTIKEEEVDLSDYRNFTEAYEHIEQFLEDVYMHKRIHSSLGYLTPCEYEQQWRQ
                QNN"
                /note="IS3_IS3_ORF2      e-value= 7.7e-58 fragment hit
                coverage= 76.74%, between model( 288 aa) positions 68;
                288 length is 211aa with 10 gaps, 0 stops, absolute
                frame= Minus3"
CDS             complement(472530..471890)
                /colour="255 0 0"
                /evidence=predicted
                /translation="VNHKRVARLMRQIGIMAKTKVKRKRKRTTNSEHSFPRYGNRVL-NL
                SIDHPEQVWVADITYIRLQQEFVYLAVVMDVFTRAIRGWHLSRHIDQQLT-LRALNKA
                LERATPE-------IHHSDQGVQYAAAAYMQLLQQHQVQISMAEVGQAWQNGYAERLM
                RTIKEEEVDL---S-DYRNFTEAYEHIEQFLEDVYMHKRIHSSLGYLTPCEYEQQWRQ
                QNNHYCMNK"
                /note="IS3_IS51_ORF1     e-value= 9.6e-44 fragment hit
                coverage= 73.94%, between model( 307 aa) positions 81;
                307 length is 214aa with 13 gaps, 0 stops, absolute
                frame= Minus3"
CDS             complement(472518..471905)
```

```
                     /colour="255 0 0"
                     /evidence=predicted
                     /translation="RVARLMRQIGIMAKTKVKRKRTTNSEHSFPRYGNRVL-NLSIDH
                     PEQVWVADITYIRLQQEFVYLAVVMDVFTRAIRGWHLSRHIDQQLTLRALNKALERAT
                     PE--------IHHSDQGVQYAAAAYMQLLQQHQVQISMAEVGQAWQNGYAERLMRTIK
                     EEEVDLSDYrnFTEAYEHIEQFLEDV--YMHKRIHSSLGYLTPCEYEQQWRQQNNH"
                     /note="IS3_IS51_ORF2     e-value= 1.8e-35 fragment hit
                     coverage= 67.51%, between model( 317 aa) positions 96;
                     309 length is 205aa with 11 gaps, 0 stops, absolute
                     frame= Minus3"
     CDS             complement(470568..470162)
                     /colour="255 0 0"
                     /evidence=predicted
                     /translation="MSLVRPISTESLRLLHRIYHSSRHHQVRQRAHCLILFAQGWPPY
                     TLASLFSVSPKTVYNWLKAWNNRG---FAGLYNHPGRGRKPMFNPDQQQQIYEWT-QA
                     SPIQ---------LNQVLAQIEQQWSVRVSKATVKRVLKQMDMSWHR"
                     /note="IS630  e-value= 3.4e-15 fragment hit coverage=
                     39.63%, between model( 376 aa) positions 1; 149 length is
                     136aa with 13 gaps, 0 stops, absolute frame= Minus3"
     CDS             complement(470508..470171)
                     /colour="255 0 0"
                     /evidence=predicted
                     /translation="SSRHHQVRQRAHCLILFAQGWPPYTLASLFSVSPKTVYNWLKAW
                     NNRGFAGLYNH---PGRGRKPMfNPDQQQQIYEWTQASPIQLNQVLAQIEQQWSVR--
                     -VSKATVKRVLKQMDMS"
                     /note="IS481  e-value= 3.5e-09 fragment hit coverage=
                     33.62%, between model( 351 aa) positions 1; 118 length is
                     113aa with 6 gaps, 0 stops, absolute frame= Minus3"
     CDS             complement(260016..259754)
                     /colour="255 0 0"
                     /evidence=predicted
                     /translation="LFYFLMQCPLCGHPK-THKHGKTSKGSQRYRCPHCQQTFS-Etf
                     DTLCYRRQiSPETIQTILQAHVEGSSLRGLSRITGVAYNTCVSVVR"
                     /note="IS1_ORF1    e-value= 1.7e-15 fragment hit coverage=
                     36.40%, between model( 239 aa) positions 1; 87 length is
                     88aa with 2 gaps, 0 stops, absolute frame= Minus3"
     CDS             complement(260016..259316)
                     /colour="255 0 0"
                     /evidence=predicted
                     /translation="LFYFLMQCPLCGHPK-THKHGKTSKGSQRYRCPHCQQTFS-Etf
                     DTLCYRRQiSPETIQTILQAHVEGSSLRGLSRITGVAYNTCVSVVRSA---SHKSQMI
                     HNGEVQAVAT---DVINADELWSFVKkskstVSRRSXGXATAGXHXVLRKRVVSCSVA
                     VLADIPTSXLknxLKILRAKPPVIIGK----------RMVGKGTH-----DNYPMRSS
                     MKXVKPXPNAxsgpmascVSRPGVGIDDRINLA-KSGNRV"
                     /note="IS1_ORF1    e-value= 2.6e-07 complete sequence hit
                     coverage= 100%, between model( 239 aa) positions 1; 239
                     length is 234aa with 24 gaps, 7 stops, absolute frame=
                     Minus3"
     CDS             complement(220944..220250)
                     /colour="255 0 0"
                     /evidence=predicted
                     /translation="FKHLHLGMIsDIKRKSLPAIARAVGLENHQSLHHFLSESPWVAS
                     hLSDkRLDIILKILDGRRLILLID------------------ETGDCKKGKSTDYVKR
                     QYIGNVGKKENGIvAVTAYGLFQGMILPLSFEVYKPR---------ErLKEEDEYLSK
                     PQIAAGMIRQLQGMGFCFELVLADSLYGEAQTNFVNVLEELKLPYILAIRSNHAVWLP
                     AEQAVFAEPWQSFERTFSNGTTETRYrQEIIYGqrhhQKRY"
                     /note="IS4   e-value= 4.7e-14 fragment hit coverage=
                     51.23%, between model( 486 aa) positions 1; 249 length is
                     232aa with 27 gaps, 0 stops, absolute frame= Minus3"
     CDS             complement(220944..219818)
```

```
                     /colour="255 0 0"
                     /evidence=predicted
                     /translation="FKHLHLGMIsDIKRKSLPAIARAVGLENHQSLHHFLSESPWVAS
                     hLSDkRLDIILKILDGRRLILLID------------------ETGDCKKGKSTDYVKR
                     QYIGNVGKKENGIvAVTAYGLFQGMILPLSFEVYKPR---------ErLKEEDEYLSK
                     PQIAAGMIRQLQGMGFCFELVLADSLYGEAQTNFVNVLEELKLPYILAIRSNHAVWLP
                     AEQAVFAEPWQSFERTFSNGT-------------------------------TETRYR
                     QEIIYGQRHHQKRyWLLTTDPQTLPENSTS-----------------------YVMAA
                     APE----IKLDEIGDCYGFRTWIEYGLKQSKD-TLGWADFRMTHYEQIEKWWEIVMSA
                     FLMVSLFADVFNDS-------------------------CPVAHQHFAQHPwWDNQNG
                     WKNLLNNVR--LIIQPLIS----WNLLKRWLEVFPSRALKKGF"
                     /note="IS4   e-value= 9.9e-07 complete sequence hit
                     coverage= 100%, between model( 486 aa) positions 1; 486
                     length is 376aa with 117 gaps, 0 stops, absolute frame=
                     Minus3"
     CDS             complement(127077..126635)
                     /colour="255 0 0"
                     /evidence=predicted
                     /translation="DYLIAVKRNQGRLYDHLQTYFeCLKPMAEHTHS-----IQSRGR
                     EEHRCIQVYEPVGI---ALQEWEAIRSVLCVQRWGTRQGKAY----HNTAYYISSAAT
                     SPHH---WQSLVREHWGIENRLHWPKDVVFGEDDYRLEDEQALLNWSVLRTIVINILR
                     LNG"
                     /note="ISAs1  e-value= 2.4e-27 fragment hit coverage=
                     41.65%, between model( 389 aa) positions 198; 359 length
                     is 148aa with 15 gaps, 0 stops, absolute frame= Minus3"

//
```
