# Supplementary material for: TnpPred: A Web Service for the Robust Prediction of Prokaryotic Transposases
Source: Comp Funct Genomics. 2012 Nov 18;2012:678761. doi: 10.1155/2012/678761 (PMC3506888; doi:10.1155/2012/678761)
Supplement: Supplementary file 3 [file 678761.f3.pdf]

Supplementary file 3
Stenotrophomonas maltophilia K279a TnpPred annotation in genbank format (.gbk)

```
LOCUS       NC_010943                    0 bp    DNA     linear   UNK
ACCESSION   unknown
COMMENT     This looks like a good IS fragment prediction
FEATURES             Location/Qualifiers
     source          1..4851126
                     /db_xref="taxon:522373"
                     /mol_type="genomic DNA"
                     /organism="Stenotrophomonas maltophilia K279a"
     CDS             385588..385701
                     /colour="255 0 0"
                     /evidence=predicted
                     /translation="MRSPRLSTRERAVLQWVLRG-CSTGEMARRSGRAASTIS"
                     /note="IS30  e-value= 5.7e-06 fragment hit coverage=
                     11.05%, length is 38aa with 1 gaps, 0 stops, frame=
                     Plus1"
     CDS             478387..479325
                     /colour="255 0 0"
                     /evidence=predicted
                     /translation="FGIGIDVSKATLDVAVHGEQ------FRQFSNDKRGFVRLIRWL
                     KTWP-----IKQVVLEASGGYERAALDVLHAA---GLPMVRINPARARRFAQGT-GRA
                     AKTDRLDATVLAQMAHLLPLTRYVP--PAPW---QQRLAEFAQCRRQLVQMRVSEMQR
                     LRRLVDPDLIA-----------------------------------------------
                     ----------------------------MKQRHIAQLSDDLKQLDKAIAEQLEGQPGW
                     KNIGA------LKGVGPILISTLACELP-ELGSLGSK-AIASLVGLAPMNRESG-TWQ
                     GQRRISG-GRAVVREALYMAALTAIRYEPRLRAFYAGLKAKGK---ASKVALVAVMRK
                     MLVILNARkrdaeVALGCPXQDSCYWGYR"
                     /note="IS110  e-value= 1.6e-50 complete sequence hit
                     coverage= 100%, length is 313aa with 108 gaps, 1 stops,
                     frame= Plus1"
     CDS             478387..479262
                     /colour="255 0 0"
                     /evidence=predicted
                     /translation="FGIGIDVSKATLDVAVHGEQ------FRQFSNDKRGFVRLIRWL
                     KTWP-----IKQVVLEASGGYERAALDVLHAA---GLPMVRINPARARRFAQGT-GRA
                     AKTDRLDATVLAQMAHLLPLTRYVP--PAPW---QQRLAEFAQCRRQLVQMRVSEMQR
                     LRRLVDPDLIA-----------------------------------------------
                     ----------------------------MKQRHIAQLSDDLKQLDKAIAEQLEGQPGW
                     KNIGA------LKGVGPILISTLACELP-ELGSLGSK-AIASLVGLAPMNRESG-TWQ
                     GQRRISG-GRAVVREALYMAALTAIRYEPRLRAFYAGLKAKGK---ASKVALVAVMRK
                     MLVILNAR"
                     /note="IS110  e-value= 1.4e-51 fragment hit coverage=
                     96.15%, length is 292aa with 108 gaps, 0 stops, frame=
                     Plus1"
     CDS             572224..573165
                     /colour="255 0 0"
                     /evidence=predicted
                     /translation="NARLTVHSRALLIRRILHEGLRPEEAAQACGVSVRTAYKWLARF
                     RQFGAPGLENRSSRPHQTPHATPAPVIEQIKEHR-RKRQTYLTISKALG---------
                     VGHSTISRLMRAHGLNRLCRLEPPK-----EVIR--------YEYDQPGGLLHLDIKK
                     -----LGNFQRPGHRTDAkRRGNAAGGGWGYVHVAIDDHSRVAFSSVHPNEQAETACQ
                     ALLGALQYYASLGIT-FKRILTDNGACYRST----AFAKLLKSLGIKHIRTKPYTPRT
                     NGKAERFIQTSLREWAYARE---YASSDQRNSVLTQWLHHYNWHRPHM--GIGGQPPI
                     SRVLLNNVVGLHSXSTIA"
                     /note="IS481  e-value= 5e-95 complete sequence hit
                     coverage= 100%, length is 314aa with 38 gaps, 1 stops,
                     frame= Plus1"
```

```
CDS             572224..573165
                /colour="255 0 0"
                /evidence=predicted
                /translation="NARLTVHSRALLIRRILHEGLRPEEAAQACGVSVRTAYKWLARF
                RQFGAPGLENRSSRPHQTPHATPAPVIEQIKEHR-RKRQTYLTISKALG---------
                VGHSTISRLMRAHGLNRLCRLEPPK-----EVIR--------YEYDQPGGLLHLDIKK
                -----LGNFQRPGHRTDAkRRGNAAGGGWGYVHVAIDDHSRVAFSSVHPNEQAETACQ
                ALLGALQYYASLGIT-FKRILTDNGACYRST----AFAKLLKSLGIKHIRTKPYTPRT
                NGKAERFIQTSLREWAYARE---YASSDQRNSVLTQWLHHYNWHRPHM--GIGGQPPI
                SRVLLNNVVGLHSXSTIA"
                /note="IS481 e-value= 1.3e-95 complete sequence hit
                coverage= 100%, length is 314aa with 38 gaps, 1 stops,
                frame= Plus1"
CDS             572845..573111
                /colour="255 0 0"
                /evidence=predicted
                /translation="RILTDNGACYRSTAFAKLLKSLGIKHIRTKPYTPRTNGKAERFI
                QTSLREWAY--AREYASSDQRNSVLTQWLHHYNWHRPHMGIGGQPPI"
                /note="IS3_IS150_ORF2    e-value= 1.8e-10 fragment hit
                coverage= 23.39%, length is 89aa with 2 gaps, 0 stops,
                frame= Plus1"
CDS             573040..573108
                /colour="255 0 0"
                /evidence=predicted
                /translation="VLTQWLHHYNWHRPHMGIGGQPP"
                /note="IS3_IS407_ORF2    e-value= 4.1e-07 fragment hit
                coverage= 6.69%, length is 23aa with 0 gaps, 0 stops,
                frame= Plus1"
CDS             850483..851532
                /colour="255 0 0"
                /evidence=predicted
                /translation="----SRRCPS-------VGTASAAAPKAD---EKDPFVQAAXGN
                EPRXAAQGS-----RLSACGD---RLPKKTRCLDPG------RAGGARRKAQAIQGLR
                QVHTLSLLLEAAELSRSTFYYQNHVL--AHPDqDEAALCERIRAIYDQSQGRYGYRTV
                TLELA---NQGHRTNHKRVQRLMGEMGLKSRVRVK-RYRSFKGAANVVVGNDLNRQFH
                AERPNQKWVTDVTEFKVQ-GMKLYLSPIMDLYNGEIVAYQIKRQPVFDLVGQMLEEAI
                MKLSPDERPMIHS-----DQGWQYQHENYRHMLEKHSLKQSMSRRGNCLDNAAMESFF
                GTLKSEFFY--LNSFDSIESLEAGLVEYIQYYNQERIKLKLKGLSPVEYreRAQSAAX
                "
                /note="IS3_IS150_ORF2    e-value= 5e-121 complete sequence
                hit coverage= 100%, length is 350aa with 42 gaps, 3
                stops, frame= Plus1"
CDS             850624..851523
                /colour="255 0 0"
                /evidence=predicted
                /translation="LPKKTRCLDPGRAggARRKAQAIQGLRQVHTLSLLLEAAELSRS
                TFYYQNHVLAHPDqDEAALCERIRAIYDQSQGRYGYRTVTLELANQGHRTNHKRVQRL
                MGEMGLKSRVRVK---RYRSFKGAANVVVGNDLNRQFHAERPNQKWVTDVTEFKV--Q
                GMKLYLSPIMDLYNGEIVAYQIKRQPVFDLVGQMLEEAIMK-LSPDERPMIHSDQGWQ
                YQHENYRHMLEKHSLKQSMSRRGNCLDNAAMESFFGTLKSEFFYLNSFDSIE--SLEA
                GLVEYIQYYNQE-RIKLKLKGLSPVEYRERAQS"
                /note="IS3_IS150_ORF1    e-value= 1.2e-86 complete sequence
                hit coverage= 100%, length is 300aa with 9 gaps, 0 stops,
                frame= Plus1"
CDS             850624..851523
                /colour="255 0 0"
                /evidence=predicted
                /translation="LPKKTRCLDPGRAggARRKAQAIQGLRQVHTLSLLLEAAELSRS
                TFYYQNHVLAHPDqDEAALCERIRAIYDQSQGRYGYRTVTLELANQGHRTNHKRVQRL
                MGEMGLKSRVRVK---RYRSFKGAANVVVGNDLNRQFHAERPNQKWVTDVTEFKV--Q
```

```
                     GMKLYLSPIMDLYNGEIVAYQIKRQPVFDLVGQMLEEAIMK-LSPDERPMIHSDQGWQ
                     YQHENYRHMLEKHSLKQSMSRRGNCLDNAAMESFFGTLKSEFFYLNSFDSIE--SLEA
                     GLVEYIQYYNQE-RIKLKLKGLSPVEYRERAQS"
                     /note="IS3_IS150_ORF1    e-value= 3.2e-87 complete sequence
                     hit coverage= 100%, length is 300aa with 9 gaps, 0 stops,
                     frame= Plus1"
     CDS             850672..851532
                     /colour="255 0 0"
                     /evidence=predicted
                     /translation="RRKAQAIQGLRQVHTLSLLLEAAELSRSTFYYQNHVL--AHPDq
                     DEAALCERIRAIYDQSQGRYGYRTVTLELA---NQGHRTNHKRVQRLMGEMGLKSRVR
                     VK-RYRSFKGAANVVVGNDLNRQFHAERPNQKWVTDVTEFKVQ-GMKLYLSPIMDLYN
                     GEIVAYQIKRQPVFDLVGQMLEEAIMKLSPDERPMIHS-----DQGWQYQHENYRHML
                     EKHSLKQSMSRRGNCLDNAAMESFFGTLKSEFFY--LNSFDSIESLEAGLVEYIQYYN
                     QERIKLKLKGLSPVEYreRAQSAAX"
                     /note="IS3_IS150_ORF2    e-value= 1.9e-151 fragment hit
                     coverage= 76.61%, length is 287aa with 14 gaps, 1 stops,
                     frame= Plus1"
     CDS             850681..851553
                     /colour="255 0 0"
                     /evidence=predicted
                     /translation="AQAIQGLRQVHTLSLLLEAAELSRSTFYYQNHVLAHPD------
                     QDEAALCERIRAIYDQSQGRYGYRTVTLELANQGHRTNHKRVQRLMGEMGLKSRVRVK
                     RYRSFKGAANVVVG-NDLNRQFHAERPNQKWVTDVTEFKV-QGmKLYLSPIMDLYNGE
                     IVAYQIKRQPVFDLV-GQMLEEAIMKLSPDE----RPMIHSDQGWQYQHENYRHMLEK
                     HSLKQSMSRRGNCLDNAAMESFFGTLKSEFFYL----NSFDSIESLEAGLVEYIQYYN
                     Q-ERIKLKLKGLSPVEYrERAQSAAXPRPSLGG"
                     /note="IS3_IS51_ORF1     e-value= 8.5e-39 complete sequence
                     hit coverage= 100%, length is 291aa with 18 gaps, 1
                     stops, frame= Plus1"
     CDS             850681..851556
                     /colour="255 0 0"
                     /evidence=predicted
                     /translation="---AQAIQGLR-QVHTLSLLLEA--AELSRS------TFYYQNH
                     VLAHPDQDEAALCERIRAIYDQSQG-----RYGYRTVTLELANQGHRTNHKRVQRLMG
                     EMGLKSRVRVKRYRSFKG-AANVVVGNDLNRQFHAERPNQKWVTDVTEFKVQGMKLYL
                     SPIMDLYNGEIVAYQIKRQPVFDLVGQMLEEAIMKLSPD-----ERPMIHSDQGWQYQ
                     HENYRHMLEKHSLKQSMSRRGNCLDNAAMESFFGTLKSEFFYLNSFDSIESLEAGLVE
                     YIQ-YYNQERIKLKLKGLSPVEYRER-AQSAAXPRPSLGGH"
                     /note="IS3_IS51_ORF2     e-value= 1.2e-17 complete sequence
                     hit coverage= 100%, length is 292aa with 25 gaps, 1
                     stops, frame= Plus1"
     CDS             850684..851520
                     /colour="255 0 0"
                     /evidence=predicted
                     /translation="QAIQGLRQVHTLSLLLEAAELSRSTFYYQNHVLAHPD---QDEA
                     ALCERIRAIYDQSQGRYGYRTVTLELA-NQGHRTNHKRVQRLMGEMGLKSRVRV-KRY
                     RSFKgAANVVVGNDLNRQFHAERPNQKWVTDVTEFKVQGMKLYLSPIMDLYNGEIVAY
                     QIKRQPVFDLVGQMLEEAIMKLSPDERPMIHSDQGWQYQHENYRHMLEKHSLKQSMSR
                     RGNCLDNAAMESFFGTLK--SEFFYLNSFDSIESLEAGLVEYIQ-YYNQERIKLKLKG
                     LSPVEYRERAQ"
                     /note="IS3_IS3_ORF1      e-value= 1.8e-74 complete sequence
                     hit coverage= 100%, length is 279aa with 8 gaps, 0 stops,
                     frame= Plus1"
     CDS             850684..851520
                     /colour="255 0 0"
                     /evidence=predicted
                     /translation="QAIQGLRQVHTLSLLLEAAELSRSTFYYQNHVLAHPD---QDEA
                     ALCERIRAIYDQSQGRYGYRTVTLELA-NQGHRTNHKRVQRLMGEMGLKSRVRV-KRY
                     RSFKgAANVVVGNDLNRQFHAERPNQKWVTDVTEFKVQGMKLYLSPIMDLYNGEIVAY
```

```
                     QIKRQPVFDLVGQMLEEAIMKLSPDERPMIHSDQGWQYQHENYRHMLEKHSLKQSMSR
                     RGNCLDNAAMESFFGTLK--SEFFYLNSFDSIESLEAGLVEYIQ-YYNQERIKLKLKG
                     LSPVEYRERAQ"
                     /note="IS3_IS3_ORF1      e-value= 4.6e-75 complete sequence
                     hit coverage= 100%, length is 279aa with 8 gaps, 0 stops,
                     frame= Plus1"
     CDS             850696..851532
                     /colour="255 0 0"
                     /evidence=predicted
                     /translation="GLRQVHTLSLLLEAAELSRSTFYYQNHV-LAHPDQDEAALCERI
                     RAIYDQSQGRYGYRTVTLELANQGHRTNHKRVQRLMGEMGLKSRVRVKRYRSFKGAAN
                     VVVG-NDLNRQFHAER--PNQKWVTDVTEFKVQGMKLYLSPIMDLYNGEIVAYQIKRQ
                     PVFDLVGQMLEEAIMK----LSPDERPMIHSDQGWQYQHENYRHMLEKHSLKQSMSRR
                     GNCLDNAAMESFFGTLKSEFFYLNSFDSIESLEAGLVEYIQYY-NQERIKLKLKGLSP
                     VEYRERAQSAAX"
                     /note="IS3_IS3_ORF2      e-value= 1.2e-63 complete sequence
                     hit coverage= 100%, length is 279aa with 9 gaps, 1 stops,
                     frame= Plus1"
     CDS             850696..851532
                     /colour="255 0 0"
                     /evidence=predicted
                     /translation="GLRQVHTLSLLLEAAELSRSTFYYQNHV-LAHPDQDEAALCERI
                     RAIYDQSQGRYGYRTVTLELANQGHRTNHKRVQRLMGEMGLKSRVRVKRYRSFKGAAN
                     VVVG-NDLNRQFHAER--PNQKWVTDVTEFKVQGMKLYLSPIMDLYNGEIVAYQIKRQ
                     PVFDLVGQMLEEAIMK----LSPDERPMIHSDQGWQYQHENYRHMLEKHSLKQSMSRR
                     GNCLDNAAMESFFGTLKSEFFYLNSFDSIESLEAGLVEYIQYY-NQERIKLKLKGLSP
                     VEYRERAQSAAX"
                     /note="IS3_IS3_ORF2      e-value= 3.3e-64 complete sequence
                     hit coverage= 100%, length is 279aa with 9 gaps, 1 stops,
                     frame= Plus1"
     CDS             850804..851505
                     /colour="255 0 0"
                     /evidence=predicted
                     /translation="AALCERIRAIYDQSQGRYGYRTVTLELANQGHRTNHKRVQRLMG
                     EMGLKSRVRVKRYRSFKGAANVVVG-NDLNRQFHAERPNQKWVTDVTEFKV-QGmKLY
                     LSPIMDLYNGEIVAYQIKRQPVFDLV-GQMLEEAIMKLSPDE----RPMIHSDQGWQY
                     QHENYRHMLEKHSLKQSMSRRGNCLDNAAMESFFGTLKSEFFYL----NSFDSIESLE
                     AGLVEYIQYYNQ-ERIKLKLKGLSPVEY"
                     /note="IS3_IS51_ORF1     e-value= 5e-45 fragment hit
                     coverage= 79.80%, length is 234aa with 12 gaps, 0 stops,
                     frame= Plus1"
     CDS             850855..851382
                     /colour="255 0 0"
                     /evidence=predicted
                     /translation="YGYRTVTLELANQGHRTNHKRVQRLMGEMGLKSRVRVKRYRSFK
                     G-AANVVVGNDLNRQFHAERPNQKWVTDVTEFKVQGMKLYLSPIMDLYNGEIVAYQIK
                     RQPVFDLVGQMLEEAIMKLSPD-----ERPMIHSDQGWQYQHENYRHMLEKHSLKQSM
                     SRRGNCLDNAAMESFFGTLKSE"
                     /note="IS3_IS51_ORF2     e-value= 7.8e-32 fragment hit
                     coverage= 57.41%, length is 176aa with 6 gaps, 0 stops,
                     frame= Plus1"
     CDS             851359..851466
                     /colour="255 0 0"
                     /evidence=predicted
                     /translation="FFGTLKSEFFYLNS---FDSIESLEAGLVEYIQYYNQER"
                     /note="IS481  e-value= 3.8e-07 fragment hit coverage=
                     11.11%, length is 36aa with 3 gaps, 0 stops, frame=
                     Plus1"
     CDS             1150204..1150311
                     /colour="255 0 0"
```

```
                     /evidence=predicted
                     /translation="LAYVVMPDHIHWLVEIR-AFSLDYIMQRFKSSSALRI"
                     /note="IS200 e-value= 6.8e-07 fragment hit coverage=
                     24.03%, length is 36aa with 1 gaps, 0 stops, frame=
                     Plus1"
     CDS             1150204..1150311
                     /colour="255 0 0"
                     /evidence=predicted
                     /translation="LAYVVMPDHIHWLVEIR-AFSLDYIMQRFKSSSALRI"
                     /note="IS200_IS605 e-value= 5.7e-06 fragment hit
                     coverage= 24.18%, length is 36aa with 1 gaps, 0 stops,
                     frame= Plus1"
     CDS             1775491..1776408
                     /colour="255 0 0"
                     /evidence=predicted
                     /translation="NGIGIDVSKRRLDIGTTDGQT------LQVSNSPSGYAELSTWL
                     STRP-----AIQIVLEATGGYEQTVLDFLHKA---GHPVIRANALRARRLA-QGLGKV
                     AKTDRLDALVLAQMA--ALVELPSYQPLEPW----QRKLREFVRARRQTMQALTVARQ
                     QQEMVTDRELRRQMQG------------------------------------------
                     ----------------------------NIIRLQTLVERLGKQISEQVAQ-QPQLAVL
                     KS---------MKGVGPALQAVLASYLP-ELGQISGK-AIASLVGVAPISHDSG-AMR
                     GRRSIHG-GRAEIRQVLYMAAMSAMRHEPRLRDFYRSLRARGK---EGKVAIVAVMRK
                     MLVILNARVRDAE--NGLVPAXGQ"
                     /note="IS110 e-value= 3.4e-46 complete sequence hit
                     coverage= 100%, length is 306aa with 110 gaps, 1 stops,
                     frame= Plus1"
     CDS             1775491..1776375
                     /colour="255 0 0"
                     /evidence=predicted
                     /translation="NGIGIDVSKRRLDIGTTDGQT------LQVSNSPSGYAELSTWL
                     STRP-----AIQIVLEATGGYEQTVLDFLHKA---GHPVIRANALRARRLA-QGLGKV
                     AKTDRLDALVLAQMA--ALVELPSYQPLEPW----QRKLREFVRARRQTMQALTVARQ
                     QQEMVTDRELRRQMQG------------------------------------------
                     ----------------------------NIIRLQTLVERLGKQISEQVAQ-QPQLAVL
                     KS---------MKGVGPALQAVLASYLP-ELGQISGK-AIASLVGVAPISHDSG-AMR
                     GRRSIHG-GRAEIRQVLYMAAMSAMRHEPRLRDFYRSLRARGK---EGKVAIVAVMRK
                     MLVILNARVRD"
                     /note="IS110 e-value= 1e-48 fragment hit coverage=
                     96.88%, length is 295aa with 108 gaps, 0 stops, frame=
                     Plus1"
     CDS             1896472..1897581
                     /colour="255 0 0"
                     /evidence=predicted
                     /translation="VQDRGGPT------DRXVRPSGSGGCRATgRVDPXSLRLEAATR
                     QRRCWPACRAGPERGS-APPQGXATQGHXRARHPKKSRRVL---CKGVRAKYAFMKQH
                     ADEFGLAAMCRMLGVHRSGYYAWLKEPASARDKDDQRLLGLIKHSWLESGSVYGHRKV
                     TTDLR---ELGETCSRHRVARLMKSEGLRAMVGYGRRPRPLSGPVGSVAKNVLARGFK
                     VSEPNRAWVTDITYIRTY-DGFLYLAVVLDLFSRQVVGWATRPTQHTDLVLQALLAAV
                     WRRKPSPGLLLHS-----DQGTQFTSEDWQSFLREHDIVCSMSRRGNCHDNAAMESFF
                     QLLKRERIK--RRIYSNHDEARADVFQYIEmFYNPKRRHSSNDGLSPVEFEKQYALN"
                     /note="IS3_IS150_ORF2    e-value= 1.2e-45 complete sequence
                     hit coverage= 100%, length is 370aa with 21 gaps, 4
                     stops, frame= Plus1"
     CDS             1896673..1897578
                     /colour="255 0 0"
                     /evidence=predicted
                     /translation="ARHPKKSRRVLCKgVRAKYAFMKQHADEFGLAAMCRMLGVHRSg
                     YYAWLKePASARDKDDQRLLGLIKHSWLESGSVYGHRKVTTDLRELGETCSRHRVARL
                     MKSEGLRAMVGYGR--RPRPLSGPVGSVAKNVLARGFKVSEPNRAWVTDITYIRT--Y
                     DGFLYLAVVLDLFSRQVVGWATRPTQHTDLVLQALLAAVWR-RKPSPGLLLHSDQGTQ
```

```
                     FTSEDWQSFLREHDIVCSMSRRGNCHDNAAMESFFQLLKRERIKRRIYSNHD--EARA
                     DVFQYIEMFYNPKRRHSSNDGLSPVEFEKQYAL"
                     /note="IS3_IS150_ORF1    e-value= 1.4e-57 complete sequence
                     hit coverage= 100%, length is 302aa with 7 gaps, 0 stops,
                     frame= Plus1"
     CDS             1896697..1897590
                     /colour="255 0 0"
                     /evidence=predicted
                     /translation="RVLCKGVRAKYAFMkqhADEFGLAAMCRMLGVHRSGYYAWLK--
                     ----EPASARDKDDQRLLGLIKHSWLESGSvYGHRKVTTDLRELGETCSR-----HRV
                     ARLMkseGLRAMVgYGRRPRPLSGP---VGSVAknvlargfkVSEPNRAWVTDITYIR
                     TYDG-FLYLAVVLDLFSRQVVGWATRPTQ--HTDLVLQALLAAVWRR----KPSPGLL
                     LH-SDQGTQFTSEDWQSFLREHDIVCSMSRRGNCHDNAAMESFFQLLKRERIkrRIYS
                     NHDEARA--DVFQYIEMfYNPKRRHSSNDGLSPVEFEKQYALNGXR"
                     /note="IS3_IS2_ORF2      e-value= 1.4e-07 complete sequence
                     hit coverage= 100%, length is 298aa with 24 gaps, 1
                     stops, frame= Plus1"
     CDS             1896700..1897578
                     /colour="255 0 0"
                     /evidence=predicted
                     /translation="VLCKgVRAKYAFMKQHADEFGLAAMCRMLGVHRSgYYAWLKePA
                     SARDKDDQRLLGLIKHSWLESGSVYGHRKVTTDLRELGETCSRHRVARLMKSEGLRAM
                     VGYGR--RPRPLSGPVGSVAKNVLARGFKVSEPNRAWVTDITYIRT--YDGFLYLAVV
                     LDLFSRQVVGWATRPTQHTDLVLQALLAAVWR-RKPSPGLLLHSDQGTQFTSEDWQSF
                     LREHDIVCSMSRRGNCHDNAAMESFFQLLKRERIKRRIYSNHD--EARADVFQYIEMF
                     YNPKRRHSSNDGLSPVEFEKQYAL"
                     /note="IS3_IS150_ORF1    e-value= 5.1e-57 fragment hit
                     coverage= 97.06%, length is 293aa with 7 gaps, 0 stops,
                     frame= Plus1"
     CDS             1896709..1897617
                     /colour="255 0 0"
                     /evidence=predicted
                     /translation="KGVRAKYAFMKQHAD--EFGLAAMCRMLGVH---RSGYYAWLKE
                     PASARDKDDQRLLGLIKHSWLESGS-----VYGHRKVTTDLRELGETCSRHRVARLMK
                     SEGLRAMVGYGRRPRPLSGPVGSVAKNVLARGFKVSEPNRAWVTDITYIRTYDGFLYL
                     AVVLDLFSRQVVGWATRPTQHTDLVLQALLAAVWRRKPS-----PGLLLHSDQGTQFT
                     SEDWQSFLREHDIVCSMSRRGNCHDNAAMESFFQLLKRERIKRRIYSNHDEARADVFQ
                     YIEMFYNPKRRHSSNDGLSPVEFEKQYALNGxRLSRKPWAIQ"
                     /note="IS3_IS51_ORF2     e-value= 1.4e-134 complete
sequence
                     hit coverage= 100%, length is 303aa with 15 gaps, 0
                     stops, frame= Plus1"
     CDS             1896727..1897572
                     /colour="255 0 0"
                     /evidence=predicted
                     /translation="YAFMKQHADEFGLAAMCRMLGVHRSGYYAWL-----KEPASARD
                     KDDQRLLGLIKHSWLESGSVYGHRKVTTDLRELGETCSRHRVARLMKSEGLRAMVGYG
                     RRPRPLSGPVGSVAKNVLARGFKVSEPNRAWVTDITYIRTYDGFLYLAVVLDLFSRQV
                     VGWATRPTQHTDLV-LQALLAAVWRRKPSP----GLLLHSDQGTQFTSEDWQSFLREH
                     DIVCSMSRRGNCHDNAAMESFFQLLKRERIKR----RIYSNHDEARADVFQYIEMFYN
                     PKRRHSSNDGLSPVEFEKQY"
                     /note="IS3_IS51_ORF1     e-value= 3.9e-110 fragment hit
                     coverage= 96.42%, length is 282aa with 14 gaps, 0 stops,
                     frame= Plus1"
     CDS             1896727..1897605
                     /colour="255 0 0"
                     /evidence=predicted
                     /translation="YAFMKQHADEFGLAAMCRMLGVHRSGYYAWL-----KEPASARD
                     KDDQRLLGLIKHSWLESGSVYGHRKVTTDLRELGETCSRHRVARLMKSEGLRAMVGYG
                     RRPRPLSGPVGSVAKNVLARGFKVSEPNRAWVTDITYIRTYDGFLYLAVVLDLFSRQV
```

```
                   VGWATRPTQHTDLV-LQALLAAVWRRKPSP----GLLLHSDQGTQFTSEDWQSFLREH
                   DIVCSMSRRGNCHDNAAMESFFQLLKRERIKR----RIYSNHDEARADVFQYIEMFYN
                   PKRRHSSNDGLSPVEFEKQYALNGXRLSRKP"
                   /note="IS3_IS51_ORF1     e-value= 3.7e-108 complete
sequence
                   hit coverage= 100%, length is 293aa with 14 gaps, 1
                   stops, frame= Plus1"
     CDS           1896730..1897575
                   /colour="255 0 0"
                   /evidence=predicted
                   /translation="AFMKQHADEFGLAAMCRMLGVHRSGYYAWLKE--PASARDKDDQ
                   RLLGLIKHSWLESGSVYGHRKVTTDLR-ELGETCSRHRVARLMKSEGLRAmVGYG-RR
                   PRPLSgPVGSVAKNVLARGFKVSEPNRAWVTDITYIRTYDGFLYLAVVLDLFSRQVVG
                   WATRPTQHTDLVLQALLAAVWRRKPSPGLLLHSDQGTQFTSEDWQSFLREHDIVCSMS
                   RRGNCHDNAAMESFFQLLK--RERIKRRIYSNHDEARADVFQYIEMFYNPKRRHSSND
                   GLSPVEFEKQYA"
                   /note="IS3_IS3_ORF1      e-value= 1.9e-94 complete sequence
                   hit coverage= 100%, length is 282aa with 6 gaps, 0 stops,
                   frame= Plus1"
     CDS           1896730..1897575
                   /colour="255 0 0"
                   /evidence=predicted
                   /translation="AFMKQHADEFGLAAMCRMLGVHRSGYYAWLKE--PASARDKDDQ
                   RLLGLIKHSWLESGSVYGHRKVTTDLR-ELGETCSRHRVARLMKSEGLRAmVGYG-RR
                   PRPLSgPVGSVAKNVLARGFKVSEPNRAWVTDITYIRTYDGFLYLAVVLDLFSRQVVG
                   WATRPTQHTDLVLQALLAAVWRRKPSPGLLLHSDQGTQFTSEDWQSFLREHDIVCSMS
                   RRGNCHDNAAMESFFQLLK--RERIKRRIYSNHDEARADVFQYIEMFYNPKRRHSSND
                   GLSPVEFEKQYA"
                   /note="IS3_IS3_ORF1      e-value= 4.9e-95 complete sequence
                   hit coverage= 100%, length is 282aa with 6 gaps, 0 stops,
                   frame= Plus1"
     CDS           1896742..1897587
                   /colour="255 0 0"
                   /evidence=predicted
                   /translation="QHADEFGLAAMCRMLGVHRSGYYAWLKEPASARDKDDQRLLGLI
                   KHSWLESGSVYGHRKVTTDLRELGETCSRHRVARLMKSEGLRAMvGYGRRPRPLSGPV
                   GSVAK-NVLARGFKVSE--PNRAWVTDITYIRTYDGFLYLAVVLDLFSRQVVGWATRP
                   TQHTDLVLQALLAAVWR----RKPSPGLLLHSDQGTQFTSEDWQSFLREHDIVCSMSR
                   RGNCHDNAAMESFFQLLKRERIKRRIYSNHDEARADVFQYIEMFYNPKRRHSSNDGLS
                   PVEFEKQYALNGX"
                   /note="IS3_IS3_ORF2      e-value= 7.1e-102 complete
sequence
                   hit coverage= 100%, length is 282aa with 7 gaps, 1 stops,
                   frame= Plus1"
     CDS           1896742..1897587
                   /colour="255 0 0"
                   /evidence=predicted
                   /translation="QHADEFGLAAMCRMLGVHRSGYYAWLKEPASARDKDDQRLLGLI
                   KHSWLESGSVYGHRKVTTDLRELGETCSRHRVARLMKSEGLRAMvGYGRRPRPLSGPV
                   GSVAK-NVLARGFKVSE--PNRAWVTDITYIRTYDGFLYLAVVLDLFSRQVVGWATRP
                   TQHTDLVLQALLAAVWR----RKPSPGLLLHSDQGTQFTSEDWQSFLREHDIVCSMSR
                   RGNCHDNAAMESFFQLLKRERIKRRIYSNHDEARADVFQYIEMFYNPKRRHSSNDGLS
                   PVEFEKQYALNGX"
                   /note="IS3_IS3_ORF2      e-value= 2e-102 complete sequence
hit
                   coverage= 100%, length is 282aa with 7 gaps, 1 stops,
                   frame= Plus1"
     CDS           1896754..1897581
                   /colour="255 0 0"
                   /evidence=predicted
```

```
                     /translation="EFGLAAMCRMLGVHRSGYYAWLKEPASARDKDDQRLLGLIKHSW
                     LESGSVYGHRKVTTDLR---ELGETCSRHRVARLMKSEGLRAMVGYGRRPRPLSGPVG
                     SVAKNVLARGFKVSEPNRAWVTDITYIRTY-DGFLYLAVVLDLFSRQVVGWATRPTQH
                     TDLVLQALLAAVWRRKPSPGLLLHS-----DQGTQFTSEDWQSFLREHDIVCSMSRRG
                     NCHDNAAMESFFQLLKRERIK--RRIYSNHDEARADVFQYIEmFYNPKRRHSSNDGLS
                     PVEFEKQYALN"
                     /note="IS3_IS150_ORF2    e-value= 1.9e-74 fragment hit
                     coverage= 73.52%, length is 276aa with 11 gaps, 0 stops,
                     frame= Plus1"
     CDS             1896757..1897575
                     /colour="255 0 0"
                     /evidence=predicted
                     /translation="FGLAAMCRMLGVH---RSGYYAWLKEPASARDKDDQRLLGLIKH
                     SWLESGS-----VYGHRKVTTDLRELGETCSRHRVARLMKSEGLRAMVGYGRRPRPLS
                     GPVGSVAKNVLARGFKVSEPNRAWVTDITYIRTYDGFLYLAVVLDLFSRQVVGWATRP
                     TQHTDLVLQALLAAVWRRKPS-----PGLLLHSDQGTQFTSEDWQSFLREHDIVCSMS
                     RRGNCHDNAAMESFFQLLKRERIKRRIYSNHDEARADVFQYIEMFYNPKRRHSSNDGL
                     SPVEFEKQYA"
                     /note="IS3_IS51_ORF2     e-value= 7.1e-152 fragment hit
                     coverage= 90.22%, length is 273aa with 13 gaps, 0 stops,
                     frame= Plus1"
     CDS             1925629..1925757
                     /colour="255 0 0"
                     /evidence=predicted
                     /translation="VATAPRQLWCWDMTFLPRdVAGrWFFLYLIMDVYSRKIVGFEV"
                     /note="IS3_IS51_ORF1     e-value= 3.8e-06 fragment hit
                     coverage= 13.36%, length is 43aa with 0 gaps, 0 stops,
                     frame= Plus1"
     CDS             1925629..1925757
                     /colour="255 0 0"
                     /evidence=predicted
                     /translation="VATAPRQLWCWDMTFLpRDVAGrWFFLYLIMDVYSRKIVGFEV"
                     /note="IS3_IS51_ORF2     e-value= 2.3e-08 fragment hit
                     coverage= 12.93%, length is 43aa with 0 gaps, 0 stops,
                     frame= Plus1"
     CDS             1925632..1926123
                     /colour="255 0 0"
                     /evidence=predicted
                     /translation="ATAPRQLWCWDMTFLprDVA-GRWFFLYLIMDVYSRKIVGFEVH
                     EdddwDHAARLVQrTALAEGIHAMPRDERPVLHG-----DNGATLKATTVLAMLWWLG
                     VKPSYSRPRVSDDNAFVESLFRTAKYRpEFP--EKGFTDLCAAREWARQFVHWYNHDH
                     RHSGIRYVSPAQ"
                     /note="IS3_IS150_ORF2    e-value= 2.6e-08 fragment hit
                     coverage= 42.16%, length is 164aa with 8 gaps, 0 stops,
                     frame= Plus1"
     CDS             2293057..2293974
                     /colour="255 0 0"
                     /evidence=predicted
                     /translation="NGIGIDVSKRRLDIGTTDGQT------LQVSNSPSGYAELSTWL
                     STRP-----AIQIVLEATGGYEQTVLDFLHKA---GHPVIRANALRARRLA-QGLGKV
                     AKTDRLDALVLAQMA--ALVELPSYQPLEPW----QRKLREFVRARRQTMQALTVARQ
                     QQEMVTDRELRRQMQG------------------------------------------
                     ----------------------------NIIRLQTLVERLGKQISEQVAQ-QPQLAVL
                     KS---------MKGVGPALQAVLASYLP-ELGQISGK-AIASLVGVAPISHDSG-AMR
                     GRRSIHG-GRAEIRQVLYMAAMSAMRHEPRLRDFYRSLRARGK---EGKVAIVAVMRK
                     MLVILNARVRDAE--NGLVPAXGQ"
                     /note="IS110 e-value= 3.4e-46 complete sequence hit
                     coverage= 100%, length is 306aa with 110 gaps, 1 stops,
                     frame= Plus1"
     CDS             2293057..2293941
```

```
                     /colour="255 0 0"
                     /evidence=predicted
                     /translation="NGIGIDVSKRRLDIGTTDGQT------LQVSNSPSGYAELSTWL
                     STRP-----AIQIVLEATGGYEQTVLDFLHKA---GHPVIRANALRARRLA-QGLGKV
                     AKTDRLDALVLAQMA--ALVELPSYQPLEPW----QRKLREFVRARRQTMQALTVARQ
                     QQEMVTDRELRRQMQG------------------------------------------
                     ---------------------------NIIRLQTLVERLGKQISEQVAQ-QPQLAVL
                     KS---------MKGVGPALQAVLASYLP-ELGQISGK-AIASLVGVAPISHDSG-AMR
                     GRRSIHG-GRAEIRQVLYMAAMSAMRHEPRLRDFYRSLRARGK---EGKVAIVAVMRK
                     MLVILNARVRD"
                     /note="IS110 e-value= 1e-48 fragment hit coverage=
                     96.88%, length is 295aa with 108 gaps, 0 stops, frame=
                     Plus1"
     CDS             2448280..2449305
                     /colour="255 0 0"
                     /evidence=predicted
                     /translation="LPPAWLQRGLVLPVAQQVRRHERARRQAAQGSGGGEHTPEEAAG
                     RTGLRERRHQGCVAKKVVTAPARRLLVRSMVEKGLSERRALTVVRMSASALRYEPRPD
                     N-NVELREQIAALAHRHRRYGVGMIHLKLRQKGLVVNYKRVERLYQEAGLQVRRRK-R
                     KKVPVSERQPLLRPSAANEVWSMDFVFERTAEGRVVKCLTIVDDATHEAVAIEVERAI
                     SGQGVSRVLDRLAMQRGLPRVIRTDNGKEFCGKAMVAWAHEKEVALRLIEPGKPNQNA
                     YIESFNGRLRDECLNEHWFPTLLHARTSIESWRRDYNEERPKRALGGLTPAQYAAQLA
                     AKNDNISTGL"
                     /note="IS3_IS407_ORF2    e-value= 2.1e-202 complete
sequence
                     hit coverage= 100%, length is 342aa with 2 gaps, 0 stops,
                     frame= Plus1"
     CDS             2448280..2449305
                     /colour="255 0 0"
                     /evidence=predicted
                     /translation="LPPAWLQRGLVLPVAQQVRRHERARRQAAQGSGGGEHTPEEAAG
                     RTGLRERRHQGCVAKKVVTAPARRLLVRSMVEKGLSERRALTVVRMSASALRYEPRPD
                     N-NVELREQIAALAHRHRRYGVGMIHLKLRQKGLVVNYKRVERLYQEAGLQVRRRK-R
                     KKVPVSERQPLLRPSAANEVWSMDFVFERTAEGRVVKCLTIVDDATHEAVAIEVERAI
                     SGQGVSRVLDRLAMQRGLPRVIRTDNGKEFCGKAMVAWAHEKEVALRLIEPGKPNQNA
                     YIESFNGRLRDECLNEHWFPTLLHARTSIESWRRDYNEERPKRALGGLTPAQYAAQLA
                     AKNDNISTGL"
                     /note="IS3_IS407_ORF2    e-value= 5.4e-203 complete
sequence
                     hit coverage= 100%, length is 342aa with 2 gaps, 0 stops,
                     frame= Plus1"
     CDS             2448421..2449278
                     /colour="255 0 0"
                     /evidence=predicted
                     /translation="LRERRHQGCVAKKvVTAPARRLLVRSMVEKGLSERRALTVVRMS
                     ASALRYEP-RPDNNVELREQIA-ALAHRHRRYGVGMIHLKLRQKGLVVNYKRVERLYQ
                     EAGLQVRRRKRK--------KVPVSE----RQPLLRPSAANEVWSMDFVFERT-AEGR
                     VVKCLTIVDDATHEAVAIEVERAISGQGVSRVLDRLAMQ-RGLPR--VIRTDNGKEFC
                     GKAMVAWAHEKEVALRLIEPGKPNQNAYIESFNGRLRDECLNEHWFPTLL--HARTSI
                     ESWRRDYNEE-RPKRALGGLTPAQYAAQLAA"
                     /note="IS3_IS150_ORF1    e-value= 6.2e-15 complete sequence
                     hit coverage= 100%, length is 286aa with 21 gaps, 0
                     stops, frame= Plus1"
     CDS             2448460..2448705
                     /colour="255 0 0"
                     /evidence=predicted
                     /translation="VVTAPARRLLVRSMVE-KGLSERRALTVVRMSASALR-YEPR-P
                     DNNVELREQIAALAHRHRRYGVGMIHLKLR-QKGLVVNYKRV"
                     /note="IS3_IS407_ORF1    e-value= 1.1e-17 complete sequence
                     hit coverage= 100%, length is 82aa with 4 gaps, 0 stops,
```

```
                     frame= Plus1"
     CDS             2448460..2448705
                     /colour="255 0 0"
                     /evidence=predicted
                     /translation="VVTAPARRLLVRSMVE-KGLSERRALTVVRMSASALR-YEPR-P
                     DNNVELREQIAALAHRHRRYGVGMIHLKLR-QKGLVVNYKRV"
                     /note="IS3_IS407_ORF1    e-value= 8.5e-17 complete sequence
                     hit coverage= 100%, length is 82aa with 4 gaps, 0 stops,
                     frame= Plus1"
     CDS             2448481..2449275
                     /colour="255 0 0"
                     /evidence=predicted
                     /translation="RLLVRSMVEKGLSERRALTVVRMSA--SALRY----EPRPDNNV
                     ELREQIAALAHRHR-RYGVGMIHLKLR-QKGLVVNYKRVERLYQEAGLQVRRRKRKKv
                     PVSERQPLlR-----------PSAANEVWSMDFVFERTAEGRVVKClTIVDDATHEAV
                     AIEVERAISGQGVSRVLDRLAMQRGLPR--VIRTDNGKEFCGKAMVAWAHEKEVALRL
                     IEPGKPNQNAYIESFNGRLRDECLNEH--WFPTLLHARTSIESWRR-DYNEERPKRAL
                     GGLTPAQYAAQLA"
                     /note="IS3_IS3_ORF1      e-value= 8.7e-09 complete sequence
                     hit coverage= 100%, length is 265aa with 24 gaps, 0
                     stops, frame= Plus1"
     CDS             2448502..2449278
                     /colour="255 0 0"
                     /evidence=predicted
                     /translation="VEKGLSERRALTVVRMSASALRYEP-RPDNNVELREQIA-ALAH
                     RHRRYGVGMIHLKLRQKGLVVNYKRVERLYQEAGLQVRRRKRK--------KVPVSE-
                     ---RQPLLRPSAANEVWSMDFVFERT-AEGRVVKCLTIVDDATHEAVAIEVERAISGQ
                     GVSRVLDRLAMQ-RGLPR--VIRTDNGKEFCGKAMVAWAHEKEVALRLIEPGKPNQNA
                     YIESFNGRLRDECLNEHWFPTLL--HARTSIESWRRDYNEE-RPKRALGGLTPAQYAA
                     QLAA"
                     /note="IS3_IS150_ORF1    e-value= 4.2e-17 fragment hit
                     coverage= 91.50%, length is 259aa with 21 gaps, 0 stops,
                     frame= Plus1"
     CDS             2449078..2449281
                     /colour="255 0 0"
                     /evidence=predicted
                     /translation="PGKPNQNAYIESFNGRLRDECLN--EHWFPTLLHARTSIESWRR
                     DYNEERPKRALGGLTPAQYAAQLAAK"
                     /note="IS3_IS150_ORF2    e-value= 2.9e-08 fragment hit
                     coverage= 17.99%, length is 68aa with 2 gaps, 0 stops,
                     frame= Plus1"
     CDS             2449081..2449260
                     /colour="255 0 0"
                     /evidence=predicted
                     /translation="GKPNQNAYIESFNGRLRDECLNEHWFPTLLHARTSIESWRRDY-
                     NEERPKRALGGLTPAQY"
                     /note="IS3_IS3_ORF2      e-value= 6.9e-07 fragment hit
                     coverage= 21.18%, length is 60aa with 1 gaps, 0 stops,
                     frame= Plus1"
     CDS             2449096..2449275
                     /colour="255 0 0"
                     /evidence=predicted
                     /translation="NAYIESFNGRLRDECLNEH--WFPTLLHARTSIESWRR-DYNEE
                     RPKRALGGLTPAQYAAQLA"
                     /note="IS3_IS3_ORF1      e-value= 2.9e-08 fragment hit
                     coverage= 22.03%, length is 60aa with 3 gaps, 0 stops,
                     frame= Plus1"
     CDS             2494939..2495808
                     /colour="255 0 0"
                     /evidence=predicted
```

```
                     /translation="Y--WGSGKSASADGTKWSVYEDNLLSEYHIRYGGYG-GIgYYHV
                     SDKY-VALFSHFIPCGVHEGIYILDGLLAN--TSDIQPEIVHGDTQAQSYPVFGLAHM
                     LGIQLMPRIRNIKDLTFFRPEPGRAY-KNIQALFGDSIDWRLIATHLHDMLRVVISIR
                     LGKITASSILRRLGTYSRKNKLYFAFRELGKAVRTLFLLRYIDDNEIRKTIHAATNKS
                     EEYNGFVKWVFFGSQGIIAENVQHEQRKIIKYSQLVANMIILHNVEGMSRTLAEMRKE
                     GIELTPEILAGLSPYRTSHIN"
                     /note="Tn3   e-value= 1.5e-20 fragment hit coverage=
                     29.48%, length is 290aa with 7 gaps, 0 stops, frame=
                     Plus1"
     CDS             3623575..3624492
                     /colour="255 0 0"
                     /evidence=predicted
                     /translation="NGIGIDVSKRRLDIGTTDGQT------LQVSNSPSGYAELSTWL
                     STRP-----AIQIVLEATGGYEQTVLDFLHKA---GHPVIRANALRARRLA-QGLGKV
                     AKTDRLDALVLAQMA--ALVELPSYQPLEPW----QRKLREFVRARRQTMQALTVARQ
                     QQEMVTDRELRRQMQG------------------------------------------
                     ----------------------------NIIRLQTLVERLGKQISEQVAQ-QPQLAVL
                     KS---------MKGVGPALQAVLASYLP-ELGQISGK-AIASLVGVAPISHDSG-AMR
                     GRRSIHG-GRAEIRQVLYMAAMSAMRHEPRLRDFYRSLRARGK---EGKVAIVAVMRK
                     MLVILNARVRDAE--NGLVPAXGQ"
                     /note="IS110  e-value= 3.4e-46 complete sequence hit
                     coverage= 100%, length is 306aa with 110 gaps, 1 stops,
                     frame= Plus1"
     CDS             3623575..3624459
                     /colour="255 0 0"
                     /evidence=predicted
                     /translation="NGIGIDVSKRRLDIGTTDGQT------LQVSNSPSGYAELSTWL
                     STRP-----AIQIVLEATGGYEQTVLDFLHKA---GHPVIRANALRARRLA-QGLGKV
                     AKTDRLDALVLAQMA--ALVELPSYQPLEPW----QRKLREFVRARRQTMQALTVARQ
                     QQEMVTDRELRRQMQG------------------------------------------
                     ----------------------------NIIRLQTLVERLGKQISEQVAQ-QPQLAVL
                     KS---------MKGVGPALQAVLASYLP-ELGQISGK-AIASLVGVAPISHDSG-AMR
                     GRRSIHG-GRAEIRQVLYMAAMSAMRHEPRLRDFYRSLRARGK---EGKVAIVAVMRK
                     MLVILNARVRD"
                     /note="IS110  e-value= 1e-48 fragment hit coverage=
                     96.88%, length is 295aa with 108 gaps, 0 stops, frame=
                     Plus1"
     CDS             1896455..1896598
                     /colour="255 0 0"
                     /evidence=predicted
                     /translation="KRYTDEFKIEAVRQIVEYGRPVAEVAERLGVSIHSLYGWKRQQG
                     KGDV"
                     /note="IS3_IS407_ORF1    e-value= 7.7e-06 fragment hit
                     coverage= 55.81%, length is 48aa with 0 gaps, 0 stops,
                     frame= Plus2"
     CDS             1896608..1896736
                     /colour="255 0 0"
                     /evidence=predicted
                     /translation="VEQDQNAEVRRLKAELRRVTEERDILK--KAAAYFAKGXEQSTP
                     S"
                     /note="IS3_IS51_ORF1     e-value= 3.2e-08 fragment hit
                     coverage= 14.66%, length is 43aa with 2 gaps, 1 stops,
                     frame= Plus2"
     CDS             2371592..2372467
                     /colour="255 0 0"
                     /evidence=predicted
                     /translation="FGIGIDVSKATLDVAVHGEQ------FRQFSNDKRGFVRLIRWL
                     KTWP-----IKQVVLEASGGYERAALDVLHAA---GLPMVRINPARARRFAQGT-GRA
                     AKTDRLDATVLAQMAHLLPLTRYVP--PAPW---QQRLAEFAQCRRQLVQMRVSEMQR
                     LRRLVDPDLIA-----------------------------------------------
```

```
                     ----------------------------MKQRHIAQLSDDLKQLDKAIAEQLEGQPGW
                     KNIGA------LKGVGPILISTLACELP-ELGSLGSK-AIASLVGLAPMNRESG-TWQ
                     GQRRISG-GRAVVREALYMAALTAIRYEPRLRAFYAGLKAKGK---ASKVALVAVMRK
                     MLVILNAR"
                     /note="IS110 e-value= 1.4e-51 fragment hit coverage=
                     96.15%, length is 292aa with 108 gaps, 0 stops, frame=
                     Plus2"
     CDS             2371592..2372530
                     /colour="255 0 0"
                     /evidence=predicted
                     /translation="FGIGIDVSKATLDVAVHGEQ------FRQFSNDKRGFVRLIRWL
                     KTWP-----IKQVVLEASGGYERAALDVLHAA---GLPMVRINPARARRFAQGT-GRA
                     AKTDRLDATVLAQMAHLLPLTRYVP--PAPW---QQRLAEFAQCRRQLVQMRVSEMQR
                     LRRLVDPDLIA-----------------------------------------------
                     ----------------------------MKQRHIAQLSDDLKQLDKAIAEQLEGQPGW
                     KNIGA------LKGVGPILISTLACELP-ELGSLGSK-AIASLVGLAPMNRESG-TWQ
                     GQRRISG-GRAVVREALYMAALTAIRYEPRLRAFYAGLKAKGK---ASKVALVAVMRK
                     MLVILNARkrdaeVALGCPXQDSCYFSAS"
                     /note="IS110 e-value= 1.7e-49 complete sequence hit
                     coverage= 100%, length is 313aa with 108 gaps, 1 stops,
                     frame= Plus2"
     CDS             2448206..2448475
                     /colour="255 0 0"
                     /evidence=predicted
                     /translation="KKRFSEEQIIGFLREAESG-----------------MPIKDLCR
                     QHGFSEASYYLWRSKFGGMSVPDAKRHKDLEAENTRLKKLLAEQVFENDVIKDALRKK
                     WXPR-R"
                     /note="IS3_IS150_ORF1    e-value= 1.8e-12 fragment hit
                     coverage= 35.29%, length is 90aa with 18 gaps, 1 stops,
                     frame= Plus2"
     CDS             2448206..2448463
                     /colour="255 0 0"
                     /evidence=predicted
                     /translation="KKRFSEEQIIGFLREAESGMPIKDLCRQHGFSEASYYLWRSKFG
                     GMSVPDAKRHKDLEAENTRLKKLLAEQVFENDVIKDALRKKW"
                     /note="IS3_IS407_ORF1    e-value= 3.2e-46 complete sequence
                     hit coverage= 100%, length is 86aa with 0 gaps, 0 stops,
                     frame= Plus2"
     CDS             2448206..2448463
                     /colour="255 0 0"
                     /evidence=predicted
                     /translation="KKRFSEEQIIGFLREAESGMPIKDLCRQHGFSEASYYLWRSKFG
                     GMSVPDAKRHKDLEAENTRLKKLLAEQVFENDVIKDALRKKW"
                     /note="IS3_IS407_ORF1    e-value= 8.1e-47 complete sequence
                     hit coverage= 100%, length is 86aa with 0 gaps, 0 stops,
                     frame= Plus2"
     CDS             2756306..2757181
                     /colour="255 0 0"
                     /evidence=predicted
                     /translation="FGIGIDVSKATLDVAVHGEQ------FRQFSNDKRGFVRLIRWL
                     KTWP-----IKQVVLEASGGYERAALDVLHAA---GLPMVRINPARARRFAQGT-GRA
                     AKTDRLDATVLAQMAHLLPLTRYVP--PAPW---QQRLAEFAQCRRQLVQMRVSEMQR
                     LRRLVDPDLIA-----------------------------------------------
                     ----------------------------MKQRHIAQLSDDLKQLDKAIAEQLEGQPGW
                     KNIGA------LKGVGPILISTLACELP-ELGSLGSK-AIASLVGLAPMNRESG-TWQ
                     GQRRISG-GRAVVREALYMAALTAIRYEPRLRAFYAGLKAKGK---ASKVALVAVMRK
                     MLVILNAR"
                     /note="IS110 e-value= 1.4e-51 fragment hit coverage=
                     96.15%, length is 292aa with 108 gaps, 0 stops, frame=
                     Plus2"
```

```
     CDS             2756306..2757214
                     /colour="255 0 0"
                     /evidence=predicted
                     /translation="FGIGIDVSKATLDVAVHGEQ------FRQFSNDKRGFVRLIRWL
                     KTWP-----IKQVVLEASGGYERAALDVLHAA---GLPMVRINPARARRFAQGT-GRA
                     AKTDRLDATVLAQMAHLLPLTRYVP--PAPW---QQRLAEFAQCRRQLVQMRVSEMQR
                     LRRLVDPDLIA-----------------------------------------------
                     ----------------------------MKQRHIAQLSDDLKQLDKAIAEQLEGQPGW
                     KNIGA------LKGVGPILISTLACELP-ELGSLGSK-AIASLVGLAPMNRESG-TWQ
                     GQRRISG-GRAVVREALYMAALTAIRYEPRLRAFYAGLKAKGK---ASKVALVAVMRK
                     MLVILNARKR-----DAEVALSCP"
                     /note="IS110  e-value= 3.1e-48 complete sequence hit
                     coverage= 100%, length is 303aa with 113 gaps, 0 stops,
                     frame= Plus2"
     CDS             3087821..3088948
                     /colour="255 0 0"
                     /evidence=predicted
                     /translation="---QAMRSVHVEAKevQRRVQAWCggAGKPVGCELRPGSPGAGn
                     pgyVTDTLEAXGAGSGVSRICWCRDAPGRGACAVEARTSPGE-EGAGFFARSGDvlcq
                     gIILRYQVIERCRDEFPVRLMCRCLKVSASGYYDWSKRLPSAGQLENQRLLDQIRALH
                     EDSRGTLGAGRMQEDLA---DAGHSLSLNRVARLMAADGLQGWPRPK-----RRGQQG
                     RPAltlpgvRNRLERDFSALEPETKWVTDITEIKTQ-QSKLYLCIVLDLFDQRVVGWS
                     MQHRQDRQMVIRAVQMAVWQRQESYEVILHS-----DRGTQFRSGDYQRYLAANSLVC
                     SMSAVGHCGDNAACEGFFGLLKRERIY--RTSYPTLDAARADVFDYIE----RRHNPR
                     M--------RRRVARQ"
                     /note="IS3_IS150_ORF2    e-value= 7.4e-24 complete sequence
                     hit coverage= 100%, length is 376aa with 32 gaps, 1
                     stops, frame= Plus2"
     CDS             3088076..3088975
                     /colour="255 0 0"
                     /evidence=predicted
                     /translation="AGFFARSGDVLCQgIILRYQVIERCRDEFPVRLMCRCLKvSASG
                     YYDWSKRLPSAGQLEnQRLLDQIRALHEDSRGTLGAGRMQEDLADAGHSLSLNRVARL
                     MAADGLQG-WPRPK--RRGQQGRPALTLpgVRNRLERDFSALEPETKWVTDITEIKT-
                     -QQSKLYLCIVLDLFDQRVVGWSMQHRQDRQMVIRAVQMAVWQ-RQESYEVILHSDRG
                     TQFRSGDYQRYLAANSLVCSMSAVGHCGDNAACEGFFGLLKRERIYRTSYPTLD--AA
                     RADVFDYIERRHNP---RMRRRVARQDQKVAALLE"
                     /note="IS3_IS150_ORF1    e-value= 6e-28 complete sequence
                     hit coverage= 100%, length is 300aa with 11 gaps, 0
                     stops, frame= Plus2"
     CDS             3088112..3089023
                     /colour="255 0 0"
                     /evidence=predicted
                     /translation="QGIILRYQVIERCRD--EFPVRLMCRCLKVS---ASGYYDWSKR
                     LPSAGQLENQRLLDQIRALHEDSRG-----TLGAGRMQEDLADAGHSLSLNRVARLMA
                     ADGLQGW--PRPKRRGQQGRPALTLPgvrNRLERDFSALEPETKWVTDITEIKTQQSK
                     LYLCIVLDLFDQRVVGWSMQHRQDRQMVIRAVQMAVWQRQES-----YEVILHSDRGT
                     QFRSGDYQRYLAANSLVCSMSAVGHCGDNAACEGFFGLLKRERIYRTSYPTLDAARAD
                     VFDYIERRHNPRmRRRVARQDQKVAALLEPSVISGXNPIVPSAAX"
                     /note="IS3_IS51_ORF2     e-value= 9.2e-49 complete sequence
                     hit coverage= 100%, length is 304aa with 17 gaps, 2
                     stops, frame= Plus2"
     CDS             3088130..3088909
                     /colour="255 0 0"
                     /evidence=predicted
                     /translation="YQVIERCRDEFPVRLMCRCLKVSASGYYDWS-----KRLPSAGQ
                     LENQRLLDQIRALHEDSRGTLGAGRMQEDLADAGHSLSLNRVARLMAADGLQGWPRPK
                     RRgQQGRPALTLPGVRNRLERDFSALEPETKWVTDITEIKTQQSKLYLCIVLDLFDQR
                     VVGWSMQHRQDRQMV-IRAVQMAVWQRQESY----EVILHSDRGTQFRSGDYQRYLAA
                     NSLVCSMSAVGHCGDNAACEGFFGLLKRERIYR----TSYPTLDAARADVFDYIER"
```

/note="IS3_IS51_ORF1   e-value= 3.8e-62 fragment hit
coverage= 88.93%, length is 260aa with 14 gaps, 0 stops,
frame= Plus2"
CDS 3088130..3089008
/colour="255 0 0"
/evidence=predicted
/translation="YQVIERCRDEFPVRLMCRCLKVSASGYYDWS-----KRLPSAGQ
LENQRLLDQIRALHEDSRGTLGAGRMQEDLADAGHSLSLNRVARLMAADGLQGWPRPK
RRgQQGRPALTLPGVRNRLERDFSALEPETKWVTDITEIKTQQSKLYLCIVLDLFDQR
VVGWSMQHRQDRQMV-IRAVQMAVWQRQESY----EVILHSDRGTQFRSGDYQRYLAA
NSLVCSMSAVGHCGDNAACEGFFGLLKRERIYR----TSYPTLDAARADVFDYIERRH
NprMRRRVARQDQKVAALLE---PSVISGXNPIV"
/note="IS3_IS51_ORF1   e-value= 1.3e-54 complete sequence
hit coverage= 100%, length is 293aa with 17 gaps, 1
stops, frame= Plus2"
CDS 3088133..3088906
/colour="255 0 0"
/evidence=predicted
/translation="QVIERCRDEFPVRLMCRCLKVSASGYYDWSKRLPSAGQLENQRL
LDQIRALHEDSRGTLGAGRMQEDLA---DAGHSLSLNRVARLMAADGLQGWPRPK---
--RRGQQGRPAltlpgvRNRLERDFSALEPETKWVTDITEIKTQ-QSKLYLCIVLDLF
DQRVVGWSMQHRQDRQMVIRAVQMAVWQRQESYEVILHS-----DRGTQFRSGDYQRY
LAANSLVCSMSAVGHCGDNAACEGFFGLLKRERIY--RTSYPTLDAARADVFDYIE"
/note="IS3_IS150_ORF2   e-value= 4.1e-56 fragment hit
coverage= 68.89%, length is 258aa with 16 gaps, 0 stops,
frame= Plus2"
CDS 3088133..3088906
/colour="255 0 0"
/evidence=predicted
/translation="QVIERCRDEFPVRLMCRCLKVSASGYYDWSKR--LPSAGQLENQ
RLLDQIRALHEDSRGTLGAGRMQEDLA-DAGHSLSLNRVARLMAADGLQGWPRPKRRG
QQGRpALTlPGVRNRLERDFSALEPETKWVTDITEIKTQQSKLYLCIVLDLFDQRVVG
WSMQHRQDRQMVIRAVQMAVWQRQESYEVILHSDRGTQFRSGDYQRYLAANSLVCSMS
AVGHCGDNAACEGFFGLLK--RERIYRTSYPTLDAARADVFDYIE"
/note="IS3_IS3_ORF1   e-value= 8e-71 fragment hit
coverage=
91.26%, length is 258aa with 5 gaps, 0 stops, frame=
Plus2"
CDS 3088133..3088966
/colour="255 0 0"
/evidence=predicted
/translation="QVIERCRDEFPVRLMCRCLKVSASGYYDWSKR--LPSAGQLENQ
RLLDQIRALHEDSRGTLGAGRMQEDLA-DAGHSLSLNRVARLMAADGLQGWPRPKRRG
QQGRpALTlPGVRNRLERDFSALEPETKWVTDITEIKTQQSKLYLCIVLDLFDQRVVG
WSMQHRQDRQMVIRAVQMAVWQRQESYEVILHSDRGTQFRSGDYQRYLAANSLVCSMS
AVGHCGDNAACEGFFGLLK--RERIYRTSYPTLDAARADVFDYIERRHNP-RMRRRVA
----RQDQKVAA"
/note="IS3_IS3_ORF1   e-value= 1.3e-67 complete sequence
hit coverage= 100%, length is 278aa with 10 gaps, 0
stops, frame= Plus2"
CDS 3088145..3088906
/colour="255 0 0"
/evidence=predicted
/translation="RCRDEFPVRLMCRCLKVSASGYYDWSKRLPSAGQLENQRLLDQI
RALHEDSRGTLGAGRMQEDLADAGHSLSLNRVARLMAADGLQGWPRPKRRGQQGR--P
ALTLPgvrNRLERDFSALE--PETKWVTDITEIKTQQSKLYLCIVLDLFDQRVVGWSM
QHRQDRQMVIRAVQMAVWQ----RQESYEVILHSDRGTQFRSGDYQRYLAANSLVCSM
SAVGHCGDNAACEGFFGLLKRERIYRTSYPTLDAARADVFDYIE"
/note="IS3_IS3_ORF2   e-value= 4.4e-65 fragment hit
coverage= 89.93%, length is 254aa with 8 gaps, 0 stops,

```
                    frame= Plus2"
CDS                 3088145..3088963
                    /colour="255 0 0"
                    /evidence=predicted
                    /translation="RCRDEFPVRLMCRCLKVSASGYYDWSKRLPSAGQLENQRLLDQI
                    RALHEDSRGTLGAGRMQEDLADAGHSLSLNRVARLMAADGLQGWPRPKRRGQQGR--P
                    ALTLPgvrNRLERDFSALE--PETKWVTDITEIKTQQSKLYLCIVLDLFDQRVVGWSM
                    QHRQDRQMVIRAVQMAVWQ----RQESYEVILHSDRGTQFRSGDYQRYLAANSLVCSM
                    SAVGHCGDNAACEGFFGLLKRERIYRTSYPTLDAARADVFDYIERRHNpRMRRRVA--
                    ---------RQDQKVA"
                    /note="IS3_IS3_ORF2      e-value= 9.6e-61 complete sequence
                    hit coverage= 100%, length is 273aa with 19 gaps, 0
                    stops, frame= Plus2"
CDS                 3088166..3088921
                    /colour="255 0 0"
                    /evidence=predicted
                    /translation="VRLMCRCLKVS---ASGYYDWSKRLPSAGQLENQRLLDQIRALH
                    EDSRG-----TLGAGRMQEDLADAGHSLSLNRVARLMAADGLQGW--PRPKRRGQQGR
                    PALTLPgvrNRLERDFSALEPETKWVTDITEIKTQQSKLYLCIVLDLFDQRVVGWSMQ
                    HRQDRQMVIRAVQMAVWQRQES-----YEVILHSDRGTQFRSGDYQRYLAANSLVCSM
                    SAVGHCGDNAACEGFFGLLKRERIYRTSYPTLDAARADVFDYIERRHNP"
                    /note="IS3_IS51_ORF2     e-value= 3.9e-65 fragment hit
                    coverage= 83.28%, length is 252aa with 15 gaps, 0 stops,
                    frame= Plus2"
CDS                 3088184..3088906
                    /colour="255 0 0"
                    /evidence=predicted
                    /translation="CLKVSASGYYDWSKRLPSAGQLEnQRLLDQIRALHEDSRGTLGA
                    GRMQEDLADAGHSLSLNRVARLMAADGLQG-WPRPK--RRGQQGRPALTLpgVRNRLE
                    RDFSALEPETKWVTDITEIKT--QQSKLYLCIVLDLFDQRVVGWSMQHRQDRQMVIRA
                    VQMAVWQ-RQESYEVILHSDRGTQFRSGDYQRYLAANSLVCSMSAVGHCGDNAACEGF
                    FGLLKRERIYRTSYPTLD--AARADVFDYIE"
                    /note="IS3_IS150_ORF1    e-value= 7.6e-35 fragment hit
                    coverage= 80.39%, length is 241aa with 8 gaps, 0 stops,
                    frame= Plus2"
CDS                 3595427..3596512
                    /colour="255 0 0"
                    /evidence=predicted
                    /translation="MAAAVCSRrcpSVGTASAAAPKADEKDpFVQAA--XGNEPRXAA
                    QGSRLSACGDRlPKKtRCLDPGRAGGARRKaqAIQGLRQVHTLSLL--LEAAELSRST
                    FYYQnhvlAHPDQDEAALCERIRAIYdQSQGRYGYRTVTLELANQGHRTNHKRVQRLM
                    GEMGLKSRVRV-KRYRSFKGAANVVVgndlnrqfhAERPNQKW----VTDVTefkVQG
                    MKLYLSPIMDLYNGEIVAYQIKRQPVFDLVGQMLEEAIKK-LSPDerpMIHSDQGWQY
                    QHENYRHMLEKHSLKQSMSRRGNCLDNAAMESFFGTLKSEF--FYLnsFDSIESLEAG
                    LVEYIQYYNQERIKLKLKGLSPVEYRERAQSAAXPRPSLG"
                    /note="IS3_IS407_ORF2    e-value= 7.1e-09 complete sequence
                    hit coverage= 100%, length is 362aa with 12 gaps, 3
                    stops, frame= Plus2"
CDS                 3595433..3596494
                    /colour="255 0 0"
                    /evidence=predicted
                    /translation="AAVCSRRCPS-------VGTASAAAPKAD---EKDPFVQAAXGN
                    EPRXAAQGS-----RLSACGD---RLPKKTRCLDPG------RAGGARRKAQAIQGLR
                    QVHTLSLLLEAAELSRSTFYYQNHVL--AHPDqDEAALCERIRAIYDQSQGRYGYRTV
                    TLELA---NQGHRTNHKRVQRLMGEMGLKSRVRVK-RYRSFKGAANVVVGNDLNRQFH
                    AERPNQKWVTDVTEFKVQ-GMKLYLSPIMDLYNGEIVAYQIKRQPVFDLVGQMLEEAI
                    KKLSPDERPMIHS-----DQGWQYQHENYRHMLEKHSLKQSMSRRGNCLDNAAMESFF
                    GTLKSEFFY--LNSFDSIESLEAGLVEYIQYYNQERIKLKLKGLSPVEYreRAQSAAX
                    "
```

/note="IS3_IS150_ORF2   e-value= 2.1e-122 complete
sequence
hit coverage= 100%, length is 354aa with 38 gaps, 3
stops, frame= Plus2"
CDS 3595586..3596485
/colour="255 0 0"
/evidence=predicted
/translation="LPKKTRCLDPGRAggARRKAQAIQGLRQVHTLSLLLEAAELSRS
TFYYQNHVLAHPDqDEAALCERIRAIYDQSQGRYGYRTVTLELANQGHRTNHKRVQRL
MGEMGLKSRVRVK---RYRSFKGAANVVVGNDLNRQFHAERPNQKWVTDVTEFKV--Q
GMKLYLSPIMDLYNGEIVAYQIKRQPVFDLVGQMLEEAIKK-LSPDERPMIHSDQGWQ
YQHENYRHMLEKHSLKQSMSRRGNCLDNAAMESFFGTLKSEFFYLNSFDSIE--SLEA
GLVEYIQYYNQE-RIKLKLKGLSPVEYRERAQS"
/note="IS3_IS150_ORF1   e-value= 1.9e-87 complete sequence
hit coverage= 100%, length is 300aa with 9 gaps, 0 stops,
frame= Plus2"
CDS 3595586..3596485
/colour="255 0 0"
/evidence=predicted
/translation="LPKKTRCLDPGRAggARRKAQAIQGLRQVHTLSLLLEAAELSRS
TFYYQNHVLAHPDqDEAALCERIRAIYDQSQGRYGYRTVTLELANQGHRTNHKRVQRL
MGEMGLKSRVRVK---RYRSFKGAANVVVGNDLNRQFHAERPNQKWVTDVTEFKV--Q
GMKLYLSPIMDLYNGEIVAYQIKRQPVFDLVGQMLEEAIKK-LSPDERPMIHSDQGWQ
YQHENYRHMLEKHSLKQSMSRRGNCLDNAAMESFFGTLKSEFFYLNSFDSIE--SLEA
GLVEYIQYYNQE-RIKLKLKGLSPVEYRERAQS"
/note="IS3_IS150_ORF1   e-value= 4.9e-88 complete sequence
hit coverage= 100%, length is 300aa with 9 gaps, 0 stops,
frame= Plus2"
CDS 3595595..3596530
/colour="255 0 0"
/evidence=predicted
/translation="KTRCLDPGRAGGARRKAQA------IQGLRQVhTLSLLLEAAEL
SRS----TFYYQ-NHVLAHPDQDEAALCERIRAIY-DQsqgRYGY-RTVTLELANQGH
R---TNHKRVQRLMGEMGLKSRVRVKRYRSFKGAANVVVGNDLNRQFHAERPNQKWVT
DVTE-------------FKVQG--------MKLYLSPIMDLYNGEIVAYQIKRQPVFD
LVGQMLEEAIKKLSPDERPM--IHSDQGWQYQHE----NYRHMLEKHSLKQSMSRRGN
CLDNAAMESFFGTLKSEFFYLNS---FDSIESLEAGLVEYIQYYNQERIKLKLKGLSP
VeyrERAQSAAXPRPSLGGHFTLG"
/note="IS481 e-value= 4.9e-06 complete sequence hit
coverage= 100%, length is 312aa with 46 gaps, 1 stops,
frame= Plus2"
CDS 3595634..3596494
/colour="255 0 0"
/evidence=predicted
/translation="RRKAQAIQGLRQVHTLSLLLEAAELSRSTFYYQNHVL--AHPDq
DEAALCERIRAIYDQSQGRYGYRTVTLELA---NQGHRTNHKRVQRLMGEMGLKSRVR
VK-RYRSFKGAANVVVGNDLNRQFHAERPNQKWVTDVTEFKVQ-GMKLYLSPIMDLYN
GEIVAYQIKRQPVFDLVGQMLEEAIKKLSPDERPMIHS-----DQGWQYQHENYRHML
EKHSLKQSMSRRGNCLDNAAMESFFGTLKSEFFY--LNSFDSIESLEAGLVEYIQYYN
QERIKLKLKGLSPVEYreRAQSAAX"
/note="IS3_IS150_ORF2   e-value= 2.4e-152 fragment hit
coverage= 76.61%, length is 287aa with 14 gaps, 1 stops,
frame= Plus2"
CDS 3595643..3596515
/colour="255 0 0"
/evidence=predicted
/translation="AQAIQGLRQVHTLSLLLEAAELSRSTFYYQNHVLAHPD------
QDEAALCERIRAIYDQSQGRYGYRTVTLELANQGHRTNHKRVQRLMGEMGLKSRVRVK
RYRSFKGAANVVVG-NDLNRQFHAERPNQKWVTDVTEFKV-QGmKLYLSPIMDLYNGE
IVAYQIKRQPVFDLV-GQMLEEAIKKLSPDE----RPMIHSDQGWQYQHENYRHMLEK

```
                    HSLKQSMSRRGNCLDNAAMESFFGTLKSEFFYL----NSFDSIESLEAGLVEYIQYYN
                    Q-ERIKLKLKGLSPVEYrERAQSAAXPRPSLGG"
                    /note="IS3_IS51_ORF1     e-value= 8.4e-39 complete sequence
                    hit coverage= 100%, length is 291aa with 18 gaps, 1
                    stops, frame= Plus2"
CDS                 3595643..3596518
                    /colour="255 0 0"
                    /evidence=predicted
                    /translation="---AQAIQGLR-QVHTLSLLLEA--AELSRS------TFYYQNH
                    VLAHPDQDEAALCERIRAIYDQSQG-----RYGYRTVTLELANQGHRTNHKRVQRLMG
                    EMGLKSRVRVKRYRSFKG-AANVVVGNDLNRQFHAERPNQKWVTDVTEFKVQGMKLYL
                    SPIMDLYNGEIVAYQIKRQPVFDLVGQMLEEAIKKLSPD-----ERPMIHSDQGWQYQ
                    HENYRHMLEKHSLKQSMSRRGNCLDNAAMESFFGTLKSEFFYLNSFDSIESLEAGLVE
                    YIQ-YYNQERIKLKLKGLSPVEYRER-AQSAAXPRPSLGGH"
                    /note="IS3_IS51_ORF2     e-value= 7.9e-18 complete sequence
                    hit coverage= 100%, length is 292aa with 25 gaps, 1
                    stops, frame= Plus2"
CDS                 3595646..3596482
                    /colour="255 0 0"
                    /evidence=predicted
                    /translation="QAIQGLRQVHTLSLLLEAAELSRSTFYYQNHVLAHPD---QDEA
                    ALCERIRAIYDQSQGRYGYRTVTLELA-NQGHRTNHKRVQRLMGEMGLKSRVRV-KRY
                    RSFKgAANVVVGNDLNRQFHAERPNQKWVTDVTEFKVQGMKLYLSPIMDLYNGEIVAY
                    QIKRQPVFDLVGQMLEEAIKKLSPDERPMIHSDQGWQYQHENYRHMLEKHSLKQSMSR
                    RGNCLDNAAMESFFGTLK--SEFFYLNSFDSIESLEAGLVEYIQ-YYNQERIKLKLKG
                    LSPVEYRERAQ"
                    /note="IS3_IS3_ORF1      e-value= 2.2e-75 complete sequence
                    hit coverage= 100%, length is 279aa with 8 gaps, 0 stops,
                    frame= Plus2"
CDS                 3595646..3596482
                    /colour="255 0 0"
                    /evidence=predicted
                    /translation="QAIQGLRQVHTLSLLLEAAELSRSTFYYQNHVLAHPD---QDEA
                    ALCERIRAIYDQSQGRYGYRTVTLELA-NQGHRTNHKRVQRLMGEMGLKSRVRV-KRY
                    RSFKgAANVVVGNDLNRQFHAERPNQKWVTDVTEFKVQGMKLYLSPIMDLYNGEIVAY
                    QIKRQPVFDLVGQMLEEAIKKLSPDERPMIHSDQGWQYQHENYRHMLEKHSLKQSMSR
                    RGNCLDNAAMESFFGTLK--SEFFYLNSFDSIESLEAGLVEYIQ-YYNQERIKLKLKG
                    LSPVEYRERAQ"
                    /note="IS3_IS3_ORF1      e-value= 5.9e-76 complete sequence
                    hit coverage= 100%, length is 279aa with 8 gaps, 0 stops,
                    frame= Plus2"
CDS                 3595658..3596494
                    /colour="255 0 0"
                    /evidence=predicted
                    /translation="GLRQVHTLSLLLEAAELSRSTFYYQNHV-LAHPDQDEAALCERI
                    RAIYDQSQGRYGYRTVTLELANQGHRTNHKRVQRLMGEMGLKSRVRVKRYRSFKGAAN
                    VVVG-NDLNRQFHAER--PNQKWVTDVTEFKVQGMKLYLSPIMDLYNGEIVAYQIKRQ
                    PVFDLVGQMLEEAIKK----LSPDERPMIHSDQGWQYQHENYRHMLEKHSLKQSMSRR
                    GNCLDNAAMESFFGTLKSEFFYLNSFDSIESLEAGLVEYIQYY-NQERIKLKLKGLSP
                    VEYRERAQSAAX"
                    /note="IS3_IS3_ORF2      e-value= 3.9e-64 complete sequence
                    hit coverage= 100%, length is 279aa with 9 gaps, 1 stops,
                    frame= Plus2"
CDS                 3595658..3596494
                    /colour="255 0 0"
                    /evidence=predicted
                    /translation="GLRQVHTLSLLLEAAELSRSTFYYQNHV-LAHPDQDEAALCERI
                    RAIYDQSQGRYGYRTVTLELANQGHRTNHKRVQRLMGEMGLKSRVRVKRYRSFKGAAN
                    VVVG-NDLNRQFHAER--PNQKWVTDVTEFKVQGMKLYLSPIMDLYNGEIVAYQIKRQ
                    PVFDLVGQMLEEAIKK----LSPDERPMIHSDQGWQYQHENYRHMLEKHSLKQSMSRR
```

```
                     GNCLDNAAMESFFGTLKSEFFYLNSFDSIESLEAGLVEYIQYY-NQERIKLKLKGLSP
                     VEYRERAQSAAX"
                     /note="IS3_IS3_ORF2      e-value= 1.1e-64 complete sequence
                     hit coverage= 100%, length is 279aa with 9 gaps, 1 stops,
                     frame= Plus2"
     CDS             3595766..3596467
                     /colour="255 0 0"
                     /evidence=predicted
                     /translation="AALCERIRAIYDQSQGRYGYRTVTLELANQGHRTNHKRVQRLMG
                     EMGLKSRVRVKRYRSFKGAANVVVG-NDLNRQFHAERPNQKWVTDVTEFKV-QGmKLY
                     LSPIMDLYNGEIVAYQIKRQPVFDLV-GQMLEEAIKKLSPDE----RPMIHSDQGWQY
                     QHENYRHMLEKHSLKQSMSRRGNCLDNAAMESFFGTLKSEFFYL----NSFDSIESLE
                     AGLVEYIQYYNQ-ERIKLKLKGLSPVEY"
                     /note="IS3_IS51_ORF1     e-value= 4.9e-45 fragment hit
                     coverage= 79.80%, length is 234aa with 12 gaps, 0 stops,
                     frame= Plus2"
     CDS             3595817..3596344
                     /colour="255 0 0"
                     /evidence=predicted
                     /translation="YGYRTVTLELANQGHRTNHKRVQRLMGEMGLKSRVRVKRYRSFK
                     G-AANVVVGNDLNRQFHAERPNQKWVTDVTEFKVQGMKLYLSPIMDLYNGEIVAYQIK
                     RQPVFDLVGQMLEEAIKKLSPD-----ERPMIHSDQGWQYQHENYRHMLEKHSLKQSM
                     SRRGNCLDNAAMESFFGTLKSE"
                     /note="IS3_IS51_ORF2     e-value= 5.2e-32 fragment hit
                     coverage= 57.41%, length is 176aa with 6 gaps, 0 stops,
                     frame= Plus2"
     CDS             3596321..3596428
                     /colour="255 0 0"
                     /evidence=predicted
                     /translation="FFGTLKSEFFYLNS---FDSIESLEAGLVEYIQYYNQER"
                     /note="IS481 e-value= 3.8e-07 fragment hit coverage=
                     11.11%, length is 36aa with 3 gaps, 0 stops, frame=
                     Plus2"
     CDS             4365179..4365262
                     /colour="255 0 0"
                     /evidence=predicted
                     /translation="LRRASPSRGPARQRHGEDAEVVDALRHL"
                     /note="IS3_IS2_ORF2      e-value= 9.6e-06 fragment hit
                     coverage= 9.27%, length is 28aa with 0 gaps, 0 stops,
                     frame= Plus2"
     CDS             1355496..1356572
                     /colour="255 0 0"
                     /evidence=predicted
                     /translation="SXTFVDCLSRTFMEKEDARKQSREVLHERRKQVIRLHRKGVGVM
                     QIVEQTGLSWSGVNTALRLFEQGGAAALKPHARGKKPGSGRSLSTEQERVIRMTIIDK
                     RPEQIKMEFALWSRPAVRELIERDLGNKLSVRAVGDYLARWGFTPQKSIKKAY--EQR
                     PEAVQAWLEDEYPTIEARAKREGAEIHWGDETALVNTDVRGR-SYAPAG---------
                     ---QTPVTRAVGGTRQKLSMIATVTNQGK---TRWMIIDEAFNSDRLIEFMQALIKES
                     qGKKIFLILDNLRVHHSKPVKEWATQHKQDIELFYLPSYSPELNPEERLNADLKHAIG
                     SKVPVRTKAKLRAAATEHMTMLENTPERVKKYFGDKHVAYAAS"
                     /note="IS630 e-value= 2.1e-108 complete sequence hit
                     coverage= 100%, length is 359aa with 18 gaps, 1 stops,
                     frame= Plus3"
     CDS             1355532..1356572
                     /colour="255 0 0"
                     /evidence=predicted
                     /translation="MEKEDARKQSREVLHERRKQVIRLHRKGVGVMQIVEQTGLSWSG
                     VNTALRLFEQGGAAALKPHARGKKPGSGRSLSTEQERVIRMTIIDKRPEQIKMEFALW
                     SRPAVRELIERDLGNKLSVRAVGDYLARWGFTPQKSIKKAY--EQRPEAVQAWLEDEY
                     PTIEARAKREGAEIHWGDETALVNTDVRGR-SYAPAG------------QTPVTRAVG
```

```
                 GTRQKLSMIATVTNQGK---TRWMIIDEAFNSDRLIEFMQALIKESqGKKIFLILDNL
                 RVHHSKPVKEWATQHKQDIELFYLPSYSPELNPEERLNADLKHAIGSKVPVRTKAKLR
                 AAATEHMTMLENTPERVKKYFGDKHVAYAAS"
                 /note="IS630 e-value= 7.9e-108 fragment hit coverage=
                 96.81%, length is 347aa with 18 gaps, 0 stops, frame=
                 Plus3"
CDS              2334351..2335400
                 /colour="255 0 0"
                 /evidence=predicted
                 /translation="----SRRCPS-------VGTASAAAPKAD---EKDPFVQAAXGN
                 EPRXAAQGS-----RLSACGD---RLPKKTRCLDPG------RAGGARRKAQAIQGLR
                 QVHTLSLLLEAAELSRSTFYYQNHVL--AHPDqDEAALCERIRAIYDQSQGRYGYRTV
                 TLELA---NQGHRTNHKRVQRLMGEMGLKSRVRVK-RYRSFKGAANVVVGNDLNRQFH
                 AERPNQKWVTDVTEFKVQ-GMKLYLSPIMDLYNGEIVAYQIKRQPVFDLVGQMLEEAI
                 MKLSPDERPMIHS-----DQGWQYQHENYRHMLEKHSLKQSMSRRGNCLDNAAMESFF
                 GTLKSEFFY--LNSFDSIESLEAGLVEYIQYYNQERIKLKLKGLSPVEYreRAQSAAX
                 "
                 /note="IS3_IS150_ORF2    e-value= 5e-121 complete sequence
                 hit coverage= 100%, length is 350aa with 42 gaps, 3
                 stops, frame= Plus3"
CDS              2334363..2335418
                 /colour="255 0 0"
                 /evidence=predicted
                 /translation="-------PSVGTASAAAPKADEKDpFVQAA--XGNEPRXAAQGS
                 RLSACGDRlPKKtRCLDPGRAGGARRKaqAIQGLRQVHTLSLL--LEAAELSRSTFYY
                 QnhvlAHPDQDEAALCERIRAIYdQSQGRYGYRTVTLELANQGHRTNHKRVQRLMGEM
                 GLKSRVRV-KRYRSFKGAANVVVgndlnrqfhAERPNQKW----VTDVTefkVQGMKL
                 YLSPIMDLYNGEIVAYQIKRQPVFDLVGQMLEEAIMK-LSPDerpMIHSDQGWQYQHE
                 NYRHMLEKHSLKQSMSRRGNCLDNAAMESFFGTLKSEF--FYLnsFDSIESLEAGLVE
                 YIQYYNQERIKLKLKGLSPVEYRERAQSAAXPRPSLG"
                 /note="IS3_IS407_ORF2    e-value= 9.5e-09 complete sequence
                 hit coverage= 100%, length is 352aa with 19 gaps, 3
                 stops, frame= Plus3"
CDS              2334492..2335391
                 /colour="255 0 0"
                 /evidence=predicted
                 /translation="LPKKTRCLDPGRAggARRKAQAIQGLRQVHTLSLLLEAAELSRS
                 TFYYQNHVLAHPDqDEAALCERIRAIYDQSQGRYGYRTVTLELANQGHRTNHKRVQRL
                 MGEMGLKSRVRVK---RYRSFKGAANVVVGNDLNRQFHAERPNQKWVTDVTEFKV--Q
                 GMKLYLSPIMDLYNGEIVAYQIKRQPVFDLVGQMLEEAIMK-LSPDERPMIHSDQGWQ
                 YQHENYRHMLEKHSLKQSMSRRGNCLDNAAMESFFGTLKSEFFYLNSFDSIE--SLEA
                 GLVEYIQYYNQE-RIKLKLKGLSPVEYRERAQS"
                 /note="IS3_IS150_ORF1    e-value= 1.2e-86 complete sequence
                 hit coverage= 100%, length is 300aa with 9 gaps, 0 stops,
                 frame= Plus3"
CDS              2334492..2335391
                 /colour="255 0 0"
                 /evidence=predicted
                 /translation="LPKKTRCLDPGRAggARRKAQAIQGLRQVHTLSLLLEAAELSRS
                 TFYYQNHVLAHPDqDEAALCERIRAIYDQSQGRYGYRTVTLELANQGHRTNHKRVQRL
                 MGEMGLKSRVRVK---RYRSFKGAANVVVGNDLNRQFHAERPNQKWVTDVTEFKV--Q
                 GMKLYLSPIMDLYNGEIVAYQIKRQPVFDLVGQMLEEAIMK-LSPDERPMIHSDQGWQ
                 YQHENYRHMLEKHSLKQSMSRRGNCLDNAAMESFFGTLKSEFFYLNSFDSIE--SLEA
                 GLVEYIQYYNQE-RIKLKLKGLSPVEYRERAQS"
                 /note="IS3_IS150_ORF1    e-value= 3.2e-87 complete sequence
                 hit coverage= 100%, length is 300aa with 9 gaps, 0 stops,
                 frame= Plus3"
CDS              2334501..2335436
                 /colour="255 0 0"
                 /evidence=predicted
```

```
                     /translation="KTRCLDPGRAGGARRKAQA------IQGLRQVhTLSLLLEAAEL
                     SRS----TFYYQ-NHVLAHPDQDEAALCERIRAIY-DQsqgRYGY-RTVTLELANQGH
                     R---TNHKRVQRLMGEMGLKSRVRVKRYRSFKGAANVVVGNDLNRQFHAERPNQKWVT
                     DVTE------------FKVQG--------MKLYLSPIMDLYNGEIVAYQIKRQPVFD
                     LVGQMLEEAIMKLSPDERPM--IHSDQGWQYQHE----NYRHMLEKHSLKQSMSRRGN
                     CLDNAAMESFFGTLKSEFFYLNS---FDSIESLEAGLVEYIQYYNQERIKLKLKGLSP
                     VeyrERAQSAAXPRPSLGGHFTLG"
                     /note="IS481 e-value= 5e-06 complete sequence hit
                     coverage= 100%, length is 312aa with 46 gaps, 1 stops,
                     frame= Plus3"
     CDS             2334540..2335400
                     /colour="255 0 0"
                     /evidence=predicted
                     /translation="RRKAQAIQGLRQVHTLSLLLEAAELSRSTFYYQNHVL--AHPDq
                     DEAALCERIRAIYDQSQGRYGYRTVTLELA---NQGHRTNHKRVQRLMGEMGLKSRVR
                     VK-RYRSFKGAANVVVGNDLNRQFHAERPNQKWVTDVTEFKVQ-GMKLYLSPIMDLYN
                     GEIVAYQIKRQPVFDLVGQMLEEAIMKLSPDERPMIHS-----DQGWQYQHENYRHML
                     EKHSLKQSMSRRGNCLDNAAMESFFGTLKSEFFY--LNSFDSIESLEAGLVEYIQYYN
                     QERIKLKLKGLSPVEYreRAQSAAX"
                     /note="IS3_IS150_ORF2    e-value= 1.9e-151 fragment hit
                     coverage= 76.61%, length is 287aa with 14 gaps, 1 stops,
                     frame= Plus3"
     CDS             2334549..2335421
                     /colour="255 0 0"
                     /evidence=predicted
                     /translation="AQAIQGLRQVHTLSLLLEAAELSRSTFYYQNHVLAHPD------
                     QDEAALCERIRAIYDQSQGRYGYRTVTLELANQGHRTNHKRVQRLMGEMGLKSRVRVK
                     RYRSFKGAANVVVG-NDLNRQFHAERPNQKWVTDVTEFKV-QGmKLYLSPIMDLYNGE
                     IVAYQIKRQPVFDLV-GQMLEEAIMKLSPDE----RPMIHSDQGWQYQHENYRHMLEK
                     HSLKQSMSRRGNCLDNAAMESFFGTLKSEFFYL----NSFDSIESLEAGLVEYIQYYN
                     Q-ERIKLKLKGLSPVEYrERAQSAAXPRPSLGG"
                     /note="IS3_IS51_ORF1     e-value= 8.5e-39 complete sequence
                     hit coverage= 100%, length is 291aa with 18 gaps, 1
                     stops, frame= Plus3"
     CDS             2334549..2335424
                     /colour="255 0 0"
                     /evidence=predicted
                     /translation="---AQAIQGLR-QVHTLSLLLEA--AELSRS------TFYYQNH
                     VLAHPDQDEAALCERIRAIYDQSQG-----RYGYRTVTLELANQGHRTNHKRVQRLMG
                     EMGLKSRVRVKRYRSFKG-AANVVVGNDLNRQFHAERPNQKWVTDVTEFKVQGMKLYL
                     SPIMDLYNGEIVAYQIKRQPVFDLVGQMLEEAIMKLSPD-----ERPMIHSDQGWQYQ
                     HENYRHMLEKHSLKQSMSRRGNCLDNAAMESFFGTLKSEFFYLNSFDSIESLEAGLVE
                     YIQ-YYNQERIKLKLKGLSPVEYRER-AQSAAXPRPSLGGH"
                     /note="IS3_IS51_ORF2     e-value= 1.2e-17 complete sequence
                     hit coverage= 100%, length is 292aa with 25 gaps, 1
                     stops, frame= Plus3"
     CDS             2334552..2335388
                     /colour="255 0 0"
                     /evidence=predicted
                     /translation="QAIQGLRQVHTLSLLLEAAELSRSTFYYQNHVLAHPD---QDEA
                     ALCERIRAIYDQSQGRYGYRTVTLELA-NQGHRTNHKRVQRLMGEMGLKSRVRV-KRY
                     RSFKgAANVVVGNDLNRQFHAERPNQKWVTDVTEFKVQGMKLYLSPIMDLYNGEIVAY
                     QIKRQPVFDLVGQMLEEAIMKLSPDERPMIHSDQGWQYQHENYRHMLEKHSLKQSMSR
                     RGNCLDNAAMESFFGTLK--SEFFYLNSFDSIESLEAGLVEYIQ-YYNQERIKLKLKG
                     LSPVEYRERAQ"
                     /note="IS3_IS3_ORF1      e-value= 1.8e-74 complete sequence
                     hit coverage= 100%, length is 279aa with 8 gaps, 0 stops,
                     frame= Plus3"
     CDS             2334552..2335388
                     /colour="255 0 0"
```

```
                     /evidence=predicted
                     /translation="QAIQGLRQVHTLSLLLEAAELSRSTFYYQNHVLAHPD---QDEA
                     ALCERIRAIYDQSQGRYGYRTVTLELA-NQGHRTNHKRVQRLMGEMGLKSRVRV-KRY
                     RSFKgAANVVVGNDLNRQFHAERPNQKWVTDVTEFKVQGMKLYLSPIMDLYNGEIVAY
                     QIKRQPVFDLVGQMLEEAIMKLSPDERPMIHSDQGWQYQHENYRHMLEKHSLKQSMSR
                     RGNCLDNAAMESFFGTLK--SEFFYLNSFDSIESLEAGLVEYIQ-YYNQERIKLKLKG
                     LSPVEYRERAQ"
                     /note="IS3_IS3_ORF1      e-value= 4.6e-75 complete sequence
                     hit coverage= 100%, length is 279aa with 8 gaps, 0 stops,
                     frame= Plus3"
     CDS             2334564..2335400
                     /colour="255 0 0"
                     /evidence=predicted
                     /translation="GLRQVHTLSLLLEAAELSRSTFYYQNHV-LAHPDQDEAALCERI
                     RAIYDQSQGRYGYRTVTLELANQGHRTNHKRVQRLMGEMGLKSRVRVKRYRSFKGAAN
                     VVVG-NDLNRQFHAER--PNQKWVTDVTEFKVQGMKLYLSPIMDLYNGEIVAYQIKRQ
                     PVFDLVGQMLEEAIMK----LSPDERPMIHSDQGWQYQHENYRHMLEKHSLKQSMSRR
                     GNCLDNAAMESFFGTLKSEFFYLNSFDSIESLEAGLVEYIQYY-NQERIKLKLKGLSP
                     VEYRERAQSAAX"
                     /note="IS3_IS3_ORF2      e-value= 1.2e-63 complete sequence
                     hit coverage= 100%, length is 279aa with 9 gaps, 1 stops,
                     frame= Plus3"
     CDS             2334564..2335400
                     /colour="255 0 0"
                     /evidence=predicted
                     /translation="GLRQVHTLSLLLEAAELSRSTFYYQNHV-LAHPDQDEAALCERI
                     RAIYDQSQGRYGYRTVTLELANQGHRTNHKRVQRLMGEMGLKSRVRVKRYRSFKGAAN
                     VVVG-NDLNRQFHAER--PNQKWVTDVTEFKVQGMKLYLSPIMDLYNGEIVAYQIKRQ
                     PVFDLVGQMLEEAIMK----LSPDERPMIHSDQGWQYQHENYRHMLEKHSLKQSMSRR
                     GNCLDNAAMESFFGTLKSEFFYLNSFDSIESLEAGLVEYIQYY-NQERIKLKLKGLSP
                     VEYRERAQSAAX"
                     /note="IS3_IS3_ORF2      e-value= 3.3e-64 complete sequence
                     hit coverage= 100%, length is 279aa with 9 gaps, 1 stops,
                     frame= Plus3"
     CDS             2334672..2335373
                     /colour="255 0 0"
                     /evidence=predicted
                     /translation="AALCERIRAIYDQSQGRYGYRTVTLELANQGHRTNHKRVQRLMG
                     EMGLKSRVRVKRYRSFKGAANVVVG-NDLNRQFHAERPNQKWVTDVTEFKV-QGmKLY
                     LSPIMDLYNGEIVAYQIKRQPVFDLV-GQMLEEAIMKLSPDE----RPMIHSDQGWQY
                     QHENYRHMLEKHSLKQSMSRRGNCLDNAAMESFFGTLKSEFFYL----NSFDSIESLE
                     AGLVEYIQYYNQ-ERIKLKLKGLSPVEY"
                     /note="IS3_IS51_ORF1     e-value= 5e-45 fragment hit
                     coverage= 79.80%, length is 234aa with 12 gaps, 0 stops,
                     frame= Plus3"
     CDS             2334723..2335250
                     /colour="255 0 0"
                     /evidence=predicted
                     /translation="YGYRTVTLELANQGHRTNHKRVQRLMGEMGLKSRVRVKRYRSFK
                     G-AANVVVGNDLNRQFHAERPNQKWVTDVTEFKVQGMKLYLSPIMDLYNGEIVAYQIK
                     RQPVFDLVGQMLEEAIMKLSPD-----ERPMIHSDQGWQYQHENYRHMLEKHSLKQSM
                     SRRGNCLDNAAMESFFGTLKSE"
                     /note="IS3_IS51_ORF2     e-value= 7.8e-32 fragment hit
                     coverage= 57.41%, length is 176aa with 6 gaps, 0 stops,
                     frame= Plus3"
     CDS             2335227..2335334
                     /colour="255 0 0"
                     /evidence=predicted
                     /translation="FFGTLKSEFFYLNS---FDSIESLEAGLVEYIQYYNQER"
                     /note="IS481 e-value= 3.8e-07 fragment hit coverage=
```

11.11%, length is 36aa with 3 gaps, 0 stops, frame= Plus3"

CDS 4666605..4666814
/colour="255 0 0"
/evidence=predicted
/translation="RLTFGDAQGLGKRKQTRREILLYEVDXVVPWKALPARIEPHYP-
RSGRVGRSPYAMETMLRIHLLQQXYAF"
/note="IS5_IS5   e-value= 2.4e-24 fragment hit coverage= 16.82%, length is 70aa with 1 gaps, 2 stops, frame= Plus3"

CDS 4666605..4667441
/colour="255 0 0"
/evidence=predicted
/translation="RLTFGDAQGLGKRKQTRREILLYEVDXVVPWKALPARIEPHYP-
RSGRVGRSPYAMETMLRIHLLQQXYAFEQFGHVASVARdxfdaavRTAHQSFSGQDAL
HN--------SRGLHAKRREESXRLLWSincriETNTDLASLQESAAA-------AAA
AQAASLQSN---
--HF--------
DACSSRIRSRLTSTRLAITMTTAP------------------MNKNGKVRPSAVAATI
ARQHTQ--------MAKVFSLIPWILTNFLMPATSGNIFF--QXGHEGSPPR-----R
GAMVALLLRXVEERNASVAASFLLV-IQSLLRKRHSRHPVRR"
/note="IS5_IS5   e-value= 2.5e-06 complete sequence hit coverage= 100%, length is 279aa with 155 gaps, 5 stops, frame= Plus3"

CDS complement(3988994..3988837)
/colour="255 0 0"
/evidence=predicted
/translation="PEPTRTIFRLNRLQGLTQAEIARRLGVSVTTVENHVRTA-LQRL
AWARSG-------RXSP"
/note="IS21_ORF1   e-value= 5e-06 fragment hit coverage= 11.62%, length is 53aa with 8 gaps, 1 stops, frame= Minus1"

CDS complement(2520995..2519887)
/colour="255 0 0"
/evidence=predicted
/translation="VQDRGGPT------DRXVRPSGSGGCRATgRVDPXSLRLEAATR
QRRCWPACRAGPERGS-APPQGXATQGHXRARHPKKSRRVL---CKGVRAKYAFMKQH
ADEFGLAAMCRMLGVHRSGYYAWLKEPASARDKDDQRLLGLIKHSWLESGSVYGHRKV
TTDLR---ELGETCSRHRVARLMKSEGLRAMVGYGRRPRPLSGPVGSVAKNVLARGFK
VSEPNRAWVTDITYIRTY-DGFLYLAVVLDLFSRQVVGWATRPTQHTDLVLQALLAAV
WRRKPSPGLLLHS-----DQGTQFTSEDWQSFLREHDIVCSMSRRGNCHDNAAMESFF
QLLKRERIK--RRIYSNHDEARADVFQYIEmFYNPKRRHSSNDGLSPVEFEKQYALN"
/note="IS3_IS150_ORF2   e-value= 1.2e-45 complete sequence hit coverage= 100%, length is 370aa with 21 gaps, 4 stops, frame= Minus1"

CDS complement(2520797..2519854)
/colour="255 0 0"
/evidence=predicted
/translation="RARHPKKSRRVLCKGVRAKYAFMKQHADEFGLAAMC--RMLGVH
RS-GYYAWLKE---PASARDKDDQRLLGLIKHsWLESGSVYGHRKVTTDLRELGET--
-CSRHRVARLMKSEGLRAMVGYGRRPRPLSGPVGSVAKNvLARGFKVSEPNRAWVTDI
TYI---------------RT------YDGFLYLAVVLDLFSRqVVGWATRPTQHTDLV
LQALLAAVWRRKPSPGL-L--LHSDQGTQFTSE----DWQSFLREHDIVCSMSRRGNC
HDNAAMESFFQLLKRERIKRRI---YSNHDEARADVFQYIEMfYNPKRRHSSNDGLSP
VEFEKQYALNGXRLSRKPWAI"
/note="IS481 e-value= 4.4e-07 complete sequence hit coverage= 100%, length is 315aa with 40 gaps, 1 stops, frame= Minus1"

CDS complement(2520794..2519890)
/colour="255 0 0"

/evidence=predicted
/translation="ARHPKKSRRVLCKgVRAKYAFMKQHADEFGLAAMCRMLGVHRSg
YYAWLKePASARDKDDQRLLGLIKHSWLESGSVYGHRKVTTDLRELGETCSRHRVARL
MKSEGLRAMVGYGR--RPRPLSGPVGSVAKNVLARGFKVSEPNRAWVTDITYIRT--Y
DGFLYLAVVLDLFSRQVVGWATRPTQHTDLVLQALLAAVWR-RKPSPGLLLHSDQGTQ
FTSEDWQSFLREHDIVCSMSRRGNCHDNAAMESFFQLLKRERIKRRIYSNHD--EARA
DVFQYIEMFYNPKRRHSSNDGLSPVEFEKQYAL"
/note="IS3_IS150_ORF1   e-value= 1.4e-57 complete sequence
hit coverage= 100%, length is 302aa with 7 gaps, 0 stops,
frame= Minus1"

CDS complement(2520770..2519878)
/colour="255 0 0"
/evidence=predicted
/translation="RVLCKGVRAKYAFMkqhADEFGLAAMCRMLGVHRSGYYAWLK--
----EPASARDKDDQRLLGLIKHSWLESGSvYGHRKVTTDLRELGETCSR-----HRV
ARLMkseGLRAMVgYGRRPRPLSGP---VGSVAknvlargfkVSEPNRAWVTDITYIR
TYDG-FLYLAVVLDLFSRQVVGWATRPTQ--HTDLVLQALLAAVWRR----KPSPGLL
LH-SDQGTQFTSEDWQSFLREHDIVCSMSRRGNCHDNAAMESFFQLLKRERIkrRIYS
NHDEARA--DVFQYIEMfYNPKRRHSSNDGLSPVEFEKQYALNGXR"
/note="IS3_IS2_ORF2   e-value= 1.4e-07 complete sequence
hit coverage= 100%, length is 298aa with 24 gaps, 1
stops, frame= Minus1"

CDS complement(2520767..2519890)
/colour="255 0 0"
/evidence=predicted
/translation="VLCKgVRAKYAFMKQHADEFGLAAMCRMLGVHRSgYYAWLKePA
SARDKDDQRLLGLIKHSWLESGSVYGHRKVTTDLRELGETCSRHRVARLMKSEGLRAM
VGYGR--RPRPLSGPVGSVAKNVLARGFKVSEPNRAWVTDITYIRT--YDGFLYLAVV
LDLFSRQVVGWATRPTQHTDLVLQALLAAVWR-RKPSPGLLLHSDQGTQFTSEDWQSF
LREHDIVCSMSRRGNCHDNAAMESFFQLLKRERIKRRIYSNHD--EARADVFQYIEMF
YNPKRRHSSNDGLSPVEFEKQYAL"
/note="IS3_IS150_ORF1   e-value= 5.1e-57 fragment hit
coverage= 97.06%, length is 293aa with 7 gaps, 0 stops,
frame= Minus1"

CDS complement(2520758..2519842)
/colour="255 0 0"
/evidence=predicted
/translation="KGVRAKYAFMKQHAD--EFGLAAMCRMLGVH---RSGYYAWLKE
PASARDKDDQRLLGLIKHSWLESGS-----VYGHRKVTTDLRELGETCSRHRVARLMK
SEGLRAMVGYGRRPRPLSGPVGSVAKNVLARGFKVSEPNRAWVTDITYIRTYDGFLYL
AVVLDLFSRQVVGWATRPTQHTDLVLQALLAAVWRRKPS-----PGLLLHSDQGTQFT
SEDWQSFLREHDIVCSMSRRGNCHDNAAMESFFQLLKRERIKRRIYSNHDEARADVFQ
YIEMFYNPKRRHSSNDGLSPVEFEKQYALNGxrlsRKPWAIQTDN"
/note="IS3_IS51_ORF2   e-value= 4.4e-135 complete
sequence
hit coverage= 100%, length is 306aa with 15 gaps, 0
stops, frame= Minus1"

CDS complement(2520740..2519896)
/colour="255 0 0"
/evidence=predicted
/translation="YAFMKQHADEFGLAAMCRMLGVHRSGYYAWL-----KEPASARD
KDDQRLLGLIKHSWLESGSVYGHRKVTTDLRELGETCSRHRVARLMKSEGLRAMVGYG
RRPRPLSGPVGSVAKNVLARGFKVSEPNRAWVTDITYIRTYDGFLYLAVVLDLFSRQV
VGWATRPTQHTDLV-LQALLAAVWRRKPSP----GLLLHSDQGTQFTSEDWQSFLREH
DIVCSMSRRGNCHDNAAMESFFQLLKRERIKR----RIYSNHDEARADVFQYIEMFYN
PKRRHSSNDGLSPVEFEKQY"
/note="IS3_IS51_ORF1   e-value= 3.9e-110 fragment hit
coverage= 96.42%, length is 282aa with 14 gaps, 0 stops,
frame= Minus1"

CDS complement(2520740..2519863)

```
                     /colour="255 0 0"
                     /evidence=predicted
                     /translation="YAFMKQHADEFGLAAMCRMLGVHRSGYYAWL-----KEPASARD
                     KDDQRLLGLIKHSWLESGSVYGHRKVTTDLRELGETCSRHRVARLMKSEGLRAMVGYG
                     RRPRPLSGPVGSVAKNVLARGFKVSEPNRAWVTDITYIRTYDGFLYLAVVLDLFSRQV
                     VGWATRPTQHTDLV-LQALLAAVWRRKPSP----GLLLHSDQGTQFTSEDWQSFLREH
                     DIVCSMSRRGNCHDNAAMESFFQLLKRERIKR----RIYSNHDEARADVFQYIEMFYN
                     PKRRHSSNDGLSPVEFEKQYALNGXRLSRKP"
                     /note="IS3_IS51_ORF1     e-value= 3.7e-108 complete
sequence
                     hit coverage= 100%, length is 293aa with 14 gaps, 1
                     stops, frame= Minus1"
     CDS             complement(2520737..2519893)
                     /colour="255 0 0"
                     /evidence=predicted
                     /translation="AFMKQHADEFGLAAMCRMLGVHRSGYYAWLKE--PASARDKDDQ
                     RLLGLIKHSWLESGSVYGHRKVTTDLR-ELGETCSRHRVARLMKSEGLRAmVGYG-RR
                     PRPLSgPVGSVAKNVLARGFKVSEPNRAWVTDITYIRTYDGFLYLAVVLDLFSRQVVG
                     WATRPTQHTDLVLQALLAAVWRRKPSPGLLLHSDQGTQFTSEDWQSFLREHDIVCSMS
                     RRGNCHDNAAMESFFQLLK--RERIKRRIYSNHDEARADVFQYIEMFYNPKRRHSSND
                     GLSPVEFEKQYA"
                     /note="IS3_IS3_ORF1      e-value= 1.9e-94 complete sequence
                     hit coverage= 100%, length is 282aa with 6 gaps, 0 stops,
                     frame= Minus1"
     CDS             complement(2520737..2519893)
                     /colour="255 0 0"
                     /evidence=predicted
                     /translation="AFMKQHADEFGLAAMCRMLGVHRSGYYAWLKE--PASARDKDDQ
                     RLLGLIKHSWLESGSVYGHRKVTTDLR-ELGETCSRHRVARLMKSEGLRAmVGYG-RR
                     PRPLSgPVGSVAKNVLARGFKVSEPNRAWVTDITYIRTYDGFLYLAVVLDLFSRQVVG
                     WATRPTQHTDLVLQALLAAVWRRKPSPGLLLHSDQGTQFTSEDWQSFLREHDIVCSMS
                     RRGNCHDNAAMESFFQLLK--RERIKRRIYSNHDEARADVFQYIEMFYNPKRRHSSND
                     GLSPVEFEKQYA"
                     /note="IS3_IS3_ORF1      e-value= 4.9e-95 complete sequence
                     hit coverage= 100%, length is 282aa with 6 gaps, 0 stops,
                     frame= Minus1"
     CDS             complement(2520725..2519881)
                     /colour="255 0 0"
                     /evidence=predicted
                     /translation="QHADEFGLAAMCRMLGVHRSGYYAWLKEPASARDKDDQRLLGLI
                     KHSWLESGSVYGHRKVTTDLRELGETCSRHRVARLMKSEGLRAMvGYGRRPRPLSGPV
                     GSVAK-NVLARGFKVSE--PNRAWVTDITYIRTYDGFLYLAVVLDLFSRQVVGWATRP
                     TQHTDLVLQALLAAVWR----RKPSPGLLLHSDQGTQFTSEDWQSFLREHDIVCSMSR
                     RGNCHDNAAMESFFQLLKRERIKRRIYSNHDEARADVFQYIEMFYNPKRRHSSNDGLS
                     PVEFEKQYALNGX"
                     /note="IS3_IS3_ORF2      e-value= 7.1e-102 complete
sequence
                     hit coverage= 100%, length is 282aa with 7 gaps, 1 stops,
                     frame= Minus1"
     CDS             complement(2520725..2519881)
                     /colour="255 0 0"
                     /evidence=predicted
                     /translation="QHADEFGLAAMCRMLGVHRSGYYAWLKEPASARDKDDQRLLGLI
                     KHSWLESGSVYGHRKVTTDLRELGETCSRHRVARLMKSEGLRAMvGYGRRPRPLSGPV
                     GSVAK-NVLARGFKVSE--PNRAWVTDITYIRTYDGFLYLAVVLDLFSRQVVGWATRP
                     TQHTDLVLQALLAAVWR----RKPSPGLLLHSDQGTQFTSEDWQSFLREHDIVCSMSR
                     RGNCHDNAAMESFFQLLKRERIKRRIYSNHDEARADVFQYIEMFYNPKRRHSSNDGLS
                     PVEFEKQYALNGX"
                     /note="IS3_IS3_ORF2      e-value= 2e-102 complete sequence
hit
```

```
                     coverage= 100%, length is 282aa with 7 gaps, 1 stops,
                     frame= Minus1"
     CDS             complement(2520713..2519887)
                     /colour="255 0 0"
                     /evidence=predicted
                     /translation="EFGLAAMCRMLGVHRSGYYAWLKEPASARDKDDQRLLGLIKHSW
                     LESGSVYGHRKVTTDLR---ELGETCSRHRVARLMKSEGLRAMVGYGRRPRPLSGPVG
                     SVAKNVLARGFKVSEPNRAWVTDITYIRTY-DGFLYLAVVLDLFSRQVVGWATRPTQH
                     TDLVLQALLAAVWRRKPSPGLLLHS-----DQGTQFTSEDWQSFLREHDIVCSMSRRG
                     NCHDNAAMESFFQLLKRERIK--RRIYSNHDEARADVFQYIEmFYNPKRRHSSNDGLS
                     PVEFEKQYALN"
                     /note="IS3_IS150_ORF2    e-value= 1.9e-74 fragment hit
                     coverage= 73.52%, length is 276aa with 11 gaps, 0 stops,
                     frame= Minus1"
     CDS             complement(2520710..2519893)
                     /colour="255 0 0"
                     /evidence=predicted
                     /translation="FGLAAMCRMLGVH---RSGYYAWLKEPASARDKDDQRLLGLIKH
                     SWLESGS-----VYGHRKVTTDLRELGETCSRHRVARLMKSEGLRAMVGYGRRPRPLS
                     GPVGSVAKNVLARGFKVSEPNRAWVTDITYIRTYDGFLYLAVVLDLFSRQVVGWATRP
                     TQHTDLVLQALLAAVWRRKPS-----PGLLLHSDQGTQFTSEDWQSFLREHDIVCSMS
                     RRGNCHDNAAMESFFQLLKRERIKRRIYSNHDEARADVFQYIEMFYNPKRRHSSNDGL
                     SPVEFEKQYA"
                     /note="IS3_IS51_ORF2     e-value= 7.1e-152 fragment hit
                     coverage= 90.22%, length is 273aa with 13 gaps, 0 stops,
                     frame= Minus1"
     CDS             complement(2443193..2442061)
                     /colour="255 0 0"
                     /evidence=predicted
                     /translation="LCTGARRVAXPGFPFHCASNPSSSRGQGDLPLIVSLHRHAPDK-
                     ---AHTEGX-KRVRLAIPETYRTRAVR---QEGLVRSSHTKIRIPKksrgvlrqgMTA
                     KYAFMREHLGEFRLTAMCRVLGVNRSGYYAWAGNS--GRPRRREDdrLRGLIKHAWLA
                     SGTVYGYRKITRELR---EAGERCSRHRVRRLMKAEGIRAEIGYGTKPRHRGGPPGMV
                     -ENVVNRDFSPAAPNKVWVTDITYIRTY-EGWTFLAVIVDLYSRQVVGWAMQSQMTTD
                     LVLQALVSAVWKRKPAAGLIIHS-----DQGSQFTSSDWLSLLKQNGMVPSMSRRGNC
                     HDNAVAESFFSALKKERIK--RRIYPTRDEARSDVFNYIEmFYNPIRRHGSAGNLAPV
                     EF-ERRYAQ"
                     /note="IS3_IS150_ORF2    e-value= 1.2e-42 complete sequence
                     hit coverage= 100%, length is 378aa with 23 gaps, 2
                     stops, frame= Minus1"
     CDS             complement(2442968..2442064)
                     /colour="255 0 0"
                     /evidence=predicted
                     /translation="TKIRIPKKSrgVLRQgMTAKYAFMREHLGEFRLTAMCRVLGVNR
                     SgYYAWAGNSGRPRRREDDRLRGLIKHAWlASGTVYGYRKITRELREAGERCSRHRVR
                     RLMKAEGIRAEIGYGT---KPRHRGGPPGMVENVVNRDFSPAAPNKVWVTDITYIRT-
                     -YEGWTFLAVIVDLYSRQVVGWAMQSQMTTDLVLQALVSAVWK-RKPAAGLIIHSDQG
                     SQFTSSDWLSLLKQNGMVPSMSRRGNCHDNAVAESFFSALKKERIKRRIYPTRD--EA
                     RSDVFNYIEMFYNPIRRHGSAGNLAPVEF-ERRYA"
                     /note="IS3_IS150_ORF1    e-value= 2.9e-46 complete sequence
                     hit coverage= 100%, length is 302aa with 9 gaps, 0 stops,
                     frame= Minus1"
     CDS             complement(2442926..2442025)
                     /colour="255 0 0"
                     /evidence=predicted
                     /translation="QGMTAKYAFMREHLGEFR--LTAMCRVLGVN---RSGYYAWAGN
                     SGRPRRREDDRLRGLIKHAWLASGT-----VYGYRKITRELREAGERCSRHRVRRLMK
                     AEGIRAEIGYGTKPRHRGGPPGMV-ENVVNRDFSPAAPNKVWVTDITYIRTYEGWTFL
                     AVIVDLYSRQVVGWAMQSQMTTDLVLQALVSAVWKRKPA-----AGLIIHSDQGSQFT
                     SSDWLSLLKQNGMVPSMSRRGNCHDNAVAESFFSALKKERIKRRIYPTRDEARSDVFN
```

YIEMFYNPIRRHGSAGNLAPVEFERRYAQGGSXMSTEVWPV"
/note="IS3_IS51_ORF2   e-value= 9.2e-135 complete
sequence
hit coverage= 100%, length is 301aa with 16 gaps, 1
stops, frame= Minus1"
CDS complement(2442908..2442037)
/colour="255 0 0"
/evidence=predicted
/translation="YAFMREHLGEFRLTAMCRVLGVNRSGYYAWA---GNSGRPRRRE
--DDRLRGLIKHAWLASGTVYGYRKITRELREAGERCSRHRVRRLMKAEGIRAEIGYG
TKPRHRGGPPGMVE-NVVNRDFSPAAPNKVWVTDITYIRTYEGWTFLAVIVDLYSRQV
VGWAMQSQMTTDLV-LQALVSAVWKRKPAA----GLIIHSDQGSQFTSSDWLSLLKQN
GMVPSMSRRGNCHDNAVAESFFSALKKERIKR----RIYPTRDEARSDVFNYIEMFYN
PIRRHGSAGNLAPVEFERRY-AQGGSXMSTE"
/note="IS3_IS51_ORF1   e-value= 1.2e-109 complete
sequence
hit coverage= 100%, length is 291aa with 16 gaps, 1
stops, frame= Minus1"
CDS complement(2442908..2442037)
/colour="255 0 0"
/evidence=predicted
/translation="YAFMREHLGEFRLTAMCRVLGVNRSGYYAWA---GNSGRPRRRE
--DDRLRGLIKHAWLASGTVYGYRKITRELREAGERCSRHRVRRLMKAEGIRAEIGYG
TKPRHRGGPPGMVE-NVVNRDFSPAAPNKVWVTDITYIRTYEGWTFLAVIVDLYSRQV
VGWAMQSQMTTDLV-LQALVSAVWKRKPAA----GLIIHSDQGSQFTSSDWLSLLKQN
GMVPSMSRRGNCHDNAVAESFFSALKKERIKR----RIYPTRDEARSDVFNYIEMFYN
PIRRHGSAGNLAPVEFERRY-AQGGSXMSTE"
/note="IS3_IS51_ORF1   e-value= 1.3e-108 complete
sequence
hit coverage= 100%, length is 291aa with 16 gaps, 1
stops, frame= Minus1"
CDS complement(2442905..2442064)
/colour="255 0 0"
/evidence=predicted
/translation="AFMREHLGEFRLTAMCRVLGVNRSGYYAWAGN--SGRPRRREDD
RLRGLIKHAWLASGTVYGYRKITRELR-EAGERCSRHRVRRLMKAEGIRAeIGYG---
-TKPRHRGgPpgMVENVVNRDFSPAAPNKVWVTDITYIRTYEGWTFLAVIVDLYSRQV
VGWAMQSQMTTDLVLQALVSAVWKRKPAAGLIIHSDQGSQFTSSDWLSLLKQNGMVPS
MSRRGNCHDNAVAESFFSALK--KERIKRRIYPTRDEARSDVFNYIEMFYNPIRRHGS
AGNLAPVEFERRYA"
/note="IS3_IS3_ORF1   e-value= 1e-93 complete sequence
hit
coverage= 100%, length is 281aa with 9 gaps, 0 stops,
frame= Minus1"
CDS complement(2442905..2442064)
/colour="255 0 0"
/evidence=predicted
/translation="AFMREHLGEFRLTAMCRVLGVNRSGYYAWAGN--SGRPRRREDD
RLRGLIKHAWLASGTVYGYRKITRELR-EAGERCSRHRVRRLMKAEGIRAeIGYG---
-TKPRHRGgPpgMVENVVNRDFSPAAPNKVWVTDITYIRTYEGWTFLAVIVDLYSRQV
VGWAMQSQMTTDLVLQALVSAVWKRKPAAGLIIHSDQGSQFTSSDWLSLLKQNGMVPS
MSRRGNCHDNAVAESFFSALK--KERIKRRIYPTRDEARSDVFNYIEMFYNPIRRHGS
AGNLAPVEFERRYA"
/note="IS3_IS3_ORF1   e-value= 2.7e-94 complete sequence
hit coverage= 100%, length is 281aa with 9 gaps, 0 stops,
frame= Minus1"
CDS complement(2442893..2442052)
/colour="255 0 0"
/evidence=predicted
/translation="EHLGEFRLTAMCRVLGVNRSGYYAWAGNSGRPRRREDDRLRGLI

```
                     KHAWLASGTVYGYRKITRELREAGERCSRHRVRRLMKAEGIRAEIgYGTKPRHRGG--
                     PPGMVENVVNRDFSPAA--PNKVWVTDITYIRTYEGWTFLAVIVDLYSRQVVGWAMQS
                     QMTTDLVLQALVSAVWK----RKPAAGLIIHSDQGSQFTSSDWLSLLKQNGMVPSMSR
                     RGNCHDNAVAESFFSALKKERIKRRIYPTRDEARSDVFNYIEMFYNPIRRHGSAGNLA
                     PVEFERRYAQGGS"
                     /note="IS3_IS3_ORF2      e-value= 2.8e-107 complete
sequence
                     hit coverage= 100%, length is 281aa with 8 gaps, 0 stops,
                     frame= Minus1"
     CDS             complement(2442893..2442052)
                     /colour="255 0 0"
                     /evidence=predicted
                     /translation="EHLGEFRLTAMCRVLGVNRSGYYAWAGNSGRPRRREDDRLRGLI
                     KHAWLASGTVYGYRKITRELREAGERCSRHRVRRLMKAEGIRAEIgYGTKPRHRGG--
                     PPGMVENVVNRDFSPAA--PNKVWVTDITYIRTYEGWTFLAVIVDLYSRQVVGWAMQS
                     QMTTDLVLQALVSAVWK----RKPAAGLIIHSDQGSQFTSSDWLSLLKQNGMVPSMSR
                     RGNCHDNAVAESFFSALKKERIKRRIYPTRDEARSDVFNYIEMFYNPIRRHGSAGNLA
                     PVEFERRYAQGGS"
                     /note="IS3_IS3_ORF2      e-value= 7.8e-108 complete
sequence
                     hit coverage= 100%, length is 281aa with 8 gaps, 0 stops,
                     frame= Minus1"
     CDS             complement(2442884..2442061)
                     /colour="255 0 0"
                     /evidence=predicted
                     /translation="GEFRLTAMCRVLGVNRSGYYAWAGNS--GRPRRREDdrLRGLIK
                     HAWLASGTVYGYRKITRELR---EAGERCSRHRVRRLMKAEGIRAEIGYGTKPRHRGG
                     PPGMV-ENVVNRDFSPAAPNKVWVTDITYIRTY-EGWTFLAVIVDLYSRQVVGWAMQS
                     QMTTDLVLQALVSAVWKRKPAAGLIIHS-----DQGSQFTSSDWLSLLKQNGMVPSMS
                     RRGNCHDNAVAESFFSALKKERIK--RRIYPTRDEARSDVFNYIEmFYNPIRRHGSAG
                     NLAPVEF-ERRYAQ"
                     /note="IS3_IS150_ORF2    e-value= 3.9e-68 fragment hit
                     coverage= 73.78%, length is 275aa with 15 gaps, 0 stops,
                     frame= Minus1"
     CDS             complement(2442866..2442052)
                     /colour="255 0 0"
                     /evidence=predicted
                     /translation="AMCRVLGVN---RSGYYAWAGNSGRPRRREDDRLRGLIKHAWLA
                     SGT-----VYGYRKITRELREAGERCSRHRVRRLMKAEGIRAEIGYGTKPRHRGGPPG
                     MV-ENVVNRDFSPAAPNKVWVTDITYIRTYEGWTFLAVIVDLYSRQVVGWAMQSQMTT
                     DLVLQALVSAVWKRKPA-----AGLIIHSDQGSQFTSSDWLSLLKQNGMVPSMSRRGN
                     CHDNAVAESFFSALKKERIKRRIYPTRDEARSDVFNYIEMFYNPIRRHGSAGNLAPVE
                     FERRYAQGGS"
                     /note="IS3_IS51_ORF2     e-value= 7.1e-151 fragment hit
                     coverage= 90.22%, length is 272aa with 14 gaps, 0 stops,
                     frame= Minus1"
     CDS             complement(2442734..2442064)
                     /colour="255 0 0"
                     /evidence=predicted
                     /translation="VYGYRKITRELREAGERCSRHRVRRLMKAEGIRAEIGYGT---K
                     PRHRGGPPGMVENVVNRDFSPAAPNKVWVTDITYIRT--YEGWTFLAVIVDLYSRQVV
                     GWAMQSQMTTDLVLQALVSAVWK-RKPAAGLIIHSDQGSQFTSSDWLSLLKQNGMVPS
                     MSRRGNCHDNAVAESFFSALKKERIKRRIYPTRD--EARSDVFNYIEMFYNPIRRHGS
                     AGNLAPVEF-ERRYA"
                     /note="IS3_IS150_ORF1    e-value= 2.3e-51 fragment hit
                     coverage= 76.14%, length is 224aa with 9 gaps, 0 stops,
                     frame= Minus1"
     CDS             complement(2395079..2394205)
                     /colour="255 0 0"
                     /evidence=predicted
```

```
                /translation="FGIGIDVSKATLDVAVHGEQ------FRQFSNDKRGFVRLIRWL
                KTWP-----IKQVVLEASGGYERAALDVLHAA---GLPMVRINPARARRFAQGT-GRA
                AKTDRLDATVLAQMAHLLPLTRYVP--PAPW---QQRLAEFAQCRRQLVQMRVSEMQR
                LRRLVDPDLIA------------------------------------------------
                ----------------------------MKQRHIAQLSDDLKQLDKAIAEQLEGQPGW
                KNIGA------LKGVGPILISTLACELP-ELGSLGSK-AIASLVGLAPMNRESG-TWQ
                GQRRISG-GRAVVREALYMAALTAIRYEPRLRAFYAGLKAKGK---ASKVALVAVMRK
                MLVILNAR"
                /note="IS110  e-value= 1.4e-51 fragment hit coverage=
                96.15%, length is 292aa with 108 gaps, 0 stops, frame=
                Minus1"
CDS             complement(2395079..2394142)
                /colour="255 0 0"
                /evidence=predicted
                /translation="FGIGIDVSKATLDVAVHGEQ------FRQFSNDKRGFVRLIRWL
                KTWP-----IKQVVLEASGGYERAALDVLHAA---GLPMVRINPARARRFAQGT-GRA
                AKTDRLDATVLAQMAHLLPLTRYVP--PAPW---QQRLAEFAQCRRQLVQMRVSEMQR
                LRRLVDPDLIA------------------------------------------------
                ----------------------------MKQRHIAQLSDDLKQLDKAIAEQLEGQPGW
                KNIGA------LKGVGPILISTLACELP-ELGSLGSK-AIASLVGLAPMNRESG-TWQ
                GQRRISG-GRAVVREALYMAALTAIRYEPRLRAFYAGLKAKGK---ASKVALVAVMRK
                MLVILNARkrdaeVALGCPXQDSCYXPPP"
                /note="IS110  e-value= 6.5e-50 complete sequence hit
                coverage= 100%, length is 313aa with 108 gaps, 2 stops,
                frame= Minus1"
CDS             complement(1723697..1723555)
                /colour="255 0 0"
                /evidence=predicted
                /translation="KRYTDEFKIEAVRQIVEYGRPVAEVAERLGVSIHSLYGWKRQQG
                KGDV"
                /note="IS3_IS407_ORF1    e-value= 7.7e-06 fragment hit
                coverage= 55.81%, length is 48aa with 0 gaps, 0 stops,
                frame= Minus1"
CDS             complement(1723544..1723417)
                /colour="255 0 0"
                /evidence=predicted
                /translation="VEQDQNAEVRRLKAELRRVTEERDILK--KAAAYFAKGXEQSTP
                S"
                /note="IS3_IS51_ORF1     e-value= 3.2e-08 fragment hit
                coverage= 14.66%, length is 43aa with 2 gaps, 1 stops,
                frame= Minus1"
CDS             complement(877250..876166)
                /colour="255 0 0"
                /evidence=predicted
                /translation="MAAAVCSRrcpSVGTASAAAPKADEKDpFVQAA--XGNEPRXAA
                QGSRLSACGDRlPKKtRCLDPGRAGGARRKaqAIQGLRQVHTLSLL--LEAAELSRST
                FYYQnhvlAHPDQDEAALCERIRAIYdQSKGRYGYRTVTLELANQGHRTNHKRVQRLM
                GEMGLKSRVRV-KRYRSFKGAANVVVgndlnrqfhAERPNQKW----VTDVTefkVQG
                MKLYLSPIMDLYNGEIVAYQIKRQPVFDLVGQMLEEAIKK-LSPDerpMIHSDQGWQY
                QHENYRHMLEKHSLKQSMSRRGNCLDNAAMESFFGTLKSEF--FYLnsFDNIESLEAG
                LVEYIQYYNQERIKLKLKGLSPVEYRERAQSAAXPRPSLG"
                /note="IS3_IS407_ORF2    e-value= 1.2e-08 complete sequence
                hit coverage= 100%, length is 362aa with 12 gaps, 3
                stops, frame= Minus1"
CDS             complement(877244..876184)
                /colour="255 0 0"
                /evidence=predicted
                /translation="AAVCSRRCPS-------VGTASAAAPKAD---EKDPFVQAAXGN
                EPRXAAQGS-----RLSACGD---RLPKKTRCLDPG------RAGGARRKAQAIQGLR
                QVHTLSLLLEAAELSRSTFYYQNHVL--AHPDqDEAALCERIRAIYDQSKGRYGYRTV
```

```
                     TLELA---NQGHRTNHKRVQRLMGEMGLKSRVRVK-RYRSFKGAANVVVGNDLNRQFH
                     AERPNQKWVTDVTEFKVQ-GMKLYLSPIMDLYNGEIVAYQIKRQPVFDLVGQMLEEAI
                     KKLSPDERPMIHS-----DQGWQYQHENYRHMLEKHSLKQSMSRRGNCLDNAAMESFF
                     GTLKSEFFY--LNSFDNIESLEAGLVEYIQYYNQERIKLKLKGLSPVEYreRAQSAAX
                     "
                     /note="IS3_IS150_ORF2    e-value= 1.4e-122 complete
sequence
                     hit coverage= 100%, length is 354aa with 38 gaps, 3
                     stops, frame= Minus1"
     CDS             complement(877091..876193)
                     /colour="255 0 0"
                     /evidence=predicted
                     /translation="LPKKTRCLDPGRAggARRKAQAIQGLRQVHTLSLLLEAAELSRS
                     TFYYQNHVLAHPDqDEAALCERIRAIYDQSKGRYGYRTVTLELANQGHRTNHKRVQRL
                     MGEMGLKSRVRVK---RYRSFKGAANVVVGNDLNRQFHAERPNQKWVTDVTEFKV--Q
                     GMKLYLSPIMDLYNGEIVAYQIKRQPVFDLVGQMLEEAIKK-LSPDERPMIHSDQGWQ
                     YQHENYRHMLEKHSLKQSMSRRGNCLDNAAMESFFGTLKSEFFYLNSFDNIE--SLEA
                     GLVEYIQYYNQE-RIKLKLKGLSPVEYRERAQS"
                     /note="IS3_IS150_ORF1    e-value= 3.1e-88 complete sequence
                     hit coverage= 100%, length is 300aa with 9 gaps, 0 stops,
                     frame= Minus1"
     CDS             complement(877091..876193)
                     /colour="255 0 0"
                     /evidence=predicted
                     /translation="LPKKTRCLDPGRAggARRKAQAIQGLRQVHTLSLLLEAAELSRS
                     TFYYQNHVLAHPDqDEAALCERIRAIYDQSKGRYGYRTVTLELANQGHRTNHKRVQRL
                     MGEMGLKSRVRVK---RYRSFKGAANVVVGNDLNRQFHAERPNQKWVTDVTEFKV--Q
                     GMKLYLSPIMDLYNGEIVAYQIKRQPVFDLVGQMLEEAIKK-LSPDERPMIHSDQGWQ
                     YQHENYRHMLEKHSLKQSMSRRGNCLDNAAMESFFGTLKSEFFYLNSFDNIE--SLEA
                     GLVEYIQYYNQE-RIKLKLKGLSPVEYRERAQS"
                     /note="IS3_IS150_ORF1    e-value= 8e-89 complete sequence
                     hit coverage= 100%, length is 300aa with 9 gaps, 0 stops,
                     frame= Minus1"
     CDS             complement(877043..876184)
                     /colour="255 0 0"
                     /evidence=predicted
                     /translation="RRKAQAIQGLRQVHTLSLLLEAAELSRSTFYYQNHVL--AHPDq
                     DEAALCERIRAIYDQSKGRYGYRTVTLELA---NQGHRTNHKRVQRLMGEMGLKSRVR
                     VK-RYRSFKGAANVVVGNDLNRQFHAERPNQKWVTDVTEFKVQ-GMKLYLSPIMDLYN
                     GEIVAYQIKRQPVFDLVGQMLEEAIKKLSPDERPMIHS-----DQGWQYQHENYRHML
                     EKHSLKQSMSRRGNCLDNAAMESFFGTLKSEFFY--LNSFDNIESLEAGLVEYIQYYN
                     QERIKLKLKGLSPVEYreRAQSAAX"
                     /note="IS3_IS150_ORF2    e-value= 1.9e-152 fragment hit
                     coverage= 76.61%, length is 287aa with 14 gaps, 1 stops,
                     frame= Minus1"
     CDS             complement(877034..876163)
                     /colour="255 0 0"
                     /evidence=predicted
                     /translation="AQAIQGLRQVHTLSLLLEAAELSRSTFYYQNHVLAHPD------
                     QDEAALCERIRAIYDQSKGRYGYRTVTLELANQGHRTNHKRVQRLMGEMGLKSRVRVK
                     RYRSFKGAANVVVG-NDLNRQFHAERPNQKWVTDVTEFKV-QGmKLYLSPIMDLYNGE
                     IVAYQIKRQPVFDLV-GQMLEEAIKKLSPDE----RPMIHSDQGWQYQHENYRHMLEK
                     HSLKQSMSRRGNCLDNAAMESFFGTLKSEFFYL----NSFDNIESLEAGLVEYIQYYN
                     Q-ERIKLKLKGLSPVEYrERAQSAAXPRPSLGG"
                     /note="IS3_IS51_ORF1     e-value= 3.8e-39 complete sequence
                     hit coverage= 100%, length is 291aa with 18 gaps, 1
                     stops, frame= Minus1"
     CDS             complement(877034..876160)
                     /colour="255 0 0"
                     /evidence=predicted
```

```
                     /translation="---AQAIQGLR-QVHTLSLLLEA--AELSRS------TFYYQNH
                     VLAHPDQDEAALCERIRAIYDQSKG-----RYGYRTVTLELANQGHRTNHKRVQRLMG
                     EMGLKSRVRVKRYRSFKG-AANVVVGNDLNRQFHAERPNQKWVTDVTEFKVQGMKLYL
                     SPIMDLYNGEIVAYQIKRQPVFDLVGQMLEEAIKKLSPD-----ERPMIHSDQGWQYQ
                     HENYRHMLEKHSLKQSMSRRGNCLDNAAMESFFGTLKSEFFYLNSFDNIESLEAGLVE
                     YIQ-YYNQERIKLKLKGLSPVEYRER-AQSAAXPRPSLGGH"
                     /note="IS3_IS51_ORF2     e-value= 1.1e-16 complete sequence
                     hit coverage= 100%, length is 292aa with 25 gaps, 1
                     stops, frame= Minus1"
     CDS             complement(877031..876196)
                     /colour="255 0 0"
                     /evidence=predicted
                     /translation="QAIQGLRQVHTLSLLLEAAELSRSTFYYQNHVLAHPD---QDEA
                     ALCERIRAIYDQSKGRYGYRTVTLELA-NQGHRTNHKRVQRLMGEMGLKSRVRV-KRY
                     RSFKgAANVVVGNDLNRQFHAERPNQKWVTDVTEFKVQGMKLYLSPIMDLYNGEIVAY
                     QIKRQPVFDLVGQMLEEAIKKLSPDERPMIHSDQGWQYQHENYRHMLEKHSLKQSMSR
                     RGNCLDNAAMESFFGTLK--SEFFYLNSFDNIESLEAGLVEYIQ-YYNQERIKLKLKG
                     LSPVEYRERAQ"
                     /note="IS3_IS3_ORF1      e-value= 9.5e-76 complete sequence
                     hit coverage= 100%, length is 279aa with 8 gaps, 0 stops,
                     frame= Minus1"
     CDS             complement(877031..876196)
                     /colour="255 0 0"
                     /evidence=predicted
                     /translation="QAIQGLRQVHTLSLLLEAAELSRSTFYYQNHVLAHPD---QDEA
                     ALCERIRAIYDQSKGRYGYRTVTLELA-NQGHRTNHKRVQRLMGEMGLKSRVRV-KRY
                     RSFKgAANVVVGNDLNRQFHAERPNQKWVTDVTEFKVQGMKLYLSPIMDLYNGEIVAY
                     QIKRQPVFDLVGQMLEEAIKKLSPDERPMIHSDQGWQYQHENYRHMLEKHSLKQSMSR
                     RGNCLDNAAMESFFGTLK--SEFFYLNSFDNIESLEAGLVEYIQ-YYNQERIKLKLKG
                     LSPVEYRERAQ"
                     /note="IS3_IS3_ORF1      e-value= 2.5e-76 complete sequence
                     hit coverage= 100%, length is 279aa with 8 gaps, 0 stops,
                     frame= Minus1"
     CDS             complement(877019..876184)
                     /colour="255 0 0"
                     /evidence=predicted
                     /translation="GLRQVHTLSLLLEAAELSRSTFYYQNHV-LAHPDQDEAALCERI
                     RAIYDQSKGRYGYRTVTLELANQGHRTNHKRVQRLMGEMGLKSRVRVKRYRSFKGAAN
                     VVVG-NDLNRQFHAER--PNQKWVTDVTEFKVQGMKLYLSPIMDLYNGEIVAYQIKRQ
                     PVFDLVGQMLEEAIKK----LSPDERPMIHSDQGWQYQHENYRHMLEKHSLKQSMSRR
                     GNCLDNAAMESFFGTLKSEFFYLNSFDNIESLEAGLVEYIQYY-NQERIKLKLKGLSP
                     VEYRERAQSAAX"
                     /note="IS3_IS3_ORF2      e-value= 5.3e-64 complete sequence
                     hit coverage= 100%, length is 279aa with 9 gaps, 1 stops,
                     frame= Minus1"
     CDS             complement(877019..876184)
                     /colour="255 0 0"
                     /evidence=predicted
                     /translation="GLRQVHTLSLLLEAAELSRSTFYYQNHV-LAHPDQDEAALCERI
                     RAIYDQSKGRYGYRTVTLELANQGHRTNHKRVQRLMGEMGLKSRVRVKRYRSFKGAAN
                     VVVG-NDLNRQFHAER--PNQKWVTDVTEFKVQGMKLYLSPIMDLYNGEIVAYQIKRQ
                     PVFDLVGQMLEEAIKK----LSPDERPMIHSDQGWQYQHENYRHMLEKHSLKQSMSRR
                     GNCLDNAAMESFFGTLKSEFFYLNSFDNIESLEAGLVEYIQYY-NQERIKLKLKGLSP
                     VEYRERAQSAAX"
                     /note="IS3_IS3_ORF2      e-value= 1.5e-64 complete sequence
                     hit coverage= 100%, length is 279aa with 9 gaps, 1 stops,
                     frame= Minus1"
     CDS             complement(876911..876211)
                     /colour="255 0 0"
                     /evidence=predicted
```

```
                     /translation="AALCERIRAIYDQSKGRYGYRTVTLELANQGHRTNHKRVQRLMG
                     EMGLKSRVRVKRYRSFKGAANVVVG-NDLNRQFHAERPNQKWVTDVTEFKV-QGmKLY
                     LSPIMDLYNGEIVAYQIKRQPVFDLV-GQMLEEAIKKLSPDE----RPMIHSDQGWQY
                     QHENYRHMLEKHSLKQSMSRRGNCLDNAAMESFFGTLKSEFFYL----NSFDNIESLE
                     AGLVEYIQYYNQ-ERIKLKLKGLSPVEY"
                     /note="IS3_IS51_ORF1     e-value= 2.3e-45 fragment hit
                     coverage= 79.80%, length is 234aa with 12 gaps, 0 stops,
                     frame= Minus1"
     CDS             complement(876860..876334)
                     /colour="255 0 0"
                     /evidence=predicted
                     /translation="YGYRTVTLELANQGHRTNHKRVQRLMGEMGLKSRVRVKRYRSFK
                     G-AANVVVGNDLNRQFHAERPNQKWVTDVTEFKVQGMKLYLSPIMDLYNGEIVAYQIK
                     RQPVFDLVGQMLEEAIKKLSPD-----ERPMIHSDQGWQYQHENYRHMLEKHSLKQSM
                     SRRGNCLDNAAMESFFGTLKSE"
                     /note="IS3_IS51_ORF2     e-value= 5.2e-32 fragment hit
                     coverage= 57.41%, length is 176aa with 6 gaps, 0 stops,
                     frame= Minus1"
     CDS             complement(876356..876250)
                     /colour="255 0 0"
                     /evidence=predicted
                     /translation="FFGTLKSEFFYLNS---FDNIESLEAGLVEYIQYYNQER"
                     /note="IS481 e-value= 1.2e-06 fragment hit coverage=
                     11.11%, length is 36aa with 3 gaps, 0 stops, frame=
                     Minus1"
     CDS             complement(504125..503017)
                     /colour="255 0 0"
                     /evidence=predicted
                     /translation="VQDRGGPT------DRXVRPSGSGGCRATgRVDPXSLRLEAATR
                     QRRCWPACRAGPERGS-APPQGXATQGHXRARHPKKSRRVL---CKGVRAKYAFMKQH
                     ADEFGLAAMCRMLGVHRSGYYAWLKEPASARDKDDQRLLGLIKHSWLESGSVYGHRKV
                     TTDLR---ELGETCSRHRVARLMKSEGLRAMVGYGRRPRPLSGPVGSVAKNVLARGFK
                     VSEPNRAWVTDITYIRTY-DGFLYLAVVLDLFSRQVVGWATRPTQHTDLVLQALLAAV
                     WRRKPSPGLLLHS-----DQGTQFTSEDWQSFLREHDIVCSMSRRGNCHDNAAMESFF
                     QLLKRERIK--RRIYSNHDEARADVFQYIEmFYNPKRRHSSNDGLSPVEFEKQYALN"
                     /note="IS3_IS150_ORF2    e-value= 1.2e-45 complete sequence
                     hit coverage= 100%, length is 370aa with 21 gaps, 4
                     stops, frame= Minus1"
     CDS             complement(503924..503020)
                     /colour="255 0 0"
                     /evidence=predicted
                     /translation="ARHPKKSRRVLCKgVRAKYAFMKQHADEFGLAAMCRMLGVHRSg
                     YYAWLKePASARDKDDQRLLGLIKHSWLESGSVYGHRKVTTDLRELGETCSRHRVARL
                     MKSEGLRAMVGYGR--RPRPLSGPVGSVAKNVLARGFKVSEPNRAWVTDITYIRT--Y
                     DGFLYLAVVLDLFSRQVVGWATRPTQHTDLVLQALLAAVWR-RKPSPGLLLHSDQGTQ
                     FTSEDWQSFLREHDIVCSMSRRGNCHDNAAMESFFQLLKRERIKRRIYSNHD--EARA
                     DVFQYIEMFYNPKRRHSSNDGLSPVEFEKQYAL"
                     /note="IS3_IS150_ORF1    e-value= 1.4e-57 complete sequence
                     hit coverage= 100%, length is 302aa with 7 gaps, 0 stops,
                     frame= Minus1"
     CDS             complement(503897..503020)
                     /colour="255 0 0"
                     /evidence=predicted
                     /translation="VLCKgVRAKYAFMKQHADEFGLAAMCRMLGVHRSgYYAWLKePA
                     SARDKDDQRLLGLIKHSWLESGSVYGHRKVTTDLRELGETCSRHRVARLMKSEGLRAM
                     VGYGR--RPRPLSGPVGSVAKNVLARGFKVSEPNRAWVTDITYIRT--YDGFLYLAVV
                     LDLFSRQVVGWATRPTQHTDLVLQALLAAVWR-RKPSPGLLLHSDQGTQFTSEDWQSF
                     LREHDIVCSMSRRGNCHDNAAMESFFQLLKRERIKRRIYSNHD--EARADVFQYIEMF
                     YNPKRRHSSNDGLSPVEFEKQYAL"
                     /note="IS3_IS150_ORF1    e-value= 5.1e-57 fragment hit
```

```
                     coverage= 97.06%, length is 293aa with 7 gaps, 0 stops,
                     frame= Minus1"
     CDS             complement(503888..502981)
                     /colour="255 0 0"
                     /evidence=predicted
                     /translation="KGVRAKYAFMKQHAD--EFGLAAMCRMLGVH---RSGYYAWLKE
                     PASARDKDDQRLLGLIKHSWLESGS-----VYGHRKVTTDLRELGETCSRHRVARLMK
                     SEGLRAMVGYGRRPRPLSGPVGSVAKNVLARGFKVSEPNRAWVTDITYIRTYDGFLYL
                     AVVLDLFSRQVVGWATRPTQHTDLVLQALLAAVWRRKPS-----PGLLLHSDQGTQFT
                     SEDWQSFLREHDIVCSMSRRGNCHDNAAMESFFQLLKRERIKRRIYSNHDEARADVFQ
                     YIEMFYNPKRRHSSNDGLSPVEFEKQYALNGxRLSRKPWAIQ"
                     /note="IS3_IS51_ORF2     e-value= 1.4e-134 complete
sequence
                     hit coverage= 100%, length is 303aa with 15 gaps, 0
                     stops, frame= Minus1"
     CDS             complement(503870..503026)
                     /colour="255 0 0"
                     /evidence=predicted
                     /translation="YAFMKQHADEFGLAAMCRMLGVHRSGYYAWL-----KEPASARD
                     KDDQRLLGLIKHSWLESGSVYGHRKVTTDLRELGETCSRHRVARLMKSEGLRAMVGYG
                     RRPRPLSGPVGSVAKNVLARGFKVSEPNRAWVTDITYIRTYDGFLYLAVVLDLFSRQV
                     VGWATRPTQHTDLV-LQALLAAVWRRKPSP----GLLLHSDQGTQFTSEDWQSFLREH
                     DIVCSMSRRGNCHDNAAMESFFQLLKRERIKR----RIYSNHDEARADVFQYIEMFYN
                     PKRRHSSNDGLSPVEFEKQY"
                     /note="IS3_IS51_ORF1     e-value= 3.9e-110 fragment hit
                     coverage= 96.42%, length is 282aa with 14 gaps, 0 stops,
                     frame= Minus1"
     CDS             complement(503870..502993)
                     /colour="255 0 0"
                     /evidence=predicted
                     /translation="YAFMKQHADEFGLAAMCRMLGVHRSGYYAWL-----KEPASARD
                     KDDQRLLGLIKHSWLESGSVYGHRKVTTDLRELGETCSRHRVARLMKSEGLRAMVGYG
                     RRPRPLSGPVGSVAKNVLARGFKVSEPNRAWVTDITYIRTYDGFLYLAVVLDLFSRQV
                     VGWATRPTQHTDLV-LQALLAAVWRRKPSP----GLLLHSDQGTQFTSEDWQSFLREH
                     DIVCSMSRRGNCHDNAAMESFFQLLKRERIKR----RIYSNHDEARADVFQYIEMFYN
                     PKRRHSSNDGLSPVEFEKQYALNGXRLSRKP"
                     /note="IS3_IS51_ORF1     e-value= 3.7e-108 complete
sequence
                     hit coverage= 100%, length is 293aa with 14 gaps, 1
                     stops, frame= Minus1"
     CDS             complement(503867..503023)
                     /colour="255 0 0"
                     /evidence=predicted
                     /translation="AFMKQHADEFGLAAMCRMLGVHRSGYYAWLKE--PASARDKDDQ
                     RLLGLIKHSWLESGSVYGHRKVTTDLR-ELGETCSRHRVARLMKSEGLRAmVGYG-RR
                     PRPLSgPVGSVAKNVLARGFKVSEPNRAWVTDITYIRTYDGFLYLAVVLDLFSRQVVG
                     WATRPTQHTDLVLQALLAAVWRRKPSPGLLLHSDQGTQFTSEDWQSFLREHDIVCSMS
                     RRGNCHDNAAMESFFQLLK--RERIKRRIYSNHDEARADVFQYIEMFYNPKRRHSSND
                     GLSPVEFEKQYA"
                     /note="IS3_IS3_ORF1      e-value= 1.9e-94 complete sequence
                     hit coverage= 100%, length is 282aa with 6 gaps, 0 stops,
                     frame= Minus1"
     CDS             complement(503867..503023)
                     /colour="255 0 0"
                     /evidence=predicted
                     /translation="AFMKQHADEFGLAAMCRMLGVHRSGYYAWLKE--PASARDKDDQ
                     RLLGLIKHSWLESGSVYGHRKVTTDLR-ELGETCSRHRVARLMKSEGLRAmVGYG-RR
                     PRPLSgPVGSVAKNVLARGFKVSEPNRAWVTDITYIRTYDGFLYLAVVLDLFSRQVVG
                     WATRPTQHTDLVLQALLAAVWRRKPSPGLLLHSDQGTQFTSEDWQSFLREHDIVCSMS
                     RRGNCHDNAAMESFFQLLK--RERIKRRIYSNHDEARADVFQYIEMFYNPKRRHSSND
```

GLSPVEFEKQYA"
/note="IS3_IS3_ORF1 e-value= 4.9e-95 complete sequence
hit coverage= 100%, length is 282aa with 6 gaps, 0 stops,
frame= Minus1"
CDS complement(503855..503011)
/colour="255 0 0"
/evidence=predicted
/translation="QHADEFGLAAMCRMLGVHRSGYYAWLKEPASARDKDDQRLLGLI
KHSWLESGSVYGHRKVTTDLRELGETCSRHRVARLMKSEGLRAMvGYGRRPRPLSGPV
GSVAK-NVLARGFKVSE--PNRAWVTDITYIRTYDGFLYLAVVLDLFSRQVVGWATRP
TQHTDLVLQALLAAVWR----RKPSPGLLLHSDQGTQFTSEDWQSFLREHDIVCSMSR
RGNCHDNAAMESFFQLLKRERIKRRIYSNHDEARADVFQYIEMFYNPKRRHSSNDGLS
PVEFEKQYALNGX"
/note="IS3_IS3_ORF2 e-value= 7.1e-102 complete
sequence
hit coverage= 100%, length is 282aa with 7 gaps, 1 stops,
frame= Minus1"
CDS complement(503855..503011)
/colour="255 0 0"
/evidence=predicted
/translation="QHADEFGLAAMCRMLGVHRSGYYAWLKEPASARDKDDQRLLGLI
KHSWLESGSVYGHRKVTTDLRELGETCSRHRVARLMKSEGLRAMvGYGRRPRPLSGPV
GSVAK-NVLARGFKVSE--PNRAWVTDITYIRTYDGFLYLAVVLDLFSRQVVGWATRP
TQHTDLVLQALLAAVWR----RKPSPGLLLHSDQGTQFTSEDWQSFLREHDIVCSMSR
RGNCHDNAAMESFFQLLKRERIKRRIYSNHDEARADVFQYIEMFYNPKRRHSSNDGLS
PVEFEKQYALNGX"
/note="IS3_IS3_ORF2 e-value= 2e-102 complete sequence
hit
coverage= 100%, length is 282aa with 7 gaps, 1 stops,
frame= Minus1"
CDS complement(503843..503017)
/colour="255 0 0"
/evidence=predicted
/translation="EFGLAAMCRMLGVHRSGYYAWLKEPASARDKDDQRLLGLIKHSW
LESGSVYGHRKVTTDLR---ELGETCSRHRVARLMKSEGLRAMVGYGRRPRPLSGPVG
SVAKNVLARGFKVSEPNRAWVTDITYIRTY-DGFLYLAVVLDLFSRQVVGWATRPTQH
TDLVLQALLAAVWRRKPSPGLLLHS-----DQGTQFTSEDWQSFLREHDIVCSMSRRG
NCHDNAAMESFFQLLKRERIK--RRIYSNHDEARADVFQYIEmFYNPKRRHSSNDGLS
PVEFEKQYALN"
/note="IS3_IS150_ORF2 e-value= 1.9e-74 fragment hit
coverage= 73.52%, length is 276aa with 11 gaps, 0 stops,
frame= Minus1"
CDS complement(503840..503023)
/colour="255 0 0"
/evidence=predicted
/translation="FGLAAMCRMLGVH---RSGYYAWLKEPASARDKDDQRLLGLIKH
SWLESGS-----VYGHRKVTTDLRELGETCSRHRVARLMKSEGLRAMVGYGRRPRPLS
GPVGSVAKNVLARGFKVSEPNRAWVTDITYIRTYDGFLYLAVVLDLFSRQVVGWATRP
TQHTDLVLQALLAAVWRRKPS-----PGLLLHSDQGTQFTSEDWQSFLREHDIVCSMS
RRGNCHDNAAMESFFQLLKRERIKRRIYSNHDEARADVFQYIEMFYNPKRRHSSNDGL
SPVEFEKQYA"
/note="IS3_IS51_ORF2 e-value= 7.1e-152 fragment hit
coverage= 90.22%, length is 273aa with 13 gaps, 0 stops,
frame= Minus1"
CDS complement(4482787..4481874)
/colour="255 0 0"
/evidence=predicted
/translation="NARLTPFSRELLVRRITEQGLRPEEAAQACGVSVRTAYKWLARY
RAEGLIGLQNRSSRPSRSPHGTPEALVEQIVQRR-RQRQTYLTISKALG---------
VAHSTISRLMRRRGLNRLCLLDPPK------KAVR-------YEYDSPGGLLHLDIKK

```
                     -----LGNFHRPGHRVDPnRRGNAGGGGWGYVHVAIDDHSRVAFSSVHTDEKGSTACQ
                     ALLAALAYYKTLGVT-FQRVLTDNGACYRSG----VFARLVASLGMKHYRTKPYTPRT
                     NGKAERFIQTSLREWAYACE---YASSEQRDAVLPEWLHYYNWHRPHM--GIGSQPPI
                     SRIPLNNVV"
                     /note="IS481 e-value= 5.6e-93 fragment hit coverage=
                     97.44%, length is 305aa with 38 gaps, 0 stops, frame=
                     Minus2"
     CDS             complement(4482787..4481847)
                     /colour="255 0 0"
                     /evidence=predicted
                     /translation="NARLTPFSRELLVRRITEQGLRPEEAAQACGVSVRTAYKWLARY
                     RAEGLIGLQNRSSRPSRSPHGTPEALVEQIVQRR-RQRQTYLTISKALG---------
                     VAHSTISRLMRRRGLNRLCLLDPPK------KAVR-------YEYDSPGGLLHLDIKK
                     -----LGNFHRPGHRVDPnRRGNAGGGGWGYVHVAIDDHSRVAFSSVHTDEKGSTACQ
                     ALLAALAYYKTLGVT-FQRVLTDNGACYRSG----VFARLVASLGMKHYRTKPYTPRT
                     NGKAERFIQTSLREWAYACE---YASSEQRDAVLPEWLHYYNWHRPHM--GIGSQPPI
                     SRIPLNNVVGLHNXQSTL"
                     /note="IS481 e-value= 1.5e-93 complete sequence hit
                     coverage= 100%, length is 314aa with 38 gaps, 1 stops,
                     frame= Minus2"
     CDS             complement(4482733..4482465)
                     /colour="255 0 0"
                     /evidence=predicted
                     /translation="QGLRPEEAAQACGVSVRTAYKWLARYRAEG---LIGLQ--NRSS
                     RPSRSPHGTPEALVEQIVQRR-------RQRQTYLTISKALG------VAHSTISRLM
                     RRRGLN"
                     /note="IS630 e-value= 8.1e-07 fragment hit coverage=
                     28.72%, length is 90aa with 18 gaps, 0 stops, frame=
                     Minus2"
     CDS             complement(4482166..4481934)
                     /colour="255 0 0"
                     /evidence=predicted
                     /translation="RVLTDNGACYRSGVFARLVASLGMKHYRTKPYTPRTNGKAERFI
                     QTSLREWAY--ACEYASSEQRDAVLPEWLHYYNWHR"
                     /note="IS3_IS150_ORF2    e-value= 2.5e-08 fragment hit
                     coverage= 20.57%, length is 78aa with 2 gaps, 0 stops,
                     frame= Minus2"
     CDS             complement(4037740..4036800)
                     /colour="255 0 0"
                     /evidence=predicted
                     /translation="NARLTVHSRALLIRRILHEGLRPEEAAQACGVSVRTAYKWLARF
                     RQFGAPGLENRSSRPHQTPHATPAPVIEQIKEHR-RKRQTYLTISKALG---------
                     VGHSTISRLMRAHGLNRLCRLEPPK-----EVIR--------YEYDQPGGLLHLDIKK
                     -----LGNFQRPGHRTDAkRRGNAAGGGWGYVHVAIDDHSRVAFSSVHPNEQAETACQ
                     ALLGALQYYASLGIT-FKRILTDNGACYRST----AFAKLLKSLGIKHIRTKPYTPRT
                     NGKAERFIQTSLREWAYARE---YASSDQRNSVLTQWLHHYNWHRPHM--GIGGQPPI
                     SRVLLNNVVGLHSXSTAF"
                     /note="IS481 e-value= 2.4e-94 complete sequence hit
                     coverage= 100%, length is 314aa with 38 gaps, 1 stops,
                     frame= Minus2"
     CDS             complement(4037740..4036800)
                     /colour="255 0 0"
                     /evidence=predicted
                     /translation="NARLTVHSRALLIRRILHEGLRPEEAAQACGVSVRTAYKWLARF
                     RQFGAPGLENRSSRPHQTPHATPAPVIEQIKEHR-RKRQTYLTISKALG---------
                     VGHSTISRLMRAHGLNRLCRLEPPK-----EVIR--------YEYDQPGGLLHLDIKK
                     -----LGNFQRPGHRTDAkRRGNAAGGGWGYVHVAIDDHSRVAFSSVHPNEQAETACQ
                     ALLGALQYYASLGIT-FKRILTDNGACYRST----AFAKLLKSLGIKHIRTKPYTPRT
                     NGKAERFIQTSLREWAYARE---YASSDQRNSVLTQWLHHYNWHRPHM--GIGGQPPI
                     SRVLLNNVVGLHSXSTAF"
```

```
                     /note="IS481 e-value= 6.4e-95 complete sequence hit
                     coverage= 100%, length is 314aa with 38 gaps, 1 stops,
                     frame= Minus2"
     CDS             complement(4037119..4036854)
                     /colour="255 0 0"
                     /evidence=predicted
                     /translation="RILTDNGACYRSTAFAKLLKSLGIKHIRTKPYTPRTNGKAERFI
                     QTSLREWAY--AREYASSDQRNSVLTQWLHHYNWHRPHMGIGGQPPI"
                     /note="IS3_IS150_ORF2    e-value= 1.8e-10 fragment hit
                     coverage= 23.39%, length is 89aa with 2 gaps, 0 stops,
                     frame= Minus2"
     CDS             complement(4036924..4036857)
                     /colour="255 0 0"
                     /evidence=predicted
                     /translation="VLTQWLHHYNWHRPHMGIGGQPP"
                     /note="IS3_IS407_ORF2    e-value= 4.1e-07 fragment hit
                     coverage= 6.69%, length is 23aa with 0 gaps, 0 stops,
                     frame= Minus2"
     CDS             complement(2521012..2520870)
                     /colour="255 0 0"
                     /evidence=predicted
                     /translation="KRYTDEFKIEAVRQIVEYGRPVAEVAERLGVSIHSLYGWKRQQG
                     KGDV"
                     /note="IS3_IS407_ORF1    e-value= 7.7e-06 fragment hit
                     coverage= 55.81%, length is 48aa with 0 gaps, 0 stops,
                     frame= Minus2"
     CDS             complement(2520859..2520732)
                     /colour="255 0 0"
                     /evidence=predicted
                     /translation="VEQDQNAEVRRLKAELRRVTEERDILK--KAAAYFAKGXEQSTP
                     S"
                     /note="IS3_IS51_ORF1     e-value= 3.2e-08 fragment hit
                     coverage= 14.66%, length is 43aa with 2 gaps, 1 stops,
                     frame= Minus2"
     CDS             complement(2508721..2507643)
                     /colour="255 0 0"
                     /evidence=predicted
                     /translation="VQARCGXAGQPAGRQLCPGGPGAGDPGC-IADPLEAGGPEPGpg
                     glRWHRDAPGRGTGAAQARAGPGE-EGAGFFARSGDVLCQGIILR---------YQVI
                     ERCRDEFPVRLMCRCLRVSASGYYDWSKRLPSARERDNQRLLGRIRELHEDSRGTLGA
                     GRMHEDLA---EEGETASLNRVARLMATDGLQGWPRPK-----RRGQRGKPAltppgv
                     RNLLERDFTALEPETRWVTDITELKTR-QGKLYLCIVLDLFDQRVVGWSMHHRQDRQM
                     VIRAVQMAVWQRQGSHPLILHS-----DRGSQFRSGDYQRYLAANGLVCSMSAVGHCG
                     DNAACEGFFGLLKRERVY--RMTYPTLDAARADVFEYIERFHNPRM------------
                     RRRVARQ"
                     /note="IS3_IS150_ORF2    e-value= 1.6e-25 complete sequence
                     hit coverage= 100%, length is 360aa with 39 gaps, 1
                     stops, frame= Minus2"
     CDS             complement(2508514..2507616)
                     /colour="255 0 0"
                     /evidence=predicted
                     /translation="AGFFARSGDVLCQgIILRYQVIERCRDEFPVRLMCRCLRvSASG
                     YYDWSKRLPSARERDnQRLLGRIRELHEDSRGTLGAGRMHEDLAEEGETASLNRVARL
                     MATDGLQGWPRPKR--RGQRGKPALTPPGVrNLLERDFTALEPETRWVTDITELKT--
                     RQGKLYLCIVLDLFDQRVVGWSMHHRQDRQMVIRAVQMAVWQ-RQGSHPLILHSDRGS
                     QFRSGDYQRYLAANGLVCSMSAVGHCGDNAACEGFFGLLKRERVYRMTYPTLD--AAR
                     ADVFEYIERFHNP---RMRRRVARQDQKFSALLQ"
                     /note="IS3_IS150_ORF1    e-value= 1.5e-33 complete sequence
                     hit coverage= 100%, length is 300aa with 10 gaps, 0
                     stops, frame= Minus2"
```

```
     CDS             complement(2508478..2507577)
                     /colour="255 0 0"
                     /evidence=predicted
                     /translation="QGIILRYQVIERCRD--EFPVRLMCRCLRVS---ASGYYDWSKR
                     LPSARERDNQRLLGRIRELHEDSRG-----TLGAGRMHEDLAEEGETASLNRVARLMA
                     TDGLQGW--PRPKRRGQRGKPALTPPgvrNLLERDFTALEPETRWVTDITELKTRQGK
                     LYLCIVLDLFDQRVVGWSMHHRQDRQMVIRAVQMAVWQRQGS-----HPLILHSDRGS
                     QFRSGDYQRYLAANGLVCSMSAVGHCGDNAACEGFFGLLKRERVYRMTYPTLDAARAD
                     VFEYIERFHNPRmRRRVARQD---QKFSALLQPSVISGXNPCNLR"
                     /note="IS3_IS51_ORF2     e-value= 7.4e-58 complete sequence
                     hit coverage= 100%, length is 301aa with 20 gaps, 1
                     stops, frame= Minus2"
     CDS             complement(2508460..2507667)
                     /colour="255 0 0"
                     /evidence=predicted
                     /translation="YQVIERCRDEFPVRLMCRCLRVSASGYYDWS-----KRLPSARE
                     RDNQRLLGRIRELHEDSRGTLGAGRMHEDLAEEGETASLNRVARLMATDGLQGWPRPK
                     RRGQRgKPALTPPGVRNLLERDFTALEPETRWVTDITELKTRQGKLYLCIVLDLFDQR
                     VVGWSMHHRQDRQMV-IRAVQMAVWQRQGSH----PLILHSDRGSQFRSGDYQRYLAA
                     NGLVCSMSAVGHCGDNAACEGFFGLLKRERVYR----MTYPTLDAARADVFEYIERFH
                     N-PR"
                     /note="IS3_IS51_ORF1     e-value= 1.7e-73 fragment hit
                     coverage= 90.88%, length is 265aa with 15 gaps, 0 stops,
                     frame= Minus2"
     CDS             complement(2508460..2507601)
                     /colour="255 0 0"
                     /evidence=predicted
                     /translation="YQVIERCRDEFPVRLMCRCLRVSASGYYDWS-----KRLPSARE
                     RDNQRLLGRIRELHEDSRGTLGAGRMHEDLAEEGETASLNRVARLMATDGLQGWPRPK
                     RRGQRgKPALTPPGVRNLLERDFTALEPETRWVTDITELKTRQGKLYLCIVLDLFDQR
                     VVGWSMHHRQDRQMV-IRAVQMAVWQRQGSH----PLILHSDRGSQFRSGDYQRYLAA
                     NGLVCSMSAVGHCGDNAACEGFFGLLKRERVYR----MTYPTLDAARADVFEYIERFH
                     NPRMRRRVARQDQK-------FSALLQPSVIS"
                     /note="IS3_IS51_ORF1     e-value= 7.6e-67 complete sequence
                     hit coverage= 100%, length is 287aa with 21 gaps, 0
                     stops, frame= Minus2"
     CDS             complement(2508457..2507685)
                     /colour="255 0 0"
                     /evidence=predicted
                     /translation="QVIERCRDEFPVRLMCRCLRVSASGYYDWSKRLPSARERDNQRL
                     LGRIRELHEDSRGTLGAGRMHEDLA---EEGETASLNRVARLMATDGLQGWPRPK---
                     --RRGQRGKPAltppgvRNLLERDFTALEPETRWVTDITELKTR-QGKLYLCIVLDLF
                     DQRVVGWSMHHRQDRQMVIRAVQMAVWQRQGSHPLILHS-----DRGSQFRSGDYQRY
                     LAANGLVCSMSAVGHCGDNAACEGFFGLLKRERVY--RMTYPTLDAARADVFEYIE"
                     /note="IS3_IS150_ORF2    e-value= 1.7e-58 fragment hit
                     coverage= 68.89%, length is 258aa with 16 gaps, 0 stops,
                     frame= Minus2"
     CDS             complement(2508457..2507664)
                     /colour="255 0 0"
                     /evidence=predicted
                     /translation="QVIERCRDEFPVRLMCRCLRVSASGYYDWSKR--LPSARERDNQ
                     RLLGRIRELHEDSRGTLGAGRMHEDLA-EEGETASLNRVARLMATDGLQGWPRPKRRG
                     QRGKpALTpPGVRNLLERDFTALEPETRWVTDITELKTRQGKLYLCIVLDLFDQRVVG
                     WSMHHRQDRQMVIRAVQMAVWQRQGSHPLILHSDRGSQFRSGDYQRYLAANGLVCSMS
                     AVGHCGDNAACEGFFGLLK--RERVYRMTYPTLDAARADVFEYIERFHNP-RM"
                     /note="IS3_IS3_ORF1      e-value= 4e-75 fragment hit
coverage=
                     94.06%, length is 265aa with 6 gaps, 0 stops, frame=
                     Minus2"
     CDS             complement(2508457..2507625)
```

```
                     /colour="255 0 0"
                     /evidence=predicted
                     /translation="QVIERCRDEFPVRLMCRCLRVSASGYYDWSKR--LPSARERDNQ
                     RLLGRIRELHEDSRGTLGAGRMHEDLA-EEGETASLNRVARLMATDGLQGWPRPKRRG
                     QRGKpALTpPGVRNLLERDFTALEPETRWVTDITELKTRQGKLYLCIVLDLFDQRVVG
                     WSMHHRQDRQMVIRAVQMAVWQRQGSHPLILHSDRGSQFRSGDYQRYLAANGLVCSMS
                     AVGHCGDNAACEGFFGLLK--RERVYRMTYPTLDAARADVFEYIERFHNP-RMRRRVA
                     ----RQDQKFSA"
                     /note="IS3_IS3_ORF1      e-value= 2.4e-72 complete sequence
                     hit coverage= 100%, length is 278aa with 10 gaps, 0
                     stops, frame= Minus2"
     CDS             complement(2508445..2507649)
                     /colour="255 0 0"
                     /evidence=predicted
                     /translation="RCRDEFPVRLMCRCLRVSASGYYDWSKRLPSARERDNQRLLGRI
                     RELHEDSRGTLGAGRMHEDLAEEGETASLNRVARLMATDGLQGWPRPKRRGQRGK--P
                     ALTPPgvrNLLERDFTALE--PETRWVTDITELKTRQGKLYLCIVLDLFDQRVVGWSM
                     HHRQDRQMVIRAVQMAVWQ----RQGSHPLILHSDRGSQFRSGDYQRYLAANGLVCSM
                     SAVGHCGDNAACEGFFGLLKRERVYRMTYPTLDAARADVFEYIERFHNpRMRRRVA"
                     /note="IS3_IS3_ORF2      e-value= 2.2e-69 fragment hit
                     coverage= 93.75%, length is 266aa with 8 gaps, 0 stops,
                     frame= Minus2"
     CDS             complement(2508445..2507628)
                     /colour="255 0 0"
                     /evidence=predicted
                     /translation="RCRDEFPVRLMCRCLRVSASGYYDWSKRLPSARERDNQRLLGRI
                     RELHEDSRGTLGAGRMHEDLAEEGETASLNRVARLMATDGLQGWPRPKRRGQRGK--P
                     ALTPPgvrNLLERDFTALE--PETRWVTDITELKTRQGKLYLCIVLDLFDQRVVGWSM
                     HHRQDRQMVIRAVQMAVWQ----RQGSHPLILHSDRGSQFRSGDYQRYLAANGLVCSM
                     SAVGHCGDNAACEGFFGLLKRERVYRMTYPTLDAARADVFEYIERFHNpRMRRRVA--
                     ---------RQDQKFS"
                     /note="IS3_IS3_ORF2      e-value= 2.6e-66 complete sequence
                     hit coverage= 100%, length is 273aa with 19 gaps, 0
                     stops, frame= Minus2"
     CDS             complement(2508424..2507670)
                     /colour="255 0 0"
                     /evidence=predicted
                     /translation="VRLMCRCLRVS---ASGYYDWSKRLPSARERDNQRLLGRIRELH
                     EDSRG-----TLGAGRMHEDLAEEGETASLNRVARLMATDGLQGW--PRPKRRGQRGK
                     PALTPPgvrNLLERDFTALEPETRWVTDITELKTRQGKLYLCIVLDLFDQRVVGWSMH
                     HRQDRQMVIRAVQMAVWQRQGS-----HPLILHSDRGSQFRSGDYQRYLAANGLVCSM
                     SAVGHCGDNAACEGFFGLLKRERVYRMTYPTLDAARADVFEYIERFHNP"
                     /note="IS3_IS51_ORF2     e-value= 1.5e-73 fragment hit
                     coverage= 83.28%, length is 252aa with 15 gaps, 0 stops,
                     frame= Minus2"
     CDS             complement(2508394..2507673)
                     /colour="255 0 0"
                     /evidence=predicted
                     /translation="SASGYYDWSKRLPSARERDnQRLLGRIRELHEDSRGTLGAGRMH
                     EDLAEEGETASLNRVARLMATDGLQGWPRPKR--RGQRGKPALTPPGVrNLLERDFTA
                     LEPETRWVTDITELKT--RQGKLYLCIVLDLFDQRVVGWSMHHRQDRQMVIRAVQMAV
                     WQ-RQGSHPLILHSDRGSQFRSGDYQRYLAANGLVCSMSAVGHCGDNAACEGFFGLLK
                     RERVYRMTYPTLD--AARADVFEYIERFHN"
                     /note="IS3_IS150_ORF1    e-value= 2.4e-37 fragment hit
                     coverage= 80.39%, length is 241aa with 7 gaps, 0 stops,
                     frame= Minus2"
     CDS             complement(2447827..2447712)
                     /colour="255 0 0"
                     /evidence=predicted
                     /translation="KQYTDEFKAEAVKQVTERGFAVAEVAKRLGVSSHSLYQW"
```

```
                     /note="IS3_IS407_ORF1    e-value= 7e-06 fragment hit
                     coverage= 45.35%, length is 39aa with 0 gaps, 0 stops,
                     frame= Minus2"
     CDS             complement(2447419..2446239)
                     /colour="255 0 0"
                     /evidence=predicted
                     /translation="FLQLRSLKTLEILTSEDS-YVVKAEG--------------VNQW
                     VTCPLCKTGCLHGHGSQEQSFQDTPTHGKPVVIFIQRRRYRCTSCTKTlFEPVADLDG
                     KRQATARLVRYIRDQSFSK--TFAALAREVAVEEKTVRHVFDDFIEEVEAKTQFRTPR
                     VLGIDELKIIG----QYRAMITNVERK----TVFDIRPSRAKAELMPYFR---DLRDK
                     DSVEWVAMDMYHVYRQVVRATLPQARIVVDRFHIQRMANDALEKLR---KRIRK----
                     ------------DLSTR--------QRLKLKDERFLLLKRQHDLTGR-DMDRMREWFQ
                     --QFPLLSEAHALKEGFLSIWDQKTRPAAEAACAKWQANIPTELAAtFKDLTTAVHNW
                     HDEIFA-YFEQPITNAYTESVNRVAKDMNRMGRGYS-FEVLRARMLYdKKARKDGSVI
                     ETVL"
                     /note="ISL3  e-value= 1.1e-43 complete sequence hit
                     coverage= 100%, length is 394aa with 60 gaps, 0 stops,
                     frame= Minus2"
     CDS             complement(2447329..2446284)
                     /colour="255 0 0"
                     /evidence=predicted
                     /translation="TCPLCKTGCLHGHGSQEQSFQDTPTHGKPVVIFIQRRRYRCTSC
                     TKTlFEPVADLDGKRQATARLVRYIRDQSFSK--TFAALAREVAVEEKTVRHVFDDFI
                     EEVEAKTQFRTPRVLGIDELKIIG----QYRAMITNVERK----TVFDIRPSRAKAEL
                     MPYFR---DLRDKDSVEWVAMDMYHVYRQVVRATLPQARIVVDRFHIQRMANDALEKL
                     RKRIRKDLSTRQRLkLKDERFLLLKRQHDLTGRDMDRMREWFQQFPLLSEAHALKEGF
                     LSI-WDQKTRPAAEAACAKWQANIPTELAATFKDLTTAVHNWHDEIFAYFEQPI--TN
                     AYTESVNRVAKDMNRMGRGYSFEVLRARMLY"
                     /note="ISL3  e-value= 5.3e-50 fragment hit coverage=
                     80.49%, length is 349aa with 16 gaps, 0 stops, frame=
                     Minus2"
     CDS             complement(2023897..2023779)
                     /colour="255 0 0"
                     /evidence=predicted
                     /translation="PPEQREVLQLRLDQELSLEEIGQITGVGRETVKSRLRYAM"
                     /note="IS21_ORF1   e-value= 2.4e-06 fragment hit coverage=
                     7.62%, length is 40aa with 0 gaps, 0 stops, frame=
                     Minus2"
     CDS             complement(1143877..1142961)
                     /colour="255 0 0"
                     /evidence=predicted
                     /translation="NGIGIDVSKRRLDIGTTDGQT------LQVSNSPSGYAELSTWL
                     STRP-----AIQIVLEATGGYEQTVLDFLHKA---GHPVIRANALRARRLA-QGLGKV
                     AKTDRLDALVLAQMA--ALVELPSYQPLEPW----QRKLREFVRARRQTMQALTVARQ
                     QQEMVTDRELRRQMQG------------------------------------------
                     ---------------------------NIIRLQTLVERLGKQISEQVAQ-QPQLAVL
                     KS---------MKGVGPALQAVLASYLP-ELGQISGK-AIASLVGVAPISHDSG-AMR
                     GRRSIHG-GRAEIRQVLYMAAMSAMRHEPRLRDFYRSLRARGK---EGKVAIVAVMRK
                     MLVILNARVRDAE--NGLVPAXGQ"
                     /note="IS110 e-value= 3.4e-46 complete sequence hit
                     coverage= 100%, length is 306aa with 110 gaps, 1 stops,
                     frame= Minus2"
     CDS             complement(1143877..1142994)
                     /colour="255 0 0"
                     /evidence=predicted
                     /translation="NGIGIDVSKRRLDIGTTDGQT------LQVSNSPSGYAELSTWL
                     STRP-----AIQIVLEATGGYEQTVLDFLHKA---GHPVIRANALRARRLA-QGLGKV
                     AKTDRLDALVLAQMA--ALVELPSYQPLEPW----QRKLREFVRARRQTMQALTVARQ
                     QQEMVTDRELRRQMQG------------------------------------------
                     ---------------------------NIIRLQTLVERLGKQISEQVAQ-QPQLAVL
```

KS---------MKGVGPALQAVLASYLP-ELGQISGK-AIASLVGVAPISHDSG-AMR
GRRSIHG-GRAEIRQVLYMAAMSAMRHEPRLRDFYRSLRARGK---EGKVAIVAVMRK
MLVILNARVRD"
/note="IS110 e-value= 1e-48 fragment hit coverage=
96.88%, length is 295aa with 108 gaps, 0 stops, frame=
Minus2"
CDS complement(730597..729681)
/colour="255 0 0"
/evidence=predicted
/translation="NGIGIDVSKRRLDIGTTDGQT------LQVSNSPSGYAELSTWL
STRP-----AIQIVLEATGGYEQTVLDFLHKA---GHPVIRANALRARRLA-QGLGKV
AKTDRLDALVLAQMA--ALVELPSYQPLEPW----QRKLREFVRARRQTMQALTVARQ
QQEMVTDRELRRQMQG--
----------------------------NIIRLQTLVERLGKQISEQVAQ-QPQLAVL
KS---------MKGGGPALQAVLASYLP-ELGQISGK-AIASLVGVAPISHDSG-AMR
GRRSIHG-GRAEIRQVLYMAAMSAMRHEPRLRDFYRSLRARGK---EGKVAIVAVMRK
MLVILNARVRDAE--NGLVPAXGQ"
/note="IS110 e-value= 2.5e-43 complete sequence hit
coverage= 100%, length is 306aa with 110 gaps, 1 stops,
frame= Minus2"
CDS complement(730597..729714)
/colour="255 0 0"
/evidence=predicted
/translation="NGIGIDVSKRRLDIGTTDGQT------LQVSNSPSGYAELSTWL
STRP-----AIQIVLEATGGYEQTVLDFLHKA---GHPVIRANALRARRLA-QGLGKV
AKTDRLDALVLAQMA--ALVELPSYQPLEPW----QRKLREFVRARRQTMQALTVARQ
QQEMVTDRELRRQMQG--
----------------------------NIIRLQTLVERLGKQISEQVAQ-QPQLAVL
KS---------MKGGGPALQAVLASYLP-ELGQISGK-AIASLVGVAPISHDSG-AMR
GRRSIHG-GRAEIRQVLYMAAMSAMRHEPRLRDFYRSLRARGK---EGKVAIVAVMRK
MLVILNARVRD"
/note="IS110 e-value= 5.3e-46 fragment hit coverage=
96.88%, length is 295aa with 108 gaps, 0 stops, frame=
Minus2"
CDS complement(687505..686589)
/colour="255 0 0"
/evidence=predicted
/translation="NGIGIDVSKRRLDIGTTDGQT------LQVSNSPSGYAELSTWL
STRP-----AIQIVLEATGGYEQTVLDFLHKA---GHPVIRANALRARRLA-QGLGKV
AKTDRLDALVLAQMA--ALVELPSYQPLEPW----QRKLREFVRARRQTMQALTVARQ
QQEMVTDRELRRQMQG--
----------------------------NIIRLQTLVERLGKQISEQVAQ-QPQLAVL
KS---------MKGVGPALQAVLASYLP-ELGQISGK-AIASLVGVAPISHDSG-AMR
GRRSIHG-GRAEIRQVLYMAAMSAMRHEPRLRDFYRSLRARGK---EGKVAIVAVMRK
MLVILNARVRDAE--NGLVPAXGQ"
/note="IS110 e-value= 3.4e-46 complete sequence hit
coverage= 100%, length is 306aa with 110 gaps, 1 stops,
frame= Minus2"
CDS complement(687505..686622)
/colour="255 0 0"
/evidence=predicted
/translation="NGIGIDVSKRRLDIGTTDGQT------LQVSNSPSGYAELSTWL
STRP-----AIQIVLEATGGYEQTVLDFLHKA---GHPVIRANALRARRLA-QGLGKV
AKTDRLDALVLAQMA--ALVELPSYQPLEPW----QRKLREFVRARRQTMQALTVARQ
QQEMVTDRELRRQMQG--
----------------------------NIIRLQTLVERLGKQISEQVAQ-QPQLAVL
KS---------MKGVGPALQAVLASYLP-ELGQISGK-AIASLVGVAPISHDSG-AMR
GRRSIHG-GRAEIRQVLYMAAMSAMRHEPRLRDFYRSLRARGK---EGKVAIVAVMRK
MLVILNARVRD"
/note="IS110 e-value= 1e-48 fragment hit coverage=

96.88%, length is 295aa with 108 gaps, 0 stops, frame= Minus2"

CDS complement(504142..504000)
/colour="255 0 0"
/evidence=predicted
/translation="KRYTDEFKIEAVRQIVEYGRPVAEVAERLGVSIHSLYGWKRQQG
KGDV"
/note="IS3_IS407_ORF1 e-value= 7.7e-06 fragment hit coverage= 55.81%, length is 48aa with 0 gaps, 0 stops, frame= Minus2"

CDS complement(503989..503862)
/colour="255 0 0"
/evidence=predicted
/translation="VEQDQNAEVRRLKAELRRVTEERDILK--KAAAYFAKGXEQSTP
S"
/note="IS3_IS51_ORF1 e-value= 3.2e-08 fragment hit coverage= 14.66%, length is 43aa with 2 gaps, 1 stops, frame= Minus2"

CDS complement(370078..369171)
/colour="255 0 0"
/evidence=predicted
/translation="FGIGIDVSKATLDVAVHGEQ------FRQFSNDKRGFVRLIRWL
KTWP-----IKQVVLEASGGYERAALDVLHAA---GLPMVRINPARARRFAQGT-GRA
AKTDRLDATVLAQMAHLLPLTRYVP--PAPW---QQRLAEFAQCRRQLVQMRVSEMQR
LRRLVDPDLIA--
----------------------------MKQRHIAQLSDDLKQLDKAIAEQLEGQPGW
KNIGA------LKGVGPILISTLACELP-ELGSLGSK-AIASLVGLAPMNRESG-TWQ
GXRRISG-GRAVVREALYMAALTAIRYEPRLRAFYAGLKAKGK---ASKVALVAVMRK
MLVILNARKR-----DAEVALGCP"
/note="IS110 e-value= 6.9e-48 complete sequence hit coverage= 100%, length is 303aa with 113 gaps, 1 stops, frame= Minus2"

CDS complement(370078..369204)
/colour="255 0 0"
/evidence=predicted
/translation="FGIGIDVSKATLDVAVHGEQ------FRQFSNDKRGFVRLIRWL
KTWP-----IKQVVLEASGGYERAALDVLHAA---GLPMVRINPARARRFAQGT-GRA
AKTDRLDATVLAQMAHLLPLTRYVP--PAPW---QQRLAEFAQCRRQLVQMRVSEMQR
LRRLVDPDLIA--
----------------------------MKQRHIAQLSDDLKQLDKAIAEQLEGQPGW
KNIGA------LKGVGPILISTLACELP-ELGSLGSK-AIASLVGLAPMNRESG-TWQ
GXRRISG-GRAVVREALYMAALTAIRYEPRLRAFYAGLKAKGK---ASKVALVAVMRK
MLVILNAR"
/note="IS110 e-value= 4.1e-51 fragment hit coverage= 96.15%, length is 292aa with 108 gaps, 1 stops, frame= Minus2"

CDS complement(27022..25944)
/colour="255 0 0"
/evidence=predicted
/translation="VQARCGXAGQPAGRQLCPGGPGAGDPGC-IADPLEAGGPEPGpg
glRWHRDAPGRGTGAAQARAGPGE-EGAGFFARSGDVLCQGIILR---------YQVI
ERCRDEFPVRLMCRCLRVSASGYYDWSKRLPSARERDNQRLLGRIRELHEDSRGTLGA
GRMHEDLA---EEGETASLNRVARLMATDGLQGWPRPK-----RRGQRGKPAltppgv
RNLLERDFTALEPETRWVTDITELKTR-QGKLYLCIVLDLFDQRVVGWSMHHRQDRQM
VIRAVQMAVWQRQGSHPLILHS-----DRGSQFRSGDYQRYLAANGLVCSMSAVGHCG
DNAACEGFFGLLKRERVY--RMTYPTLDAARADVFEYIERFHNPRM------------
RRRVARQ"
/note="IS3_IS150_ORF2 e-value= 1.6e-25 complete sequence hit coverage= 100%, length is 360aa with 39 gaps, 1 stops, frame= Minus2"

```
CDS             complement(26815..25917)
                /colour="255 0 0"
                /evidence=predicted
                /translation="AGFFARSGDVLCQgIILRYQVIERCRDEFPVRLMCRCLRvSASG
                YYDWSKRLPSARERDnQRLLGRIRELHEDSRGTLGAGRMHEDLAEEGETASLNRVARL
                MATDGLQGWPRPKR--RGQRGKPALTPPGVrNLLERDFTALEPETRWVTDITELKT--
                RQGKLYLCIVLDLFDQRVVGWSMHHRQDRQMVIRAVQMAVWQ-RQGSHPLILHSDRGS
                QFRSGDYQRYLAANGLVCSMSAVGHCGDNAACEGFFGLLKRERVYRMTYPTLD--AAR
                ADVFEYIERFHNP---RMRRRVARQDQKFSALLQ"
                /note="IS3_IS150_ORF1    e-value= 1.5e-33 complete sequence
                hit coverage= 100%, length is 300aa with 10 gaps, 0
                stops, frame= Minus2"
CDS             complement(26779..25851)
                /colour="255 0 0"
                /evidence=predicted
                /translation="QGIILRYQVIERCRD--EFPVRLMCRCLRVS---ASGYYDWSKR
                LPSARERDNQRLLGRIRELHEDSRG-----TLGAGRMHEDLAEEGETASLNRVARLMA
                TDGLQGW--PRPKRRGQRGKPALTPPgvrNLLERDFTALEPETRWVTDITELKTRQGK
                LYLCIVLDLFDQRVVGWSMHHRQDRQMVIRAVQMAVWQRQGS-----HPLILHSDRGS
                QFRSGDYQRYLAANGLVCSMSAVGHCGDNAACEGFFGLLKRERVYRMTYPTLDAARAD
                VFEYIERFHNPRmRRRVARQDqkfsalLQPSVISGXNPNSGAGXGGEARLS"
                /note="IS3_IS51_ORF2     e-value= 9.6e-57 complete sequence
                hit coverage= 100%, length is 310aa with 17 gaps, 2
                stops, frame= Minus2"
CDS             complement(26761..25968)
                /colour="255 0 0"
                /evidence=predicted
                /translation="YQVIERCRDEFPVRLMCRCLRVSASGYYDWS-----KRLPSARE
                RDNQRLLGRIRELHEDSRGTLGAGRMHEDLAEEGETASLNRVARLMATDGLQGWPRPK
                RRGQRgKPALTPPGVRNLLERDFTALEPETRWVTDITELKTRQGKLYLCIVLDLFDQR
                VVGWSMHHRQDRQMV-IRAVQMAVWQRQGSH----PLILHSDRGSQFRSGDYQRYLAA
                NGLVCSMSAVGHCGDNAACEGFFGLLKRERVYR----MTYPTLDAARADVFEYIERFH
                N-PR"
                /note="IS3_IS51_ORF1     e-value= 1.7e-73 fragment hit
                coverage= 90.88%, length is 265aa with 15 gaps, 0 stops,
                frame= Minus2"
CDS             complement(26761..25902)
                /colour="255 0 0"
                /evidence=predicted
                /translation="YQVIERCRDEFPVRLMCRCLRVSASGYYDWS-----KRLPSARE
                RDNQRLLGRIRELHEDSRGTLGAGRMHEDLAEEGETASLNRVARLMATDGLQGWPRPK
                RRGQRgKPALTPPGVRNLLERDFTALEPETRWVTDITELKTRQGKLYLCIVLDLFDQR
                VVGWSMHHRQDRQMV-IRAVQMAVWQRQGSH----PLILHSDRGSQFRSGDYQRYLAA
                NGLVCSMSAVGHCGDNAACEGFFGLLKRERVYR----MTYPTLDAARADVFEYIERFH
                NPRMRRRVARQDQK-------FSALLQPSVIS"
                /note="IS3_IS51_ORF1     e-value= 7.6e-67 complete sequence
                hit coverage= 100%, length is 287aa with 21 gaps, 0
                stops, frame= Minus2"
CDS             complement(26758..25986)
                /colour="255 0 0"
                /evidence=predicted
                /translation="QVIERCRDEFPVRLMCRCLRVSASGYYDWSKRLPSARERDNQRL
                LGRIRELHEDSRGTLGAGRMHEDLA---EEGETASLNRVARLMATDGLQGWPRPK---
                --RRGQRGKPAltppgvRNLLERDFTALEPETRWVTDITELKTR-QGKLYLCIVLDLF
                DQRVVGWSMHHRQDRQMVIRAVQMAVWQRQGSHPLILHS-----DRGSQFRSGDYQRY
                LAANGLVCSMSAVGHCGDNAACEGFFGLLKRERVY--RMTYPTLDAARADVFEYIE"
                /note="IS3_IS150_ORF2    e-value= 1.7e-58 fragment hit
                coverage= 68.89%, length is 258aa with 16 gaps, 0 stops,
                frame= Minus2"
CDS             complement(26758..25965)
```

```
                     /colour="255 0 0"
                     /evidence=predicted
                     /translation="QVIERCRDEFPVRLMCRCLRVSASGYYDWSKR--LPSARERDNQ
                     RLLGRIRELHEDSRGTLGAGRMHEDLA-EEGETASLNRVARLMATDGLQGWPRPKRRG
                     QRGKpALTpPGVRNLLERDFTALEPETRWVTDITELKTRQGKLYLCIVLDLFDQRVVG
                     WSMHHRQDRQMVIRAVQMAVWQRQGSHPLILHSDRGSQFRSGDYQRYLAANGLVCSMS
                     AVGHCGDNAACEGFFGLLK--RERVYRMTYPTLDAARADVFEYIERFHNP-RM"
                     /note="IS3_IS3_ORF1      e-value= 4e-75 fragment hit
coverage=
                     94.06%, length is 265aa with 6 gaps, 0 stops, frame=
                     Minus2"
     CDS             complement(26758..25926)
                     /colour="255 0 0"
                     /evidence=predicted
                     /translation="QVIERCRDEFPVRLMCRCLRVSASGYYDWSKR--LPSARERDNQ
                     RLLGRIRELHEDSRGTLGAGRMHEDLA-EEGETASLNRVARLMATDGLQGWPRPKRRG
                     QRGKpALTpPGVRNLLERDFTALEPETRWVTDITELKTRQGKLYLCIVLDLFDQRVVG
                     WSMHHRQDRQMVIRAVQMAVWQRQGSHPLILHSDRGSQFRSGDYQRYLAANGLVCSMS
                     AVGHCGDNAACEGFFGLLK--RERVYRMTYPTLDAARADVFEYIERFHNP-RMRRRVA
                     ----RQDQKFSA"
                     /note="IS3_IS3_ORF1      e-value= 2.4e-72 complete sequence
                     hit coverage= 100%, length is 278aa with 10 gaps, 0
                     stops, frame= Minus2"
     CDS             complement(26746..25950)
                     /colour="255 0 0"
                     /evidence=predicted
                     /translation="RCRDEFPVRLMCRCLRVSASGYYDWSKRLPSARERDNQRLLGRI
                     RELHEDSRGTLGAGRMHEDLAEEGETASLNRVARLMATDGLQGWPRPKRRGQRGK--P
                     ALTPPgvrNLLERDFTALE--PETRWVTDITELKTRQGKLYLCIVLDLFDQRVVGWSM
                     HHRQDRQMVIRAVQMAVWQ----RQGSHPLILHSDRGSQFRSGDYQRYLAANGLVCSM
                     SAVGHCGDNAACEGFFGLLKRERVYRMTYPTLDAARADVFEYIERFHNpRMRRRVA"
                     /note="IS3_IS3_ORF2      e-value= 2.2e-69 fragment hit
                     coverage= 93.75%, length is 266aa with 8 gaps, 0 stops,
                     frame= Minus2"
     CDS             complement(26746..25929)
                     /colour="255 0 0"
                     /evidence=predicted
                     /translation="RCRDEFPVRLMCRCLRVSASGYYDWSKRLPSARERDNQRLLGRI
                     RELHEDSRGTLGAGRMHEDLAEEGETASLNRVARLMATDGLQGWPRPKRRGQRGK--P
                     ALTPPgvrNLLERDFTALE--PETRWVTDITELKTRQGKLYLCIVLDLFDQRVVGWSM
                     HHRQDRQMVIRAVQMAVWQ----RQGSHPLILHSDRGSQFRSGDYQRYLAANGLVCSM
                     SAVGHCGDNAACEGFFGLLKRERVYRMTYPTLDAARADVFEYIERFHNpRMRRRVA--
                     ---------RQDQKFS"
                     /note="IS3_IS3_ORF2      e-value= 2.6e-66 complete sequence
                     hit coverage= 100%, length is 273aa with 19 gaps, 0
                     stops, frame= Minus2"
     CDS             complement(26725..25971)
                     /colour="255 0 0"
                     /evidence=predicted
                     /translation="VRLMCRCLRVS---ASGYYDWSKRLPSARERDNQRLLGRIRELH
                     EDSRG-----TLGAGRMHEDLAEEGETASLNRVARLMATDGLQGW--PRPKRRGQRGK
                     PALTPPgvrNLLERDFTALEPETRWVTDITELKTRQGKLYLCIVLDLFDQRVVGWSMH
                     HRQDRQMVIRAVQMAVWQRQGS-----HPLILHSDRGSQFRSGDYQRYLAANGLVCSM
                     SAVGHCGDNAACEGFFGLLKRERVYRMTYPTLDAARADVFEYIERFHNP"
                     /note="IS3_IS51_ORF2     e-value= 1.5e-73 fragment hit
                     coverage= 83.28%, length is 252aa with 15 gaps, 0 stops,
                     frame= Minus2"
     CDS             complement(26695..25974)
                     /colour="255 0 0"
                     /evidence=predicted
```

```
                     /translation="SASGYYDWSKRLPSARERDnQRLLGRIRELHEDSRGTLGAGRMH
                     EDLAEEGETASLNRVARLMATDGLQGWPRPKR--RGQRGKPALTPPGVrNLLERDFTA
                     LEPETRWVTDITELKT--RQGKLYLCIVLDLFDQRVVGWSMHHRQDRQMVIRAVQMAV
                     WQ-RQGSHPLILHSDRGSQFRSGDYQRYLAANGLVCSMSAVGHCGDNAACEGFFGLLK
                     RERVYRMTYPTLD--AARADVFEYIERFHN"
                     /note="IS3_IS150_ORF1    e-value= 2.4e-37 fragment hit
                     coverage= 80.39%, length is 241aa with 7 gaps, 0 stops,
                     frame= Minus2"
     CDS             complement(4665888..4665734)
                     /colour="255 0 0"
                     /evidence=predicted
                     /translation="QLTFGDTQGLGKRKQTRREILLYEVDXVVPWKALLARIEPHYP-
                     RSGRVGQRA"
                     /note="IS5_IS5     e-value= 3.8e-15 fragment hit coverage=
                     12.56%, length is 52aa with 1 gaps, 1 stops, frame=
                     Minus3"
     CDS             complement(3123708..3122633)
                     /colour="255 0 0"
                     /evidence=predicted
                     /translation="-SSSAVRLSRPAGRQLCPGGPGAGDPGC-IADPLEAGGPEPGpg
                     glRWHRDAPGRGTGAAQARAGPGE-EGAGFFARSGDVLCQGIILR---------YQVI
                     ERCRDEFPVRLMCRCLRVSASGYYDWSKRLPSARERDNQRLLGRIRELHEDSRGTLGA
                     GRMHEDLA---EEGETASLNRVARLMATDGLQGWPRPK-----RRGQRGKPAltppgv
                     RNLLERDFTALEPETRWVTDITELKTR-QGKLYLCIVLDLFDQRVVGWSMHHRQDRQM
                     VIRAVQMAVWQRQGSHPLILHS-----DRGSQFRSGDYQRYLAANGLVCSMSAVGHCG
                     DNAACEGFFGLLKRERVY--RMTYPTLDAARADVFEYIERFHNPRM------------
                     RRRVARQ"
                     /note="IS3_IS150_ORF2    e-value= 8.1e-26 complete sequence
                     hit coverage= 100%, length is 359aa with 40 gaps, 0
                     stops, frame= Minus3"
     CDS             complement(3123504..3122606)
                     /colour="255 0 0"
                     /evidence=predicted
                     /translation="AGFFARSGDVLCQgIILRYQVIERCRDEFPVRLMCRCLRvSASG
                     YYDWSKRLPSARERDnQRLLGRIRELHEDSRGTLGAGRMHEDLAEEGETASLNRVARL
                     MATDGLQGWPRPKR--RGQRGKPALTPPGVrNLLERDFTALEPETRWVTDITELKT--
                     RQGKLYLCIVLDLFDQRVVGWSMHHRQDRQMVIRAVQMAVWQ-RQGSHPLILHSDRGS
                     QFRSGDYQRYLAANGLVCSMSAVGHCGDNAACEGFFGLLKRERVYRMTYPTLD--AAR
                     ADVFEYIERFHNP---RMRRRVARQDQKFSALLQ"
                     /note="IS3_IS150_ORF1    e-value= 1.5e-33 complete sequence
                     hit coverage= 100%, length is 300aa with 10 gaps, 0
                     stops, frame= Minus3"
     CDS             complement(3123468..3122558)
                     /colour="255 0 0"
                     /evidence=predicted
                     /translation="QGIILRYQVIERCRD--EFPVRLMCRCLRVS---ASGYYDWSKR
                     LPSARERDNQRLLGRIRELHEDSRG-----TLGAGRMHEDLAEEGETASLNRVARLMA
                     TDGLQGW--PRPKRRGQRGKPALTPPgvrNLLERDFTALEPETRWVTDITELKTRQGK
                     LYLCIVLDLFDQRVVGWSMHHRQDRQMVIRAVQMAVWQRQGS-----HPLILHSDRGS
                     QFRSGDYQRYLAANGLVCSMSAVGHCGDNAACEGFFGLLKRERVYRMTYPTLDAARAD
                     VFEYIERFHNPRmRRRVARQD---QKFSALLqpsVISGXNPRQSRTTR"
                     /note="IS3_IS51_ORF2     e-value= 2.2e-57 complete sequence
                     hit coverage= 100%, length is 304aa with 20 gaps, 1
                     stops, frame= Minus3"
     CDS             complement(3123450..3122657)
                     /colour="255 0 0"
                     /evidence=predicted
                     /translation="YQVIERCRDEFPVRLMCRCLRVSASGYYDWS-----KRLPSARE
                     RDNQRLLGRIRELHEDSRGTLGAGRMHEDLAEEGETASLNRVARLMATDGLQGWPRPK
                     RRGQRgKPALTPPGVRNLLERDFTALEPETRWVTDITELKTRQGKLYLCIVLDLFDQR
```

```
                     VVGWSMHHRQDRQMV-IRAVQMAVWQRQGSH----PLILHSDRGSQFRSGDYQRYLAA
                     NGLVCSMSAVGHCGDNAACEGFFGLLKRERVYR----MTYPTLDAARADVFEYIERFH
                     N-PR"
                     /note="IS3_IS51_ORF1     e-value= 1.7e-73 fragment hit
                     coverage= 90.88%, length is 265aa with 15 gaps, 0 stops,
                     frame= Minus3"
     CDS             complement(3123450..3122591)
                     /colour="255 0 0"
                     /evidence=predicted
                     /translation="YQVIERCRDEFPVRLMCRCLRVSASGYYDWS-----KRLPSARE
                     RDNQRLLGRIRELHEDSRGTLGAGRMHEDLAEEGETASLNRVARLMATDGLQGWPRPK
                     RRGQRgKPALTPPGVRNLLERDFTALEPETRWVTDITELKTRQGKLYLCIVLDLFDQR
                     VVGWSMHHRQDRQMV-IRAVQMAVWQRQGSH----PLILHSDRGSQFRSGDYQRYLAA
                     NGLVCSMSAVGHCGDNAACEGFFGLLKRERVYR----MTYPTLDAARADVFEYIERFH
                     NPRMRRRVARQDQK-------FSALLQPSVIS"
                     /note="IS3_IS51_ORF1     e-value= 7.6e-67 complete sequence
                     hit coverage= 100%, length is 287aa with 21 gaps, 0
                     stops, frame= Minus3"
     CDS             complement(3123447..3122675)
                     /colour="255 0 0"
                     /evidence=predicted
                     /translation="QVIERCRDEFPVRLMCRCLRVSASGYYDWSKRLPSARERDNQRL
                     LGRIRELHEDSRGTLGAGRMHEDLA---EEGETASLNRVARLMATDGLQGWPRPK---
                     --RRGQRGKPAltppgvRNLLERDFTALEPETRWVTDITELKTR-QGKLYLCIVLDLF
                     DQRVVGWSMHHRQDRQMVIRAVQMAVWQRQGSHPLILHS-----DRGSQFRSGDYQRY
                     LAANGLVCSMSAVGHCGDNAACEGFFGLLKRERVY--RMTYPTLDAARADVFEYIE"
                     /note="IS3_IS150_ORF2    e-value= 1.7e-58 fragment hit
                     coverage= 68.89%, length is 258aa with 16 gaps, 0 stops,
                     frame= Minus3"
     CDS             complement(3123447..3122654)
                     /colour="255 0 0"
                     /evidence=predicted
                     /translation="QVIERCRDEFPVRLMCRCLRVSASGYYDWSKR--LPSARERDNQ
                     RLLGRIRELHEDSRGTLGAGRMHEDLA-EEGETASLNRVARLMATDGLQGWPRPKRRG
                     QRGKpALTpPGVRNLLERDFTALEPETRWVTDITELKTRQGKLYLCIVLDLFDQRVVG
                     WSMHHRQDRQMVIRAVQMAVWQRQGSHPLILHSDRGSQFRSGDYQRYLAANGLVCSMS
                     AVGHCGDNAACEGFFGLLK--RERVYRMTYPTLDAARADVFEYIERFHNP-RM"
                     /note="IS3_IS3_ORF1      e-value= 4e-75 fragment hit
coverage=
                     94.06%, length is 265aa with 6 gaps, 0 stops, frame=
                     Minus3"
     CDS             complement(3123447..3122615)
                     /colour="255 0 0"
                     /evidence=predicted
                     /translation="QVIERCRDEFPVRLMCRCLRVSASGYYDWSKR--LPSARERDNQ
                     RLLGRIRELHEDSRGTLGAGRMHEDLA-EEGETASLNRVARLMATDGLQGWPRPKRRG
                     QRGKpALTpPGVRNLLERDFTALEPETRWVTDITELKTRQGKLYLCIVLDLFDQRVVG
                     WSMHHRQDRQMVIRAVQMAVWQRQGSHPLILHSDRGSQFRSGDYQRYLAANGLVCSMS
                     AVGHCGDNAACEGFFGLLK--RERVYRMTYPTLDAARADVFEYIERFHNP-RMRRRVA
                     ----RQDQKFSA"
                     /note="IS3_IS3_ORF1      e-value= 2.4e-72 complete sequence
                     hit coverage= 100%, length is 278aa with 10 gaps, 0
                     stops, frame= Minus3"
     CDS             complement(3123435..3122639)
                     /colour="255 0 0"
                     /evidence=predicted
                     /translation="RCRDEFPVRLMCRCLRVSASGYYDWSKRLPSARERDNQRLLGRI
                     RELHEDSRGTLGAGRMHEDLAEEGETASLNRVARLMATDGLQGWPRPKRRGQRGK--P
                     ALTPPgvrNLLERDFTALE--PETRWVTDITELKTRQGKLYLCIVLDLFDQRVVGWSM
                     HHRQDRQMVIRAVQMAVWQ----RQGSHPLILHSDRGSQFRSGDYQRYLAANGLVCSM
```

```
                     SAVGHCGDNAACEGFFGLLKRERVYRMTYPTLDAARADVFEYIERFHNpRMRRRVA"
                     /note="IS3_IS3_ORF2      e-value= 2.2e-69 fragment hit
                     coverage= 93.75%, length is 266aa with 8 gaps, 0 stops,
                     frame= Minus3"
     CDS             complement(3123435..3122618)
                     /colour="255 0 0"
                     /evidence=predicted
                     /translation="RCRDEFPVRLMCRCLRVSASGYYDWSKRLPSARERDNQRLLGRI
                     RELHEDSRGTLGAGRMHEDLAEEGETASLNRVARLMATDGLQGWPRPKRRGQRGK--P
                     ALTPPgvrNLLERDFTALE--PETRWVTDITELKTRQGKLYLCIVLDLFDQRVVGWSM
                     HHRQDRQMVIRAVQMAVWQ----RQGSHPLILHSDRGSQFRSGDYQRYLAANGLVCSM
                     SAVGHCGDNAACEGFFGLLKRERVYRMTYPTLDAARADVFEYIERFHNpRMRRRVA--
                     ---------RQDQKFS"
                     /note="IS3_IS3_ORF2      e-value= 2.6e-66 complete sequence
                     hit coverage= 100%, length is 273aa with 19 gaps, 0
                     stops, frame= Minus3"
     CDS             complement(3123414..3122660)
                     /colour="255 0 0"
                     /evidence=predicted
                     /translation="VRLMCRCLRVS---ASGYYDWSKRLPSARERDNQRLLGRIRELH
                     EDSRG-----TLGAGRMHEDLAEEGETASLNRVARLMATDGLQGW--PRPKRRGQRGK
                     PALTPPgvrNLLERDFTALEPETRWVTDITELKTRQGKLYLCIVLDLFDQRVVGWSMH
                     HRQDRQMVIRAVQMAVWQRQGS-----HPLILHSDRGSQFRSGDYQRYLAANGLVCSM
                     SAVGHCGDNAACEGFFGLLKRERVYRMTYPTLDAARADVFEYIERFHNP"
                     /note="IS3_IS51_ORF2     e-value= 1.5e-73 fragment hit
                     coverage= 83.28%, length is 252aa with 15 gaps, 0 stops,
                     frame= Minus3"
     CDS             complement(3123384..3122663)
                     /colour="255 0 0"
                     /evidence=predicted
                     /translation="SASGYYDWSKRLPSARERDnQRLLGRIRELHEDSRGTLGAGRMH
                     EDLAEEGETASLNRVARLMATDGLQGWPRPKR--RGQRGKPALTPPGVrNLLERDFTA
                     LEPETRWVTDITELKT--RQGKLYLCIVLDLFDQRVVGWSMHHRQDRQMVIRAVQMAV
                     WQ-RQGSHPLILHSDRGSQFRSGDYQRYLAANGLVCSMSAVGHCGDNAACEGFFGLLK
                     RERVYRMTYPTLD--AARADVFEYIERFHN"
                     /note="IS3_IS150_ORF1    e-value= 2.4e-37 fragment hit
                     coverage= 80.39%, length is 241aa with 7 gaps, 0 stops,
                     frame= Minus3"
     CDS             complement(2403027..2402087)
                     /colour="255 0 0"
                     /evidence=predicted
                     /translation="NARLTVHSRALLIRRILHEGLRPEEAAQACGVSVRTAYKWLARF
                     RQFGAPGLENRSSRPHQTPHATPAPVIEQIKEHR-RKRQTYLTISKALG---------
                     VGHSTISRLMRAHGLNRLCRLEPPK-----EVIR--------YEYDQPGGLLHLDIKK
                     -----LGNFQRPGHRTDAkRRGNAAGGGWGYVHVAIDDHSRVAFSSVHPNEQAETACQ
                     ALLGALQYYASLGIT-FKRILTDNGACYRST----AFAKLLKSLGIKHIRTKPYTPRT
                     NGKAERFIQTSLREWAYARE---YASSDQRNSVLTQWLHHYNWHRPHM--GIGGQPPI
                     SRVLLNNVVGLHSXSTNK"
                     /note="IS481 e-value= 4.3e-95 complete sequence hit
                     coverage= 100%, length is 314aa with 38 gaps, 1 stops,
                     frame= Minus3"
     CDS             complement(2403027..2402087)
                     /colour="255 0 0"
                     /evidence=predicted
                     /translation="NARLTVHSRALLIRRILHEGLRPEEAAQACGVSVRTAYKWLARF
                     RQFGAPGLENRSSRPHQTPHATPAPVIEQIKEHR-RKRQTYLTISKALG---------
                     VGHSTISRLMRAHGLNRLCRLEPPK-----EVIR--------YEYDQPGGLLHLDIKK
                     -----LGNFQRPGHRTDAkRRGNAAGGGWGYVHVAIDDHSRVAFSSVHPNEQAETACQ
                     ALLGALQYYASLGIT-FKRILTDNGACYRST----AFAKLLKSLGIKHIRTKPYTPRT
                     NGKAERFIQTSLREWAYARE---YASSDQRNSVLTQWLHHYNWHRPHM--GIGGQPPI
```

```
                     SRVLLNNVVGLHSXSTNK"
                     /note="IS481 e-value= 1.1e-95 complete sequence hit
                     coverage= 100%, length is 314aa with 38 gaps, 1 stops,
                     frame= Minus3"
     CDS             complement(2402406..2402141)
                     /colour="255 0 0"
                     /evidence=predicted
                     /translation="RILTDNGACYRSTAFAKLLKSLGIKHIRTKPYTPRTNGKAERFI
                     QTSLREWAY--AREYASSDQRNSVLTQWLHHYNWHRPHMGIGGQPPI"
                     /note="IS3_IS150_ORF2    e-value= 1.8e-10 fragment hit
                     coverage= 23.39%, length is 89aa with 2 gaps, 0 stops,
                     frame= Minus3"
     CDS             complement(2402211..2402144)
                     /colour="255 0 0"
                     /evidence=predicted
                     /translation="VLTQWLHHYNWHRPHMGIGGQPP"
                     /note="IS3_IS407_ORF2    e-value= 4.1e-07 fragment hit
                     coverage= 6.69%, length is 23aa with 0 gaps, 0 stops,
                     frame= Minus3"
     CDS             complement(1723680..1722572)
                     /colour="255 0 0"
                     /evidence=predicted
                     /translation="VQDRGGPT------DRXVRPSGSGGCRATgRVDPXSLRLEAATR
                     QRRCWPACRAGPERGS-APPQGXATQGHXRARHPKKSRRVL---CKGVRAKYAFMKQH
                     ADEFGLAAMCRMLGVHRSGYYAWLKEPASARDKDDQRLLGLIKHSWLESGSVYGHRKV
                     TTDLR---ELGETCSRHRVARLMKSEGLRAMVGYGRRPRPLSGPVGSVAKNVLARGFK
                     VSEPNRAWVTDITYIRTY-DGFLYLAVVLDLFSRQVVGWATRPTQHTDLVLQALLAAV
                     WRRKPSPGLLLHS-----DQGTQFTSEDWQSFLREHDIVCSMSRRGNCHDNAAMESFF
                     QLLKRERIK--RRIYSNHDEARADVFQYIEmFYNPKRRHSSNDGLSPVEFEKQYALN"
                     /note="IS3_IS150_ORF2    e-value= 1.2e-45 complete sequence
                     hit coverage= 100%, length is 370aa with 21 gaps, 4
                     stops, frame= Minus3"
     CDS             complement(1723617..1722548)
                     /colour="255 0 0"
                     /evidence=predicted
                     /translation="--ATGRVDPXSLRLEAATRQ---RRCWPACRAG-PERGSAPPQG
                     XATQGHXRARhPKKSRRVLCKGVRAKYAFMKQHAdEFGLAAMCRMLGVHRSGYYAWLK
                     E-PASARDKddQRLLGLIkHSWLEsgsvYGHRKVTTDLRELGETCSRHRVARLMKSEG
                     LRAmvgygRRPR-PLSGPVGSVAKnvLARgfkVSEPNRAWVTDITYIRTYDG--FLYL
                     AVVlDLFSRQVVGWATRPTQHTDLVLQALLAAVWRRkPSPgLLLHSDQGTQFTSEDWQ
                     SFLREHDIVCSMSRRGNCHDNAAMESFFQLLKRERIKRRIYSNHDEARADVFQYIEMf
                     YNPKRRHSSNDGLSPVEFEKQYALNGXRLSRKP"
                     /note="IS3_IS407_ORF2    e-value= 2.1e-06 complete sequence
                     hit coverage= 100%, length is 357aa with 10 gaps, 4
                     stops, frame= Minus3"
     CDS             complement(1723479..1722575)
                     /colour="255 0 0"
                     /evidence=predicted
                     /translation="ARHPKKSRRVLCKgVRAKYAFMKQHADEFGLAAMCRMLGVHRSg
                     YYAWLKePASARDKDDQRLLGLIKHSWLESGSVYGHRKVTTDLRELGETCSRHRVARL
                     MKSEGLRAMVGYGR--RPRPLSGPVGSVAKNVLARGFKVSEPNRAWVTDITYIRT--Y
                     DGFLYLAVVLDLFSRQVVGWATRPTQHTDLVLQALLAAVWR-RKPSPGLLLHSDQGTQ
                     FTSEDWQSFLREHDIVCSMSRRGNCHDNAAMESFFQLLKRERIKRRIYSNHD--EARA
                     DVFQYIEMFYNPKRRHSSNDGLSPVEFEKQYAL"
                     /note="IS3_IS150_ORF1    e-value= 1.4e-57 complete sequence
                     hit coverage= 100%, length is 302aa with 7 gaps, 0 stops,
                     frame= Minus3"
     CDS             complement(1723455..1722563)
                     /colour="255 0 0"
                     /evidence=predicted
```

/translation="RVLCKGVRAKYAFMkqhADEFGLAAMCRMLGVHRSGYYAWLK--
----EPASARDKDDQRLLGLIKHSWLESGSvYGHRKVTTDLRELGETCSR-----HRV
ARLMkseGLRAMVgYGRRPRPLSGP---VGSVAknvlargfkVSEPNRAWVTDITYIR
TYDG-FLYLAVVLDLFSRQVVGWATRPTQ--HTDLVLQALLAAVWRR----KPSPGLL
LH-SDQGTQFTSEDWQSFLREHDIVCSMSRRGNCHDNAAMESFFQLLKRERIkrRIYS
NHDEARA--DVFQYIEMfYNPKRRHSSNDGLSPVEFEKQYALNGXR"
/note="IS3_IS2_ORF2   e-value= 1.4e-07 complete sequence
hit coverage= 100%, length is 298aa with 24 gaps, 1
stops, frame= Minus3"

CDS   complement(1723452..1722575)
/colour="255 0 0"
/evidence=predicted
/translation="VLCKgVRAKYAFMKQHADEFGLAAMCRMLGVHRSgYYAWLKePA
SARDKDDQRLLGLIKHSWLESGSVYGHRKVTTDLRELGETCSRHRVARLMKSEGLRAM
VGYGR--RPRPLSGPVGSVAKNVLARGFKVSEPNRAWVTDITYIRT--YDGFLYLAVV
LDLFSRQVVGWATRPTQHTDLVLQALLAAVWR-RKPSPGLLLHSDQGTQFTSEDWQSF
LREHDIVCSMSRRGNCHDNAAMESFFQLLKRERIKRRIYSNHD--EARADVFQYIEMF
YNPKRRHSSNDGLSPVEFEKQYAL"
/note="IS3_IS150_ORF1   e-value= 5.1e-57 fragment hit
coverage= 97.06%, length is 293aa with 7 gaps, 0 stops,
frame= Minus3"

CDS   complement(1723443..1722536)
/colour="255 0 0"
/evidence=predicted
/translation="KGVRAKYAFMKQHAD--EFGLAAMCRMLGVH---RSGYYAWLKE
PASARDKDDQRLLGLIKHSWLESGS-----VYGHRKVTTDLRELGETCSRHRVARLMK
SEGLRAMVGYGRRPRPLSGPVGSVAKNVLARGFKVSEPNRAWVTDITYIRTYDGFLYL
AVVLDLFSRQVVGWATRPTQHTDLVLQALLAAVWRRKPS-----PGLLLHSDQGTQFT
SEDWQSFLREHDIVCSMSRRGNCHDNAAMESFFQLLKRERIKRRIYSNHDEARADVFQ
YIEMFYNPKRRHSSNDGLSPVEFEKQYALNGxRLSRKPWAIH"
/note="IS3_IS51_ORF2   e-value= 2.7e-134 complete
sequence
hit coverage= 100%, length is 303aa with 15 gaps, 0
stops, frame= Minus3"

CDS   complement(1723425..1722581)
/colour="255 0 0"
/evidence=predicted
/translation="YAFMKQHADEFGLAAMCRMLGVHRSGYYAWL-----KEPASARD
KDDQRLLGLIKHSWLESGSVYGHRKVTTDLRELGETCSRHRVARLMKSEGLRAMVGYG
RRPRPLSGPVGSVAKNVLARGFKVSEPNRAWVTDITYIRTYDGFLYLAVVLDLFSRQV
VGWATRPTQHTDLV-LQALLAAVWRRKPSP----GLLLHSDQGTQFTSEDWQSFLREH
DIVCSMSRRGNCHDNAAMESFFQLLKRERIKR----RIYSNHDEARADVFQYIEMFYN
PKRRHSSNDGLSPVEFEKQY"
/note="IS3_IS51_ORF1   e-value= 3.9e-110 fragment hit
coverage= 96.42%, length is 282aa with 14 gaps, 0 stops,
frame= Minus3"

CDS   complement(1723425..1722548)
/colour="255 0 0"
/evidence=predicted
/translation="YAFMKQHADEFGLAAMCRMLGVHRSGYYAWL-----KEPASARD
KDDQRLLGLIKHSWLESGSVYGHRKVTTDLRELGETCSRHRVARLMKSEGLRAMVGYG
RRPRPLSGPVGSVAKNVLARGFKVSEPNRAWVTDITYIRTYDGFLYLAVVLDLFSRQV
VGWATRPTQHTDLV-LQALLAAVWRRKPSP----GLLLHSDQGTQFTSEDWQSFLREH
DIVCSMSRRGNCHDNAAMESFFQLLKRERIKR----RIYSNHDEARADVFQYIEMFYN
PKRRHSSNDGLSPVEFEKQYALNGXRLSRKP"
/note="IS3_IS51_ORF1   e-value= 3.7e-108 complete
sequence
hit coverage= 100%, length is 293aa with 14 gaps, 1
stops, frame= Minus3"

CDS   complement(1723422..1722578)

```
                     /colour="255 0 0"
                     /evidence=predicted
                     /translation="AFMKQHADEFGLAAMCRMLGVHRSGYYAWLKE--PASARDKDDQ
                     RLLGLIKHSWLESGSVYGHRKVTTDLR-ELGETCSRHRVARLMKSEGLRAmVGYG-RR
                     PRPLSgPVGSVAKNVLARGFKVSEPNRAWVTDITYIRTYDGFLYLAVVLDLFSRQVVG
                     WATRPTQHTDLVLQALLAAVWRRKPSPGLLLHSDQGTQFTSEDWQSFLREHDIVCSMS
                     RRGNCHDNAAMESFFQLLK--RERIKRRIYSNHDEARADVFQYIEMFYNPKRRHSSND
                     GLSPVEFEKQYA"
                     /note="IS3_IS3_ORF1      e-value= 1.9e-94 complete sequence
                     hit coverage= 100%, length is 282aa with 6 gaps, 0 stops,
                     frame= Minus3"
     CDS             complement(1723422..1722578)
                     /colour="255 0 0"
                     /evidence=predicted
                     /translation="AFMKQHADEFGLAAMCRMLGVHRSGYYAWLKE--PASARDKDDQ
                     RLLGLIKHSWLESGSVYGHRKVTTDLR-ELGETCSRHRVARLMKSEGLRAmVGYG-RR
                     PRPLSgPVGSVAKNVLARGFKVSEPNRAWVTDITYIRTYDGFLYLAVVLDLFSRQVVG
                     WATRPTQHTDLVLQALLAAVWRRKPSPGLLLHSDQGTQFTSEDWQSFLREHDIVCSMS
                     RRGNCHDNAAMESFFQLLK--RERIKRRIYSNHDEARADVFQYIEMFYNPKRRHSSND
                     GLSPVEFEKQYA"
                     /note="IS3_IS3_ORF1      e-value= 4.9e-95 complete sequence
                     hit coverage= 100%, length is 282aa with 6 gaps, 0 stops,
                     frame= Minus3"
     CDS             complement(1723410..1722566)
                     /colour="255 0 0"
                     /evidence=predicted
                     /translation="QHADEFGLAAMCRMLGVHRSGYYAWLKEPASARDKDDQRLLGLI
                     KHSWLESGSVYGHRKVTTDLRELGETCSRHRVARLMKSEGLRAMvGYGRRPRPLSGPV
                     GSVAK-NVLARGFKVSE--PNRAWVTDITYIRTYDGFLYLAVVLDLFSRQVVGWATRP
                     TQHTDLVLQALLAAVWR----RKPSPGLLLHSDQGTQFTSEDWQSFLREHDIVCSMSR
                     RGNCHDNAAMESFFQLLKRERIKRRIYSNHDEARADVFQYIEMFYNPKRRHSSNDGLS
                     PVEFEKQYALNGX"
                     /note="IS3_IS3_ORF2      e-value= 7.1e-102 complete
sequence
                     hit coverage= 100%, length is 282aa with 7 gaps, 1 stops,
                     frame= Minus3"
     CDS             complement(1723410..1722566)
                     /colour="255 0 0"
                     /evidence=predicted
                     /translation="QHADEFGLAAMCRMLGVHRSGYYAWLKEPASARDKDDQRLLGLI
                     KHSWLESGSVYGHRKVTTDLRELGETCSRHRVARLMKSEGLRAMvGYGRRPRPLSGPV
                     GSVAK-NVLARGFKVSE--PNRAWVTDITYIRTYDGFLYLAVVLDLFSRQVVGWATRP
                     TQHTDLVLQALLAAVWR----RKPSPGLLLHSDQGTQFTSEDWQSFLREHDIVCSMSR
                     RGNCHDNAAMESFFQLLKRERIKRRIYSNHDEARADVFQYIEMFYNPKRRHSSNDGLS
                     PVEFEKQYALNGX"
                     /note="IS3_IS3_ORF2      e-value= 2e-102 complete sequence
hit
                     coverage= 100%, length is 282aa with 7 gaps, 1 stops,
                     frame= Minus3"
     CDS             complement(1723398..1722572)
                     /colour="255 0 0"
                     /evidence=predicted
                     /translation="EFGLAAMCRMLGVHRSGYYAWLKEPASARDKDDQRLLGLIKHSW
                     LESGSVYGHRKVTTDLR---ELGETCSRHRVARLMKSEGLRAMVGYGRRPRPLSGPVG
                     SVAKNVLARGFKVSEPNRAWVTDITYIRTY-DGFLYLAVVLDLFSRQVVGWATRPTQH
                     TDLVLQALLAAVWRRKPSPGLLLHS-----DQGTQFTSEDWQSFLREHDIVCSMSRRG
                     NCHDNAAMESFFQLLKRERIK--RRIYSNHDEARADVFQYIEmFYNPKRRHSSNDGLS
                     PVEFEKQYALN"
                     /note="IS3_IS150_ORF2    e-value= 1.9e-74 fragment hit
                     coverage= 73.52%, length is 276aa with 11 gaps, 0 stops,
```

```
                     frame= Minus3"
     CDS             complement(1723395..1722578)
                     /colour="255 0 0"
                     /evidence=predicted
                     /translation="FGLAAMCRMLGVH---RSGYYAWLKEPASARDKDDQRLLGLIKH
                     SWLESGS-----VYGHRKVTTDLRELGETCSRHRVARLMKSEGLRAMVGYGRRPRPLS
                     GPVGSVAKNVLARGFKVSEPNRAWVTDITYIRTYDGFLYLAVVLDLFSRQVVGWATRP
                     TQHTDLVLQALLAAVWRRKPS-----PGLLLHSDQGTQFTSEDWQSFLREHDIVCSMS
                     RRGNCHDNAAMESFFQLLKRERIKRRIYSNHDEARADVFQYIEMFYNPKRRHSSNDGL
                     SPVEFEKQYA"
                     /note="IS3_IS51_ORF2     e-value= 7.1e-152 fragment hit
                     coverage= 90.22%, length is 273aa with 13 gaps, 0 stops,
                     frame= Minus3"
     CDS             complement(526827..525914)
                     /colour="255 0 0"
                     /evidence=predicted
                     /translation="NARLTVHSRALLIRRILHEGLRPEEAAQACGVSVRTAYKWLARF
                     RQFGAPGLENRSSRPHQTPHATPAPVIEQIKEHR-RKRQTYLTISKALG---------
                     VGHSTISRLMRAHGLNRLCRLEPPK-----EVIR--------YEYDQPGGLLHLDIKK
                     -----LGNFQRPGHRTDAkRRGNAAGGGWGYVHVAIDDHSRVAFSSVHPNEQAETACQ
                     ALLGALQYYASLGIT-FKRILTDNGACYRST----AFAKLLKSLGIKHIRTKPYTPRT
                     NGKAERFIQTSLREWAYARE---YASSDQRNSVLTQWLHHYNWHRPHM--GIGGQPPI
                     SRVLLNNVV"
                     /note="IS481  e-value= 6.1e-93 fragment hit coverage=
                     97.44%, length is 305aa with 38 gaps, 0 stops, frame=
                     Minus3"
     CDS             complement(526827..525887)
                     /colour="255 0 0"
                     /evidence=predicted
                     /translation="NARLTVHSRALLIRRILHEGLRPEEAAQACGVSVRTAYKWLARF
                     RQFGAPGLENRSSRPHQTPHATPAPVIEQIKEHR-RKRQTYLTISKALG---------
                     VGHSTISRLMRAHGLNRLCRLEPPK-----EVIR--------YEYDQPGGLLHLDIKK
                     -----LGNFQRPGHRTDAkRRGNAAGGGWGYVHVAIDDHSRVAFSSVHPNEQAETACQ
                     ALLGALQYYASLGIT-FKRILTDNGACYRST----AFAKLLKSLGIKHIRTKPYTPRT
                     NGKAERFIQTSLREWAYARE---YASSDQRNSVLTQWLHHYNWHRPHM--GIGGQPPI
                     SRVLLNNVVGLHNXQSTL"
                     /note="IS481  e-value= 1.2e-93 complete sequence hit
                     coverage= 100%, length is 314aa with 38 gaps, 1 stops,
                     frame= Minus3"
     CDS             complement(526206..525941)
                     /colour="255 0 0"
                     /evidence=predicted
                     /translation="RILTDNGACYRSTAFAKLLKSLGIKHIRTKPYTPRTNGKAERFI
                     QTSLREWAY--AREYASSDQRNSVLTQWLHHYNWHRPHMGIGGQPPI"
                     /note="IS3_IS150_ORF2    e-value= 1.8e-10 fragment hit
                     coverage= 23.39%, length is 89aa with 2 gaps, 0 stops,
                     frame= Minus3"
     CDS             complement(526011..525944)
                     /colour="255 0 0"
                     /evidence=predicted
                     /translation="VLTQWLHHYNWHRPHMGIGGQPP"
                     /note="IS3_IS407_ORF2    e-value= 4.1e-07 fragment hit
                     coverage= 6.69%, length is 23aa with 0 gaps, 0 stops,
                     frame= Minus3"

//
```
