# Supplementary material for: TnpPred: A Web Service for the Robust Prediction of Prokaryotic Transposases
Source: Comp Funct Genomics. 2012 Nov 18;2012:678761. doi: 10.1155/2012/678761 (PMC3506888; doi:10.1155/2012/678761)
Supplement: Supplementary file 4 [file 678761.f4.pdf]

Supplementary file 4
Acaryochloris marina MBIC11017 uid58167 full report of TnpPred predictions.
NC_009925.gbk Chromosome

1.
CDS YP_001514421.1 response regulator intersects 24.63%(33aa) with IS5_IS427 e-value= 4.4e-06 complete sequence hit coverage= 100%, between model(296 aa) positions 1; 296 length is 280aa with 33 gaps, 7 stops, absolute frame= Plus1 at 19129..19968 in the opposite strand
misc_feature no id Signal receiver domain; originally thought to be unique to bacteria (CheY, OmpR, NtrC, and PhoB), now recently identified in eukaroytes ETR1 Arabidopsis thaliana; this domain receives the signal from the sensor partner in a two-component systems; Region: REC; cd00156 intersects 22.13%(27aa) with IS5_IS427 e-value= 4.4e-06 complete sequence hit coverage= 100%, between model(296 aa) positions 1; 296 length is 280aa with 33 gaps, 7 stops, absolute frame= Plus1 at 19129..19968 in the opposite strand
misc_feature no id Response regulator receiver domain; Region: Response_reg; pfam00072 intersects 23.33%(28aa) with IS5_IS427 e-value= 4.4e-06 complete sequence hit coverage= 100%, between model(296 aa) positions 1; 296 length is 280aa with 33 gaps, 7 stops, absolute frame= Plus1 at 19129..19968 in the opposite strand
CDS YP_001514422.1 IS4 family transposase intersects 56.55%(151aa) with IS5_IS427 e-value= 4.4e-06 complete sequence hit coverage= 100%, between model(296 aa) positions 1; 296 length is 280aa with 33 gaps, 7 stops, absolute frame= Plus1 at 19129..19968 in the same strand
misc_feature no id DDE superfamily endonuclease; Region: DDE_4; cl15789 intersects 100%(75aa) with IS5_IS427 e-value= 4.4e-06 complete sequence hit coverage= 100%, between model(296 aa) positions 1; 296 length is 280aa with 33 gaps, 7 stops, absolute frame= Plus1 at 19129..19968 in the same strand
misc_feature no id Transposase DDE domain; Region: DDE_Tnp_1; pfam01609 intersects 30.32%(47aa) with IS5_IS427 e-value= 4.4e-06 complete sequence hit coverage= 100%, between model(296 aa) positions 1; 296 length is 280aa with 33 gaps, 7 stops, absolute frame= Plus1 at 19129..19968 in the same strand
CDS YP_001514422.1 IS4 family transposase intersects 100%(267aa) with IS5_IS1031 e-value= 1.9e-108 complete sequence hit coverage= 100%, between model(295 aa) positions 1; 295 length is 284aa with 14 gaps, 2 stops, absolute frame= Plus1 at 19486..20337 in the same strand
misc_feature no id DDE superfamily endonuclease; Region: DDE_4; cl15789 intersects 100%(75aa) with IS5_IS1031 e-value= 1.9e-108 complete sequence hit coverage= 100%, between model(295 aa) positions 1; 295 length is 284aa with 14 gaps, 2 stops, absolute frame= Plus1 at 19486..20337 in the same strand
misc_feature no id Transposase DDE domain; Region: DDE_Tnp_1; pfam01609 intersects 100%(155aa) with IS5_IS1031 e-value= 1.9e-108 complete sequence hit coverage= 100%, between model(295 aa) positions 1; 295 length is 284aa with 14 gaps, 2 stops, absolute frame= Plus1 at 19486..20337 in the same strand
misc_feature no id DDE superfamily endonuclease; Region: DDE_4; cl15789 intersects 100%(83aa) with IS5_IS1031 e-value= 1.9e-108 complete sequence hit coverage= 100%, between model(295 aa) positions 1; 295 length is 284aa with 14 gaps, 2 stops, absolute frame= Plus1 at 19486..20337 in the same strand
CDS YP_001514422.1 IS4 family transposase intersects 98.50%(263aa) with IS5_IS1031 e-value= 1.3e-110 fragment hit coverage= 93.56%, between model(295 aa) positions 20; 295 length is 270aa with 9 gaps, 1 stops, absolute frame= Plus1 at 19528..20337 in the same strand
misc_feature no id DDE superfamily endonuclease; Region: DDE_4; cl15789 intersects 100%(75aa) with IS5_IS1031 e-value= 1.3e-110 fragment hit coverage= 93.56%, between model(295 aa) positions 20; 295 length is 270aa with 9 gaps, 1 stops, absolute frame= Plus1 at 19528..20337 in the same strand
misc_feature no id Transposase DDE domain; Region: DDE_Tnp_1; pfam01609 intersects 100%(155aa) with IS5_IS1031 e-value= 1.3e-110 fragment hit coverage= 93.56%, between model(295 aa) positions 20; 295 length is 270aa with 9 gaps, 1 stops, absolute frame= Plus1 at 19528..20337 in the same strand
misc_feature no id DDE superfamily endonuclease; Region: DDE_4; cl15789 intersects 100%(83aa) with IS5_IS1031 e-value= 1.3e-110 fragment hit coverage= 93.56%, between model(295 aa) positions 20; 295 length is 270aa with 9 gaps, 1 stops, absolute frame= Plus1 at 19528..20337 in the same strand
CDS YP_001514422.1 IS4 family transposase intersects 29.96%(80aa) with IS5_IS427 e-value= 3.3e-18 fragment hit coverage= 27.70%, between model(296 aa) positions 125; 206 length is 80aa with 2 gaps, 0 stops, absolute frame= Plus1 at 19537..19776 in the same strand
misc_feature no id DDE superfamily endonuclease; Region: DDE_4; cl15789 intersects 100%(75aa) with IS5_IS427 e-value= 3.3e-18 fragment hit coverage= 27.70%, between model(296 aa) positions 125; 206 length is 80aa with 2 gaps, 0 stops, absolute frame= Plus1 at 19537..19776 in the same strand
Class A

2.
CDS YP_001514462.1 IS4 family transposase intersects 99.35%(308aa) with IS5_IS903 e-value= 1.7e-97 complete sequence hit coverage= 100%, between model(347 aa) positions 1; 347 length is 314aa with 37 gaps, 1 stops, absolute frame= Plus1 at 57724..58665 in the same strand
misc_feature no id Transposase DDE domain; Region: DDE_Tnp_1_5; pfam13737 intersects 100%(112aa) with IS5_IS903 e-value= 1.7e-97 complete sequence hit coverage= 100%, between model(347 aa) positions 1; 347 length is 314aa with 37 gaps, 1 stops, absolute frame= Plus1 at 57724..58665 in the same strand
misc_feature no id Transposase DDE domain; Region: DDE_Tnp_1; pfam01609 intersects 100%(186aa) with IS5_IS903 e-value= 1.7e-97 complete sequence hit coverage= 100%, between model(347 aa) positions 1; 347 length is 314aa with 37 gaps, 1 stops, absolute frame= Plus1 at 57724..58665 in the same strand
Class A

3.
CDS YP_001514463.1 transposase intersects 81.70%(83aa) with IS3_IS150_ORF2 e-value= 1.7e-22 complete sequence hit coverage= 100%, between model(389 aa) positions 1; 389 length is 354aa with 39 gaps, 5 stops, absolute frame= Plus2 at 59159..60220 in the same strand
misc_feature no id Helix-turn-helix domains; Region: HTH; cl00088 intersects 79.11%(59aa) with IS3_IS150_ORF2 e-value= 1.7e-22 complete sequence hit coverage= 100%, between model(389 aa) positions 1; 389 length is 354aa with 39 gaps, 5 stops, absolute frame= Plus2 at 59159..60220 in the same strand
CDS YP_001514464.1 integrase catalytic subunit intersects 95.45%(273aa) with IS3_IS150_ORF2 e-value= 1.7e-22 complete sequence hit coverage= 100%, between model(389 aa) positions 1; 389 length is 354aa with 39 gaps, 5 stops, absolute frame= Plus2 at 59159..60220 in the same strand
misc_feature no id putative transposase OrfB; Reviewed; Region: PHA02517 intersects 99.21%(252aa) with IS3_IS150_ORF2 e-value= 1.7e-22 complete sequence hit coverage= 100%, between model(389 aa) positions 1; 389 length is 354aa with 39 gaps, 5 stops, absolute frame= Plus2 at 59159..60220 in the same strand
misc_feature no id HTH-like domain; Region: HTH_21; pfam13276 intersects 100%(42aa) with IS3_IS150_ORF2 e-value= 1.7e-22 complete sequence hit coverage= 100%, between model(389 aa) positions 1; 389 length is 354aa with 39 gaps, 5 stops, absolute frame= Plus2 at 59159..60220 in the same strand
misc_feature no id Integrase core domain; Region: rve; cl01316 intersects 100%(110aa) with IS3_IS150_ORF2 e-value= 1.7e-22 complete sequence hit coverage= 100%, between model(389 aa) positions 1; 389 length is 354aa with 39 gaps, 5 stops, absolute frame= Plus2 at 59159..60220 in the same strand
misc_feature no id Integrase core domain; Region: rve_3; cl15866 intersects 100%(68aa) with IS3_IS150_ORF2 e-value= 1.7e-22 complete sequence hit coverage= 100%, between model(389 aa) positions 1; 389 length is 354aa with 39 gaps, 5 stops, absolute frame= Plus2 at 59159..60220 in the same strand
CDS YP_001514463.1 transposase intersects 68.95%(70aa) with IS3_IS407_ORF2 e-value= 6.3e-13 complete sequence hit coverage= 100%, between model(344 aa) positions 1; 344 length is 349aa with 10 gaps, 4 stops, absolute frame= Plus2 at 59198..60244 in the same strand
misc_feature no id Helix-turn-helix domains; Region: HTH; cl00088 intersects 61.78%(46aa) with IS3_IS407_ORF2 e-value= 6.3e-13 complete sequence hit coverage= 100%, between model(344 aa) positions 1; 344 length is 349aa with 10 gaps, 4 stops, absolute frame= Plus2 at 59198..60244 in the same strand
CDS YP_001514464.1 integrase catalytic subunit intersects 98.25%(281aa) with IS3_IS407_ORF2 e-value= 6.3e-13 complete sequence hit coverage= 100%, between model(344 aa) positions 1; 344 length is 349aa with 10 gaps, 4 stops, absolute frame= Plus2 at 59198..60244 in the same strand
misc_feature no id putative transposase OrfB; Reviewed; Region: PHA02517 intersects 100%(254aa) with IS3_IS407_ORF2 e-value= 6.3e-13 complete sequence hit coverage= 100%, between model(344 aa) positions 1; 344 length is 349aa with 10 gaps, 4 stops, absolute frame= Plus2 at 59198..60244 in the same strand
misc_feature no id HTH-like domain; Region: HTH_21; pfam13276 intersects 100%(42aa) with IS3_IS407_ORF2 e-value= 6.3e-13 complete sequence hit coverage= 100%, between model(344 aa) positions 1; 344 length is 349aa with 10 gaps, 4 stops, absolute frame= Plus2 at 59198..60244 in the same strand
misc_feature no id Integrase core domain; Region: rve; cl01316 intersects 100%(110aa) with IS3_IS407_ORF2 e-value= 6.3e-13 complete sequence hit coverage= 100%, between model(344 aa) positions 1; 344 length is 349aa with 10 gaps, 4 stops, absolute frame= Plus2 at 59198..60244 in the same strand
misc_feature no id Integrase core domain; Region: rve_3; cl15866 intersects 100%(68aa) with IS3_IS407_ORF2 e-value= 6.3e-13 complete sequence hit coverage= 100%, between model(344 aa) positions 1; 344 length is 349aa with 10 gaps, 4 stops, absolute frame= Plus2 at 59198..60244 in the same strand

CDS YP_001514463.1 transposase intersects 24.84%(25aa) with IS3_IS150_ORF1 e-value= 4.3e-49 complete sequence hit coverage= 100%, between model(306 aa) positions 1; 306 length is 295aa with 14 gaps, 0 stops, absolute frame= Plus2 at 59333..60217 in the same strand
misc_feature no id Helix-turn-helix domains; Region: HTH; cl00088 intersects 1.78%(1aa) with IS3_IS150_ORF1 e-value= 4.3e-49 complete sequence hit coverage= 100%, between model(306 aa) positions 1; 306 length is 295aa with 14 gaps, 0 stops, absolute frame= Plus2 at 59333..60217 in the same strand
CDS YP_001514464.1 integrase catalytic subunit intersects 95.10%(272aa) with IS3_IS150_ORF1 e-value= 4.3e-49 complete sequence hit coverage= 100%, between model(306 aa) positions 1; 306 length is 295aa with 14 gaps, 0 stops, absolute frame= Plus2 at 59333..60217 in the same strand
misc_feature no id putative transposase OrfB; Reviewed; Region: PHA02517 intersects 98.82%(251aa) with IS3_IS150_ORF1 e-value= 4.3e-49 complete sequence hit coverage= 100%, between model(306 aa) positions 1; 306 length is 295aa with 14 gaps, 0 stops, absolute frame= Plus2 at 59333..60217 in the same strand
misc_feature no id HTH-like domain; Region: HTH_21; pfam13276 intersects 100%(42aa) with IS3_IS150_ORF1 e-value= 4.3e-49 complete sequence hit coverage= 100%, between model(306 aa) positions 1; 306 length is 295aa with 14 gaps, 0 stops, absolute frame= Plus2 at 59333..60217 in the same strand
misc_feature no id Integrase core domain; Region: rve; cl01316 intersects 100%(110aa) with IS3_IS150_ORF1 e-value= 4.3e-49 complete sequence hit coverage= 100%, between model(306 aa) positions 1; 306 length is 295aa with 14 gaps, 0 stops, absolute frame= Plus2 at 59333..60217 in the same strand
misc_feature no id Integrase core domain; Region: rve_3; cl15866 intersects 100%(68aa) with IS3_IS150_ORF1 e-value= 4.3e-49 complete sequence hit coverage= 100%, between model(306 aa) positions 1; 306 length is 295aa with 14 gaps, 0 stops, absolute frame= Plus2 at 59333..60217 in the same strand
CDS YP_001514463.1 transposase intersects 13.07%(13aa) with IS3_IS51_ORF2 e-value= 1.6e-26 complete sequence hit coverage= 100%, between model(317 aa) positions 1; 317 length is 295aa with 27 gaps, 0 stops, absolute frame= Plus2 at 59369..60253 in the same strand
CDS YP_001514464.1 integrase catalytic subunit intersects 99.30%(284aa) with IS3_IS51_ORF2 e-value= 1.6e-26 complete sequence hit coverage= 100%, between model(317 aa) positions 1; 317 length is 295aa with 27 gaps, 0 stops, absolute frame= Plus2 at 59369..60253 in the same strand
misc_feature no id putative transposase OrfB; Reviewed; Region: PHA02517 intersects 100%(254aa) with IS3_IS51_ORF2 e-value= 1.6e-26 complete sequence hit coverage= 100%, between model(317 aa) positions 1; 317 length is 295aa with 27 gaps, 0 stops, absolute frame= Plus2 at 59369..60253 in the same strand
misc_feature no id HTH-like domain; Region: HTH_21; pfam13276 intersects 100%(42aa) with IS3_IS51_ORF2 e-value= 1.6e-26 complete sequence hit coverage= 100%, between model(317 aa) positions 1; 317 length is 295aa with 27 gaps, 0 stops, absolute frame= Plus2 at 59369..60253 in the same strand
misc_feature no id Integrase core domain; Region: rve; cl01316 intersects 100%(110aa) with IS3_IS51_ORF2 e-value= 1.6e-26 complete sequence hit coverage= 100%, between model(317 aa) positions 1; 317 length is 295aa with 27 gaps, 0 stops, absolute frame= Plus2 at 59369..60253 in the same strand
misc_feature no id Integrase core domain; Region: rve_3; cl15866 intersects 100%(68aa) with IS3_IS51_ORF2 e-value= 1.6e-26 complete sequence hit coverage= 100%, between model(317 aa) positions 1; 317 length is 295aa with 27 gaps, 0 stops, absolute frame= Plus2 at 59369..60253 in the same strand
CDS YP_001514463.1 transposase intersects 11.11%(11aa) with IS3_IS407_ORF2 e-value= 1.2e-17 fragment hit coverage= 25.29%, between model(344 aa) positions 60; 146 length is 85aa with 2 gaps, 0 stops, absolute frame= Plus2 at 59375..59629 in the same strand
CDS YP_001514464.1 integrase catalytic subunit intersects 26.57%(76aa) with IS3_IS407_ORF2 e-value= 1.2e-17 fragment hit coverage= 25.29%, between model(344 aa) positions 60; 146 length is 85aa with 2 gaps, 0 stops, absolute frame= Plus2 at 59375..59629 in the same strand
misc_feature no id putative transposase OrfB; Reviewed; Region: PHA02517 intersects 21.65%(55aa) with IS3_IS407_ORF2 e-value= 1.2e-17 fragment hit coverage= 25.29%, between model(344 aa) positions 60; 146 length is 85aa with 2 gaps, 0 stops, absolute frame= Plus2 at 59375..59629 in the same strand
misc_feature no id HTH-like domain; Region: HTH_21; pfam13276 intersects 66.67%(28aa) with IS3_IS407_ORF2 e-value= 1.2e-17 fragment hit coverage= 25.29%, between model(344 aa) positions 60; 146 length is 85aa with 2 gaps, 0 stops, absolute frame= Plus2 at 59375..59629 in the same strand
CDS YP_001514463.1 transposase intersects 10.13%(10aa) with IS3_IS2_ORF2 e-value= 2.2e-08 complete sequence hit coverage= 100%, between model(302 aa) positions 1; 302 length is 284aa with 31 gaps, 0 stops, absolute frame= Plus2 at 59378..60229 in the same strand
CDS YP_001514464.1 integrase catalytic subunit intersects 96.50%(276aa) with IS3_IS2_ORF2 e-value= 2.2e-08 complete sequence hit coverage= 100%, between model(302 aa) positions 1; 302 length is 284aa with 31 gaps, 0 stops, absolute frame= Plus2 at 59378..60229 in the same strand

misc_feature no id putative transposase OrfB; Reviewed; Region: PHA02517 intersects 100%(254aa) with IS3_IS2_ORF2 e-value= 2.2e-08 complete sequence hit coverage= 100%, between model(302 aa) positions 1; 302 length is 284aa with 31 gaps, 0 stops, absolute frame= Plus2 at 59378..60229 in the same strand
misc_feature no id HTH-like domain; Region: HTH_21; pfam13276 intersects 100%(42aa) with IS3_IS2_ORF2 e-value= 2.2e-08 complete sequence hit coverage= 100%, between model(302 aa) positions 1; 302 length is 284aa with 31 gaps, 0 stops, absolute frame= Plus2 at 59378..60229 in the same strand
misc_feature no id Integrase core domain; Region: rve; cl01316 intersects 100%(110aa) with IS3_IS2_ORF2 e-value= 2.2e-08 complete sequence hit coverage= 100%, between model(302 aa) positions 1; 302 length is 284aa with 31 gaps, 0 stops, absolute frame= Plus2 at 59378..60229 in the same strand
misc_feature no id Integrase core domain; Region: rve_3; cl15866 intersects 100%(68aa) with IS3_IS2_ORF2 e-value= 2.2e-08 complete sequence hit coverage= 100%, between model(302 aa) positions 1; 302 length is 284aa with 31 gaps, 0 stops, absolute frame= Plus2 at 59378..60229 in the same strand
CDS YP_001514463.1 transposase intersects 5.23%(5aa) with IS3_IS150_ORF2 e-value= 2.2e-47 fragment hit coverage= 76.09%, between model(389 aa) positions 94; 389 length is 276aa with 23 gaps, 0 stops, absolute frame= Plus2 at 59393..60220 in the same strand
CDS YP_001514464.1 integrase catalytic subunit intersects 95.45%(273aa) with IS3_IS150_ORF2 e-value= 2.2e-47 fragment hit coverage= 76.09%, between model(389 aa) positions 94; 389 length is 276aa with 23 gaps, 0 stops, absolute frame= Plus2 at 59393..60220 in the same strand
misc_feature no id putative transposase OrfB; Reviewed; Region: PHA02517 intersects 99.21%(252aa) with IS3_IS150_ORF2 e-value= 2.2e-47 fragment hit coverage= 76.09%, between model(389 aa) positions 94; 389 length is 276aa with 23 gaps, 0 stops, absolute frame= Plus2 at 59393..60220 in the same strand
misc_feature no id HTH-like domain; Region: HTH_21; pfam13276 intersects 100%(42aa) with IS3_IS150_ORF2 e-value= 2.2e-47 fragment hit coverage= 76.09%, between model(389 aa) positions 94; 389 length is 276aa with 23 gaps, 0 stops, absolute frame= Plus2 at 59393..60220 in the same strand
misc_feature no id Integrase core domain; Region: rve; cl01316 intersects 100%(110aa) with IS3_IS150_ORF2 e-value= 2.2e-47 fragment hit coverage= 76.09%, between model(389 aa) positions 94; 389 length is 276aa with 23 gaps, 0 stops, absolute frame= Plus2 at 59393..60220 in the same strand
misc_feature no id Integrase core domain; Region: rve_3; cl15866 intersects 100%(68aa) with IS3_IS150_ORF2 e-value= 2.2e-47 fragment hit coverage= 76.09%, between model(389 aa) positions 94; 389 length is 276aa with 23 gaps, 0 stops, absolute frame= Plus2 at 59393..60220 in the same strand
CDS YP_001514463.1 transposase intersects 4.25%(4aa) with IS3_IS51_ORF1 e-value= 6.3e-51 complete sequence hit coverage= 100%, between model(307 aa) positions 1; 307 length is 283aa with 25 gaps, 0 stops, absolute frame= Plus2 at 59396..60244 in the same strand
CDS YP_001514464.1 integrase catalytic subunit intersects 98.25%(281aa) with IS3_IS51_ORF1 e-value= 6.3e-51 complete sequence hit coverage= 100%, between model(307 aa) positions 1; 307 length is 283aa with 25 gaps, 0 stops, absolute frame= Plus2 at 59396..60244 in the same strand
misc_feature no id putative transposase OrfB; Reviewed; Region: PHA02517 intersects 100%(254aa) with IS3_IS51_ORF1 e-value= 6.3e-51 complete sequence hit coverage= 100%, between model(307 aa) positions 1; 307 length is 283aa with 25 gaps, 0 stops, absolute frame= Plus2 at 59396..60244 in the same strand
misc_feature no id HTH-like domain; Region: HTH_21; pfam13276 intersects 100%(42aa) with IS3_IS51_ORF1 e-value= 6.3e-51 complete sequence hit coverage= 100%, between model(307 aa) positions 1; 307 length is 283aa with 25 gaps, 0 stops, absolute frame= Plus2 at 59396..60244 in the same strand
misc_feature no id Integrase core domain; Region: rve; cl01316 intersects 100%(110aa) with IS3_IS51_ORF1 e-value= 6.3e-51 complete sequence hit coverage= 100%, between model(307 aa) positions 1; 307 length is 283aa with 25 gaps, 0 stops, absolute frame= Plus2 at 59396..60244 in the same strand
misc_feature no id Integrase core domain; Region: rve_3; cl15866 intersects 100%(68aa) with IS3_IS51_ORF1 e-value= 6.3e-51 complete sequence hit coverage= 100%, between model(307 aa) positions 1; 307 length is 283aa with 25 gaps, 0 stops, absolute frame= Plus2 at 59396..60244 in the same strand
CDS YP_001514463.1 transposase intersects 3.27%(3aa) with IS3_IS3_ORF1 e-value= 2.5e-54 complete sequence hit coverage= 100%, between model(286 aa) positions 1; 286 length is 272aa with 15 gaps, 0 stops, absolute frame= Plus2 at 59399..60214 in the same strand
CDS YP_001514464.1 integrase catalytic subunit intersects 94.76%(271aa) with IS3_IS3_ORF1 e-value= 2.5e-54 complete sequence hit coverage= 100%, between model(286 aa) positions 1; 286 length is 272aa with 15 gaps, 0 stops, absolute frame= Plus2 at 59399..60214 in the same strand
misc_feature no id putative transposase OrfB; Reviewed; Region: PHA02517 intersects 98.43%(250aa) with IS3_IS3_ORF1 e-value= 2.5e-54 complete sequence hit coverage= 100%, between model(286 aa) positions 1; 286 length is 272aa with 15 gaps, 0 stops, absolute frame= Plus2 at 59399..60214 in the same strand

misc_feature no id HTH-like domain; Region: HTH_21; pfam13276 intersects 100%(42aa) with IS3_IS3_ORF1 e-value= 2.5e-54 complete sequence hit coverage= 100%, between model(286 aa) positions 1; 286 length is 272aa with 15 gaps, 0 stops, absolute frame= Plus2 at 59399..60214 in the same strand
misc_feature no id Integrase core domain; Region: rve; cl01316 intersects 100%(110aa) with IS3_IS3_ORF1 e-value= 2.5e-54 complete sequence hit coverage= 100%, between model(286 aa) positions 1; 286 length is 272aa with 15 gaps, 0 stops, absolute frame= Plus2 at 59399..60214 in the same strand
misc_feature no id Integrase core domain; Region: rve_3; cl15866 intersects 100%(68aa) with IS3_IS3_ORF1 e-value= 2.5e-54 complete sequence hit coverage= 100%, between model(286 aa) positions 1; 286 length is 272aa with 15 gaps, 0 stops, absolute frame= Plus2 at 59399..60214 in the same strand
CDS YP_001514463.1 transposase intersects 2.29%(2aa) with IS3_IS150_ORF1 e-value= 6.3e-50 fragment hit coverage= 92.81%, between model(306 aa) positions 23; 306 length is 272aa with 14 gaps, 0 stops, absolute frame= Plus2 at 59402..60217 in the same strand
CDS YP_001514464.1 integrase catalytic subunit intersects 95.10%(272aa) with IS3_IS150_ORF1 e-value= 6.3e-50 fragment hit coverage= 92.81%, between model(306 aa) positions 23; 306 length is 272aa with 14 gaps, 0 stops, absolute frame= Plus2 at 59402..60217 in the same strand
misc_feature no id putative transposase OrfB; Reviewed; Region: PHA02517 intersects 98.82%(251aa) with IS3_IS150_ORF1 e-value= 6.3e-50 fragment hit coverage= 92.81%, between model(306 aa) positions 23; 306 length is 272aa with 14 gaps, 0 stops, absolute frame= Plus2 at 59402..60217 in the same strand
misc_feature no id HTH-like domain; Region: HTH_21; pfam13276 intersects 100%(42aa) with IS3_IS150_ORF1 e-value= 6.3e-50 fragment hit coverage= 92.81%, between model(306 aa) positions 23; 306 length is 272aa with 14 gaps, 0 stops, absolute frame= Plus2 at 59402..60217 in the same strand
misc_feature no id Integrase core domain; Region: rve; cl01316 intersects 100%(110aa) with IS3_IS150_ORF1 e-value= 6.3e-50 fragment hit coverage= 92.81%, between model(306 aa) positions 23; 306 length is 272aa with 14 gaps, 0 stops, absolute frame= Plus2 at 59402..60217 in the same strand
misc_feature no id Integrase core domain; Region: rve_3; cl15866 intersects 100%(68aa) with IS3_IS150_ORF1 e-value= 6.3e-50 fragment hit coverage= 92.81%, between model(306 aa) positions 23; 306 length is 272aa with 14 gaps, 0 stops, absolute frame= Plus2 at 59402..60217 in the same strand
CDS YP_001514464.1 integrase catalytic subunit intersects 95.10%(272aa) with IS3_IS3_ORF2 e-value= 3.3e-65 complete sequence hit coverage= 100%, between model(288 aa) positions 1; 288 length is 272aa with 19 gaps, 0 stops, absolute frame= Plus2 at 59411..60226 in the same strand
misc_feature no id putative transposase OrfB; Reviewed; Region: PHA02517 intersects 100%(254aa) with IS3_IS3_ORF2 e-value= 3.3e-65 complete sequence hit coverage= 100%, between model(288 aa) positions 1; 288 length is 272aa with 19 gaps, 0 stops, absolute frame= Plus2 at 59411..60226 in the same strand
misc_feature no id HTH-like domain; Region: HTH_21; pfam13276 intersects 100%(42aa) with IS3_IS3_ORF2 e-value= 3.3e-65 complete sequence hit coverage= 100%, between model(288 aa) positions 1; 288 length is 272aa with 19 gaps, 0 stops, absolute frame= Plus2 at 59411..60226 in the same strand
misc_feature no id Integrase core domain; Region: rve; cl01316 intersects 100%(110aa) with IS3_IS3_ORF2 e-value= 3.3e-65 complete sequence hit coverage= 100%, between model(288 aa) positions 1; 288 length is 272aa with 19 gaps, 0 stops, absolute frame= Plus2 at 59411..60226 in the same strand
misc_feature no id Integrase core domain; Region: rve_3; cl15866 intersects 100%(68aa) with IS3_IS3_ORF2 e-value= 3.3e-65 complete sequence hit coverage= 100%, between model(288 aa) positions 1; 288 length is 272aa with 19 gaps, 0 stops, absolute frame= Plus2 at 59411..60226 in the same strand
CDS YP_001514464.1 integrase catalytic subunit intersects 18.53%(53aa) with IS3_IS2_ORF2 e-value= 9.1e-09 fragment hit coverage= 18.87%, between model(302 aa) positions 48; 104 length is 53aa with 5 gaps, 0 stops, absolute frame= Plus2 at 59480..59638 in the same strand
misc_feature no id putative transposase OrfB; Reviewed; Region: PHA02517 intersects 20.87%(53aa) with IS3_IS2_ORF2 e-value= 9.1e-09 fragment hit coverage= 18.87%, between model(302 aa) positions 48; 104 length is 53aa with 5 gaps, 0 stops, absolute frame= Plus2 at 59480..59638 in the same strand
misc_feature no id HTH-like domain; Region: HTH_21; pfam13276 intersects 73.81%(31aa) with IS3_IS2_ORF2 e-value= 9.1e-09 fragment hit coverage= 18.87%, between model(302 aa) positions 48; 104 length is 53aa with 5 gaps, 0 stops, absolute frame= Plus2 at 59480..59638 in the same strand
CDS YP_001514464.1 integrase catalytic subunit intersects 79.72%(228aa) with IS3_IS51_ORF2 e-value= 1.9e-38 fragment hit coverage= 74.76%, between model(317 aa) positions 73; 309 length is 228aa with 11 gaps, 0 stops, absolute frame= Plus2 at 59546..60229 in the same strand
misc_feature no id putative transposase OrfB; Reviewed; Region: PHA02517 intersects 89.37%(227aa) with IS3_IS51_ORF2 e-value= 1.9e-38 fragment hit coverage= 74.76%, between model(317 aa) positions 73; 309 length is 228aa with 11 gaps, 0 stops, absolute frame= Plus2 at 59546..60229 in the same strand

misc_feature no id HTH-like domain; Region: HTH_21; pfam13276 intersects 100%(42aa) with IS3_IS51_ORF2 e-value= 1.9e-38 fragment hit coverage= 74.76%, between model(317 aa) positions 73; 309 length is 228aa with 11 gaps, 0 stops, absolute frame= Plus2 at 59546..60229 in the same strand
misc_feature no id Integrase core domain; Region: rve; cl01316 intersects 100%(110aa) with IS3_IS51_ORF2 e-value= 1.9e-38 fragment hit coverage= 74.76%, between model(317 aa) positions 73; 309 length is 228aa with 11 gaps, 0 stops, absolute frame= Plus2 at 59546..60229 in the same strand
misc_feature no id Integrase core domain; Region: rve_3; cl15866 intersects 100%(68aa) with IS3_IS51_ORF2 e-value= 1.9e-38 fragment hit coverage= 74.76%, between model(317 aa) positions 73; 309 length is 228aa with 11 gaps, 0 stops, absolute frame= Plus2 at 59546..60229 in the same strand
Class A

4.
CDS YP_001514466.1 transposase mutator family protein intersects 96.32%(401aa) with IS256 e-value= 3.7e-167 complete sequence hit coverage= 100%, between model(410 aa) positions 1; 410 length is 404aa with 11 gaps, 1 stops, absolute frame= Minus2 at 61035..62245 in the same strand
misc_feature no id Transposase, Mutator family; Region: Transposase_mut; pfam00872 intersects 100%(362aa) with IS256 e-value= 3.7e-167 complete sequence hit coverage= 100%, between model(410 aa) positions 1; 410 length is 404aa with 11 gaps, 1 stops, absolute frame= Minus2 at 61035..62245 in the same strand
misc_feature no id MULE transposase domain; Region: MULE; pfam10551 intersects 100%(78aa) with IS256 e-value= 3.7e-167 complete sequence hit coverage= 100%, between model(410 aa) positions 1; 410 length is 404aa with 11 gaps, 1 stops, absolute frame= Minus2 at 61035..62245 in the same strand
Class A

5.
CDS YP_001514483.1 IS4 family transposase intersects 100%(267aa) with IS5_IS1031 e-value= 1.9e-108 complete sequence hit coverage= 100%, between model(295 aa) positions 1; 295 length is 284aa with 14 gaps, 2 stops, absolute frame= Plus2 at 86624..87475 in the same strand
misc_feature no id DDE superfamily endonuclease; Region: DDE_4; cl15789 intersects 100%(75aa) with IS5_IS1031 e-value= 1.9e-108 complete sequence hit coverage= 100%, between model(295 aa) positions 1; 295 length is 284aa with 14 gaps, 2 stops, absolute frame= Plus2 at 86624..87475 in the same strand
misc_feature no id Transposase DDE domain; Region: DDE_Tnp_1; pfam01609 intersects 100%(155aa) with IS5_IS1031 e-value= 1.9e-108 complete sequence hit coverage= 100%, between model(295 aa) positions 1; 295 length is 284aa with 14 gaps, 2 stops, absolute frame= Plus2 at 86624..87475 in the same strand
misc_feature no id DDE superfamily endonuclease; Region: DDE_4; cl15789 intersects 100%(83aa) with IS5_IS1031 e-value= 1.9e-108 complete sequence hit coverage= 100%, between model(295 aa) positions 1; 295 length is 284aa with 14 gaps, 2 stops, absolute frame= Plus2 at 86624..87475 in the same strand
CDS YP_001514483.1 IS4 family transposase intersects 98.50%(263aa) with IS5_IS1031 e-value= 1.3e-110 fragment hit coverage= 93.56%, between model(295 aa) positions 20; 295 length is 270aa with 9 gaps, 1 stops, absolute frame= Plus2 at 86666..87475 in the same strand
misc_feature no id DDE superfamily endonuclease; Region: DDE_4; cl15789 intersects 100%(75aa) with IS5_IS1031 e-value= 1.3e-110 fragment hit coverage= 93.56%, between model(295 aa) positions 20; 295 length is 270aa with 9 gaps, 1 stops, absolute frame= Plus2 at 86666..87475 in the same strand
misc_feature no id Transposase DDE domain; Region: DDE_Tnp_1; pfam01609 intersects 100%(155aa) with IS5_IS1031 e-value= 1.3e-110 fragment hit coverage= 93.56%, between model(295 aa) positions 20; 295 length is 270aa with 9 gaps, 1 stops, absolute frame= Plus2 at 86666..87475 in the same strand
misc_feature no id DDE superfamily endonuclease; Region: DDE_4; cl15789 intersects 100%(83aa) with IS5_IS1031 e-value= 1.3e-110 fragment hit coverage= 93.56%, between model(295 aa) positions 20; 295 length is 270aa with 9 gaps, 1 stops, absolute frame= Plus2 at 86666..87475 in the same strand
CDS YP_001514483.1 IS4 family transposase intersects 29.96%(80aa) with IS5_IS427 e-value= 3.3e-18 fragment hit coverage= 27.70%, between model(296 aa) positions 125; 206 length is 80aa with 2 gaps, 0 stops, absolute frame= Plus2 at 86675..86914 in the same strand
misc_feature no id DDE superfamily endonuclease; Region: DDE_4; cl15789 intersects 100%(75aa) with IS5_IS427 e-value= 3.3e-18 fragment hit coverage= 27.70%, between model(296 aa) positions 125; 206 length is 80aa with 2 gaps, 0 stops, absolute frame= Plus2 at 86675..86914 in the same strand
Class A

6.

CDS YP_001514497.1 transposase intersects 86.18%(357aa) with ISL3 e-value= 1e-49 fragment hit coverage= 86.03%, between model(451 aa) positions 64; 451 length is 360aa with 34 gaps, 1 stops, absolute frame= Minus1 at 99016..100094 in the same strand
misc_feature no id Transposase and inactivated derivatives [DNA replication, recombination, and repair]; Region: COG3464 intersects 85.77%(345aa) with ISL3 e-value= 1e-49 fragment hit coverage= 86.03%, between model(451 aa) positions 64; 451 length is 360aa with 34 gaps, 1 stops, absolute frame= Minus1 at 99016..100094 in the same strand
misc_feature no id Transposase; Region: DDE_Tnp_ISL3; pfam01610 intersects 100%(246aa) with ISL3 e-value= 1e-49 fragment hit coverage= 86.03%, between model(451 aa) positions 64; 451 length is 360aa with 34 gaps, 1 stops, absolute frame= Minus1 at 99016..100094 in the same strand
CDS YP_001514497.1 transposase intersects 98.96%(410aa) with ISL3 e-value= 1.8e-43 complete sequence hit coverage= 100%, between model(451 aa) positions 1; 451 length is 413aa with 44 gaps, 1 stops, absolute frame= Minus1 at 99016..100253 in the same strand
misc_feature no id Transposase and inactivated derivatives [DNA replication, recombination, and repair]; Region: COG3464 intersects 98.92%(398aa) with ISL3 e-value= 1.8e-43 complete sequence hit coverage= 100%, between model(451 aa) positions 1; 451 length is 413aa with 44 gaps, 1 stops, absolute frame= Minus1 at 99016..100253 in the same strand
misc_feature no id Transposase; Region: DDE_Tnp_ISL3; pfam01610 intersects 100%(246aa) with ISL3 e-value= 1.8e-43 complete sequence hit coverage= 100%, between model(451 aa) positions 1; 451 length is 413aa with 44 gaps, 1 stops, absolute frame= Minus1 at 99016..100253 in the same strand
Class A

7.
CDS YP_001514504.1 transposase mutator family protein intersects 96.40%(402aa) with IS256 e-value= 3.7e-167 complete sequence hit coverage= 100%, between model(410 aa) positions 1; 410 length is 404aa with 11 gaps, 1 stops, absolute frame= Plus2 at 104411..105622 in the same strand
misc_feature no id Transposase, Mutator family; Region: Transposase_mut; pfam00872 intersects 100%(362aa) with IS256 e-value= 3.7e-167 complete sequence hit coverage= 100%, between model(410 aa) positions 1; 410 length is 404aa with 11 gaps, 1 stops, absolute frame= Plus2 at 104411..105622 in the same strand
misc_feature no id MULE transposase domain; Region: MULE; pfam10551 intersects 100%(78aa) with IS256 e-value= 3.7e-167 complete sequence hit coverage= 100%, between model(410 aa) positions 1; 410 length is 404aa with 11 gaps, 1 stops, absolute frame= Plus2 at 104411..105622 in the same strand
Class A

8.
CDS YP_001514505.1 hypothetical protein intersects 38.46%(15aa) with IS200_IS605 e-value= 4.7e-06 fragment hit coverage= 20.26%, between model(153 aa) positions 1; 31 length is 31aa with 0 gaps, 0 stops, absolute frame= Minus2 at 105807..105898 in the same strand
CDS YP_001514505.1 hypothetical protein intersects 38.46%(15aa) with IS605 e-value= 9.3e-06 fragment hit coverage= 21.05%, between model(152 aa) positions 1; 32 length is 29aa with 3 gaps, 0 stops, absolute frame= Minus2 at 105807..105892 in the same strand
CDS YP_001514505.1 hypothetical protein intersects 33.33%(13aa) with IS200 e-value= 2.1e-06 fragment hit coverage= 18.83%, between model(154 aa) positions 1; 29 length is 29aa with 0 gaps, 0 stops, absolute frame= Minus2 at 105813..105898 in the same strand
Class B

9.
CDS YP_001514526.1 transposase intersects 83.56%(122aa) with ISAs1 e-value= 2.4e-27 fragment hit coverage= 41.65%, between model(389 aa) positions 198; 359 length is 148aa with 15 gaps, 0 stops, absolute frame= Minus3 at 126635..127077 in the same strand
misc_feature no id Transposase [DNA replication, recombination, and repair]; Region: COG5433 intersects 100%(47aa) with ISAs1 e-value= 2.4e-27 fragment hit coverage= 41.65%, between model(389 aa) positions 198; 359 length is 148aa with 15 gaps, 0 stops, absolute frame= Minus3 at 126635..127077 in the same strand
Class A

10.

gene no id fragment; similar to N-terminus of transposase intersects 48.69%(93aa) with ISAs1 e-value= 1.2e-14 fragment hit coverage= 25.45%, between model(389 aa) positions 1; 99 length is 94aa with 5 gaps, 0 stops, absolute frame= Minus2 at 127374..127654 in the same strand
Class A

11.
CDS YP_001514529.1 hypothetical protein intersects 93.42%(345aa) with IS630 e-value= 6.7e-25 complete sequence hit coverage= 100%, between model(376 aa) positions 1; 376 length is 346aa with 33 gaps, 0 stops, absolute frame= Minus1 at 128584..129620 in the same strand
misc_feature no id Integrase core domain; Region: rve; cl01316 intersects 100%(141aa) with IS630 e-value= 6.7e-25 complete sequence hit coverage= 100%, between model(376 aa) positions 1; 376 length is 346aa with 33 gaps, 0 stops, absolute frame= Minus1 at 128584..129620 in the same strand
misc_feature no id Winged helix-turn helix; Region: HTH_33; pfam13592 intersects 100%(56aa) with IS630 e-value= 6.7e-25 complete sequence hit coverage= 100%, between model(376 aa) positions 1; 376 length is 346aa with 33 gaps, 0 stops, absolute frame= Minus1 at 128584..129620 in the same strand
misc_feature no id Winged helix-turn helix; Region: HTH_29; pfam13551 intersects 100%(102aa) with IS630 e-value= 6.7e-25 complete sequence hit coverage= 100%, between model(376 aa) positions 1; 376 length is 346aa with 33 gaps, 0 stops, absolute frame= Minus1 at 128584..129620 in the same strand
misc_feature no id Helix-turn-helix domains; Region: HTH; cl00088 intersects 100%(50aa) with IS630 e-value= 6.7e-25 complete sequence hit coverage= 100%, between model(376 aa) positions 1; 376 length is 346aa with 33 gaps, 0 stops, absolute frame= Minus1 at 128584..129620 in the same strand
CDS YP_001514529.1 hypothetical protein intersects 14.23%(52aa) with IS481 e-value= 3.7e-09 fragment hit coverage= 15.10%, between model(351 aa) positions 1; 53 length is 53aa with 0 gaps, 0 stops, absolute frame= Minus1 at 129403..129560 in the same strand
misc_feature no id Winged helix-turn helix; Region: HTH_29; pfam13551 intersects 43.14%(44aa) with IS481 e-value= 3.7e-09 fragment hit coverage= 15.10%, between model(351 aa) positions 1; 53 length is 53aa with 0 gaps, 0 stops, absolute frame= Minus1 at 129403..129560 in the same strand
misc_feature no id Helix-turn-helix domains; Region: HTH; cl00088 intersects 100%(50aa) with IS481 e-value= 3.7e-09 fragment hit coverage= 15.10%, between model(351 aa) positions 1; 53 length is 53aa with 0 gaps, 0 stops, absolute frame= Minus1 at 129403..129560 in the same strand
Class A

12.
CDS YP_001514560.1 hypothetical protein intersects 93.51%(346aa) with IS630 e-value= 4.9e-24 complete sequence hit coverage= 100%, between model(376 aa) positions 1; 376 length is 346aa with 33 gaps, 0 stops, absolute frame= Plus2 at 159026..160063 in the same strand
misc_feature no id Helix-turn-helix domains; Region: HTH; cl00088 intersects 100%(50aa) with IS630 e-value= 4.9e-24 complete sequence hit coverage= 100%, between model(376 aa) positions 1; 376 length is 346aa with 33 gaps, 0 stops, absolute frame= Plus2 at 159026..160063 in the same strand
misc_feature no id Winged helix-turn helix; Region: HTH_29; pfam13551 intersects 100%(102aa) with IS630 e-value= 4.9e-24 complete sequence hit coverage= 100%, between model(376 aa) positions 1; 376 length is 346aa with 33 gaps, 0 stops, absolute frame= Plus2 at 159026..160063 in the same strand
misc_feature no id Winged helix-turn helix; Region: HTH_33; pfam13592 intersects 100%(56aa) with IS630 e-value= 4.9e-24 complete sequence hit coverage= 100%, between model(376 aa) positions 1; 376 length is 346aa with 33 gaps, 0 stops, absolute frame= Plus2 at 159026..160063 in the same strand
misc_feature no id Integrase core domain; Region: rve; cl01316 intersects 100%(141aa) with IS630 e-value= 4.9e-24 complete sequence hit coverage= 100%, between model(376 aa) positions 1; 376 length is 346aa with 33 gaps, 0 stops, absolute frame= Plus2 at 159026..160063 in the same strand
CDS YP_001514560.1 hypothetical protein intersects 14.32%(53aa) with IS481 e-value= 3.7e-09 fragment hit coverage= 15.10%, between model(351 aa) positions 1; 53 length is 53aa with 0 gaps, 0 stops, absolute frame= Plus2 at 159086..159244 in the same strand
misc_feature no id Helix-turn-helix domains; Region: HTH; cl00088 intersects 100%(50aa) with IS481 e-value= 3.7e-09 fragment hit coverage= 15.10%, between model(351 aa) positions 1; 53 length is 53aa with 0 gaps, 0 stops, absolute frame= Plus2 at 159086..159244 in the same strand
misc_feature no id Winged helix-turn helix; Region: HTH_29; pfam13551 intersects 43.14%(44aa) with IS481 e-value= 3.7e-09 fragment hit coverage= 15.10%, between model(351 aa) positions 1; 53 length is 53aa with 0 gaps, 0 stops, absolute frame= Plus2 at 159086..159244 in the same strand

Class A

13.
CDS YP_001514623.1 transposase intersects 85.96%(375aa) with IS4 e-value= 9.9e-07 complete sequence hit coverage= 100%, between model(486 aa) positions 1; 486 length is 376aa with 117 gaps, 0 stops, absolute frame= Minus3 at 219818..220944 in the same strand
misc_feature no id DDE superfamily endonuclease; Region: DDE_5; cl02413 intersects 93.48%(348aa) with IS4 e-value= 9.9e-07 complete sequence hit coverage= 100%, between model(486 aa) positions 1; 486 length is 376aa with 117 gaps, 0 stops, absolute frame= Minus3 at 219818..220944 in the same strand
CDS YP_001514623.1 transposase intersects 53.01%(231aa) with IS4 e-value= 4.7e-14 fragment hit coverage= 51.23%, between model(486 aa) positions 1; 249 length is 232aa with 27 gaps, 0 stops, absolute frame= Minus3 at 220250..220944 in the same strand
misc_feature no id DDE superfamily endonuclease; Region: DDE_5; cl02413 intersects 62.11%(231aa) with IS4 e-value= 4.7e-14 fragment hit coverage= 51.23%, between model(486 aa) positions 1; 249 length is 232aa with 27 gaps, 0 stops, absolute frame= Minus3 at 220250..220944 in the same strand
Class A

14.
CDS YP_001514653.1 IS4 family transposase intersects 100%(267aa) with IS5_IS1031 e-value= 6.8e-110 complete sequence hit coverage= 100%, between model(295 aa) positions 1; 295 length is 286aa with 14 gaps, 0 stops, absolute frame= Plus2 at 249134..249991 in the same strand
misc_feature no id DDE superfamily endonuclease; Region: DDE_4; cl15789 intersects 100%(75aa) with IS5_IS1031 e-value= 6.8e-110 complete sequence hit coverage= 100%, between model(295 aa) positions 1; 295 length is 286aa with 14 gaps, 0 stops, absolute frame= Plus2 at 249134..249991 in the same strand
misc_feature no id Transposase DDE domain; Region: DDE_Tnp_1; pfam01609 intersects 100%(155aa) with IS5_IS1031 e-value= 6.8e-110 complete sequence hit coverage= 100%, between model(295 aa) positions 1; 295 length is 286aa with 14 gaps, 0 stops, absolute frame= Plus2 at 249134..249991 in the same strand
misc_feature no id DDE superfamily endonuclease; Region: DDE_4; cl15789 intersects 100%(83aa) with IS5_IS1031 e-value= 6.8e-110 complete sequence hit coverage= 100%, between model(295 aa) positions 1; 295 length is 286aa with 14 gaps, 0 stops, absolute frame= Plus2 at 249134..249991 in the same strand
CDS YP_001514653.1 IS4 family transposase intersects 32.58%(87aa) with IS5_IS427 e-value= 1e-22 fragment hit coverage= 30.07%, between model(296 aa) positions 118; 206 length is 87aa with 2 gaps, 0 stops, absolute frame= Plus2 at 249164..249424 in the same strand
misc_feature no id DDE superfamily endonuclease; Region: DDE_4; cl15789 intersects 100%(75aa) with IS5_IS427 e-value= 1e-22 fragment hit coverage= 30.07%, between model(296 aa) positions 118; 206 length is 87aa with 2 gaps, 0 stops, absolute frame= Plus2 at 249164..249424 in the same strand
CDS YP_001514653.1 IS4 family transposase intersects 98.50%(263aa) with IS5_IS1031 e-value= 1.4e-111 fragment hit coverage= 93.56%, between model(295 aa) positions 20; 295 length is 272aa with 9 gaps, 0 stops, absolute frame= Plus2 at 249176..249991 in the same strand
misc_feature no id DDE superfamily endonuclease; Region: DDE_4; cl15789 intersects 100%(75aa) with IS5_IS1031 e-value= 1.4e-111 fragment hit coverage= 93.56%, between model(295 aa) positions 20; 295 length is 272aa with 9 gaps, 0 stops, absolute frame= Plus2 at 249176..249991 in the same strand
misc_feature no id Transposase DDE domain; Region: DDE_Tnp_1; pfam01609 intersects 100%(155aa) with IS5_IS1031 e-value= 1.4e-111 fragment hit coverage= 93.56%, between model(295 aa) positions 20; 295 length is 272aa with 9 gaps, 0 stops, absolute frame= Plus2 at 249176..249991 in the same strand
misc_feature no id DDE superfamily endonuclease; Region: DDE_4; cl15789 intersects 100%(83aa) with IS5_IS1031 e-value= 1.4e-111 fragment hit coverage= 93.56%, between model(295 aa) positions 20; 295 length is 272aa with 9 gaps, 0 stops, absolute frame= Plus2 at 249176..249991 in the same strand
Class A

15.
CDS YP_001514660.1 hypothetical protein intersects 71.46%(119aa) with IS1_ORF1 e-value= 2.6e-07 complete sequence hit coverage= 100%, between model(239 aa) positions 1; 239 length is 234aa with 24 gaps, 7 stops, absolute frame= Minus3 at 259316..260016 in the same strand
misc_feature no id IS1 transposase; Region: DDE_Tnp_IS1; cl00721 intersects 100%(93aa) with IS1_ORF1 e-value= 2.6e-07 complete sequence hit coverage= 100%, between model(239 aa) positions 1; 239 length is 234aa with 24 gaps, 7 stops, absolute frame= Minus3 at 259316..260016 in the same strand

CDS YP_001514661.1 hypothetical protein intersects 99.75%(130aa) with IS1_ORF1 e-value= 2.6e-07 complete sequence hit coverage= 100%, between model(239 aa) positions 1; 239 length is 234aa with 24 gaps, 7 stops, absolute frame= Minus3 at 259316..260016 in the same strand
misc_feature no id Transposase and inactivated derivatives [DNA replication, recombination, and repair]; Region: COG3677 intersects 99.68%(104aa) with IS1_ORF1 e-value= 2.6e-07 complete sequence hit coverage= 100%, between model(239 aa) positions 1; 239 length is 234aa with 24 gaps, 7 stops, absolute frame= Minus3 at 259316..260016 in the same strand
CDS YP_001514660.1 hypothetical protein intersects 27.35%(45aa) with IS1_ORF2 e-value= 1.8e-11 fragment hit coverage= 20.68%, between model(237 aa) positions 164; 212 length is 46aa with 3 gaps, 0 stops, absolute frame= Minus2 at 259350..259486 in the same strand
misc_feature no id IS1 transposase; Region: DDE_Tnp_IS1; cl00721 intersects 49.10%(45aa) with IS1_ORF2 e-value= 1.8e-11 fragment hit coverage= 20.68%, between model(237 aa) positions 164; 212 length is 46aa with 3 gaps, 0 stops, absolute frame= Minus2 at 259350..259486 in the same strand
CDS YP_001514661.1 hypothetical protein intersects 66.92%(87aa) with IS1_ORF1 e-value= 1.7e-15 fragment hit coverage= 36.40%, between model(239 aa) positions 1; 87 length is 88aa with 2 gaps, 0 stops, absolute frame= Minus3 at 259754..260016 in the same strand
misc_feature no id Transposase and inactivated derivatives [DNA replication, recombination, and repair]; Region: COG3677 intersects 83.49%(87aa) with IS1_ORF1 e-value= 1.7e-15 fragment hit coverage= 36.40%, between model(239 aa) positions 1; 87 length is 88aa with 2 gaps, 0 stops, absolute frame= Minus3 at 259754..260016 in the same strand
Class A

16.
CDS YP_001514717.1 IS4 family transposase intersects 99.35%(308aa) with IS5_IS903 e-value= 2.2e-96 complete sequence hit coverage= 100%, between model(347 aa) positions 1; 347 length is 314aa with 37 gaps, 1 stops, absolute frame= Plus3 at 316002..316943 in the same strand
misc_feature no id Transposase DDE domain; Region: DDE_Tnp_1_5; pfam13737 intersects 100%(112aa) with IS5_IS903 e-value= 2.2e-96 complete sequence hit coverage= 100%, between model(347 aa) positions 1; 347 length is 314aa with 37 gaps, 1 stops, absolute frame= Plus3 at 316002..316943 in the same strand
misc_feature no id Transposase DDE domain; Region: DDE_Tnp_1; pfam01609 intersects 100%(186aa) with IS5_IS903 e-value= 2.2e-96 complete sequence hit coverage= 100%, between model(347 aa) positions 1; 347 length is 314aa with 37 gaps, 1 stops, absolute frame= Plus3 at 316002..316943 in the same strand
Class A

17.
CDS YP_001514725.1 hypothetical protein intersects 39.56%(36aa) with IS630 e-value= 4.3e-06 fragment hit coverage= 11.70%, between model(376 aa) positions 279; 322 length is 51aa with 1 gaps, 0 stops, absolute frame= Minus2 at 321891..322042 in the same strand
misc_feature no id Integrase core domain; Region: rve; cl01316 intersects 84.21%(16aa) with IS630 e-value= 4.3e-06 fragment hit coverage= 11.70%, between model(376 aa) positions 279; 322 length is 51aa with 1 gaps, 0 stops, absolute frame= Minus2 at 321891..322042 in the same strand
Class A

18.
CDS YP_001514726.1 hypothetical protein intersects 47.43%(129aa) with IS630 e-value= 1.1e-13 fragment hit coverage= 38.83%, between model(376 aa) positions 188; 333 length is 129aa with 20 gaps, 0 stops, absolute frame= Plus1 at 322582..322968 in the same strand
misc_feature no id Integrase core domain; Region: rve; cl01316 intersects 90.21%(129aa) with IS630 e-value= 1.1e-13 fragment hit coverage= 38.83%, between model(376 aa) positions 188; 333 length is 129aa with 20 gaps, 0 stops, absolute frame= Plus1 at 322582..322968 in the same strand
Class A

19.
CDS YP_001514728.1 hypothetical protein intersects 76.22%(135aa) with IS630 e-value= 9.2e-30 fragment hit coverage= 37.50%, between model(376 aa) positions 30; 170 length is 136aa with 5 gaps, 0 stops, absolute frame= Minus2 at 323280..323686 in the same strand

misc_feature no id Winged helix-turn helix; Region: HTH_33; pfam13592 intersects 98.33%(59aa) with IS630 e-value= 9.2e-30 fragment hit coverage= 37.50%, between model(376 aa) positions 30; 170 length is 136aa with 5 gaps, 0 stops, absolute frame= Minus2 at 323280..323686 in the same strand
misc_feature no id Winged helix-turn helix; Region: HTH_29; pfam13551 intersects 99.70%(111aa) with IS630 e-value= 9.2e-30 fragment hit coverage= 37.50%, between model(376 aa) positions 30; 170 length is 136aa with 5 gaps, 0 stops, absolute frame= Minus2 at 323280..323686 in the same strand
misc_feature no id Helix-turn-helix domains; Region: HTH; cl00088 intersects 92.91%(43aa) with IS630 e-value= 9.2e-30 fragment hit coverage= 37.50%, between model(376 aa) positions 30; 170 length is 136aa with 5 gaps, 0 stops, absolute frame= Minus2 at 323280..323686 in the same strand
CDS YP_001514728.1 hypothetical protein intersects 44.76%(79aa) with IS481 e-value= 6.3e-08 fragment hit coverage= 23.36%, between model(351 aa) positions 1; 82 length is 80aa with 2 gaps, 0 stops, absolute frame= Minus2 at 323475..323713 in the same strand
misc_feature no id Winged helix-turn helix; Region: HTH_29; pfam13551 intersects 63.39%(71aa) with IS481 e-value= 6.3e-08 fragment hit coverage= 23.36%, between model(351 aa) positions 1; 82 length is 80aa with 2 gaps, 0 stops, absolute frame= Minus2 at 323475..323713 in the same strand
misc_feature no id Helix-turn-helix domains; Region: HTH; cl00088 intersects 100%(47aa) with IS481 e-value= 6.3e-08 fragment hit coverage= 23.36%, between model(351 aa) positions 1; 82 length is 80aa with 2 gaps, 0 stops, absolute frame= Minus2 at 323475..323713 in the same strand
Class B

20.
gene no id transposase, IS200 family intersects 100%(127aa) with IS605 e-value= 2.6e-17 complete sequence hit coverage= 100%, between model(152 aa) positions 1; 152 length is 160aa with 0 gaps, 2 stops, absolute frame= Minus2 at 338973..339451 in the same strand
gene no id transposase, IS200 family intersects 100%(127aa) with IS200_IS605 e-value= 4.2e-45 complete sequence hit coverage= 100%, between model(153 aa) positions 1; 153 length is 152aa with 1 gaps, 2 stops, absolute frame= Minus2 at 338994..339448 in the same strand
gene no id transposase, IS200 family intersects 100%(127aa) with IS200 e-value= 1.5e-46 complete sequence hit coverage= 100%, between model(154 aa) positions 1; 154 length is 145aa with 9 gaps, 2 stops, absolute frame= Minus2 at 339015..339448 in the same strand
gene no id transposase, IS200 family intersects 88.19%(112aa) with IS200_IS605 e-value= 1.3e-45 fragment hit coverage= 84.97%, between model(153 aa) positions 1; 130 length is 129aa with 1 gaps, 1 stops, absolute frame= Minus2 at 339063..339448 in the same strand
gene no id transposase, IS200 family intersects 72.44%(92aa) with IS605 e-value= 3e-25 fragment hit coverage= 72.37%, between model(152 aa) positions 1; 110 length is 110aa with 0 gaps, 1 stops, absolute frame= Minus2 at 339123..339451 in the same strand
Class A

21.
CDS YP_001514804.1 IS4 family transposase intersects 100%(267aa) with IS5_IS1031 e-value= 4.2e-111 complete sequence hit coverage= 100%, between model(295 aa) positions 1; 295 length is 284aa with 14 gaps, 2 stops, absolute frame= Minus1 at 408043..408893 in the same strand
misc_feature no id DDE superfamily endonuclease; Region: DDE_4; cl15789 intersects 100%(83aa) with IS5_IS1031 e-value= 4.2e-111 complete sequence hit coverage= 100%, between model(295 aa) positions 1; 295 length is 284aa with 14 gaps, 2 stops, absolute frame= Minus1 at 408043..408893 in the same strand
misc_feature no id Transposase DDE domain; Region: DDE_Tnp_1; pfam01609 intersects 100%(155aa) with IS5_IS1031 e-value= 4.2e-111 complete sequence hit coverage= 100%, between model(295 aa) positions 1; 295 length is 284aa with 14 gaps, 2 stops, absolute frame= Minus1 at 408043..408893 in the same strand
misc_feature no id DDE superfamily endonuclease; Region: DDE_4; cl15789 intersects 100%(75aa) with IS5_IS1031 e-value= 4.2e-111 complete sequence hit coverage= 100%, between model(295 aa) positions 1; 295 length is 284aa with 14 gaps, 2 stops, absolute frame= Minus1 at 408043..408893 in the same strand
CDS YP_001514804.1 IS4 family transposase intersects 98.38%(262aa) with IS5_IS1031 e-value= 2.8e-113 fragment hit coverage= 93.56%, between model(295 aa) positions 20; 295 length is 270aa with 9 gaps, 1 stops, absolute frame= Minus1 at 408043..408851 in the same strand
misc_feature no id DDE superfamily endonuclease; Region: DDE_4; cl15789 intersects 100%(83aa) with IS5_IS1031 e-value= 2.8e-113 fragment hit coverage= 93.56%, between model(295 aa) positions 20; 295 length is 270aa with 9 gaps, 1 stops, absolute frame= Minus1 at 408043..408851 in the same strand

misc_feature no id Transposase DDE domain; Region: DDE_Tnp_1; pfam01609 intersects 100%(155aa) with IS5_IS1031 e-value= 2.8e-113 fragment hit coverage= 93.56%, between model(295 aa) positions 20; 295 length is 270aa with 9 gaps, 1 stops, absolute frame= Minus1 at 408043..408851 in the same strand
misc_feature no id DDE superfamily endonuclease; Region: DDE_4; cl15789 intersects 100%(75aa) with IS5_IS1031 e-value= 2.8e-113 fragment hit coverage= 93.56%, between model(295 aa) positions 20; 295 length is 270aa with 9 gaps, 1 stops, absolute frame= Minus1 at 408043..408851 in the same strand
CDS YP_001514804.1 IS4 family transposase intersects 100%(267aa) with IS5_IS5 e-value= 1.3e-09 complete sequence hit coverage= 100%, between model(422 aa) positions 1; 422 length is 290aa with 140 gaps, 4 stops, absolute frame= Minus1 at 408064..408932 in the same strand
misc_feature no id DDE superfamily endonuclease; Region: DDE_4; cl15789 intersects 100%(83aa) with IS5_IS5 e-value= 1.3e-09 complete sequence hit coverage= 100%, between model(422 aa) positions 1; 422 length is 290aa with 140 gaps, 4 stops, absolute frame= Minus1 at 408064..408932 in the same strand
misc_feature no id Transposase DDE domain; Region: DDE_Tnp_1; pfam01609 intersects 100%(155aa) with IS5_IS5 e-value= 1.3e-09 complete sequence hit coverage= 100%, between model(422 aa) positions 1; 422 length is 290aa with 140 gaps, 4 stops, absolute frame= Minus1 at 408064..408932 in the same strand
misc_feature no id DDE superfamily endonuclease; Region: DDE_4; cl15789 intersects 100%(75aa) with IS5_IS5 e-value= 1.3e-09 complete sequence hit coverage= 100%, between model(422 aa) positions 1; 422 length is 290aa with 140 gaps, 4 stops, absolute frame= Minus1 at 408064..408932 in the same strand
CDS YP_001514805.1 phage integrase intersects 2.15%(7aa) with IS5_IS5 e-value= 1.3e-09 complete sequence hit coverage= 100%, between model(422 aa) positions 1; 422 length is 290aa with 140 gaps, 4 stops, absolute frame= Minus1 at 408064..408932 in the opposite strand
CDS YP_001514804.1 IS4 family transposase intersects 29.84%(79aa) with IS5_IS427 e-value= 2e-19 fragment hit coverage= 27.70%, between model(296 aa) positions 125; 206 length is 80aa with 2 gaps, 0 stops, absolute frame= Minus1 at 408604..408842 in the same strand
misc_feature no id DDE superfamily endonuclease; Region: DDE_4; cl15789 intersects 100%(75aa) with IS5_IS427 e-value= 2e-19 fragment hit coverage= 27.70%, between model(296 aa) positions 125; 206 length is 80aa with 2 gaps, 0 stops, absolute frame= Minus1 at 408604..408842 in the same strand
Class A

22.
CDS YP_001514832.1 IS4 family transposase intersects 100%(267aa) with IS5_IS1031 e-value= 2.3e-109 complete sequence hit coverage= 100%, between model(295 aa) positions 1; 295 length is 284aa with 14 gaps, 2 stops, absolute frame= Plus2 at 440219..441070 in the same strand
misc_feature no id DDE superfamily endonuclease; Region: DDE_4; cl15789 intersects 100%(75aa) with IS5_IS1031 e-value= 2.3e-109 complete sequence hit coverage= 100%, between model(295 aa) positions 1; 295 length is 284aa with 14 gaps, 2 stops, absolute frame= Plus2 at 440219..441070 in the same strand
misc_feature no id Transposase DDE domain; Region: DDE_Tnp_1; pfam01609 intersects 100%(155aa) with IS5_IS1031 e-value= 2.3e-109 complete sequence hit coverage= 100%, between model(295 aa) positions 1; 295 length is 284aa with 14 gaps, 2 stops, absolute frame= Plus2 at 440219..441070 in the same strand
misc_feature no id DDE superfamily endonuclease; Region: DDE_4; cl15789 intersects 100%(83aa) with IS5_IS1031 e-value= 2.3e-109 complete sequence hit coverage= 100%, between model(295 aa) positions 1; 295 length is 284aa with 14 gaps, 2 stops, absolute frame= Plus2 at 440219..441070 in the same strand
CDS YP_001514832.1 IS4 family transposase intersects 98.50%(263aa) with IS5_IS1031 e-value= 1.5e-111 fragment hit coverage= 93.56%, between model(295 aa) positions 20; 295 length is 270aa with 9 gaps, 1 stops, absolute frame= Plus2 at 440261..441070 in the same strand
misc_feature no id DDE superfamily endonuclease; Region: DDE_4; cl15789 intersects 100%(75aa) with IS5_IS1031 e-value= 1.5e-111 fragment hit coverage= 93.56%, between model(295 aa) positions 20; 295 length is 270aa with 9 gaps, 1 stops, absolute frame= Plus2 at 440261..441070 in the same strand
misc_feature no id Transposase DDE domain; Region: DDE_Tnp_1; pfam01609 intersects 100%(155aa) with IS5_IS1031 e-value= 1.5e-111 fragment hit coverage= 93.56%, between model(295 aa) positions 20; 295 length is 270aa with 9 gaps, 1 stops, absolute frame= Plus2 at 440261..441070 in the same strand
misc_feature no id DDE superfamily endonuclease; Region: DDE_4; cl15789 intersects 100%(83aa) with IS5_IS1031 e-value= 1.5e-111 fragment hit coverage= 93.56%, between model(295 aa) positions 20; 295 length is 270aa with 9 gaps, 1 stops, absolute frame= Plus2 at 440261..441070 in the same strand
CDS YP_001514832.1 IS4 family transposase intersects 29.96%(80aa) with IS5_IS427 e-value= 2e-19 fragment hit coverage= 27.70%, between model(296 aa) positions 125; 206 length is 80aa with 2 gaps, 0 stops, absolute frame= Plus2 at 440270..440509 in the same strand

misc_feature no id DDE superfamily endonuclease; Region: DDE_4; cl15789 intersects 100%(75aa) with IS5_IS427 e-value= 2e-19 fragment hit coverage= 27.70%, between model(296 aa) positions 125; 206 length is 80aa with 2 gaps, 0 stops, absolute frame= Plus2 at 440270..440509 in the same strand
Class A

23.
gene no id fragment; similar to N-terminus of transposase intersects 34.80%(31aa) with IS630 e-value= 1.7e-09 fragment hit coverage= 9.04%, between model(376 aa) positions 300; 333 length is 32aa with 2 gaps, 0 stops, absolute frame= Minus2 at 466083..466177 in the same strand
Class A

24.
CDS YP_001514856.1 transposase intersects 21.35%(27aa) with IS630 e-value= 3e-16 fragment hit coverage= 50.80%, between model(376 aa) positions 140; 330 length is 165aa with 28 gaps, 0 stops, absolute frame= Plus3 at 469320..469814 in the same strand
misc_feature no id Transposase; Region: HTH_Tnp_IS630; pfam01710 intersects 18.77%(22aa) with IS630 e-value= 3e-16 fragment hit coverage= 50.80%, between model(376 aa) positions 140; 330 length is 165aa with 28 gaps, 0 stops, absolute frame= Plus3 at 469320..469814 in the same strand
CDS YP_001514857.1 transposase intersects 78.68%(107aa) with IS630 e-value= 3e-16 fragment hit coverage= 50.80%, between model(376 aa) positions 140; 330 length is 165aa with 28 gaps, 0 stops, absolute frame= Plus3 at 469320..469814 in the same strand
misc_feature no id Integrase core domain; Region: rve; cl01316 intersects 88.33%(106aa) with IS630 e-value= 3e-16 fragment hit coverage= 50.80%, between model(376 aa) positions 140; 330 length is 165aa with 28 gaps, 0 stops, absolute frame= Plus3 at 469320..469814 in the same strand
Class A

25.
CDS YP_001514858.1 hypothetical protein intersects 86.41%(135aa) with IS630 e-value= 3.4e-15 fragment hit coverage= 39.63%, between model(376 aa) positions 1; 149 length is 136aa with 13 gaps, 0 stops, absolute frame= Minus3 at 470162..470568 in the same strand
misc_feature no id Winged helix-turn helix; Region: HTH_29; pfam13551 intersects 100%(102aa) with IS630 e-value= 3.4e-15 fragment hit coverage= 39.63%, between model(376 aa) positions 1; 149 length is 136aa with 13 gaps, 0 stops, absolute frame= Minus3 at 470162..470568 in the same strand
misc_feature no id Helix-turn-helix domains; Region: HTH; cl00088 intersects 100%(72aa) with IS630 e-value= 3.4e-15 fragment hit coverage= 39.63%, between model(376 aa) positions 1; 149 length is 136aa with 13 gaps, 0 stops, absolute frame= Minus3 at 470162..470568 in the same strand
misc_feature no id Helix-turn-helix domains; Region: HTH; cl00088 intersects 100%(50aa) with IS630 e-value= 3.4e-15 fragment hit coverage= 39.63%, between model(376 aa) positions 1; 149 length is 136aa with 13 gaps, 0 stops, absolute frame= Minus3 at 470162..470568 in the same strand
CDS YP_001514858.1 hypothetical protein intersects 71.76%(112aa) with IS481 e-value= 3.5e-09 fragment hit coverage= 33.62%, between model(351 aa) positions 1; 118 length is 113aa with 6 gaps, 0 stops, absolute frame= Minus3 at 470171..470508 in the same strand
misc_feature no id Winged helix-turn helix; Region: HTH_29; pfam13551 intersects 100%(102aa) with IS481 e-value= 3.5e-09 fragment hit coverage= 33.62%, between model(351 aa) positions 1; 118 length is 113aa with 6 gaps, 0 stops, absolute frame= Minus3 at 470171..470508 in the same strand
misc_feature no id Helix-turn-helix domains; Region: HTH; cl00088 intersects 100%(72aa) with IS481 e-value= 3.5e-09 fragment hit coverage= 33.62%, between model(351 aa) positions 1; 118 length is 113aa with 6 gaps, 0 stops, absolute frame= Minus3 at 470171..470508 in the same strand
misc_feature no id Helix-turn-helix domains; Region: HTH; cl00088 intersects 100%(50aa) with IS481 e-value= 3.5e-09 fragment hit coverage= 33.62%, between model(351 aa) positions 1; 118 length is 113aa with 6 gaps, 0 stops, absolute frame= Minus3 at 470171..470508 in the same strand
Class B

26.
CDS YP_001514859.1 hypothetical protein intersects 20.37%(11aa) with IS1_ORF1 e-value= 4.6e-14 fragment hit coverage= 36.40%, between model(239 aa) positions 1; 87 length is 88aa with 1 gaps, 0 stops, absolute frame= Plus1 at 470638..470901 in the same strand

Class B

27.
CDS YP_001514860.1 hypothetical protein intersects 32.17%(46aa) with IS1_ORF2 e-value= 3.3e-13 fragment hit coverage= 20.68%, between model(237 aa) positions 164; 212 length is 46aa with 3 gaps, 0 stops, absolute frame= Plus3 at 471168..471305 in the same strand
misc_feature no id IS1 transposase; Region: DDE_Tnp_IS1; cl00721 intersects 54.76%(46aa) with IS1_ORF2 e-value= 3.3e-13 fragment hit coverage= 20.68%, between model(237 aa) positions 164; 212 length is 46aa with 3 gaps, 0 stops, absolute frame= Plus3 at 471168..471305 in the same strand
Class A

28.
CDS YP_001514862.1 integrase catalytic subunit intersects 99.07%(212aa) with IS3_IS51_ORF2 e-value= 1.6e-14 complete sequence hit coverage= 100%, between model(317 aa) positions 1; 317 length is 291aa with 31 gaps, 3 stops, absolute frame= Minus3 at 471881..472752 in the same strand
misc_feature no id Integrase core domain; Region: rve_3; cl15866 intersects 100%(68aa) with IS3_IS51_ORF2 e-value= 1.6e-14 complete sequence hit coverage= 100%, between model(317 aa) positions 1; 317 length is 291aa with 31 gaps, 3 stops, absolute frame= Minus3 at 471881..472752 in the same strand
misc_feature no id DDE domain; Region: DDE_Tnp_IS240; pfam13610 intersects 100%(140aa) with IS3_IS51_ORF2 e-value= 1.6e-14 complete sequence hit coverage= 100%, between model(317 aa) positions 1; 317 length is 291aa with 31 gaps, 3 stops, absolute frame= Minus3 at 471881..472752 in the same strand
misc_feature no id Integrase core domain; Region: rve; cl01316 intersects 100%(110aa) with IS3_IS51_ORF2 e-value= 1.6e-14 complete sequence hit coverage= 100%, between model(317 aa) positions 1; 317 length is 291aa with 31 gaps, 3 stops, absolute frame= Minus3 at 471881..472752 in the same strand
CDS YP_001514863.1 hypothetical protein intersects 9.37%(40aa) with IS3_IS51_ORF2 e-value= 1.6e-14 complete sequence hit coverage= 100%, between model(317 aa) positions 1; 317 length is 291aa with 31 gaps, 3 stops, absolute frame= Minus3 at 471881..472752 in the opposite strand
CDS YP_001514862.1 integrase catalytic subunit intersects 97.66%(209aa) with IS3_IS51_ORF1 e-value= 9.6e-44 fragment hit coverage= 73.94%, between model(307 aa) positions 81; 307 length is 214aa with 13 gaps, 0 stops, absolute frame= Minus3 at 471890..472530 in the same strand
misc_feature no id Integrase core domain; Region: rve_3; cl15866 intersects 100%(68aa) with IS3_IS51_ORF1 e-value= 9.6e-44 fragment hit coverage= 73.94%, between model(307 aa) positions 81; 307 length is 214aa with 13 gaps, 0 stops, absolute frame= Minus3 at 471890..472530 in the same strand
misc_feature no id DDE domain; Region: DDE_Tnp_IS240; pfam13610 intersects 100%(140aa) with IS3_IS51_ORF1 e-value= 9.6e-44 fragment hit coverage= 73.94%, between model(307 aa) positions 81; 307 length is 214aa with 13 gaps, 0 stops, absolute frame= Minus3 at 471890..472530 in the same strand
misc_feature no id Integrase core domain; Region: rve; cl01316 intersects 100%(110aa) with IS3_IS51_ORF1 e-value= 9.6e-44 fragment hit coverage= 73.94%, between model(307 aa) positions 81; 307 length is 214aa with 13 gaps, 0 stops, absolute frame= Minus3 at 471890..472530 in the same strand
CDS YP_001514862.1 integrase catalytic subunit intersects 97.66%(209aa) with IS3_IS51_ORF1 e-value= 2.6e-25 complete sequence hit coverage= 100%, between model(307 aa) positions 1; 307 length is 299aa with 20 gaps, 4 stops, absolute frame= Minus3 at 471890..472785 in the same strand
misc_feature no id Integrase core domain; Region: rve_3; cl15866 intersects 100%(68aa) with IS3_IS51_ORF1 e-value= 2.6e-25 complete sequence hit coverage= 100%, between model(307 aa) positions 1; 307 length is 299aa with 20 gaps, 4 stops, absolute frame= Minus3 at 471890..472785 in the same strand
misc_feature no id DDE domain; Region: DDE_Tnp_IS240; pfam13610 intersects 100%(140aa) with IS3_IS51_ORF1 e-value= 2.6e-25 complete sequence hit coverage= 100%, between model(307 aa) positions 1; 307 length is 299aa with 20 gaps, 4 stops, absolute frame= Minus3 at 471890..472785 in the same strand
misc_feature no id Integrase core domain; Region: rve; cl01316 intersects 100%(110aa) with IS3_IS51_ORF1 e-value= 2.6e-25 complete sequence hit coverage= 100%, between model(307 aa) positions 1; 307 length is 299aa with 20 gaps, 4 stops, absolute frame= Minus3 at 471890..472785 in the same strand
CDS YP_001514863.1 hypothetical protein intersects 11.90%(51aa) with IS3_IS51_ORF1 e-value= 2.6e-25 complete sequence hit coverage= 100%, between model(307 aa) positions 1; 307 length is 299aa with 20 gaps, 4 stops, absolute frame= Minus3 at 471890..472785 in the opposite strand
CDS YP_001514862.1 integrase catalytic subunit intersects 97.66%(209aa) with IS3_IS407_ORF2 e-value= 9.2e-07 complete sequence hit coverage= 100%, between model(344 aa) positions 1; 344 length is 313aa with 42 gaps, 5 stops, absolute frame= Minus3 at 471890..472827 in the same strand

misc_feature	no id	Integrase core domain; Region: rve_3; cl15866 intersects 100%(68aa) with IS3_IS407_ORF2	e-value= 9.2e-07 complete sequence hit coverage= 100%, between model(344 aa) positions 1; 344 length is 313aa with 42 gaps, 5 stops, absolute frame= Minus3 at 471890..472827 in the same strand
misc_feature	no id	DDE domain; Region: DDE_Tnp_IS240; pfam13610 intersects 100%(140aa) with IS3_IS407_ORF2	e-value= 9.2e-07 complete sequence hit coverage= 100%, between model(344 aa) positions 1; 344 length is 313aa with 42 gaps, 5 stops, absolute frame= Minus3 at 471890..472827 in the same strand
misc_feature	no id	Integrase core domain; Region: rve; cl01316 intersects 100%(110aa) with IS3_IS407_ORF2	e-value= 9.2e-07 complete sequence hit coverage= 100%, between model(344 aa) positions 1; 344 length is 313aa with 42 gaps, 5 stops, absolute frame= Minus3 at 471890..472827 in the same strand
CDS	YP_001514863.1	hypothetical protein intersects 15.13%(65aa) with IS3_IS407_ORF2	e-value= 9.2e-07 complete sequence hit coverage= 100%, between model(344 aa) positions 1; 344 length is 313aa with 42 gaps, 5 stops, absolute frame= Minus3 at 471890..472827 in the opposite strand
CDS	YP_001514862.1	integrase catalytic subunit intersects 95.33%(204aa) with IS3_IS51_ORF2	e-value= 1.8e-35 fragment hit coverage= 67.51%, between model(317 aa) positions 96; 309 length is 205aa with 11 gaps, 0 stops, absolute frame= Minus3 at 471905..472518 in the same strand
misc_feature	no id	Integrase core domain; Region: rve_3; cl15866 intersects 100%(68aa) with IS3_IS51_ORF2	e-value= 1.8e-35 fragment hit coverage= 67.51%, between model(317 aa) positions 96; 309 length is 205aa with 11 gaps, 0 stops, absolute frame= Minus3 at 471905..472518 in the same strand
misc_feature	no id	DDE domain; Region: DDE_Tnp_IS240; pfam13610 intersects 100%(140aa) with IS3_IS51_ORF2	e-value= 1.8e-35 fragment hit coverage= 67.51%, between model(317 aa) positions 96; 309 length is 205aa with 11 gaps, 0 stops, absolute frame= Minus3 at 471905..472518 in the same strand
misc_feature	no id	Integrase core domain; Region: rve; cl01316 intersects 100%(110aa) with IS3_IS51_ORF2	e-value= 1.8e-35 fragment hit coverage= 67.51%, between model(317 aa) positions 96; 309 length is 205aa with 11 gaps, 0 stops, absolute frame= Minus3 at 471905..472518 in the same strand
CDS	YP_001514862.1	integrase catalytic subunit intersects 94.86%(203aa) with IS3_IS3_ORF2	e-value= 7.7e-58 fragment hit coverage= 76.74%, between model(288 aa) positions 68; 288 length is 211aa with 10 gaps, 0 stops, absolute frame= Minus3 at 471908..472539 in the same strand
misc_feature	no id	Integrase core domain; Region: rve_3; cl15866 intersects 100%(68aa) with IS3_IS3_ORF2	e-value= 7.7e-58 fragment hit coverage= 76.74%, between model(288 aa) positions 68; 288 length is 211aa with 10 gaps, 0 stops, absolute frame= Minus3 at 471908..472539 in the same strand
misc_feature	no id	DDE domain; Region: DDE_Tnp_IS240; pfam13610 intersects 100%(140aa) with IS3_IS3_ORF2	e-value= 7.7e-58 fragment hit coverage= 76.74%, between model(288 aa) positions 68; 288 length is 211aa with 10 gaps, 0 stops, absolute frame= Minus3 at 471908..472539 in the same strand
misc_feature	no id	Integrase core domain; Region: rve; cl01316 intersects 100%(110aa) with IS3_IS3_ORF2	e-value= 7.7e-58 fragment hit coverage= 76.74%, between model(288 aa) positions 68; 288 length is 211aa with 10 gaps, 0 stops, absolute frame= Minus3 at 471908..472539 in the same strand
CDS	YP_001514862.1	integrase catalytic subunit intersects 94.86%(203aa) with IS3_IS3_ORF2	e-value= 7.9e-44 complete sequence hit coverage= 100%, between model(288 aa) positions 1; 288 length is 277aa with 14 gaps, 3 stops, absolute frame= Minus3 at 471908..472737 in the same strand
misc_feature	no id	Integrase core domain; Region: rve_3; cl15866 intersects 100%(68aa) with IS3_IS3_ORF2	e-value= 7.9e-44 complete sequence hit coverage= 100%, between model(288 aa) positions 1; 288 length is 277aa with 14 gaps, 3 stops, absolute frame= Minus3 at 471908..472737 in the same strand
misc_feature	no id	DDE domain; Region: DDE_Tnp_IS240; pfam13610 intersects 100%(140aa) with IS3_IS3_ORF2	e-value= 7.9e-44 complete sequence hit coverage= 100%, between model(288 aa) positions 1; 288 length is 277aa with 14 gaps, 3 stops, absolute frame= Minus3 at 471908..472737 in the same strand
misc_feature	no id	Integrase core domain; Region: rve; cl01316 intersects 100%(110aa) with IS3_IS3_ORF2	e-value= 7.9e-44 complete sequence hit coverage= 100%, between model(288 aa) positions 1; 288 length is 277aa with 14 gaps, 3 stops, absolute frame= Minus3 at 471908..472737 in the same strand
CDS	YP_001514863.1	hypothetical protein intersects 8.22%(35aa) with IS3_IS3_ORF2	e-value= 7.9e-44 complete sequence hit coverage= 100%, between model(288 aa) positions 1; 288 length is 277aa with 14 gaps, 3 stops, absolute frame= Minus3 at 471908..472737 in the opposite strand
CDS	YP_001514862.1	integrase catalytic subunit intersects 93.93%(201aa) with IS3_IS150_ORF2	e-value= 3.3e-35 fragment hit coverage= 57.58%, between model(389 aa) positions 166; 389 length is 213aa with 14 gaps, 0 stops, absolute frame= Minus3 at 471914..472551 in the same strand
misc_feature	no id	Integrase core domain; Region: rve_3; cl15866 intersects 100%(68aa) with IS3_IS150_ORF2	e-value= 3.3e-35 fragment hit coverage= 57.58%, between model(389 aa) positions 166; 389 length is 213aa with 14 gaps, 0 stops, absolute frame= Minus3 at 471914..472551 in the same strand

misc_feature no id DDE domain; Region: DDE_Tnp_IS240; pfam13610 intersects 100%(140aa) with IS3_IS150_ORF2 e-value= 3.3e-35 fragment hit coverage= 57.58%, between model(389 aa) positions 166; 389 length is 213aa with 14 gaps, 0 stops, absolute frame= Minus3 at 471914..472551 in the same strand
misc_feature no id Integrase core domain; Region: rve; cl01316 intersects 100%(110aa) with IS3_IS150_ORF2 e-value= 3.3e-35 fragment hit coverage= 57.58%, between model(389 aa) positions 166; 389 length is 213aa with 14 gaps, 0 stops, absolute frame= Minus3 at 471914..472551 in the same strand
CDS YP_001514862.1 integrase catalytic subunit intersects 93.93%(201aa) with IS3_IS150_ORF2 e-value= 4.3e-13 complete sequence hit coverage= 100%, between model(389 aa) positions 1; 389 length is 351aa with 50 gaps, 5 stops, absolute frame= Minus3 at 471914..472965 in the same strand
misc_feature no id Integrase core domain; Region: rve_3; cl15866 intersects 100%(68aa) with IS3_IS150_ORF2 e-value= 4.3e-13 complete sequence hit coverage= 100%, between model(389 aa) positions 1; 389 length is 351aa with 50 gaps, 5 stops, absolute frame= Minus3 at 471914..472965 in the same strand
misc_feature no id DDE domain; Region: DDE_Tnp_IS240; pfam13610 intersects 100%(140aa) with IS3_IS150_ORF2 e-value= 4.3e-13 complete sequence hit coverage= 100%, between model(389 aa) positions 1; 389 length is 351aa with 50 gaps, 5 stops, absolute frame= Minus3 at 471914..472965 in the same strand
misc_feature no id Integrase core domain; Region: rve; cl01316 intersects 100%(110aa) with IS3_IS150_ORF2 e-value= 4.3e-13 complete sequence hit coverage= 100%, between model(389 aa) positions 1; 389 length is 351aa with 50 gaps, 5 stops, absolute frame= Minus3 at 471914..472965 in the same strand
CDS YP_001514863.1 hypothetical protein intersects 25.73%(111aa) with IS3_IS150_ORF2 e-value= 4.3e-13 complete sequence hit coverage= 100%, between model(389 aa) positions 1; 389 length is 351aa with 50 gaps, 5 stops, absolute frame= Minus3 at 471914..472965 in the opposite strand
CDS YP_001514862.1 integrase catalytic subunit intersects 93.46%(200aa) with IS3_IS150_ORF1 e-value= 6.7e-29 complete sequence hit coverage= 100%, between model(306 aa) positions 1; 306 length is 304aa with 13 gaps, 5 stops, absolute frame= Minus3 at 471917..472827 in the same strand
misc_feature no id Integrase core domain; Region: rve_3; cl15866 intersects 100%(68aa) with IS3_IS150_ORF1 e-value= 6.7e-29 complete sequence hit coverage= 100%, between model(306 aa) positions 1; 306 length is 304aa with 13 gaps, 5 stops, absolute frame= Minus3 at 471917..472827 in the same strand
misc_feature no id DDE domain; Region: DDE_Tnp_IS240; pfam13610 intersects 100%(140aa) with IS3_IS150_ORF1 e-value= 6.7e-29 complete sequence hit coverage= 100%, between model(306 aa) positions 1; 306 length is 304aa with 13 gaps, 5 stops, absolute frame= Minus3 at 471917..472827 in the same strand
misc_feature no id Integrase core domain; Region: rve; cl01316 intersects 100%(110aa) with IS3_IS150_ORF1 e-value= 6.7e-29 complete sequence hit coverage= 100%, between model(306 aa) positions 1; 306 length is 304aa with 13 gaps, 5 stops, absolute frame= Minus3 at 471917..472827 in the same strand
CDS YP_001514863.1 hypothetical protein intersects 15.13%(65aa) with IS3_IS150_ORF1 e-value= 6.7e-29 complete sequence hit coverage= 100%, between model(306 aa) positions 1; 306 length is 304aa with 13 gaps, 5 stops, absolute frame= Minus3 at 471917..472827 in the opposite strand
CDS YP_001514862.1 integrase catalytic subunit intersects 93.46%(200aa) with IS3_IS150_ORF1 e-value= 2.3e-40 fragment hit coverage= 72.55%, between model(306 aa) positions 85; 306 length is 211aa with 13 gaps, 0 stops, absolute frame= Minus3 at 471917..472548 in the same strand
misc_feature no id Integrase core domain; Region: rve_3; cl15866 intersects 100%(68aa) with IS3_IS150_ORF1 e-value= 2.3e-40 fragment hit coverage= 72.55%, between model(306 aa) positions 85; 306 length is 211aa with 13 gaps, 0 stops, absolute frame= Minus3 at 471917..472548 in the same strand
misc_feature no id DDE domain; Region: DDE_Tnp_IS240; pfam13610 intersects 100%(140aa) with IS3_IS150_ORF1 e-value= 2.3e-40 fragment hit coverage= 72.55%, between model(306 aa) positions 85; 306 length is 211aa with 13 gaps, 0 stops, absolute frame= Minus3 at 471917..472548 in the same strand
misc_feature no id Integrase core domain; Region: rve; cl01316 intersects 100%(110aa) with IS3_IS150_ORF1 e-value= 2.3e-40 fragment hit coverage= 72.55%, between model(306 aa) positions 85; 306 length is 211aa with 13 gaps, 0 stops, absolute frame= Minus3 at 471917..472548 in the same strand
CDS YP_001514862.1 integrase catalytic subunit intersects 92.99%(199aa) with IS3_IS3_ORF1 e-value= 1.8e-35 complete sequence hit coverage= 100%, between model(286 aa) positions 1; 286 length is 268aa with 19 gaps, 3 stops, absolute frame= Minus3 at 471920..472722 in the same strand
misc_feature no id Integrase core domain; Region: rve_3; cl15866 intersects 100%(68aa) with IS3_IS3_ORF1 e-value= 1.8e-35 complete sequence hit coverage= 100%, between model(286 aa) positions 1; 286 length is 268aa with 19 gaps, 3 stops, absolute frame= Minus3 at 471920..472722 in the same strand
misc_feature no id DDE domain; Region: DDE_Tnp_IS240; pfam13610 intersects 100%(140aa) with IS3_IS3_ORF1 e-value= 1.8e-35 complete sequence hit coverage= 100%, between model(286 aa) positions 1; 286 length is 268aa with 19 gaps, 3 stops, absolute frame= Minus3 at 471920..472722 in the same strand

misc_feature no id Integrase core domain; Region: rve; cl01316 intersects 100%(110aa) with IS3_IS3_ORF1 e-value= 1.8e-35 complete sequence hit coverage= 100%, between model(286 aa) positions 1; 286 length is 268aa with 19 gaps, 3 stops, absolute frame= Minus3 at 471920..472722 in the same strand
CDS YP_001514863.1 hypothetical protein intersects 7.07%(30aa) with IS3_IS3_ORF1 e-value= 1.8e-35 complete sequence hit coverage= 100%, between model(286 aa) positions 1; 286 length is 268aa with 19 gaps, 3 stops, absolute frame= Minus3 at 471920..472722 in the opposite strand
CDS YP_001514862.1 integrase catalytic subunit intersects 92.99%(199aa) with IS3_IS3_ORF1 e-value= 2.3e-45 fragment hit coverage= 75.52%, between model(286 aa) positions 71; 286 length is 210aa with 7 gaps, 0 stops, absolute frame= Minus3 at 471920..472548 in the same strand
misc_feature no id Integrase core domain; Region: rve_3; cl15866 intersects 100%(68aa) with IS3_IS3_ORF1 e-value= 2.3e-45 fragment hit coverage= 75.52%, between model(286 aa) positions 71; 286 length is 210aa with 7 gaps, 0 stops, absolute frame= Minus3 at 471920..472548 in the same strand
misc_feature no id DDE domain; Region: DDE_Tnp_IS240; pfam13610 intersects 100%(140aa) with IS3_IS3_ORF1 e-value= 2.3e-45 fragment hit coverage= 75.52%, between model(286 aa) positions 71; 286 length is 210aa with 7 gaps, 0 stops, absolute frame= Minus3 at 471920..472548 in the same strand
misc_feature no id Integrase core domain; Region: rve; cl01316 intersects 100%(110aa) with IS3_IS3_ORF1 e-value= 2.3e-45 fragment hit coverage= 75.52%, between model(286 aa) positions 71; 286 length is 210aa with 7 gaps, 0 stops, absolute frame= Minus3 at 471920..472548 in the same strand
Class A

29.
CDS YP_001514864.1 hypothetical protein intersects 74.55%(41aa) with IS3_IS407_ORF2 e-value= 1.2e-07 fragment hit coverage= 14.83%, between model(344 aa) positions 85; 135 length is 51aa with 0 gaps, 0 stops, absolute frame= Minus3 at 474032..474183 in the same strand
misc_feature no id HTH-like domain; Region: HTH_21; pfam13276 intersects 89.47%(17aa) with IS3_IS407_ORF2 e-value= 1.2e-07 fragment hit coverage= 14.83%, between model(344 aa) positions 85; 135 length is 51aa with 0 gaps, 0 stops, absolute frame= Minus3 at 474032..474183 in the same strand
CDS YP_001514864.1 hypothetical protein intersects 74.55%(41aa) with IS3_IS150_ORF2 e-value= 2.1e-06 fragment hit coverage= 17.48%, between model(389 aa) positions 103; 170 length is 59aa with 9 gaps, 1 stops, absolute frame= Minus3 at 474032..474207 in the same strand
misc_feature no id HTH-like domain; Region: HTH_21; pfam13276 intersects 89.47%(17aa) with IS3_IS150_ORF2 e-value= 2.1e-06 fragment hit coverage= 17.48%, between model(389 aa) positions 103; 170 length is 59aa with 9 gaps, 1 stops, absolute frame= Minus3 at 474032..474207 in the same strand
CDS YP_001514864.1 hypothetical protein intersects 68.48%(37aa) with IS3_IS2_ORF2 e-value= 9.4e-07 fragment hit coverage= 12.25%, between model(302 aa) positions 48; 84 length is 38aa with 0 gaps, 0 stops, absolute frame= Minus3 at 474035..474147 in the same strand
misc_feature no id HTH-like domain; Region: HTH_21; pfam13276 intersects 84.21%(16aa) with IS3_IS2_ORF2 e-value= 9.4e-07 fragment hit coverage= 12.25%, between model(302 aa) positions 48; 84 length is 38aa with 0 gaps, 0 stops, absolute frame= Minus3 at 474035..474147 in the same strand
Class B

30.
CDS YP_001514865.1 transposase-associated ATP-binding protein intersects 97.54%(238aa) with IS21_ORF2 e-value= 1.9e-80 complete sequence hit coverage= 100%, between model(258 aa) positions 1; 258 length is 255aa with 4 gaps, 0 stops, absolute frame= Minus2 at 474390..475153 in the same strand
misc_feature no id P-loop containing Nucleoside Triphosphate Hydrolases; Region: P-loop_NTPase; cl09099 intersects 100%(179aa) with IS21_ORF2 e-value= 1.9e-80 complete sequence hit coverage= 100%, between model(258 aa) positions 1; 258 length is 255aa with 4 gaps, 0 stops, absolute frame= Minus2 at 474390..475153 in the same strand
CDS YP_001514866.1 transposase intersects 12.84%(74aa) with IS21_ORF2 e-value= 1.9e-80 complete sequence hit coverage= 100%, between model(258 aa) positions 1; 258 length is 255aa with 4 gaps, 0 stops, absolute frame= Minus2 at 474390..475153 in the same strand
Class A

31.

CDS YP_001514866.1 transposase intersects 85.09%(492aa) with IS21_ORF1 e-value= 5e-20 complete sequence hit coverage= 100%, between model(525 aa) positions 1; 525 length is 493aa with 39 gaps, 0 stops, absolute frame= Minus1 at 475171..476648 in the same strand
misc_feature no id Integrase core domain; Region: rve; cl01316 intersects 100%(97aa) with IS21_ORF1 e-value= 5e-20 complete sequence hit coverage= 100%, between model(525 aa) positions 1; 525 length is 493aa with 39 gaps, 0 stops, absolute frame= Minus1 at 475171..476648 in the same strand
CDS YP_001514866.1 transposase intersects 47.78%(276aa) with IS21_ORF1 e-value= 3.2e-28 fragment hit coverage= 57.90%, between model(525 aa) positions 99; 402 length is 277aa with 28 gaps, 0 stops, absolute frame= Minus1 at 475549..476378 in the same strand
misc_feature no id Integrase core domain; Region: rve; cl01316 intersects 100%(97aa) with IS21_ORF1 e-value= 3.2e-28 fragment hit coverage= 57.90%, between model(525 aa) positions 99; 402 length is 277aa with 28 gaps, 0 stops, absolute frame= Minus1 at 475549..476378 in the same strand
Class A

32.
CDS YP_001514867.1 transposase intersects 78.68%(107aa) with IS630 e-value= 3e-16 fragment hit coverage= 50.80%, between model(376 aa) positions 140; 330 length is 165aa with 28 gaps, 0 stops, absolute frame= Minus3 at 477176..477669 in the same strand
misc_feature no id Integrase core domain; Region: rve; cl01316 intersects 88.33%(106aa) with IS630 e-value= 3e-16 fragment hit coverage= 50.80%, between model(376 aa) positions 140; 330 length is 165aa with 28 gaps, 0 stops, absolute frame= Minus3 at 477176..477669 in the same strand
CDS YP_001514868.1 transposase intersects 21.09%(27aa) with IS630 e-value= 3e-16 fragment hit coverage= 50.80%, between model(376 aa) positions 140; 330 length is 165aa with 28 gaps, 0 stops, absolute frame= Minus3 at 477176..477669 in the same strand
misc_feature no id Transposase; Region: HTH_Tnp_IS630; pfam01710 intersects 18.49%(22aa) with IS630 e-value= 3e-16 fragment hit coverage= 50.80%, between model(376 aa) positions 140; 330 length is 165aa with 28 gaps, 0 stops, absolute frame= Minus3 at 477176..477669 in the same strand
Class A

33.
CDS YP_001514871.1 hypothetical protein intersects 6.67%(3aa) with IS5_ISL2 e-value= 2.5e-62 fragment hit coverage= 51.54%, between model(260 aa) positions 127; 260 length is 134aa with 0 gaps, 0 stops, absolute frame= Minus1 at 480364..480764 in the opposite strand
CDS YP_001514872.1 hypothetical protein intersects 89.36%(42aa) with IS5_ISL2 e-value= 2.5e-62 fragment hit coverage= 51.54%, between model(260 aa) positions 127; 260 length is 134aa with 0 gaps, 0 stops, absolute frame= Minus1 at 480364..480764 in the same strand
gene no id fragment; similar to N-terminus of transposase intersects 11.54%(18aa) with IS5_ISL2 e-value= 2.5e-62 fragment hit coverage= 51.54%, between model(260 aa) positions 127; 260 length is 134aa with 0 gaps, 0 stops, absolute frame= Minus1 at 480364..480764 in the same strand
CDS YP_001514871.1 hypothetical protein intersects 6.67%(3aa) with IS5_ISL2 e-value= 1e-38 complete sequence hit coverage= 100%, between model(260 aa) positions 1; 260 length is 239aa with 23 gaps, 5 stops, absolute frame= Minus1 at 480364..481079 in the opposite strand
CDS YP_001514872.1 hypothetical protein intersects 89.36%(42aa) with IS5_ISL2 e-value= 1e-38 complete sequence hit coverage= 100%, between model(260 aa) positions 1; 260 length is 239aa with 23 gaps, 5 stops, absolute frame= Minus1 at 480364..481079 in the same strand
gene no id fragment; similar to N-terminus of transposase intersects 78.85%(123aa) with IS5_ISL2 e-value= 1e-38 complete sequence hit coverage= 100%, between model(260 aa) positions 1; 260 length is 239aa with 23 gaps, 5 stops, absolute frame= Minus1 at 480364..481079 in the same strand
CDS YP_001514872.1 hypothetical protein intersects 70.92%(33aa) with IS5_ISL2 e-value= 1.9e-21 complete sequence hit coverage= 100%, between model(260 aa) positions 1; 260 length is 246aa with 23 gaps, 5 stops, absolute frame= Minus2 at 480390..481126 in the same strand
gene no id fragment; similar to N-terminus of transposase intersects 88.89%(138aa) with IS5_ISL2 e-value= 1.9e-21 complete sequence hit coverage= 100%, between model(260 aa) positions 1; 260 length is 246aa with 23 gaps, 5 stops, absolute frame= Minus2 at 480390..481126 in the same strand
gene no id fragment; similar to N-terminus of transposase intersects 66.45%(103aa) with IS5_ISL2 e-value= 9.3e-44 fragment hit coverage= 40%, between model(260 aa) positions 26; 129 length is 104aa with 0 gaps, 0 stops, absolute frame= Minus2 at 480756..481066 in the same strand

Class A

34.
CDS YP_001514874.1 IS5 family transposase intersects 15.28%(80aa) with IS5_IS5 e-value= 2.6e-06 fragment hit coverage= 19.91%, between model(422 aa) positions 23; 106 length is 81aa with 3 gaps, 0 stops, absolute frame= Minus3 at 483299..483540 in the same strand
misc_feature no id Transposase domain (DUF772); Region: DUF772; pfam05598 intersects 74.03%(57aa) with IS5_IS5 e-value= 2.6e-06 fragment hit coverage= 19.91%, between model(422 aa) positions 23; 106 length is 81aa with 3 gaps, 0 stops, absolute frame= Minus3 at 483299..483540 in the same strand
Class A

35.
CDS YP_001514901.1 transposase intersects 86.04%(376aa) with IS4 e-value= 9.9e-07 complete sequence hit coverage= 100%, between model(486 aa) positions 1; 486 length is 376aa with 117 gaps, 0 stops, absolute frame= Plus3 at 516039..517166 in the same strand
misc_feature no id DDE superfamily endonuclease; Region: DDE_5; cl02413 intersects 93.57%(349aa) with IS4 e-value= 9.9e-07 complete sequence hit coverage= 100%, between model(486 aa) positions 1; 486 length is 376aa with 117 gaps, 0 stops, absolute frame= Plus3 at 516039..517166 in the same strand
CDS YP_001514901.1 transposase intersects 53.09%(232aa) with IS4 e-value= 4.7e-14 fragment hit coverage= 51.23%, between model(486 aa) positions 1; 249 length is 232aa with 27 gaps, 0 stops, absolute frame= Plus3 at 516039..516734 in the same strand
misc_feature no id DDE superfamily endonuclease; Region: DDE_5; cl02413 intersects 62.20%(232aa) with IS4 e-value= 4.7e-14 fragment hit coverage= 51.23%, between model(486 aa) positions 1; 249 length is 232aa with 27 gaps, 0 stops, absolute frame= Plus3 at 516039..516734 in the same strand
Class A

36.
gene no id transposase, putative; conserved hypothetical protein intersects 18.58%(65aa) with IS630 e-value= 1.4e-09 fragment hit coverage= 19.15%, between model(376 aa) positions 275; 346 length is 66aa with 6 gaps, 0 stops, absolute frame= Minus1 at 517393..517589 in the same strand
Class A

37.
CDS YP_001514904.1 IS5 family transposase intersects 14.48%(80aa) with IS5_IS5 e-value= 2.6e-06 fragment hit coverage= 19.91%, between model(422 aa) positions 23; 106 length is 81aa with 3 gaps, 0 stops, absolute frame= Minus2 at 522219..522460 in the same strand
misc_feature no id Transposase domain (DUF772); Region: DUF772; pfam05598 intersects 74.03%(57aa) with IS5_IS5 e-value= 2.6e-06 fragment hit coverage= 19.91%, between model(422 aa) positions 23; 106 length is 81aa with 3 gaps, 0 stops, absolute frame= Minus2 at 522219..522460 in the same strand
Class A

38.
CDS YP_001514933.1 hypothetical protein intersects 27.35%(45aa) with IS1_ORF2 e-value= 1.2e-12 fragment hit coverage= 20.68%, between model(237 aa) positions 164; 212 length is 46aa with 3 gaps, 0 stops, absolute frame= Minus2 at 557520..557656 in the same strand
misc_feature no id IS1 transposase; Region: DDE_Tnp_IS1; cl00721 intersects 49.10%(45aa) with IS1_ORF2 e-value= 1.2e-12 fragment hit coverage= 20.68%, between model(237 aa) positions 164; 212 length is 46aa with 3 gaps, 0 stops, absolute frame= Minus2 at 557520..557656 in the same strand
Class A

39.
CDS YP_001514934.1 hypothetical protein intersects 100%(54aa) with IS1_ORF1 e-value= 2.1e-12 fragment hit coverage= 34.31%, between model(239 aa) positions 6; 87 length is 83aa with 3 gaps, 0 stops, absolute frame= Minus3 at 557924..558171 in the opposite strand
Class B

40.

CDS YP_001515042.1 transposase intersects 53.09%(232aa) with IS4 e-value= 4.7e-14 fragment hit coverage= 51.23%, between model(486 aa) positions 1; 249 length is 232aa with 27 gaps, 0 stops, absolute frame= Plus3 at 666744..667439 in the same strand
misc_feature no id DDE superfamily endonuclease; Region: DDE_5; cl02413 intersects 62.20%(232aa) with IS4 e-value= 4.7e-14 fragment hit coverage= 51.23%, between model(486 aa) positions 1; 249 length is 232aa with 27 gaps, 0 stops, absolute frame= Plus3 at 666744..667439 in the same strand
CDS YP_001515042.1 transposase intersects 86.04%(376aa) with IS4 e-value= 9.9e-07 complete sequence hit coverage= 100%, between model(486 aa) positions 1; 486 length is 376aa with 117 gaps, 0 stops, absolute frame= Plus3 at 666744..667871 in the same strand
misc_feature no id DDE superfamily endonuclease; Region: DDE_5; cl02413 intersects 93.57%(349aa) with IS4 e-value= 9.9e-07 complete sequence hit coverage= 100%, between model(486 aa) positions 1; 486 length is 376aa with 117 gaps, 0 stops, absolute frame= Plus3 at 666744..667871 in the same strand
Class A

41.
CDS YP_001515045.1 IS1 transposase intersects 96.95%(127aa) with IS1_ORF2 e-value= 4.6e-13 complete sequence hit coverage= 100%, between model(237 aa) positions 1; 237 length is 213aa with 27 gaps, 3 stops, absolute frame= Minus2 at 668715..669352 in the same strand
misc_feature no id IS1 transposase; Region: DDE_Tnp_IS1; cl00721 intersects 100%(123aa) with IS1_ORF2 e-value= 4.6e-13 complete sequence hit coverage= 100%, between model(237 aa) positions 1; 237 length is 213aa with 27 gaps, 3 stops, absolute frame= Minus2 at 668715..669352 in the same strand
gene no id fragment; similar to N-terminus of transposase intersects 81.88%(84aa) with IS1_ORF2 e-value= 4.6e-13 complete sequence hit coverage= 100%, between model(237 aa) positions 1; 237 length is 213aa with 27 gaps, 3 stops, absolute frame= Minus2 at 668715..669352 in the same strand
CDS YP_001515045.1 IS1 transposase intersects 96.95%(127aa) with IS1_ORF2 e-value= 6.2e-32 fragment hit coverage= 54.43%, between model(237 aa) positions 109; 237 length is 129aa with 1 gaps, 0 stops, absolute frame= Minus2 at 668715..669100 in the same strand
misc_feature no id IS1 transposase; Region: DDE_Tnp_IS1; cl00721 intersects 100%(123aa) with IS1_ORF2 e-value= 6.2e-32 fragment hit coverage= 54.43%, between model(237 aa) positions 109; 237 length is 129aa with 1 gaps, 0 stops, absolute frame= Minus2 at 668715..669100 in the same strand
gene no id fragment; similar to N-terminus of transposase intersects 0.32%(0aa) with IS1_ORF2 e-value= 6.2e-32 fragment hit coverage= 54.43%, between model(237 aa) positions 109; 237 length is 129aa with 1 gaps, 0 stops, absolute frame= Minus2 at 668715..669100 in the same strand
CDS YP_001515045.1 IS1 transposase intersects 94.66%(124aa) with IS1_ORF1 e-value= 4.6e-14 fragment hit coverage= 53.97%, between model(239 aa) positions 111; 239 length is 126aa with 7 gaps, 0 stops, absolute frame= Minus2 at 668724..669100 in the same strand
misc_feature no id IS1 transposase; Region: DDE_Tnp_IS1; cl00721 intersects 100%(123aa) with IS1_ORF1 e-value= 4.6e-14 fragment hit coverage= 53.97%, between model(239 aa) positions 111; 239 length is 126aa with 7 gaps, 0 stops, absolute frame= Minus2 at 668724..669100 in the same strand
gene no id fragment; similar to N-terminus of transposase intersects 0.32%(0aa) with IS1_ORF1 e-value= 4.6e-14 fragment hit coverage= 53.97%, between model(239 aa) positions 111; 239 length is 126aa with 7 gaps, 0 stops, absolute frame= Minus2 at 668724..669100 in the same strand
gene no id fragment; similar to N-terminus of transposase intersects 67.96%(70aa) with IS1_ORF1 e-value= 3.6e-14 fragment hit coverage= 32.64%, between model(239 aa) positions 1; 78 length is 75aa with 3 gaps, 0 stops, absolute frame= Minus1 at 669199..669422 in the same strand
Class A

42.
misc_feature no id Transposase and inactivated derivatives [DNA replication, recombination, and repair]; Region: COG3677 intersects 83.81%(88aa) with IS1_ORF1 e-value= 1.7e-15 fragment hit coverage= 36.40%, between model(239 aa) positions 1; 87 length is 88aa with 2 gaps, 0 stops, absolute frame= Plus1 at 689875..690138 in the same strand
CDS YP_001515067.1 hypothetical protein intersects 67.18%(88aa) with IS1_ORF1 e-value= 1.7e-15 fragment hit coverage= 36.40%, between model(239 aa) positions 1; 87 length is 88aa with 2 gaps, 0 stops, absolute frame= Plus1 at 689875..690138 in the same strand
Class A

43.
CDS YP_001515068.1 hypothetical protein intersects 26.59%(46aa) with IS1_ORF2 e-value= 1.8e-11 fragment hit coverage= 20.68%, between model(237 aa) positions 164; 212 length is 46aa with 3 gaps, 0 stops, absolute frame= Plus3 at 690405..690542 in the same strand
misc_feature no id IS1 transposase; Region: DDE_Tnp_IS1; cl00721 intersects 49.46%(46aa) with IS1_ORF2 e-value= 1.8e-11 fragment hit coverage= 20.68%, between model(237 aa) positions 164; 212 length is 46aa with 3 gaps, 0 stops, absolute frame= Plus3 at 690405..690542 in the same strand
Class A

44.
CDS YP_001515068.1 hypothetical protein intersects 2.50%(4aa) with IS256 e-value= 9.9e-166 complete sequence hit coverage= 100%, between model(410 aa) positions 1; 410 length is 404aa with 11 gaps, 1 stops, absolute frame= Minus3 at 690725..691935 in the opposite strand
CDS YP_001515069.1 transposase mutator family protein intersects 96.32%(401aa) with IS256 e-value= 9.9e-166 complete sequence hit coverage= 100%, between model(410 aa) positions 1; 410 length is 404aa with 11 gaps, 1 stops, absolute frame= Minus3 at 690725..691935 in the same strand
misc_feature no id Transposase, Mutator family; Region: Transposase_mut; pfam00872 intersects 100%(362aa) with IS256 e-value= 9.9e-166 complete sequence hit coverage= 100%, between model(410 aa) positions 1; 410 length is 404aa with 11 gaps, 1 stops, absolute frame= Minus3 at 690725..691935 in the same strand
misc_feature no id MULE transposase domain; Region: MULE; pfam10551 intersects 100%(78aa) with IS256 e-value= 9.9e-166 complete sequence hit coverage= 100%, between model(410 aa) positions 1; 410 length is 404aa with 11 gaps, 1 stops, absolute frame= Minus3 at 690725..691935 in the same strand
Class A

45.
misc_feature no id Transposase and inactivated derivatives [DNA replication, recombination, and repair]; Region: COG3677 intersects 100%(81aa) with IS1_ORF1 e-value= 2.4e-40 complete sequence hit coverage= 100%, between model(239 aa) positions 1; 239 length is 225aa with 19 gaps, 1 stops, absolute frame= Plus2 at 720377..721051 in the same strand
CDS YP_001515100.1 IS1 transposase intersects 95.65%(220aa) with IS1_ORF1 e-value= 2.4e-40 complete sequence hit coverage= 100%, between model(239 aa) positions 1; 239 length is 225aa with 19 gaps, 1 stops, absolute frame= Plus2 at 720377..721051 in the same strand
misc_feature no id IS1 transposase; Region: DDE_Tnp_IS1; cl00721 intersects 100%(120aa) with IS1_ORF1 e-value= 2.4e-40 complete sequence hit coverage= 100%, between model(239 aa) positions 1; 239 length is 225aa with 19 gaps, 1 stops, absolute frame= Plus2 at 720377..721051 in the same strand
misc_feature no id Transposase and inactivated derivatives [DNA replication, recombination, and repair]; Region: COG3677 intersects 100%(81aa) with IS1_ORF2 e-value= 1.5e-28 complete sequence hit coverage= 100%, between model(237 aa) positions 1; 237 length is 227aa with 14 gaps, 1 stops, absolute frame= Plus2 at 720377..721057 in the same strand
CDS YP_001515100.1 IS1 transposase intersects 96.52%(222aa) with IS1_ORF2 e-value= 1.5e-28 complete sequence hit coverage= 100%, between model(237 aa) positions 1; 237 length is 227aa with 14 gaps, 1 stops, absolute frame= Plus2 at 720377..721057 in the same strand
misc_feature no id IS1 transposase; Region: DDE_Tnp_IS1; cl00721 intersects 100%(120aa) with IS1_ORF2 e-value= 1.5e-28 complete sequence hit coverage= 100%, between model(237 aa) positions 1; 237 length is 227aa with 14 gaps, 1 stops, absolute frame= Plus2 at 720377..721057 in the same strand
CDS YP_001515100.1 IS1 transposase intersects 58.26%(134aa) with IS1_ORF2 e-value= 3e-31 fragment hit coverage= 59.07%, between model(237 aa) positions 98; 237 length is 134aa with 6 gaps, 0 stops, absolute frame= Plus2 at 720656..721057 in the same strand
misc_feature no id IS1 transposase; Region: DDE_Tnp_IS1; cl00721 intersects 100%(120aa) with IS1_ORF2 e-value= 3e-31 fragment hit coverage= 59.07%, between model(237 aa) positions 98; 237 length is 134aa with 6 gaps, 0 stops, absolute frame= Plus2 at 720656..721057 in the same strand
Class A

46.
CDS YP_001515121.1 transposase intersects 41.19%(71aa) with IS630 e-value= 2.5e-08 fragment hit coverage= 20.74%, between model(376 aa) positions 44; 121 length is 72aa with 7 gaps, 0 stops, absolute frame= Minus1 at 741721..741935 in the same strand

misc_feature no id Winged helix-turn helix; Region: HTH_29; pfam13551 intersects 63.42%(71aa) with IS630 e-value= 2.5e-08 fragment hit coverage= 20.74%, between model(376 aa) positions 44; 121 length is 72aa with 7 gaps, 0 stops, absolute frame= Minus1 at 741721..741935 in the same strand
misc_feature no id Helix-turn-helix domains; Region: HTH; cl00088 intersects 75%(60aa) with IS630 e-value= 2.5e-08 fragment hit coverage= 20.74%, between model(376 aa) positions 44; 121 length is 72aa with 7 gaps, 0 stops, absolute frame= Minus1 at 741721..741935 in the same strand
misc_feature no id Helix-turn-helix domains; Region: HTH; cl00088 intersects 54.94%(29aa) with IS630 e-value= 2.5e-08 fragment hit coverage= 20.74%, between model(376 aa) positions 44; 121 length is 72aa with 7 gaps, 0 stops, absolute frame= Minus1 at 741721..741935 in the same strand
Class A

47.
CDS YP_001515128.1 aspartate kinase intersects 4.74%(28aa) with IS5_IS427 e-value= 2.8e-07 complete sequence hit coverage= 100%, between model(296 aa) positions 1; 296 length is 266aa with 39 gaps, 5 stops, absolute frame= Plus2 at 748838..749635 in the same strand
misc_feature no id aspartate kinase; Provisional; Region: PRK07431 intersects 4.58%(27aa) with IS5_IS427 e-value= 2.8e-07 complete sequence hit coverage= 100%, between model(296 aa) positions 1; 296 length is 266aa with 39 gaps, 5 stops, absolute frame= Plus2 at 748838..749635 in the same strand
misc_feature no id ACT domains of the lysine-sensitive, aspartokinase (AK) isoenzyme AKII of Bacillus subtilis (BS) strain 168 and related domains; Region: ACT_AKii-LysC-BS-like_2; cd04936 intersects 39.68%(25aa) with IS5_IS427 e-value= 2.8e-07 complete sequence hit coverage= 100%, between model(296 aa) positions 1; 296 length is 266aa with 39 gaps, 5 stops, absolute frame= Plus2 at 748838..749635 in the same strand
CDS YP_001515129.1 IS4 family transposase intersects 57.30%(153aa) with IS5_IS427 e-value= 2.8e-07 complete sequence hit coverage= 100%, between model(296 aa) positions 1; 296 length is 266aa with 39 gaps, 5 stops, absolute frame= Plus2 at 748838..749635 in the same strand
misc_feature no id DDE superfamily endonuclease; Region: DDE_4; cl15789 intersects 100%(75aa) with IS5_IS427 e-value= 2.8e-07 complete sequence hit coverage= 100%, between model(296 aa) positions 1; 296 length is 266aa with 39 gaps, 5 stops, absolute frame= Plus2 at 748838..749635 in the same strand
misc_feature no id Transposase DDE domain; Region: DDE_Tnp_1; pfam01609 intersects 31.61%(49aa) with IS5_IS427 e-value= 2.8e-07 complete sequence hit coverage= 100%, between model(296 aa) positions 1; 296 length is 266aa with 39 gaps, 5 stops, absolute frame= Plus2 at 748838..749635 in the same strand
CDS YP_001515129.1 IS4 family transposase intersects 100%(267aa) with IS5_IS5 e-value= 1.8e-09 complete sequence hit coverage= 100%, between model(422 aa) positions 1; 422 length is 290aa with 140 gaps, 4 stops, absolute frame= Plus2 at 749108..749977 in the same strand
misc_feature no id DDE superfamily endonuclease; Region: DDE_4; cl15789 intersects 100%(75aa) with IS5_IS5 e-value= 1.8e-09 complete sequence hit coverage= 100%, between model(422 aa) positions 1; 422 length is 290aa with 140 gaps, 4 stops, absolute frame= Plus2 at 749108..749977 in the same strand
misc_feature no id Transposase DDE domain; Region: DDE_Tnp_1; pfam01609 intersects 100%(155aa) with IS5_IS5 e-value= 1.8e-09 complete sequence hit coverage= 100%, between model(422 aa) positions 1; 422 length is 290aa with 140 gaps, 4 stops, absolute frame= Plus2 at 749108..749977 in the same strand
misc_feature no id DDE superfamily endonuclease; Region: DDE_4; cl15789 intersects 100%(83aa) with IS5_IS5 e-value= 1.8e-09 complete sequence hit coverage= 100%, between model(422 aa) positions 1; 422 length is 290aa with 140 gaps, 4 stops, absolute frame= Plus2 at 749108..749977 in the same strand
CDS YP_001515129.1 IS4 family transposase intersects 100%(267aa) with IS5_IS1031 e-value= 1.8e-111 complete sequence hit coverage= 100%, between model(295 aa) positions 1; 295 length is 284aa with 14 gaps, 3 stops, absolute frame= Plus2 at 749147..749998 in the same strand
misc_feature no id DDE superfamily endonuclease; Region: DDE_4; cl15789 intersects 100%(75aa) with IS5_IS1031 e-value= 1.8e-111 complete sequence hit coverage= 100%, between model(295 aa) positions 1; 295 length is 284aa with 14 gaps, 3 stops, absolute frame= Plus2 at 749147..749998 in the same strand
misc_feature no id Transposase DDE domain; Region: DDE_Tnp_1; pfam01609 intersects 100%(155aa) with IS5_IS1031 e-value= 1.8e-111 complete sequence hit coverage= 100%, between model(295 aa) positions 1; 295 length is 284aa with 14 gaps, 3 stops, absolute frame= Plus2 at 749147..749998 in the same strand
misc_feature no id DDE superfamily endonuclease; Region: DDE_4; cl15789 intersects 100%(83aa) with IS5_IS1031 e-value= 1.8e-111 complete sequence hit coverage= 100%, between model(295 aa) positions 1; 295 length is 284aa with 14 gaps, 3 stops, absolute frame= Plus2 at 749147..749998 in the same strand

CDS YP_001515129.1 IS4 family transposase intersects 98.50%(263aa) with IS5_IS1031 e-value= 1.2e-113 fragment hit coverage= 93.56%, between model(295 aa) positions 20; 295 length is 270aa with 9 gaps, 2 stops, absolute frame= Plus2 at 749189..749998 in the same strand
misc_feature no id DDE superfamily endonuclease; Region: DDE_4; cl15789 intersects 100%(75aa) with IS5_IS1031 e-value= 1.2e-113 fragment hit coverage= 93.56%, between model(295 aa) positions 20; 295 length is 270aa with 9 gaps, 2 stops, absolute frame= Plus2 at 749189..749998 in the same strand
misc_feature no id Transposase DDE domain; Region: DDE_Tnp_1; pfam01609 intersects 100%(155aa) with IS5_IS1031 e-value= 1.2e-113 fragment hit coverage= 93.56%, between model(295 aa) positions 20; 295 length is 270aa with 9 gaps, 2 stops, absolute frame= Plus2 at 749189..749998 in the same strand
misc_feature no id DDE superfamily endonuclease; Region: DDE_4; cl15789 intersects 100%(83aa) with IS5_IS1031 e-value= 1.2e-113 fragment hit coverage= 93.56%, between model(295 aa) positions 20; 295 length is 270aa with 9 gaps, 2 stops, absolute frame= Plus2 at 749189..749998 in the same strand
CDS YP_001515129.1 IS4 family transposase intersects 29.96%(80aa) with IS5_IS427 e-value= 2e-19 fragment hit coverage= 27.70%, between model(296 aa) positions 125; 206 length is 80aa with 2 gaps, 0 stops, absolute frame= Plus2 at 749198..749437 in the same strand
misc_feature no id DDE superfamily endonuclease; Region: DDE_4; cl15789 intersects 100%(75aa) with IS5_IS427 e-value= 2e-19 fragment hit coverage= 27.70%, between model(296 aa) positions 125; 206 length is 80aa with 2 gaps, 0 stops, absolute frame= Plus2 at 749198..749437 in the same strand
Class A

48.
CDS YP_001515172.1 IS5 family transposase intersects 15%(81aa) with IS5_IS5 e-value= 2.6e-06 fragment hit coverage= 19.91%, between model(422 aa) positions 23; 106 length is 81aa with 3 gaps, 0 stops, absolute frame= Plus2 at 791804..792046 in the same strand
misc_feature no id Transposase domain (DUF772); Region: DUF772; pfam05598 intersects 74.03%(57aa) with IS5_IS5 e-value= 2.6e-06 fragment hit coverage= 19.91%, between model(422 aa) positions 23; 106 length is 81aa with 3 gaps, 0 stops, absolute frame= Plus2 at 791804..792046 in the same strand
Class A

49.
CDS YP_001515203.1 hypothetical protein intersects 1.27%(2aa) with IS630 e-value= 8.8e-10 fragment hit coverage= 32.45%, between model(376 aa) positions 25; 146 length is 111aa with 11 gaps, 1 stops, absolute frame= Minus1 at 826267..826598 in the same strand
CDS YP_001515204.1 hypothetical protein intersects 80.05%(109aa) with IS630 e-value= 8.8e-10 fragment hit coverage= 32.45%, between model(376 aa) positions 25; 146 length is 111aa with 11 gaps, 1 stops, absolute frame= Minus1 at 826267..826598 in the same strand
misc_feature no id Winged helix-turn helix; Region: HTH_29; pfam13551 intersects 100%(104aa) with IS630 e-value= 8.8e-10 fragment hit coverage= 32.45%, between model(376 aa) positions 25; 146 length is 111aa with 11 gaps, 1 stops, absolute frame= Minus1 at 826267..826598 in the same strand
misc_feature no id Helix-turn-helix domains; Region: HTH; cl00088 intersects 100%(74aa) with IS630 e-value= 8.8e-10 fragment hit coverage= 32.45%, between model(376 aa) positions 25; 146 length is 111aa with 11 gaps, 1 stops, absolute frame= Minus1 at 826267..826598 in the same strand
misc_feature no id Helix-turn-helix domains; Region: HTH; cl00088 intersects 97.33%(48aa) with IS630 e-value= 8.8e-10 fragment hit coverage= 32.45%, between model(376 aa) positions 25; 146 length is 111aa with 11 gaps, 1 stops, absolute frame= Minus1 at 826267..826598 in the same strand
Class B

50.
CDS YP_001515229.1 transposase intersects 81.70%(83aa) with IS3_IS150_ORF2 e-value= 1.7e-22 complete sequence hit coverage= 100%, between model(389 aa) positions 1; 389 length is 354aa with 39 gaps, 5 stops, absolute frame= Plus1 at 850231..851292 in the same strand
misc_feature no id Helix-turn-helix domains; Region: HTH; cl00088 intersects 79.11%(59aa) with IS3_IS150_ORF2 e-value= 1.7e-22 complete sequence hit coverage= 100%, between model(389 aa) positions 1; 389 length is 354aa with 39 gaps, 5 stops, absolute frame= Plus1 at 850231..851292 in the same strand
CDS YP_001515230.1 integrase catalytic subunit intersects 95.45%(273aa) with IS3_IS150_ORF2 e-value= 1.7e-22 complete sequence hit coverage= 100%, between model(389 aa) positions 1; 389 length is 354aa with 39 gaps, 5 stops, absolute frame= Plus1 at 850231..851292 in the same strand

misc_feature no id putative transposase OrfB; Reviewed; Region: PHA02517 intersects 99.21%(252aa) with IS3_IS150_ORF2 e-value= 1.7e-22 complete sequence hit coverage= 100%, between model(389 aa) positions 1; 389 length is 354aa with 39 gaps, 5 stops, absolute frame= Plus1 at 850231..851292 in the same strand
misc_feature no id HTH-like domain; Region: HTH_21; pfam13276 intersects 100%(42aa) with IS3_IS150_ORF2 e-value= 1.7e-22 complete sequence hit coverage= 100%, between model(389 aa) positions 1; 389 length is 354aa with 39 gaps, 5 stops, absolute frame= Plus1 at 850231..851292 in the same strand
misc_feature no id Integrase core domain; Region: rve; cl01316 intersects 100%(110aa) with IS3_IS150_ORF2 e-value= 1.7e-22 complete sequence hit coverage= 100%, between model(389 aa) positions 1; 389 length is 354aa with 39 gaps, 5 stops, absolute frame= Plus1 at 850231..851292 in the same strand
misc_feature no id Integrase core domain; Region: rve_3; cl15866 intersects 100%(68aa) with IS3_IS150_ORF2 e-value= 1.7e-22 complete sequence hit coverage= 100%, between model(389 aa) positions 1; 389 length is 354aa with 39 gaps, 5 stops, absolute frame= Plus1 at 850231..851292 in the same strand
CDS YP_001515229.1 transposase intersects 68.95%(70aa) with IS3_IS407_ORF2 e-value= 6.3e-13 complete sequence hit coverage= 100%, between model(344 aa) positions 1; 344 length is 349aa with 10 gaps, 4 stops, absolute frame= Plus1 at 850270..851316 in the same strand
misc_feature no id Helix-turn-helix domains; Region: HTH; cl00088 intersects 61.78%(46aa) with IS3_IS407_ORF2 e-value= 6.3e-13 complete sequence hit coverage= 100%, between model(344 aa) positions 1; 344 length is 349aa with 10 gaps, 4 stops, absolute frame= Plus1 at 850270..851316 in the same strand
CDS YP_001515230.1 integrase catalytic subunit intersects 98.25%(281aa) with IS3_IS407_ORF2 e-value= 6.3e-13 complete sequence hit coverage= 100%, between model(344 aa) positions 1; 344 length is 349aa with 10 gaps, 4 stops, absolute frame= Plus1 at 850270..851316 in the same strand
misc_feature no id putative transposase OrfB; Reviewed; Region: PHA02517 intersects 100%(254aa) with IS3_IS407_ORF2 e-value= 6.3e-13 complete sequence hit coverage= 100%, between model(344 aa) positions 1; 344 length is 349aa with 10 gaps, 4 stops, absolute frame= Plus1 at 850270..851316 in the same strand
misc_feature no id HTH-like domain; Region: HTH_21; pfam13276 intersects 100%(42aa) with IS3_IS407_ORF2 e-value= 6.3e-13 complete sequence hit coverage= 100%, between model(344 aa) positions 1; 344 length is 349aa with 10 gaps, 4 stops, absolute frame= Plus1 at 850270..851316 in the same strand
misc_feature no id Integrase core domain; Region: rve; cl01316 intersects 100%(110aa) with IS3_IS407_ORF2 e-value= 6.3e-13 complete sequence hit coverage= 100%, between model(344 aa) positions 1; 344 length is 349aa with 10 gaps, 4 stops, absolute frame= Plus1 at 850270..851316 in the same strand
misc_feature no id Integrase core domain; Region: rve_3; cl15866 intersects 100%(68aa) with IS3_IS407_ORF2 e-value= 6.3e-13 complete sequence hit coverage= 100%, between model(344 aa) positions 1; 344 length is 349aa with 10 gaps, 4 stops, absolute frame= Plus1 at 850270..851316 in the same strand
CDS YP_001515229.1 transposase intersects 24.84%(25aa) with IS3_IS150_ORF1 e-value= 4.3e-49 complete sequence hit coverage= 100%, between model(306 aa) positions 1; 306 length is 295aa with 14 gaps, 0 stops, absolute frame= Plus1 at 850405..851289 in the same strand
misc_feature no id Helix-turn-helix domains; Region: HTH; cl00088 intersects 1.78%(1aa) with IS3_IS150_ORF1 e-value= 4.3e-49 complete sequence hit coverage= 100%, between model(306 aa) positions 1; 306 length is 295aa with 14 gaps, 0 stops, absolute frame= Plus1 at 850405..851289 in the same strand
CDS YP_001515230.1 integrase catalytic subunit intersects 95.10%(272aa) with IS3_IS150_ORF1 e-value= 4.3e-49 complete sequence hit coverage= 100%, between model(306 aa) positions 1; 306 length is 295aa with 14 gaps, 0 stops, absolute frame= Plus1 at 850405..851289 in the same strand
misc_feature no id putative transposase OrfB; Reviewed; Region: PHA02517 intersects 98.82%(251aa) with IS3_IS150_ORF1 e-value= 4.3e-49 complete sequence hit coverage= 100%, between model(306 aa) positions 1; 306 length is 295aa with 14 gaps, 0 stops, absolute frame= Plus1 at 850405..851289 in the same strand
misc_feature no id HTH-like domain; Region: HTH_21; pfam13276 intersects 100%(42aa) with IS3_IS150_ORF1 e-value= 4.3e-49 complete sequence hit coverage= 100%, between model(306 aa) positions 1; 306 length is 295aa with 14 gaps, 0 stops, absolute frame= Plus1 at 850405..851289 in the same strand
misc_feature no id Integrase core domain; Region: rve; cl01316 intersects 100%(110aa) with IS3_IS150_ORF1 e-value= 4.3e-49 complete sequence hit coverage= 100%, between model(306 aa) positions 1; 306 length is 295aa with 14 gaps, 0 stops, absolute frame= Plus1 at 850405..851289 in the same strand
misc_feature no id Integrase core domain; Region: rve_3; cl15866 intersects 100%(68aa) with IS3_IS150_ORF1 e-value= 4.3e-49 complete sequence hit coverage= 100%, between model(306 aa) positions 1; 306 length is 295aa with 14 gaps, 0 stops, absolute frame= Plus1 at 850405..851289 in the same strand
CDS YP_001515229.1 transposase intersects 13.07%(13aa) with IS3_IS51_ORF2 e-value= 1.6e-26 complete sequence hit coverage= 100%, between model(317 aa) positions 1; 317 length is 295aa with 27 gaps, 0 stops, absolute frame= Plus1 at 850441..851325 in the same strand

CDS	YP_001515230.1		integrase catalytic subunit intersects 99.30%(284aa) with IS3_IS51_ORF2	e-value= 1.6e-26 complete sequence hit coverage= 100%, between model(317 aa) positions 1; 317 length is 295aa with 27 gaps, 0 stops, absolute frame= Plus1 at 850441..851325 in the same strand
misc_feature	no id	putative transposase OrfB; Reviewed; Region: PHA02517 intersects 100%(254aa) with IS3_IS51_ORF2 e-value= 1.6e-26 complete sequence hit coverage= 100%, between model(317 aa) positions 1; 317 length is 295aa with 27 gaps, 0 stops, absolute frame= Plus1 at 850441..851325 in the same strand
misc_feature	no id	HTH-like domain; Region: HTH_21; pfam13276 intersects 100%(42aa) with IS3_IS51_ORF2 e-value= 1.6e-26 complete sequence hit coverage= 100%, between model(317 aa) positions 1; 317 length is 295aa with 27 gaps, 0 stops, absolute frame= Plus1 at 850441..851325 in the same strand
misc_feature	no id	Integrase core domain; Region: rve; cl01316 intersects 100%(110aa) with IS3_IS51_ORF2 e-value= 1.6e-26 complete sequence hit coverage= 100%, between model(317 aa) positions 1; 317 length is 295aa with 27 gaps, 0 stops, absolute frame= Plus1 at 850441..851325 in the same strand
misc_feature	no id	Integrase core domain; Region: rve_3; cl15866 intersects 100%(68aa) with IS3_IS51_ORF2 e-value= 1.6e-26 complete sequence hit coverage= 100%, between model(317 aa) positions 1; 317 length is 295aa with 27 gaps, 0 stops, absolute frame= Plus1 at 850441..851325 in the same strand
CDS	YP_001515229.1		transposase intersects 11.11%(11aa) with IS3_IS407_ORF2		e-value= 1.2e-17 fragment hit coverage= 25.29%, between model(344 aa) positions 60; 146 length is 85aa with 2 gaps, 0 stops, absolute frame= Plus1 at 850447..850701 in the same strand
CDS	YP_001515230.1		integrase catalytic subunit intersects 26.57%(76aa) with IS3_IS407_ORF2	e-value= 1.2e-17 fragment hit coverage= 25.29%, between model(344 aa) positions 60; 146 length is 85aa with 2 gaps, 0 stops, absolute frame= Plus1 at 850447..850701 in the same strand
misc_feature	no id	putative transposase OrfB; Reviewed; Region: PHA02517 intersects 21.65%(55aa) with IS3_IS407_ORF2		e-value= 1.2e-17 fragment hit coverage= 25.29%, between model(344 aa) positions 60; 146 length is 85aa with 2 gaps, 0 stops, absolute frame= Plus1 at 850447..850701 in the same strand
misc_feature	no id	HTH-like domain; Region: HTH_21; pfam13276 intersects 66.67%(28aa) with IS3_IS407_ORF2		e-value= 1.2e-17 fragment hit coverage= 25.29%, between model(344 aa) positions 60; 146 length is 85aa with 2 gaps, 0 stops, absolute frame= Plus1 at 850447..850701 in the same strand
CDS	YP_001515229.1		transposase intersects 10.13%(10aa) with IS3_IS2_ORF2	e-value= 2.2e-08 complete sequence hit coverage= 100%, between model(302 aa) positions 1; 302 length is 284aa with 31 gaps, 0 stops, absolute frame= Plus1 at 850450..851301 in the same strand
CDS	YP_001515230.1		integrase catalytic subunit intersects 96.50%(276aa) with IS3_IS2_ORF2	e-value= 2.2e-08 complete sequence hit coverage= 100%, between model(302 aa) positions 1; 302 length is 284aa with 31 gaps, 0 stops, absolute frame= Plus1 at 850450..851301 in the same strand
misc_feature	no id	putative transposase OrfB; Reviewed; Region: PHA02517 intersects 100%(254aa) with IS3_IS2_ORF2	e-value= 2.2e-08 complete sequence hit coverage= 100%, between model(302 aa) positions 1; 302 length is 284aa with 31 gaps, 0 stops, absolute frame= Plus1 at 850450..851301 in the same strand
misc_feature	no id	HTH-like domain; Region: HTH_21; pfam13276 intersects 100%(42aa) with IS3_IS2_ORF2 e-value= 2.2e-08 complete sequence hit coverage= 100%, between model(302 aa) positions 1; 302 length is 284aa with 31 gaps, 0 stops, absolute frame= Plus1 at 850450..851301 in the same strand
misc_feature	no id	Integrase core domain; Region: rve; cl01316 intersects 100%(110aa) with IS3_IS2_ORF2 e-value= 2.2e-08 complete sequence hit coverage= 100%, between model(302 aa) positions 1; 302 length is 284aa with 31 gaps, 0 stops, absolute frame= Plus1 at 850450..851301 in the same strand
misc_feature	no id	Integrase core domain; Region: rve_3; cl15866 intersects 100%(68aa) with IS3_IS2_ORF2 e-value= 2.2e-08 complete sequence hit coverage= 100%, between model(302 aa) positions 1; 302 length is 284aa with 31 gaps, 0 stops, absolute frame= Plus1 at 850450..851301 in the same strand
CDS	YP_001515229.1		transposase intersects 5.23%(5aa) with IS3_IS150_ORF2	e-value= 2.2e-47 fragment hit coverage= 76.09%, between model(389 aa) positions 94; 389 length is 276aa with 23 gaps, 0 stops, absolute frame= Plus1 at 850465..851292 in the same strand
CDS	YP_001515230.1		integrase catalytic subunit intersects 95.45%(273aa) with IS3_IS150_ORF2	e-value= 2.2e-47 fragment hit coverage= 76.09%, between model(389 aa) positions 94; 389 length is 276aa with 23 gaps, 0 stops, absolute frame= Plus1 at 850465..851292 in the same strand
misc_feature	no id	putative transposase OrfB; Reviewed; Region: PHA02517 intersects 99.21%(252aa) with IS3_IS150_ORF2		e-value= 2.2e-47 fragment hit coverage= 76.09%, between model(389 aa) positions 94; 389 length is 276aa with 23 gaps, 0 stops, absolute frame= Plus1 at 850465..851292 in the same strand
misc_feature	no id	HTH-like domain; Region: HTH_21; pfam13276 intersects 100%(42aa) with IS3_IS150_ORF2		e-value= 2.2e-47 fragment hit coverage= 76.09%, between model(389 aa) positions 94; 389 length is 276aa with 23 gaps, 0 stops, absolute frame= Plus1 at 850465..851292 in the same strand

misc_feature no id Integrase core domain; Region: rve; cl01316 intersects 100%(110aa) with IS3_IS150_ORF2 e-value= 2.2e-47 fragment hit coverage= 76.09%, between model(389 aa) positions 94; 389 length is 276aa with 23 gaps, 0 stops, absolute frame= Plus1 at 850465..851292 in the same strand
misc_feature no id Integrase core domain; Region: rve_3; cl15866 intersects 100%(68aa) with IS3_IS150_ORF2 e-value= 2.2e-47 fragment hit coverage= 76.09%, between model(389 aa) positions 94; 389 length is 276aa with 23 gaps, 0 stops, absolute frame= Plus1 at 850465..851292 in the same strand
CDS YP_001515229.1 transposase intersects 4.25%(4aa) with IS3_IS51_ORF1 e-value= 8.1e-53 complete sequence hit coverage= 100%, between model(307 aa) positions 1; 307 length is 283aa with 25 gaps, 0 stops, absolute frame= Plus1 at 850468..851316 in the same strand
CDS YP_001515230.1 integrase catalytic subunit intersects 98.25%(281aa) with IS3_IS51_ORF1 e-value= 8.1e-53 complete sequence hit coverage= 100%, between model(307 aa) positions 1; 307 length is 283aa with 25 gaps, 0 stops, absolute frame= Plus1 at 850468..851316 in the same strand
misc_feature no id putative transposase OrfB; Reviewed; Region: PHA02517 intersects 100%(254aa) with IS3_IS51_ORF1 e-value= 8.1e-53 complete sequence hit coverage= 100%, between model(307 aa) positions 1; 307 length is 283aa with 25 gaps, 0 stops, absolute frame= Plus1 at 850468..851316 in the same strand
misc_feature no id HTH-like domain; Region: HTH_21; pfam13276 intersects 100%(42aa) with IS3_IS51_ORF1 e-value= 8.1e-53 complete sequence hit coverage= 100%, between model(307 aa) positions 1; 307 length is 283aa with 25 gaps, 0 stops, absolute frame= Plus1 at 850468..851316 in the same strand
misc_feature no id Integrase core domain; Region: rve; cl01316 intersects 100%(110aa) with IS3_IS51_ORF1 e-value= 8.1e-53 complete sequence hit coverage= 100%, between model(307 aa) positions 1; 307 length is 283aa with 25 gaps, 0 stops, absolute frame= Plus1 at 850468..851316 in the same strand
misc_feature no id Integrase core domain; Region: rve_3; cl15866 intersects 100%(68aa) with IS3_IS51_ORF1 e-value= 8.1e-53 complete sequence hit coverage= 100%, between model(307 aa) positions 1; 307 length is 283aa with 25 gaps, 0 stops, absolute frame= Plus1 at 850468..851316 in the same strand
CDS YP_001515229.1 transposase intersects 3.27%(3aa) with IS3_IS3_ORF1 e-value= 2.5e-54 complete sequence hit coverage= 100%, between model(286 aa) positions 1; 286 length is 272aa with 15 gaps, 0 stops, absolute frame= Plus1 at 850471..851286 in the same strand
CDS YP_001515230.1 integrase catalytic subunit intersects 94.76%(271aa) with IS3_IS3_ORF1 e-value= 2.5e-54 complete sequence hit coverage= 100%, between model(286 aa) positions 1; 286 length is 272aa with 15 gaps, 0 stops, absolute frame= Plus1 at 850471..851286 in the same strand
misc_feature no id putative transposase OrfB; Reviewed; Region: PHA02517 intersects 98.43%(250aa) with IS3_IS3_ORF1 e-value= 2.5e-54 complete sequence hit coverage= 100%, between model(286 aa) positions 1; 286 length is 272aa with 15 gaps, 0 stops, absolute frame= Plus1 at 850471..851286 in the same strand
misc_feature no id HTH-like domain; Region: HTH_21; pfam13276 intersects 100%(42aa) with IS3_IS3_ORF1 e-value= 2.5e-54 complete sequence hit coverage= 100%, between model(286 aa) positions 1; 286 length is 272aa with 15 gaps, 0 stops, absolute frame= Plus1 at 850471..851286 in the same strand
misc_feature no id Integrase core domain; Region: rve; cl01316 intersects 100%(110aa) with IS3_IS3_ORF1 e-value= 2.5e-54 complete sequence hit coverage= 100%, between model(286 aa) positions 1; 286 length is 272aa with 15 gaps, 0 stops, absolute frame= Plus1 at 850471..851286 in the same strand
misc_feature no id Integrase core domain; Region: rve_3; cl15866 intersects 100%(68aa) with IS3_IS3_ORF1 e-value= 2.5e-54 complete sequence hit coverage= 100%, between model(286 aa) positions 1; 286 length is 272aa with 15 gaps, 0 stops, absolute frame= Plus1 at 850471..851286 in the same strand
CDS YP_001515229.1 transposase intersects 2.29%(2aa) with IS3_IS150_ORF1 e-value= 6.3e-50 fragment hit coverage= 92.81%, between model(306 aa) positions 23; 306 length is 272aa with 14 gaps, 0 stops, absolute frame= Plus1 at 850474..851289 in the same strand
CDS YP_001515230.1 integrase catalytic subunit intersects 95.10%(272aa) with IS3_IS150_ORF1 e-value= 6.3e-50 fragment hit coverage= 92.81%, between model(306 aa) positions 23; 306 length is 272aa with 14 gaps, 0 stops, absolute frame= Plus1 at 850474..851289 in the same strand
misc_feature no id putative transposase OrfB; Reviewed; Region: PHA02517 intersects 98.82%(251aa) with IS3_IS150_ORF1 e-value= 6.3e-50 fragment hit coverage= 92.81%, between model(306 aa) positions 23; 306 length is 272aa with 14 gaps, 0 stops, absolute frame= Plus1 at 850474..851289 in the same strand
misc_feature no id HTH-like domain; Region: HTH_21; pfam13276 intersects 100%(42aa) with IS3_IS150_ORF1 e-value= 6.3e-50 fragment hit coverage= 92.81%, between model(306 aa) positions 23; 306 length is 272aa with 14 gaps, 0 stops, absolute frame= Plus1 at 850474..851289 in the same strand
misc_feature no id Integrase core domain; Region: rve; cl01316 intersects 100%(110aa) with IS3_IS150_ORF1 e-value= 6.3e-50 fragment hit coverage= 92.81%, between model(306 aa) positions 23; 306 length is 272aa with 14 gaps, 0 stops, absolute frame= Plus1 at 850474..851289 in the same strand

misc_feature no id Integrase core domain; Region: rve_3; cl15866 intersects 100%(68aa) with IS3_IS150_ORF1 e-value= 6.3e-50 fragment hit coverage= 92.81%, between model(306 aa) positions 23; 306 length is 272aa with 14 gaps, 0 stops, absolute frame= Plus1 at 850474..851289 in the same strand
CDS YP_001515230.1 integrase catalytic subunit intersects 95.10%(272aa) with IS3_IS3_ORF2 e-value= 3.3e-65 complete sequence hit coverage= 100%, between model(288 aa) positions 1; 288 length is 272aa with 19 gaps, 0 stops, absolute frame= Plus1 at 850483..851298 in the same strand
misc_feature no id putative transposase OrfB; Reviewed; Region: PHA02517 intersects 100%(254aa) with IS3_IS3_ORF2 e-value= 3.3e-65 complete sequence hit coverage= 100%, between model(288 aa) positions 1; 288 length is 272aa with 19 gaps, 0 stops, absolute frame= Plus1 at 850483..851298 in the same strand
misc_feature no id HTH-like domain; Region: HTH_21; pfam13276 intersects 100%(42aa) with IS3_IS3_ORF2 e-value= 3.3e-65 complete sequence hit coverage= 100%, between model(288 aa) positions 1; 288 length is 272aa with 19 gaps, 0 stops, absolute frame= Plus1 at 850483..851298 in the same strand
misc_feature no id Integrase core domain; Region: rve; cl01316 intersects 100%(110aa) with IS3_IS3_ORF2 e-value= 3.3e-65 complete sequence hit coverage= 100%, between model(288 aa) positions 1; 288 length is 272aa with 19 gaps, 0 stops, absolute frame= Plus1 at 850483..851298 in the same strand
misc_feature no id Integrase core domain; Region: rve_3; cl15866 intersects 100%(68aa) with IS3_IS3_ORF2 e-value= 3.3e-65 complete sequence hit coverage= 100%, between model(288 aa) positions 1; 288 length is 272aa with 19 gaps, 0 stops, absolute frame= Plus1 at 850483..851298 in the same strand
CDS YP_001515230.1 integrase catalytic subunit intersects 18.53%(53aa) with IS3_IS2_ORF2 e-value= 9.1e-09 fragment hit coverage= 18.87%, between model(302 aa) positions 48; 104 length is 53aa with 5 gaps, 0 stops, absolute frame= Plus1 at 850552..850710 in the same strand
misc_feature no id putative transposase OrfB; Reviewed; Region: PHA02517 intersects 20.87%(53aa) with IS3_IS2_ORF2 e-value= 9.1e-09 fragment hit coverage= 18.87%, between model(302 aa) positions 48; 104 length is 53aa with 5 gaps, 0 stops, absolute frame= Plus1 at 850552..850710 in the same strand
misc_feature no id HTH-like domain; Region: HTH_21; pfam13276 intersects 73.81%(31aa) with IS3_IS2_ORF2 e-value= 9.1e-09 fragment hit coverage= 18.87%, between model(302 aa) positions 48; 104 length is 53aa with 5 gaps, 0 stops, absolute frame= Plus1 at 850552..850710 in the same strand
CDS YP_001515230.1 integrase catalytic subunit intersects 79.72%(228aa) with IS3_IS51_ORF2 e-value= 1.9e-38 fragment hit coverage= 74.76%, between model(317 aa) positions 73; 309 length is 228aa with 11 gaps, 0 stops, absolute frame= Plus1 at 850618..851301 in the same strand
misc_feature no id putative transposase OrfB; Reviewed; Region: PHA02517 intersects 89.37%(227aa) with IS3_IS51_ORF2 e-value= 1.9e-38 fragment hit coverage= 74.76%, between model(317 aa) positions 73; 309 length is 228aa with 11 gaps, 0 stops, absolute frame= Plus1 at 850618..851301 in the same strand
misc_feature no id HTH-like domain; Region: HTH_21; pfam13276 intersects 100%(42aa) with IS3_IS51_ORF2 e-value= 1.9e-38 fragment hit coverage= 74.76%, between model(317 aa) positions 73; 309 length is 228aa with 11 gaps, 0 stops, absolute frame= Plus1 at 850618..851301 in the same strand
misc_feature no id Integrase core domain; Region: rve; cl01316 intersects 100%(110aa) with IS3_IS51_ORF2 e-value= 1.9e-38 fragment hit coverage= 74.76%, between model(317 aa) positions 73; 309 length is 228aa with 11 gaps, 0 stops, absolute frame= Plus1 at 850618..851301 in the same strand
misc_feature no id Integrase core domain; Region: rve_3; cl15866 intersects 100%(68aa) with IS3_IS51_ORF2 e-value= 1.9e-38 fragment hit coverage= 74.76%, between model(317 aa) positions 73; 309 length is 228aa with 11 gaps, 0 stops, absolute frame= Plus1 at 850618..851301 in the same strand
Class A

51.
CDS YP_001515321.1 transposase intersects 86.04%(376aa) with IS4 e-value= 9.9e-07 complete sequence hit coverage= 100%, between model(486 aa) positions 1; 486 length is 376aa with 117 gaps, 0 stops, absolute frame= Plus2 at 940328..941455 in the same strand
misc_feature no id DDE superfamily endonuclease; Region: DDE_5; cl02413 intersects 93.57%(349aa) with IS4 e-value= 9.9e-07 complete sequence hit coverage= 100%, between model(486 aa) positions 1; 486 length is 376aa with 117 gaps, 0 stops, absolute frame= Plus2 at 940328..941455 in the same strand
CDS YP_001515321.1 transposase intersects 53.09%(232aa) with IS4 e-value= 4.7e-14 fragment hit coverage= 51.23%, between model(486 aa) positions 1; 249 length is 232aa with 27 gaps, 0 stops, absolute frame= Plus2 at 940328..941023 in the same strand
misc_feature no id DDE superfamily endonuclease; Region: DDE_5; cl02413 intersects 62.20%(232aa) with IS4 e-value= 4.7e-14 fragment hit coverage= 51.23%, between model(486 aa) positions 1; 249 length is 232aa with 27 gaps, 0 stops, absolute frame= Plus2 at 940328..941023 in the same strand

Class A

52.
CDS YP_001515322.1 transposase intersects 47.43%(129aa) with IS630 e-value= 1.1e-13 fragment hit coverage= 38.83%, between model(376 aa) positions 188; 333 length is 129aa with 20 gaps, 0 stops, absolute frame= Plus2 at 942041..942427 in the same strand
misc_feature no id Integrase core domain; Region: rve; cl01316 intersects 90.21%(129aa) with IS630 e-value= 1.1e-13 fragment hit coverage= 38.83%, between model(376 aa) positions 188; 333 length is 129aa with 20 gaps, 0 stops, absolute frame= Plus2 at 942041..942427 in the same strand
Class A

53.
CDS YP_001515324.1 IS4 family transposase intersects 99.35%(308aa) with IS5_IS903 e-value= 1.7e-97 complete sequence hit coverage= 100%, between model(347 aa) positions 1; 347 length is 314aa with 37 gaps, 1 stops, absolute frame= Plus1 at 943111..944052 in the same strand
misc_feature no id Transposase DDE domain; Region: DDE_Tnp_1_5; pfam13737 intersects 100%(112aa) with IS5_IS903 e-value= 1.7e-97 complete sequence hit coverage= 100%, between model(347 aa) positions 1; 347 length is 314aa with 37 gaps, 1 stops, absolute frame= Plus1 at 943111..944052 in the same strand
misc_feature no id Transposase DDE domain; Region: DDE_Tnp_1; pfam01609 intersects 100%(186aa) with IS5_IS903 e-value= 1.7e-97 complete sequence hit coverage= 100%, between model(347 aa) positions 1; 347 length is 314aa with 37 gaps, 1 stops, absolute frame= Plus1 at 943111..944052 in the same strand
Class A

54.
CDS YP_001515354.1 hypothetical protein intersects 16.60%(44aa) with IS5_IS427 e-value= 8.6e-07 complete sequence hit coverage= 100%, between model(296 aa) positions 1; 296 length is 259aa with 44 gaps, 10 stops, absolute frame= Plus3 at 972294..973070 in the opposite strand
CDS YP_001515355.1 IS4 family transposase intersects 57.30%(153aa) with IS5_IS427 e-value= 8.6e-07 complete sequence hit coverage= 100%, between model(296 aa) positions 1; 296 length is 259aa with 44 gaps, 10 stops, absolute frame= Plus3 at 972294..973070 in the same strand
misc_feature no id DDE superfamily endonuclease; Region: DDE_4; cl15789 intersects 100%(75aa) with IS5_IS427 e-value= 8.6e-07 complete sequence hit coverage= 100%, between model(296 aa) positions 1; 296 length is 259aa with 44 gaps, 10 stops, absolute frame= Plus3 at 972294..973070 in the same strand
misc_feature no id Transposase DDE domain; Region: DDE_Tnp_1; pfam01609 intersects 31.61%(49aa) with IS5_IS427 e-value= 8.6e-07 complete sequence hit coverage= 100%, between model(296 aa) positions 1; 296 length is 259aa with 44 gaps, 10 stops, absolute frame= Plus3 at 972294..973070 in the same strand
CDS YP_001515355.1 IS4 family transposase intersects 100%(267aa) with IS5_IS5 e-value= 1.8e-09 complete sequence hit coverage= 100%, between model(422 aa) positions 1; 422 length is 290aa with 140 gaps, 4 stops, absolute frame= Plus3 at 972543..973412 in the same strand
misc_feature no id DDE superfamily endonuclease; Region: DDE_4; cl15789 intersects 100%(75aa) with IS5_IS5 e-value= 1.8e-09 complete sequence hit coverage= 100%, between model(422 aa) positions 1; 422 length is 290aa with 140 gaps, 4 stops, absolute frame= Plus3 at 972543..973412 in the same strand
misc_feature no id Transposase DDE domain; Region: DDE_Tnp_1; pfam01609 intersects 100%(155aa) with IS5_IS5 e-value= 1.8e-09 complete sequence hit coverage= 100%, between model(422 aa) positions 1; 422 length is 290aa with 140 gaps, 4 stops, absolute frame= Plus3 at 972543..973412 in the same strand
misc_feature no id DDE superfamily endonuclease; Region: DDE_4; cl15789 intersects 100%(83aa) with IS5_IS5 e-value= 1.8e-09 complete sequence hit coverage= 100%, between model(422 aa) positions 1; 422 length is 290aa with 140 gaps, 4 stops, absolute frame= Plus3 at 972543..973412 in the same strand
CDS YP_001515355.1 IS4 family transposase intersects 100%(267aa) with IS5_IS1031 e-value= 1.8e-111 complete sequence hit coverage= 100%, between model(295 aa) positions 1; 295 length is 284aa with 14 gaps, 3 stops, absolute frame= Plus3 at 972582..973433 in the same strand
misc_feature no id DDE superfamily endonuclease; Region: DDE_4; cl15789 intersects 100%(75aa) with IS5_IS1031 e-value= 1.8e-111 complete sequence hit coverage= 100%, between model(295 aa) positions 1; 295 length is 284aa with 14 gaps, 3 stops, absolute frame= Plus3 at 972582..973433 in the same strand
misc_feature no id Transposase DDE domain; Region: DDE_Tnp_1; pfam01609 intersects 100%(155aa) with IS5_IS1031 e-value= 1.8e-111 complete sequence hit coverage= 100%, between model(295 aa) positions 1; 295 length is 284aa with 14 gaps, 3 stops, absolute frame= Plus3 at 972582..973433 in the same strand

misc_feature no id DDE superfamily endonuclease; Region: DDE_4; cl15789 intersects 100%(83aa) with IS5_IS1031 e-value= 1.8e-111 complete sequence hit coverage= 100%, between model(295 aa) positions 1; 295 length is 284aa with 14 gaps, 3 stops, absolute frame= Plus3 at 972582..973433 in the same strand
CDS YP_001515356.1 hypothetical protein intersects 6.41%(5aa) with IS5_IS1031e-value= 1.8e-111 complete sequence hit coverage= 100%, between model(295 aa) positions 1; 295 length is 284aa with 14 gaps, 3 stops, absolute frame= Plus3 at 972582..973433 in the opposite strand
CDS YP_001515355.1 IS4 family transposase intersects 98.50%(263aa) with IS5_IS1031 e-value= 1.2e-113 fragment hit coverage= 93.56%, between model(295 aa) positions 20; 295 length is 270aa with 9 gaps, 2 stops, absolute frame= Plus3 at 972624..973433 in the same strand
misc_feature no id DDE superfamily endonuclease; Region: DDE_4; cl15789 intersects 100%(75aa) with IS5_IS1031 e-value= 1.2e-113 fragment hit coverage= 93.56%, between model(295 aa) positions 20; 295 length is 270aa with 9 gaps, 2 stops, absolute frame= Plus3 at 972624..973433 in the same strand
misc_feature no id Transposase DDE domain; Region: DDE_Tnp_1; pfam01609 intersects 100%(155aa) with IS5_IS1031 e-value= 1.2e-113 fragment hit coverage= 93.56%, between model(295 aa) positions 20; 295 length is 270aa with 9 gaps, 2 stops, absolute frame= Plus3 at 972624..973433 in the same strand
misc_feature no id DDE superfamily endonuclease; Region: DDE_4; cl15789 intersects 100%(83aa) with IS5_IS1031 e-value= 1.2e-113 fragment hit coverage= 93.56%, between model(295 aa) positions 20; 295 length is 270aa with 9 gaps, 2 stops, absolute frame= Plus3 at 972624..973433 in the same strand
CDS YP_001515356.1 hypothetical protein intersects 6.41%(5aa) with IS5_IS1031e-value= 1.2e-113 fragment hit coverage= 93.56%, between model(295 aa) positions 20; 295 length is 270aa with 9 gaps, 2 stops, absolute frame= Plus3 at 972624..973433 in the opposite strand
CDS YP_001515355.1 IS4 family transposase intersects 29.96%(80aa) with IS5_IS427 e-value= 2e-19 fragment hit coverage= 27.70%, between model(296 aa) positions 125; 206 length is 80aa with 2 gaps, 0 stops, absolute frame= Plus3 at 972633..972872 in the same strand
misc_feature no id DDE superfamily endonuclease; Region: DDE_4; cl15789 intersects 100%(75aa) with IS5_IS427 e-value= 2e-19 fragment hit coverage= 27.70%, between model(296 aa) positions 125; 206 length is 80aa with 2 gaps, 0 stops, absolute frame= Plus3 at 972633..972872 in the same strand
Class A

55.
CDS YP_001515392.1 transposase IS66 intersects 7.63%(69aa) with IS66_ORF2 e-value= 1e-08 fragment hit coverage= 16.26%, between model(535 aa) positions 243; 329 length is 69aa with 18 gaps, 0 stops, absolute frame= Plus2 at 1009808..1010014 in the same strand
misc_feature no id Integrase core domain; Region: rve; cl01316 intersects 27.38%(69aa) with IS66_ORF2 e-value= 1e-08 fragment hit coverage= 16.26%, between model(535 aa) positions 243; 329 length is 69aa with 18 gaps, 0 stops, absolute frame= Plus2 at 1009808..1010014 in the same strand
CDS YP_001515392.1 transposase IS66 intersects 6.31%(57aa) with IS66_ORF3 e-value= 3.6e-07 fragment hit coverage= 12.72%, between model(558 aa) positions 287; 357 length is 57aa with 14 gaps, 0 stops, absolute frame= Plus2 at 1009844..1010014 in the same strand
misc_feature no id Integrase core domain; Region: rve; cl01316 intersects 22.62%(57aa) with IS66_ORF3 e-value= 3.6e-07 fragment hit coverage= 12.72%, between model(558 aa) positions 287; 357 length is 57aa with 14 gaps, 0 stops, absolute frame= Plus2 at 1009844..1010014 in the same strand
Class A

56.
CDS YP_001515405.1 hypothetical protein intersects 93.51%(346aa) with IS630 e-value= 3.5e-21 complete sequence hit coverage= 100%, between model(376 aa) positions 1; 376 length is 346aa with 33 gaps, 0 stops, absolute frame= Plus3 at 1016775..1017812 in the same strand
misc_feature no id Helix-turn-helix domains; Region: HTH; cl00088 intersects 100%(50aa) with IS630 e-value= 3.5e-21 complete sequence hit coverage= 100%, between model(376 aa) positions 1; 376 length is 346aa with 33 gaps, 0 stops, absolute frame= Plus3 at 1016775..1017812 in the same strand
misc_feature no id Winged helix-turn helix; Region: HTH_29; pfam13551 intersects 100%(102aa) with IS630 e-value= 3.5e-21 complete sequence hit coverage= 100%, between model(376 aa) positions 1; 376 length is 346aa with 33 gaps, 0 stops, absolute frame= Plus3 at 1016775..1017812 in the same strand
misc_feature no id Winged helix-turn helix; Region: HTH_33; pfam13592 intersects 100%(56aa) with IS630 e-value= 3.5e-21 complete sequence hit coverage= 100%, between model(376 aa) positions 1; 376 length is 346aa with 33 gaps, 0 stops, absolute frame= Plus3 at 1016775..1017812 in the same strand

misc_feature no id Integrase core domain; Region: rve; cl01316 intersects 100%(141aa) with IS630 e-value= 3.5e-21 complete sequence hit coverage= 100%, between model(376 aa) positions 1; 376 length is 346aa with 33 gaps, 0 stops, absolute frame= Plus3 at 1016775..1017812 in the same strand
CDS YP_001515405.1 hypothetical protein intersects 14.32%(53aa) with IS481 e-value= 3.7e-09 fragment hit coverage= 15.10%, between model(351 aa) positions 1; 53 length is 53aa with 0 gaps, 0 stops, absolute frame= Plus3 at 1016835..1016993 in the same strand
misc_feature no id Helix-turn-helix domains; Region: HTH; cl00088 intersects 100%(50aa) with IS481 e-value= 3.7e-09 fragment hit coverage= 15.10%, between model(351 aa) positions 1; 53 length is 53aa with 0 gaps, 0 stops, absolute frame= Plus3 at 1016835..1016993 in the same strand
misc_feature no id Winged helix-turn helix; Region: HTH_29; pfam13551 intersects 43.14%(44aa) with IS481 e-value= 3.7e-09 fragment hit coverage= 15.10%, between model(351 aa) positions 1; 53 length is 53aa with 0 gaps, 0 stops, absolute frame= Plus3 at 1016835..1016993 in the same strand
Class A

57.
CDS YP_001515477.1 transposase intersects 81.65%(129aa) with IS630 e-value= 1.1e-13 fragment hit coverage= 38.83%, between model(376 aa) positions 188; 333 length is 129aa with 20 gaps, 0 stops, absolute frame= Plus3 at 1099857..1100243 in the same strand
misc_feature no id Integrase core domain; Region: rve; cl01316 intersects 90.21%(129aa) with IS630 e-value= 1.1e-13 fragment hit coverage= 38.83%, between model(376 aa) positions 188; 333 length is 129aa with 20 gaps, 0 stops, absolute frame= Plus3 at 1099857..1100243 in the same strand
Class A

58.
CDS YP_001515478.1 transposase intersects 53.09%(232aa) with IS4 e-value= 4.7e-14 fragment hit coverage= 51.23%, between model(486 aa) positions 1; 249 length is 232aa with 27 gaps, 0 stops, absolute frame= Plus3 at 1100496..1101191 in the same strand
misc_feature no id DDE superfamily endonuclease; Region: DDE_5; cl02413 intersects 62.20%(232aa) with IS4 e-value= 4.7e-14 fragment hit coverage= 51.23%, between model(486 aa) positions 1; 249 length is 232aa with 27 gaps, 0 stops, absolute frame= Plus3 at 1100496..1101191 in the same strand
CDS YP_001515478.1 transposase intersects 86.04%(376aa) with IS4 e-value= 9.9e-07 complete sequence hit coverage= 100%, between model(486 aa) positions 1; 486 length is 376aa with 117 gaps, 0 stops, absolute frame= Plus3 at 1100496..1101623 in the same strand
misc_feature no id DDE superfamily endonuclease; Region: DDE_5; cl02413 intersects 93.57%(349aa) with IS4 e-value= 9.9e-07 complete sequence hit coverage= 100%, between model(486 aa) positions 1; 486 length is 376aa with 117 gaps, 0 stops, absolute frame= Plus3 at 1100496..1101623 in the same strand
Class A

59.
CDS YP_001515503.1 hypothetical protein intersects 100%(39aa) with IS5_ISL2 e-value= 6.5e-76 complete sequence hit coverage= 100%, between model(260 aa) positions 1; 260 length is 256aa with 12 gaps, 5 stops, absolute frame= Minus1 at 1118947..1119713 in the same strand
gene no id fragment; similar to N-terminus of transposase intersects 100%(76aa) with IS5_ISL2 e-value= 6.5e-76 complete sequence hit coverage= 100%, between model(260 aa) positions 1; 260 length is 256aa with 12 gaps, 5 stops, absolute frame= Minus1 at 1118947..1119713 in the same strand
CDS YP_001515503.1 hypothetical protein intersects 100%(39aa) with IS5_ISL2 e-value= 1.1e-77 fragment hit coverage= 90.38%, between model(260 aa) positions 26; 260 length is 228aa with 10 gaps, 4 stops, absolute frame= Minus1 at 1118947..1119629 in the same strand
gene no id fragment; similar to N-terminus of transposase intersects 95.61%(72aa) with IS5_ISL2 e-value= 1.1e-77 fragment hit coverage= 90.38%, between model(260 aa) positions 26; 260 length is 228aa with 10 gaps, 4 stops, absolute frame= Minus1 at 1118947..1119629 in the same strand
CDS YP_001515503.1 hypothetical protein intersects 61.54%(24aa) with IS5_ISL2 e-value= 1.8e-17 fragment hit coverage= 19.23%, between model(260 aa) positions 80; 129 length is 50aa with 0 gaps, 0 stops, absolute frame= Minus2 at 1119321..1119469 in the same strand
gene no id fragment; similar to N-terminus of transposase intersects 25.44%(19aa) with IS5_ISL2 e-value= 1.8e-17 fragment hit coverage= 19.23%, between model(260 aa) positions 80; 129 length is 50aa with 0 gaps, 0 stops, absolute frame= Minus2 at 1119321..1119469 in the same strand

Class A

60.
CDS YP_001515555.1 hypothetical protein intersects 93.42%(345aa) with IS630 e-value= 3.1e-22 complete sequence hit coverage= 100%, between model(376 aa) positions 1; 376 length is 346aa with 33 gaps, 0 stops, absolute frame= Minus1 at 1183279..1184315 in the same strand
misc_feature no id Integrase core domain; Region: rve; cl01316 intersects 100%(141aa) with IS630 e-value= 3.1e-22 complete sequence hit coverage= 100%, between model(376 aa) positions 1; 376 length is 346aa with 33 gaps, 0 stops, absolute frame= Minus1 at 1183279..1184315 in the same strand
misc_feature no id Winged helix-turn helix; Region: HTH_33; pfam13592 intersects 100%(56aa) with IS630 e-value= 3.1e-22 complete sequence hit coverage= 100%, between model(376 aa) positions 1; 376 length is 346aa with 33 gaps, 0 stops, absolute frame= Minus1 at 1183279..1184315 in the same strand
misc_feature no id Winged helix-turn helix; Region: HTH_29; pfam13551 intersects 100%(102aa) with IS630 e-value= 3.1e-22 complete sequence hit coverage= 100%, between model(376 aa) positions 1; 376 length is 346aa with 33 gaps, 0 stops, absolute frame= Minus1 at 1183279..1184315 in the same strand
misc_feature no id Helix-turn-helix domains; Region: HTH; cl00088 intersects 100%(50aa) with IS630 e-value= 3.1e-22 complete sequence hit coverage= 100%, between model(376 aa) positions 1; 376 length is 346aa with 33 gaps, 0 stops, absolute frame= Minus1 at 1183279..1184315 in the same strand
CDS YP_001515555.1 hypothetical protein intersects 14.23%(52aa) with IS481 e-value= 3.7e-09 fragment hit coverage= 15.10%, between model(351 aa) positions 1; 53 length is 53aa with 0 gaps, 0 stops, absolute frame= Minus1 at 1184098..1184255 in the same strand
misc_feature no id Winged helix-turn helix; Region: HTH_29; pfam13551 intersects 43.14%(44aa) with IS481 e-value= 3.7e-09 fragment hit coverage= 15.10%, between model(351 aa) positions 1; 53 length is 53aa with 0 gaps, 0 stops, absolute frame= Minus1 at 1184098..1184255 in the same strand
misc_feature no id Helix-turn-helix domains; Region: HTH; cl00088 intersects 100%(50aa) with IS481 e-value= 3.7e-09 fragment hit coverage= 15.10%, between model(351 aa) positions 1; 53 length is 53aa with 0 gaps, 0 stops, absolute frame= Minus1 at 1184098..1184255 in the same strand
Class A

61.
CDS YP_001515585.1 IS1 transposase intersects 95.40%(166aa) with IS1_ORF2 e-value= 4.1e-24 complete sequence hit coverage= 100%, between model(237 aa) positions 1; 237 length is 228aa with 13 gaps, 1 stops, absolute frame= Plus3 at 1211556..1212239 in the same strand
misc_feature no id IS1 transposase; Region: DDE_Tnp_IS1; cl00721 intersects 100%(120aa) with IS1_ORF2 e-value= 4.1e-24 complete sequence hit coverage= 100%, between model(237 aa) positions 1; 237 length is 228aa with 13 gaps, 1 stops, absolute frame= Plus3 at 1211556..1212239 in the same strand
CDS YP_001515586.1 hypothetical protein intersects 5.22%(4aa) with IS1_ORF2 e-value= 4.1e-24 complete sequence hit coverage= 100%, between model(237 aa) positions 1; 237 length is 228aa with 13 gaps, 1 stops, absolute frame= Plus3 at 1211556..1212239 in the opposite strand
CDS YP_001515585.1 IS1 transposase intersects 94.25%(164aa) with IS1_ORF1 e-value= 5.6e-36 complete sequence hit coverage= 100%, between model(239 aa) positions 1; 239 length is 216aa with 28 gaps, 0 stops, absolute frame= Plus3 at 1211586..1212233 in the same strand
misc_feature no id IS1 transposase; Region: DDE_Tnp_IS1; cl00721 intersects 100%(120aa) with IS1_ORF1 e-value= 5.6e-36 complete sequence hit coverage= 100%, between model(239 aa) positions 1; 239 length is 216aa with 28 gaps, 0 stops, absolute frame= Plus3 at 1211586..1212233 in the same strand
CDS YP_001515586.1 hypothetical protein intersects 2.81%(2aa) with IS1_ORF1 e-value= 5.6e-36 complete sequence hit coverage= 100%, between model(239 aa) positions 1; 239 length is 216aa with 28 gaps, 0 stops, absolute frame= Plus3 at 1211586..1212233 in the opposite strand
CDS YP_001515585.1 IS1 transposase intersects 77.01%(134aa) with IS1_ORF2 e-value= 1.9e-31 fragment hit coverage= 59.07%, between model(237 aa) positions 98; 237 length is 134aa with 6 gaps, 0 stops, absolute frame= Plus3 at 1211838..1212239 in the same strand
misc_feature no id IS1 transposase; Region: DDE_Tnp_IS1; cl00721 intersects 100%(120aa) with IS1_ORF2 e-value= 1.9e-31 fragment hit coverage= 59.07%, between model(237 aa) positions 98; 237 length is 134aa with 6 gaps, 0 stops, absolute frame= Plus3 at 1211838..1212239 in the same strand
CDS YP_001515586.1 hypothetical protein intersects 5.22%(4aa) with IS1_ORF2 e-value= 1.9e-31 fragment hit coverage= 59.07%, between model(237 aa) positions 98; 237 length is 134aa with 6 gaps, 0 stops, absolute frame= Plus3 at 1211838..1212239 in the opposite strand

Class A

62.
CDS YP_001515590.1 transposase intersects 59.84%(125aa) with IS630 e-value= 2.3e-07 fragment hit coverage= 32.45%, between model(376 aa) positions 255; 376 length is 126aa with 2 gaps, 0 stops, absolute frame= Minus1 at 1225171..1225547 in the same strand
misc_feature no id Integrase core domain; Region: rve; cl01316 intersects 61.87%(94aa) with IS630 e-value= 2.3e-07 fragment hit coverage= 32.45%, between model(376 aa) positions 255; 376 length is 126aa with 2 gaps, 0 stops, absolute frame= Minus1 at 1225171..1225547 in the same strand
Class A

63.
CDS YP_001515591.1 transposase intersects 78%(114aa) with IS630 e-value= 1.2e-15 fragment hit coverage= 32.18%, between model(376 aa) positions 1; 121 length is 115aa with 7 gaps, 0 stops, absolute frame= Minus2 at 1225905..1226248 in the same strand
misc_feature no id Winged helix-turn helix; Region: HTH_29; pfam13551 intersects 79.63%(86aa) with IS630 e-value= 1.2e-15 fragment hit coverage= 32.18%, between model(376 aa) positions 1; 121 length is 115aa with 7 gaps, 0 stops, absolute frame= Minus2 at 1225905..1226248 in the same strand
misc_feature no id Helix-turn-helix domains; Region: HTH; cl00088 intersects 75%(60aa) with IS630 e-value= 1.2e-15 fragment hit coverage= 32.18%, between model(376 aa) positions 1; 121 length is 115aa with 7 gaps, 0 stops, absolute frame= Minus2 at 1225905..1226248 in the same strand
misc_feature no id Helix-turn-helix domains; Region: HTH; cl00088 intersects 100%(47aa) with IS630 e-value= 1.2e-15 fragment hit coverage= 32.18%, between model(376 aa) positions 1; 121 length is 115aa with 7 gaps, 0 stops, absolute frame= Minus2 at 1225905..1226248 in the same strand
Class A

64.
CDS YP_001515662.1 transposase intersects 81.65%(129aa) with IS630 e-value= 1.1e-13 fragment hit coverage= 38.83%, between model(376 aa) positions 188; 333 length is 129aa with 20 gaps, 0 stops, absolute frame= Plus1 at 1300873..1301259 in the same strand
misc_feature no id Integrase core domain; Region: rve; cl01316 intersects 90.21%(129aa) with IS630 e-value= 1.1e-13 fragment hit coverage= 38.83%, between model(376 aa) positions 188; 333 length is 129aa with 20 gaps, 0 stops, absolute frame= Plus1 at 1300873..1301259 in the same strand
Class A

65.
CDS YP_001515678.1 hypothetical protein intersects 93.51%(346aa) with IS630 e-value= 4.9e-24 complete sequence hit coverage= 100%, between model(376 aa) positions 1; 376 length is 346aa with 33 gaps, 0 stops, absolute frame= Plus2 at 1313054..1314091 in the same strand
misc_feature no id Helix-turn-helix domains; Region: HTH; cl00088 intersects 100%(50aa) with IS630 e-value= 4.9e-24 complete sequence hit coverage= 100%, between model(376 aa) positions 1; 376 length is 346aa with 33 gaps, 0 stops, absolute frame= Plus2 at 1313054..1314091 in the same strand
misc_feature no id Winged helix-turn helix; Region: HTH_29; pfam13551 intersects 100%(102aa) with IS630 e-value= 4.9e-24 complete sequence hit coverage= 100%, between model(376 aa) positions 1; 376 length is 346aa with 33 gaps, 0 stops, absolute frame= Plus2 at 1313054..1314091 in the same strand
misc_feature no id Winged helix-turn helix; Region: HTH_33; pfam13592 intersects 100%(56aa) with IS630 e-value= 4.9e-24 complete sequence hit coverage= 100%, between model(376 aa) positions 1; 376 length is 346aa with 33 gaps, 0 stops, absolute frame= Plus2 at 1313054..1314091 in the same strand
misc_feature no id Integrase core domain; Region: rve; cl01316 intersects 100%(141aa) with IS630 e-value= 4.9e-24 complete sequence hit coverage= 100%, between model(376 aa) positions 1; 376 length is 346aa with 33 gaps, 0 stops, absolute frame= Plus2 at 1313054..1314091 in the same strand
CDS YP_001515678.1 hypothetical protein intersects 14.32%(53aa) with IS481 e-value= 3.7e-09 fragment hit coverage= 15.10%, between model(351 aa) positions 1; 53 length is 53aa with 0 gaps, 0 stops, absolute frame= Plus2 at 1313114..1313272 in the same strand
misc_feature no id Helix-turn-helix domains; Region: HTH; cl00088 intersects 100%(50aa) with IS481 e-value= 3.7e-09 fragment hit coverage= 15.10%, between model(351 aa) positions 1; 53 length is 53aa with 0 gaps, 0 stops, absolute frame= Plus2 at 1313114..1313272 in the same strand

misc_feature no id Winged helix-turn helix; Region: HTH_29; pfam13551 intersects 43.14%(44aa) with IS481 e-value= 3.7e-09 fragment hit coverage= 15.10%, between model(351 aa) positions 1; 53 length is 53aa with 0 gaps, 0 stops, absolute frame= Plus2 at 1313114..1313272 in the same strand
Class A

66.
CDS YP_001515704.1 IS4 family transposase intersects 100%(261aa) with IS5_IS5 e-value= 6.1e-15 complete sequence hit coverage= 100%, between model(422 aa) positions 1; 422 length is 280aa with 142 gaps, 2 stops, absolute frame= Plus1 at 1334545..1335384 in the same strand
misc_feature no id DDE superfamily endonuclease; Region: DDE_4; cl15789 intersects 100%(75aa) with IS5_IS5 e-value= 6.1e-15 complete sequence hit coverage= 100%, between model(422 aa) positions 1; 422 length is 280aa with 142 gaps, 2 stops, absolute frame= Plus1 at 1334545..1335384 in the same strand
misc_feature no id Transposase DDE domain; Region: DDE_Tnp_1; pfam01609 intersects 100%(157aa) with IS5_IS5 e-value= 6.1e-15 complete sequence hit coverage= 100%, between model(422 aa) positions 1; 422 length is 280aa with 142 gaps, 2 stops, absolute frame= Plus1 at 1334545..1335384 in the same strand
misc_feature no id DDE superfamily endonuclease; Region: DDE_4; cl15789 intersects 100%(53aa) with IS5_IS5 e-value= 6.1e-15 complete sequence hit coverage= 100%, between model(422 aa) positions 1; 422 length is 280aa with 142 gaps, 2 stops, absolute frame= Plus1 at 1334545..1335384 in the same strand
CDS YP_001515704.1 IS4 family transposase intersects 100%(261aa) with IS5_IS1031 e-value= 2.7e-109 complete sequence hit coverage= 100%, between model(295 aa) positions 1; 295 length is 287aa with 10 gaps, 2 stops, absolute frame= Plus1 at 1334545..1335405 in the same strand
misc_feature no id DDE superfamily endonuclease; Region: DDE_4; cl15789 intersects 100%(75aa) with IS5_IS1031 e-value= 2.7e-109 complete sequence hit coverage= 100%, between model(295 aa) positions 1; 295 length is 287aa with 10 gaps, 2 stops, absolute frame= Plus1 at 1334545..1335405 in the same strand
misc_feature no id Transposase DDE domain; Region: DDE_Tnp_1; pfam01609 intersects 100%(157aa) with IS5_IS1031 e-value= 2.7e-109 complete sequence hit coverage= 100%, between model(295 aa) positions 1; 295 length is 287aa with 10 gaps, 2 stops, absolute frame= Plus1 at 1334545..1335405 in the same strand
misc_feature no id DDE superfamily endonuclease; Region: DDE_4; cl15789 intersects 100%(53aa) with IS5_IS1031 e-value= 2.7e-109 complete sequence hit coverage= 100%, between model(295 aa) positions 1; 295 length is 287aa with 10 gaps, 2 stops, absolute frame= Plus1 at 1334545..1335405 in the same strand
CDS YP_001515704.1 IS4 family transposase intersects 99.23%(259aa) with IS5_IS1031 e-value= 3.5e-110 fragment hit coverage= 91.53%, between model(295 aa) positions 17; 286 length is 260aa with 10 gaps, 1 stops, absolute frame= Plus1 at 1334599..1335378 in the same strand
misc_feature no id DDE superfamily endonuclease; Region: DDE_4; cl15789 intersects 100%(75aa) with IS5_IS1031 e-value= 3.5e-110 fragment hit coverage= 91.53%, between model(295 aa) positions 17; 286 length is 260aa with 10 gaps, 1 stops, absolute frame= Plus1 at 1334599..1335378 in the same strand
misc_feature no id Transposase DDE domain; Region: DDE_Tnp_1; pfam01609 intersects 100%(157aa) with IS5_IS1031 e-value= 3.5e-110 fragment hit coverage= 91.53%, between model(295 aa) positions 17; 286 length is 260aa with 10 gaps, 1 stops, absolute frame= Plus1 at 1334599..1335378 in the same strand
misc_feature no id DDE superfamily endonuclease; Region: DDE_4; cl15789 intersects 100%(53aa) with IS5_IS1031 e-value= 3.5e-110 fragment hit coverage= 91.53%, between model(295 aa) positions 17; 286 length is 260aa with 10 gaps, 1 stops, absolute frame= Plus1 at 1334599..1335378 in the same strand
CDS YP_001515704.1 IS4 family transposase intersects 29.89%(78aa) with IS5_IS427 e-value= 7.9e-16 fragment hit coverage= 27.03%, between model(296 aa) positions 129; 208 length is 78aa with 2 gaps, 0 stops, absolute frame= Plus1 at 1334629..1334862 in the same strand
misc_feature no id DDE superfamily endonuclease; Region: DDE_4; cl15789 intersects 96%(72aa) with IS5_IS427 e-value= 7.9e-16 fragment hit coverage= 27.03%, between model(296 aa) positions 129; 208 length is 78aa with 2 gaps, 0 stops, absolute frame= Plus1 at 1334629..1334862 in the same strand
CDS YP_001515704.1 IS4 family transposase intersects 29.12%(76aa) with IS5_IS5 e-value= 7.9e-09 fragment hit coverage= 19.19%, between model(422 aa) positions 79; 159 length is 76aa with 5 gaps, 0 stops, absolute frame= Plus1 at 1334704..1334931 in the same strand
misc_feature no id DDE superfamily endonuclease; Region: DDE_4; cl15789 intersects 62.67%(47aa) with IS5_IS5 e-value= 7.9e-09 fragment hit coverage= 19.19%, between model(422 aa) positions 79; 159 length is 76aa with 5 gaps, 0 stops, absolute frame= Plus1 at 1334704..1334931 in the same strand
misc_feature no id Transposase DDE domain; Region: DDE_Tnp_1; pfam01609 intersects 8.92%(14aa) with IS5_IS5 e-value= 7.9e-09 fragment hit coverage= 19.19%, between model(422 aa) positions 79; 159 length is 76aa with 5 gaps, 0 stops, absolute frame= Plus1 at 1334704..1334931 in the same strand

CDS YP_001515704.1 IS4 family transposase intersects 56.70%(148aa) with IS5_IS5 e-value= 1.2e-15 fragment hit coverage= 39.34%, between model(422 aa) positions 257; 422 length is 151aa with 15 gaps, 1 stops, absolute frame= Plus1 at 1334932..1335384 in the same strand
misc_feature no id Transposase DDE domain; Region: DDE_Tnp_1; pfam01609 intersects 91.08%(143aa) with IS5_IS5 e-value= 1.2e-15 fragment hit coverage= 39.34%, between model(422 aa) positions 257; 422 length is 151aa with 15 gaps, 1 stops, absolute frame= Plus1 at 1334932..1335384 in the same strand
misc_feature no id DDE superfamily endonuclease; Region: DDE_4; cl15789 intersects 100%(53aa) with IS5_IS5 e-value= 1.2e-15 fragment hit coverage= 39.34%, between model(422 aa) positions 257; 422 length is 151aa with 15 gaps, 1 stops, absolute frame= Plus1 at 1334932..1335384 in the same strand
Class A

67.
CDS YP_001515705.1 IS4 family transposase intersects 100%(267aa) with IS5_IS1031 e-value= 3e-112 complete sequence hit coverage= 100%, between model(295 aa) positions 1; 295 length is 284aa with 14 gaps, 3 stops, absolute frame= Minus1 at 1335394..1336244 in the same strand
misc_feature no id DDE superfamily endonuclease; Region: DDE_4; cl15789 intersects 100%(83aa) with IS5_IS1031 e-value= 3e-112 complete sequence hit coverage= 100%, between model(295 aa) positions 1; 295 length is 284aa with 14 gaps, 3 stops, absolute frame= Minus1 at 1335394..1336244 in the same strand
misc_feature no id Transposase DDE domain; Region: DDE_Tnp_1; pfam01609 intersects 100%(164aa) with IS5_IS1031 e-value= 3e-112 complete sequence hit coverage= 100%, between model(295 aa) positions 1; 295 length is 284aa with 14 gaps, 3 stops, absolute frame= Minus1 at 1335394..1336244 in the same strand
misc_feature no id DDE superfamily endonuclease; Region: DDE_4; cl15789 intersects 100%(75aa) with IS5_IS1031 e-value= 3e-112 complete sequence hit coverage= 100%, between model(295 aa) positions 1; 295 length is 284aa with 14 gaps, 3 stops, absolute frame= Minus1 at 1335394..1336244 in the same strand
CDS YP_001515705.1 IS4 family transposase intersects 98.38%(262aa) with IS5_IS1031 e-value= 2.4e-114 fragment hit coverage= 93.56%, between model(295 aa) positions 20; 295 length is 270aa with 9 gaps, 2 stops, absolute frame= Minus1 at 1335394..1336202 in the same strand
misc_feature no id DDE superfamily endonuclease; Region: DDE_4; cl15789 intersects 100%(83aa) with IS5_IS1031 e-value= 2.4e-114 fragment hit coverage= 93.56%, between model(295 aa) positions 20; 295 length is 270aa with 9 gaps, 2 stops, absolute frame= Minus1 at 1335394..1336202 in the same strand
misc_feature no id Transposase DDE domain; Region: DDE_Tnp_1; pfam01609 intersects 100%(164aa) with IS5_IS1031 e-value= 2.4e-114 fragment hit coverage= 93.56%, between model(295 aa) positions 20; 295 length is 270aa with 9 gaps, 2 stops, absolute frame= Minus1 at 1335394..1336202 in the same strand
misc_feature no id DDE superfamily endonuclease; Region: DDE_4; cl15789 intersects 100%(75aa) with IS5_IS1031 e-value= 2.4e-114 fragment hit coverage= 93.56%, between model(295 aa) positions 20; 295 length is 270aa with 9 gaps, 2 stops, absolute frame= Minus1 at 1335394..1336202 in the same strand
CDS YP_001515705.1 IS4 family transposase intersects 29.84%(79aa) with IS5_IS427 e-value= 1.2e-20 fragment hit coverage= 27.70%, between model(296 aa) positions 125; 206 length is 80aa with 2 gaps, 0 stops, absolute frame= Minus1 at 1335955..1336193 in the same strand
misc_feature no id DDE superfamily endonuclease; Region: DDE_4; cl15789 intersects 100%(75aa) with IS5_IS427 e-value= 1.2e-20 fragment hit coverage= 27.70%, between model(296 aa) positions 125; 206 length is 80aa with 2 gaps, 0 stops, absolute frame= Minus1 at 1335955..1336193 in the same strand
Class A

68.
gene no id fragment; similar to N-terminus of transposase intersects 100%(129aa) with IS5_ISL2 e-value= 2.2e-49 complete sequence hit coverage= 100%, between model(260 aa) positions 1; 260 length is 255aa with 23 gaps, 3 stops, absolute frame= Plus1 at 1474789..1475553 in the same strand
CDS YP_001515829.1 hypothetical protein intersects 88.89%(40aa) with IS5_ISL2 e-value= 2.2e-49 complete sequence hit coverage= 100%, between model(260 aa) positions 1; 260 length is 255aa with 23 gaps, 3 stops, absolute frame= Plus1 at 1474789..1475553 in the same strand
gene no id fragment; similar to N-terminus of transposase intersects 97.93%(126aa) with IS5_ISL2 e-value= 5e-10 complete sequence hit coverage= 100%, between model(260 aa) positions 1; 260 length is 233aa with 31 gaps, 7 stops, absolute frame= Plus3 at 1474827..1475525 in the same strand
CDS YP_001515829.1 hypothetical protein intersects 68.15%(30aa) with IS5_ISL2 e-value= 5e-10 complete sequence hit coverage= 100%, between model(260 aa) positions 1; 260 length is 233aa with 31 gaps, 7 stops, absolute frame= Plus3 at 1474827..1475525 in the same strand

gene no id fragment; similar to N-terminus of transposase intersects 79.84%(103aa) with IS5_ISL2 e-value= 1.5e-44 fragment hit coverage= 39.62%, between model(260 aa) positions 27; 129 length is 103aa with 0 gaps, 0 stops, absolute frame= Plus1 at 1474852..1475160 in the same strand
gene no id fragment; similar to N-terminus of transposase intersects 13.44%(17aa) with IS5_ISL2 e-value= 4.6e-35 fragment hit coverage= 37.31%, between model(260 aa) positions 128; 224 length is 97aa with 0 gaps, 0 stops, absolute frame= Plus3 at 1475154..1475444 in the same strand
CDS YP_001515829.1 hypothetical protein intersects 8.15%(3aa) with IS5_ISL2 e-value= 4.6e-35 fragment hit coverage= 37.31%, between model(260 aa) positions 128; 224 length is 97aa with 0 gaps, 0 stops, absolute frame= Plus3 at 1475154..1475444 in the same strand
CDS YP_001515829.1 hypothetical protein intersects 80%(36aa) with IS5_ISL2 e-value= 1.1e-16 fragment hit coverage= 13.85%, between model(260 aa) positions 225; 260 length is 36aa with 0 gaps, 0 stops, absolute frame= Plus1 at 1475446..1475553 in the same strand
Class A

69.
CDS YP_001515850.1 hypothetical protein intersects 67.18%(88aa) with IS1_ORF1 e-value= 1.7e-15 fragment hit coverage= 36.40%, between model(239 aa) positions 1; 87 length is 88aa with 2 gaps, 0 stops, absolute frame= Plus1 at 1499707..1499970 in the same strand
misc_feature no id Transposase and inactivated derivatives [DNA replication, recombination, and repair]; Region: COG3677 intersects 83.81%(88aa) with IS1_ORF1 e-value= 1.7e-15 fragment hit coverage= 36.40%, between model(239 aa) positions 1; 87 length is 88aa with 2 gaps, 0 stops, absolute frame= Plus1 at 1499707..1499970 in the same strand
Class A

70.
CDS YP_001515851.1 hypothetical protein intersects 27.54%(46aa) with IS1_ORF2 e-value= 1.8e-11 fragment hit coverage= 20.68%, between model(237 aa) positions 164; 212 length is 46aa with 3 gaps, 0 stops, absolute frame= Plus3 at 1500237..1500374 in the same strand
misc_feature no id IS1 transposase; Region: DDE_Tnp_IS1; cl00721 intersects 49.46%(46aa) with IS1_ORF2 e-value= 1.8e-11 fragment hit coverage= 20.68%, between model(237 aa) positions 164; 212 length is 46aa with 3 gaps, 0 stops, absolute frame= Plus3 at 1500237..1500374 in the same strand
Class A

71.
CDS YP_001515856.1 hypothetical protein intersects 81.36%(48aa) with IS1380 e-value= 2.1e-64 complete sequence hit coverage= 100%, between model(487 aa) positions 1; 487 length is 442aa with 47 gaps, 1 stops, absolute frame= Minus3 at 1503287..1504611 in the same strand
CDS YP_001515857.1 IS4 family transposase intersects 99.14%(382aa) with IS1380 e-value= 2.1e-64 complete sequence hit coverage= 100%, between model(487 aa) positions 1; 487 length is 442aa with 47 gaps, 1 stops, absolute frame= Minus3 at 1503287..1504611 in the same strand
misc_feature no id Transposase DDE domain group 1; Region: DDE_Tnp_1_4; pfam13701 intersects 100%(366aa) with IS1380 e-value= 2.1e-64 complete sequence hit coverage= 100%, between model(487 aa) positions 1; 487 length is 442aa with 47 gaps, 1 stops, absolute frame= Minus3 at 1503287..1504611 in the same strand
CDS YP_001515856.1 hypothetical protein intersects 81.36%(48aa) with IS1380 e-value= 1.5e-67 fragment hit coverage= 94.46%, between model(487 aa) positions 28; 487 length is 419aa with 43 gaps, 1 stops, absolute frame= Minus3 at 1503287..1504542 in the same strand
CDS YP_001515857.1 IS4 family transposase intersects 93.18%(359aa) with IS1380 e-value= 1.5e-67 fragment hit coverage= 94.46%, between model(487 aa) positions 28; 487 length is 419aa with 43 gaps, 1 stops, absolute frame= Minus3 at 1503287..1504542 in the same strand
misc_feature no id Transposase DDE domain group 1; Region: DDE_Tnp_1_4; pfam13701 intersects 98%(358aa) with IS1380 e-value= 1.5e-67 fragment hit coverage= 94.46%, between model(487 aa) positions 28; 487 length is 419aa with 43 gaps, 1 stops, absolute frame= Minus3 at 1503287..1504542 in the same strand
Class A

72.

CDS YP_001515882.1 hypothetical protein intersects 53.85%(49aa) with IS1_ORF1 e-value= 2.6e-10 fragment hit coverage= 27.62%, between model(239 aa) positions 28; 93 length is 68aa with 1 gaps, 0 stops, absolute frame= Plus2 at 1525481..1525684 in the same strand
misc_feature no id Helix-turn-helix domains; Region: HTH; cl00088 intersects 100%(35aa) with IS1_ORF1 e-value= 2.6e-10 fragment hit coverage= 27.62%, between model(239 aa) positions 28; 93 length is 68aa with 1 gaps, 0 stops, absolute frame= Plus2 at 1525481..1525684 in the same strand
Class B

73.
CDS YP_001515886.1 transposase intersects 41.38%(72aa) with IS630 e-value= 9.9e-08 fragment hit coverage= 20.74%, between model(376 aa) positions 44; 121 length is 72aa with 7 gaps, 0 stops, absolute frame= Plus2 at 1529090..1529305 in the same strand
misc_feature no id Helix-turn-helix domains; Region: HTH; cl00088 intersects 55.56%(30aa) with IS630 e-value= 9.9e-08 fragment hit coverage= 20.74%, between model(376 aa) positions 44; 121 length is 72aa with 7 gaps, 0 stops, absolute frame= Plus2 at 1529090..1529305 in the same strand
misc_feature no id Winged helix-turn helix; Region: HTH_29; pfam13551 intersects 63.72%(72aa) with IS630 e-value= 9.9e-08 fragment hit coverage= 20.74%, between model(376 aa) positions 44; 121 length is 72aa with 7 gaps, 0 stops, absolute frame= Plus2 at 1529090..1529305 in the same strand
misc_feature no id Helix-turn-helix domains; Region: HTH; cl00088 intersects 75%(60aa) with IS630 e-value= 9.9e-08 fragment hit coverage= 20.74%, between model(376 aa) positions 44; 121 length is 72aa with 7 gaps, 0 stops, absolute frame= Plus2 at 1529090..1529305 in the same strand
Class A

74.
CDS YP_001515943.1 transposase intersects 65.48%(129aa) with IS630 e-value= 6.9e-14 fragment hit coverage= 38.83%, between model(376 aa) positions 188; 333 length is 129aa with 20 gaps, 0 stops, absolute frame= Plus2 at 1580969..1581355 in the same strand
misc_feature no id Integrase core domain; Region: rve; cl01316 intersects 90.21%(129aa) with IS630 e-value= 6.9e-14 fragment hit coverage= 38.83%, between model(376 aa) positions 188; 333 length is 129aa with 20 gaps, 0 stops, absolute frame= Plus2 at 1580969..1581355 in the same strand
Class A

75.
CDS YP_001515950.1 transposase intersects 41.38%(72aa) with IS630 e-value= 9.9e-08 fragment hit coverage= 20.74%, between model(376 aa) positions 44; 121 length is 72aa with 7 gaps, 0 stops, absolute frame= Plus1 at 1585042..1585257 in the same strand
misc_feature no id Helix-turn-helix domains; Region: HTH; cl00088 intersects 55.56%(30aa) with IS630 e-value= 9.9e-08 fragment hit coverage= 20.74%, between model(376 aa) positions 44; 121 length is 72aa with 7 gaps, 0 stops, absolute frame= Plus1 at 1585042..1585257 in the same strand
misc_feature no id Winged helix-turn helix; Region: HTH_29; pfam13551 intersects 63.72%(72aa) with IS630 e-value= 9.9e-08 fragment hit coverage= 20.74%, between model(376 aa) positions 44; 121 length is 72aa with 7 gaps, 0 stops, absolute frame= Plus1 at 1585042..1585257 in the same strand
misc_feature no id Helix-turn-helix domains; Region: HTH; cl00088 intersects 75%(60aa) with IS630 e-value= 9.9e-08 fragment hit coverage= 20.74%, between model(376 aa) positions 44; 121 length is 72aa with 7 gaps, 0 stops, absolute frame= Plus1 at 1585042..1585257 in the same strand
Class A

76.
CDS YP_001515952.1 hypothetical protein intersects 67.18%(88aa) with IS1_ORF1 e-value= 1.7e-15 fragment hit coverage= 36.40%, between model(239 aa) positions 1; 87 length is 88aa with 2 gaps, 0 stops, absolute frame= Plus3 at 1586793..1587056 in the same strand
misc_feature no id Transposase and inactivated derivatives [DNA replication, recombination, and repair]; Region: COG3677 intersects 83.81%(88aa) with IS1_ORF1 e-value= 1.7e-15 fragment hit coverage= 36.40%, between model(239 aa) positions 1; 87 length is 88aa with 2 gaps, 0 stops, absolute frame= Plus3 at 1586793..1587056 in the same strand
Class A

77.
CDS YP_001515953.1 hypothetical protein intersects 27.54%(46aa) with IS1_ORF2 e-value= 1.8e-11 fragment hit coverage= 20.68%, between model(237 aa) positions 164; 212 length is 46aa with 3 gaps, 0 stops, absolute frame= Plus2 at 1587323..1587460 in the same strand
misc_feature no id IS1 transposase; Region: DDE_Tnp_IS1; cl00721 intersects 49.46%(46aa) with IS1_ORF2 e-value= 1.8e-11 fragment hit coverage= 20.68%, between model(237 aa) positions 164; 212 length is 46aa with 3 gaps, 0 stops, absolute frame= Plus2 at 1587323..1587460 in the same strand
Class A

78.
gene no id fragment; similar to N-terminus of transposase intersects 41.38%(72aa) with IS630 e-value= 9.9e-08 fragment hit coverage= 20.74%, between model(376 aa) positions 44; 121 length is 72aa with 7 gaps, 0 stops, absolute frame= Plus1 at 1602370..1602585 in the same strand
Class A

79.
CDS YP_001515991.1 IS4 family transposase intersects 100%(267aa) with IS5_IS1031 e-value= 2.8e-110 complete sequence hit coverage= 100%, between model(295 aa) positions 1; 295 length is 281aa with 17 gaps, 1 stops, absolute frame= Plus2 at 1631051..1631893 in the same strand
misc_feature no id DDE superfamily endonuclease; Region: DDE_4; cl15789 intersects 100%(75aa) with IS5_IS1031 e-value= 2.8e-110 complete sequence hit coverage= 100%, between model(295 aa) positions 1; 295 length is 281aa with 17 gaps, 1 stops, absolute frame= Plus2 at 1631051..1631893 in the same strand
misc_feature no id Transposase DDE domain; Region: DDE_Tnp_1; pfam01609 intersects 100%(164aa) with IS5_IS1031 e-value= 2.8e-110 complete sequence hit coverage= 100%, between model(295 aa) positions 1; 295 length is 281aa with 17 gaps, 1 stops, absolute frame= Plus2 at 1631051..1631893 in the same strand
misc_feature no id DDE superfamily endonuclease; Region: DDE_4; cl15789 intersects 100%(83aa) with IS5_IS1031 e-value= 2.8e-110 complete sequence hit coverage= 100%, between model(295 aa) positions 1; 295 length is 281aa with 17 gaps, 1 stops, absolute frame= Plus2 at 1631051..1631893 in the same strand
CDS YP_001515991.1 IS4 family transposase intersects 98.50%(263aa) with IS5_IS1031 e-value= 1.4e-112 fragment hit coverage= 93.56%, between model(295 aa) positions 20; 295 length is 270aa with 9 gaps, 1 stops, absolute frame= Plus2 at 1631084..1631893 in the same strand
misc_feature no id DDE superfamily endonuclease; Region: DDE_4; cl15789 intersects 100%(75aa) with IS5_IS1031 e-value= 1.4e-112 fragment hit coverage= 93.56%, between model(295 aa) positions 20; 295 length is 270aa with 9 gaps, 1 stops, absolute frame= Plus2 at 1631084..1631893 in the same strand
misc_feature no id Transposase DDE domain; Region: DDE_Tnp_1; pfam01609 intersects 100%(164aa) with IS5_IS1031 e-value= 1.4e-112 fragment hit coverage= 93.56%, between model(295 aa) positions 20; 295 length is 270aa with 9 gaps, 1 stops, absolute frame= Plus2 at 1631084..1631893 in the same strand
misc_feature no id DDE superfamily endonuclease; Region: DDE_4; cl15789 intersects 100%(83aa) with IS5_IS1031 e-value= 1.4e-112 fragment hit coverage= 93.56%, between model(295 aa) positions 20; 295 length is 270aa with 9 gaps, 1 stops, absolute frame= Plus2 at 1631084..1631893 in the same strand
CDS YP_001515991.1 IS4 family transposase intersects 29.96%(80aa) with IS5_IS427 e-value= 1.2e-18 fragment hit coverage= 27.70%, between model(296 aa) positions 125; 206 length is 80aa with 2 gaps, 0 stops, absolute frame= Plus2 at 1631093..1631332 in the same strand
misc_feature no id DDE superfamily endonuclease; Region: DDE_4; cl15789 intersects 100%(75aa) with IS5_IS427 e-value= 1.2e-18 fragment hit coverage= 27.70%, between model(296 aa) positions 125; 206 length is 80aa with 2 gaps, 0 stops, absolute frame= Plus2 at 1631093..1631332 in the same strand
Class A

80.
CDS YP_001516231.1 hypothetical protein intersects 1.75%(1aa) with IS630 e-value= 4e-10 fragment hit coverage= 19.15%, between model(376 aa) positions 305; 376 length is 73aa with 0 gaps, 1 stops, absolute frame= Minus2 at 1894341..1894558 in the same strand
gene no id fragment; similar to C-terminus of transposase intersects 30.46%(70aa) with IS630 e-value= 4e-10 fragment hit coverage= 19.15%, between model(376 aa) positions 305; 376 length is 73aa with 0 gaps, 1 stops, absolute frame= Minus2 at 1894341..1894558 in the same strand

gene no id fragment; similar to C-terminus of transposase intersects 15.80%(36aa) with IS3_IS150_ORF2 e-value= 2e-08 fragment hit coverage= 10.03%, between model(389 aa) positions 329; 367 length is 37aa with 2 gaps, 0 stops, absolute frame= Minus2 at 1894401..1894510 in the same strand
Class A

81.
gene no id fragment; similar to N-terminus of transposase intersects 36.69%(65aa) with IS630 e-value= 4e-07 fragment hit coverage= 17.55%, between model(376 aa) positions 1; 66 length is 66aa with 0 gaps, 0 stops, absolute frame= Minus1 at 1895383..1895579 in the same strand
Class A

82.
CDS YP_001516259.1 transposase intersects 85.96%(375aa) with IS4 e-value= 9.9e-07 complete sequence hit coverage= 100%, between model(486 aa) positions 1; 486 length is 376aa with 117 gaps, 0 stops, absolute frame= Minus1 at 1923067..1924193 in the same strand
misc_feature no id DDE superfamily endonuclease; Region: DDE_5; cl02413 intersects 93.48%(348aa) with IS4 e-value= 9.9e-07 complete sequence hit coverage= 100%, between model(486 aa) positions 1; 486 length is 376aa with 117 gaps, 0 stops, absolute frame= Minus1 at 1923067..1924193 in the same strand
CDS YP_001516259.1 transposase intersects 53.01%(231aa) with IS4 e-value= 4.7e-14 fragment hit coverage= 51.23%, between model(486 aa) positions 1; 249 length is 232aa with 27 gaps, 0 stops, absolute frame= Minus1 at 1923499..1924193 in the same strand
misc_feature no id DDE superfamily endonuclease; Region: DDE_5; cl02413 intersects 62.11%(231aa) with IS4 e-value= 4.7e-14 fragment hit coverage= 51.23%, between model(486 aa) positions 1; 249 length is 232aa with 27 gaps, 0 stops, absolute frame= Minus1 at 1923499..1924193 in the same strand
Class A

83.
CDS YP_001516423.1 hypothetical protein intersects 35.29%(48aa) with IS630 e-value= 1.7e-14 fragment hit coverage= 13.30%, between model(376 aa) positions 281; 330 length is 48aa with 2 gaps, 0 stops, absolute frame= Plus2 at 2086388..2086531 in the same strand
misc_feature no id Integrase core domain; Region: rve; cl01316 intersects 40%(48aa) with IS630 e-value= 1.7e-14 fragment hit coverage= 13.30%, between model(376 aa) positions 281; 330 length is 48aa with 2 gaps, 0 stops, absolute frame= Plus2 at 2086388..2086531 in the same strand
Class A

84.
CDS YP_001516424.1 IS4 family transposase intersects 85.36%(147aa) with IS5_IS1031 e-value= 2.2e-60 fragment hit coverage= 51.86%, between model(295 aa) positions 143; 295 length is 155aa with 1 gaps, 2 stops, absolute frame= Minus2 at 2086668..2087131 in the same strand
misc_feature no id DDE superfamily endonuclease; Region: DDE_4; cl15789 intersects 100%(83aa) with IS5_IS1031 e-value= 2.2e-60 fragment hit coverage= 51.86%, between model(295 aa) positions 143; 295 length is 155aa with 1 gaps, 2 stops, absolute frame= Minus2 at 2086668..2087131 in the same strand
CDS YP_001516424.1 IS4 family transposase intersects 100%(173aa) with IS5_IS1031 e-value= 2.9e-19 complete sequence hit coverage= 100%, between model(295 aa) positions 1; 295 length is 190aa with 108 gaps, 2 stops, absolute frame= Minus2 at 2086668..2087236 in the same strand
misc_feature no id DDE superfamily endonuclease; Region: DDE_4; cl15789 intersects 100%(83aa) with IS5_IS1031 e-value= 2.9e-19 complete sequence hit coverage= 100%, between model(295 aa) positions 1; 295 length is 190aa with 108 gaps, 2 stops, absolute frame= Minus2 at 2086668..2087236 in the same strand
CDS YP_001516425.1 transposase intersects 6.46%(10aa) with IS5_IS1031 e-value= 2.9e-19 complete sequence hit coverage= 100%, between model(295 aa) positions 1; 295 length is 190aa with 108 gaps, 2 stops, absolute frame= Minus2 at 2086668..2087236 in the opposite strand
Class A

85.
CDS YP_001516426.1 transposase intersects 43.60%(80aa) with IS5_IS1031 e-value= 3.4e-16 complete sequence hit coverage= 100%, between model(295 aa) positions 1; 295 length is 211aa with 86 gaps, 7 stops, absolute frame= Minus2 at 2088024..2088655 in the opposite strand

misc_feature no id Integrase core domain; Region: rve; cl01316 intersects 33.33%(45aa) with IS5_IS1031 e-value= 3.4e-16 complete sequence hit coverage= 100%, between model(295 aa) positions 1; 295 length is 211aa with 86 gaps, 7 stops, absolute frame= Minus2 at 2088024..2088655 in the opposite strand
CDS YP_001516427.1 transposase intersects 100%(124aa) with IS5_IS1031 e-value= 3.4e-16 complete sequence hit coverage= 100%, between model(295 aa) positions 1; 295 length is 211aa with 86 gaps, 7 stops, absolute frame= Minus2 at 2088024..2088655 in the same strand
misc_feature no id DDE superfamily endonuclease; Region: DDE_4; cl15789 intersects 100%(75aa) with IS5_IS1031 e-value= 3.4e-16 complete sequence hit coverage= 100%, between model(295 aa) positions 1; 295 length is 211aa with 86 gaps, 7 stops, absolute frame= Minus2 at 2088024..2088655 in the same strand
CDS YP_001516427.1 transposase intersects 85.22%(105aa) with IS5_IS1031 e-value= 7.2e-51 fragment hit coverage= 38.31%, between model(295 aa) positions 20; 132 length is 106aa with 7 gaps, 0 stops, absolute frame= Minus2 at 2088297..2088613 in the same strand
misc_feature no id DDE superfamily endonuclease; Region: DDE_4; cl15789 intersects 100%(75aa) with IS5_IS1031 e-value= 7.2e-51 fragment hit coverage= 38.31%, between model(295 aa) positions 20; 132 length is 106aa with 7 gaps, 0 stops, absolute frame= Minus2 at 2088297..2088613 in the same strand
CDS YP_001516427.1 transposase intersects 64.25%(79aa) with IS5_IS427 e-value= 9.2e-19 fragment hit coverage= 27.70%, between model(296 aa) positions 125; 206 length is 80aa with 2 gaps, 0 stops, absolute frame= Minus2 at 2088366..2088604 in the same strand
misc_feature no id DDE superfamily endonuclease; Region: DDE_4; cl15789 intersects 100%(75aa) with IS5_IS427 e-value= 9.2e-19 fragment hit coverage= 27.70%, between model(296 aa) positions 125; 206 length is 80aa with 2 gaps, 0 stops, absolute frame= Minus2 at 2088366..2088604 in the same strand
Class A

86.
CDS YP_001516538.1 IS5 family transposase intersects 15.17%(81aa) with IS5_IS5 e-value= 2.6e-06 fragment hit coverage= 19.91%, between model(422 aa) positions 23; 106 length is 81aa with 3 gaps, 0 stops, absolute frame= Plus2 at 2216453..2216695 in the same strand
misc_feature no id Transposase domain (DUF772); Region: DUF772; pfam05598 intersects 74.03%(57aa) with IS5_IS5 e-value= 2.6e-06 fragment hit coverage= 19.91%, between model(422 aa) positions 23; 106 length is 81aa with 3 gaps, 0 stops, absolute frame= Plus2 at 2216453..2216695 in the same strand
Class A

87.
CDS YP_001516600.1 hypothetical protein intersects 100%(65aa) with IS200_IS605 e-value= 3.6e-09 complete sequence hit coverage= 100%, between model(153 aa) positions 1; 153 length is 154aa with 3 gaps, 3 stops, absolute frame= Plus2 at 2274167..2274628 in the same strand
misc_feature no id Transposase IS200 like; Region: Y1_Tnp; cl00848 intersects 100%(50aa) with IS200_IS605 e-value= 3.6e-09 complete sequence hit coverage= 100%, between model(153 aa) positions 1; 153 length is 154aa with 3 gaps, 3 stops, absolute frame= Plus2 at 2274167..2274628 in the same strand
CDS YP_001516600.1 hypothetical protein intersects 100%(65aa) with IS200 e-value= 1.5e-09 complete sequence hit coverage= 100%, between model(154 aa) positions 1; 154 length is 155aa with 5 gaps, 3 stops, absolute frame= Plus2 at 2274167..2274631 in the same strand
misc_feature no id Transposase IS200 like; Region: Y1_Tnp; cl00848 intersects 100%(50aa) with IS200 e-value= 1.5e-09 complete sequence hit coverage= 100%, between model(154 aa) positions 1; 154 length is 155aa with 5 gaps, 3 stops, absolute frame= Plus2 at 2274167..2274631 in the same strand
CDS YP_001516600.1 hypothetical protein intersects 76.92%(50aa) with IS200_IS605 e-value= 7.4e-15 fragment hit coverage= 52.29%, between model(153 aa) positions 51; 130 length is 80aa with 0 gaps, 0 stops, absolute frame= Plus2 at 2274320..2274559 in the same strand
misc_feature no id Transposase IS200 like; Region: Y1_Tnp; cl00848 intersects 100%(50aa) with IS200_IS605 e-value= 7.4e-15 fragment hit coverage= 52.29%, between model(153 aa) positions 51; 130 length is 80aa with 0 gaps, 0 stops, absolute frame= Plus2 at 2274320..2274559 in the same strand
Class A

88.
CDS YP_001516604.1 hypothetical protein intersects 51.25%(41aa) with IS1_ORF1 e-value= 8.4e-08 fragment hit coverage= 33.89%, between model(239 aa) positions 5; 85 length is 86aa with 2 gaps, 0 stops, absolute frame= Minus3 at 2279714..2279970 in the same strand

Class B

89.
CDS YP_001516610.1 IS1 transposase intersects 96.95%(127aa) with IS1_ORF2 e-value= 4.6e-13 complete sequence hit coverage= 100%, between model(237 aa) positions 1; 237 length is 213aa with 27 gaps, 3 stops, absolute frame= Minus2 at 2284359..2284996 in the same strand
misc_feature no id IS1 transposase; Region: DDE_Tnp_IS1; cl00721 intersects 100%(123aa) with IS1_ORF2 e-value= 4.6e-13 complete sequence hit coverage= 100%, between model(237 aa) positions 1; 237 length is 213aa with 27 gaps, 3 stops, absolute frame= Minus2 at 2284359..2284996 in the same strand
gene no id fragment; similar to N-terminus of transposase intersects 81.88%(84aa) with IS1_ORF2 e-value= 4.6e-13 complete sequence hit coverage= 100%, between model(237 aa) positions 1; 237 length is 213aa with 27 gaps, 3 stops, absolute frame= Minus2 at 2284359..2284996 in the same strand
CDS YP_001516610.1 IS1 transposase intersects 96.95%(127aa) with IS1_ORF2 e-value= 6.2e-32 fragment hit coverage= 54.43%, between model(237 aa) positions 109; 237 length is 129aa with 1 gaps, 0 stops, absolute frame= Minus2 at 2284359..2284744 in the same strand
misc_feature no id IS1 transposase; Region: DDE_Tnp_IS1; cl00721 intersects 100%(123aa) with IS1_ORF2 e-value= 6.2e-32 fragment hit coverage= 54.43%, between model(237 aa) positions 109; 237 length is 129aa with 1 gaps, 0 stops, absolute frame= Minus2 at 2284359..2284744 in the same strand
gene no id fragment; similar to N-terminus of transposase intersects 0.32%(0aa) with IS1_ORF2 e-value= 6.2e-32 fragment hit coverage= 54.43%, between model(237 aa) positions 109; 237 length is 129aa with 1 gaps, 0 stops, absolute frame= Minus2 at 2284359..2284744 in the same strand
CDS YP_001516610.1 IS1 transposase intersects 94.66%(124aa) with IS1_ORF1 e-value= 4.6e-14 fragment hit coverage= 53.97%, between model(239 aa) positions 111; 239 length is 126aa with 7 gaps, 0 stops, absolute frame= Minus2 at 2284368..2284744 in the same strand
misc_feature no id IS1 transposase; Region: DDE_Tnp_IS1; cl00721 intersects 100%(123aa) with IS1_ORF1 e-value= 4.6e-14 fragment hit coverage= 53.97%, between model(239 aa) positions 111; 239 length is 126aa with 7 gaps, 0 stops, absolute frame= Minus2 at 2284368..2284744 in the same strand
gene no id fragment; similar to N-terminus of transposase intersects 0.32%(0aa) with IS1_ORF1 e-value= 4.6e-14 fragment hit coverage= 53.97%, between model(239 aa) positions 111; 239 length is 126aa with 7 gaps, 0 stops, absolute frame= Minus2 at 2284368..2284744 in the same strand
gene no id fragment; similar to N-terminus of transposase intersects 67.96%(70aa) with IS1_ORF1 e-value= 3.6e-14 fragment hit coverage= 32.64%, between model(239 aa) positions 1; 78 length is 75aa with 3 gaps, 0 stops, absolute frame= Minus1 at 2284843..2285066 in the same strand
CDS YP_001516611.1 hypothetical protein intersects 9.80%(5aa) with IS1_ORF1 e-value= 3.6e-14 fragment hit coverage= 32.64%, between model(239 aa) positions 1; 78 length is 75aa with 3 gaps, 0 stops, absolute frame= Minus1 at 2284843..2285066 in the opposite strand
Class A

90.
CDS YP_001516695.1 hypothetical protein intersects 45.61%(26aa) with IS5_ISL2 e-value= 1.6e-11 complete sequence hit coverage= 100%, between model(260 aa) positions 1; 260 length is 273aa with 11 gaps, 0 stops, absolute frame= Plus1 at 2373475..2374293 in the same strand
misc_feature no id DDE superfamily endonuclease; Region: DDE_4_2; pfam13613 intersects 100%(69aa) with IS5_ISL2 e-value= 1.6e-11 complete sequence hit coverage= 100%, between model(260 aa) positions 1; 260 length is 273aa with 11 gaps, 0 stops, absolute frame= Plus1 at 2373475..2374293 in the same strand
CDS YP_001516696.1 hypothetical protein intersects 94.62%(211aa) with IS5_ISL2 e-value= 1.6e-11 complete sequence hit coverage= 100%, between model(260 aa) positions 1; 260 length is 273aa with 11 gaps, 0 stops, absolute frame= Plus1 at 2373475..2374293 in the same strand
misc_feature no id DDE superfamily endonuclease; Region: DDE_4; cl15789 intersects 100%(151aa) with IS5_ISL2 e-value= 1.6e-11 complete sequence hit coverage= 100%, between model(260 aa) positions 1; 260 length is 273aa with 11 gaps, 0 stops, absolute frame= Plus1 at 2373475..2374293 in the same strand
CDS YP_001516696.1 hypothetical protein intersects 53.81%(120aa) with IS5_ISL2 e-value= 8.7e-11 fragment hit coverage= 46.92%, between model(260 aa) positions 120; 241 length is 120aa with 4 gaps, 0 stops, absolute frame= Plus1 at 2373877..2374236 in the same strand
misc_feature no id DDE superfamily endonuclease; Region: DDE_4; cl15789 intersects 79.47%(120aa) with IS5_ISL2 e-value= 8.7e-11 fragment hit coverage= 46.92%, between model(260 aa) positions 120; 241 length is 120aa with 4 gaps, 0 stops, absolute frame= Plus1 at 2373877..2374236 in the same strand

Class B

91.
CDS YP_001516702.1 hypothetical protein intersects 80%(128aa) with IS630 e-value= 6.5e-11 fragment hit coverage= 39.10%, between model(376 aa) positions 187; 333 length is 130aa with 21 gaps, 0 stops, absolute frame= Minus3 at 2382314..2382702 in the same strand
misc_feature no id Integrase core domain; Region: rve; cl01316 intersects 100%(128aa) with IS630 e-value= 6.5e-11 fragment hit coverage= 39.10%, between model(376 aa) positions 187; 333 length is 130aa with 21 gaps, 0 stops, absolute frame= Minus3 at 2382314..2382702 in the same strand
Class A

92.
CDS YP_001516703.1 hypothetical protein intersects 86.41%(135aa) with IS630 e-value= 8.7e-15 fragment hit coverage= 39.63%, between model(376 aa) positions 1; 149 length is 136aa with 13 gaps, 0 stops, absolute frame= Minus1 at 2382808..2383214 in the same strand
misc_feature no id Winged helix-turn helix; Region: HTH_29; pfam13551 intersects 100%(102aa) with IS630 e-value= 8.7e-15 fragment hit coverage= 39.63%, between model(376 aa) positions 1; 149 length is 136aa with 13 gaps, 0 stops, absolute frame= Minus1 at 2382808..2383214 in the same strand
misc_feature no id Helix-turn-helix domains; Region: HTH; cl00088 intersects 100%(72aa) with IS630 e-value= 8.7e-15 fragment hit coverage= 39.63%, between model(376 aa) positions 1; 149 length is 136aa with 13 gaps, 0 stops, absolute frame= Minus1 at 2382808..2383214 in the same strand
misc_feature no id Helix-turn-helix domains; Region: HTH; cl00088 intersects 100%(50aa) with IS630 e-value= 8.7e-15 fragment hit coverage= 39.63%, between model(376 aa) positions 1; 149 length is 136aa with 13 gaps, 0 stops, absolute frame= Minus1 at 2382808..2383214 in the same strand
CDS YP_001516703.1 hypothetical protein intersects 71.76%(112aa) with IS481 e-value= 3.5e-09 fragment hit coverage= 33.62%, between model(351 aa) positions 1; 118 length is 113aa with 6 gaps, 0 stops, absolute frame= Minus1 at 2382817..2383154 in the same strand
misc_feature no id Winged helix-turn helix; Region: HTH_29; pfam13551 intersects 100%(102aa) with IS481 e-value= 3.5e-09 fragment hit coverage= 33.62%, between model(351 aa) positions 1; 118 length is 113aa with 6 gaps, 0 stops, absolute frame= Minus1 at 2382817..2383154 in the same strand
misc_feature no id Helix-turn-helix domains; Region: HTH; cl00088 intersects 100%(72aa) with IS481 e-value= 3.5e-09 fragment hit coverage= 33.62%, between model(351 aa) positions 1; 118 length is 113aa with 6 gaps, 0 stops, absolute frame= Minus1 at 2382817..2383154 in the same strand
misc_feature no id Helix-turn-helix domains; Region: HTH; cl00088 intersects 100%(50aa) with IS481 e-value= 3.5e-09 fragment hit coverage= 33.62%, between model(351 aa) positions 1; 118 length is 113aa with 6 gaps, 0 stops, absolute frame= Minus1 at 2382817..2383154 in the same strand
Class B

93.
CDS YP_001516720.1 transposase intersects 85.96%(375aa) with IS4 e-value= 9.9e-07 complete sequence hit coverage= 100%, between model(486 aa) positions 1; 486 length is 376aa with 117 gaps, 0 stops, absolute frame= Minus3 at 2402957..2404083 in the same strand
misc_feature no id DDE superfamily endonuclease; Region: DDE_5; cl02413 intersects 93.48%(348aa) with IS4 e-value= 9.9e-07 complete sequence hit coverage= 100%, between model(486 aa) positions 1; 486 length is 376aa with 117 gaps, 0 stops, absolute frame= Minus3 at 2402957..2404083 in the same strand
CDS YP_001516720.1 transposase intersects 53.01%(231aa) with IS4 e-value= 4.7e-14 fragment hit coverage= 51.23%, between model(486 aa) positions 1; 249 length is 232aa with 27 gaps, 0 stops, absolute frame= Minus3 at 2403389..2404083 in the same strand
misc_feature no id DDE superfamily endonuclease; Region: DDE_5; cl02413 intersects 62.11%(231aa) with IS4 e-value= 4.7e-14 fragment hit coverage= 51.23%, between model(486 aa) positions 1; 249 length is 232aa with 27 gaps, 0 stops, absolute frame= Minus3 at 2403389..2404083 in the same strand
Class A

94.
CDS YP_001516721.1 hypothetical protein intersects 66.20%(141aa) with IS630 e-value= 6.2e-18 fragment hit coverage= 38.56%, between model(376 aa) positions 232; 376 length is 142aa with 4 gaps, 0 stops, absolute frame= Minus2 at 2404278..2404702 in the same strand

misc_feature no id Integrase core domain; Region: rve; cl01316 intersects 78.49%(110aa) with IS630 e-value= 6.2e-18 fragment hit coverage= 38.56%, between model(376 aa) positions 232; 376 length is 142aa with 4 gaps, 0 stops, absolute frame= Minus2 at 2404278..2404702 in the same strand
Class A

95.
CDS YP_001516722.1 hypothetical protein intersects 32.11%(52aa) with IS481 e-value= 1.1e-08 fragment hit coverage= 15.10%, between model(351 aa) positions 1; 53 length is 53aa with 0 gaps, 0 stops, absolute frame= Minus1 at 2405098..2405255 in the same strand
misc_feature no id Winged helix-turn helix; Region: HTH_29; pfam13551 intersects 45.36%(44aa) with IS481 e-value= 1.1e-08 fragment hit coverage= 15.10%, between model(351 aa) positions 1; 53 length is 53aa with 0 gaps, 0 stops, absolute frame= Minus1 at 2405098..2405255 in the same strand
misc_feature no id Helix-turn-helix domains; Region: HTH; cl00088 intersects 100%(50aa) with IS481 e-value= 1.1e-08 fragment hit coverage= 15.10%, between model(351 aa) positions 1; 53 length is 53aa with 0 gaps, 0 stops, absolute frame= Minus1 at 2405098..2405255 in the same strand
Class B

96.
CDS YP_001516727.1 transposase intersects 81.70%(83aa) with IS3_IS150_ORF2 e-value= 1.7e-22 complete sequence hit coverage= 100%, between model(389 aa) positions 1; 389 length is 354aa with 39 gaps, 5 stops, absolute frame= Plus2 at 2409029..2410090 in the same strand
misc_feature no id Helix-turn-helix domains; Region: HTH; cl00088 intersects 79.11%(59aa) with IS3_IS150_ORF2 e-value= 1.7e-22 complete sequence hit coverage= 100%, between model(389 aa) positions 1; 389 length is 354aa with 39 gaps, 5 stops, absolute frame= Plus2 at 2409029..2410090 in the same strand
CDS YP_001516728.1 integrase catalytic subunit intersects 95.45%(273aa) with IS3_IS150_ORF2 e-value= 1.7e-22 complete sequence hit coverage= 100%, between model(389 aa) positions 1; 389 length is 354aa with 39 gaps, 5 stops, absolute frame= Plus2 at 2409029..2410090 in the same strand
misc_feature no id putative transposase OrfB; Reviewed; Region: PHA02517 intersects 99.21%(252aa) with IS3_IS150_ORF2 e-value= 1.7e-22 complete sequence hit coverage= 100%, between model(389 aa) positions 1; 389 length is 354aa with 39 gaps, 5 stops, absolute frame= Plus2 at 2409029..2410090 in the same strand
misc_feature no id HTH-like domain; Region: HTH_21; pfam13276 intersects 100%(42aa) with IS3_IS150_ORF2 e-value= 1.7e-22 complete sequence hit coverage= 100%, between model(389 aa) positions 1; 389 length is 354aa with 39 gaps, 5 stops, absolute frame= Plus2 at 2409029..2410090 in the same strand
misc_feature no id Integrase core domain; Region: rve; cl01316 intersects 100%(110aa) with IS3_IS150_ORF2 e-value= 1.7e-22 complete sequence hit coverage= 100%, between model(389 aa) positions 1; 389 length is 354aa with 39 gaps, 5 stops, absolute frame= Plus2 at 2409029..2410090 in the same strand
misc_feature no id Integrase core domain; Region: rve_3; cl15866 intersects 100%(68aa) with IS3_IS150_ORF2 e-value= 1.7e-22 complete sequence hit coverage= 100%, between model(389 aa) positions 1; 389 length is 354aa with 39 gaps, 5 stops, absolute frame= Plus2 at 2409029..2410090 in the same strand
CDS YP_001516727.1 transposase intersects 24.84%(25aa) with IS3_IS150_ORF1 e-value= 4.3e-49 complete sequence hit coverage= 100%, between model(306 aa) positions 1; 306 length is 295aa with 14 gaps, 0 stops, absolute frame= Plus2 at 2409203..2410087 in the same strand
misc_feature no id Helix-turn-helix domains; Region: HTH; cl00088 intersects 1.78%(1aa) with IS3_IS150_ORF1 e-value= 4.3e-49 complete sequence hit coverage= 100%, between model(306 aa) positions 1; 306 length is 295aa with 14 gaps, 0 stops, absolute frame= Plus2 at 2409203..2410087 in the same strand
CDS YP_001516728.1 integrase catalytic subunit intersects 95.10%(272aa) with IS3_IS150_ORF1 e-value= 4.3e-49 complete sequence hit coverage= 100%, between model(306 aa) positions 1; 306 length is 295aa with 14 gaps, 0 stops, absolute frame= Plus2 at 2409203..2410087 in the same strand
misc_feature no id putative transposase OrfB; Reviewed; Region: PHA02517 intersects 98.82%(251aa) with IS3_IS150_ORF1 e-value= 4.3e-49 complete sequence hit coverage= 100%, between model(306 aa) positions 1; 306 length is 295aa with 14 gaps, 0 stops, absolute frame= Plus2 at 2409203..2410087 in the same strand
misc_feature no id HTH-like domain; Region: HTH_21; pfam13276 intersects 100%(42aa) with IS3_IS150_ORF1 e-value= 4.3e-49 complete sequence hit coverage= 100%, between model(306 aa) positions 1; 306 length is 295aa with 14 gaps, 0 stops, absolute frame= Plus2 at 2409203..2410087 in the same strand
misc_feature no id Integrase core domain; Region: rve; cl01316 intersects 100%(110aa) with IS3_IS150_ORF1 e-value= 4.3e-49 complete sequence hit coverage= 100%, between model(306 aa) positions 1; 306 length is 295aa with 14 gaps, 0 stops, absolute frame= Plus2 at 2409203..2410087 in the same strand

misc_feature no id Integrase core domain; Region: rve_3; cl15866 intersects 100%(68aa) with IS3_IS150_ORF1 e-value= 4.3e-49 complete sequence hit coverage= 100%, between model(306 aa) positions 1; 306 length is 295aa with 14 gaps, 0 stops, absolute frame= Plus2 at 2409203..2410087 in the same strand
CDS YP_001516727.1 transposase intersects 13.07%(13aa) with IS3_IS51_ORF2 e-value= 1.6e-26 complete sequence hit coverage= 100%, between model(317 aa) positions 1; 317 length is 295aa with 27 gaps, 0 stops, absolute frame= Plus2 at 2409239..2410123 in the same strand
CDS YP_001516728.1 integrase catalytic subunit intersects 99.30%(284aa) with IS3_IS51_ORF2 e-value= 1.6e-26 complete sequence hit coverage= 100%, between model(317 aa) positions 1; 317 length is 295aa with 27 gaps, 0 stops, absolute frame= Plus2 at 2409239..2410123 in the same strand
misc_feature no id putative transposase OrfB; Reviewed; Region: PHA02517 intersects 100%(254aa) with IS3_IS51_ORF2 e-value= 1.6e-26 complete sequence hit coverage= 100%, between model(317 aa) positions 1; 317 length is 295aa with 27 gaps, 0 stops, absolute frame= Plus2 at 2409239..2410123 in the same strand
misc_feature no id HTH-like domain; Region: HTH_21; pfam13276 intersects 100%(42aa) with IS3_IS51_ORF2 e-value= 1.6e-26 complete sequence hit coverage= 100%, between model(317 aa) positions 1; 317 length is 295aa with 27 gaps, 0 stops, absolute frame= Plus2 at 2409239..2410123 in the same strand
misc_feature no id Integrase core domain; Region: rve; cl01316 intersects 100%(110aa) with IS3_IS51_ORF2 e-value= 1.6e-26 complete sequence hit coverage= 100%, between model(317 aa) positions 1; 317 length is 295aa with 27 gaps, 0 stops, absolute frame= Plus2 at 2409239..2410123 in the same strand
misc_feature no id Integrase core domain; Region: rve_3; cl15866 intersects 100%(68aa) with IS3_IS51_ORF2 e-value= 1.6e-26 complete sequence hit coverage= 100%, between model(317 aa) positions 1; 317 length is 295aa with 27 gaps, 0 stops, absolute frame= Plus2 at 2409239..2410123 in the same strand
CDS YP_001516727.1 transposase intersects 11.11%(11aa) with IS3_IS407_ORF2 e-value= 1.2e-17 fragment hit coverage= 25.29%, between model(344 aa) positions 60; 146 length is 85aa with 2 gaps, 0 stops, absolute frame= Plus2 at 2409245..2409499 in the same strand
CDS YP_001516728.1 integrase catalytic subunit intersects 26.57%(76aa) with IS3_IS407_ORF2 e-value= 1.2e-17 fragment hit coverage= 25.29%, between model(344 aa) positions 60; 146 length is 85aa with 2 gaps, 0 stops, absolute frame= Plus2 at 2409245..2409499 in the same strand
misc_feature no id putative transposase OrfB; Reviewed; Region: PHA02517 intersects 21.65%(55aa) with IS3_IS407_ORF2 e-value= 1.2e-17 fragment hit coverage= 25.29%, between model(344 aa) positions 60; 146 length is 85aa with 2 gaps, 0 stops, absolute frame= Plus2 at 2409245..2409499 in the same strand
misc_feature no id HTH-like domain; Region: HTH_21; pfam13276 intersects 66.67%(28aa) with IS3_IS407_ORF2 e-value= 1.2e-17 fragment hit coverage= 25.29%, between model(344 aa) positions 60; 146 length is 85aa with 2 gaps, 0 stops, absolute frame= Plus2 at 2409245..2409499 in the same strand
CDS YP_001516727.1 transposase intersects 5.23%(5aa) with IS3_IS150_ORF2 e-value= 2.2e-47 fragment hit coverage= 76.09%, between model(389 aa) positions 94; 389 length is 276aa with 23 gaps, 0 stops, absolute frame= Plus2 at 2409263..2410090 in the same strand
CDS YP_001516728.1 integrase catalytic subunit intersects 95.45%(273aa) with IS3_IS150_ORF2 e-value= 2.2e-47 fragment hit coverage= 76.09%, between model(389 aa) positions 94; 389 length is 276aa with 23 gaps, 0 stops, absolute frame= Plus2 at 2409263..2410090 in the same strand
misc_feature no id putative transposase OrfB; Reviewed; Region: PHA02517 intersects 99.21%(252aa) with IS3_IS150_ORF2 e-value= 2.2e-47 fragment hit coverage= 76.09%, between model(389 aa) positions 94; 389 length is 276aa with 23 gaps, 0 stops, absolute frame= Plus2 at 2409263..2410090 in the same strand
misc_feature no id HTH-like domain; Region: HTH_21; pfam13276 intersects 100%(42aa) with IS3_IS150_ORF2 e-value= 2.2e-47 fragment hit coverage= 76.09%, between model(389 aa) positions 94; 389 length is 276aa with 23 gaps, 0 stops, absolute frame= Plus2 at 2409263..2410090 in the same strand
misc_feature no id Integrase core domain; Region: rve; cl01316 intersects 100%(110aa) with IS3_IS150_ORF2 e-value= 2.2e-47 fragment hit coverage= 76.09%, between model(389 aa) positions 94; 389 length is 276aa with 23 gaps, 0 stops, absolute frame= Plus2 at 2409263..2410090 in the same strand
misc_feature no id Integrase core domain; Region: rve_3; cl15866 intersects 100%(68aa) with IS3_IS150_ORF2 e-value= 2.2e-47 fragment hit coverage= 76.09%, between model(389 aa) positions 94; 389 length is 276aa with 23 gaps, 0 stops, absolute frame= Plus2 at 2409263..2410090 in the same strand
CDS YP_001516727.1 transposase intersects 4.25%(4aa) with IS3_IS51_ORF1 e-value= 6.3e-51 complete sequence hit coverage= 100%, between model(307 aa) positions 1; 307 length is 283aa with 25 gaps, 0 stops, absolute frame= Plus2 at 2409266..2410114 in the same strand
CDS YP_001516728.1 integrase catalytic subunit intersects 98.25%(281aa) with IS3_IS51_ORF1 e-value= 6.3e-51 complete sequence hit coverage= 100%, between model(307 aa) positions 1; 307 length is 283aa with 25 gaps, 0 stops, absolute frame= Plus2 at 2409266..2410114 in the same strand

misc_feature	no id	putative transposase OrfB; Reviewed; Region: PHA02517 intersects 100%(254aa) with IS3_IS51_ORF1 e-value= 6.3e-51 complete sequence hit coverage= 100%, between model(307 aa) positions 1; 307 length is 283aa with 25 gaps, 0 stops, absolute frame= Plus2 at 2409266..2410114 in the same strand
misc_feature	no id	HTH-like domain; Region: HTH_21; pfam13276 intersects 100%(42aa) with IS3_IS51_ORF1 e-value= 6.3e-51 complete sequence hit coverage= 100%, between model(307 aa) positions 1; 307 length is 283aa with 25 gaps, 0 stops, absolute frame= Plus2 at 2409266..2410114 in the same strand
misc_feature	no id	Integrase core domain; Region: rve; cl01316 intersects 100%(110aa) with IS3_IS51_ORF1 e-value= 6.3e-51 complete sequence hit coverage= 100%, between model(307 aa) positions 1; 307 length is 283aa with 25 gaps, 0 stops, absolute frame= Plus2 at 2409266..2410114 in the same strand
misc_feature	no id	Integrase core domain; Region: rve_3; cl15866 intersects 100%(68aa) with IS3_IS51_ORF1 e-value= 6.3e-51 complete sequence hit coverage= 100%, between model(307 aa) positions 1; 307 length is 283aa with 25 gaps, 0 stops, absolute frame= Plus2 at 2409266..2410114 in the same strand
CDS	YP_001516727.1		transposase intersects 3.27%(3aa) with IS3_IS3_ORF1	e-value= 2.5e-54 complete sequence hit coverage= 100%, between model(286 aa) positions 1; 286 length is 272aa with 15 gaps, 0 stops, absolute frame= Plus2 at 2409269..2410084 in the same strand
CDS	YP_001516728.1		integrase catalytic subunit intersects 94.76%(271aa) with IS3_IS3_ORF1	e-value= 2.5e-54 complete sequence hit coverage= 100%, between model(286 aa) positions 1; 286 length is 272aa with 15 gaps, 0 stops, absolute frame= Plus2 at 2409269..2410084 in the same strand
misc_feature	no id	putative transposase OrfB; Reviewed; Region: PHA02517 intersects 98.43%(250aa) with IS3_IS3_ORF1	e-value= 2.5e-54 complete sequence hit coverage= 100%, between model(286 aa) positions 1; 286 length is 272aa with 15 gaps, 0 stops, absolute frame= Plus2 at 2409269..2410084 in the same strand
misc_feature	no id	HTH-like domain; Region: HTH_21; pfam13276 intersects 100%(42aa) with IS3_IS3_ORF1 e-value= 2.5e-54 complete sequence hit coverage= 100%, between model(286 aa) positions 1; 286 length is 272aa with 15 gaps, 0 stops, absolute frame= Plus2 at 2409269..2410084 in the same strand
misc_feature	no id	Integrase core domain; Region: rve; cl01316 intersects 100%(110aa) with IS3_IS3_ORF1 e-value= 2.5e-54 complete sequence hit coverage= 100%, between model(286 aa) positions 1; 286 length is 272aa with 15 gaps, 0 stops, absolute frame= Plus2 at 2409269..2410084 in the same strand
misc_feature	no id	Integrase core domain; Region: rve_3; cl15866 intersects 100%(68aa) with IS3_IS3_ORF1 e-value= 2.5e-54 complete sequence hit coverage= 100%, between model(286 aa) positions 1; 286 length is 272aa with 15 gaps, 0 stops, absolute frame= Plus2 at 2409269..2410084 in the same strand
CDS	YP_001516727.1		transposase intersects 2.29%(2aa) with IS3_IS150_ORF1	e-value= 6.3e-50 fragment hit coverage= 92.81%, between model(306 aa) positions 23; 306 length is 272aa with 14 gaps, 0 stops, absolute frame= Plus2 at 2409272..2410087 in the same strand
CDS	YP_001516728.1		integrase catalytic subunit intersects 95.10%(272aa) with IS3_IS150_ORF1	e-value= 6.3e-50 fragment hit coverage= 92.81%, between model(306 aa) positions 23; 306 length is 272aa with 14 gaps, 0 stops, absolute frame= Plus2 at 2409272..2410087 in the same strand
misc_feature	no id	putative transposase OrfB; Reviewed; Region: PHA02517 intersects 98.82%(251aa) with IS3_IS150_ORF1	e-value= 6.3e-50 fragment hit coverage= 92.81%, between model(306 aa) positions 23; 306 length is 272aa with 14 gaps, 0 stops, absolute frame= Plus2 at 2409272..2410087 in the same strand
misc_feature	no id	HTH-like domain; Region: HTH_21; pfam13276 intersects 100%(42aa) with IS3_IS150_ORF1	e-value= 6.3e-50 fragment hit coverage= 92.81%, between model(306 aa) positions 23; 306 length is 272aa with 14 gaps, 0 stops, absolute frame= Plus2 at 2409272..2410087 in the same strand
misc_feature	no id	Integrase core domain; Region: rve; cl01316 intersects 100%(110aa) with IS3_IS150_ORF1 e-value= 6.3e-50 fragment hit coverage= 92.81%, between model(306 aa) positions 23; 306 length is 272aa with 14 gaps, 0 stops, absolute frame= Plus2 at 2409272..2410087 in the same strand
misc_feature	no id	Integrase core domain; Region: rve_3; cl15866 intersects 100%(68aa) with IS3_IS150_ORF1 e-value= 6.3e-50 fragment hit coverage= 92.81%, between model(306 aa) positions 23; 306 length is 272aa with 14 gaps, 0 stops, absolute frame= Plus2 at 2409272..2410087 in the same strand
CDS	YP_001516728.1		integrase catalytic subunit intersects 95.10%(272aa) with IS3_IS3_ORF2	e-value= 3.3e-65 complete sequence hit coverage= 100%, between model(288 aa) positions 1; 288 length is 272aa with 19 gaps, 0 stops, absolute frame= Plus2 at 2409281..2410096 in the same strand
misc_feature	no id	putative transposase OrfB; Reviewed; Region: PHA02517 intersects 100%(254aa) with IS3_IS3_ORF2	e-value= 3.3e-65 complete sequence hit coverage= 100%, between model(288 aa) positions 1; 288 length is 272aa with 19 gaps, 0 stops, absolute frame= Plus2 at 2409281..2410096 in the same strand
misc_feature	no id	HTH-like domain; Region: HTH_21; pfam13276 intersects 100%(42aa) with IS3_IS3_ORF2 e-value= 3.3e-65 complete sequence hit coverage= 100%, between model(288 aa) positions 1; 288 length is 272aa with 19 gaps, 0 stops, absolute frame= Plus2 at 2409281..2410096 in the same strand

misc_feature no id Integrase core domain; Region: rve; cl01316 intersects 100%(110aa) with IS3_IS3_ORF2 e-value= 3.3e-65 complete sequence hit coverage= 100%, between model(288 aa) positions 1; 288 length is 272aa with 19 gaps, 0 stops, absolute frame= Plus2 at 2409281..2410096 in the same strand
misc_feature no id Integrase core domain; Region: rve_3; cl15866 intersects 100%(68aa) with IS3_IS3_ORF2 e-value= 3.3e-65 complete sequence hit coverage= 100%, between model(288 aa) positions 1; 288 length is 272aa with 19 gaps, 0 stops, absolute frame= Plus2 at 2409281..2410096 in the same strand
CDS YP_001516728.1 integrase catalytic subunit intersects 18.53%(53aa) with IS3_IS2_ORF2 e-value= 9.1e-09 fragment hit coverage= 18.87%, between model(302 aa) positions 48; 104 length is 53aa with 5 gaps, 0 stops, absolute frame= Plus2 at 2409350..2409508 in the same strand
misc_feature no id putative transposase OrfB; Reviewed; Region: PHA02517 intersects 20.87%(53aa) with IS3_IS2_ORF2 e-value= 9.1e-09 fragment hit coverage= 18.87%, between model(302 aa) positions 48; 104 length is 53aa with 5 gaps, 0 stops, absolute frame= Plus2 at 2409350..2409508 in the same strand
misc_feature no id HTH-like domain; Region: HTH_21; pfam13276 intersects 73.81%(31aa) with IS3_IS2_ORF2 e-value= 9.1e-09 fragment hit coverage= 18.87%, between model(302 aa) positions 48; 104 length is 53aa with 5 gaps, 0 stops, absolute frame= Plus2 at 2409350..2409508 in the same strand
CDS YP_001516728.1 integrase catalytic subunit intersects 79.72%(228aa) with IS3_IS51_ORF2 e-value= 1.9e-38 fragment hit coverage= 74.76%, between model(317 aa) positions 73; 309 length is 228aa with 11 gaps, 0 stops, absolute frame= Plus2 at 2409416..2410099 in the same strand
misc_feature no id putative transposase OrfB; Reviewed; Region: PHA02517 intersects 89.37%(227aa) with IS3_IS51_ORF2 e-value= 1.9e-38 fragment hit coverage= 74.76%, between model(317 aa) positions 73; 309 length is 228aa with 11 gaps, 0 stops, absolute frame= Plus2 at 2409416..2410099 in the same strand
misc_feature no id HTH-like domain; Region: HTH_21; pfam13276 intersects 100%(42aa) with IS3_IS51_ORF2 e-value= 1.9e-38 fragment hit coverage= 74.76%, between model(317 aa) positions 73; 309 length is 228aa with 11 gaps, 0 stops, absolute frame= Plus2 at 2409416..2410099 in the same strand
misc_feature no id Integrase core domain; Region: rve; cl01316 intersects 100%(110aa) with IS3_IS51_ORF2 e-value= 1.9e-38 fragment hit coverage= 74.76%, between model(317 aa) positions 73; 309 length is 228aa with 11 gaps, 0 stops, absolute frame= Plus2 at 2409416..2410099 in the same strand
misc_feature no id Integrase core domain; Region: rve_3; cl15866 intersects 100%(68aa) with IS3_IS51_ORF2 e-value= 1.9e-38 fragment hit coverage= 74.76%, between model(317 aa) positions 73; 309 length is 228aa with 11 gaps, 0 stops, absolute frame= Plus2 at 2409416..2410099 in the same strand
Class A

97.
gene no id fragment; similar to C-terminus of transposase intersects 100%(146aa) with ISAs1 e-value= 1.9e-08 complete sequence hit coverage= 100%, between model(389 aa) positions 1; 389 length is 321aa with 77 gaps, 7 stops, absolute frame= Minus3 at 2412026..2412987 in the same strand
gene no id fragment; similar to N-terminus of transposase intersects 75.57%(144aa) with ISAs1 e-value= 1.9e-08 complete sequence hit coverage= 100%, between model(389 aa) positions 1; 389 length is 321aa with 77 gaps, 7 stops, absolute frame= Minus3 at 2412026..2412987 in the same strand
gene no id fragment; similar to C-terminus of transposase intersects 86.76%(126aa) with ISAs1 e-value= 7e-07 complete sequence hit coverage= 100%, between model(389 aa) positions 1; 389 length is 345aa with 59 gaps, 13 stops, absolute frame= Minus2 at 2412096..2413129 in the same strand
gene no id fragment; similar to N-terminus of transposase intersects 100%(191aa) with ISAs1 e-value= 7e-07 complete sequence hit coverage= 100%, between model(389 aa) positions 1; 389 length is 345aa with 59 gaps, 13 stops, absolute frame= Minus2 at 2412096..2413129 in the same strand
gene no id fragment; similar to C-terminus of transposase intersects 83.56%(122aa) with ISAs1 e-value= 2.4e-27 fragment hit coverage= 41.65%, between model(389 aa) positions 198; 359 length is 148aa with 15 gaps, 0 stops, absolute frame= Minus3 at 2412110..2412552 in the same strand
gene no id fragment; similar to N-terminus of transposase intersects 48.69%(93aa) with ISAs1 e-value= 1.2e-14 fragment hit coverage= 25.45%, between model(389 aa) positions 1; 99 length is 94aa with 5 gaps, 0 stops, absolute frame= Minus2 at 2412849..2413129 in the same strand
Class A

98.
CDS YP_001516771.1 transposase intersects 86.18%(357aa) with ISL3 e-value= 1e-49 fragment hit coverage= 86.03%, between model(451 aa) positions 64; 451 length is 360aa with 34 gaps, 1 stops, absolute frame= Minus2 at 2458710..2459788 in the same strand

misc_feature no id Transposase and inactivated derivatives [DNA replication, recombination, and repair]; Region: COG3464 intersects 85.77%(345aa) with ISL3 e-value= 1e-49 fragment hit coverage= 86.03%, between model(451 aa) positions 64; 451 length is 360aa with 34 gaps, 1 stops, absolute frame= Minus2 at 2458710..2459788 in the same strand
misc_feature no id Transposase; Region: DDE_Tnp_ISL3; pfam01610 intersects 100%(246aa) with ISL3 e-value= 1e-49 fragment hit coverage= 86.03%, between model(451 aa) positions 64; 451 length is 360aa with 34 gaps, 1 stops, absolute frame= Minus2 at 2458710..2459788 in the same strand
CDS YP_001516771.1 transposase intersects 98.96%(410aa) with ISL3 e-value= 1.8e-43 complete sequence hit coverage= 100%, between model(451 aa) positions 1; 451 length is 413aa with 44 gaps, 1 stops, absolute frame= Minus2 at 2458710..2459947 in the same strand
misc_feature no id Transposase and inactivated derivatives [DNA replication, recombination, and repair]; Region: COG3464 intersects 98.92%(398aa) with ISL3 e-value= 1.8e-43 complete sequence hit coverage= 100%, between model(451 aa) positions 1; 451 length is 413aa with 44 gaps, 1 stops, absolute frame= Minus2 at 2458710..2459947 in the same strand
misc_feature no id Transposase; Region: DDE_Tnp_ISL3; pfam01610 intersects 100%(246aa) with ISL3 e-value= 1.8e-43 complete sequence hit coverage= 100%, between model(451 aa) positions 1; 451 length is 413aa with 44 gaps, 1 stops, absolute frame= Minus2 at 2458710..2459947 in the same strand
Class A

99.
CDS YP_001516795.1 IS4 family transposase intersects 98.38%(262aa) with IS5_IS1031 e-value= 2.1e-110 fragment hit coverage= 93.56%, between model(295 aa) positions 20; 295 length is 270aa with 9 gaps, 2 stops, absolute frame= Minus1 at 2497765..2498573 in the same strand
misc_feature no id DDE superfamily endonuclease; Region: DDE_4; cl15789 intersects 100%(83aa) with IS5_IS1031 e-value= 2.1e-110 fragment hit coverage= 93.56%, between model(295 aa) positions 20; 295 length is 270aa with 9 gaps, 2 stops, absolute frame= Minus1 at 2497765..2498573 in the same strand
misc_feature no id Transposase DDE domain; Region: DDE_Tnp_1; pfam01609 intersects 100%(155aa) with IS5_IS1031 e-value= 2.1e-110 fragment hit coverage= 93.56%, between model(295 aa) positions 20; 295 length is 270aa with 9 gaps, 2 stops, absolute frame= Minus1 at 2497765..2498573 in the same strand
misc_feature no id DDE superfamily endonuclease; Region: DDE_4; cl15789 intersects 100%(75aa) with IS5_IS1031 e-value= 2.1e-110 fragment hit coverage= 93.56%, between model(295 aa) positions 20; 295 length is 270aa with 9 gaps, 2 stops, absolute frame= Minus1 at 2497765..2498573 in the same strand
CDS YP_001516795.1 IS4 family transposase intersects 100%(267aa) with IS5_IS1031 e-value= 3.2e-108 complete sequence hit coverage= 100%, between model(295 aa) positions 1; 295 length is 284aa with 14 gaps, 3 stops, absolute frame= Minus1 at 2497765..2498615 in the same strand
misc_feature no id DDE superfamily endonuclease; Region: DDE_4; cl15789 intersects 100%(83aa) with IS5_IS1031 e-value= 3.2e-108 complete sequence hit coverage= 100%, between model(295 aa) positions 1; 295 length is 284aa with 14 gaps, 3 stops, absolute frame= Minus1 at 2497765..2498615 in the same strand
misc_feature no id Transposase DDE domain; Region: DDE_Tnp_1; pfam01609 intersects 100%(155aa) with IS5_IS1031 e-value= 3.2e-108 complete sequence hit coverage= 100%, between model(295 aa) positions 1; 295 length is 284aa with 14 gaps, 3 stops, absolute frame= Minus1 at 2497765..2498615 in the same strand
misc_feature no id DDE superfamily endonuclease; Region: DDE_4; cl15789 intersects 100%(75aa) with IS5_IS1031 e-value= 3.2e-108 complete sequence hit coverage= 100%, between model(295 aa) positions 1; 295 length is 284aa with 14 gaps, 3 stops, absolute frame= Minus1 at 2497765..2498615 in the same strand
CDS YP_001516795.1 IS4 family transposase intersects 56.55%(151aa) with IS5_IS427 e-value= 7.3e-07 complete sequence hit coverage= 100%, between model(296 aa) positions 1; 296 length is 262aa with 39 gaps, 5 stops, absolute frame= Minus1 at 2498134..2498918 in the same strand
misc_feature no id Transposase DDE domain; Region: DDE_Tnp_1; pfam01609 intersects 30.32%(47aa) with IS5_IS427 e-value= 7.3e-07 complete sequence hit coverage= 100%, between model(296 aa) positions 1; 296 length is 262aa with 39 gaps, 5 stops, absolute frame= Minus1 at 2498134..2498918 in the same strand
misc_feature no id DDE superfamily endonuclease; Region: DDE_4; cl15789 intersects 100%(75aa) with IS5_IS427 e-value= 7.3e-07 complete sequence hit coverage= 100%, between model(296 aa) positions 1; 296 length is 262aa with 39 gaps, 5 stops, absolute frame= Minus1 at 2498134..2498918 in the same strand
CDS YP_001516796.1 hypothetical protein intersects 28.10%(71aa) with IS5_IS427 e-value= 7.3e-07 complete sequence hit coverage= 100%, between model(296 aa) positions 1; 296 length is 262aa with 39 gaps, 5 stops, absolute frame= Minus1 at 2498134..2498918 in the opposite strand

misc_feature no id Forkhead associated domain (FHA); found in eukaryotic and prokaryotic proteins. Putative nuclear signalling domain. FHA domains may bind phosphothreonine, phosphoserine and sometimes phosphotyrosine. In eukaryotes, many FHA domain-containing proteins...; Region: FHA; cl00062 intersects 55.97%(29aa) with IS5_IS427 e-value= 7.3e-07 complete sequence hit coverage= 100%, between model(296 aa) positions 1; 296 length is 262aa with 39 gaps, 5 stops, absolute frame= Minus1 at 2498134..2498918 in the opposite strand
CDS YP_001516795.1 IS4 family transposase intersects 29.84%(79aa) with IS5_IS427 e-value= 3.3e-18 fragment hit coverage= 27.70%, between model(296 aa) positions 125; 206 length is 80aa with 2 gaps, 0 stops, absolute frame= Minus1 at 2498326..2498564 in the same strand
misc_feature no id DDE superfamily endonuclease; Region: DDE_4; cl15789 intersects 100%(75aa) with IS5_IS427 e-value= 3.3e-18 fragment hit coverage= 27.70%, between model(296 aa) positions 125; 206 length is 80aa with 2 gaps, 0 stops, absolute frame= Minus1 at 2498326..2498564 in the same strand
Class A

100.
CDS YP_001516798.1 transposase intersects 81.70%(83aa) with IS3_IS150_ORF2 e-value= 1.7e-22 complete sequence hit coverage= 100%, between model(389 aa) positions 1; 389 length is 354aa with 39 gaps, 5 stops, absolute frame= Plus2 at 2500838..2501899 in the same strand
misc_feature no id Helix-turn-helix domains; Region: HTH; cl00088 intersects 79.11%(59aa) with IS3_IS150_ORF2 e-value= 1.7e-22 complete sequence hit coverage= 100%, between model(389 aa) positions 1; 389 length is 354aa with 39 gaps, 5 stops, absolute frame= Plus2 at 2500838..2501899 in the same strand
CDS YP_001516799.1 integrase catalytic subunit intersects 95.45%(273aa) with IS3_IS150_ORF2 e-value= 1.7e-22 complete sequence hit coverage= 100%, between model(389 aa) positions 1; 389 length is 354aa with 39 gaps, 5 stops, absolute frame= Plus2 at 2500838..2501899 in the same strand
misc_feature no id putative transposase OrfB; Reviewed; Region: PHA02517 intersects 99.21%(252aa) with IS3_IS150_ORF2 e-value= 1.7e-22 complete sequence hit coverage= 100%, between model(389 aa) positions 1; 389 length is 354aa with 39 gaps, 5 stops, absolute frame= Plus2 at 2500838..2501899 in the same strand
misc_feature no id HTH-like domain; Region: HTH_21; pfam13276 intersects 100%(42aa) with IS3_IS150_ORF2 e-value= 1.7e-22 complete sequence hit coverage= 100%, between model(389 aa) positions 1; 389 length is 354aa with 39 gaps, 5 stops, absolute frame= Plus2 at 2500838..2501899 in the same strand
misc_feature no id Integrase core domain; Region: rve; cl01316 intersects 100%(110aa) with IS3_IS150_ORF2 e-value= 1.7e-22 complete sequence hit coverage= 100%, between model(389 aa) positions 1; 389 length is 354aa with 39 gaps, 5 stops, absolute frame= Plus2 at 2500838..2501899 in the same strand
misc_feature no id Integrase core domain; Region: rve_3; cl15866 intersects 100%(68aa) with IS3_IS150_ORF2 e-value= 1.7e-22 complete sequence hit coverage= 100%, between model(389 aa) positions 1; 389 length is 354aa with 39 gaps, 5 stops, absolute frame= Plus2 at 2500838..2501899 in the same strand
CDS YP_001516798.1 transposase intersects 24.84%(25aa) with IS3_IS150_ORF1 e-value= 4.3e-49 complete sequence hit coverage= 100%, between model(306 aa) positions 1; 306 length is 295aa with 14 gaps, 0 stops, absolute frame= Plus2 at 2501012..2501896 in the same strand
misc_feature no id Helix-turn-helix domains; Region: HTH; cl00088 intersects 1.78%(1aa) with IS3_IS150_ORF1 e-value= 4.3e-49 complete sequence hit coverage= 100%, between model(306 aa) positions 1; 306 length is 295aa with 14 gaps, 0 stops, absolute frame= Plus2 at 2501012..2501896 in the same strand
CDS YP_001516799.1 integrase catalytic subunit intersects 95.10%(272aa) with IS3_IS150_ORF1 e-value= 4.3e-49 complete sequence hit coverage= 100%, between model(306 aa) positions 1; 306 length is 295aa with 14 gaps, 0 stops, absolute frame= Plus2 at 2501012..2501896 in the same strand
misc_feature no id putative transposase OrfB; Reviewed; Region: PHA02517 intersects 98.82%(251aa) with IS3_IS150_ORF1 e-value= 4.3e-49 complete sequence hit coverage= 100%, between model(306 aa) positions 1; 306 length is 295aa with 14 gaps, 0 stops, absolute frame= Plus2 at 2501012..2501896 in the same strand
misc_feature no id HTH-like domain; Region: HTH_21; pfam13276 intersects 100%(42aa) with IS3_IS150_ORF1 e-value= 4.3e-49 complete sequence hit coverage= 100%, between model(306 aa) positions 1; 306 length is 295aa with 14 gaps, 0 stops, absolute frame= Plus2 at 2501012..2501896 in the same strand
misc_feature no id Integrase core domain; Region: rve; cl01316 intersects 100%(110aa) with IS3_IS150_ORF1 e-value= 4.3e-49 complete sequence hit coverage= 100%, between model(306 aa) positions 1; 306 length is 295aa with 14 gaps, 0 stops, absolute frame= Plus2 at 2501012..2501896 in the same strand
misc_feature no id Integrase core domain; Region: rve_3; cl15866 intersects 100%(68aa) with IS3_IS150_ORF1 e-value= 4.3e-49 complete sequence hit coverage= 100%, between model(306 aa) positions 1; 306 length is 295aa with 14 gaps, 0 stops, absolute frame= Plus2 at 2501012..2501896 in the same strand

CDS YP_001516798.1 transposase intersects 13.07%(13aa) with IS3_IS51_ORF2 e-value= 1.6e-26 complete sequence hit coverage= 100%, between model(317 aa) positions 1; 317 length is 295aa with 27 gaps, 0 stops, absolute frame= Plus2 at 2501048..2501932 in the same strand
CDS YP_001516799.1 integrase catalytic subunit intersects 99.30%(284aa) with IS3_IS51_ORF2 e-value= 1.6e-26 complete sequence hit coverage= 100%, between model(317 aa) positions 1; 317 length is 295aa with 27 gaps, 0 stops, absolute frame= Plus2 at 2501048..2501932 in the same strand
misc_feature no id putative transposase OrfB; Reviewed; Region: PHA02517 intersects 100%(254aa) with IS3_IS51_ORF2 e-value= 1.6e-26 complete sequence hit coverage= 100%, between model(317 aa) positions 1; 317 length is 295aa with 27 gaps, 0 stops, absolute frame= Plus2 at 2501048..2501932 in the same strand
misc_feature no id HTH-like domain; Region: HTH_21; pfam13276 intersects 100%(42aa) with IS3_IS51_ORF2 e-value= 1.6e-26 complete sequence hit coverage= 100%, between model(317 aa) positions 1; 317 length is 295aa with 27 gaps, 0 stops, absolute frame= Plus2 at 2501048..2501932 in the same strand
misc_feature no id Integrase core domain; Region: rve; cl01316 intersects 100%(110aa) with IS3_IS51_ORF2 e-value= 1.6e-26 complete sequence hit coverage= 100%, between model(317 aa) positions 1; 317 length is 295aa with 27 gaps, 0 stops, absolute frame= Plus2 at 2501048..2501932 in the same strand
misc_feature no id Integrase core domain; Region: rve_3; cl15866 intersects 100%(68aa) with IS3_IS51_ORF2 e-value= 1.6e-26 complete sequence hit coverage= 100%, between model(317 aa) positions 1; 317 length is 295aa with 27 gaps, 0 stops, absolute frame= Plus2 at 2501048..2501932 in the same strand
CDS YP_001516798.1 transposase intersects 11.11%(11aa) with IS3_IS407_ORF2 e-value= 1.2e-17 fragment hit coverage= 25.29%, between model(344 aa) positions 60; 146 length is 85aa with 2 gaps, 0 stops, absolute frame= Plus2 at 2501054..2501308 in the same strand
CDS YP_001516799.1 integrase catalytic subunit intersects 26.57%(76aa) with IS3_IS407_ORF2 e-value= 1.2e-17 fragment hit coverage= 25.29%, between model(344 aa) positions 60; 146 length is 85aa with 2 gaps, 0 stops, absolute frame= Plus2 at 2501054..2501308 in the same strand
misc_feature no id putative transposase OrfB; Reviewed; Region: PHA02517 intersects 21.65%(55aa) with IS3_IS407_ORF2 e-value= 1.2e-17 fragment hit coverage= 25.29%, between model(344 aa) positions 60; 146 length is 85aa with 2 gaps, 0 stops, absolute frame= Plus2 at 2501054..2501308 in the same strand
misc_feature no id HTH-like domain; Region: HTH_21; pfam13276 intersects 66.67%(28aa) with IS3_IS407_ORF2 e-value= 1.2e-17 fragment hit coverage= 25.29%, between model(344 aa) positions 60; 146 length is 85aa with 2 gaps, 0 stops, absolute frame= Plus2 at 2501054..2501308 in the same strand
CDS YP_001516798.1 transposase intersects 5.23%(5aa) with IS3_IS150_ORF2 e-value= 2.2e-47 fragment hit coverage= 76.09%, between model(389 aa) positions 94; 389 length is 276aa with 23 gaps, 0 stops, absolute frame= Plus2 at 2501072..2501899 in the same strand
CDS YP_001516799.1 integrase catalytic subunit intersects 95.45%(273aa) with IS3_IS150_ORF2 e-value= 2.2e-47 fragment hit coverage= 76.09%, between model(389 aa) positions 94; 389 length is 276aa with 23 gaps, 0 stops, absolute frame= Plus2 at 2501072..2501899 in the same strand
misc_feature no id putative transposase OrfB; Reviewed; Region: PHA02517 intersects 99.21%(252aa) with IS3_IS150_ORF2 e-value= 2.2e-47 fragment hit coverage= 76.09%, between model(389 aa) positions 94; 389 length is 276aa with 23 gaps, 0 stops, absolute frame= Plus2 at 2501072..2501899 in the same strand
misc_feature no id HTH-like domain; Region: HTH_21; pfam13276 intersects 100%(42aa) with IS3_IS150_ORF2 e-value= 2.2e-47 fragment hit coverage= 76.09%, between model(389 aa) positions 94; 389 length is 276aa with 23 gaps, 0 stops, absolute frame= Plus2 at 2501072..2501899 in the same strand
misc_feature no id Integrase core domain; Region: rve; cl01316 intersects 100%(110aa) with IS3_IS150_ORF2 e-value= 2.2e-47 fragment hit coverage= 76.09%, between model(389 aa) positions 94; 389 length is 276aa with 23 gaps, 0 stops, absolute frame= Plus2 at 2501072..2501899 in the same strand
misc_feature no id Integrase core domain; Region: rve_3; cl15866 intersects 100%(68aa) with IS3_IS150_ORF2 e-value= 2.2e-47 fragment hit coverage= 76.09%, between model(389 aa) positions 94; 389 length is 276aa with 23 gaps, 0 stops, absolute frame= Plus2 at 2501072..2501899 in the same strand
CDS YP_001516798.1 transposase intersects 4.25%(4aa) with IS3_IS51_ORF1 e-value= 6.3e-51 complete sequence hit coverage= 100%, between model(307 aa) positions 1; 307 length is 283aa with 25 gaps, 0 stops, absolute frame= Plus2 at 2501075..2501923 in the same strand
CDS YP_001516799.1 integrase catalytic subunit intersects 98.25%(281aa) with IS3_IS51_ORF1 e-value= 6.3e-51 complete sequence hit coverage= 100%, between model(307 aa) positions 1; 307 length is 283aa with 25 gaps, 0 stops, absolute frame= Plus2 at 2501075..2501923 in the same strand
misc_feature no id putative transposase OrfB; Reviewed; Region: PHA02517 intersects 100%(254aa) with IS3_IS51_ORF1 e-value= 6.3e-51 complete sequence hit coverage= 100%, between model(307 aa) positions 1; 307 length is 283aa with 25 gaps, 0 stops, absolute frame= Plus2 at 2501075..2501923 in the same strand

misc_feature no id HTH-like domain; Region: HTH_21; pfam13276 intersects 100%(42aa) with IS3_IS51_ORF1 e-value= 6.3e-51 complete sequence hit coverage= 100%, between model(307 aa) positions 1; 307 length is 283aa with 25 gaps, 0 stops, absolute frame= Plus2 at 2501075..2501923 in the same strand
misc_feature no id Integrase core domain; Region: rve; cl01316 intersects 100%(110aa) with IS3_IS51_ORF1 e-value= 6.3e-51 complete sequence hit coverage= 100%, between model(307 aa) positions 1; 307 length is 283aa with 25 gaps, 0 stops, absolute frame= Plus2 at 2501075..2501923 in the same strand
misc_feature no id Integrase core domain; Region: rve_3; cl15866 intersects 100%(68aa) with IS3_IS51_ORF1 e-value= 6.3e-51 complete sequence hit coverage= 100%, between model(307 aa) positions 1; 307 length is 283aa with 25 gaps, 0 stops, absolute frame= Plus2 at 2501075..2501923 in the same strand
CDS YP_001516798.1 transposase intersects 3.27%(3aa) with IS3_IS3_ORF1 e-value= 2.5e-54 complete sequence hit coverage= 100%, between model(286 aa) positions 1; 286 length is 272aa with 15 gaps, 0 stops, absolute frame= Plus2 at 2501078..2501893 in the same strand
CDS YP_001516799.1 integrase catalytic subunit intersects 94.76%(271aa) with IS3_IS3_ORF1 e-value= 2.5e-54 complete sequence hit coverage= 100%, between model(286 aa) positions 1; 286 length is 272aa with 15 gaps, 0 stops, absolute frame= Plus2 at 2501078..2501893 in the same strand
misc_feature no id putative transposase OrfB; Reviewed; Region: PHA02517 intersects 98.43%(250aa) with IS3_IS3_ORF1 e-value= 2.5e-54 complete sequence hit coverage= 100%, between model(286 aa) positions 1; 286 length is 272aa with 15 gaps, 0 stops, absolute frame= Plus2 at 2501078..2501893 in the same strand
misc_feature no id HTH-like domain; Region: HTH_21; pfam13276 intersects 100%(42aa) with IS3_IS3_ORF1 e-value= 2.5e-54 complete sequence hit coverage= 100%, between model(286 aa) positions 1; 286 length is 272aa with 15 gaps, 0 stops, absolute frame= Plus2 at 2501078..2501893 in the same strand
misc_feature no id Integrase core domain; Region: rve; cl01316 intersects 100%(110aa) with IS3_IS3_ORF1 e-value= 2.5e-54 complete sequence hit coverage= 100%, between model(286 aa) positions 1; 286 length is 272aa with 15 gaps, 0 stops, absolute frame= Plus2 at 2501078..2501893 in the same strand
misc_feature no id Integrase core domain; Region: rve_3; cl15866 intersects 100%(68aa) with IS3_IS3_ORF1 e-value= 2.5e-54 complete sequence hit coverage= 100%, between model(286 aa) positions 1; 286 length is 272aa with 15 gaps, 0 stops, absolute frame= Plus2 at 2501078..2501893 in the same strand
CDS YP_001516798.1 transposase intersects 2.29%(2aa) with IS3_IS150_ORF1 e-value= 6.3e-50 fragment hit coverage= 92.81%, between model(306 aa) positions 23; 306 length is 272aa with 14 gaps, 0 stops, absolute frame= Plus2 at 2501081..2501896 in the same strand
CDS YP_001516799.1 integrase catalytic subunit intersects 95.10%(272aa) with IS3_IS150_ORF1 e-value= 6.3e-50 fragment hit coverage= 92.81%, between model(306 aa) positions 23; 306 length is 272aa with 14 gaps, 0 stops, absolute frame= Plus2 at 2501081..2501896 in the same strand
misc_feature no id putative transposase OrfB; Reviewed; Region: PHA02517 intersects 98.82%(251aa) with IS3_IS150_ORF1 e-value= 6.3e-50 fragment hit coverage= 92.81%, between model(306 aa) positions 23; 306 length is 272aa with 14 gaps, 0 stops, absolute frame= Plus2 at 2501081..2501896 in the same strand
misc_feature no id HTH-like domain; Region: HTH_21; pfam13276 intersects 100%(42aa) with IS3_IS150_ORF1 e-value= 6.3e-50 fragment hit coverage= 92.81%, between model(306 aa) positions 23; 306 length is 272aa with 14 gaps, 0 stops, absolute frame= Plus2 at 2501081..2501896 in the same strand
misc_feature no id Integrase core domain; Region: rve; cl01316 intersects 100%(110aa) with IS3_IS150_ORF1 e-value= 6.3e-50 fragment hit coverage= 92.81%, between model(306 aa) positions 23; 306 length is 272aa with 14 gaps, 0 stops, absolute frame= Plus2 at 2501081..2501896 in the same strand
misc_feature no id Integrase core domain; Region: rve_3; cl15866 intersects 100%(68aa) with IS3_IS150_ORF1 e-value= 6.3e-50 fragment hit coverage= 92.81%, between model(306 aa) positions 23; 306 length is 272aa with 14 gaps, 0 stops, absolute frame= Plus2 at 2501081..2501896 in the same strand
CDS YP_001516799.1 integrase catalytic subunit intersects 95.10%(272aa) with IS3_IS3_ORF2 e-value= 3.3e-65 complete sequence hit coverage= 100%, between model(288 aa) positions 1; 288 length is 272aa with 19 gaps, 0 stops, absolute frame= Plus2 at 2501090..2501905 in the same strand
misc_feature no id putative transposase OrfB; Reviewed; Region: PHA02517 intersects 100%(254aa) with IS3_IS3_ORF2 e-value= 3.3e-65 complete sequence hit coverage= 100%, between model(288 aa) positions 1; 288 length is 272aa with 19 gaps, 0 stops, absolute frame= Plus2 at 2501090..2501905 in the same strand
misc_feature no id HTH-like domain; Region: HTH_21; pfam13276 intersects 100%(42aa) with IS3_IS3_ORF2 e-value= 3.3e-65 complete sequence hit coverage= 100%, between model(288 aa) positions 1; 288 length is 272aa with 19 gaps, 0 stops, absolute frame= Plus2 at 2501090..2501905 in the same strand
misc_feature no id Integrase core domain; Region: rve; cl01316 intersects 100%(110aa) with IS3_IS3_ORF2 e-value= 3.3e-65 complete sequence hit coverage= 100%, between model(288 aa) positions 1; 288 length is 272aa with 19 gaps, 0 stops, absolute frame= Plus2 at 2501090..2501905 in the same strand

misc_feature no id Integrase core domain; Region: rve_3; cl15866 intersects 100%(68aa) with IS3_IS3_ORF2 e-value= 3.3e-65 complete sequence hit coverage= 100%, between model(288 aa) positions 1; 288 length is 272aa with 19 gaps, 0 stops, absolute frame= Plus2 at 2501090..2501905 in the same strand
CDS YP_001516799.1 integrase catalytic subunit intersects 18.53%(53aa) with IS3_IS2_ORF2 e-value= 9.1e-09 fragment hit coverage= 18.87%, between model(302 aa) positions 48; 104 length is 53aa with 5 gaps, 0 stops, absolute frame= Plus2 at 2501159..2501317 in the same strand
misc_feature no id putative transposase OrfB; Reviewed; Region: PHA02517 intersects 20.87%(53aa) with IS3_IS2_ORF2 e-value= 9.1e-09 fragment hit coverage= 18.87%, between model(302 aa) positions 48; 104 length is 53aa with 5 gaps, 0 stops, absolute frame= Plus2 at 2501159..2501317 in the same strand
misc_feature no id HTH-like domain; Region: HTH_21; pfam13276 intersects 73.81%(31aa) with IS3_IS2_ORF2 e-value= 9.1e-09 fragment hit coverage= 18.87%, between model(302 aa) positions 48; 104 length is 53aa with 5 gaps, 0 stops, absolute frame= Plus2 at 2501159..2501317 in the same strand
CDS YP_001516799.1 integrase catalytic subunit intersects 79.72%(228aa) with IS3_IS51_ORF2 e-value= 1.9e-38 fragment hit coverage= 74.76%, between model(317 aa) positions 73; 309 length is 228aa with 11 gaps, 0 stops, absolute frame= Plus2 at 2501225..2501908 in the same strand
misc_feature no id putative transposase OrfB; Reviewed; Region: PHA02517 intersects 89.37%(227aa) with IS3_IS51_ORF2 e-value= 1.9e-38 fragment hit coverage= 74.76%, between model(317 aa) positions 73; 309 length is 228aa with 11 gaps, 0 stops, absolute frame= Plus2 at 2501225..2501908 in the same strand
misc_feature no id HTH-like domain; Region: HTH_21; pfam13276 intersects 100%(42aa) with IS3_IS51_ORF2 e-value= 1.9e-38 fragment hit coverage= 74.76%, between model(317 aa) positions 73; 309 length is 228aa with 11 gaps, 0 stops, absolute frame= Plus2 at 2501225..2501908 in the same strand
misc_feature no id Integrase core domain; Region: rve; cl01316 intersects 100%(110aa) with IS3_IS51_ORF2 e-value= 1.9e-38 fragment hit coverage= 74.76%, between model(317 aa) positions 73; 309 length is 228aa with 11 gaps, 0 stops, absolute frame= Plus2 at 2501225..2501908 in the same strand
misc_feature no id Integrase core domain; Region: rve_3; cl15866 intersects 100%(68aa) with IS3_IS51_ORF2 e-value= 1.9e-38 fragment hit coverage= 74.76%, between model(317 aa) positions 73; 309 length is 228aa with 11 gaps, 0 stops, absolute frame= Plus2 at 2501225..2501908 in the same strand
Class A

101.
misc_feature no id Transposase and inactivated derivatives [DNA replication, recombination, and repair]; Region: COG3677 intersects 100%(88aa) with IS1_ORF1 e-value= 3.2e-11 fragment hit coverage= 37.24%, between model(239 aa) positions 5; 93 length is 94aa with 2 gaps, 0 stops, absolute frame= Plus1 at 2559835..2560116 in the same strand
CDS YP_001516847.1 hypothetical protein intersects 88.57%(93aa) with IS1_ORF1 e-value= 3.2e-11 fragment hit coverage= 37.24%, between model(239 aa) positions 5; 93 length is 94aa with 2 gaps, 0 stops, absolute frame= Plus1 at 2559835..2560116 in the same strand
Class A

102.
CDS YP_001516871.1 hypothetical protein intersects 27.24%(50aa) with IS630 e-value= 4.3e-06 fragment hit coverage= 11.70%, between model(376 aa) positions 279; 322 length is 51aa with 1 gaps, 0 stops, absolute frame= Minus2 at 2586240..2586391 in the same strand
misc_feature no id Integrase core domain; Region: rve; cl01316 intersects 38.10%(50aa) with IS630 e-value= 4.3e-06 fragment hit coverage= 11.70%, between model(376 aa) positions 279; 322 length is 51aa with 1 gaps, 0 stops, absolute frame= Minus2 at 2586240..2586391 in the same strand
Class A

103.
CDS YP_001516872.1 hypothetical protein intersects 76.22%(135aa) with IS630 e-value= 1.9e-29 fragment hit coverage= 37.50%, between model(376 aa) positions 30; 170 length is 136aa with 5 gaps, 0 stops, absolute frame= Minus1 at 2586673..2587079 in the same strand
misc_feature no id Winged helix-turn helix; Region: HTH_33; pfam13592 intersects 98.33%(59aa) with IS630 e-value= 1.9e-29 fragment hit coverage= 37.50%, between model(376 aa) positions 30; 170 length is 136aa with 5 gaps, 0 stops, absolute frame= Minus1 at 2586673..2587079 in the same strand

misc_feature no id Winged helix-turn helix; Region: HTH_29; pfam13551 intersects 99.70%(111aa) with IS630 e-value= 1.9e-29 fragment hit coverage= 37.50%, between model(376 aa) positions 30; 170 length is 136aa with 5 gaps, 0 stops, absolute frame= Minus1 at 2586673..2587079 in the same strand
misc_feature no id Helix-turn-helix domains; Region: HTH; cl00088 intersects 92.91%(43aa) with IS630 e-value= 1.9e-29 fragment hit coverage= 37.50%, between model(376 aa) positions 30; 170 length is 136aa with 5 gaps, 0 stops, absolute frame= Minus1 at 2586673..2587079 in the same strand
CDS YP_001516872.1 hypothetical protein intersects 44.76%(79aa) with IS481 e-value= 1.2e-07 fragment hit coverage= 23.36%, between model(351 aa) positions 1; 82 length is 80aa with 2 gaps, 0 stops, absolute frame= Minus1 at 2586868..2587106 in the same strand
misc_feature no id Winged helix-turn helix; Region: HTH_29; pfam13551 intersects 63.39%(71aa) with IS481 e-value= 1.2e-07 fragment hit coverage= 23.36%, between model(351 aa) positions 1; 82 length is 80aa with 2 gaps, 0 stops, absolute frame= Minus1 at 2586868..2587106 in the same strand
misc_feature no id Helix-turn-helix domains; Region: HTH; cl00088 intersects 100%(47aa) with IS481 e-value= 1.2e-07 fragment hit coverage= 23.36%, between model(351 aa) positions 1; 82 length is 80aa with 2 gaps, 0 stops, absolute frame= Minus1 at 2586868..2587106 in the same strand
Class B

104.
CDS YP_001516911.1 transposase intersects 86.04%(376aa) with IS4 e-value= 9.9e-07 complete sequence hit coverage= 100%, between model(486 aa) positions 1; 486 length is 376aa with 117 gaps, 0 stops, absolute frame= Plus2 at 2629103..2630230 in the same strand
misc_feature no id DDE superfamily endonuclease; Region: DDE_5; cl02413 intersects 93.57%(349aa) with IS4 e-value= 9.9e-07 complete sequence hit coverage= 100%, between model(486 aa) positions 1; 486 length is 376aa with 117 gaps, 0 stops, absolute frame= Plus2 at 2629103..2630230 in the same strand
CDS YP_001516911.1 transposase intersects 53.09%(232aa) with IS4 e-value= 4.7e-14 fragment hit coverage= 51.23%, between model(486 aa) positions 1; 249 length is 232aa with 27 gaps, 0 stops, absolute frame= Plus2 at 2629103..2629798 in the same strand
misc_feature no id DDE superfamily endonuclease; Region: DDE_5; cl02413 intersects 62.20%(232aa) with IS4 e-value= 4.7e-14 fragment hit coverage= 51.23%, between model(486 aa) positions 1; 249 length is 232aa with 27 gaps, 0 stops, absolute frame= Plus2 at 2629103..2629798 in the same strand
Class A

105.
CDS YP_001516912.1 hypothetical protein intersects 32.32%(53aa) with IS481 e-value= 1.1e-08 fragment hit coverage= 15.10%, between model(351 aa) positions 1; 53 length is 53aa with 0 gaps, 0 stops, absolute frame= Plus1 at 2630473..2630631 in the same strand
misc_feature no id Helix-turn-helix domains; Region: HTH; cl00088 intersects 100%(50aa) with IS481 e-value= 1.1e-08 fragment hit coverage= 15.10%, between model(351 aa) positions 1; 53 length is 53aa with 0 gaps, 0 stops, absolute frame= Plus1 at 2630473..2630631 in the same strand
misc_feature no id Winged helix-turn helix; Region: HTH_29; pfam13551 intersects 45.36%(44aa) with IS481 e-value= 1.1e-08 fragment hit coverage= 15.10%, between model(351 aa) positions 1; 53 length is 53aa with 0 gaps, 0 stops, absolute frame= Plus1 at 2630473..2630631 in the same strand
Class B

106.
CDS YP_001516913.1 hypothetical protein intersects 65.71%(138aa) with IS630 e-value= 1.1e-17 fragment hit coverage= 38.56%, between model(376 aa) positions 232; 376 length is 138aa with 7 gaps, 0 stops, absolute frame= Plus2 at 2631026..2631439 in the same strand
misc_feature no id Integrase core domain; Region: rve; cl01316 intersects 78.10%(107aa) with IS630 e-value= 1.1e-17 fragment hit coverage= 38.56%, between model(376 aa) positions 232; 376 length is 138aa with 7 gaps, 0 stops, absolute frame= Plus2 at 2631026..2631439 in the same strand
Class A

107.
CDS YP_001516914.1 transposase intersects 85.96%(375aa) with IS4 e-value= 9.9e-07 complete sequence hit coverage= 100%, between model(486 aa) positions 1; 486 length is 376aa with 117 gaps, 0 stops, absolute frame= Minus2 at 2631567..2632693 in the same strand

misc_feature	no id	DDE superfamily endonuclease; Region: DDE_5; cl02413 intersects 93.48%(348aa) with IS4	e-value= 9.9e-07 complete sequence hit coverage= 100%, between model(486 aa) positions 1; 486 length is 376aa with 117 gaps, 0 stops, absolute frame= Minus2 at 2631567..2632693 in the same strand
CDS	YP_001516914.1	transposase intersects 53.01%(231aa) with IS4	e-value= 4.7e-14 fragment hit coverage= 51.23%, between model(486 aa) positions 1; 249 length is 232aa with 27 gaps, 0 stops, absolute frame= Minus2 at 2631999..2632693 in the same strand
misc_feature	no id	DDE superfamily endonuclease; Region: DDE_5; cl02413 intersects 62.11%(231aa) with IS4	e-value= 4.7e-14 fragment hit coverage= 51.23%, between model(486 aa) positions 1; 249 length is 232aa with 27 gaps, 0 stops, absolute frame= Minus2 at 2631999..2632693 in the same strand
Class A

108.
CDS	YP_001516987.1	hypothetical protein intersects 99.30%(332aa) with IS630	e-value= 1.1e-19 complete sequence hit coverage= 100%, between model(376 aa) positions 1; 376 length is 345aa with 36 gaps, 1 stops, absolute frame= Minus1 at 2705968..2707001 in the same strand
misc_feature	no id	Integrase core domain; Region: rve; cl01316 intersects 100%(143aa) with IS630	e-value= 1.1e-19 complete sequence hit coverage= 100%, between model(376 aa) positions 1; 376 length is 345aa with 36 gaps, 1 stops, absolute frame= Minus1 at 2705968..2707001 in the same strand
misc_feature	no id	FinO bacterial conjugation repressor domain; the basic protein FinO is part of the the two component FinOP system which is responsible for repressing bacterial conjugation; the FinOP system represses the transfer (tra) operon of the F-plasmid which...; Region: FinO_conjug_rep; cl15270 intersects 100%(65aa) with IS630	e-value= 1.1e-19 complete sequence hit coverage= 100%, between model(376 aa) positions 1; 376 length is 345aa with 36 gaps, 1 stops, absolute frame= Minus1 at 2705968..2707001 in the same strand
misc_feature	no id	Winged helix-turn helix; Region: HTH_29; pfam13551 intersects 100%(99aa) with IS630	e-value= 1.1e-19 complete sequence hit coverage= 100%, between model(376 aa) positions 1; 376 length is 345aa with 36 gaps, 1 stops, absolute frame= Minus1 at 2705968..2707001 in the same strand
misc_feature	no id	Helix-turn-helix domains; Region: HTH; cl00088 intersects 100%(72aa) with IS630	e-value= 1.1e-19 complete sequence hit coverage= 100%, between model(376 aa) positions 1; 376 length is 345aa with 36 gaps, 1 stops, absolute frame= Minus1 at 2705968..2707001 in the same strand
misc_feature	no id	Helix-turn-helix domains; Region: HTH; cl00088 intersects 100%(52aa) with IS630	e-value= 1.1e-19 complete sequence hit coverage= 100%, between model(376 aa) positions 1; 376 length is 345aa with 36 gaps, 1 stops, absolute frame= Minus1 at 2705968..2707001 in the same strand
CDS	YP_001516987.1	hypothetical protein intersects 89.75%(300aa) with IS630	e-value= 1.1e-24 fragment hit coverage= 88.56%, between model(376 aa) positions 1; 333 length is 301aa with 36 gaps, 0 stops, absolute frame= Minus1 at 2706100..2707001 in the same strand
misc_feature	no id	Integrase core domain; Region: rve; cl01316 intersects 92.31%(132aa) with IS630	e-value= 1.1e-24 fragment hit coverage= 88.56%, between model(376 aa) positions 1; 333 length is 301aa with 36 gaps, 0 stops, absolute frame= Minus1 at 2706100..2707001 in the same strand
misc_feature	no id	FinO bacterial conjugation repressor domain; the basic protein FinO is part of the the two component FinOP system which is responsible for repressing bacterial conjugation; the FinOP system represses the transfer (tra) operon of the F-plasmid which...; Region: FinO_conjug_rep; cl15270 intersects 100%(65aa) with IS630	e-value= 1.1e-24 fragment hit coverage= 88.56%, between model(376 aa) positions 1; 333 length is 301aa with 36 gaps, 0 stops, absolute frame= Minus1 at 2706100..2707001 in the same strand
misc_feature	no id	Winged helix-turn helix; Region: HTH_29; pfam13551 intersects 100%(99aa) with IS630	e-value= 1.1e-24 fragment hit coverage= 88.56%, between model(376 aa) positions 1; 333 length is 301aa with 36 gaps, 0 stops, absolute frame= Minus1 at 2706100..2707001 in the same strand
misc_feature	no id	Helix-turn-helix domains; Region: HTH; cl00088 intersects 100%(72aa) with IS630	e-value= 1.1e-24 fragment hit coverage= 88.56%, between model(376 aa) positions 1; 333 length is 301aa with 36 gaps, 0 stops, absolute frame= Minus1 at 2706100..2707001 in the same strand
misc_feature	no id	Helix-turn-helix domains; Region: HTH; cl00088 intersects 100%(52aa) with IS630	e-value= 1.1e-24 fragment hit coverage= 88.56%, between model(376 aa) positions 1; 333 length is 301aa with 36 gaps, 0 stops, absolute frame= Minus1 at 2706100..2707001 in the same strand
Class A

109.

gene no id fragment; similar to C-terminus of transposase intersects 78.68%(107aa) with IS630 e-value= 7.3e-15 fragment hit coverage= 42.02%, between model(376 aa) positions 173; 330 length is 139aa with 21 gaps, 0 stops, absolute frame= Minus3 at 2709254..2709669 in the same strand
Class A

110.
gene no id conserved hypothetical protein; transposase, putative intersects 18.68%(66aa) with IS630 e-value= 1.4e-09 fragment hit coverage= 19.15%, between model(376 aa) positions 275; 346 length is 66aa with 6 gaps, 0 stops, absolute frame= Plus2 at 2714843..2715040 in the same strand
Class A

111.
CDS YP_001517124.1 chain length determinant protein intersects 8.28%(37aa) with IS630 e-value= 5.3e-21 complete sequence hit coverage= 100%, between model(376 aa) positions 1; 376 length is 345aa with 36 gaps, 2 stops, absolute frame= Plus2 at 2845484..2846518 in the opposite strand
misc_feature no id Uncharacterized protein involved in exopolysaccharide biosynthesis [Cell envelope biogenesis, outer membrane]; Region: GumC; COG3206 intersects 3.05%(11aa) with IS630 e-value= 5.3e-21 complete sequence hit coverage= 100%, between model(376 aa) positions 1; 376 length is 345aa with 36 gaps, 2 stops, absolute frame= Plus2 at 2845484..2846518 in the opposite strand
misc_feature no id The Bin/Amphiphysin/Rvs (BAR) domain, a dimerization module that binds membranes and detects membrane curvature; Region: BAR; cl12013 intersects 7.09%(9aa) with IS630 e-value= 5.3e-21 complete sequence hit coverage= 100%, between model(376 aa) positions 1; 376 length is 345aa with 36 gaps, 2 stops, absolute frame= Plus2 at 2845484..2846518 in the opposite strand
Class B

112.
CDS YP_001517188.1 IS4 family transposase intersects 99.35%(308aa) with IS5_IS903 e-value= 2.2e-96 complete sequence hit coverage= 100%, between model(347 aa) positions 1; 347 length is 314aa with 37 gaps, 1 stops, absolute frame= Plus3 at 2912997..2913938 in the same strand
misc_feature no id Transposase DDE domain; Region: DDE_Tnp_1_5; pfam13737 intersects 100%(112aa) with IS5_IS903 e-value= 2.2e-96 complete sequence hit coverage= 100%, between model(347 aa) positions 1; 347 length is 314aa with 37 gaps, 1 stops, absolute frame= Plus3 at 2912997..2913938 in the same strand
misc_feature no id Transposase DDE domain; Region: DDE_Tnp_1; pfam01609 intersects 100%(186aa) with IS5_IS903 e-value= 2.2e-96 complete sequence hit coverage= 100%, between model(347 aa) positions 1; 347 length is 314aa with 37 gaps, 1 stops, absolute frame= Plus3 at 2912997..2913938 in the same strand
Class A

113.
gene no id fragment; similar to N-terminus of transposase intersects 89.10%(139aa) with IS5_ISL2 e-value= 2.8e-20 complete sequence hit coverage= 100%, between model(260 aa) positions 1; 260 length is 246aa with 25 gaps, 5 stops, absolute frame= Plus1 at 2982304..2983041 in the same strand
CDS YP_001517265.1 hypothetical protein intersects 70.92%(33aa) with IS5_ISL2 e-value= 2.8e-20 complete sequence hit coverage= 100%, between model(260 aa) positions 1; 260 length is 246aa with 25 gaps, 5 stops, absolute frame= Plus1 at 2982304..2983041 in the same strand
gene no id fragment; similar to N-terminus of transposase intersects 87.39%(136aa) with IS5_ISL2 e-value= 4.5e-45 complete sequence hit coverage= 100%, between model(260 aa) positions 1; 260 length is 252aa with 12 gaps, 6 stops, absolute frame= Plus3 at 2982312..2983067 in the same strand
CDS YP_001517265.1 hypothetical protein intersects 89.36%(42aa) with IS5_ISL2 e-value= 4.5e-45 complete sequence hit coverage= 100%, between model(260 aa) positions 1; 260 length is 252aa with 12 gaps, 6 stops, absolute frame= Plus3 at 2982312..2983067 in the same strand
gene no id fragment; similar to N-terminus of transposase intersects 66.67%(104aa) with IS5_ISL2 e-value= 1.3e-41 fragment hit coverage= 40%, between model(260 aa) positions 26; 129 length is 104aa with 0 gaps, 0 stops, absolute frame= Plus1 at 2982364..2982675 in the same strand
gene no id fragment; similar to N-terminus of transposase intersects 11.75%(18aa) with IS5_ISL2 e-value= 5.1e-63 fragment hit coverage= 51.54%, between model(260 aa) positions 127; 260 length is 134aa with 0 gaps, 0 stops, absolute frame= Plus3 at 2982666..2983067 in the same strand

CDS YP_001517265.1 hypothetical protein intersects 89.36%(42aa) with IS5_ISL2 e-value= 5.1e-63 fragment hit coverage= 51.54%, between model(260 aa) positions 127; 260 length is 134aa with 0 gaps, 0 stops, absolute frame= Plus3 at 2982666..2983067 in the same strand
Class A

114.
CDS YP_001517497.1 hypothetical protein intersects 47.30%(128aa) with IS630 e-value= 1.1e-13 fragment hit coverage= 38.83%, between model(376 aa) positions 188; 333 length is 129aa with 20 gaps, 0 stops, absolute frame= Minus3 at 3216083..3216468 in the same strand
misc_feature no id Integrase core domain; Region: rve; cl01316 intersects 89.98%(128aa) with IS630 e-value= 1.1e-13 fragment hit coverage= 38.83%, between model(376 aa) positions 188; 333 length is 129aa with 20 gaps, 0 stops, absolute frame= Minus3 at 3216083..3216468 in the same strand
Class A

115.
CDS YP_001517531.1 hypothetical protein intersects 33.33%(13aa) with ISL3 e-value= 4e-07 complete sequence hit coverage= 100%, between model(451 aa) positions 1; 451 length is 386aa with 75 gaps, 2 stops, absolute frame= Plus2 at 3258380..3259537 in the same strand
CDS YP_001517532.1 transposase intersects 100%(289aa) with ISL3 e-value= 4e-07 complete sequence hit coverage= 100%, between model(451 aa) positions 1; 451 length is 386aa with 75 gaps, 2 stops, absolute frame= Plus2 at 3258380..3259537 in the same strand
misc_feature no id Transposase and inactivated derivatives [DNA replication, recombination, and repair]; Region: COG3464 intersects 100%(207aa) with ISL3 e-value= 4e-07 complete sequence hit coverage= 100%, between model(451 aa) positions 1; 451 length is 386aa with 75 gaps, 2 stops, absolute frame= Plus2 at 3258380..3259537 in the same strand
misc_feature no id Transposase; Region: DDE_Tnp_ISL3; pfam01610 intersects 100%(90aa) with ISL3 e-value= 4e-07 complete sequence hit coverage= 100%, between model(451 aa) positions 1; 451 length is 386aa with 75 gaps, 2 stops, absolute frame= Plus2 at 3258380..3259537 in the same strand
CDS YP_001517533.1 hypothetical protein intersects 25.56%(80aa) with ISL3 e-value= 4e-07 complete sequence hit coverage= 100%, between model(451 aa) positions 1; 451 length is 386aa with 75 gaps, 2 stops, absolute frame= Plus2 at 3258380..3259537 in the same strand
CDS YP_001517532.1 transposase intersects 32.53%(94aa) with ISL3 e-value= 1.4e-18 fragment hit coverage= 23.50%, between model(451 aa) positions 157; 262 length is 94aa with 12 gaps, 0 stops, absolute frame= Plus2 at 3258827..3259108 in the same strand
misc_feature no id Transposase and inactivated derivatives [DNA replication, recombination, and repair]; Region: COG3464 intersects 45.41%(94aa) with ISL3 e-value= 1.4e-18 fragment hit coverage= 23.50%, between model(451 aa) positions 157; 262 length is 94aa with 12 gaps, 0 stops, absolute frame= Plus2 at 3258827..3259108 in the same strand
misc_feature no id Transposase; Region: DDE_Tnp_ISL3; pfam01610 intersects 100%(90aa) with ISL3 e-value= 1.4e-18 fragment hit coverage= 23.50%, between model(451 aa) positions 157; 262 length is 94aa with 12 gaps, 0 stops, absolute frame= Plus2 at 3258827..3259108 in the same strand
Class A

116.
gene no id fragment; similar to N-terminus of transposase, mutator family intersects 82.08%(229aa) with IS256 e-value= 1.9e-79 fragment hit coverage= 56.59%, between model(410 aa) positions 1; 232 length is 229aa with 8 gaps, 0 stops, absolute frame= Plus3 at 3307083..3307769 in the same strand
gene no id fragment; similar to N-terminus of transposase, mutator family intersects 94.62%(264aa) with IS256 e-value= 4.1e-83 complete sequence hit coverage= 100%, between model(410 aa) positions 1; 410 length is 855aa with 11 gaps, 1 stops, absolute frame= Plus3 at 3307083..3309647 in the same strand
CDS YP_001517582.1 transposase mutator family protein intersects 100%(417aa) with IS256 e-value= 4.1e-83 complete sequence hit coverage= 100%, between model(410 aa) positions 1; 410 length is 855aa with 11 gaps, 1 stops, absolute frame= Plus3 at 3307083..3309647 in the same strand
misc_feature no id Transposase, Mutator family; Region: Transposase_mut; pfam00872 intersects 100%(362aa) with IS256 e-value= 4.1e-83 complete sequence hit coverage= 100%, between model(410 aa) positions 1; 410 length is 855aa with 11 gaps, 1 stops, absolute frame= Plus3 at 3307083..3309647 in the same strand

misc_feature no id MULE transposase domain; Region: MULE; pfam10551 intersects 100%(78aa) with IS256 e-value= 4.1e-83 complete sequence hit coverage= 100%, between model(410 aa) positions 1; 410 length is 855aa with 11 gaps, 1 stops, absolute frame= Plus3 at 3307083..3309647 in the same strand
gene no id fragment; similar to C-terminus of transposase, mutator family intersects 100%(175aa) with IS256 e-value= 4.1e-83 complete sequence hit coverage= 100%, between model(410 aa) positions 1; 410 length is 855aa with 11 gaps, 1 stops, absolute frame= Plus3 at 3307083..3309647 in the same strand
Class A

117.
gene no id fragment; similar to N-terminus of transposase, mutator family intersects 0.12%(0aa) with IS256 e-value= 3.7e-167 complete sequence hit coverage= 100%, between model(410 aa) positions 1; 410 length is 404aa with 11 gaps, 1 stops, absolute frame= Plus2 at 3307874..3309085 in the same strand
CDS YP_001517582.1 transposase mutator family protein intersects 96.40%(402aa) with IS256 e-value= 3.7e-167 complete sequence hit coverage= 100%, between model(410 aa) positions 1; 410 length is 404aa with 11 gaps, 1 stops, absolute frame= Plus2 at 3307874..3309085 in the same strand
misc_feature no id Transposase, Mutator family; Region: Transposase_mut; pfam00872 intersects 100%(362aa) with IS256 e-value= 3.7e-167 complete sequence hit coverage= 100%, between model(410 aa) positions 1; 410 length is 404aa with 11 gaps, 1 stops, absolute frame= Plus2 at 3307874..3309085 in the same strand
misc_feature no id MULE transposase domain; Region: MULE; pfam10551 intersects 100%(78aa) with IS256 e-value= 3.7e-167 complete sequence hit coverage= 100%, between model(410 aa) positions 1; 410 length is 404aa with 11 gaps, 1 stops, absolute frame= Plus2 at 3307874..3309085 in the same strand
Class A

118.
gene no id fragment; similar to C-terminus of transposase, mutator family intersects 100%(175aa) with IS256 e-value= 5.1e-77 fragment hit coverage= 43.90%, between model(410 aa) positions 231; 410 length is 177aa with 3 gaps, 1 stops, absolute frame= Plus3 at 3309117..3309647 in the same strand
Class A

119.
CDS YP_001517593.1 IS4 family transposase intersects 99.35%(308aa) with IS5_IS903 e-value= 1.7e-97 complete sequence hit coverage= 100%, between model(347 aa) positions 1; 347 length is 314aa with 37 gaps, 1 stops, absolute frame= Plus3 at 3315393..3316334 in the same strand
misc_feature no id Transposase DDE domain; Region: DDE_Tnp_1_5; pfam13737 intersects 100%(112aa) with IS5_IS903 e-value= 1.7e-97 complete sequence hit coverage= 100%, between model(347 aa) positions 1; 347 length is 314aa with 37 gaps, 1 stops, absolute frame= Plus3 at 3315393..3316334 in the same strand
misc_feature no id Transposase DDE domain; Region: DDE_Tnp_1; pfam01609 intersects 100%(186aa) with IS5_IS903 e-value= 1.7e-97 complete sequence hit coverage= 100%, between model(347 aa) positions 1; 347 length is 314aa with 37 gaps, 1 stops, absolute frame= Plus3 at 3315393..3316334 in the same strand
Class A

120.
CDS YP_001517685.1 hypothetical protein intersects 80%(128aa) with IS630 e-value= 6.5e-11 fragment hit coverage= 39.10%, between model(376 aa) positions 187; 333 length is 130aa with 21 gaps, 0 stops, absolute frame= Minus1 at 3406153..3406541 in the same strand
misc_feature no id Integrase core domain; Region: rve; cl01316 intersects 100%(128aa) with IS630 e-value= 6.5e-11 fragment hit coverage= 39.10%, between model(376 aa) positions 187; 333 length is 130aa with 21 gaps, 0 stops, absolute frame= Minus1 at 3406153..3406541 in the same strand
Class A

121.
CDS YP_001517686.1 hypothetical protein intersects 86.41%(135aa) with IS630 e-value= 8.7e-15 fragment hit coverage= 39.63%, between model(376 aa) positions 1; 149 length is 136aa with 13 gaps, 0 stops, absolute frame= Minus2 at 3406647..3407053 in the same strand
misc_feature no id Winged helix-turn helix; Region: HTH_29; pfam13551 intersects 100%(102aa) with IS630 e-value= 8.7e-15 fragment hit coverage= 39.63%, between model(376 aa) positions 1; 149 length is 136aa with 13 gaps, 0 stops, absolute frame= Minus2 at 3406647..3407053 in the same strand

misc_feature no id Helix-turn-helix domains; Region: HTH; cl00088 intersects 100%(72aa) with IS630 e-value= 8.7e-15 fragment hit coverage= 39.63%, between model(376 aa) positions 1; 149 length is 136aa with 13 gaps, 0 stops, absolute frame= Minus2 at 3406647..3407053 in the same strand
misc_feature no id Helix-turn-helix domains; Region: HTH; cl00088 intersects 100%(50aa) with IS630 e-value= 8.7e-15 fragment hit coverage= 39.63%, between model(376 aa) positions 1; 149 length is 136aa with 13 gaps, 0 stops, absolute frame= Minus2 at 3406647..3407053 in the same strand
CDS YP_001517686.1 hypothetical protein intersects 71.76%(112aa) with IS481 e-value= 3.5e-09 fragment hit coverage= 33.62%, between model(351 aa) positions 1; 118 length is 113aa with 6 gaps, 0 stops, absolute frame= Minus2 at 3406656..3406993 in the same strand
misc_feature no id Winged helix-turn helix; Region: HTH_29; pfam13551 intersects 100%(102aa) with IS481 e-value= 3.5e-09 fragment hit coverage= 33.62%, between model(351 aa) positions 1; 118 length is 113aa with 6 gaps, 0 stops, absolute frame= Minus2 at 3406656..3406993 in the same strand
misc_feature no id Helix-turn-helix domains; Region: HTH; cl00088 intersects 100%(72aa) with IS481 e-value= 3.5e-09 fragment hit coverage= 33.62%, between model(351 aa) positions 1; 118 length is 113aa with 6 gaps, 0 stops, absolute frame= Minus2 at 3406656..3406993 in the same strand
misc_feature no id Helix-turn-helix domains; Region: HTH; cl00088 intersects 100%(50aa) with IS481 e-value= 3.5e-09 fragment hit coverage= 33.62%, between model(351 aa) positions 1; 118 length is 113aa with 6 gaps, 0 stops, absolute frame= Minus2 at 3406656..3406993 in the same strand
Class B

122.
CDS YP_001517783.1 IS1 transposase intersects 96.52%(222aa) with IS1_ORF2 e-value= 3.2e-29 complete sequence hit coverage= 100%, between model(237 aa) positions 1; 237 length is 227aa with 14 gaps, 1 stops, absolute frame= Minus2 at 3510489..3511168 in the same strand
misc_feature no id IS1 transposase; Region: DDE_Tnp_IS1; cl00721 intersects 100%(120aa) with IS1_ORF2 e-value= 3.2e-29 complete sequence hit coverage= 100%, between model(237 aa) positions 1; 237 length is 227aa with 14 gaps, 1 stops, absolute frame= Minus2 at 3510489..3511168 in the same strand
misc_feature no id Transposase and inactivated derivatives [DNA replication, recombination, and repair]; Region: COG3677 intersects 100%(81aa) with IS1_ORF2 e-value= 3.2e-29 complete sequence hit coverage= 100%, between model(237 aa) positions 1; 237 length is 227aa with 14 gaps, 1 stops, absolute frame= Minus2 at 3510489..3511168 in the same strand
CDS YP_001517783.1 IS1 transposase intersects 58.12%(133aa) with IS1_ORF2 e-value= 8.3e-32 fragment hit coverage= 59.07%, between model(237 aa) positions 98; 237 length is 134aa with 6 gaps, 0 stops, absolute frame= Minus2 at 3510489..3510889 in the same strand
misc_feature no id IS1 transposase; Region: DDE_Tnp_IS1; cl00721 intersects 100%(120aa) with IS1_ORF2 e-value= 8.3e-32 fragment hit coverage= 59.07%, between model(237 aa) positions 98; 237 length is 134aa with 6 gaps, 0 stops, absolute frame= Minus2 at 3510489..3510889 in the same strand
CDS YP_001517783.1 IS1 transposase intersects 95.65%(220aa) with IS1_ORF1 e-value= 1.9e-41 complete sequence hit coverage= 100%, between model(239 aa) positions 1; 239 length is 225aa with 22 gaps, 1 stops, absolute frame= Minus2 at 3510495..3511168 in the same strand
misc_feature no id IS1 transposase; Region: DDE_Tnp_IS1; cl00721 intersects 100%(120aa) with IS1_ORF1 e-value= 1.9e-41 complete sequence hit coverage= 100%, between model(239 aa) positions 1; 239 length is 225aa with 22 gaps, 1 stops, absolute frame= Minus2 at 3510495..3511168 in the same strand
misc_feature no id Transposase and inactivated derivatives [DNA replication, recombination, and repair]; Region: COG3677 intersects 100%(81aa) with IS1_ORF1 e-value= 1.9e-41 complete sequence hit coverage= 100%, between model(239 aa) positions 1; 239 length is 225aa with 22 gaps, 1 stops, absolute frame= Minus2 at 3510495..3511168 in the same strand
Class A

123.
CDS YP_001517848.1 IS5 family transposase intersects 14.33%(80aa) with IS5_IS5 e-value= 2.6e-06 fragment hit coverage= 19.91%, between model(422 aa) positions 23; 106 length is 81aa with 3 gaps, 0 stops, absolute frame= Minus3 at 3585497..3585738 in the same strand
misc_feature no id Transposase domain (DUF772); Region: DUF772; pfam05598 intersects 74.03%(57aa) with IS5_IS5 e-value= 2.6e-06 fragment hit coverage= 19.91%, between model(422 aa) positions 23; 106 length is 81aa with 3 gaps, 0 stops, absolute frame= Minus3 at 3585497..3585738 in the same strand
Class A

124.
CDS YP_001517935.1 transposase intersects 85.96%(375aa) with IS4 e-value= 9.9e-07 complete sequence hit coverage= 100%, between model(486 aa) positions 1; 486 length is 376aa with 117 gaps, 0 stops, absolute frame= Minus2 at 3687618..3688744 in the same strand
misc_feature no id DDE superfamily endonuclease; Region: DDE_5; cl02413 intersects 93.48%(348aa) with IS4 e-value= 9.9e-07 complete sequence hit coverage= 100%, between model(486 aa) positions 1; 486 length is 376aa with 117 gaps, 0 stops, absolute frame= Minus2 at 3687618..3688744 in the same strand
CDS YP_001517935.1 transposase intersects 53.01%(231aa) with IS4 e-value= 4.7e-14 fragment hit coverage= 51.23%, between model(486 aa) positions 1; 249 length is 232aa with 27 gaps, 0 stops, absolute frame= Minus2 at 3688050..3688744 in the same strand
misc_feature no id DDE superfamily endonuclease; Region: DDE_5; cl02413 intersects 62.11%(231aa) with IS4 e-value= 4.7e-14 fragment hit coverage= 51.23%, between model(486 aa) positions 1; 249 length is 232aa with 27 gaps, 0 stops, absolute frame= Minus2 at 3688050..3688744 in the same strand
Class A

125.
CDS YP_001518001.1 transposase mutator family protein intersects 96.40%(402aa) with IS256 e-value= 3.7e-167 complete sequence hit coverage= 100%, between model(410 aa) positions 1; 410 length is 404aa with 11 gaps, 1 stops, absolute frame= Plus2 at 3753371..3754582 in the same strand
misc_feature no id Transposase, Mutator family; Region: Transposase_mut; pfam00872 intersects 100%(362aa) with IS256 e-value= 3.7e-167 complete sequence hit coverage= 100%, between model(410 aa) positions 1; 410 length is 404aa with 11 gaps, 1 stops, absolute frame= Plus2 at 3753371..3754582 in the same strand
misc_feature no id MULE transposase domain; Region: MULE; pfam10551 intersects 100%(78aa) with IS256 e-value= 3.7e-167 complete sequence hit coverage= 100%, between model(410 aa) positions 1; 410 length is 404aa with 11 gaps, 1 stops, absolute frame= Plus2 at 3753371..3754582 in the same strand
Class A

126.
CDS YP_001518006.1 hypothetical protein intersects 47.43%(129aa) with IS630 e-value= 1.1e-13 fragment hit coverage= 38.83%, between model(376 aa) positions 188; 333 length is 129aa with 20 gaps, 0 stops, absolute frame= Plus3 at 3758064..3758450 in the same strand
misc_feature no id Integrase core domain; Region: rve; cl01316 intersects 90.21%(129aa) with IS630 e-value= 1.1e-13 fragment hit coverage= 38.83%, between model(376 aa) positions 188; 333 length is 129aa with 20 gaps, 0 stops, absolute frame= Plus3 at 3758064..3758450 in the same strand
Class A

127.
CDS YP_001518007.1 transposase intersects 85.96%(375aa) with IS4 e-value= 9.9e-07 complete sequence hit coverage= 100%, between model(486 aa) positions 1; 486 length is 376aa with 117 gaps, 0 stops, absolute frame= Minus2 at 3758637..3759763 in the same strand
misc_feature no id DDE superfamily endonuclease; Region: DDE_5; cl02413 intersects 93.48%(348aa) with IS4 e-value= 9.9e-07 complete sequence hit coverage= 100%, between model(486 aa) positions 1; 486 length is 376aa with 117 gaps, 0 stops, absolute frame= Minus2 at 3758637..3759763 in the same strand
CDS YP_001518007.1 transposase intersects 53.01%(231aa) with IS4 e-value= 4.7e-14 fragment hit coverage= 51.23%, between model(486 aa) positions 1; 249 length is 232aa with 27 gaps, 0 stops, absolute frame= Minus2 at 3759069..3759763 in the same strand
misc_feature no id DDE superfamily endonuclease; Region: DDE_5; cl02413 intersects 62.11%(231aa) with IS4 e-value= 4.7e-14 fragment hit coverage= 51.23%, between model(486 aa) positions 1; 249 length is 232aa with 27 gaps, 0 stops, absolute frame= Minus2 at 3759069..3759763 in the same strand
Class A

128.
CDS YP_001518130.1 hypothetical protein intersects 47.30%(128aa) with IS630 e-value= 1.1e-13 fragment hit coverage= 38.83%, between model(376 aa) positions 188; 333 length is 129aa with 20 gaps, 0 stops, absolute frame= Minus3 at 3873578..3873963 in the same strand

misc_feature no id Integrase core domain; Region: rve; cl01316 intersects 89.98%(128aa) with IS630 e-value= 1.1e-13 fragment hit coverage= 38.83%, between model(376 aa) positions 188; 333 length is 129aa with 20 gaps, 0 stops, absolute frame= Minus3 at 3873578..3873963 in the same strand
Class A

129.
CDS YP_001518162.1 IS4 family transposase intersects 99.35%(308aa) with IS5_IS903 e-value= 2.2e-96 complete sequence hit coverage= 100%, between model(347 aa) positions 1; 347 length is 314aa with 37 gaps, 1 stops, absolute frame= Plus2 at 3911570..3912511 in the same strand
misc_feature no id Transposase DDE domain; Region: DDE_Tnp_1_5; pfam13737 intersects 100%(112aa) with IS5_IS903 e-value= 2.2e-96 complete sequence hit coverage= 100%, between model(347 aa) positions 1; 347 length is 314aa with 37 gaps, 1 stops, absolute frame= Plus2 at 3911570..3912511 in the same strand
misc_feature no id Transposase DDE domain; Region: DDE_Tnp_1; pfam01609 intersects 100%(186aa) with IS5_IS903 e-value= 2.2e-96 complete sequence hit coverage= 100%, between model(347 aa) positions 1; 347 length is 314aa with 37 gaps, 1 stops, absolute frame= Plus2 at 3911570..3912511 in the same strand
Class A

130.
CDS YP_001518164.1 integrase catalytic subunit intersects 99.30%(283aa) with IS3_IS51_ORF2 e-value= 3.7e-25 complete sequence hit coverage= 100%, between model(317 aa) positions 1; 317 length is 290aa with 30 gaps, 0 stops, absolute frame= Minus2 at 3913140..3914008 in the same strand
misc_feature no id putative transposase OrfB; Reviewed; Region: PHA02517 intersects 100%(254aa) with IS3_IS51_ORF2 e-value= 3.7e-25 complete sequence hit coverage= 100%, between model(317 aa) positions 1; 317 length is 290aa with 30 gaps, 0 stops, absolute frame= Minus2 at 3913140..3914008 in the same strand
misc_feature no id Integrase core domain; Region: rve_3; cl15866 intersects 100%(68aa) with IS3_IS51_ORF2 e-value= 3.7e-25 complete sequence hit coverage= 100%, between model(317 aa) positions 1; 317 length is 290aa with 30 gaps, 0 stops, absolute frame= Minus2 at 3913140..3914008 in the same strand
misc_feature no id Integrase core domain; Region: rve; cl01316 intersects 100%(110aa) with IS3_IS51_ORF2 e-value= 3.7e-25 complete sequence hit coverage= 100%, between model(317 aa) positions 1; 317 length is 290aa with 30 gaps, 0 stops, absolute frame= Minus2 at 3913140..3914008 in the same strand
misc_feature no id HTH-like domain; Region: HTH_21; pfam13276 intersects 100%(42aa) with IS3_IS51_ORF2 e-value= 3.7e-25 complete sequence hit coverage= 100%, between model(317 aa) positions 1; 317 length is 290aa with 30 gaps, 0 stops, absolute frame= Minus2 at 3913140..3914008 in the same strand
CDS YP_001518165.1 hypothetical protein intersects 0.49%(2aa) with IS3_IS51_ORF2 e-value= 3.7e-25 complete sequence hit coverage= 100%, between model(317 aa) positions 1; 317 length is 290aa with 30 gaps, 0 stops, absolute frame= Minus2 at 3913140..3914008 in the same strand
CDS YP_001518164.1 integrase catalytic subunit intersects 99.30%(283aa) with IS481 e-value= 2.7e-07 complete sequence hit coverage= 100%, between model(351 aa) positions 1; 351 length is 296aa with 57 gaps, 0 stops, absolute frame= Minus2 at 3913140..3914026 in the same strand
misc_feature no id putative transposase OrfB; Reviewed; Region: PHA02517 intersects 100%(254aa) with IS481 e-value= 2.7e-07 complete sequence hit coverage= 100%, between model(351 aa) positions 1; 351 length is 296aa with 57 gaps, 0 stops, absolute frame= Minus2 at 3913140..3914026 in the same strand
misc_feature no id Integrase core domain; Region: rve_3; cl15866 intersects 100%(68aa) with IS481 e-value= 2.7e-07 complete sequence hit coverage= 100%, between model(351 aa) positions 1; 351 length is 296aa with 57 gaps, 0 stops, absolute frame= Minus2 at 3913140..3914026 in the same strand
misc_feature no id Integrase core domain; Region: rve; cl01316 intersects 100%(110aa) with IS481 e-value= 2.7e-07 complete sequence hit coverage= 100%, between model(351 aa) positions 1; 351 length is 296aa with 57 gaps, 0 stops, absolute frame= Minus2 at 3913140..3914026 in the same strand
misc_feature no id HTH-like domain; Region: HTH_21; pfam13276 intersects 100%(42aa) with IS481 e-value= 2.7e-07 complete sequence hit coverage= 100%, between model(351 aa) positions 1; 351 length is 296aa with 57 gaps, 0 stops, absolute frame= Minus2 at 3913140..3914026 in the same strand
CDS YP_001518165.1 hypothetical protein intersects 1.97%(8aa) with IS481 e-value= 2.7e-07 complete sequence hit coverage= 100%, between model(351 aa) positions 1; 351 length is 296aa with 57 gaps, 0 stops, absolute frame= Minus2 at 3913140..3914026 in the same strand
misc_feature no id Transposase Tn5 dimerisation domain; Region: Dimer_Tnp_Tn5; pfam02281 intersects 4.29%(3aa) with IS481 e-value= 2.7e-07 complete sequence hit coverage= 100%, between model(351 aa) positions 1; 351 length is 296aa with 57 gaps, 0 stops, absolute frame= Minus2 at 3913140..3914026 in the same strand

CDS YP_001518164.1 integrase catalytic subunit intersects 98.25%(280aa) with IS3_IS51_ORF1 e-value= 3.9e-53 complete sequence hit coverage= 100%, between model(307 aa) positions 1; 307 length is 283aa with 25 gaps, 0 stops, absolute frame= Minus2 at 3913149..3913996 in the same strand
misc_feature no id putative transposase OrfB; Reviewed; Region: PHA02517 intersects 100%(254aa) with IS3_IS51_ORF1 e-value= 3.9e-53 complete sequence hit coverage= 100%, between model(307 aa) positions 1; 307 length is 283aa with 25 gaps, 0 stops, absolute frame= Minus2 at 3913149..3913996 in the same strand
misc_feature no id Integrase core domain; Region: rve_3; cl15866 intersects 100%(68aa) with IS3_IS51_ORF1 e-value= 3.9e-53 complete sequence hit coverage= 100%, between model(307 aa) positions 1; 307 length is 283aa with 25 gaps, 0 stops, absolute frame= Minus2 at 3913149..3913996 in the same strand
misc_feature no id Integrase core domain; Region: rve; cl01316 intersects 100%(110aa) with IS3_IS51_ORF1 e-value= 3.9e-53 complete sequence hit coverage= 100%, between model(307 aa) positions 1; 307 length is 283aa with 25 gaps, 0 stops, absolute frame= Minus2 at 3913149..3913996 in the same strand
misc_feature no id HTH-like domain; Region: HTH_21; pfam13276 intersects 100%(42aa) with IS3_IS51_ORF1 e-value= 3.9e-53 complete sequence hit coverage= 100%, between model(307 aa) positions 1; 307 length is 283aa with 25 gaps, 0 stops, absolute frame= Minus2 at 3913149..3913996 in the same strand
CDS YP_001518164.1 integrase catalytic subunit intersects 98.25%(280aa) with IS3_IS407_ORF2 e-value= 1.2e-11 complete sequence hit coverage= 100%, between model(344 aa) positions 1; 344 length is 333aa with 22 gaps, 1 stops, absolute frame= Minus2 at 3913149..3914146 in the same strand
misc_feature no id putative transposase OrfB; Reviewed; Region: PHA02517 intersects 100%(254aa) with IS3_IS407_ORF2 e-value= 1.2e-11 complete sequence hit coverage= 100%, between model(344 aa) positions 1; 344 length is 333aa with 22 gaps, 1 stops, absolute frame= Minus2 at 3913149..3914146 in the same strand
misc_feature no id Integrase core domain; Region: rve_3; cl15866 intersects 100%(68aa) with IS3_IS407_ORF2 e-value= 1.2e-11 complete sequence hit coverage= 100%, between model(344 aa) positions 1; 344 length is 333aa with 22 gaps, 1 stops, absolute frame= Minus2 at 3913149..3914146 in the same strand
misc_feature no id Integrase core domain; Region: rve; cl01316 intersects 100%(110aa) with IS3_IS407_ORF2 e-value= 1.2e-11 complete sequence hit coverage= 100%, between model(344 aa) positions 1; 344 length is 333aa with 22 gaps, 1 stops, absolute frame= Minus2 at 3913149..3914146 in the same strand
misc_feature no id HTH-like domain; Region: HTH_21; pfam13276 intersects 100%(42aa) with IS3_IS407_ORF2 e-value= 1.2e-11 complete sequence hit coverage= 100%, between model(344 aa) positions 1; 344 length is 333aa with 22 gaps, 1 stops, absolute frame= Minus2 at 3913149..3914146 in the same strand
CDS YP_001518165.1 hypothetical protein intersects 11.82%(48aa) with IS3_IS407_ORF2 e-value= 1.2e-11 complete sequence hit coverage= 100%, between model(344 aa) positions 1; 344 length is 333aa with 22 gaps, 1 stops, absolute frame= Minus2 at 3913149..3914146 in the same strand
misc_feature no id Transposase Tn5 dimerisation domain; Region: Dimer_Tnp_Tn5; pfam02281 intersects 61.43%(43aa) with IS3_IS407_ORF2 e-value= 1.2e-11 complete sequence hit coverage= 100%, between model(344 aa) positions 1; 344 length is 333aa with 22 gaps, 1 stops, absolute frame= Minus2 at 3913149..3914146 in the same strand
CDS YP_001518164.1 integrase catalytic subunit intersects 96.49%(275aa) with IS3_IS2_ORF2 e-value= 3.7e-08 complete sequence hit coverage= 100%, between model(302 aa) positions 1; 302 length is 281aa with 34 gaps, 0 stops, absolute frame= Minus2 at 3913164..3914005 in the same strand
misc_feature no id putative transposase OrfB; Reviewed; Region: PHA02517 intersects 100%(254aa) with IS3_IS2_ORF2 e-value= 3.7e-08 complete sequence hit coverage= 100%, between model(302 aa) positions 1; 302 length is 281aa with 34 gaps, 0 stops, absolute frame= Minus2 at 3913164..3914005 in the same strand
misc_feature no id Integrase core domain; Region: rve_3; cl15866 intersects 100%(68aa) with IS3_IS2_ORF2 e-value= 3.7e-08 complete sequence hit coverage= 100%, between model(302 aa) positions 1; 302 length is 281aa with 34 gaps, 0 stops, absolute frame= Minus2 at 3913164..3914005 in the same strand
misc_feature no id Integrase core domain; Region: rve; cl01316 intersects 100%(110aa) with IS3_IS2_ORF2 e-value= 3.7e-08 complete sequence hit coverage= 100%, between model(302 aa) positions 1; 302 length is 281aa with 34 gaps, 0 stops, absolute frame= Minus2 at 3913164..3914005 in the same strand
misc_feature no id HTH-like domain; Region: HTH_21; pfam13276 intersects 100%(42aa) with IS3_IS2_ORF2 e-value= 3.7e-08 complete sequence hit coverage= 100%, between model(302 aa) positions 1; 302 length is 281aa with 34 gaps, 0 stops, absolute frame= Minus2 at 3913164..3914005 in the same strand
CDS YP_001518165.1 hypothetical protein intersects 0.25%(1aa) with IS3_IS2_ORF2 e-value= 3.7e-08 complete sequence hit coverage= 100%, between model(302 aa) positions 1; 302 length is 281aa with 34 gaps, 0 stops, absolute frame= Minus2 at 3913164..3914005 in the same strand

CDS YP_001518164.1 integrase catalytic subunit intersects 79.88%(227aa) with IS3_IS51_ORF2 e-value= 1.9e-38 fragment hit coverage= 74.76%, between model(317 aa) positions 73; 309 length is 228aa with 11 gaps, 0 stops, absolute frame= Minus2 at 3913164..3913846 in the same strand
misc_feature no id putative transposase OrfB; Reviewed; Region: PHA02517 intersects 89.24%(226aa) with IS3_IS51_ORF2 e-value= 1.9e-38 fragment hit coverage= 74.76%, between model(317 aa) positions 73; 309 length is 228aa with 11 gaps, 0 stops, absolute frame= Minus2 at 3913164..3913846 in the same strand
misc_feature no id Integrase core domain; Region: rve_3; cl15866 intersects 100%(68aa) with IS3_IS51_ORF2 e-value= 1.9e-38 fragment hit coverage= 74.76%, between model(317 aa) positions 73; 309 length is 228aa with 11 gaps, 0 stops, absolute frame= Minus2 at 3913164..3913846 in the same strand
misc_feature no id Integrase core domain; Region: rve; cl01316 intersects 100%(110aa) with IS3_IS51_ORF2 e-value= 1.9e-38 fragment hit coverage= 74.76%, between model(317 aa) positions 73; 309 length is 228aa with 11 gaps, 0 stops, absolute frame= Minus2 at 3913164..3913846 in the same strand
misc_feature no id HTH-like domain; Region: HTH_21; pfam13276 intersects 99.21%(41aa) with IS3_IS51_ORF2 e-value= 1.9e-38 fragment hit coverage= 74.76%, between model(317 aa) positions 73; 309 length is 228aa with 11 gaps, 0 stops, absolute frame= Minus2 at 3913164..3913846 in the same strand
CDS YP_001518164.1 integrase catalytic subunit intersects 95.32%(271aa) with IS3_IS3_ORF2 e-value= 1.2e-64 complete sequence hit coverage= 100%, between model(288 aa) positions 1; 288 length is 272aa with 19 gaps, 0 stops, absolute frame= Minus2 at 3913167..3913981 in the same strand
misc_feature no id putative transposase OrfB; Reviewed; Region: PHA02517 intersects 100%(254aa) with IS3_IS3_ORF2 e-value= 1.2e-64 complete sequence hit coverage= 100%, between model(288 aa) positions 1; 288 length is 272aa with 19 gaps, 0 stops, absolute frame= Minus2 at 3913167..3913981 in the same strand
misc_feature no id Integrase core domain; Region: rve_3; cl15866 intersects 100%(68aa) with IS3_IS3_ORF2 e-value= 1.2e-64 complete sequence hit coverage= 100%, between model(288 aa) positions 1; 288 length is 272aa with 19 gaps, 0 stops, absolute frame= Minus2 at 3913167..3913981 in the same strand
misc_feature no id Integrase core domain; Region: rve; cl01316 intersects 100%(110aa) with IS3_IS3_ORF2 e-value= 1.2e-64 complete sequence hit coverage= 100%, between model(288 aa) positions 1; 288 length is 272aa with 19 gaps, 0 stops, absolute frame= Minus2 at 3913167..3913981 in the same strand
misc_feature no id HTH-like domain; Region: HTH_21; pfam13276 intersects 100%(42aa) with IS3_IS3_ORF2 e-value= 1.2e-64 complete sequence hit coverage= 100%, between model(288 aa) positions 1; 288 length is 272aa with 19 gaps, 0 stops, absolute frame= Minus2 at 3913167..3913981 in the same strand
CDS YP_001518164.1 integrase catalytic subunit intersects 95.44%(272aa) with IS3_IS150_ORF2 e-value= 5e-28 complete sequence hit coverage= 100%, between model(389 aa) positions 1; 389 length is 353aa with 40 gaps, 2 stops, absolute frame= Minus2 at 3913173..3914230 in the same strand
misc_feature no id putative transposase OrfB; Reviewed; Region: PHA02517 intersects 99.21%(252aa) with IS3_IS150_ORF2 e-value= 5e-28 complete sequence hit coverage= 100%, between model(389 aa) positions 1; 389 length is 353aa with 40 gaps, 2 stops, absolute frame= Minus2 at 3913173..3914230 in the same strand
misc_feature no id Integrase core domain; Region: rve_3; cl15866 intersects 100%(68aa) with IS3_IS150_ORF2 e-value= 5e-28 complete sequence hit coverage= 100%, between model(389 aa) positions 1; 389 length is 353aa with 40 gaps, 2 stops, absolute frame= Minus2 at 3913173..3914230 in the same strand
misc_feature no id Integrase core domain; Region: rve; cl01316 intersects 100%(110aa) with IS3_IS150_ORF2 e-value= 5e-28 complete sequence hit coverage= 100%, between model(389 aa) positions 1; 389 length is 353aa with 40 gaps, 2 stops, absolute frame= Minus2 at 3913173..3914230 in the same strand
misc_feature no id HTH-like domain; Region: HTH_21; pfam13276 intersects 100%(42aa) with IS3_IS150_ORF2 e-value= 5e-28 complete sequence hit coverage= 100%, between model(389 aa) positions 1; 389 length is 353aa with 40 gaps, 2 stops, absolute frame= Minus2 at 3913173..3914230 in the same strand
CDS YP_001518165.1 hypothetical protein intersects 18.72%(76aa) with IS3_IS150_ORF2 e-value= 5e-28 complete sequence hit coverage= 100%, between model(389 aa) positions 1; 389 length is 353aa with 40 gaps, 2 stops, absolute frame= Minus2 at 3913173..3914230 in the same strand
misc_feature no id Transposase Tn5 dimerisation domain; Region: Dimer_Tnp_Tn5; pfam02281 intersects 100%(70aa) with IS3_IS150_ORF2 e-value= 5e-28 complete sequence hit coverage= 100%, between model(389 aa) positions 1; 389 length is 353aa with 40 gaps, 2 stops, absolute frame= Minus2 at 3913173..3914230 in the same strand
CDS YP_001518164.1 integrase catalytic subunit intersects 94.27%(268aa) with IS3_IS150_ORF2 e-value= 2.2e-46 fragment hit coverage= 74.29%, between model(389 aa) positions 101; 389 length is 269aa with 23 gaps, 0 stops, absolute frame= Minus2 at 3913173..3913978 in the same strand

misc_feature no id putative transposase OrfB; Reviewed; Region: PHA02517 intersects 99.21%(252aa) with IS3_IS150_ORF2 e-value= 2.2e-46 fragment hit coverage= 74.29%, between model(389 aa) positions 101; 389 length is 269aa with 23 gaps, 0 stops, absolute frame= Minus2 at 3913173..3913978 in the same strand
misc_feature no id Integrase core domain; Region: rve_3; cl15866 intersects 100%(68aa) with IS3_IS150_ORF2 e-value= 2.2e-46 fragment hit coverage= 74.29%, between model(389 aa) positions 101; 389 length is 269aa with 23 gaps, 0 stops, absolute frame= Minus2 at 3913173..3913978 in the same strand
misc_feature no id Integrase core domain; Region: rve; cl01316 intersects 100%(110aa) with IS3_IS150_ORF2 e-value= 2.2e-46 fragment hit coverage= 74.29%, between model(389 aa) positions 101; 389 length is 269aa with 23 gaps, 0 stops, absolute frame= Minus2 at 3913173..3913978 in the same strand
misc_feature no id HTH-like domain; Region: HTH_21; pfam13276 intersects 100%(42aa) with IS3_IS150_ORF2 e-value= 2.2e-46 fragment hit coverage= 74.29%, between model(389 aa) positions 101; 389 length is 269aa with 23 gaps, 0 stops, absolute frame= Minus2 at 3913173..3913978 in the same strand
CDS YP_001518164.1 integrase catalytic subunit intersects 95.09%(271aa) with IS3_IS150_ORF1 e-value= 2.2e-46 complete sequence hit coverage= 100%, between model(306 aa) positions 1; 306 length is 292aa with 18 gaps, 0 stops, absolute frame= Minus2 at 3913176..3914050 in the same strand
misc_feature no id putative transposase OrfB; Reviewed; Region: PHA02517 intersects 98.82%(251aa) with IS3_IS150_ORF1 e-value= 2.2e-46 complete sequence hit coverage= 100%, between model(306 aa) positions 1; 306 length is 292aa with 18 gaps, 0 stops, absolute frame= Minus2 at 3913176..3914050 in the same strand
misc_feature no id Integrase core domain; Region: rve_3; cl15866 intersects 100%(68aa) with IS3_IS150_ORF1 e-value= 2.2e-46 complete sequence hit coverage= 100%, between model(306 aa) positions 1; 306 length is 292aa with 18 gaps, 0 stops, absolute frame= Minus2 at 3913176..3914050 in the same strand
misc_feature no id Integrase core domain; Region: rve; cl01316 intersects 100%(110aa) with IS3_IS150_ORF1 e-value= 2.2e-46 complete sequence hit coverage= 100%, between model(306 aa) positions 1; 306 length is 292aa with 18 gaps, 0 stops, absolute frame= Minus2 at 3913176..3914050 in the same strand
misc_feature no id HTH-like domain; Region: HTH_21; pfam13276 intersects 100%(42aa) with IS3_IS150_ORF1 e-value= 2.2e-46 complete sequence hit coverage= 100%, between model(306 aa) positions 1; 306 length is 292aa with 18 gaps, 0 stops, absolute frame= Minus2 at 3913176..3914050 in the same strand
CDS YP_001518165.1 hypothetical protein intersects 3.94%(16aa) with IS3_IS150_ORF1 e-value= 2.2e-46 complete sequence hit coverage= 100%, between model(306 aa) positions 1; 306 length is 292aa with 18 gaps, 0 stops, absolute frame= Minus2 at 3913176..3914050 in the same strand
misc_feature no id Transposase Tn5 dimerisation domain; Region: Dimer_Tnp_Tn5; pfam02281 intersects 15.71%(11aa) with IS3_IS150_ORF1 e-value= 2.2e-46 complete sequence hit coverage= 100%, between model(306 aa) positions 1; 306 length is 292aa with 18 gaps, 0 stops, absolute frame= Minus2 at 3913176..3914050 in the same strand
CDS YP_001518164.1 integrase catalytic subunit intersects 92.16%(262aa) with IS3_IS150_ORF1 e-value= 1.4e-49 fragment hit coverage= 89.87%, between model(306 aa) positions 32; 306 length is 263aa with 14 gaps, 0 stops, absolute frame= Minus2 at 3913176..3913963 in the same strand
misc_feature no id putative transposase OrfB; Reviewed; Region: PHA02517 intersects 98.82%(251aa) with IS3_IS150_ORF1 e-value= 1.4e-49 fragment hit coverage= 89.87%, between model(306 aa) positions 32; 306 length is 263aa with 14 gaps, 0 stops, absolute frame= Minus2 at 3913176..3913963 in the same strand
misc_feature no id Integrase core domain; Region: rve_3; cl15866 intersects 100%(68aa) with IS3_IS150_ORF1 e-value= 1.4e-49 fragment hit coverage= 89.87%, between model(306 aa) positions 32; 306 length is 263aa with 14 gaps, 0 stops, absolute frame= Minus2 at 3913176..3913963 in the same strand
misc_feature no id Integrase core domain; Region: rve; cl01316 intersects 100%(110aa) with IS3_IS150_ORF1 e-value= 1.4e-49 fragment hit coverage= 89.87%, between model(306 aa) positions 32; 306 length is 263aa with 14 gaps, 0 stops, absolute frame= Minus2 at 3913176..3913963 in the same strand
misc_feature no id HTH-like domain; Region: HTH_21; pfam13276 intersects 100%(42aa) with IS3_IS150_ORF1 e-value= 1.4e-49 fragment hit coverage= 89.87%, between model(306 aa) positions 32; 306 length is 263aa with 14 gaps, 0 stops, absolute frame= Minus2 at 3913176..3913963 in the same strand
CDS YP_001518164.1 integrase catalytic subunit intersects 94.74%(270aa) with IS3_IS3_ORF1 e-value= 2e-54 complete sequence hit coverage= 100%, between model(286 aa) positions 1; 286 length is 272aa with 15 gaps, 0 stops, absolute frame= Minus2 at 3913179..3913993 in the same strand
misc_feature no id putative transposase OrfB; Reviewed; Region: PHA02517 intersects 98.43%(250aa) with IS3_IS3_ORF1 e-value= 2e-54 complete sequence hit coverage= 100%, between model(286 aa) positions 1; 286 length is 272aa with 15 gaps, 0 stops, absolute frame= Minus2 at 3913179..3913993 in the same strand

misc_feature	no id	Integrase core domain; Region: rve_3; cl15866 intersects 100%(68aa) with IS3_IS3_ORF1	e-value= 2e-54 complete sequence hit coverage= 100%, between model(286 aa) positions 1; 286 length is 272aa with 15 gaps, 0 stops, absolute frame= Minus2 at 3913179..3913993 in the same strand
misc_feature	no id	Integrase core domain; Region: rve; cl01316 intersects 100%(110aa) with IS3_IS3_ORF1	e-value= 2e-54 complete sequence hit coverage= 100%, between model(286 aa) positions 1; 286 length is 272aa with 15 gaps, 0 stops, absolute frame= Minus2 at 3913179..3913993 in the same strand
misc_feature	no id	HTH-like domain; Region: HTH_21; pfam13276 intersects 100%(42aa) with IS3_IS3_ORF1	e-value= 2e-54 complete sequence hit coverage= 100%, between model(286 aa) positions 1; 286 length is 272aa with 15 gaps, 0 stops, absolute frame= Minus2 at 3913179..3913993 in the same strand
CDS	YP_001518164.1	integrase catalytic subunit intersects 18.48%(52aa) with IS3_IS2_ORF2	e-value= 9.1e-09 fragment hit coverage= 18.87%, between model(302 aa) positions 48; 104 length is 53aa with 5 gaps, 0 stops, absolute frame= Minus2 at 3913755..3913912 in the same strand
misc_feature	no id	putative transposase OrfB; Reviewed; Region: PHA02517 intersects 20.73%(52aa) with IS3_IS2_ORF2	e-value= 9.1e-09 fragment hit coverage= 18.87%, between model(302 aa) positions 48; 104 length is 53aa with 5 gaps, 0 stops, absolute frame= Minus2 at 3913755..3913912 in the same strand
misc_feature	no id	HTH-like domain; Region: HTH_21; pfam13276 intersects 73.81%(31aa) with IS3_IS2_ORF2	e-value= 9.1e-09 fragment hit coverage= 18.87%, between model(302 aa) positions 48; 104 length is 53aa with 5 gaps, 0 stops, absolute frame= Minus2 at 3913755..3913912 in the same strand
CDS	YP_001518164.1	integrase catalytic subunit intersects 23.39%(66aa) with IS3_IS407_ORF2	e-value= 2e-15 fragment hit coverage= 19.48%, between model(344 aa) positions 80; 146 length is 67aa with 0 gaps, 0 stops, absolute frame= Minus2 at 3913764..3913963 in the same strand
misc_feature	no id	putative transposase OrfB; Reviewed; Region: PHA02517 intersects 21.65%(55aa) with IS3_IS407_ORF2	e-value= 2e-15 fragment hit coverage= 19.48%, between model(344 aa) positions 80; 146 length is 67aa with 0 gaps, 0 stops, absolute frame= Minus2 at 3913764..3913963 in the same strand
misc_feature	no id	HTH-like domain; Region: HTH_21; pfam13276 intersects 66.67%(28aa) with IS3_IS407_ORF2	e-value= 2e-15 fragment hit coverage= 19.48%, between model(344 aa) positions 80; 146 length is 67aa with 0 gaps, 0 stops, absolute frame= Minus2 at 3913764..3913963 in the same strand
Class A

131.
CDS	YP_001518225.1	hypothetical protein intersects 81.65%(129aa) with IS630	e-value= 1.1e-13 fragment hit coverage= 38.83%, between model(376 aa) positions 188; 333 length is 129aa with 20 gaps, 0 stops, absolute frame= Plus1 at 3974686..3975072 in the same strand
misc_feature	no id	Integrase core domain; Region: rve; cl01316 intersects 90.21%(129aa) with IS630	e-value= 1.1e-13 fragment hit coverage= 38.83%, between model(376 aa) positions 188; 333 length is 129aa with 20 gaps, 0 stops, absolute frame= Plus1 at 3974686..3975072 in the same strand
Class A

132.
CDS	YP_001518226.1	transposase intersects 53.09%(232aa) with IS4	e-value= 4.7e-14 fragment hit coverage= 51.23%, between model(486 aa) positions 1; 249 length is 232aa with 27 gaps, 0 stops, absolute frame= Plus1 at 3975325..3976020 in the same strand
misc_feature	no id	DDE superfamily endonuclease; Region: DDE_5; cl02413 intersects 62.20%(232aa) with IS4	e-value= 4.7e-14 fragment hit coverage= 51.23%, between model(486 aa) positions 1; 249 length is 232aa with 27 gaps, 0 stops, absolute frame= Plus1 at 3975325..3976020 in the same strand
CDS	YP_001518226.1	transposase intersects 86.04%(376aa) with IS4	e-value= 9.9e-07 complete sequence hit coverage= 100%, between model(486 aa) positions 1; 486 length is 376aa with 117 gaps, 0 stops, absolute frame= Plus1 at 3975325..3976452 in the same strand
misc_feature	no id	DDE superfamily endonuclease; Region: DDE_5; cl02413 intersects 93.57%(349aa) with IS4	e-value= 9.9e-07 complete sequence hit coverage= 100%, between model(486 aa) positions 1; 486 length is 376aa with 117 gaps, 0 stops, absolute frame= Plus1 at 3975325..3976452 in the same strand
Class A

133.
CDS	YP_001518376.1	transposase mutator family protein intersects 96.40%(402aa) with IS256	e-value= 3.7e-167 complete sequence hit coverage= 100%, between model(410 aa) positions 1; 410 length is 404aa with 11 gaps, 1 stops, absolute frame= Plus2 at 4126139..4127350 in the same strand

misc_feature no id Transposase, Mutator family; Region: Transposase_mut; pfam00872 intersects 100%(362aa) with IS256 e-value= 3.7e-167 complete sequence hit coverage= 100%, between model(410 aa) positions 1; 410 length is 404aa with 11 gaps, 1 stops, absolute frame= Plus2 at 4126139..4127350 in the same strand
misc_feature no id MULE transposase domain; Region: MULE; pfam10551 intersects 100%(78aa) with IS256 e-value= 3.7e-167 complete sequence hit coverage= 100%, between model(410 aa) positions 1; 410 length is 404aa with 11 gaps, 1 stops, absolute frame= Plus2 at 4126139..4127350 in the same strand
Class A

134.
CDS YP_001518385.1 hypothetical protein intersects 40.58%(37aa) with IS1_ORF1 e-value= 1.6e-06 complete sequence hit coverage= 100%, between model(239 aa) positions 1; 239 length is 229aa with 25 gaps, 10 stops, absolute frame= Minus1 at 4134334..4135019 in the same strand
CDS YP_001518386.1 hypothetical protein intersects 100%(68aa) with IS1_ORF1 e-value= 1.6e-06 complete sequence hit coverage= 100%, between model(239 aa) positions 1; 239 length is 229aa with 25 gaps, 10 stops, absolute frame= Minus1 at 4134334..4135019 in the same strand
CDS YP_001518385.1 hypothetical protein intersects 28.26%(26aa) with IS1_ORF2 e-value= 3.9e-08 fragment hit coverage= 20.68%, between model(237 aa) positions 164; 212 length is 46aa with 3 gaps, 1 stops, absolute frame= Minus3 at 4134368..4134504 in the same strand
CDS YP_001518386.1 hypothetical protein intersects 8.33%(5aa) with IS1_ORF2 e-value= 3.9e-08 fragment hit coverage= 20.68%, between model(237 aa) positions 164; 212 length is 46aa with 3 gaps, 1 stops, absolute frame= Minus3 at 4134368..4134504 in the same strand
Class B

135.
CDS YP_001518389.1 IS200 family transposase intersects 99.77%(142aa) with IS200 e-value= 5.7e-55 complete sequence hit coverage= 100%, between model(154 aa) positions 1; 154 length is 153aa with 3 gaps, 1 stops, absolute frame= Minus2 at 4135503..4135960 in the same strand
misc_feature no id Transposase IS200 like; Region: Y1_Tnp; cl00848 intersects 100%(117aa) with IS200 e-value= 5.7e-55 complete sequence hit coverage= 100%, between model(154 aa) positions 1; 154 length is 153aa with 3 gaps, 1 stops, absolute frame= Minus2 at 4135503..4135960 in the same strand
CDS YP_001518389.1 IS200 family transposase intersects 99.77%(142aa) with IS605 e-value= 5.5e-52 complete sequence hit coverage= 100%, between model(152 aa) positions 1; 152 length is 152aa with 2 gaps, 1 stops, absolute frame= Minus2 at 4135506..4135960 in the same strand
misc_feature no id Transposase IS200 like; Region: Y1_Tnp; cl00848 intersects 100%(117aa) with IS605 e-value= 5.5e-52 complete sequence hit coverage= 100%, between model(152 aa) positions 1; 152 length is 152aa with 2 gaps, 1 stops, absolute frame= Minus2 at 4135506..4135960 in the same strand
Class A

136.
gene no id fragment; similar to N-terminus of transposase intersects 38.55%(64aa) with IS630 e-value= 8.9e-08 fragment hit coverage= 17.02%, between model(376 aa) positions 1; 64 length is 64aa with 0 gaps, 0 stops, absolute frame= Plus2 at 4199279..4199470 in the same strand
Class A

137.
CDS YP_001518464.1 transposase intersects 7.69%(13aa) with IS630 e-value= 5.4e-08 fragment hit coverage= 19.15%, between model(376 aa) positions 305; 376 length is 73aa with 0 gaps, 2 stops, absolute frame= Plus3 at 4200300..4200518 in the same strand
misc_feature no id Integrase core domain; Region: rve; cl01316 intersects 8.11%(12aa) with IS630 e-value= 5.4e-08 fragment hit coverage= 19.15%, between model(376 aa) positions 305; 376 length is 73aa with 0 gaps, 2 stops, absolute frame= Plus3 at 4200300..4200518 in the same strand
CDS YP_001518465.1 hypothetical protein intersects 100%(48aa) with IS630 e-value= 5.4e-08 fragment hit coverage= 19.15%, between model(376 aa) positions 305; 376 length is 73aa with 0 gaps, 2 stops, absolute frame= Plus3 at 4200300..4200518 in the same strand
CDS YP_001518465.1 hypothetical protein intersects 62.50%(30aa) with IS3_IS150_ORF2 e-value= 4.8e-09 fragment hit coverage= 10.03%, between model(389 aa) positions 329; 367 length is 37aa with 2 gaps, 0 stops, absolute frame= Plus3 at 4200348..4200458 in the same strand

Class A

138.
CDS YP_001518488.1 hypothetical protein intersects 47.43%(129aa) with IS630 e-value= 1.1e-13 fragment hit coverage= 38.83%, between model(376 aa) positions 188; 333 length is 129aa with 20 gaps, 0 stops, absolute frame= Plus1 at 4217206..4217592 in the same strand
misc_feature no id Integrase core domain; Region: rve; cl01316 intersects 90.21%(129aa) with IS630 e-value= 1.1e-13 fragment hit coverage= 38.83%, between model(376 aa) positions 188; 333 length is 129aa with 20 gaps, 0 stops, absolute frame= Plus1 at 4217206..4217592 in the same strand
Class A

139.
CDS YP_001518490.1 hypothetical protein intersects 80.49%(141aa) with IS630 e-value= 6.2e-18 fragment hit coverage= 38.56%, between model(376 aa) positions 232; 376 length is 142aa with 4 gaps, 0 stops, absolute frame= Minus1 at 4218454..4218878 in the same strand
misc_feature no id Integrase core domain; Region: rve; cl01316 intersects 80.19%(110aa) with IS630 e-value= 6.2e-18 fragment hit coverage= 38.56%, between model(376 aa) positions 232; 376 length is 142aa with 4 gaps, 0 stops, absolute frame= Minus1 at 4218454..4218878 in the same strand
Class A

140.
CDS YP_001518491.1 hypothetical protein intersects 24.74%(31aa) with IS630 e-value= 1.8e-09 fragment hit coverage= 9.04%, between model(376 aa) positions 300; 333 length is 32aa with 2 gaps, 0 stops, absolute frame= Minus3 at 4219103..4219197 in the same strand
misc_feature no id Integrase core domain; Region: rve; cl01316 intersects 32.99%(31aa) with IS630 e-value= 1.8e-09 fragment hit coverage= 9.04%, between model(376 aa) positions 300; 333 length is 32aa with 2 gaps, 0 stops, absolute frame= Minus3 at 4219103..4219197 in the same strand
Class A

141.
CDS YP_001518493.1 transposase associated ATP binding protein intersects 86.41%(135aa) with IS630 e-value= 8.7e-15 fragment hit coverage= 39.63%, between model(376 aa) positions 1; 149 length is 136aa with 13 gaps, 0 stops, absolute frame= Minus1 at 4221091..4221497 in the same strand
misc_feature no id Winged helix-turn helix; Region: HTH_29; pfam13551 intersects 100%(102aa) with IS630 e-value= 8.7e-15 fragment hit coverage= 39.63%, between model(376 aa) positions 1; 149 length is 136aa with 13 gaps, 0 stops, absolute frame= Minus1 at 4221091..4221497 in the same strand
misc_feature no id Helix-turn-helix domains; Region: HTH; cl00088 intersects 100%(72aa) with IS630 e-value= 8.7e-15 fragment hit coverage= 39.63%, between model(376 aa) positions 1; 149 length is 136aa with 13 gaps, 0 stops, absolute frame= Minus1 at 4221091..4221497 in the same strand
misc_feature no id Helix-turn-helix domains; Region: HTH; cl00088 intersects 100%(50aa) with IS630 e-value= 8.7e-15 fragment hit coverage= 39.63%, between model(376 aa) positions 1; 149 length is 136aa with 13 gaps, 0 stops, absolute frame= Minus1 at 4221091..4221497 in the same strand
CDS YP_001518493.1 transposase associated ATP binding protein intersects 71.76%(112aa) with IS481 e-value= 3.5e-09 fragment hit coverage= 33.62%, between model(351 aa) positions 1; 118 length is 113aa with 6 gaps, 0 stops, absolute frame= Minus1 at 4221100..4221437 in the same strand
misc_feature no id Winged helix-turn helix; Region: HTH_29; pfam13551 intersects 100%(102aa) with IS481 e-value= 3.5e-09 fragment hit coverage= 33.62%, between model(351 aa) positions 1; 118 length is 113aa with 6 gaps, 0 stops, absolute frame= Minus1 at 4221100..4221437 in the same strand
misc_feature no id Helix-turn-helix domains; Region: HTH; cl00088 intersects 100%(72aa) with IS481 e-value= 3.5e-09 fragment hit coverage= 33.62%, between model(351 aa) positions 1; 118 length is 113aa with 6 gaps, 0 stops, absolute frame= Minus1 at 4221100..4221437 in the same strand
misc_feature no id Helix-turn-helix domains; Region: HTH; cl00088 intersects 100%(50aa) with IS481 e-value= 3.5e-09 fragment hit coverage= 33.62%, between model(351 aa) positions 1; 118 length is 113aa with 6 gaps, 0 stops, absolute frame= Minus1 at 4221100..4221437 in the same strand
Class A

142.

CDS YP_001518494.1 hypothetical protein intersects 29.26%(52aa) with IS481 e-value= 1.1e-08 fragment hit coverage= 15.10%, between model(351 aa) positions 1; 53 length is 53aa with 0 gaps, 0 stops, absolute frame= Minus1 at 4221850..4222007 in the same strand
misc_feature no id Winged helix-turn helix; Region: HTH_29; pfam13551 intersects 43.14%(44aa) with IS481 e-value= 1.1e-08 fragment hit coverage= 15.10%, between model(351 aa) positions 1; 53 length is 53aa with 0 gaps, 0 stops, absolute frame= Minus1 at 4221850..4222007 in the same strand
misc_feature no id Helix-turn-helix domains; Region: HTH; cl00088 intersects 100%(50aa) with IS481 e-value= 1.1e-08 fragment hit coverage= 15.10%, between model(351 aa) positions 1; 53 length is 53aa with 0 gaps, 0 stops, absolute frame= Minus1 at 4221850..4222007 in the same strand
Class B

143.
CDS YP_001518500.1 hypothetical protein intersects 3.11%(2aa) with IS630 e-value= 1.2e-10 complete sequence hit coverage= 100%, between model(376 aa) positions 1; 376 length is 332aa with 50 gaps, 3 stops, absolute frame= Plus1 at 4225801..4226796 in the same strand
CDS YP_001518501.1 transposase intersects 100%(270aa) with IS630 e-value= 1.2e-10 complete sequence hit coverage= 100%, between model(376 aa) positions 1; 376 length is 332aa with 50 gaps, 3 stops, absolute frame= Plus1 at 4225801..4226796 in the same strand
misc_feature no id Transposase; Region: HTH_Tnp_IS630; pfam01710 intersects 100%(99aa) with IS630 e-value= 1.2e-10 complete sequence hit coverage= 100%, between model(376 aa) positions 1; 376 length is 332aa with 50 gaps, 3 stops, absolute frame= Plus1 at 4225801..4226796 in the same strand
misc_feature no id Winged helix-turn helix; Region: HTH_33; pfam13592 intersects 100%(45aa) with IS630 e-value= 1.2e-10 complete sequence hit coverage= 100%, between model(376 aa) positions 1; 376 length is 332aa with 50 gaps, 3 stops, absolute frame= Plus1 at 4225801..4226796 in the same strand
misc_feature no id Integrase core domain; Region: rve; cl01316 intersects 100%(143aa) with IS630 e-value= 1.2e-10 complete sequence hit coverage= 100%, between model(376 aa) positions 1; 376 length is 332aa with 50 gaps, 3 stops, absolute frame= Plus1 at 4225801..4226796 in the same strand
CDS YP_001518502.1 hypothetical protein intersects 36.88%(17aa) with IS630 e-value= 1.2e-10 complete sequence hit coverage= 100%, between model(376 aa) positions 1; 376 length is 332aa with 50 gaps, 3 stops, absolute frame= Plus1 at 4225801..4226796 in the same strand
CDS YP_001518501.1 transposase intersects 72.96%(197aa) with IS630 e-value= 1.2e-14 fragment hit coverage= 59.57%, between model(376 aa) positions 110; 333 length is 197aa with 30 gaps, 0 stops, absolute frame= Plus1 at 4226083..4226673 in the same strand
misc_feature no id Transposase; Region: HTH_Tnp_IS630; pfam01710 intersects 51.52%(51aa) with IS630 e-value= 1.2e-14 fragment hit coverage= 59.57%, between model(376 aa) positions 110; 333 length is 197aa with 30 gaps, 0 stops, absolute frame= Plus1 at 4226083..4226673 in the same strand
misc_feature no id Winged helix-turn helix; Region: HTH_33; pfam13592 intersects 100%(45aa) with IS630 e-value= 1.2e-14 fragment hit coverage= 59.57%, between model(376 aa) positions 110; 333 length is 197aa with 30 gaps, 0 stops, absolute frame= Plus1 at 4226083..4226673 in the same strand
misc_feature no id Integrase core domain; Region: rve; cl01316 intersects 92.31%(132aa) with IS630 e-value= 1.2e-14 fragment hit coverage= 59.57%, between model(376 aa) positions 110; 333 length is 197aa with 30 gaps, 0 stops, absolute frame= Plus1 at 4226083..4226673 in the same strand
Class A

144.
CDS YP_001518503.1 transposase intersects 78.23%(115aa) with IS630 e-value= 1.2e-15 fragment hit coverage= 32.18%, between model(376 aa) positions 1; 121 length is 115aa with 7 gaps, 0 stops, absolute frame= Plus2 at 4226972..4227316 in the same strand
misc_feature no id Helix-turn-helix domains; Region: HTH; cl00088 intersects 100%(47aa) with IS630 e-value= 1.2e-15 fragment hit coverage= 32.18%, between model(376 aa) positions 1; 121 length is 115aa with 7 gaps, 0 stops, absolute frame= Plus2 at 4226972..4227316 in the same strand
misc_feature no id Winged helix-turn helix; Region: HTH_29; pfam13551 intersects 79.63%(86aa) with IS630 e-value= 1.2e-15 fragment hit coverage= 32.18%, between model(376 aa) positions 1; 121 length is 115aa with 7 gaps, 0 stops, absolute frame= Plus2 at 4226972..4227316 in the same strand
misc_feature no id Helix-turn-helix domains; Region: HTH; cl00088 intersects 75%(60aa) with IS630 e-value= 1.2e-15 fragment hit coverage= 32.18%, between model(376 aa) positions 1; 121 length is 115aa with 7 gaps, 0 stops, absolute frame= Plus2 at 4226972..4227316 in the same strand

Class A

145.
CDS YP_001518504.1 transposase intersects 50.95%(107aa) with IS630 e-value= 3.3e-07 fragment hit coverage= 27.39%, between model(376 aa) positions 274; 376 length is 107aa with 2 gaps, 0 stops, absolute frame= Plus1 at 4227730..4228050 in the same strand
misc_feature no id Integrase core domain; Region: rve; cl01316 intersects 49.67%(76aa) with IS630 e-value= 3.3e-07 fragment hit coverage= 27.39%, between model(376 aa) positions 274; 376 length is 107aa with 2 gaps, 0 stops, absolute frame= Plus1 at 4227730..4228050 in the same strand
Class A

146.
CDS YP_001518528.1 transposase intersects 86.04%(376aa) with IS4 e-value= 9.9e-07 complete sequence hit coverage= 100%, between model(486 aa) positions 1; 486 length is 376aa with 117 gaps, 0 stops, absolute frame= Plus3 at 4250727..4251854 in the same strand
misc_feature no id DDE superfamily endonuclease; Region: DDE_5; cl02413 intersects 93.57%(349aa) with IS4 e-value= 9.9e-07 complete sequence hit coverage= 100%, between model(486 aa) positions 1; 486 length is 376aa with 117 gaps, 0 stops, absolute frame= Plus3 at 4250727..4251854 in the same strand
CDS YP_001518528.1 transposase intersects 53.09%(232aa) with IS4 e-value= 4.7e-14 fragment hit coverage= 51.23%, between model(486 aa) positions 1; 249 length is 232aa with 27 gaps, 0 stops, absolute frame= Plus3 at 4250727..4251422 in the same strand
misc_feature no id DDE superfamily endonuclease; Region: DDE_5; cl02413 intersects 62.20%(232aa) with IS4 e-value= 4.7e-14 fragment hit coverage= 51.23%, between model(486 aa) positions 1; 249 length is 232aa with 27 gaps, 0 stops, absolute frame= Plus3 at 4250727..4251422 in the same strand
Class A

147.
CDS YP_001518529.1 hypothetical protein intersects 66.20%(141aa) with IS630 e-value= 6.2e-18 fragment hit coverage= 38.56%, between model(376 aa) positions 232; 376 length is 142aa with 4 gaps, 0 stops, absolute frame= Minus1 at 4251982..4252406 in the same strand
misc_feature no id Integrase core domain; Region: rve; cl01316 intersects 78.49%(110aa) with IS630 e-value= 6.2e-18 fragment hit coverage= 38.56%, between model(376 aa) positions 232; 376 length is 142aa with 4 gaps, 0 stops, absolute frame= Minus1 at 4251982..4252406 in the same strand
Class A

148.
CDS YP_001518530.1 hypothetical protein intersects 32.11%(52aa) with IS481 e-value= 1.1e-08 fragment hit coverage= 15.10%, between model(351 aa) positions 1; 53 length is 53aa with 0 gaps, 0 stops, absolute frame= Minus3 at 4252802..4252959 in the same strand
misc_feature no id Winged helix-turn helix; Region: HTH_29; pfam13551 intersects 45.36%(44aa) with IS481 e-value= 1.1e-08 fragment hit coverage= 15.10%, between model(351 aa) positions 1; 53 length is 53aa with 0 gaps, 0 stops, absolute frame= Minus3 at 4252802..4252959 in the same strand
misc_feature no id Helix-turn-helix domains; Region: HTH; cl00088 intersects 100%(50aa) with IS481 e-value= 1.1e-08 fragment hit coverage= 15.10%, between model(351 aa) positions 1; 53 length is 53aa with 0 gaps, 0 stops, absolute frame= Minus3 at 4252802..4252959 in the same strand
Class B

149.
CDS YP_001518560.1 hypothetical protein intersects 89.36%(42aa) with IS5_ISL2 e-value= 3.9e-10 complete sequence hit coverage= 100%, between model(260 aa) positions 1; 260 length is 185aa with 77 gaps, 5 stops, absolute frame= Plus3 at 4281402..4281956 in the same strand
CDS YP_001518560.1 hypothetical protein intersects 89.36%(42aa) with IS5_ISL2 e-value= 3.1e-31 fragment hit coverage= 26.54%, between model(260 aa) positions 192; 260 length is 69aa with 0 gaps, 1 stops, absolute frame= Plus3 at 4281750..4281956 in the same strand
Class B

150.

CDS YP_001518574.1 hypothetical protein intersects 93.42%(345aa) with IS630 e-value= 4.9e-24 complete sequence hit coverage= 100%, between model(376 aa) positions 1; 376 length is 346aa with 33 gaps, 0 stops, absolute frame= Minus2 at 4296789..4297825 in the same strand
misc_feature no id Integrase core domain; Region: rve; cl01316 intersects 100%(141aa) with IS630 e-value= 4.9e-24 complete sequence hit coverage= 100%, between model(376 aa) positions 1; 376 length is 346aa with 33 gaps, 0 stops, absolute frame= Minus2 at 4296789..4297825 in the same strand
misc_feature no id Winged helix-turn helix; Region: HTH_33; pfam13592 intersects 100%(56aa) with IS630 e-value= 4.9e-24 complete sequence hit coverage= 100%, between model(376 aa) positions 1; 376 length is 346aa with 33 gaps, 0 stops, absolute frame= Minus2 at 4296789..4297825 in the same strand
misc_feature no id Winged helix-turn helix; Region: HTH_29; pfam13551 intersects 100%(102aa) with IS630 e-value= 4.9e-24 complete sequence hit coverage= 100%, between model(376 aa) positions 1; 376 length is 346aa with 33 gaps, 0 stops, absolute frame= Minus2 at 4296789..4297825 in the same strand
misc_feature no id Helix-turn-helix domains; Region: HTH; cl00088 intersects 100%(50aa) with IS630 e-value= 4.9e-24 complete sequence hit coverage= 100%, between model(376 aa) positions 1; 376 length is 346aa with 33 gaps, 0 stops, absolute frame= Minus2 at 4296789..4297825 in the same strand
CDS YP_001518574.1 hypothetical protein intersects 14.23%(52aa) with IS481 e-value= 3.7e-09 fragment hit coverage= 15.10%, between model(351 aa) positions 1; 53 length is 53aa with 0 gaps, 0 stops, absolute frame= Minus2 at 4297608..4297765 in the same strand
misc_feature no id Winged helix-turn helix; Region: HTH_29; pfam13551 intersects 43.14%(44aa) with IS481 e-value= 3.7e-09 fragment hit coverage= 15.10%, between model(351 aa) positions 1; 53 length is 53aa with 0 gaps, 0 stops, absolute frame= Minus2 at 4297608..4297765 in the same strand
misc_feature no id Helix-turn-helix domains; Region: HTH; cl00088 intersects 100%(50aa) with IS481 e-value= 3.7e-09 fragment hit coverage= 15.10%, between model(351 aa) positions 1; 53 length is 53aa with 0 gaps, 0 stops, absolute frame= Minus2 at 4297608..4297765 in the same strand
Class A

151.
CDS YP_001518595.1 hypothetical protein intersects 27.35%(45aa) with IS1_ORF2 e-value= 1.8e-11 fragment hit coverage= 20.68%, between model(237 aa) positions 164; 212 length is 46aa with 3 gaps, 0 stops, absolute frame= Minus1 at 4319854..4319990 in the same strand
misc_feature no id IS1 transposase; Region: DDE_Tnp_IS1; cl00721 intersects 49.10%(45aa) with IS1_ORF2 e-value= 1.8e-11 fragment hit coverage= 20.68%, between model(237 aa) positions 164; 212 length is 46aa with 3 gaps, 0 stops, absolute frame= Minus1 at 4319854..4319990 in the same strand
Class A

152.
CDS YP_001518596.1 hypothetical protein intersects 66.92%(87aa) with IS1_ORF1 e-value= 1.7e-15 fragment hit coverage= 36.40%, between model(239 aa) positions 1; 87 length is 88aa with 2 gaps, 0 stops, absolute frame= Minus2 at 4320258..4320520 in the same strand
misc_feature no id Transposase and inactivated derivatives [DNA replication, recombination, and repair]; Region: COG3677 intersects 83.49%(87aa) with IS1_ORF1 e-value= 1.7e-15 fragment hit coverage= 36.40%, between model(239 aa) positions 1; 87 length is 88aa with 2 gaps, 0 stops, absolute frame= Minus2 at 4320258..4320520 in the same strand
Class A

153.
CDS YP_001518726.1 transposase intersects 59.84%(125aa) with IS630 e-value= 2.3e-07 fragment hit coverage= 32.45%, between model(376 aa) positions 255; 376 length is 126aa with 2 gaps, 0 stops, absolute frame= Minus2 at 4460748..4461124 in the same strand
misc_feature no id Integrase core domain; Region: rve; cl01316 intersects 61.87%(94aa) with IS630 e-value= 2.3e-07 fragment hit coverage= 32.45%, between model(376 aa) positions 255; 376 length is 126aa with 2 gaps, 0 stops, absolute frame= Minus2 at 4460748..4461124 in the same strand
Class A

154.

CDS YP_001518727.1 transposase intersects 78%(114aa) with IS630 e-value= 1.2e-15 fragment hit coverage= 32.18%, between model(376 aa) positions 1; 121 length is 115aa with 7 gaps, 0 stops, absolute frame= Minus3 at 4461482..4461825 in the same strand
misc_feature no id Winged helix-turn helix; Region: HTH_29; pfam13551 intersects 79.63%(86aa) with IS630 e-value= 1.2e-15 fragment hit coverage= 32.18%, between model(376 aa) positions 1; 121 length is 115aa with 7 gaps, 0 stops, absolute frame= Minus3 at 4461482..4461825 in the same strand
misc_feature no id Helix-turn-helix domains; Region: HTH; cl00088 intersects 75%(60aa) with IS630 e-value= 1.2e-15 fragment hit coverage= 32.18%, between model(376 aa) positions 1; 121 length is 115aa with 7 gaps, 0 stops, absolute frame= Minus3 at 4461482..4461825 in the same strand
misc_feature no id Helix-turn-helix domains; Region: HTH; cl00088 intersects 100%(47aa) with IS630 e-value= 1.2e-15 fragment hit coverage= 32.18%, between model(376 aa) positions 1; 121 length is 115aa with 7 gaps, 0 stops, absolute frame= Minus3 at 4461482..4461825 in the same strand
Class A

155.
CDS YP_001518866.1 hypothetical protein intersects 66.20%(141aa) with IS630 e-value= 6.2e-18 fragment hit coverage= 38.56%, between model(376 aa) positions 232; 376 length is 142aa with 4 gaps, 0 stops, absolute frame= Minus2 at 4611591..4612015 in the same strand
misc_feature no id Integrase core domain; Region: rve; cl01316 intersects 78.49%(110aa) with IS630 e-value= 6.2e-18 fragment hit coverage= 38.56%, between model(376 aa) positions 232; 376 length is 142aa with 4 gaps, 0 stops, absolute frame= Minus2 at 4611591..4612015 in the same strand
Class A

156.
CDS YP_001518867.1 hypothetical protein intersects 32.11%(52aa) with IS481 e-value= 1.1e-08 fragment hit coverage= 15.10%, between model(351 aa) positions 1; 53 length is 53aa with 0 gaps, 0 stops, absolute frame= Minus1 at 4612411..4612568 in the same strand
misc_feature no id Winged helix-turn helix; Region: HTH_29; pfam13551 intersects 45.36%(44aa) with IS481 e-value= 1.1e-08 fragment hit coverage= 15.10%, between model(351 aa) positions 1; 53 length is 53aa with 0 gaps, 0 stops, absolute frame= Minus1 at 4612411..4612568 in the same strand
misc_feature no id Helix-turn-helix domains; Region: HTH; cl00088 intersects 100%(50aa) with IS481 e-value= 1.1e-08 fragment hit coverage= 15.10%, between model(351 aa) positions 1; 53 length is 53aa with 0 gaps, 0 stops, absolute frame= Minus1 at 4612411..4612568 in the same strand
Class B

157.
gene no id fragment; similar to N-terminus of transposase intersects 41.19%(71aa) with IS630 e-value= 9.9e-08 fragment hit coverage= 20.74%, between model(376 aa) positions 44; 121 length is 72aa with 7 gaps, 0 stops, absolute frame= Minus1 at 4715806..4716020 in the same strand
Class A

158.
CDS YP_001519151.1 hypothetical protein intersects 47.30%(128aa) with IS630 e-value= 1.1e-13 fragment hit coverage= 38.83%, between model(376 aa) positions 188; 333 length is 129aa with 20 gaps, 0 stops, absolute frame= Minus1 at 4908313..4908698 in the same strand
misc_feature no id Integrase core domain; Region: rve; cl01316 intersects 89.98%(128aa) with IS630 e-value= 1.1e-13 fragment hit coverage= 38.83%, between model(376 aa) positions 188; 333 length is 129aa with 20 gaps, 0 stops, absolute frame= Minus1 at 4908313..4908698 in the same strand
Class A

159.
CDS YP_001519163.1 IS5 family transposase intersects 14.09%(81aa) with IS5_IS5 e-value= 2.6e-06 fragment hit coverage= 19.91%, between model(422 aa) positions 23; 106 length is 81aa with 3 gaps, 0 stops, absolute frame= Plus2 at 4926788..4927030 in the same strand
misc_feature no id Transposase domain (DUF772); Region: DUF772; pfam05598 intersects 74.03%(57aa) with IS5_IS5 e-value= 2.6e-06 fragment hit coverage= 19.91%, between model(422 aa) positions 23; 106 length is 81aa with 3 gaps, 0 stops, absolute frame= Plus2 at 4926788..4927030 in the same strand

Class A

160.
CDS YP_001519167.1 hypothetical protein intersects 80.33%(49aa) with IS1_ORF1 e-value= 3.2e-11 fragment hit coverage= 37.24%, between model(239 aa) positions 5; 93 length is 94aa with 2 gaps, 0 stops, absolute frame= Minus2 at 4929975..4930255 in the same strand
Class B

161.
CDS YP_001519171.1 transposase intersects 81.62%(365aa) with IS4 e-value= 1.2e-06 complete sequence hit coverage= 100%, between model(486 aa) positions 1; 486 length is 366aa with 126 gaps, 0 stops, absolute frame= Minus1 at 4932184..4933280 in the same strand
misc_feature no id DDE superfamily endonuclease; Region: DDE_5; cl02413 intersects 93.59%(311aa) with IS4 e-value= 1.2e-06 complete sequence hit coverage= 100%, between model(486 aa) positions 1; 486 length is 366aa with 126 gaps, 0 stops, absolute frame= Minus1 at 4932184..4933280 in the same strand
CDS YP_001519171.1 transposase intersects 49.48%(221aa) with IS4 e-value= 4.5e-12 fragment hit coverage= 53.29%, between model(486 aa) positions 1; 259 length is 222aa with 42 gaps, 0 stops, absolute frame= Minus1 at 4932616..4933280 in the same strand
misc_feature no id DDE superfamily endonuclease; Region: DDE_5; cl02413 intersects 66.57%(221aa) with IS4 e-value= 4.5e-12 fragment hit coverage= 53.29%, between model(486 aa) positions 1; 259 length is 222aa with 42 gaps, 0 stops, absolute frame= Minus1 at 4932616..4933280 in the same strand
Class A

162.
CDS YP_001519292.1 IS4 family transposase intersects 97.66%(376aa) with ISAs1 e-value= 1.5e-144 complete sequence hit coverage= 100%, between model(389 aa) positions 1; 389 length is 376aa with 16 gaps, 0 stops, absolute frame= Plus2 at 5053775..5054902 in the same strand
misc_feature no id DDE_Tnp_1-associated; Region: DDE_Tnp_1_assoc; pfam13808 intersects 100%(90aa) with ISAs1 e-value= 1.5e-144 complete sequence hit coverage= 100%, between model(389 aa) positions 1; 389 length is 376aa with 16 gaps, 0 stops, absolute frame= Plus2 at 5053775..5054902 in the same strand
misc_feature no id Transposase DDE domain; Region: DDE_Tnp_1; pfam01609 intersects 100%(238aa) with ISAs1 e-value= 1.5e-144 complete sequence hit coverage= 100%, between model(389 aa) positions 1; 389 length is 376aa with 16 gaps, 0 stops, absolute frame= Plus2 at 5053775..5054902 in the same strand
misc_feature no id Transposase [DNA replication, recombination, and repair]; Region: COG5433 intersects 100%(111aa) with ISAs1 e-value= 1.5e-144 complete sequence hit coverage= 100%, between model(389 aa) positions 1; 389 length is 376aa with 16 gaps, 0 stops, absolute frame= Plus2 at 5053775..5054902 in the same strand
misc_feature no id Transposase [DNA replication, recombination, and repair]; Region: COG5433 intersects 100%(61aa) with ISAs1 e-value= 1.5e-144 complete sequence hit coverage= 100%, between model(389 aa) positions 1; 389 length is 376aa with 16 gaps, 0 stops, absolute frame= Plus2 at 5053775..5054902 in the same strand
Class A

163.
gene no id fragment; similar to N-terminus of transposase intersects 36.87%(66aa) with IS630 e-value= 4e-07 fragment hit coverage= 17.55%, between model(376 aa) positions 1; 66 length is 66aa with 0 gaps, 0 stops, absolute frame= Plus2 at 5055743..5055940 in the same strand
Class A

164.
gene no id fragment; similar to C-terminus of transposase intersects 30.60%(71aa) with IS630 e-value= 4e-10 fragment hit coverage= 19.15%, between model(376 aa) positions 305; 376 length is 73aa with 0 gaps, 1 stops, absolute frame= Plus3 at 5056764..5056982 in the same strand
gene no id fragment; similar to C-terminus of transposase intersects 15.95%(37aa) with IS3_IS150_ORF2 e-value= 2e-08 fragment hit coverage= 10.03%, between model(389 aa) positions 329; 367 length is 37aa with 2 gaps, 0 stops, absolute frame= Plus3 at 5056812..5056922 in the same strand
Class A

165.

CDS YP_001519354.1 hypothetical protein intersects 0.67%(0aa) with IS5_IS1031e-value= 1.2e-20 fragment hit coverage= 21.36%, between model(295 aa) positions 15; 77 length is 56aa with 7 gaps, 0 stops, absolute frame= Minus3 at 5122250..5122416 in the same strand
Class B

166.
CDS YP_001519377.1 hypothetical protein intersects 93.51%(346aa) with IS630 e-value= 4.9e-24 complete sequence hit coverage= 100%, between model(376 aa) positions 1; 376 length is 346aa with 33 gaps, 0 stops, absolute frame= Plus3 at 5147340..5148377 in the same strand
misc_feature no id Helix-turn-helix domains; Region: HTH; cl00088 intersects 100%(50aa) with IS630 e-value= 4.9e-24 complete sequence hit coverage= 100%, between model(376 aa) positions 1; 376 length is 346aa with 33 gaps, 0 stops, absolute frame= Plus3 at 5147340..5148377 in the same strand
misc_feature no id Winged helix-turn helix; Region: HTH_29; pfam13551 intersects 100%(102aa) with IS630 e-value= 4.9e-24 complete sequence hit coverage= 100%, between model(376 aa) positions 1; 376 length is 346aa with 33 gaps, 0 stops, absolute frame= Plus3 at 5147340..5148377 in the same strand
misc_feature no id Winged helix-turn helix; Region: HTH_33; pfam13592 intersects 100%(56aa) with IS630 e-value= 4.9e-24 complete sequence hit coverage= 100%, between model(376 aa) positions 1; 376 length is 346aa with 33 gaps, 0 stops, absolute frame= Plus3 at 5147340..5148377 in the same strand
misc_feature no id Integrase core domain; Region: rve; cl01316 intersects 100%(141aa) with IS630 e-value= 4.9e-24 complete sequence hit coverage= 100%, between model(376 aa) positions 1; 376 length is 346aa with 33 gaps, 0 stops, absolute frame= Plus3 at 5147340..5148377 in the same strand
CDS YP_001519377.1 hypothetical protein intersects 14.32%(53aa) with IS481 e-value= 3.7e-09 fragment hit coverage= 15.10%, between model(351 aa) positions 1; 53 length is 53aa with 0 gaps, 0 stops, absolute frame= Plus3 at 5147400..5147558 in the same strand
misc_feature no id Helix-turn-helix domains; Region: HTH; cl00088 intersects 100%(50aa) with IS481 e-value= 3.7e-09 fragment hit coverage= 15.10%, between model(351 aa) positions 1; 53 length is 53aa with 0 gaps, 0 stops, absolute frame= Plus3 at 5147400..5147558 in the same strand
misc_feature no id Winged helix-turn helix; Region: HTH_29; pfam13551 intersects 43.14%(44aa) with IS481 e-value= 3.7e-09 fragment hit coverage= 15.10%, between model(351 aa) positions 1; 53 length is 53aa with 0 gaps, 0 stops, absolute frame= Plus3 at 5147400..5147558 in the same strand
Class A

167.
CDS YP_001519394.1 hypothetical protein intersects 4.02%(3aa) with IS5_ISL2 e-value= 1.5e-39 complete sequence hit coverage= 100%, between model(260 aa) positions 1; 260 length is 239aa with 23 gaps, 4 stops, absolute frame= Minus3 at 5174819..5175534 in the opposite strand
CDS YP_001519395.1 hypothetical protein intersects 89.36%(42aa) with IS5_ISL2 e-value= 1.5e-39 complete sequence hit coverage= 100%, between model(260 aa) positions 1; 260 length is 239aa with 23 gaps, 4 stops, absolute frame= Minus3 at 5174819..5175534 in the same strand
gene no id fragment; similar to N-terminus of transposase intersects 78.85%(123aa) with IS5_ISL2 e-value= 1.5e-39 complete sequence hit coverage= 100%, between model(260 aa) positions 1; 260 length is 239aa with 23 gaps, 4 stops, absolute frame= Minus3 at 5174819..5175534 in the same strand
CDS YP_001519394.1 hypothetical protein intersects 4.02%(3aa) with IS5_ISL2 e-value= 6.5e-62 fragment hit coverage= 51.54%, between model(260 aa) positions 127; 260 length is 134aa with 0 gaps, 0 stops, absolute frame= Minus3 at 5174819..5175219 in the opposite strand
CDS YP_001519395.1 hypothetical protein intersects 89.36%(42aa) with IS5_ISL2 e-value= 6.5e-62 fragment hit coverage= 51.54%, between model(260 aa) positions 127; 260 length is 134aa with 0 gaps, 0 stops, absolute frame= Minus3 at 5174819..5175219 in the same strand
gene no id fragment; similar to N-terminus of transposase intersects 11.54%(18aa) with IS5_ISL2 e-value= 6.5e-62 fragment hit coverage= 51.54%, between model(260 aa) positions 127; 260 length is 134aa with 0 gaps, 0 stops, absolute frame= Minus3 at 5174819..5175219 in the same strand
CDS YP_001519395.1 hypothetical protein intersects 70.92%(33aa) with IS5_ISL2 e-value= 3.2e-21 complete sequence hit coverage= 100%, between model(260 aa) positions 1; 260 length is 246aa with 22 gaps, 5 stops, absolute frame= Minus1 at 5174845..5175581 in the same strand
gene no id fragment; similar to N-terminus of transposase intersects 88.89%(138aa) with IS5_ISL2 e-value= 3.2e-21 complete sequence hit coverage= 100%, between model(260 aa) positions 1; 260 length is 246aa with 22 gaps, 5 stops, absolute frame= Minus1 at 5174845..5175581 in the same strand

gene no id fragment; similar to N-terminus of transposase intersects 66.45%(103aa) with IS5_ISL2 e-value= 7.6e-44 fragment hit coverage= 40%, between model(260 aa) positions 26; 129 length is 104aa with 0 gaps, 0 stops, absolute frame= Minus1 at 5175211..5175521 in the same strand
Class A

168.
CDS YP_001519397.1 hypothetical protein intersects 85.31%(122aa) with IS630 e-value= 8.7e-15 fragment hit coverage= 39.63%, between model(376 aa) positions 1; 149 length is 136aa with 13 gaps, 0 stops, absolute frame= Plus2 at 5176034..5176441 in the same strand
misc_feature no id Helix-turn-helix domains; Region: HTH; cl00088 intersects 100%(50aa) with IS630 e-value= 8.7e-15 fragment hit coverage= 39.63%, between model(376 aa) positions 1; 149 length is 136aa with 13 gaps, 0 stops, absolute frame= Plus2 at 5176034..5176441 in the same strand
misc_feature no id Winged helix-turn helix; Region: HTH_29; pfam13551 intersects 100%(102aa) with IS630 e-value= 8.7e-15 fragment hit coverage= 39.63%, between model(376 aa) positions 1; 149 length is 136aa with 13 gaps, 0 stops, absolute frame= Plus2 at 5176034..5176441 in the same strand
misc_feature no id Helix-turn-helix domains; Region: HTH; cl00088 intersects 100%(72aa) with IS630 e-value= 8.7e-15 fragment hit coverage= 39.63%, between model(376 aa) positions 1; 149 length is 136aa with 13 gaps, 0 stops, absolute frame= Plus2 at 5176034..5176441 in the same strand
CDS YP_001519397.1 hypothetical protein intersects 79.02%(113aa) with IS481 e-value= 3.5e-09 fragment hit coverage= 33.62%, between model(351 aa) positions 1; 118 length is 113aa with 6 gaps, 0 stops, absolute frame= Plus2 at 5176094..5176432 in the same strand
misc_feature no id Helix-turn-helix domains; Region: HTH; cl00088 intersects 100%(50aa) with IS481 e-value= 3.5e-09 fragment hit coverage= 33.62%, between model(351 aa) positions 1; 118 length is 113aa with 6 gaps, 0 stops, absolute frame= Plus2 at 5176094..5176432 in the same strand
misc_feature no id Winged helix-turn helix; Region: HTH_29; pfam13551 intersects 100%(102aa) with IS481 e-value= 3.5e-09 fragment hit coverage= 33.62%, between model(351 aa) positions 1; 118 length is 113aa with 6 gaps, 0 stops, absolute frame= Plus2 at 5176094..5176432 in the same strand
misc_feature no id Helix-turn-helix domains; Region: HTH; cl00088 intersects 100%(72aa) with IS481 e-value= 3.5e-09 fragment hit coverage= 33.62%, between model(351 aa) positions 1; 118 length is 113aa with 6 gaps, 0 stops, absolute frame= Plus2 at 5176094..5176432 in the same strand
Class B

169.
misc_feature no id Integrase core domain; Region: rve; cl01316 intersects 100%(128aa) with IS630 e-value= 6.5e-11 fragment hit coverage= 39.10%, between model(376 aa) positions 187; 333 length is 130aa with 21 gaps, 0 stops, absolute frame= Plus1 at 5176546..5176935 in the same strand
CDS YP_001519398.1 hypothetical protein intersects 80%(128aa) with IS630 e-value= 6.5e-11 fragment hit coverage= 39.10%, between model(376 aa) positions 187; 333 length is 130aa with 21 gaps, 0 stops, absolute frame= Plus1 at 5176546..5176935 in the same strand
Class A

170.
CDS YP_001519428.1 IS4 family transposase intersects 97.66%(376aa) with ISAs1 e-value= 1.8e-144 complete sequence hit coverage= 100%, between model(389 aa) positions 1; 389 length is 376aa with 16 gaps, 0 stops, absolute frame= Plus3 at 5209563..5210690 in the same strand
misc_feature no id DDE_Tnp_1-associated; Region: DDE_Tnp_1_assoc; pfam13808 intersects 100%(90aa) with ISAs1 e-value= 1.8e-144 complete sequence hit coverage= 100%, between model(389 aa) positions 1; 389 length is 376aa with 16 gaps, 0 stops, absolute frame= Plus3 at 5209563..5210690 in the same strand
misc_feature no id Transposase DDE domain; Region: DDE_Tnp_1; pfam01609 intersects 100%(239aa) with ISAs1 e-value= 1.8e-144 complete sequence hit coverage= 100%, between model(389 aa) positions 1; 389 length is 376aa with 16 gaps, 0 stops, absolute frame= Plus3 at 5209563..5210690 in the same strand
misc_feature no id Transposase [DNA replication, recombination, and repair]; Region: COG5433 intersects 100%(111aa) with ISAs1 e-value= 1.8e-144 complete sequence hit coverage= 100%, between model(389 aa) positions 1; 389 length is 376aa with 16 gaps, 0 stops, absolute frame= Plus3 at 5209563..5210690 in the same strand
misc_feature no id Transposase [DNA replication, recombination, and repair]; Region: COG5433 intersects 100%(82aa) with ISAs1 e-value= 1.8e-144 complete sequence hit coverage= 100%, between model(389 aa) positions 1; 389 length is 376aa with 16 gaps, 0 stops, absolute frame= Plus3 at 5209563..5210690 in the same strand

Class A

171.
CDS YP_001519457.1 transposase intersects 84.83%(123aa) with IS630 e-value= 4e-14 fragment hit coverage= 38.83%, between model(376 aa) positions 188; 333 length is 129aa with 20 gaps, 0 stops, absolute frame= Plus1 at 5240533..5240919 in the same strand
misc_feature no id Integrase core domain; Region: rve; cl01316 intersects 91.73%(122aa) with IS630 e-value= 4e-14 fragment hit coverage= 38.83%, between model(376 aa) positions 188; 333 length is 129aa with 20 gaps, 0 stops, absolute frame= Plus1 at 5240533..5240919 in the same strand
Class A

172.
CDS YP_001519458.1 transposase intersects 85.96%(375aa) with IS4 e-value= 9.9e-07 complete sequence hit coverage= 100%, between model(486 aa) positions 1; 486 length is 376aa with 117 gaps, 0 stops, absolute frame= Minus1 at 5241106..5242232 in the same strand
misc_feature no id DDE superfamily endonuclease; Region: DDE_5; cl02413 intersects 93.48%(348aa) with IS4 e-value= 9.9e-07 complete sequence hit coverage= 100%, between model(486 aa) positions 1; 486 length is 376aa with 117 gaps, 0 stops, absolute frame= Minus1 at 5241106..5242232 in the same strand
CDS YP_001519458.1 transposase intersects 53.01%(231aa) with IS4 e-value= 4.7e-14 fragment hit coverage= 51.23%, between model(486 aa) positions 1; 249 length is 232aa with 27 gaps, 0 stops, absolute frame= Minus1 at 5241538..5242232 in the same strand
misc_feature no id DDE superfamily endonuclease; Region: DDE_5; cl02413 intersects 62.11%(231aa) with IS4 e-value= 4.7e-14 fragment hit coverage= 51.23%, between model(486 aa) positions 1; 249 length is 232aa with 27 gaps, 0 stops, absolute frame= Minus1 at 5241538..5242232 in the same strand
Class A

173.
CDS YP_001519511.1 hypothetical protein intersects 44.94%(80aa) with IS481 e-value= 1.2e-07 fragment hit coverage= 23.36%, between model(351 aa) positions 1; 82 length is 80aa with 2 gaps, 0 stops, absolute frame= Plus3 at 5296908..5297147 in the same strand
misc_feature no id Helix-turn-helix domains; Region: HTH; cl00088 intersects 100%(47aa) with IS481 e-value= 1.2e-07 fragment hit coverage= 23.36%, between model(351 aa) positions 1; 82 length is 80aa with 2 gaps, 0 stops, absolute frame= Plus3 at 5296908..5297147 in the same strand
misc_feature no id Winged helix-turn helix; Region: HTH_29; pfam13551 intersects 63.39%(71aa) with IS481 e-value= 1.2e-07 fragment hit coverage= 23.36%, between model(351 aa) positions 1; 82 length is 80aa with 2 gaps, 0 stops, absolute frame= Plus3 at 5296908..5297147 in the same strand
CDS YP_001519511.1 hypothetical protein intersects 76.40%(136aa) with IS630 e-value= 1.9e-29 fragment hit coverage= 37.50%, between model(376 aa) positions 30; 170 length is 136aa with 5 gaps, 0 stops, absolute frame= Plus3 at 5296935..5297342 in the same strand
misc_feature no id Helix-turn-helix domains; Region: HTH; cl00088 intersects 93.62%(44aa) with IS630 e-value= 1.9e-29 fragment hit coverage= 37.50%, between model(376 aa) positions 30; 170 length is 136aa with 5 gaps, 0 stops, absolute frame= Plus3 at 5296935..5297342 in the same strand
misc_feature no id Winged helix-turn helix; Region: HTH_29; pfam13551 intersects 100%(112aa) with IS630 e-value= 1.9e-29 fragment hit coverage= 37.50%, between model(376 aa) positions 30; 170 length is 136aa with 5 gaps, 0 stops, absolute frame= Plus3 at 5296935..5297342 in the same strand
misc_feature no id Winged helix-turn helix; Region: HTH_33; pfam13592 intersects 98.33%(59aa) with IS630 e-value= 1.9e-29 fragment hit coverage= 37.50%, between model(376 aa) positions 30; 170 length is 136aa with 5 gaps, 0 stops, absolute frame= Plus3 at 5296935..5297342 in the same strand
Class B

174.
CDS YP_001519512.1 hypothetical protein intersects 27.42%(51aa) with IS630 e-value= 4.3e-06 fragment hit coverage= 11.70%, between model(376 aa) positions 279; 322 length is 51aa with 1 gaps, 0 stops, absolute frame= Plus1 at 5297623..5297775 in the same strand
misc_feature no id Integrase core domain; Region: rve; cl01316 intersects 38.35%(51aa) with IS630 e-value= 4.3e-06 fragment hit coverage= 11.70%, between model(376 aa) positions 279; 322 length is 51aa with 1 gaps, 0 stops, absolute frame= Plus1 at 5297623..5297775 in the same strand

Class A

175.
CDS YP_001519548.1 hypothetical protein intersects 89.36%(42aa) with IS5_ISL2 e-value= 6.9e-39 complete sequence hit coverage= 100%, between model(260 aa) positions 1; 260 length is 239aa with 23 gaps, 4 stops, absolute frame= Minus2 at 5338023..5338738 in the same strand
gene no id fragment; similar to N-terminus of transposase intersects 78.85%(123aa) with IS5_ISL2 e-value= 6.9e-39 complete sequence hit coverage= 100%, between model(260 aa) positions 1; 260 length is 239aa with 23 gaps, 4 stops, absolute frame= Minus2 at 5338023..5338738 in the same strand
CDS YP_001519548.1 hypothetical protein intersects 89.36%(42aa) with IS5_ISL2 e-value= 6.5e-62 fragment hit coverage= 51.54%, between model(260 aa) positions 127; 260 length is 134aa with 0 gaps, 0 stops, absolute frame= Minus2 at 5338023..5338423 in the same strand
gene no id fragment; similar to N-terminus of transposase intersects 11.54%(18aa) with IS5_ISL2 e-value= 6.5e-62 fragment hit coverage= 51.54%, between model(260 aa) positions 127; 260 length is 134aa with 0 gaps, 0 stops, absolute frame= Minus2 at 5338023..5338423 in the same strand
CDS YP_001519548.1 hypothetical protein intersects 70.92%(33aa) with IS5_ISL2 e-value= 3.1e-21 complete sequence hit coverage= 100%, between model(260 aa) positions 1; 260 length is 246aa with 22 gaps, 5 stops, absolute frame= Minus3 at 5338049..5338785 in the same strand
gene no id fragment; similar to N-terminus of transposase intersects 88.89%(138aa) with IS5_ISL2 e-value= 3.1e-21 complete sequence hit coverage= 100%, between model(260 aa) positions 1; 260 length is 246aa with 22 gaps, 5 stops, absolute frame= Minus3 at 5338049..5338785 in the same strand
gene no id fragment; similar to N-terminus of transposase intersects 66.45%(103aa) with IS5_ISL2 e-value= 9.3e-44 fragment hit coverage= 40%, between model(260 aa) positions 26; 129 length is 104aa with 0 gaps, 0 stops, absolute frame= Minus3 at 5338415..5338725 in the same strand
Class A

176.
CDS YP_001519553.1 hypothetical protein intersects 44.94%(80aa) with IS481 e-value= 1.2e-07 fragment hit coverage= 23.36%, between model(351 aa) positions 1; 82 length is 80aa with 2 gaps, 0 stops, absolute frame= Plus2 at 5341466..5341705 in the same strand
misc_feature no id Helix-turn-helix domains; Region: HTH; cl00088 intersects 100%(47aa) with IS481 e-value= 1.2e-07 fragment hit coverage= 23.36%, between model(351 aa) positions 1; 82 length is 80aa with 2 gaps, 0 stops, absolute frame= Plus2 at 5341466..5341705 in the same strand
misc_feature no id Winged helix-turn helix; Region: HTH_29; pfam13551 intersects 63.39%(71aa) with IS481 e-value= 1.2e-07 fragment hit coverage= 23.36%, between model(351 aa) positions 1; 82 length is 80aa with 2 gaps, 0 stops, absolute frame= Plus2 at 5341466..5341705 in the same strand
CDS YP_001519553.1 hypothetical protein intersects 76.40%(136aa) with IS630 e-value= 1.9e-29 fragment hit coverage= 37.50%, between model(376 aa) positions 30; 170 length is 136aa with 5 gaps, 0 stops, absolute frame= Plus2 at 5341493..5341900 in the same strand
misc_feature no id Helix-turn-helix domains; Region: HTH; cl00088 intersects 93.62%(44aa) with IS630 e-value= 1.9e-29 fragment hit coverage= 37.50%, between model(376 aa) positions 30; 170 length is 136aa with 5 gaps, 0 stops, absolute frame= Plus2 at 5341493..5341900 in the same strand
misc_feature no id Winged helix-turn helix; Region: HTH_29; pfam13551 intersects 100%(112aa) with IS630 e-value= 1.9e-29 fragment hit coverage= 37.50%, between model(376 aa) positions 30; 170 length is 136aa with 5 gaps, 0 stops, absolute frame= Plus2 at 5341493..5341900 in the same strand
misc_feature no id Winged helix-turn helix; Region: HTH_33; pfam13592 intersects 98.33%(59aa) with IS630 e-value= 1.9e-29 fragment hit coverage= 37.50%, between model(376 aa) positions 30; 170 length is 136aa with 5 gaps, 0 stops, absolute frame= Plus2 at 5341493..5341900 in the same strand
Class B

177.
CDS YP_001519554.1 hypothetical protein intersects 27.42%(51aa) with IS630 e-value= 4.3e-06 fragment hit coverage= 11.70%, between model(376 aa) positions 279; 322 length is 51aa with 1 gaps, 0 stops, absolute frame= Plus3 at 5342181..5342333 in the same strand
misc_feature no id Integrase core domain; Region: rve; cl01316 intersects 38.35%(51aa) with IS630 e-value= 4.3e-06 fragment hit coverage= 11.70%, between model(376 aa) positions 279; 322 length is 51aa with 1 gaps, 0 stops, absolute frame= Plus3 at 5342181..5342333 in the same strand

Class A

178.
CDS YP_001519566.1 transposase intersects 86.04%(376aa) with IS4 e-value= 9.9e-07 complete sequence hit coverage= 100%, between model(486 aa) positions 1; 486 length is 376aa with 117 gaps, 0 stops, absolute frame= Plus3 at 5352645..5353772 in the same strand
misc_feature no id DDE superfamily endonuclease; Region: DDE_5; cl02413 intersects 93.57%(349aa) with IS4 e-value= 9.9e-07 complete sequence hit coverage= 100%, between model(486 aa) positions 1; 486 length is 376aa with 117 gaps, 0 stops, absolute frame= Plus3 at 5352645..5353772 in the same strand
CDS YP_001519566.1 transposase intersects 53.09%(232aa) with IS4 e-value= 4.7e-14 fragment hit coverage= 51.23%, between model(486 aa) positions 1; 249 length is 232aa with 27 gaps, 0 stops, absolute frame= Plus3 at 5352645..5353340 in the same strand
misc_feature no id DDE superfamily endonuclease; Region: DDE_5; cl02413 intersects 62.20%(232aa) with IS4 e-value= 4.7e-14 fragment hit coverage= 51.23%, between model(486 aa) positions 1; 249 length is 232aa with 27 gaps, 0 stops, absolute frame= Plus3 at 5352645..5353340 in the same strand
Class A

179.
CDS YP_001519568.1 hypothetical protein intersects 89.36%(42aa) with IS5_ISL2 e-value= 4.7e-61 fragment hit coverage= 51.54%, between model(260 aa) positions 127; 260 length is 134aa with 0 gaps, 0 stops, absolute frame= Minus1 at 5354170..5354570 in the same strand
gene no id fragment; similar to N-terminus of transposase intersects 11.54%(18aa) with IS5_ISL2 e-value= 4.7e-61 fragment hit coverage= 51.54%, between model(260 aa) positions 127; 260 length is 134aa with 0 gaps, 0 stops, absolute frame= Minus1 at 5354170..5354570 in the same strand
CDS YP_001519568.1 hypothetical protein intersects 89.36%(42aa) with IS5_ISL2 e-value= 8.9e-38 complete sequence hit coverage= 100%, between model(260 aa) positions 1; 260 length is 239aa with 23 gaps, 5 stops, absolute frame= Minus1 at 5354170..5354885 in the same strand
gene no id fragment; similar to N-terminus of transposase intersects 78.85%(123aa) with IS5_ISL2 e-value= 8.9e-38 complete sequence hit coverage= 100%, between model(260 aa) positions 1; 260 length is 239aa with 23 gaps, 5 stops, absolute frame= Minus1 at 5354170..5354885 in the same strand
CDS YP_001519568.1 hypothetical protein intersects 70.92%(33aa) with IS5_ISL2 e-value= 9.6e-21 complete sequence hit coverage= 100%, between model(260 aa) positions 1; 260 length is 246aa with 23 gaps, 5 stops, absolute frame= Minus2 at 5354196..5354932 in the same strand
gene no id fragment; similar to N-terminus of transposase intersects 88.89%(138aa) with IS5_ISL2 e-value= 9.6e-21 complete sequence hit coverage= 100%, between model(260 aa) positions 1; 260 length is 246aa with 23 gaps, 5 stops, absolute frame= Minus2 at 5354196..5354932 in the same strand
gene no id fragment; similar to N-terminus of transposase intersects 66.45%(103aa) with IS5_ISL2 e-value= 9.3e-44 fragment hit coverage= 40%, between model(260 aa) positions 26; 129 length is 104aa with 0 gaps, 0 stops, absolute frame= Minus2 at 5354562..5354872 in the same strand
Class A

180.
gene no id fragment; similar to N-terminus of transposase intersects 36.87%(66aa) with IS630 e-value= 4e-07 fragment hit coverage= 17.55%, between model(376 aa) positions 1; 66 length is 66aa with 0 gaps, 0 stops, absolute frame= Plus3 at 5358090..5358287 in the same strand
Class A

181.
gene no id fragment; similar to C-terminus of transposase intersects 30.60%(71aa) with IS630 e-value= 4e-10 fragment hit coverage= 19.15%, between model(376 aa) positions 305; 376 length is 73aa with 0 gaps, 1 stops, absolute frame= Plus1 at 5359111..5359329 in the same strand
CDS YP_001519572.1 hypothetical protein intersects 4.68%(10aa) with IS630 e-value= 4e-10 fragment hit coverage= 19.15%, between model(376 aa) positions 305; 376 length is 73aa with 0 gaps, 1 stops, absolute frame= Plus1 at 5359111..5359329 in the opposite strand
gene no id fragment; similar to C-terminus of transposase intersects 15.95%(37aa) with IS3_IS150_ORF2 e-value= 2e-08 fragment hit coverage= 10.03%, between model(389 aa) positions 329; 367 length is 37aa with 2 gaps, 0 stops, absolute frame= Plus1 at 5359159..5359269 in the same strand

Class A

182.
CDS YP_001519691.1 IS200 family transposase intersects 99.77%(142aa) with IS200 e-value= 5.7e-55 complete sequence hit coverage= 100%, between model(154 aa) positions 1; 154 length is 153aa with 3 gaps, 1 stops, absolute frame= Minus2 at 5474574..5475031 in the same strand
misc_feature no id Transposase IS200 like; Region: Y1_Tnp; cl00848 intersects 100%(117aa) with IS200 e-value= 5.7e-55 complete sequence hit coverage= 100%, between model(154 aa) positions 1; 154 length is 153aa with 3 gaps, 1 stops, absolute frame= Minus2 at 5474574..5475031 in the same strand
CDS YP_001519691.1 IS200 family transposase intersects 99.77%(142aa) with IS605 e-value= 5.5e-52 complete sequence hit coverage= 100%, between model(152 aa) positions 1; 152 length is 152aa with 2 gaps, 1 stops, absolute frame= Minus2 at 5474577..5475031 in the same strand
misc_feature no id Transposase IS200 like; Region: Y1_Tnp; cl00848 intersects 100%(117aa) with IS605 e-value= 5.5e-52 complete sequence hit coverage= 100%, between model(152 aa) positions 1; 152 length is 152aa with 2 gaps, 1 stops, absolute frame= Minus2 at 5474577..5475031 in the same strand
Class A

183.
CDS YP_001519725.1 hypothetical protein intersects 27.35%(45aa) with IS1_ORF2 e-value= 1.8e-11 fragment hit coverage= 20.68%, between model(237 aa) positions 164; 212 length is 46aa with 3 gaps, 0 stops, absolute frame= Minus1 at 5509063..5509199 in the same strand
misc_feature no id IS1 transposase; Region: DDE_Tnp_IS1; cl00721 intersects 49.10%(45aa) with IS1_ORF2 e-value= 1.8e-11 fragment hit coverage= 20.68%, between model(237 aa) positions 164; 212 length is 46aa with 3 gaps, 0 stops, absolute frame= Minus1 at 5509063..5509199 in the same strand
Class A

184.
CDS YP_001519726.1 hypothetical protein intersects 66.92%(87aa) with IS1_ORF1 e-value= 1.7e-15 fragment hit coverage= 36.40%, between model(239 aa) positions 1; 87 length is 88aa with 2 gaps, 0 stops, absolute frame= Minus2 at 5509467..5509729 in the same strand
misc_feature no id Transposase and inactivated derivatives [DNA replication, recombination, and repair]; Region: COG3677 intersects 83.49%(87aa) with IS1_ORF1 e-value= 1.7e-15 fragment hit coverage= 36.40%, between model(239 aa) positions 1; 87 length is 88aa with 2 gaps, 0 stops, absolute frame= Minus2 at 5509467..5509729 in the same strand
Class A

185.
CDS YP_001519727.1 transposase intersects 84.83%(359aa) with ISL3 e-value= 2.6e-50 fragment hit coverage= 86.03%, between model(451 aa) positions 64; 451 length is 360aa with 34 gaps, 0 stops, absolute frame= Minus2 at 5509761..5510839 in the same strand
misc_feature no id Transposase and inactivated derivatives [DNA replication, recombination, and repair]; Region: COG3464 intersects 85.77%(345aa) with ISL3 e-value= 2.6e-50 fragment hit coverage= 86.03%, between model(451 aa) positions 64; 451 length is 360aa with 34 gaps, 0 stops, absolute frame= Minus2 at 5509761..5510839 in the same strand
misc_feature no id Transposase; Region: DDE_Tnp_ISL3; pfam01610 intersects 100%(246aa) with ISL3 e-value= 2.6e-50 fragment hit coverage= 86.03%, between model(451 aa) positions 64; 451 length is 360aa with 34 gaps, 0 stops, absolute frame= Minus2 at 5509761..5510839 in the same strand
CDS YP_001519727.1 transposase intersects 97.33%(412aa) with ISL3 e-value= 4.5e-44 complete sequence hit coverage= 100%, between model(451 aa) positions 1; 451 length is 413aa with 44 gaps, 0 stops, absolute frame= Minus2 at 5509761..5510998 in the same strand
misc_feature no id Transposase and inactivated derivatives [DNA replication, recombination, and repair]; Region: COG3464 intersects 98.92%(398aa) with ISL3 e-value= 4.5e-44 complete sequence hit coverage= 100%, between model(451 aa) positions 1; 451 length is 413aa with 44 gaps, 0 stops, absolute frame= Minus2 at 5509761..5510998 in the same strand
misc_feature no id Transposase; Region: DDE_Tnp_ISL3; pfam01610 intersects 100%(246aa) with ISL3 e-value= 4.5e-44 complete sequence hit coverage= 100%, between model(451 aa) positions 1; 451 length is 413aa with 44 gaps, 0 stops, absolute frame= Minus2 at 5509761..5510998 in the same strand

Class A

186.
CDS YP_001519738.1 hypothetical protein intersects 23.18%(45aa) with IS1_ORF2 e-value= 1.6e-12 fragment hit coverage= 20.68%, between model(237 aa) positions 164; 212 length is 46aa with 3 gaps, 0 stops, absolute frame= Minus2 at 5521956..5522092 in the same strand
misc_feature no id IS1 transposase; Region: DDE_Tnp_IS1; cl00721 intersects 39.03%(45aa) with IS1_ORF2 e-value= 1.6e-12 fragment hit coverage= 20.68%, between model(237 aa) positions 164; 212 length is 46aa with 3 gaps, 0 stops, absolute frame= Minus2 at 5521956..5522092 in the same strand
Class A

187.
CDS YP_001519739.1 hypothetical protein intersects 100%(54aa) with IS1_ORF1 e-value= 9e-13 fragment hit coverage= 33.89%, between model(239 aa) positions 1; 81 length is 85aa with 2 gaps, 1 stops, absolute frame= Minus3 at 5522375..5522628 in the opposite strand
Class B

188.
CDS YP_001519763.1 transposase intersects 53.09%(232aa) with IS4 e-value= 2.3e-13 fragment hit coverage= 51.23%, between model(486 aa) positions 1; 249 length is 232aa with 27 gaps, 0 stops, absolute frame= Plus2 at 5545037..5545732 in the same strand
misc_feature no id DDE superfamily endonuclease; Region: DDE_5; cl02413 intersects 62.20%(232aa) with IS4 e-value= 2.3e-13 fragment hit coverage= 51.23%, between model(486 aa) positions 1; 249 length is 232aa with 27 gaps, 0 stops, absolute frame= Plus2 at 5545037..5545732 in the same strand
CDS YP_001519763.1 transposase intersects 88.79%(388aa) with IS4 e-value= 1.2e-06 complete sequence hit coverage= 100%, between model(486 aa) positions 1; 486 length is 388aa with 105 gaps, 0 stops, absolute frame= Plus2 at 5545037..5546200 in the same strand
misc_feature no id DDE superfamily endonuclease; Region: DDE_5; cl02413 intersects 93.57%(349aa) with IS4 e-value= 1.2e-06 complete sequence hit coverage= 100%, between model(486 aa) positions 1; 486 length is 388aa with 105 gaps, 0 stops, absolute frame= Plus2 at 5545037..5546200 in the same strand
Class A

189.
CDS YP_001519864.1 transposase intersects 44.30%(66aa) with IS630 e-value= 1.4e-09 fragment hit coverage= 19.15%, between model(376 aa) positions 275; 346 length is 66aa with 6 gaps, 0 stops, absolute frame= Plus3 at 5653053..5653250 in the same strand
misc_feature no id Integrase core domain; Region: rve; cl01316 intersects 54.62%(65aa) with IS630 e-value= 1.4e-09 fragment hit coverage= 19.15%, between model(376 aa) positions 275; 346 length is 66aa with 6 gaps, 0 stops, absolute frame= Plus3 at 5653053..5653250 in the same strand
Class A

190.
gene no id fragment; similar to C-terminus of transposase, IS200 family intersects 100%(87aa) with IS200 e-value= 5.1e-50 complete sequence hit coverage= 100%, between model(154 aa) positions 1; 154 length is 153aa with 3 gaps, 2 stops, absolute frame= Plus2 at 5653406..5653864 in the same strand
gene no id fragment; similar to C-terminus of transposase, IS200 family intersects 100%(87aa) with IS605 e-value= 8.2e-45 complete sequence hit coverage= 100%, between model(152 aa) positions 1; 152 length is 152aa with 2 gaps, 2 stops, absolute frame= Plus2 at 5653406..5653861 in the same strand
Class A

191.
CDS YP_001519917.1 hypothetical protein intersects 5.50%(10aa) with IS630 e-value= 4e-10 fragment hit coverage= 19.15%, between model(376 aa) positions 305; 376 length is 73aa with 0 gaps, 1 stops, absolute frame= Minus2 at 5716776..5716993 in the opposite strand
gene no id fragment; similar to C-terminus of transposase intersects 30.46%(70aa) with IS630 e-value= 4e-10 fragment hit coverage= 19.15%, between model(376 aa) positions 305; 376 length is 73aa with 0 gaps, 1 stops, absolute frame= Minus2 at 5716776..5716993 in the same strand

gene no id fragment; similar to C-terminus of transposase intersects 15.80%(36aa) with IS3_IS150_ORF2 e-value= 2e-08 fragment hit coverage= 10.03%, between model(389 aa) positions 329; 367 length is 37aa with 2 gaps, 0 stops, absolute frame= Minus2 at 5716836..5716945 in the same strand
Class A

192.
gene no id fragment; similar to N-terminus of transposase intersects 36.69%(65aa) with IS630 e-value= 4e-07 fragment hit coverage= 17.55%, between model(376 aa) positions 1; 66 length is 66aa with 0 gaps, 0 stops, absolute frame= Minus1 at 5717818..5718014 in the same strand
Class A

193.
CDS YP_001519918.1 mechanosensitive ion channel intersects 10.99%(39aa) with IS1_ORF2 e-value= 6.2e-32 fragment hit coverage= 54.43%, between model(237 aa) positions 109; 237 length is 129aa with 1 gaps, 0 stops, absolute frame= Minus1 at 5719186..5719571 in the opposite strand
gene no id fragment; similar to N-terminus of transposase intersects 0.32%(0aa) with IS1_ORF2 e-value= 6.2e-32 fragment hit coverage= 54.43%, between model(237 aa) positions 109; 237 length is 129aa with 1 gaps, 0 stops, absolute frame= Minus1 at 5719186..5719571 in the same strand
CDS YP_001519918.1 mechanosensitive ion channel intersects 10.99%(39aa) with IS1_ORF2 e-value= 4.6e-13 complete sequence hit coverage= 100%, between model(237 aa) positions 1; 237 length is 213aa with 27 gaps, 3 stops, absolute frame= Minus1 at 5719186..5719823 in the opposite strand
gene no id fragment; similar to N-terminus of transposase intersects 81.88%(84aa) with IS1_ORF2 e-value= 4.6e-13 complete sequence hit coverage= 100%, between model(237 aa) positions 1; 237 length is 213aa with 27 gaps, 3 stops, absolute frame= Minus1 at 5719186..5719823 in the same strand
CDS YP_001519918.1 mechanosensitive ion channel intersects 10.16%(36aa) with IS1_ORF1 e-value= 4.6e-14 fragment hit coverage= 53.97%, between model(239 aa) positions 111; 239 length is 126aa with 7 gaps, 0 stops, absolute frame= Minus1 at 5719195..5719571 in the opposite strand
gene no id fragment; similar to N-terminus of transposase intersects 0.32%(0aa) with IS1_ORF1 e-value= 4.6e-14 fragment hit coverage= 53.97%, between model(239 aa) positions 111; 239 length is 126aa with 7 gaps, 0 stops, absolute frame= Minus1 at 5719195..5719571 in the same strand
Class A

194.
gene no id fragment; similar to N-terminus of transposase intersects 67.96%(70aa) with IS1_ORF1 e-value= 3.6e-14 fragment hit coverage= 32.64%, between model(239 aa) positions 1; 78 length is 75aa with 3 gaps, 0 stops, absolute frame= Minus3 at 5719670..5719893 in the same strand
Class A

195.
CDS YP_001519930.1 TetR family transcriptional regulator intersects 14.05%(36aa) with IS1_ORF1 e-value= 4.1e-06 fragment hit coverage= 16.74%, between model(239 aa) positions 1; 40 length is 37aa with 3 gaps, 0 stops, absolute frame= Minus1 at 5728174..5728283 in the same strand
misc_feature no id Transposase and inactivated derivatives [DNA replication, recombination, and repair]; Region: COG3677 intersects 87.30%(36aa) with IS1_ORF1 e-value= 4.1e-06 fragment hit coverage= 16.74%, between model(239 aa) positions 1; 40 length is 37aa with 3 gaps, 0 stops, absolute frame= Minus1 at 5728174..5728283 in the same strand
Class A

196.
CDS YP_001519945.1 transposase intersects 85.96%(375aa) with IS4 e-value= 9.9e-07 complete sequence hit coverage= 100%, between model(486 aa) positions 1; 486 length is 376aa with 117 gaps, 0 stops, absolute frame= Minus3 at 5744048..5745174 in the same strand
misc_feature no id DDE superfamily endonuclease; Region: DDE_5; cl02413 intersects 93.48%(348aa) with IS4 e-value= 9.9e-07 complete sequence hit coverage= 100%, between model(486 aa) positions 1; 486 length is 376aa with 117 gaps, 0 stops, absolute frame= Minus3 at 5744048..5745174 in the same strand

CDS YP_001519945.1 transposase intersects 53.01%(231aa) with IS4 e-value= 4.7e-14 fragment hit coverage= 51.23%, between model(486 aa) positions 1; 249 length is 232aa with 27 gaps, 0 stops, absolute frame= Minus3 at 5744480..5745174 in the same strand
misc_feature no id DDE superfamily endonuclease; Region: DDE_5; cl02413 intersects 62.11%(231aa) with IS4 e-value= 4.7e-14 fragment hit coverage= 51.23%, between model(486 aa) positions 1; 249 length is 232aa with 27 gaps, 0 stops, absolute frame= Minus3 at 5744480..5745174 in the same strand
Class A

197.
CDS YP_001519946.1 hypothetical protein intersects 88.43%(323aa) with IS630 e-value= 9.5e-24 fragment hit coverage= 94.15%, between model(376 aa) positions 23; 376 length is 324aa with 34 gaps, 0 stops, absolute frame= Minus2 at 5745369..5746339 in the same strand
misc_feature no id Integrase core domain; Region: rve; cl01316 intersects 100%(141aa) with IS630 e-value= 9.5e-24 fragment hit coverage= 94.15%, between model(376 aa) positions 23; 376 length is 324aa with 34 gaps, 0 stops, absolute frame= Minus2 at 5745369..5746339 in the same strand
misc_feature no id Winged helix-turn helix; Region: HTH_29; pfam13551 intersects 100%(102aa) with IS630 e-value= 9.5e-24 fragment hit coverage= 94.15%, between model(376 aa) positions 23; 376 length is 324aa with 34 gaps, 0 stops, absolute frame= Minus2 at 5745369..5746339 in the same strand
misc_feature no id Helix-turn-helix domains; Region: HTH; cl00088 intersects 100%(50aa) with IS630 e-value= 9.5e-24 fragment hit coverage= 94.15%, between model(376 aa) positions 23; 376 length is 324aa with 34 gaps, 0 stops, absolute frame= Minus2 at 5745369..5746339 in the same strand
CDS YP_001519946.1 hypothetical protein intersects 94.44%(345aa) with IS630 e-value= 4e-22 complete sequence hit coverage= 100%, between model(376 aa) positions 1; 376 length is 346aa with 34 gaps, 0 stops, absolute frame= Minus2 at 5745369..5746405 in the same strand
misc_feature no id Integrase core domain; Region: rve; cl01316 intersects 100%(141aa) with IS630 e-value= 4e-22 complete sequence hit coverage= 100%, between model(376 aa) positions 1; 376 length is 346aa with 34 gaps, 0 stops, absolute frame= Minus2 at 5745369..5746405 in the same strand
misc_feature no id Winged helix-turn helix; Region: HTH_29; pfam13551 intersects 100%(102aa) with IS630 e-value= 4e-22 complete sequence hit coverage= 100%, between model(376 aa) positions 1; 376 length is 346aa with 34 gaps, 0 stops, absolute frame= Minus2 at 5745369..5746405 in the same strand
misc_feature no id Helix-turn-helix domains; Region: HTH; cl00088 intersects 100%(50aa) with IS630 e-value= 4e-22 complete sequence hit coverage= 100%, between model(376 aa) positions 1; 376 length is 346aa with 34 gaps, 0 stops, absolute frame= Minus2 at 5745369..5746405 in the same strand
CDS YP_001519946.1 hypothetical protein intersects 14.39%(52aa) with IS481 e-value= 1.1e-08 fragment hit coverage= 15.10%, between model(351 aa) positions 1; 53 length is 53aa with 0 gaps, 0 stops, absolute frame= Minus2 at 5746188..5746345 in the same strand
misc_feature no id Winged helix-turn helix; Region: HTH_29; pfam13551 intersects 43.14%(44aa) with IS481 e-value= 1.1e-08 fragment hit coverage= 15.10%, between model(351 aa) positions 1; 53 length is 53aa with 0 gaps, 0 stops, absolute frame= Minus2 at 5746188..5746345 in the same strand
misc_feature no id Helix-turn-helix domains; Region: HTH; cl00088 intersects 100%(50aa) with IS481 e-value= 1.1e-08 fragment hit coverage= 15.10%, between model(351 aa) positions 1; 53 length is 53aa with 0 gaps, 0 stops, absolute frame= Minus2 at 5746188..5746345 in the same strand
Class A

198.
misc_feature no id Transposase and inactivated derivatives [DNA replication, recombination, and repair]; Region: COG3677 intersects 83.81%(88aa) with IS1_ORF1 e-value= 1.7e-15 fragment hit coverage= 36.40%, between model(239 aa) positions 1; 87 length is 88aa with 2 gaps, 0 stops, absolute frame= Plus2 at 5747213..5747476 in the same strand
CDS YP_001519948.1 hypothetical protein intersects 67.18%(88aa) with IS1_ORF1 e-value= 1.7e-15 fragment hit coverage= 36.40%, between model(239 aa) positions 1; 87 length is 88aa with 2 gaps, 0 stops, absolute frame= Plus2 at 5747213..5747476 in the same strand
Class A

199.

CDS YP_001519949.1 hypothetical protein intersects 27.54%(46aa) with IS1_ORF2 e-value= 1.8e-11 fragment hit coverage= 20.68%, between model(237 aa) positions 164; 212 length is 46aa with 3 gaps, 0 stops, absolute frame= Plus1 at 5747743..5747880 in the same strand
misc_feature no id IS1 transposase; Region: DDE_Tnp_IS1; cl00721 intersects 49.46%(46aa) with IS1_ORF2 e-value= 1.8e-11 fragment hit coverage= 20.68%, between model(237 aa) positions 164; 212 length is 46aa with 3 gaps, 0 stops, absolute frame= Plus1 at 5747743..5747880 in the same strand
Class A

200.
CDS YP_001520080.1 IS5 family transposase intersects 15.34%(81aa) with IS5_IS5 e-value= 2.6e-06 fragment hit coverage= 19.91%, between model(422 aa) positions 23; 106 length is 81aa with 3 gaps, 0 stops, absolute frame= Plus2 at 5896091..5896333 in the same strand
misc_feature no id Transposase domain (DUF772); Region: DUF772; pfam05598 intersects 74.03%(57aa) with IS5_IS5 e-value= 2.6e-06 fragment hit coverage= 19.91%, between model(422 aa) positions 23; 106 length is 81aa with 3 gaps, 0 stops, absolute frame= Plus2 at 5896091..5896333 in the same strand
Class A

201.
gene no id fragment; similar to N-terminus of transposase intersects 29.71%(27aa) with IS630 e-value= 4e-13 fragment hit coverage= 50.80%, between model(376 aa) positions 140; 330 length is 165aa with 28 gaps, 0 stops, absolute frame= Plus1 at 5903662..5904156 in the same strand
gene no id fragment; similar to C-terminus of transposase intersects 72.38%(76aa) with IS630 e-value= 4e-13 fragment hit coverage= 50.80%, between model(376 aa) positions 140; 330 length is 165aa with 28 gaps, 0 stops, absolute frame= Plus1 at 5903662..5904156 in the same strand
Class A

202.
CDS YP_001520095.1 hypothetical protein intersects 86.62%(136aa) with IS630 e-value= 8.7e-15 fragment hit coverage= 39.63%, between model(376 aa) positions 1; 149 length is 136aa with 13 gaps, 0 stops, absolute frame= Plus3 at 5908257..5908664 in the same strand
misc_feature no id Helix-turn-helix domains; Region: HTH; cl00088 intersects 100%(50aa) with IS630 e-value= 8.7e-15 fragment hit coverage= 39.63%, between model(376 aa) positions 1; 149 length is 136aa with 13 gaps, 0 stops, absolute frame= Plus3 at 5908257..5908664 in the same strand
misc_feature no id Winged helix-turn helix; Region: HTH_29; pfam13551 intersects 100%(102aa) with IS630 e-value= 8.7e-15 fragment hit coverage= 39.63%, between model(376 aa) positions 1; 149 length is 136aa with 13 gaps, 0 stops, absolute frame= Plus3 at 5908257..5908664 in the same strand
misc_feature no id Helix-turn-helix domains; Region: HTH; cl00088 intersects 100%(72aa) with IS630 e-value= 8.7e-15 fragment hit coverage= 39.63%, between model(376 aa) positions 1; 149 length is 136aa with 13 gaps, 0 stops, absolute frame= Plus3 at 5908257..5908664 in the same strand
CDS YP_001520095.1 hypothetical protein intersects 71.97%(113aa) with IS481 e-value= 3.5e-09 fragment hit coverage= 33.62%, between model(351 aa) positions 1; 118 length is 113aa with 6 gaps, 0 stops, absolute frame= Plus3 at 5908317..5908655 in the same strand
misc_feature no id Helix-turn-helix domains; Region: HTH; cl00088 intersects 100%(50aa) with IS481 e-value= 3.5e-09 fragment hit coverage= 33.62%, between model(351 aa) positions 1; 118 length is 113aa with 6 gaps, 0 stops, absolute frame= Plus3 at 5908317..5908655 in the same strand
misc_feature no id Winged helix-turn helix; Region: HTH_29; pfam13551 intersects 100%(102aa) with IS481 e-value= 3.5e-09 fragment hit coverage= 33.62%, between model(351 aa) positions 1; 118 length is 113aa with 6 gaps, 0 stops, absolute frame= Plus3 at 5908317..5908655 in the same strand
misc_feature no id Helix-turn-helix domains; Region: HTH; cl00088 intersects 100%(72aa) with IS481 e-value= 3.5e-09 fragment hit coverage= 33.62%, between model(351 aa) positions 1; 118 length is 113aa with 6 gaps, 0 stops, absolute frame= Plus3 at 5908317..5908655 in the same strand
Class B

203.
CDS YP_001520096.1 hypothetical protein intersects 80%(128aa) with IS630 e-value= 6.5e-11 fragment hit coverage= 39.10%, between model(376 aa) positions 187; 333 length is 130aa with 21 gaps, 0 stops, absolute frame= Plus2 at 5908769..5909158 in the same strand

misc_feature no id Integrase core domain; Region: rve; cl01316 intersects 100%(128aa) with IS630 e-value= 6.5e-11 fragment hit coverage= 39.10%, between model(376 aa) positions 187; 333 length is 130aa with 21 gaps, 0 stops, absolute frame= Plus2 at 5908769..5909158 in the same strand
Class A

204.
misc_feature no id Transposase and inactivated derivatives [DNA replication, recombination, and repair]; Region: COG3464 intersects 99.01%(399aa) with ISL3 e-value= 1.8e-43 complete sequence hit coverage= 100%, between model(451 aa) positions 1; 451 length is 413aa with 44 gaps, 1 stops, absolute frame= Plus3 at 5916063..5917301 in the same strand
CDS YP_001520108.1 transposase intersects 99.04%(411aa) with ISL3 e-value= 1.8e-43 complete sequence hit coverage= 100%, between model(451 aa) positions 1; 451 length is 413aa with 44 gaps, 1 stops, absolute frame= Plus3 at 5916063..5917301 in the same strand
misc_feature no id Transposase; Region: DDE_Tnp_ISL3; pfam01610 intersects 100%(246aa) with ISL3 e-value= 1.8e-43 complete sequence hit coverage= 100%, between model(451 aa) positions 1; 451 length is 413aa with 44 gaps, 1 stops, absolute frame= Plus3 at 5916063..5917301 in the same strand
misc_feature no id Transposase and inactivated derivatives [DNA replication, recombination, and repair]; Region: COG3464 intersects 85.86%(346aa) with ISL3 e-value= 1e-49 fragment hit coverage= 86.03%, between model(451 aa) positions 64; 451 length is 360aa with 34 gaps, 1 stops, absolute frame= Plus3 at 5916222..5917301 in the same strand
CDS YP_001520108.1 transposase intersects 86.27%(358aa) with ISL3 e-value= 1e-49 fragment hit coverage= 86.03%, between model(451 aa) positions 64; 451 length is 360aa with 34 gaps, 1 stops, absolute frame= Plus3 at 5916222..5917301 in the same strand
misc_feature no id Transposase; Region: DDE_Tnp_ISL3; pfam01610 intersects 100%(246aa) with ISL3 e-value= 1e-49 fragment hit coverage= 86.03%, between model(451 aa) positions 64; 451 length is 360aa with 34 gaps, 1 stops, absolute frame= Plus3 at 5916222..5917301 in the same strand
Class A

205.
CDS YP_001520122.1 hypothetical protein intersects 92.31%(36aa) with IS6 e-value= 6.2e-80 complete sequence hit coverage= 100%, between model(235 aa) positions 1; 235 length is 230aa with 5 gaps, 3 stops, absolute frame= Minus2 at 5927166..5927854 in the same strand
CDS YP_001520123.1 transposase intersects 96.08%(81aa) with IS6 e-value= 6.2e-80 complete sequence hit coverage= 100%, between model(235 aa) positions 1; 235 length is 230aa with 5 gaps, 3 stops, absolute frame= Minus2 at 5927166..5927854 in the same strand
misc_feature no id Transposase and inactivated derivatives [DNA replication, recombination, and repair]; Region: COG3316 intersects 100%(78aa) with IS6 e-value= 6.2e-80 complete sequence hit coverage= 100%, between model(235 aa) positions 1; 235 length is 230aa with 5 gaps, 3 stops, absolute frame= Minus2 at 5927166..5927854 in the same strand
Class A

206.
gene no id fragment; similar to N-terminus of transposase intersects 89.10%(139aa) with IS5_ISL2 e-value= 1.8e-23 complete sequence hit coverage= 100%, between model(260 aa) positions 1; 260 length is 222aa with 39 gaps, 4 stops, absolute frame= Plus3 at 5942076..5942741 in the same strand
gene no id fragment; similar to C-terminus of transposase intersects 58.15%(52aa) with IS5_ISL2 e-value= 1.8e-23 complete sequence hit coverage= 100%, between model(260 aa) positions 1; 260 length is 222aa with 39 gaps, 4 stops, absolute frame= Plus3 at 5942076..5942741 in the same strand
gene no id fragment; similar to N-terminus of transposase intersects 80.34%(125aa) with IS5_ISL2 e-value= 1e-38 complete sequence hit coverage= 100%, between model(260 aa) positions 1; 260 length is 241aa with 25 gaps, 6 stops, absolute frame= Plus2 at 5942117..5942839 in the same strand
gene no id fragment; similar to C-terminus of transposase intersects 94.44%(85aa) with IS5_ISL2 e-value= 1e-38 complete sequence hit coverage= 100%, between model(260 aa) positions 1; 260 length is 241aa with 25 gaps, 6 stops, absolute frame= Plus2 at 5942117..5942839 in the same strand
gene no id fragment; similar to N-terminus of transposase intersects 66.67%(104aa) with IS5_ISL2 e-value= 5e-44 fragment hit coverage= 40%, between model(260 aa) positions 26; 129 length is 104aa with 0 gaps, 0 stops, absolute frame= Plus3 at 5942136..5942447 in the same strand

gene no id fragment; similar to N-terminus of transposase intersects 11.75%(18aa) with IS5_ISL2 e-value= 2.1e-57 fragment hit coverage= 51.54%, between model(260 aa) positions 127; 260 length is 134aa with 0 gaps, 0 stops, absolute frame= Plus2 at 5942438..5942839 in the same strand
gene no id fragment; similar to C-terminus of transposase intersects 94.44%(85aa) with IS5_ISL2 e-value= 2.1e-57 fragment hit coverage= 51.54%, between model(260 aa) positions 127; 260 length is 134aa with 0 gaps, 0 stops, absolute frame= Plus2 at 5942438..5942839 in the same strand
Class A

207.
CDS YP_001520183.1 hypothetical protein intersects 93.51%(346aa) with IS630 e-value= 4.9e-24 complete sequence hit coverage= 100%, between model(376 aa) positions 1; 376 length is 346aa with 33 gaps, 0 stops, absolute frame= Plus3 at 5992275..5993312 in the same strand
misc_feature no id Helix-turn-helix domains; Region: HTH; cl00088 intersects 100%(50aa) with IS630 e-value= 4.9e-24 complete sequence hit coverage= 100%, between model(376 aa) positions 1; 376 length is 346aa with 33 gaps, 0 stops, absolute frame= Plus3 at 5992275..5993312 in the same strand
misc_feature no id Winged helix-turn helix; Region: HTH_29; pfam13551 intersects 100%(102aa) with IS630 e-value= 4.9e-24 complete sequence hit coverage= 100%, between model(376 aa) positions 1; 376 length is 346aa with 33 gaps, 0 stops, absolute frame= Plus3 at 5992275..5993312 in the same strand
misc_feature no id Winged helix-turn helix; Region: HTH_33; pfam13592 intersects 100%(56aa) with IS630 e-value= 4.9e-24 complete sequence hit coverage= 100%, between model(376 aa) positions 1; 376 length is 346aa with 33 gaps, 0 stops, absolute frame= Plus3 at 5992275..5993312 in the same strand
misc_feature no id Integrase core domain; Region: rve; cl01316 intersects 100%(141aa) with IS630 e-value= 4.9e-24 complete sequence hit coverage= 100%, between model(376 aa) positions 1; 376 length is 346aa with 33 gaps, 0 stops, absolute frame= Plus3 at 5992275..5993312 in the same strand
CDS YP_001520183.1 hypothetical protein intersects 14.32%(53aa) with IS481 e-value= 3.7e-09 fragment hit coverage= 15.10%, between model(351 aa) positions 1; 53 length is 53aa with 0 gaps, 0 stops, absolute frame= Plus3 at 5992335..5992493 in the same strand
misc_feature no id Helix-turn-helix domains; Region: HTH; cl00088 intersects 100%(50aa) with IS481 e-value= 3.7e-09 fragment hit coverage= 15.10%, between model(351 aa) positions 1; 53 length is 53aa with 0 gaps, 0 stops, absolute frame= Plus3 at 5992335..5992493 in the same strand
misc_feature no id Winged helix-turn helix; Region: HTH_29; pfam13551 intersects 43.14%(44aa) with IS481 e-value= 3.7e-09 fragment hit coverage= 15.10%, between model(351 aa) positions 1; 53 length is 53aa with 0 gaps, 0 stops, absolute frame= Plus3 at 5992335..5992493 in the same strand
Class A

208.
gene no id fragment; similar to C-terminus of transposase intersects 30.46%(70aa) with IS630 e-value= 4e-10 fragment hit coverage= 19.15%, between model(376 aa) positions 305; 376 length is 73aa with 0 gaps, 1 stops, absolute frame= Minus2 at 6013572..6013789 in the same strand
gene no id fragment; similar to C-terminus of transposase intersects 15.80%(36aa) with IS3_IS150_ORF2 e-value= 2e-08 fragment hit coverage= 10.03%, between model(389 aa) positions 329; 367 length is 37aa with 2 gaps, 0 stops, absolute frame= Minus2 at 6013632..6013741 in the same strand
Class A

209.
gene no id fragment; similar to N-terminus of transposase intersects 36.69%(65aa) with IS630 e-value= 4e-07 fragment hit coverage= 17.55%, between model(376 aa) positions 1; 66 length is 66aa with 0 gaps, 0 stops, absolute frame= Minus1 at 6014614..6014810 in the same strand
Class A

210.
CDS YP_001520233.1 IS1 transposase intersects 96.52%(222aa) with IS1_ORF2 e-value= 3.9e-29 complete sequence hit coverage= 100%, between model(237 aa) positions 1; 237 length is 227aa with 14 gaps, 1 stops, absolute frame= Plus1 at 6055453..6056133 in the same strand
misc_feature no id Transposase and inactivated derivatives [DNA replication, recombination, and repair]; Region: COG3677 intersects 100%(81aa) with IS1_ORF2 e-value= 3.9e-29 complete sequence hit coverage= 100%,

between model(237 aa) positions 1; 237 length is 227aa with 14 gaps, 1 stops, absolute frame= Plus1 at 6055453..6056133 in the same strand
misc_feature no id IS1 transposase; Region: DDE_Tnp_IS1; cl00721 intersects 100%(120aa) with IS1_ORF2 e-value= 3.9e-29 complete sequence hit coverage= 100%, between model(237 aa) positions 1; 237 length is 227aa with 14 gaps, 1 stops, absolute frame= Plus1 at 6055453..6056133 in the same strand
CDS YP_001520233.1 IS1 transposase intersects 95.65%(220aa) with IS1_ORF1 e-value= 3.3e-41 complete sequence hit coverage= 100%, between model(239 aa) positions 1; 239 length is 225aa with 19 gaps, 1 stops, absolute frame= Plus1 at 6055453..6056127 in the same strand
misc_feature no id Transposase and inactivated derivatives [DNA replication, recombination, and repair]; Region: COG3677 intersects 100%(81aa) with IS1_ORF1 e-value= 3.3e-41 complete sequence hit coverage= 100%, between model(239 aa) positions 1; 239 length is 225aa with 19 gaps, 1 stops, absolute frame= Plus1 at 6055453..6056127 in the same strand
misc_feature no id IS1 transposase; Region: DDE_Tnp_IS1; cl00721 intersects 100%(120aa) with IS1_ORF1 e-value= 3.3e-41 complete sequence hit coverage= 100%, between model(239 aa) positions 1; 239 length is 225aa with 19 gaps, 1 stops, absolute frame= Plus1 at 6055453..6056127 in the same strand
CDS YP_001520233.1 IS1 transposase intersects 58.26%(134aa) with IS1_ORF2 e-value= 8.3e-32 fragment hit coverage= 59.07%, between model(237 aa) positions 98; 237 length is 134aa with 6 gaps, 0 stops, absolute frame= Plus1 at 6055732..6056133 in the same strand
misc_feature no id IS1 transposase; Region: DDE_Tnp_IS1; cl00721 intersects 100%(120aa) with IS1_ORF2 e-value= 8.3e-32 fragment hit coverage= 59.07%, between model(237 aa) positions 98; 237 length is 134aa with 6 gaps, 0 stops, absolute frame= Plus1 at 6055732..6056133 in the same strand
Class A

211.
gene no id fragment; similar to C-terminus of transposase intersects 78.68%(107aa) with IS630 e-value= 3e-16 fragment hit coverage= 50.80%, between model(376 aa) positions 140; 330 length is 165aa with 28 gaps, 0 stops, absolute frame= Minus1 at 6059137..6059630 in the same strand
gene no id fragment; similar to N-terminus of transposase intersects 21.09%(27aa) with IS630 e-value= 3e-16 fragment hit coverage= 50.80%, between model(376 aa) positions 140; 330 length is 165aa with 28 gaps, 0 stops, absolute frame= Minus1 at 6059137..6059630 in the same strand
Class A

212.
gene no id fragment; similar to N-terminus of transposase intersects 78%(114aa) with IS630 e-value= 1.2e-15 fragment hit coverage= 32.18%, between model(376 aa) positions 1; 121 length is 115aa with 7 gaps, 0 stops, absolute frame= Minus3 at 6061148..6061491 in the same strand
Class A

213.
CDS YP_001520271.1 hypothetical protein intersects 47.43%(129aa) with IS630 e-value= 6.9e-14 fragment hit coverage= 38.83%, between model(376 aa) positions 188; 333 length is 129aa with 20 gaps, 0 stops, absolute frame= Plus2 at 6104879..6105265 in the same strand
misc_feature no id Integrase core domain; Region: rve; cl01316 intersects 90.21%(129aa) with IS630 e-value= 6.9e-14 fragment hit coverage= 38.83%, between model(376 aa) positions 188; 333 length is 129aa with 20 gaps, 0 stops, absolute frame= Plus2 at 6104879..6105265 in the same strand
Class A

214.
misc_feature no id Transposase and inactivated derivatives [DNA replication, recombination, and repair]; Region: COG3677 intersects 83.81%(88aa) with IS1_ORF1 e-value= 1.7e-15 fragment hit coverage= 36.40%, between model(239 aa) positions 1; 87 length is 88aa with 2 gaps, 0 stops, absolute frame= Plus1 at 6108877..6109140 in the same strand
CDS YP_001520276.1 hypothetical protein intersects 67.18%(88aa) with IS1_ORF1 e-value= 1.7e-15 fragment hit coverage= 36.40%, between model(239 aa) positions 1; 87 length is 88aa with 2 gaps, 0 stops, absolute frame= Plus1 at 6108877..6109140 in the same strand
Class A

215.
CDS YP_001520277.1 hypothetical protein intersects 27.54%(46aa) with IS1_ORF2 e-value= 1.8e-11 fragment hit coverage= 20.68%, between model(237 aa) positions 164; 212 length is 46aa with 3 gaps, 0 stops, absolute frame= Plus3 at 6109407..6109544 in the same strand
misc_feature no id IS1 transposase; Region: DDE_Tnp_IS1; cl00721 intersects 49.46%(46aa) with IS1_ORF2 e-value= 1.8e-11 fragment hit coverage= 20.68%, between model(237 aa) positions 164; 212 length is 46aa with 3 gaps, 0 stops, absolute frame= Plus3 at 6109407..6109544 in the same strand
Class A

216.
CDS YP_001520288.1 IS116/IS110/IS902 family transposase intersects 98.32%(311aa) with IS110 e-value= 2.2e-68 complete sequence hit coverage= 100%, between model(416 aa) positions 1; 416 length is 312aa with 104 gaps, 1 stops, absolute frame= Minus2 at 6119784..6120718 in the same strand
misc_feature no id Transposase and inactivated derivatives [DNA replication, recombination, and repair]; Region: COG3547 intersects 99.89%(292aa) with IS110 e-value= 2.2e-68 complete sequence hit coverage= 100%, between model(416 aa) positions 1; 416 length is 312aa with 104 gaps, 1 stops, absolute frame= Minus2 at 6119784..6120718 in the same strand
misc_feature no id Transposase IS116/IS110/IS902 family; Region: Transposase_20; pfam02371 intersects 100%(57aa) with IS110 e-value= 2.2e-68 complete sequence hit coverage= 100%, between model(416 aa) positions 1; 416 length is 312aa with 104 gaps, 1 stops, absolute frame= Minus2 at 6119784..6120718 in the same strand
misc_feature no id Transposase; Region: DEDD_Tnp_IS110; pfam01548 intersects 100%(140aa) with IS110 e-value= 2.2e-68 complete sequence hit coverage= 100%, between model(416 aa) positions 1; 416 length is 312aa with 104 gaps, 1 stops, absolute frame= Minus2 at 6119784..6120718 in the same strand
Class A

217.
CDS YP_001520328.1 IS5 family transposase intersects 15.28%(80aa) with IS5_IS5 e-value= 2.6e-06 fragment hit coverage= 19.91%, between model(422 aa) positions 23; 106 length is 81aa with 3 gaps, 0 stops, absolute frame= Minus2 at 6167601..6167842 in the same strand
misc_feature no id Transposase domain (DUF772); Region: DUF772; pfam05598 intersects 74.03%(57aa) with IS5_IS5 e-value= 2.6e-06 fragment hit coverage= 19.91%, between model(422 aa) positions 23; 106 length is 81aa with 3 gaps, 0 stops, absolute frame= Minus2 at 6167601..6167842 in the same strand
Class A

218.
CDS YP_001520371.1 transposase intersects 98.86%(551aa) with ISL3 e-value= 9.2e-10 complete sequence hit coverage= 100%, between model(451 aa) positions 1; 451 length is 552aa with 73 gaps, 0 stops, absolute frame= Minus3 at 6203885..6205539 in the same strand
misc_feature no id Transposase; Region: DDE_Tnp_ISL3; pfam01610 intersects 100%(121aa) with ISL3 e-value= 9.2e-10 complete sequence hit coverage= 100%, between model(451 aa) positions 1; 451 length is 552aa with 73 gaps, 0 stops, absolute frame= Minus3 at 6203885..6205539 in the same strand
misc_feature no id Helix-turn-helix domains; Region: HTH; cl00088 intersects 100%(42aa) with ISL3 e-value= 9.2e-10 complete sequence hit coverage= 100%, between model(451 aa) positions 1; 451 length is 552aa with 73 gaps, 0 stops, absolute frame= Minus3 at 6203885..6205539 in the same strand
misc_feature no id Transposase; Region: DDE_Tnp_ISL3; pfam01610 intersects 100%(90aa) with ISL3 e-value= 9.2e-10 complete sequence hit coverage= 100%, between model(451 aa) positions 1; 451 length is 552aa with 73 gaps, 0 stops, absolute frame= Minus3 at 6203885..6205539 in the same strand
misc_feature no id Transposase and inactivated derivatives [DNA replication, recombination, and repair]; Region: COG3464 intersects 100%(198aa) with ISL3 e-value= 9.2e-10 complete sequence hit coverage= 100%, between model(451 aa) positions 1; 451 length is 552aa with 73 gaps, 0 stops, absolute frame= Minus3 at 6203885..6205539 in the same strand
CDS YP_001520372.1 hypothetical protein intersects 60.14%(27aa) with ISL3 e-value= 9.2e-10 complete sequence hit coverage= 100%, between model(451 aa) positions 1; 451 length is 552aa with 73 gaps, 0 stops, absolute frame= Minus3 at 6203885..6205539 in the opposite strand
CDS YP_001520371.1 transposase intersects 16.07%(89aa) with ISL3 e-value= 1.6e-06 fragment hit coverage= 20.84%, between model(451 aa) positions 331; 424 length is 90aa with 4 gaps, 0 stops, absolute frame= Minus3 at 6203924..6204192 in the same strand

misc_feature no id Transposase; Region: DDE_Tnp_ISL3; pfam01610 intersects 74.10%(89aa) with ISL3 e-value= 1.6e-06 fragment hit coverage= 20.84%, between model(451 aa) positions 331; 424 length is 90aa with 4 gaps, 0 stops, absolute frame= Minus3 at 6203924..6204192 in the same strand
CDS YP_001520371.1 transposase intersects 17.86%(99aa) with IS21_ORF1 e-value= 2.7e-14 fragment hit coverage= 21.90%, between model(525 aa) positions 1; 115 length is 100aa with 15 gaps, 0 stops, absolute frame= Minus3 at 6204359..6204657 in the same strand
misc_feature no id Helix-turn-helix domains; Region: HTH; cl00088 intersects 92.06%(38aa) with IS21_ORF1 e-value= 2.7e-14 fragment hit coverage= 21.90%, between model(525 aa) positions 1; 115 length is 100aa with 15 gaps, 0 stops, absolute frame= Minus3 at 6204359..6204657 in the same strand
CDS YP_001520371.1 transposase intersects 15.35%(85aa) with ISL3 e-value= 1.5e-21 fragment hit coverage= 21.73%, between model(451 aa) positions 156; 253 length is 86aa with 12 gaps, 0 stops, absolute frame= Minus3 at 6204836..6205092 in the same strand
misc_feature no id Transposase; Region: DDE_Tnp_ISL3; pfam01610 intersects 90%(81aa) with ISL3 e-value= 1.5e-21 fragment hit coverage= 21.73%, between model(451 aa) positions 156; 253 length is 86aa with 12 gaps, 0 stops, absolute frame= Minus3 at 6204836..6205092 in the same strand
misc_feature no id Transposase and inactivated derivatives [DNA replication, recombination, and repair]; Region: COG3464 intersects 43.27%(85aa) with ISL3 e-value= 1.5e-21 fragment hit coverage= 21.73%, between model(451 aa) positions 156; 253 length is 86aa with 12 gaps, 0 stops, absolute frame= Minus3 at 6204836..6205092 in the same strand
Class A

219.
CDS YP_001520373.1 transposase intersects 98.86%(551aa) with ISL3 e-value= 9.2e-10 complete sequence hit coverage= 100%, between model(451 aa) positions 1; 451 length is 552aa with 73 gaps, 0 stops, absolute frame= Minus3 at 6205703..6207357 in the same strand
misc_feature no id Transposase; Region: DDE_Tnp_ISL3; pfam01610 intersects 100%(121aa) with ISL3 e-value= 9.2e-10 complete sequence hit coverage= 100%, between model(451 aa) positions 1; 451 length is 552aa with 73 gaps, 0 stops, absolute frame= Minus3 at 6205703..6207357 in the same strand
misc_feature no id Helix-turn-helix domains; Region: HTH; cl00088 intersects 100%(42aa) with ISL3 e-value= 9.2e-10 complete sequence hit coverage= 100%, between model(451 aa) positions 1; 451 length is 552aa with 73 gaps, 0 stops, absolute frame= Minus3 at 6205703..6207357 in the same strand
misc_feature no id Transposase; Region: DDE_Tnp_ISL3; pfam01610 intersects 100%(90aa) with ISL3 e-value= 9.2e-10 complete sequence hit coverage= 100%, between model(451 aa) positions 1; 451 length is 552aa with 73 gaps, 0 stops, absolute frame= Minus3 at 6205703..6207357 in the same strand
misc_feature no id Transposase and inactivated derivatives [DNA replication, recombination, and repair]; Region: COG3464 intersects 100%(198aa) with ISL3 e-value= 9.2e-10 complete sequence hit coverage= 100%, between model(451 aa) positions 1; 451 length is 552aa with 73 gaps, 0 stops, absolute frame= Minus3 at 6205703..6207357 in the same strand
CDS YP_001520374.1 hypothetical protein intersects 60.14%(27aa) with ISL3 e-value= 9.2e-10 complete sequence hit coverage= 100%, between model(451 aa) positions 1; 451 length is 552aa with 73 gaps, 0 stops, absolute frame= Minus3 at 6205703..6207357 in the opposite strand
CDS YP_001520373.1 transposase intersects 16.07%(89aa) with ISL3 e-value= 1.6e-06 fragment hit coverage= 20.84%, between model(451 aa) positions 331; 424 length is 90aa with 4 gaps, 0 stops, absolute frame= Minus3 at 6205742..6206010 in the same strand
misc_feature no id Transposase; Region: DDE_Tnp_ISL3; pfam01610 intersects 74.10%(89aa) with ISL3 e-value= 1.6e-06 fragment hit coverage= 20.84%, between model(451 aa) positions 331; 424 length is 90aa with 4 gaps, 0 stops, absolute frame= Minus3 at 6205742..6206010 in the same strand
CDS YP_001520373.1 transposase intersects 17.86%(99aa) with IS21_ORF1 e-value= 2.7e-14 fragment hit coverage= 21.90%, between model(525 aa) positions 1; 115 length is 100aa with 15 gaps, 0 stops, absolute frame= Minus3 at 6206177..6206475 in the same strand
misc_feature no id Helix-turn-helix domains; Region: HTH; cl00088 intersects 92.06%(38aa) with IS21_ORF1 e-value= 2.7e-14 fragment hit coverage= 21.90%, between model(525 aa) positions 1; 115 length is 100aa with 15 gaps, 0 stops, absolute frame= Minus3 at 6206177..6206475 in the same strand
CDS YP_001520373.1 transposase intersects 15.35%(85aa) with ISL3 e-value= 1.5e-21 fragment hit coverage= 21.73%, between model(451 aa) positions 156; 253 length is 86aa with 12 gaps, 0 stops, absolute frame= Minus3 at 6206654..6206910 in the same strand

misc_feature	no id	Transposase; Region: DDE_Tnp_ISL3; pfam01610 intersects 90%(81aa) with ISL3	e-value= 1.5e-21 fragment hit coverage= 21.73%, between model(451 aa) positions 156; 253 length is 86aa with 12 gaps, 0 stops, absolute frame= Minus3 at 6206654..6206910 in the same strand
misc_feature	no id	Transposase and inactivated derivatives [DNA replication, recombination, and repair]; Region: COG3464 intersects 43.27%(85aa) with ISL3	e-value= 1.5e-21 fragment hit coverage= 21.73%, between model(451 aa) positions 156; 253 length is 86aa with 12 gaps, 0 stops, absolute frame= Minus3 at 6206654..6206910 in the same strand
Class A

220.
CDS	YP_001520377.1		IS4 family transposase intersects 99.35%(308aa) with IS5_IS903	e-value= 2.2e-96 complete sequence hit coverage= 100%, between model(347 aa) positions 1; 347 length is 314aa with 37 gaps, 1 stops, absolute frame= Minus2 at 6208452..6209392 in the same strand
misc_feature	no id	Transposase DDE domain; Region: DDE_Tnp_1; pfam01609 intersects 100%(186aa) with IS5_IS903	e-value= 2.2e-96 complete sequence hit coverage= 100%, between model(347 aa) positions 1; 347 length is 314aa with 37 gaps, 1 stops, absolute frame= Minus2 at 6208452..6209392 in the same strand
misc_feature	no id	Transposase DDE domain; Region: DDE_Tnp_1_5; pfam13737 intersects 100%(112aa) with IS5_IS903	e-value= 2.2e-96 complete sequence hit coverage= 100%, between model(347 aa) positions 1; 347 length is 314aa with 37 gaps, 1 stops, absolute frame= Minus2 at 6208452..6209392 in the same strand
Class A

221.
CDS	YP_001520402.1		transposase-associated ATP-binding protein intersects 97.54%(238aa) with IS21_ORF2	e-value= 3.4e-83 complete sequence hit coverage= 100%, between model(258 aa) positions 1; 258 length is 255aa with 4 gaps, 0 stops, absolute frame= Minus2 at 6231831..6232594 in the same strand
misc_feature	no id	P-loop containing Nucleoside Triphosphate Hydrolases; Region: P-loop_NTPase; cl09099 intersects 100%(179aa) with IS21_ORF2	e-value= 3.4e-83 complete sequence hit coverage= 100%, between model(258 aa) positions 1; 258 length is 255aa with 4 gaps, 0 stops, absolute frame= Minus2 at 6231831..6232594 in the same strand
CDS	YP_001520403.1		transposase intersects 12.84%(74aa) with IS21_ORF2	e-value= 3.4e-83 complete sequence hit coverage= 100%, between model(258 aa) positions 1; 258 length is 255aa with 4 gaps, 0 stops, absolute frame= Minus2 at 6231831..6232594 in the same strand
Class A

222.
CDS	YP_001520403.1		transposase intersects 85.09%(492aa) with IS21_ORF1	e-value= 3.3e-24 complete sequence hit coverage= 100%, between model(525 aa) positions 1; 525 length is 493aa with 39 gaps, 0 stops, absolute frame= Minus1 at 6232612..6234089 in the same strand
misc_feature	no id	Integrase core domain; Region: rve; cl01316 intersects 100%(126aa) with IS21_ORF1	e-value= 3.3e-24 complete sequence hit coverage= 100%, between model(525 aa) positions 1; 525 length is 493aa with 39 gaps, 0 stops, absolute frame= Minus1 at 6232612..6234089 in the same strand
CDS	YP_001520403.1		transposase intersects 47.78%(276aa) with IS21_ORF1	e-value= 4.2e-32 fragment hit coverage= 57.90%, between model(525 aa) positions 99; 402 length is 277aa with 28 gaps, 0 stops, absolute frame= Minus1 at 6232990..6233819 in the same strand
misc_feature	no id	Integrase core domain; Region: rve; cl01316 intersects 100%(126aa) with IS21_ORF1	e-value= 4.2e-32 fragment hit coverage= 57.90%, between model(525 aa) positions 99; 402 length is 277aa with 28 gaps, 0 stops, absolute frame= Minus1 at 6232990..6233819 in the same strand
Class A

223.
CDS	YP_001520412.1		hypothetical protein intersects 80%(128aa) with IS630	e-value= 6.5e-11 fragment hit coverage= 39.10%, between model(376 aa) positions 187; 333 length is 130aa with 21 gaps, 0 stops, absolute frame= Minus2 at 6238101..6238489 in the same strand
misc_feature	no id	Integrase core domain; Region: rve; cl01316 intersects 100%(128aa) with IS630	e-value= 6.5e-11 fragment hit coverage= 39.10%, between model(376 aa) positions 187; 333 length is 130aa with 21 gaps, 0 stops, absolute frame= Minus2 at 6238101..6238489 in the same strand
Class A

224.
CDS YP_001520413.1 hypothetical protein intersects 86.41%(135aa) with IS630 e-value= 8.7e-15 fragment hit coverage= 39.63%, between model(376 aa) positions 1; 149 length is 136aa with 13 gaps, 0 stops, absolute frame= Minus3 at 6238595..6239001 in the same strand
misc_feature no id Winged helix-turn helix; Region: HTH_29; pfam13551 intersects 100%(102aa) with IS630 e-value= 8.7e-15 fragment hit coverage= 39.63%, between model(376 aa) positions 1; 149 length is 136aa with 13 gaps, 0 stops, absolute frame= Minus3 at 6238595..6239001 in the same strand
misc_feature no id Helix-turn-helix domains; Region: HTH; cl00088 intersects 100%(72aa) with IS630 e-value= 8.7e-15 fragment hit coverage= 39.63%, between model(376 aa) positions 1; 149 length is 136aa with 13 gaps, 0 stops, absolute frame= Minus3 at 6238595..6239001 in the same strand
misc_feature no id Helix-turn-helix domains; Region: HTH; cl00088 intersects 100%(50aa) with IS630 e-value= 8.7e-15 fragment hit coverage= 39.63%, between model(376 aa) positions 1; 149 length is 136aa with 13 gaps, 0 stops, absolute frame= Minus3 at 6238595..6239001 in the same strand
CDS YP_001520413.1 hypothetical protein intersects 71.76%(112aa) with IS481 e-value= 3.5e-09 fragment hit coverage= 33.62%, between model(351 aa) positions 1; 118 length is 113aa with 6 gaps, 0 stops, absolute frame= Minus3 at 6238604..6238941 in the same strand
misc_feature no id Winged helix-turn helix; Region: HTH_29; pfam13551 intersects 100%(102aa) with IS481 e-value= 3.5e-09 fragment hit coverage= 33.62%, between model(351 aa) positions 1; 118 length is 113aa with 6 gaps, 0 stops, absolute frame= Minus3 at 6238604..6238941 in the same strand
misc_feature no id Helix-turn-helix domains; Region: HTH; cl00088 intersects 100%(72aa) with IS481 e-value= 3.5e-09 fragment hit coverage= 33.62%, between model(351 aa) positions 1; 118 length is 113aa with 6 gaps, 0 stops, absolute frame= Minus3 at 6238604..6238941 in the same strand
misc_feature no id Helix-turn-helix domains; Region: HTH; cl00088 intersects 100%(50aa) with IS481 e-value= 3.5e-09 fragment hit coverage= 33.62%, between model(351 aa) positions 1; 118 length is 113aa with 6 gaps, 0 stops, absolute frame= Minus3 at 6238604..6238941 in the same strand
Class B

225.
CDS YP_001520423.1 IS5 family transposase intersects 14.59%(81aa) with IS5_IS5 e-value= 2.6e-06 fragment hit coverage= 19.91%, between model(422 aa) positions 23; 106 length is 81aa with 3 gaps, 0 stops, absolute frame= Plus2 at 6243806..6244048 in the same strand
misc_feature no id Transposase domain (DUF772); Region: DUF772; pfam05598 intersects 74.03%(57aa) with IS5_IS5 e-value= 2.6e-06 fragment hit coverage= 19.91%, between model(422 aa) positions 23; 106 length is 81aa with 3 gaps, 0 stops, absolute frame= Plus2 at 6243806..6244048 in the same strand
Class A

226.
CDS YP_001520431.1 Tn3 family transposase intersects 15.51%(156aa) with Tn3 e-value= 2.6e-14 fragment hit coverage= 15.94%, between model(1004 aa) positions 740; 899 length is 157aa with 4 gaps, 0 stops, absolute frame= Minus2 at 6249804..6250273 in the same strand
misc_feature no id Tn3 transposase DDE domain; Region: DDE_Tnp_Tn3; cl14901 intersects 40.38%(156aa) with Tn3 e-value= 2.6e-14 fragment hit coverage= 15.94%, between model(1004 aa) positions 740; 899 length is 157aa with 4 gaps, 0 stops, absolute frame= Minus2 at 6249804..6250273 in the same strand
Class A

227.
CDS YP_001520448.1 transposase intersects 99.04%(411aa) with ISL3 e-value= 1.8e-43 complete sequence hit coverage= 100%, between model(451 aa) positions 1; 451 length is 413aa with 44 gaps, 1 stops, absolute frame= Plus3 at 6263616..6264854 in the same strand
misc_feature no id Transposase and inactivated derivatives [DNA replication, recombination, and repair]; Region: COG3464 intersects 99.01%(399aa) with ISL3 e-value= 1.8e-43 complete sequence hit coverage= 100%, between model(451 aa) positions 1; 451 length is 413aa with 44 gaps, 1 stops, absolute frame= Plus3 at 6263616..6264854 in the same strand
misc_feature no id Transposase; Region: DDE_Tnp_ISL3; pfam01610 intersects 100%(246aa) with ISL3 e-value= 1.8e-43 complete sequence hit coverage= 100%, between model(451 aa) positions 1; 451 length is 413aa with 44 gaps, 1 stops, absolute frame= Plus3 at 6263616..6264854 in the same strand

CDS YP_001520448.1 transposase intersects 86.27%(358aa) with ISL3 e-value= 1e-49 fragment hit coverage= 86.03%, between model(451 aa) positions 64; 451 length is 360aa with 34 gaps, 1 stops, absolute frame= Plus3 at 6263775..6264854 in the same strand
misc_feature no id Transposase and inactivated derivatives [DNA replication, recombination, and repair]; Region: COG3464 intersects 85.86%(346aa) with ISL3 e-value= 1e-49 fragment hit coverage= 86.03%, between model(451 aa) positions 64; 451 length is 360aa with 34 gaps, 1 stops, absolute frame= Plus3 at 6263775..6264854 in the same strand
misc_feature no id Transposase; Region: DDE_Tnp_ISL3; pfam01610 intersects 100%(246aa) with ISL3 e-value= 1e-49 fragment hit coverage= 86.03%, between model(451 aa) positions 64; 451 length is 360aa with 34 gaps, 1 stops, absolute frame= Plus3 at 6263775..6264854 in the same strand
Class A

228.
CDS YP_001520464.1 transposase intersects 98.96%(410aa) with ISL3 e-value= 1.8e-43 complete sequence hit coverage= 100%, between model(451 aa) positions 1; 451 length is 413aa with 44 gaps, 1 stops, absolute frame= Minus2 at 6279558..6280795 in the same strand
misc_feature no id Transposase and inactivated derivatives [DNA replication, recombination, and repair]; Region: COG3464 intersects 98.92%(398aa) with ISL3 e-value= 1.8e-43 complete sequence hit coverage= 100%, between model(451 aa) positions 1; 451 length is 413aa with 44 gaps, 1 stops, absolute frame= Minus2 at 6279558..6280795 in the same strand
misc_feature no id Transposase; Region: DDE_Tnp_ISL3; pfam01610 intersects 100%(246aa) with ISL3 e-value= 1.8e-43 complete sequence hit coverage= 100%, between model(451 aa) positions 1; 451 length is 413aa with 44 gaps, 1 stops, absolute frame= Minus2 at 6279558..6280795 in the same strand
CDS YP_001520464.1 transposase intersects 86.18%(357aa) with ISL3 e-value= 1e-49 fragment hit coverage= 86.03%, between model(451 aa) positions 64; 451 length is 360aa with 34 gaps, 1 stops, absolute frame= Minus2 at 6279558..6280636 in the same strand
misc_feature no id Transposase and inactivated derivatives [DNA replication, recombination, and repair]; Region: COG3464 intersects 85.77%(345aa) with ISL3 e-value= 1e-49 fragment hit coverage= 86.03%, between model(451 aa) positions 64; 451 length is 360aa with 34 gaps, 1 stops, absolute frame= Minus2 at 6279558..6280636 in the same strand
misc_feature no id Transposase; Region: DDE_Tnp_ISL3; pfam01610 intersects 100%(246aa) with ISL3 e-value= 1e-49 fragment hit coverage= 86.03%, between model(451 aa) positions 64; 451 length is 360aa with 34 gaps, 1 stops, absolute frame= Minus2 at 6279558..6280636 in the same strand
Class A

229.
CDS YP_001520478.1 transposase intersects 49.06%(131aa) with Tn3 e-value= 7.4e-17 fragment hit coverage= 12.95%, between model(1004 aa) positions 764; 893 length is 131aa with 0 gaps, 0 stops, absolute frame= Plus3 at 6300402..6300794 in the same strand
misc_feature no id Tn3 transposase DDE domain; Region: DDE_Tnp_Tn3; cl14901 intersects 52.19%(131aa) with Tn3 e-value= 7.4e-17 fragment hit coverage= 12.95%, between model(1004 aa) positions 764; 893 length is 131aa with 0 gaps, 0 stops, absolute frame= Plus3 at 6300402..6300794 in the same strand
Class A

230.
CDS YP_001520488.1 transposase intersects 85.15%(493aa) with IS21_ORF1 e-value= 1.3e-21 complete sequence hit coverage= 100%, between model(525 aa) positions 1; 525 length is 493aa with 39 gaps, 0 stops, absolute frame= Plus3 at 6306396..6307874 in the same strand
misc_feature no id Integrase core domain; Region: rve; cl01316 intersects 100%(97aa) with IS21_ORF1 e-value= 1.3e-21 complete sequence hit coverage= 100%, between model(525 aa) positions 1; 525 length is 493aa with 39 gaps, 0 stops, absolute frame= Plus3 at 6306396..6307874 in the same strand
CDS YP_001520488.1 transposase intersects 47.84%(277aa) with IS21_ORF1 e-value= 1e-29 fragment hit coverage= 57.90%, between model(525 aa) positions 99; 402 length is 277aa with 28 gaps, 0 stops, absolute frame= Plus3 at 6306666..6307496 in the same strand
misc_feature no id Integrase core domain; Region: rve; cl01316 intersects 100%(97aa) with IS21_ORF1 e-value= 1e-29 fragment hit coverage= 57.90%, between model(525 aa) positions 99; 402 length is 277aa with 28 gaps, 0 stops, absolute frame= Plus3 at 6306666..6307496 in the same strand

Class A

231.
CDS YP_001520488.1 transposase intersects 12.90%(74aa) with IS21_ORF2 e-value= 1.9e-80 complete sequence hit coverage= 100%, between model(258 aa) positions 1; 258 length is 255aa with 4 gaps, 0 stops, absolute frame= Plus1 at 6307891..6308655 in the same strand
CDS YP_001520489.1 transposase-associated ATP-binding protein intersects 97.54%(238aa) with IS21_ORF2 e-value= 1.9e-80 complete sequence hit coverage= 100%, between model(258 aa) positions 1; 258 length is 255aa with 4 gaps, 0 stops, absolute frame= Plus1 at 6307891..6308655 in the same strand
misc_feature no id P-loop containing Nucleoside Triphosphate Hydrolases; Region: P-loop_NTPase; cl09099 intersects 100%(179aa) with IS21_ORF2 e-value= 1.9e-80 complete sequence hit coverage= 100%, between model(258 aa) positions 1; 258 length is 255aa with 4 gaps, 0 stops, absolute frame= Plus1 at 6307891..6308655 in the same strand
Class A

232.
CDS YP_001520491.1 transposase mutator family protein intersects 100%(375aa) with IS256 e-value= 6.6e-165 complete sequence hit coverage= 100%, between model(410 aa) positions 1; 410 length is 402aa with 11 gaps, 2 stops, absolute frame= Minus3 at 6314273..6315477 in the same strand
misc_feature no id Transposase, Mutator family; Region: Transposase_mut; pfam00872 intersects 100%(352aa) with IS256 e-value= 6.6e-165 complete sequence hit coverage= 100%, between model(410 aa) positions 1; 410 length is 402aa with 11 gaps, 2 stops, absolute frame= Minus3 at 6314273..6315477 in the same strand
misc_feature no id MULE transposase domain; Region: MULE; pfam10551 intersects 100%(78aa) with IS256 e-value= 6.6e-165 complete sequence hit coverage= 100%, between model(410 aa) positions 1; 410 length is 402aa with 11 gaps, 2 stops, absolute frame= Minus3 at 6314273..6315477 in the same strand
CDS YP_001520491.1 transposase mutator family protein intersects 100%(375aa) with IS256 e-value= 1.1e-164 fragment hit coverage= 96.59%, between model(410 aa) positions 15; 410 length is 388aa with 11 gaps, 1 stops, absolute frame= Minus3 at 6314273..6315435 in the same strand
misc_feature no id Transposase, Mutator family; Region: Transposase_mut; pfam00872 intersects 100%(352aa) with IS256 e-value= 1.1e-164 fragment hit coverage= 96.59%, between model(410 aa) positions 15; 410 length is 388aa with 11 gaps, 1 stops, absolute frame= Minus3 at 6314273..6315435 in the same strand
misc_feature no id MULE transposase domain; Region: MULE; pfam10551 intersects 100%(78aa) with IS256 e-value= 1.1e-164 fragment hit coverage= 96.59%, between model(410 aa) positions 15; 410 length is 388aa with 11 gaps, 1 stops, absolute frame= Minus3 at 6314273..6315435 in the same strand
Class A

233.
CDS YP_001520507.1 IS4 family transposase intersects 100%(267aa) with IS5_IS1031 e-value= 3.6e-111 complete sequence hit coverage= 100%, between model(295 aa) positions 1; 295 length is 284aa with 14 gaps, 2 stops, absolute frame= Minus2 at 6332403..6333253 in the same strand
misc_feature no id DDE superfamily endonuclease; Region: DDE_4; cl15789 intersects 100%(83aa) with IS5_IS1031 e-value= 3.6e-111 complete sequence hit coverage= 100%, between model(295 aa) positions 1; 295 length is 284aa with 14 gaps, 2 stops, absolute frame= Minus2 at 6332403..6333253 in the same strand
misc_feature no id Transposase DDE domain; Region: DDE_Tnp_1; pfam01609 intersects 100%(155aa) with IS5_IS1031 e-value= 3.6e-111 complete sequence hit coverage= 100%, between model(295 aa) positions 1; 295 length is 284aa with 14 gaps, 2 stops, absolute frame= Minus2 at 6332403..6333253 in the same strand
misc_feature no id DDE superfamily endonuclease; Region: DDE_4; cl15789 intersects 100%(75aa) with IS5_IS1031 e-value= 3.6e-111 complete sequence hit coverage= 100%, between model(295 aa) positions 1; 295 length is 284aa with 14 gaps, 2 stops, absolute frame= Minus2 at 6332403..6333253 in the same strand
CDS YP_001520507.1 IS4 family transposase intersects 98.38%(262aa) with IS5_IS1031 e-value= 2.4e-113 fragment hit coverage= 93.56%, between model(295 aa) positions 20; 295 length is 270aa with 9 gaps, 1 stops, absolute frame= Minus2 at 6332403..6333211 in the same strand
misc_feature no id DDE superfamily endonuclease; Region: DDE_4; cl15789 intersects 100%(83aa) with IS5_IS1031 e-value= 2.4e-113 fragment hit coverage= 93.56%, between model(295 aa) positions 20; 295 length is 270aa with 9 gaps, 1 stops, absolute frame= Minus2 at 6332403..6333211 in the same strand

misc_feature no id Transposase DDE domain; Region: DDE_Tnp_1; pfam01609 intersects 100%(155aa) with IS5_IS1031 e-value= 2.4e-113 fragment hit coverage= 93.56%, between model(295 aa) positions 20; 295 length is 270aa with 9 gaps, 1 stops, absolute frame= Minus2 at 6332403..6333211 in the same strand
misc_feature no id DDE superfamily endonuclease; Region: DDE_4; cl15789 intersects 100%(75aa) with IS5_IS1031 e-value= 2.4e-113 fragment hit coverage= 93.56%, between model(295 aa) positions 20; 295 length is 270aa with 9 gaps, 1 stops, absolute frame= Minus2 at 6332403..6333211 in the same strand
CDS YP_001520507.1 IS4 family transposase intersects 100%(267aa) with IS5_IS5 e-value= 1.8e-09 complete sequence hit coverage= 100%, between model(422 aa) positions 1; 422 length is 290aa with 140 gaps, 4 stops, absolute frame= Minus2 at 6332424..6333292 in the same strand
misc_feature no id DDE superfamily endonuclease; Region: DDE_4; cl15789 intersects 100%(83aa) with IS5_IS5 e-value= 1.8e-09 complete sequence hit coverage= 100%, between model(422 aa) positions 1; 422 length is 290aa with 140 gaps, 4 stops, absolute frame= Minus2 at 6332424..6333292 in the same strand
misc_feature no id Transposase DDE domain; Region: DDE_Tnp_1; pfam01609 intersects 100%(155aa) with IS5_IS5 e-value= 1.8e-09 complete sequence hit coverage= 100%, between model(422 aa) positions 1; 422 length is 290aa with 140 gaps, 4 stops, absolute frame= Minus2 at 6332424..6333292 in the same strand
misc_feature no id DDE superfamily endonuclease; Region: DDE_4; cl15789 intersects 100%(75aa) with IS5_IS5 e-value= 1.8e-09 complete sequence hit coverage= 100%, between model(422 aa) positions 1; 422 length is 290aa with 140 gaps, 4 stops, absolute frame= Minus2 at 6332424..6333292 in the same strand
CDS YP_001520508.1 hypothetical protein intersects 0.11%(0aa) with IS5_IS5 e-value= 1.8e-09 complete sequence hit coverage= 100%, between model(422 aa) positions 1; 422 length is 290aa with 140 gaps, 4 stops, absolute frame= Minus2 at 6332424..6333292 in the same strand
CDS YP_001520507.1 IS4 family transposase intersects 57.30%(153aa) with IS5_IS427 e-value= 2.1e-06 complete sequence hit coverage= 100%, between model(296 aa) positions 1; 296 length is 275aa with 37 gaps, 14 stops, absolute frame= Minus2 at 6332766..6333589 in the same strand
misc_feature no id Transposase DDE domain; Region: DDE_Tnp_1; pfam01609 intersects 31.61%(49aa) with IS5_IS427 e-value= 2.1e-06 complete sequence hit coverage= 100%, between model(296 aa) positions 1; 296 length is 275aa with 37 gaps, 14 stops, absolute frame= Minus2 at 6332766..6333589 in the same strand
misc_feature no id DDE superfamily endonuclease; Region: DDE_4; cl15789 intersects 100%(75aa) with IS5_IS427 e-value= 2.1e-06 complete sequence hit coverage= 100%, between model(296 aa) positions 1; 296 length is 275aa with 37 gaps, 14 stops, absolute frame= Minus2 at 6332766..6333589 in the same strand
CDS YP_001520508.1 hypothetical protein intersects 33%(99aa) with IS5_IS427 e-value= 2.1e-06 complete sequence hit coverage= 100%, between model(296 aa) positions 1; 296 length is 275aa with 37 gaps, 14 stops, absolute frame= Minus2 at 6332766..6333589 in the same strand
CDS YP_001520507.1 IS4 family transposase intersects 29.84%(79aa) with IS5_IS427 e-value= 2e-19 fragment hit coverage= 27.70%, between model(296 aa) positions 125; 206 length is 80aa with 2 gaps, 0 stops, absolute frame= Minus2 at 6332964..6333202 in the same strand
misc_feature no id DDE superfamily endonuclease; Region: DDE_4; cl15789 intersects 100%(75aa) with IS5_IS427 e-value= 2e-19 fragment hit coverage= 27.70%, between model(296 aa) positions 125; 206 length is 80aa with 2 gaps, 0 stops, absolute frame= Minus2 at 6332964..6333202 in the same strand
Class A

234.
CDS YP_001520509.1 transposase intersects 98.96%(410aa) with ISL3 e-value= 1.2e-45 complete sequence hit coverage= 100%, between model(451 aa) positions 1; 451 length is 413aa with 44 gaps, 1 stops, absolute frame= Minus3 at 6334313..6335550 in the same strand
misc_feature no id Transposase and inactivated derivatives [DNA replication, recombination, and repair]; Region: COG3464 intersects 98.92%(398aa) with ISL3 e-value= 1.2e-45 complete sequence hit coverage= 100%, between model(451 aa) positions 1; 451 length is 413aa with 44 gaps, 1 stops, absolute frame= Minus3 at 6334313..6335550 in the same strand
misc_feature no id Transposase; Region: DDE_Tnp_ISL3; pfam01610 intersects 100%(246aa) with ISL3 e-value= 1.2e-45 complete sequence hit coverage= 100%, between model(451 aa) positions 1; 451 length is 413aa with 44 gaps, 1 stops, absolute frame= Minus3 at 6334313..6335550 in the same strand
CDS YP_001520509.1 transposase intersects 86.18%(357aa) with ISL3 e-value= 8.8e-52 fragment hit coverage= 86.03%, between model(451 aa) positions 64; 451 length is 360aa with 34 gaps, 1 stops, absolute frame= Minus3 at 6334313..6335391 in the same strand
misc_feature no id Transposase and inactivated derivatives [DNA replication, recombination, and repair]; Region: COG3464 intersects 85.77%(345aa) with ISL3 e-value= 8.8e-52 fragment hit coverage= 86.03%, between

model(451 aa) positions 64; 451 length is 360aa with 34 gaps, 1 stops, absolute frame= Minus3 at 6334313..6335391 in the same strand
misc_feature no id Transposase; Region: DDE_Tnp_ISL3; pfam01610 intersects 100%(246aa) with ISL3 e-value= 8.8e-52 fragment hit coverage= 86.03%, between model(451 aa) positions 64; 451 length is 360aa with 34 gaps, 1 stops, absolute frame= Minus3 at 6334313..6335391 in the same strand
Class A

235.
CDS YP_001520511.1 hypothetical protein intersects 93.51%(346aa) with IS630 e-value= 6.7e-25 complete sequence hit coverage= 100%, between model(376 aa) positions 1; 376 length is 346aa with 33 gaps, 0 stops, absolute frame= Plus1 at 6335869..6336906 in the same strand
misc_feature no id Helix-turn-helix domains; Region: HTH; cl00088 intersects 100%(50aa) with IS630 e-value= 6.7e-25 complete sequence hit coverage= 100%, between model(376 aa) positions 1; 376 length is 346aa with 33 gaps, 0 stops, absolute frame= Plus1 at 6335869..6336906 in the same strand
misc_feature no id Winged helix-turn helix; Region: HTH_29; pfam13551 intersects 100%(102aa) with IS630 e-value= 6.7e-25 complete sequence hit coverage= 100%, between model(376 aa) positions 1; 376 length is 346aa with 33 gaps, 0 stops, absolute frame= Plus1 at 6335869..6336906 in the same strand
misc_feature no id Winged helix-turn helix; Region: HTH_33; pfam13592 intersects 100%(56aa) with IS630 e-value= 6.7e-25 complete sequence hit coverage= 100%, between model(376 aa) positions 1; 376 length is 346aa with 33 gaps, 0 stops, absolute frame= Plus1 at 6335869..6336906 in the same strand
misc_feature no id Integrase core domain; Region: rve; cl01316 intersects 100%(141aa) with IS630 e-value= 6.7e-25 complete sequence hit coverage= 100%, between model(376 aa) positions 1; 376 length is 346aa with 33 gaps, 0 stops, absolute frame= Plus1 at 6335869..6336906 in the same strand
CDS YP_001520511.1 hypothetical protein intersects 14.32%(53aa) with IS481 e-value= 3.7e-09 fragment hit coverage= 15.10%, between model(351 aa) positions 1; 53 length is 53aa with 0 gaps, 0 stops, absolute frame= Plus1 at 6335929..6336087 in the same strand
misc_feature no id Helix-turn-helix domains; Region: HTH; cl00088 intersects 100%(50aa) with IS481 e-value= 3.7e-09 fragment hit coverage= 15.10%, between model(351 aa) positions 1; 53 length is 53aa with 0 gaps, 0 stops, absolute frame= Plus1 at 6335929..6336087 in the same strand
misc_feature no id Winged helix-turn helix; Region: HTH_29; pfam13551 intersects 43.14%(44aa) with IS481 e-value= 3.7e-09 fragment hit coverage= 15.10%, between model(351 aa) positions 1; 53 length is 53aa with 0 gaps, 0 stops, absolute frame= Plus1 at 6335929..6336087 in the same strand
Class A

236.
CDS YP_001520512.1 transposase intersects 85.96%(375aa) with IS4 e-value= 9.9e-07 complete sequence hit coverage= 100%, between model(486 aa) positions 1; 486 length is 376aa with 117 gaps, 0 stops, absolute frame= Minus3 at 6337034..6338160 in the same strand
misc_feature no id DDE superfamily endonuclease; Region: DDE_5; cl02413 intersects 93.48%(348aa) with IS4 e-value= 9.9e-07 complete sequence hit coverage= 100%, between model(486 aa) positions 1; 486 length is 376aa with 117 gaps, 0 stops, absolute frame= Minus3 at 6337034..6338160 in the same strand
CDS YP_001520512.1 transposase intersects 53.01%(231aa) with IS4 e-value= 4.7e-14 fragment hit coverage= 51.23%, between model(486 aa) positions 1; 249 length is 232aa with 27 gaps, 0 stops, absolute frame= Minus3 at 6337466..6338160 in the same strand
misc_feature no id DDE superfamily endonuclease; Region: DDE_5; cl02413 intersects 62.11%(231aa) with IS4 e-value= 4.7e-14 fragment hit coverage= 51.23%, between model(486 aa) positions 1; 249 length is 232aa with 27 gaps, 0 stops, absolute frame= Minus3 at 6337466..6338160 in the same strand
Class A

237.
CDS YP_001520519.1 hypothetical protein intersects 52.08%(166aa) with IS4 e-value= 3e-08 fragment hit coverage= 37.04%, between model(486 aa) positions 307; 486 length is 167aa with 19 gaps, 0 stops, absolute frame= Minus1 at 6343381..6343880 in the same strand
misc_feature no id Transposase [DNA replication, recombination, and repair]; Region: COG5421 intersects 62.68%(146aa) with IS4 e-value= 3e-08 fragment hit coverage= 37.04%, between model(486 aa) positions 307; 486 length is 167aa with 19 gaps, 0 stops, absolute frame= Minus1 at 6343381..6343880 in the same strand
Class A

238.
CDS YP_001520524.1 IS4 family transposase intersects 99.35%(308aa) with IS5_IS903 e-value= 2.2e-96 complete sequence hit coverage= 100%, between model(347 aa) positions 1; 347 length is 314aa with 37 gaps, 1 stops, absolute frame= Minus3 at 6346655..6347595 in the same strand
misc_feature no id Transposase DDE domain; Region: DDE_Tnp_1; pfam01609 intersects 100%(186aa) with IS5_IS903 e-value= 2.2e-96 complete sequence hit coverage= 100%, between model(347 aa) positions 1; 347 length is 314aa with 37 gaps, 1 stops, absolute frame= Minus3 at 6346655..6347595 in the same strand
misc_feature no id Transposase DDE domain; Region: DDE_Tnp_1_5; pfam13737 intersects 100%(112aa) with IS5_IS903 e-value= 2.2e-96 complete sequence hit coverage= 100%, between model(347 aa) positions 1; 347 length is 314aa with 37 gaps, 1 stops, absolute frame= Minus3 at 6346655..6347595 in the same strand
Class A

239.
CDS YP_001520527.1 hypothetical protein intersects 45.07%(32aa) with IS1_ORF1 e-value= 9.8e-10 fragment hit coverage= 36.82%, between model(239 aa) positions 1; 88 length is 94aa with 3 gaps, 0 stops, absolute frame= Minus1 at 6348694..6348974 in the same strand
Class B

240.
CDS YP_001520529.1 transposase intersects 100%(96aa) with IS200 e-value= 2.5e-35 complete sequence hit coverage= 100%, between model(154 aa) positions 1; 154 length is 148aa with 6 gaps, 4 stops, absolute frame= Minus3 at 6349730..6350172 in the same strand
misc_feature no id Transposase IS200 like; Region: Y1_Tnp; cl00848 intersects 100%(92aa) with IS200 e-value= 2.5e-35 complete sequence hit coverage= 100%, between model(154 aa) positions 1; 154 length is 148aa with 6 gaps, 4 stops, absolute frame= Minus3 at 6349730..6350172 in the same strand
CDS YP_001520529.1 transposase intersects 100%(96aa) with IS200_IS605 e-value= 8.1e-37 complete sequence hit coverage= 100%, between model(153 aa) positions 1; 153 length is 147aa with 6 gaps, 4 stops, absolute frame= Minus3 at 6349733..6350172 in the same strand
misc_feature no id Transposase IS200 like; Region: Y1_Tnp; cl00848 intersects 100%(92aa) with IS200_IS605 e-value= 8.1e-37 complete sequence hit coverage= 100%, between model(153 aa) positions 1; 153 length is 147aa with 6 gaps, 4 stops, absolute frame= Minus3 at 6349733..6350172 in the same strand
CDS YP_001520529.1 transposase intersects 100%(96aa) with IS605 e-value= 1e-36 complete sequence hit coverage= 100%, between model(152 aa) positions 1; 152 length is 147aa with 5 gaps, 4 stops, absolute frame= Minus3 at 6349745..6350184 in the same strand
misc_feature no id Transposase IS200 like; Region: Y1_Tnp; cl00848 intersects 100%(92aa) with IS605 e-value= 1e-36 complete sequence hit coverage= 100%, between model(152 aa) positions 1; 152 length is 147aa with 5 gaps, 4 stops, absolute frame= Minus3 at 6349745..6350184 in the same strand
CDS YP_001520529.1 transposase intersects 95.49%(91aa) with IS605 e-value= 2.1e-41 fragment hit coverage= 83.55%, between model(152 aa) positions 17; 143 length is 127aa with 0 gaps, 1 stops, absolute frame= Minus3 at 6349760..6350139 in the same strand
misc_feature no id Transposase IS200 like; Region: Y1_Tnp; cl00848 intersects 98.55%(90aa) with IS605 e-value= 2.1e-41 fragment hit coverage= 83.55%, between model(152 aa) positions 17; 143 length is 127aa with 0 gaps, 1 stops, absolute frame= Minus3 at 6349760..6350139 in the same strand
CDS YP_001520529.1 transposase intersects 95.49%(91aa) with IS200 e-value= 1.2e-40 fragment hit coverage= 83.12%, between model(154 aa) positions 16; 143 length is 126aa with 2 gaps, 1 stops, absolute frame= Minus3 at 6349763..6350139 in the same strand
misc_feature no id Transposase IS200 like; Region: Y1_Tnp; cl00848 intersects 98.55%(90aa) with IS200 e-value= 1.2e-40 fragment hit coverage= 83.12%, between model(154 aa) positions 16; 143 length is 126aa with 2 gaps, 1 stops, absolute frame= Minus3 at 6349763..6350139 in the same strand
Class A

241.
CDS YP_001520534.1 IS4 family transposase intersects 2.31%(6aa) with IS630 e-value= 3.7e-16 complete sequence hit coverage= 100%, between model(376 aa) positions 1; 376 length is 342aa with 39 gaps, 1 stops, absolute frame= Minus3 at 6360251..6361275 in the same strand

CDS YP_001520535.1 hypothetical protein intersects 99.30%(332aa) with IS630 e-value= 3.7e-16 complete sequence hit coverage= 100%, between model(376 aa) positions 1; 376 length is 342aa with 39 gaps, 1 stops, absolute frame= Minus3 at 6360251..6361275 in the same strand
misc_feature no id Integrase core domain; Region: rve; cl01316 intersects 100%(132aa) with IS630 e-value= 3.7e-16 complete sequence hit coverage= 100%, between model(376 aa) positions 1; 376 length is 342aa with 39 gaps, 1 stops, absolute frame= Minus3 at 6360251..6361275 in the same strand
misc_feature no id Winged helix-turn helix; Region: HTH_29; pfam13551 intersects 100%(99aa) with IS630 e-value= 3.7e-16 complete sequence hit coverage= 100%, between model(376 aa) positions 1; 376 length is 342aa with 39 gaps, 1 stops, absolute frame= Minus3 at 6360251..6361275 in the same strand
misc_feature no id Helix-turn-helix domains; Region: HTH; cl00088 intersects 100%(72aa) with IS630 e-value= 3.7e-16 complete sequence hit coverage= 100%, between model(376 aa) positions 1; 376 length is 342aa with 39 gaps, 1 stops, absolute frame= Minus3 at 6360251..6361275 in the same strand
misc_feature no id Helix-turn-helix domains; Region: HTH; cl00088 intersects 100%(52aa) with IS630 e-value= 3.7e-16 complete sequence hit coverage= 100%, between model(376 aa) positions 1; 376 length is 342aa with 39 gaps, 1 stops, absolute frame= Minus3 at 6360251..6361275 in the same strand
CDS YP_001520535.1 hypothetical protein intersects 16.92%(56aa) with IS630 e-value= 1.9e-12 fragment hit coverage= 14.89%, between model(376 aa) positions 277; 332 length is 57aa with 0 gaps, 0 stops, absolute frame= Minus3 at 6360377..6360546 in the same strand
misc_feature no id Integrase core domain; Region: rve; cl01316 intersects 42.93%(56aa) with IS630 e-value= 1.9e-12 fragment hit coverage= 14.89%, between model(376 aa) positions 277; 332 length is 57aa with 0 gaps, 0 stops, absolute frame= Minus3 at 6360377..6360546 in the same strand
CDS YP_001520535.1 hypothetical protein intersects 66.47%(222aa) with IS630 e-value= 4.6e-12 fragment hit coverage= 63.83%, between model(376 aa) positions 1; 240 length is 223aa with 20 gaps, 0 stops, absolute frame= Minus3 at 6360608..6361275 in the same strand
misc_feature no id Integrase core domain; Region: rve; cl01316 intersects 40.91%(54aa) with IS630 e-value= 4.6e-12 fragment hit coverage= 63.83%, between model(376 aa) positions 1; 240 length is 223aa with 20 gaps, 0 stops, absolute frame= Minus3 at 6360608..6361275 in the same strand
misc_feature no id Winged helix-turn helix; Region: HTH_29; pfam13551 intersects 100%(99aa) with IS630 e-value= 4.6e-12 fragment hit coverage= 63.83%, between model(376 aa) positions 1; 240 length is 223aa with 20 gaps, 0 stops, absolute frame= Minus3 at 6360608..6361275 in the same strand
misc_feature no id Helix-turn-helix domains; Region: HTH; cl00088 intersects 100%(72aa) with IS630 e-value= 4.6e-12 fragment hit coverage= 63.83%, between model(376 aa) positions 1; 240 length is 223aa with 20 gaps, 0 stops, absolute frame= Minus3 at 6360608..6361275 in the same strand
misc_feature no id Helix-turn-helix domains; Region: HTH; cl00088 intersects 100%(52aa) with IS630 e-value= 4.6e-12 fragment hit coverage= 63.83%, between model(376 aa) positions 1; 240 length is 223aa with 20 gaps, 0 stops, absolute frame= Minus3 at 6360608..6361275 in the same strand
Class A

242.
CDS YP_001520558.1 IS4 family transposase intersects 98.38%(262aa) with IS5_IS1031 e-value= 2.4e-113 fragment hit coverage= 93.56%, between model(295 aa) positions 20; 295 length is 270aa with 9 gaps, 1 stops, absolute frame= Minus3 at 6378272..6379080 in the same strand
misc_feature no id DDE superfamily endonuclease; Region: DDE_4; cl15789 intersects 100%(83aa) with IS5_IS1031 e-value= 2.4e-113 fragment hit coverage= 93.56%, between model(295 aa) positions 20; 295 length is 270aa with 9 gaps, 1 stops, absolute frame= Minus3 at 6378272..6379080 in the same strand
misc_feature no id Transposase DDE domain; Region: DDE_Tnp_1; pfam01609 intersects 100%(155aa) with IS5_IS1031 e-value= 2.4e-113 fragment hit coverage= 93.56%, between model(295 aa) positions 20; 295 length is 270aa with 9 gaps, 1 stops, absolute frame= Minus3 at 6378272..6379080 in the same strand
misc_feature no id DDE superfamily endonuclease; Region: DDE_4; cl15789 intersects 100%(75aa) with IS5_IS1031 e-value= 2.4e-113 fragment hit coverage= 93.56%, between model(295 aa) positions 20; 295 length is 270aa with 9 gaps, 1 stops, absolute frame= Minus3 at 6378272..6379080 in the same strand
CDS YP_001520558.1 IS4 family transposase intersects 100%(267aa) with IS5_IS1031 e-value= 3.6e-111 complete sequence hit coverage= 100%, between model(295 aa) positions 1; 295 length is 284aa with 14 gaps, 2 stops, absolute frame= Minus3 at 6378272..6379122 in the same strand
misc_feature no id DDE superfamily endonuclease; Region: DDE_4; cl15789 intersects 100%(83aa) with IS5_IS1031 e-value= 3.6e-111 complete sequence hit coverage= 100%, between model(295 aa) positions 1; 295 length is 284aa with 14 gaps, 2 stops, absolute frame= Minus3 at 6378272..6379122 in the same strand

misc_feature no id Transposase DDE domain; Region: DDE_Tnp_1; pfam01609 intersects 100%(155aa) with IS5_IS1031 e-value= 3.6e-111 complete sequence hit coverage= 100%, between model(295 aa) positions 1; 295 length is 284aa with 14 gaps, 2 stops, absolute frame= Minus3 at 6378272..6379122 in the same strand
misc_feature no id DDE superfamily endonuclease; Region: DDE_4; cl15789 intersects 100%(75aa) with IS5_IS1031 e-value= 3.6e-111 complete sequence hit coverage= 100%, between model(295 aa) positions 1; 295 length is 284aa with 14 gaps, 2 stops, absolute frame= Minus3 at 6378272..6379122 in the same strand
CDS YP_001520558.1 IS4 family transposase intersects 100%(267aa) with IS5_IS5 e-value= 1.8e-09 complete sequence hit coverage= 100%, between model(422 aa) positions 1; 422 length is 290aa with 140 gaps, 4 stops, absolute frame= Minus3 at 6378293..6379161 in the same strand
misc_feature no id DDE superfamily endonuclease; Region: DDE_4; cl15789 intersects 100%(83aa) with IS5_IS5 e-value= 1.8e-09 complete sequence hit coverage= 100%, between model(422 aa) positions 1; 422 length is 290aa with 140 gaps, 4 stops, absolute frame= Minus3 at 6378293..6379161 in the same strand
misc_feature no id Transposase DDE domain; Region: DDE_Tnp_1; pfam01609 intersects 100%(155aa) with IS5_IS5 e-value= 1.8e-09 complete sequence hit coverage= 100%, between model(422 aa) positions 1; 422 length is 290aa with 140 gaps, 4 stops, absolute frame= Minus3 at 6378293..6379161 in the same strand
misc_feature no id DDE superfamily endonuclease; Region: DDE_4; cl15789 intersects 100%(75aa) with IS5_IS5 e-value= 1.8e-09 complete sequence hit coverage= 100%, between model(422 aa) positions 1; 422 length is 290aa with 140 gaps, 4 stops, absolute frame= Minus3 at 6378293..6379161 in the same strand
CDS YP_001520559.1 hypothetical protein intersects 0.42%(0aa) with IS5_IS5 e-value= 1.8e-09 complete sequence hit coverage= 100%, between model(422 aa) positions 1; 422 length is 290aa with 140 gaps, 4 stops, absolute frame= Minus3 at 6378293..6379161 in the same strand
CDS YP_001520558.1 IS4 family transposase intersects 57.30%(153aa) with IS5_IS427 e-value= 4.4e-06 complete sequence hit coverage= 100%, between model(296 aa) positions 1; 296 length is 256aa with 47 gaps, 6 stops, absolute frame= Minus3 at 6378635..6379401 in the same strand
misc_feature no id Transposase DDE domain; Region: DDE_Tnp_1; pfam01609 intersects 31.61%(49aa) with IS5_IS427 e-value= 4.4e-06 complete sequence hit coverage= 100%, between model(296 aa) positions 1; 296 length is 256aa with 47 gaps, 6 stops, absolute frame= Minus3 at 6378635..6379401 in the same strand
misc_feature no id DDE superfamily endonuclease; Region: DDE_4; cl15789 intersects 100%(75aa) with IS5_IS427 e-value= 4.4e-06 complete sequence hit coverage= 100%, between model(296 aa) positions 1; 296 length is 256aa with 47 gaps, 6 stops, absolute frame= Minus3 at 6378635..6379401 in the same strand
CDS YP_001520559.1 hypothetical protein intersects 100%(80aa) with IS5_IS427 e-value= 4.4e-06 complete sequence hit coverage= 100%, between model(296 aa) positions 1; 296 length is 256aa with 47 gaps, 6 stops, absolute frame= Minus3 at 6378635..6379401 in the same strand
CDS YP_001520558.1 IS4 family transposase intersects 29.84%(79aa) with IS5_IS427 e-value= 2e-19 fragment hit coverage= 27.70%, between model(296 aa) positions 125; 206 length is 80aa with 2 gaps, 0 stops, absolute frame= Minus3 at 6378833..6379071 in the same strand
misc_feature no id DDE superfamily endonuclease; Region: DDE_4; cl15789 intersects 100%(75aa) with IS5_IS427 e-value= 2e-19 fragment hit coverage= 27.70%, between model(296 aa) positions 125; 206 length is 80aa with 2 gaps, 0 stops, absolute frame= Minus3 at 6378833..6379071 in the same strand
Class A

243.
CDS YP_001520575.1 IS4 family transposase intersects 99.35%(308aa) with IS5_IS903 e-value= 2.2e-96 complete sequence hit coverage= 100%, between model(347 aa) positions 1; 347 length is 314aa with 37 gaps, 1 stops, absolute frame= Minus1 at 6394549..6395489 in the same strand
misc_feature no id Transposase DDE domain; Region: DDE_Tnp_1; pfam01609 intersects 100%(186aa) with IS5_IS903 e-value= 2.2e-96 complete sequence hit coverage= 100%, between model(347 aa) positions 1; 347 length is 314aa with 37 gaps, 1 stops, absolute frame= Minus1 at 6394549..6395489 in the same strand
misc_feature no id Transposase DDE domain; Region: DDE_Tnp_1_5; pfam13737 intersects 100%(112aa) with IS5_IS903 e-value= 2.2e-96 complete sequence hit coverage= 100%, between model(347 aa) positions 1; 347 length is 314aa with 37 gaps, 1 stops, absolute frame= Minus1 at 6394549..6395489 in the same strand
Class A

244.
CDS YP_001520642.1 transposase intersects 80.29%(110aa) with IS630 e-value= 6e-07 fragment hit coverage= 32.45%, between model(376 aa) positions 25; 146 length is 111aa with 11 gaps, 1 stops, absolute frame= Plus1 at 6483169..6483501 in the same strand

misc_feature no id Helix-turn-helix domains; Region: HTH; cl00088 intersects 98%(49aa) with IS630 e-value= 6e-07 fragment hit coverage= 32.45%, between model(376 aa) positions 25; 146 length is 111aa with 11 gaps, 1 stops, absolute frame= Plus1 at 6483169..6483501 in the same strand
misc_feature no id Winged helix-turn helix; Region: HTH_29; pfam13551 intersects 100%(104aa) with IS630 e-value= 6e-07 fragment hit coverage= 32.45%, between model(376 aa) positions 25; 146 length is 111aa with 11 gaps, 1 stops, absolute frame= Plus1 at 6483169..6483501 in the same strand
misc_feature no id Helix-turn-helix domains; Region: HTH; cl00088 intersects 100%(74aa) with IS630 e-value= 6e-07 fragment hit coverage= 32.45%, between model(376 aa) positions 25; 146 length is 111aa with 11 gaps, 1 stops, absolute frame= Plus1 at 6483169..6483501 in the same strand
Class A

245.
gene no id fragment; similar to N-terminus of transposase intersects 79.57%(74aa) with IS5_IS1031 e-value= 3.3e-39 fragment hit coverage= 27.46%, between model(295 aa) positions 20; 100 length is 74aa with 7 gaps, 0 stops, absolute frame= Plus2 at 6501464..6501685 in the same strand
CDS YP_001520657.1 chorismate mutase intersects 3.36%(4aa) with IS5_IS1031 e-value= 3.3e-39 fragment hit coverage= 27.46%, between model(295 aa) positions 20; 100 length is 74aa with 7 gaps, 0 stops, absolute frame= Plus2 at 6501464..6501685 in the same strand
gene no id fragment; similar to N-terminus of transposase intersects 76.34%(71aa) with IS5_IS427 e-value= 7.3e-19 fragment hit coverage= 24.66%, between model(296 aa) positions 125; 197 length is 71aa with 2 gaps, 0 stops, absolute frame= Plus2 at 6501473..6501685 in the same strand
CDS YP_001520657.1 chorismate mutase intersects 3.36%(4aa) with IS5_IS427 e-value= 7.3e-19 fragment hit coverage= 24.66%, between model(296 aa) positions 125; 197 length is 71aa with 2 gaps, 0 stops, absolute frame= Plus2 at 6501473..6501685 in the same strand
Class A

Not Found: 467429..468847	-1	transposase
Not Found: 692424..692906	1	Transposase DDE domain; Region: DDE_Tnp_1; pfam01609
Not Found: 740957..741583	-1	transposase
Not Found: 1529443..1530069	1	fragment; similar to C-terminus of transposase
Not Found: 1769008..1770426	1	IS4 family transposase
Not Found: 2065371..2066789	1	IS4 family transposase
Not Found: 2085748..2086086	1	Transposase; Region: HTH_Tnp_IS630; pfam01710
Not Found: 2128098..2128580	1	Transposase DDE domain; Region: DDE_Tnp_1; pfam01609
Not Found: 2407731..2408351	-1	Transposase DDE domain; Region: DDE_Tnp_1; pfam01609
Not Found: 2709690..2710049	-1	fragment; similar to N-terminus of transposase
Not Found: 2744668..2745222	-1	transposase
Not Found: 3048166..3049584	1	IS4 family transposase
Not Found: 3243381..3244799	-1	IS4 family transposase
Not Found: 3756217..3756426	1	Transposase Tn5 dimerisation domain; Region: Dimer_Tnp_Tn5; pfam02281
Not Found: 4219460..4220878	-1	IS4 family transposase
Not Found: 4308561..4309040	-1	transposase
Not Found: 4467315..4467794	-1	transposase
Not Found: 5351415..5351825	1	Transposase DDE domain; Region: DDE_Tnp_1; pfam01609
Not Found: 5623434..5624852	-1	IS4 family transposase
Not Found: 5721194..5722612	1	IS4 family transposase
Not Found: 5945791..5947209	-1	IS4 family transposase
Not Found: 6060468..6061013	-1	fragment; similar to C-terminus of transposase
Not Found: 6062553..6062762	1	Transposase Tn5 dimerisation domain; Region: Dimer_Tnp_Tn5; pfam02281
Not Found: 6150219..6151637	1	IS4 family transposase
Not Found: 6252000..6252455	-1	Tn3 transposase DDE domain; Region: DDE_Tnp_Tn3; cl14901
Not Found: 6295713..6296156	1	Tn3 transposase DDE domain; Region: DDE_Tnp_Tn3; cl14901
Not Found: 6483576..6484103	1	transposase

Pred: 105655..105725 -1 IS605 e-value= 2.8e-08 fragment hit coverage= 15.79%, between model(152 aa) positions 61; 84 length is 24aa with 0 gaps, 0 stops, absolute frame= Minus1
Pred: 439435..439710 1 IS21_ORF2 e-value= 4.1e-16 fragment hit coverage= 36.43%, between model(258 aa) positions 148; 241 length is 92aa with 2 gaps, 1 stops, absolute frame= Plus1
Pred: 689482..689604 1 IS200_IS605 e-value= 3.1e-06 fragment hit coverage= 30.07%, between model(153 aa) positions 1; 46 length is 41aa with 5 gaps, 0 stops, absolute frame= Plus1
Pred: 689482..689622 1 IS200 e-value= 3.9e-06 fragment hit coverage= 33.77%, between model(154 aa) positions 1; 52 length is 47aa with 5 gaps, 0 stops, absolute frame= Plus1
Pred: 2845484..2846386 1 IS630 e-value= 1.1e-24 fragment hit coverage= 88.56%, between model(376 aa) positions 1; 333 length is 301aa with 36 gaps, 0 stops, absolute frame= Plus2
Pred: 4134772..4135019 -1 IS1_ORF1 e-value= 2.5e-12 fragment hit coverage= 34.31%, between model(239 aa) positions 6; 87 length is 83aa with 1 gaps, 2 stops, absolute frame= Minus1
Pred: 4134790..4134977 -1 IS1_ORF2 e-value= 4.3e-06 fragment hit coverage= 24.89%, between model(237 aa) positions 21; 79 length is 63aa with 0 gaps, 1 stops, absolute frame= Minus1
Pred: 5069026..5069105 -1 IS5_IS1031 e-value= 5.8e-07 fragment hit coverage= 9.15%, between model(295 aa) positions 246; 272 length is 27aa with 0 gaps, 0 stops, absolute frame= Minus1
Pred: 5391422..5391534 -1 IS5_IS1031 e-value= 2.7e-08 fragment hit coverage= 12.88%, between model(295 aa) positions 258; 295 length is 38aa with 0 gaps, 2 stops, absolute frame= Minus3
Pred: 5652012..5652220 -1 IS3_IS51_ORF1 e-value= 1.5e-22 fragment hit coverage= 24.10%, between model(307 aa) positions 234; 307 length is 70aa with 4 gaps, 1 stops, absolute frame= Minus2
Pred: 5652030..5652220 -1 IS3_IS51_ORF2 e-value= 2.3e-14 fragment hit coverage= 20.50%, between model(317 aa) positions 245; 309 length is 64aa with 1 gaps, 0 stops, absolute frame= Minus2
Pred: 5652039..5652220 -1 IS3_IS150_ORF1 e-value= 1.1e-07 fragment hit coverage= 20.59%, between model(306 aa) positions 244; 306 length is 61aa with 2 gaps, 0 stops, absolute frame= Minus2
Pred: 5652042..5652220 -1 IS3_IS3_ORF1 e-value= 3.3e-23 fragment hit coverage= 21.68%, between model(286 aa) positions 225; 286 length is 60aa with 2 gaps, 0 stops, absolute frame= Minus2
Pred: 5652060..5652220 -1 IS3_IS150_ORF2 e-value= 3.7e-12 fragment hit coverage= 14.14%, between model(389 aa) positions 327; 381 length is 54aa with 2 gaps, 0 stops, absolute frame= Minus2
Pred: 5666475..5666933 1 IS200 e-value= 3.1e-10 complete sequence hit coverage= 100%, between model(154 aa) positions 1; 154 length is 153aa with 7 gaps, 8 stops, absolute frame= Plus3
Pred: 5666475..5666906 1 IS605 e-value= 1.7e-10 complete sequence hit coverage= 100%, between model(152 aa) positions 1; 152 length is 144aa with 11 gaps, 7 stops, absolute frame= Plus3
Pred: 5666475..5666576 1 IS605 e-value= 1.1e-11 fragment hit coverage= 23.03%, between model(152 aa) positions 1; 35 length is 34aa with 1 gaps, 0 stops, absolute frame= Plus3
Pred: 5666503..5666847 1 IS605 e-value= 9.1e-06 complete sequence hit coverage= 100%, between model(152 aa) positions 1; 152 length is 115aa with 40 gaps, 3 stops, absolute frame= Plus1
Pred: 5666590..5666721 1 IS200_IS605 e-value= 1.4e-14 fragment hit coverage= 28.76%, between model(153 aa) positions 41; 84 length is 44aa with 0 gaps, 0 stops, absolute frame= Plus1
Pred: 5666629..5666721 1 IS605 e-value= 2.2e-10 fragment hit coverage= 20.39%, between model(152 aa) positions 54; 84 length is 31aa with 0 gaps, 0 stops, absolute frame= Plus1
Pred: 5666769..5666846 1 IS605 e-value= 5.7e-09 fragment hit coverage= 17.11%, between model(152 aa) positions 102; 127 length is 26aa with 0 gaps, 1 stops, absolute frame= Plus3
Pred: 6313894..6314132 -1 IS21_ORF2 e-value= 1.3e-09 fragment hit coverage= 31.40%, between model(258 aa) positions 157; 237 length is 80aa with 1 gaps, 1 stops, absolute frame= Minus1
