# Supplementary material for: TnpPred: A Web Service for the Robust Prediction of Prokaryotic Transposases
Source: Comp Funct Genomics. 2012 Nov 18;2012:678761. doi: 10.1155/2012/678761 (PMC3506888; doi:10.1155/2012/678761)
Supplement: Supplementary file 5 [file 678761.f5.pdf]

**Supplementary file 4**
*Stenotrophomonas maltophilia* K279a full report of TnpPred predictions.

NC_010943.gbk Chromosome

1.
CDS YP_001969960.1 putative ISXac3 like transposase intersects 100%(287aa) with IS3_IS51_ORF2 e-value= 9.6e-57 complete sequence hit coverage= 100%, length is 310aa with 17 gaps, 2 stops, frame= Minus2 at 25851..26779 in the same strand
misc_feature no id HMMPfam hit to PF00665, Integrase core domain, score 3.3e-32 intersects 100%(161aa) with IS3_IS51_ORF2 e-value= 9.6e-57 complete sequence hit coverage= 100%, length is 310aa with 17 gaps, 2 stops, frame= Minus2 at 25851..26779 in the same strand
misc_feature no id putative transposase OrfB; Reviewed; Region: PHA02517 intersects 100%(250aa) with IS3_IS51_ORF2 e-value= 9.6e-57 complete sequence hit coverage= 100%, length is 310aa with 17 gaps, 2 stops, frame= Minus2 at 25851..26779 in the same strand
misc_feature no id Integrase core domain; Region: rve; cl01316 intersects 100%(117aa) with IS3_IS51_ORF2 e-value= 9.6e-57 complete sequence hit coverage= 100%, length is 310aa with 17 gaps, 2 stops, frame= Minus2 at 25851..26779 in the same strand
CDS YP_001969961.1 putative ISXac3 related insertion element intersects 5.21%(5aa) with IS3_IS51_ORF2 e-value= 9.6e-57 complete sequence hit coverage= 100%, length is 310aa with 17 gaps, 2 stops, frame= Minus2 at 25851..26779 in the same strand
CDS YP_001969960.1 putative ISXac3 like transposase intersects 99.30%(285aa) with IS3_IS51_ORF1 e-value= 7.6e-67 complete sequence hit coverage= 100%, length is 287aa with 21 gaps, 0 stops, frame= Minus2 at 25902..26761 in the same strand
misc_feature no id HMMPfam hit to PF00665, Integrase core domain, score 3.3e-32 intersects 100%(161aa) with IS3_IS51_ORF1 e-value= 7.6e-67 complete sequence hit coverage= 100%, length is 287aa with 21 gaps, 0 stops, frame= Minus2 at 25902..26761 in the same strand
misc_feature no id putative transposase OrfB; Reviewed; Region: PHA02517 intersects 100%(250aa) with IS3_IS51_ORF1 e-value= 7.6e-67 complete sequence hit coverage= 100%, length is 287aa with 21 gaps, 0 stops, frame= Minus2 at 25902..26761 in the same strand
misc_feature no id Integrase core domain; Region: rve; cl01316 intersects 100%(117aa) with IS3_IS51_ORF1 e-value= 7.6e-67 complete sequence hit coverage= 100%, length is 287aa with 21 gaps, 0 stops, frame= Minus2 at 25902..26761 in the same strand
CDS YP_001969960.1 putative ISXac3 like transposase intersects 97.56%(280aa) with IS3_IS150_ORF1 e-value= 1.5e-33 complete sequence hit coverage= 100%, length is 300aa with 10 gaps, 0 stops, frame= Minus2 at 25917..26815 in the same strand
misc_feature no id HMMPfam hit to PF00665, Integrase core domain, score 3.3e-32 intersects 100%(161aa) with IS3_IS150_ORF1 e-value= 1.5e-33 complete sequence hit coverage= 100%, length is 300aa with 10 gaps, 0 stops, frame= Minus2 at 25917..26815 in the same strand
misc_feature no id putative transposase OrfB; Reviewed; Region: PHA02517 intersects 100%(250aa) with IS3_IS150_ORF1 e-value= 1.5e-33 complete sequence hit coverage= 100%, length is 300aa with 10 gaps, 0 stops, frame= Minus2 at 25917..26815 in the same strand
misc_feature no id Integrase core domain; Region: rve; cl01316 intersects 100%(117aa) with IS3_IS150_ORF1 e-value= 1.5e-33 complete sequence hit coverage= 100%, length is 300aa with 10 gaps, 0 stops, frame= Minus2 at 25917..26815 in the same strand
CDS YP_001969961.1 putative ISXac3 related insertion element intersects 17.71%(17aa) with IS3_IS150_ORF1 e-value= 1.5e-33 complete sequence hit coverage= 100%, length is 300aa with 10 gaps, 0 stops, frame= Minus2 at 25917..26815 in the same strand
misc_feature no id HMMPfam hit to PF01527, Transposase, score 1.1e-20 intersects 5%(4aa) with IS3_IS150_ORF1 e-value= 1.5e-33 complete sequence hit coverage= 100%, length is 300aa with 10 gaps, 0 stops, frame= Minus2 at 25917..26815 in the same strand
CDS YP_001969960.1 putative ISXac3 like transposase intersects 96.52%(277aa) with IS3_IS3_ORF1 e-value= 2.4e-72 complete sequence hit coverage= 100%, length is 278aa with 10 gaps, 0 stops, frame= Minus2 at 25926..26758 in the same strand
misc_feature no id HMMPfam hit to PF00665, Integrase core domain, score 3.3e-32 intersects 98.76%(159aa) with IS3_IS3_ORF1 e-value= 2.4e-72 complete sequence hit coverage= 100%, length is 278aa with 10 gaps, 0 stops, frame= Minus2 at 25926..26758 in the same strand

misc_feature	no id	putative transposase OrfB; Reviewed; Region: PHA02517 intersects 100%(250aa) with IS3_IS3_ORF1	e-value= 2.4e-72 complete sequence hit coverage= 100%, length is 278aa with 10 gaps, 0 stops, frame= Minus2 at 25926..26758 in the same strand
misc_feature	no id	Integrase core domain; Region: rve; cl01316 intersects 100%(117aa) with IS3_IS3_ORF1	e-value= 2.4e-72 complete sequence hit coverage= 100%, length is 278aa with 10 gaps, 0 stops, frame= Minus2 at 25926..26758 in the same strand
CDS	YP_001969960.1	putative ISXac3 like transposase intersects 95.01%(272aa) with IS3_IS3_ORF2	e-value= 2.6e-66 complete sequence hit coverage= 100%, length is 273aa with 19 gaps, 0 stops, frame= Minus2 at 25929..26746 in the same strand
misc_feature	no id	HMMPfam hit to PF00665, Integrase core domain, score 3.3e-32 intersects 98.14%(158aa) with IS3_IS3_ORF2	e-value= 2.6e-66 complete sequence hit coverage= 100%, length is 273aa with 19 gaps, 0 stops, frame= Minus2 at 25929..26746 in the same strand
misc_feature	no id	putative transposase OrfB; Reviewed; Region: PHA02517 intersects 100%(250aa) with IS3_IS3_ORF2	e-value= 2.6e-66 complete sequence hit coverage= 100%, length is 273aa with 19 gaps, 0 stops, frame= Minus2 at 25929..26746 in the same strand
misc_feature	no id	Integrase core domain; Region: rve; cl01316 intersects 100%(117aa) with IS3_IS3_ORF2	e-value= 2.6e-66 complete sequence hit coverage= 100%, length is 273aa with 19 gaps, 0 stops, frame= Minus2 at 25929..26746 in the same strand
CDS	YP_001969960.1	putative ISXac3 like transposase intersects 94.43%(271aa) with IS3_IS150_ORF2	e-value= 1.6e-25 complete sequence hit coverage= 100%, length is 360aa with 39 gaps, 1 stops, frame= Minus2 at 25944..27022 in the same strand
misc_feature	no id	HMMPfam hit to PF00665, Integrase core domain, score 3.3e-32 intersects 95.03%(153aa) with IS3_IS150_ORF2	e-value= 1.6e-25 complete sequence hit coverage= 100%, length is 360aa with 39 gaps, 1 stops, frame= Minus2 at 25944..27022 in the same strand
misc_feature	no id	putative transposase OrfB; Reviewed; Region: PHA02517 intersects 100%(250aa) with IS3_IS150_ORF2	e-value= 1.6e-25 complete sequence hit coverage= 100%, length is 360aa with 39 gaps, 1 stops, frame= Minus2 at 25944..27022 in the same strand
misc_feature	no id	Integrase core domain; Region: rve; cl01316 intersects 100%(117aa) with IS3_IS150_ORF2	e-value= 1.6e-25 complete sequence hit coverage= 100%, length is 360aa with 39 gaps, 1 stops, frame= Minus2 at 25944..27022 in the same strand
CDS	YP_001969961.1	putative ISXac3 related insertion element intersects 89.58%(86aa) with IS3_IS150_ORF2	e-value= 1.6e-25 complete sequence hit coverage= 100%, length is 360aa with 39 gaps, 1 stops, frame= Minus2 at 25944..27022 in the same strand
misc_feature	no id	HMMPfam hit to PF01527, Transposase, score 1.1e-20 intersects 91.25%(73aa) with IS3_IS150_ORF2	e-value= 1.6e-25 complete sequence hit coverage= 100%, length is 360aa with 39 gaps, 1 stops, frame= Minus2 at 25944..27022 in the same strand
misc_feature	no id	Helix-turn-helix domain of Hin and related proteins, a family of DNA-binding domains unique to bacteria and represented by the Hin protein of Salmonella. The basic HTH domain is a simple fold comprised of three core helices that form a right-handed...; Region: HTH_Hin_like; cl01116 intersects 86.67%(65aa) with IS3_IS150_ORF2	e-value= 1.6e-25 complete sequence hit coverage= 100%, length is 360aa with 39 gaps, 1 stops, frame= Minus2 at 25944..27022 in the same strand
CDS	YP_001969960.1	putative ISXac3 like transposase intersects 92.57%(265aa) with IS3_IS3_ORF2	e-value= 2.2e-69 fragment hit coverage= 93.75%, length is 266aa with 8 gaps, 0 stops, frame= Minus2 at 25950..26746 in the same strand
misc_feature	no id	HMMPfam hit to PF00665, Integrase core domain, score 3.3e-32 intersects 93.79%(151aa) with IS3_IS3_ORF2	e-value= 2.2e-69 fragment hit coverage= 93.75%, length is 266aa with 8 gaps, 0 stops, frame= Minus2 at 25950..26746 in the same strand
misc_feature	no id	putative transposase OrfB; Reviewed; Region: PHA02517 intersects 100%(250aa) with IS3_IS3_ORF2	e-value= 2.2e-69 fragment hit coverage= 93.75%, length is 266aa with 8 gaps, 0 stops, frame= Minus2 at 25950..26746 in the same strand
misc_feature	no id	Integrase core domain; Region: rve; cl01316 intersects 100%(117aa) with IS3_IS3_ORF2	e-value= 2.2e-69 fragment hit coverage= 93.75%, length is 266aa with 8 gaps, 0 stops, frame= Minus2 at 25950..26746 in the same strand
CDS	YP_001969960.1	putative ISXac3 like transposase intersects 91.99%(264aa) with IS3_IS3_ORF1	e-value= 4e-75 fragment hit coverage= 94.06%, length is 265aa with 6 gaps, 0 stops, frame= Minus2 at 25965..26758 in the same strand

misc_feature no id HMMPfam hit to PF00665, Integrase core domain, score 3.3e-32 intersects 90.68%(146aa) with IS3_IS3_ORF1 e-value= 4e-75 fragment hit coverage= 94.06%, length is 265aa with 6 gaps, 0 stops, frame= Minus2 at 25965..26758 in the same strand
misc_feature no id putative transposase OrfB; Reviewed; Region: PHA02517 intersects 98.80%(247aa) with IS3_IS3_ORF1 e-value= 4e-75 fragment hit coverage= 94.06%, length is 265aa with 6 gaps, 0 stops, frame= Minus2 at 25965..26758 in the same strand
misc_feature no id Integrase core domain; Region: rve; cl01316 intersects 100%(117aa) with IS3_IS3_ORF1 e-value= 4e-75 fragment hit coverage= 94.06%, length is 265aa with 6 gaps, 0 stops, frame= Minus2 at 25965..26758 in the same strand
CDS YP_001969960.1 putative ISXac3 like transposase intersects 91.64%(263aa) with IS3_IS51_ORF1 e-value= 1.7e-73 fragment hit coverage= 90.88%, length is 265aa with 15 gaps, 0 stops, frame= Minus2 at 25968..26761 in the same strand
misc_feature no id HMMPfam hit to PF00665, Integrase core domain, score 3.3e-32 intersects 90.06%(145aa) with IS3_IS51_ORF1 e-value= 1.7e-73 fragment hit coverage= 90.88%, length is 265aa with 15 gaps, 0 stops, frame= Minus2 at 25968..26761 in the same strand
misc_feature no id putative transposase OrfB; Reviewed; Region: PHA02517 intersects 98.40%(246aa) with IS3_IS51_ORF1 e-value= 1.7e-73 fragment hit coverage= 90.88%, length is 265aa with 15 gaps, 0 stops, frame= Minus2 at 25968..26761 in the same strand
misc_feature no id Integrase core domain; Region: rve; cl01316 intersects 100%(117aa) with IS3_IS51_ORF1 e-value= 1.7e-73 fragment hit coverage= 90.88%, length is 265aa with 15 gaps, 0 stops, frame= Minus2 at 25968..26761 in the same strand
CDS YP_001969960.1 putative ISXac3 like transposase intersects 87.69%(251aa) with IS3_IS51_ORF2 e-value= 1.5e-73 fragment hit coverage= 83.28%, length is 252aa with 15 gaps, 0 stops, frame= Minus2 at 25971..26725 in the same strand
misc_feature no id HMMPfam hit to PF00665, Integrase core domain, score 3.3e-32 intersects 89.44%(144aa) with IS3_IS51_ORF2 e-value= 1.5e-73 fragment hit coverage= 83.28%, length is 252aa with 15 gaps, 0 stops, frame= Minus2 at 25971..26725 in the same strand
misc_feature no id putative transposase OrfB; Reviewed; Region: PHA02517 intersects 98%(245aa) with IS3_IS51_ORF2 e-value= 1.5e-73 fragment hit coverage= 83.28%, length is 252aa with 15 gaps, 0 stops, frame= Minus2 at 25971..26725 in the same strand
misc_feature no id Integrase core domain; Region: rve; cl01316 intersects 100%(117aa) with IS3_IS51_ORF2 e-value= 1.5e-73 fragment hit coverage= 83.28%, length is 252aa with 15 gaps, 0 stops, frame= Minus2 at 25971..26725 in the same strand
CDS YP_001969960.1 putative ISXac3 like transposase intersects 83.86%(240aa) with IS3_IS150_ORF1 e-value= 2.4e-37 fragment hit coverage= 80.39%, length is 241aa with 7 gaps, 0 stops, frame= Minus2 at 25974..26695 in the same strand
misc_feature no id HMMPfam hit to PF00665, Integrase core domain, score 3.3e-32 intersects 88.82%(143aa) with IS3_IS150_ORF1 e-value= 2.4e-37 fragment hit coverage= 80.39%, length is 241aa with 7 gaps, 0 stops, frame= Minus2 at 25974..26695 in the same strand
misc_feature no id putative transposase OrfB; Reviewed; Region: PHA02517 intersects 96.27%(240aa) with IS3_IS150_ORF1 e-value= 2.4e-37 fragment hit coverage= 80.39%, length is 241aa with 7 gaps, 0 stops, frame= Minus2 at 25974..26695 in the same strand
misc_feature no id Integrase core domain; Region: rve; cl01316 intersects 100%(117aa) with IS3_IS150_ORF1 e-value= 2.4e-37 fragment hit coverage= 80.39%, length is 241aa with 7 gaps, 0 stops, frame= Minus2 at 25974..26695 in the same strand
CDS YP_001969960.1 putative ISXac3 like transposase intersects 89.55%(257aa) with IS3_IS150_ORF2 e-value= 1.7e-58 fragment hit coverage= 68.89%, length is 258aa with 16 gaps, 0 stops, frame= Minus2 at 25986..26758 in the same strand
misc_feature no id HMMPfam hit to PF00665, Integrase core domain, score 3.3e-32 intersects 86.34%(139aa) with IS3_IS150_ORF2 e-value= 1.7e-58 fragment hit coverage= 68.89%, length is 258aa with 16 gaps, 0 stops, frame= Minus2 at 25986..26758 in the same strand
misc_feature no id putative transposase OrfB; Reviewed; Region: PHA02517 intersects 96%(240aa) with IS3_IS150_ORF2 e-value= 1.7e-58 fragment hit coverage= 68.89%, length is 258aa with 16 gaps, 0 stops, frame= Minus2 at 25986..26758 in the same strand
misc_feature no id Integrase core domain; Region: rve; cl01316 intersects 100%(117aa) with IS3_IS150_ORF2 e-value= 1.7e-58 fragment hit coverage= 68.89%, length is 258aa with 16 gaps, 0 stops, frame= Minus2 at 25986..26758 in the same strand

Class A

2.
CDS YP_001970283.1 putative transposase intersects 98.44%(63aa) with IS110 e-value= 6.9e-48 complete sequence hit coverage= 100%, length is 303aa with 113 gaps, 1 stops, frame= Minus2 at 369171..370078 in the same strand
repeat_region no id no product intersects 80.67%(296aa) with IS110 e-value= 6.9e-48 complete sequence hit coverage= 100%, length is 303aa with 113 gaps, 1 stops, frame= Minus2 at 369171..370078 in the opposite strand
misc_feature no id Transposase IS116/IS110/IS902 family; Region: Transposase_20; pfam02371 intersects 100%(25aa) with IS110 e-value= 6.9e-48 complete sequence hit coverage= 100%, length is 303aa with 113 gaps, 1 stops, frame= Minus2 at 369171..370078 in the same strand
CDS YP_001970284.1 putative transposase intersects 99.43%(231aa) with IS110 e-value= 6.9e-48 complete sequence hit coverage= 100%, length is 303aa with 113 gaps, 1 stops, frame= Minus2 at 369171..370078 in the same strand
misc_feature no id Transposase IS116/IS110/IS902 family; Region: Transposase_20; pfam02371 intersects 100%(45aa) with IS110 e-value= 6.9e-48 complete sequence hit coverage= 100%, length is 303aa with 113 gaps, 1 stops, frame= Minus2 at 369171..370078 in the same strand
misc_feature no id HMMPfam hit to PF01548, Transposase, score 2.6e-10 intersects 100%(99aa) with IS110 e-value= 6.9e-48 complete sequence hit coverage= 100%, length is 303aa with 113 gaps, 1 stops, frame= Minus2 at 369171..370078 in the same strand
misc_feature no id Transposase; Region: Transposase_9; pfam01548 intersects 100%(85aa) with IS110 e-value= 6.9e-48 complete sequence hit coverage= 100%, length is 303aa with 113 gaps, 1 stops, frame= Minus2 at 369171..370078 in the same strand
CDS YP_001970283.1 putative transposase intersects 81.25%(52aa) with IS110 e-value= 4.1e-51 fragment hit coverage= 96.15%, length is 292aa with 108 gaps, 1 stops, frame= Minus2 at 369204..370078 in the same strand
repeat_region no id no product intersects 79.40%(291aa) with IS110 e-value= 4.1e-51 fragment hit coverage= 96.15%, length is 292aa with 108 gaps, 1 stops, frame= Minus2 at 369204..370078 in the opposite strand
misc_feature no id Transposase IS116/IS110/IS902 family; Region: Transposase_20; pfam02371 intersects 100%(25aa) with IS110 e-value= 4.1e-51 fragment hit coverage= 96.15%, length is 292aa with 108 gaps, 1 stops, frame= Minus2 at 369204..370078 in the same strand
CDS YP_001970284.1 putative transposase intersects 99.43%(231aa) with IS110 e-value= 4.1e-51 fragment hit coverage= 96.15%, length is 292aa with 108 gaps, 1 stops, frame= Minus2 at 369204..370078 in the same strand
misc_feature no id Transposase IS116/IS110/IS902 family; Region: Transposase_20; pfam02371 intersects 100%(45aa) with IS110 e-value= 4.1e-51 fragment hit coverage= 96.15%, length is 292aa with 108 gaps, 1 stops, frame= Minus2 at 369204..370078 in the same strand
misc_feature no id HMMPfam hit to PF01548, Transposase, score 2.6e-10 intersects 100%(99aa) with IS110 e-value= 4.1e-51 fragment hit coverage= 96.15%, length is 292aa with 108 gaps, 1 stops, frame= Minus2 at 369204..370078 in the same strand
misc_feature no id Transposase; Region: Transposase_9; pfam01548 intersects 100%(85aa) with IS110 e-value= 4.1e-51 fragment hit coverage= 96.15%, length is 292aa with 108 gaps, 1 stops, frame= Minus2 at 369204..370078 in the same strand
Class A

3.
CDS YP_001970303.1 putative two-component LuxR family transcriptional regulator intersects 16.96%(38aa) with IS30 e-value= 5.7e-06 fragment hit coverage= 11.05%, length is 38aa with 1 gaps, 0 stops, frame= Plus1 at 385588..385701 in the same strand
misc_feature no id HMMPfam hit to PF00196, Bacterial regulatory proteins, luxR fami, score 6.9e-11 intersects 62.07%(36aa) with IS30 e-value= 5.7e-06 fragment hit coverage= 11.05%, length is 38aa with 1 gaps, 0 stops, frame= Plus1 at 385588..385701 in the same strand
misc_feature no id C-terminal DNA-binding domain of LuxR-like proteins. This domain contains a helix-turn-helix motif and binds DNA. Proteins belonging to this group are response regulators; some act as transcriptional activators, others as transcriptional repressors...; Region: LuxR_C_like; cd06170 intersects 58.82%(30aa) with IS30 e-value= 5.7e-06 fragment hit coverage= 11.05%, length is 38aa with 1 gaps, 0 stops, frame= Plus1 at 385588..385701 in the same strand

misc_feature no id DNA binding residues intersects 100%(1aa) with IS30 e-value= 5.7e-06 fragment hit coverage= 11.05%, length is 38aa with 1 gaps, 0 stops, frame= Plus1 at 385588..385701 in the same strand
misc_feature no id dimerization interface; other site intersects 100%(1aa) with IS30 e-value= 5.7e-06 fragment hit coverage= 11.05%, length is 38aa with 1 gaps, 0 stops, frame= Plus1 at 385588..385701 in the same strand
Class B

4.
repeat_region no id no product intersects 79.49%(292aa) with IS110 e-value= 1.4e-51 fragment hit coverage= 96.15%, length is 292aa with 108 gaps, 0 stops, frame= Plus1 at 478387..479262 in the opposite strand
CDS YP_001970376.1 putative transposase for IS element intersects 95.74%(292aa) with IS110 e-value= 1.4e-51 fragment hit coverage= 96.15%, length is 292aa with 108 gaps, 0 stops, frame= Plus1 at 478387..479262 in the same strand
misc_feature no id Transposase and inactivated derivatives [DNA replication, recombination, and repair]; Region: COG3547 intersects 100%(290aa) with IS110 e-value= 1.4e-51 fragment hit coverage= 96.15%, length is 292aa with 108 gaps, 0 stops, frame= Plus1 at 478387..479262 in the same strand
misc_feature no id HMMPfam hit to PF01548, Transposase, score 2.6e-10 intersects 100%(99aa) with IS110 e-value= 1.4e-51 fragment hit coverage= 96.15%, length is 292aa with 108 gaps, 0 stops, frame= Plus1 at 478387..479262 in the same strand
misc_feature no id Transposase; Region: Transposase_9; pfam01548 intersects 100%(85aa) with IS110 e-value= 1.4e-51 fragment hit coverage= 96.15%, length is 292aa with 108 gaps, 0 stops, frame= Plus1 at 478387..479262 in the same strand
misc_feature no id HMMPfam hit to PF02371, Transposase IS116/IS110/IS902 family, score 2e-24 intersects 100%(108aa) with IS110 e-value= 1.4e-51 fragment hit coverage= 96.15%, length is 292aa with 108 gaps, 0 stops, frame= Plus1 at 478387..479262 in the same strand
misc_feature no id Transposase IS116/IS110/IS902 family; Region: Transposase_20; pfam02371 intersects 100%(79aa) with IS110 e-value= 1.4e-51 fragment hit coverage= 96.15%, length is 292aa with 108 gaps, 0 stops, frame= Plus1 at 478387..479262 in the same strand
repeat_region no id no product intersects 80.76%(296aa) with IS110 e-value= 1.6e-50 complete sequence hit coverage= 100%, length is 313aa with 108 gaps, 1 stops, frame= Plus1 at 478387..479325 in the opposite strand
CDS YP_001970376.1 putative transposase for IS element intersects 99.67%(304aa) with IS110 e-value= 1.6e-50 complete sequence hit coverage= 100%, length is 313aa with 108 gaps, 1 stops, frame= Plus1 at 478387..479325 in the same strand
misc_feature no id Transposase and inactivated derivatives [DNA replication, recombination, and repair]; Region: COG3547 intersects 100%(290aa) with IS110 e-value= 1.6e-50 complete sequence hit coverage= 100%, length is 313aa with 108 gaps, 1 stops, frame= Plus1 at 478387..479325 in the same strand
misc_feature no id HMMPfam hit to PF01548, Transposase, score 2.6e-10 intersects 100%(99aa) with IS110 e-value= 1.6e-50 complete sequence hit coverage= 100%, length is 313aa with 108 gaps, 1 stops, frame= Plus1 at 478387..479325 in the same strand
misc_feature no id Transposase; Region: Transposase_9; pfam01548 intersects 100%(85aa) with IS110 e-value= 1.6e-50 complete sequence hit coverage= 100%, length is 313aa with 108 gaps, 1 stops, frame= Plus1 at 478387..479325 in the same strand
misc_feature no id HMMPfam hit to PF02371, Transposase IS116/IS110/IS902 family, score 2e-24 intersects 100%(108aa) with IS110 e-value= 1.6e-50 complete sequence hit coverage= 100%, length is 313aa with 108 gaps, 1 stops, frame= Plus1 at 478387..479325 in the same strand
misc_feature no id Transposase IS116/IS110/IS902 family; Region: Transposase_20; pfam02371 intersects 100%(79aa) with IS110 e-value= 1.6e-50 complete sequence hit coverage= 100%, length is 313aa with 108 gaps, 1 stops, frame= Plus1 at 478387..479325 in the same strand
Class A

5.
misc_feature no id putative helicase, pseudogene;This CDS has been interrupted by the insertion of a mobile element; similarity:fasta; with=UniProt:Q2ZSP4; Shewanella putrefaciens CN-32.; Helicase, C-terminal:Type III restriction enzyme, res subunit:DEAD/DEAH box helicase, N-terminal.; length=1020; id 38.596%; ungapped id 39.839%; E()=1.5e-90; 1026 aa overlap; query 13-1031; subject 15-1015 intersects 21.23%(302aa) with IS3_IS51_ORF2 e-value= 1.4e-134 complete sequence hit coverage= 100%, length is 303aa with 15 gaps, 0 stops, frame= Minus1 at 502981..503888 in the opposite strand

CDS YP_001970397.1 putative ISXac3 like transposase intersects 100%(284aa) with IS3_IS51_ORF2 e-value= 1.4e-134 complete sequence hit coverage= 100%, length is 303aa with 15 gaps, 0 stops, frame= Minus1 at 502981..503888 in the same strand
misc_feature no id putative transposase OrfB; Reviewed; Region: PHA02517 intersects 100%(263aa) with IS3_IS51_ORF2 e-value= 1.4e-134 complete sequence hit coverage= 100%, length is 303aa with 15 gaps, 0 stops, frame= Minus1 at 502981..503888 in the same strand
misc_feature no id HMMPfam hit to PF00665, Integrase core domain, score 9e-51 intersects 100%(161aa) with IS3_IS51_ORF2 e-value= 1.4e-134 complete sequence hit coverage= 100%, length is 303aa with 15 gaps, 0 stops, frame= Minus1 at 502981..503888 in the same strand
misc_feature no id Integrase core domain; Region: rve; cl01316 intersects 100%(117aa) with IS3_IS51_ORF2 e-value= 1.4e-134 complete sequence hit coverage= 100%, length is 303aa with 15 gaps, 0 stops, frame= Minus1 at 502981..503888 in the same strand
CDS YP_001970398.1 putative ISXac3 like transposase intersects 3.30%(3aa) with IS3_IS51_ORF2 e-value= 1.4e-134 complete sequence hit coverage= 100%, length is 303aa with 15 gaps, 0 stops, frame= Minus1 at 502981..503888 in the same strand
misc_feature no id putative helicase, pseudogene;This CDS has been interrupted by the insertion of a mobile element; similarity:fasta; with=UniProt:Q2ZSP4; Shewanella putrefaciens CN-32.; Helicase, C-terminal:Type III restriction enzyme, res subunit:DEAD/DEAH box helicase, N-terminal.; length=1020; id 38.596%; ungapped id 39.839%; E()=1.5e-90; 1026 aa overlap; query 13-1031; subject 15-1015 intersects 20.53%(292aa) with IS3_IS51_ORF1 e-value= 3.7e-108 complete sequence hit coverage= 100%, length is 293aa with 14 gaps, 1 stops, frame= Minus1 at 502993..503870 in the opposite strand
CDS YP_001970397.1 putative ISXac3 like transposase intersects 100%(284aa) with IS3_IS51_ORF1 e-value= 3.7e-108 complete sequence hit coverage= 100%, length is 293aa with 14 gaps, 1 stops, frame= Minus1 at 502993..503870 in the same strand
misc_feature no id putative transposase OrfB; Reviewed; Region: PHA02517 intersects 100%(263aa) with IS3_IS51_ORF1 e-value= 3.7e-108 complete sequence hit coverage= 100%, length is 293aa with 14 gaps, 1 stops, frame= Minus1 at 502993..503870 in the same strand
misc_feature no id HMMPfam hit to PF00665, Integrase core domain, score 9e-51 intersects 100%(161aa) with IS3_IS51_ORF1 e-value= 3.7e-108 complete sequence hit coverage= 100%, length is 293aa with 14 gaps, 1 stops, frame= Minus1 at 502993..503870 in the same strand
misc_feature no id Integrase core domain; Region: rve; cl01316 intersects 100%(117aa) with IS3_IS51_ORF1 e-value= 3.7e-108 complete sequence hit coverage= 100%, length is 293aa with 14 gaps, 1 stops, frame= Minus1 at 502993..503870 in the same strand
misc_feature no id putative helicase, pseudogene;This CDS has been interrupted by the insertion of a mobile element; similarity:fasta; with=UniProt:Q2ZSP4; Shewanella putrefaciens CN-32.; Helicase, C-terminal:Type III restriction enzyme, res subunit:DEAD/DEAH box helicase, N-terminal.; length=1020; id 38.596%; ungapped id 39.839%; E()=1.5e-90; 1026 aa overlap; query 13-1031; subject 15-1015 intersects 19.76%(281aa) with IS3_IS3_ORF2 e-value= 2e-102 complete sequence hit coverage= 100%, length is 282aa with 7 gaps, 1 stops, frame= Minus1 at 503011..503855 in the opposite strand
CDS YP_001970397.1 putative ISXac3 like transposase intersects 99.18%(281aa) with IS3_IS3_ORF2 e-value= 2e-102 complete sequence hit coverage= 100%, length is 282aa with 7 gaps, 1 stops, frame= Minus1 at 503011..503855 in the same strand
misc_feature no id putative transposase OrfB; Reviewed; Region: PHA02517 intersects 100%(263aa) with IS3_IS3_ORF2 e-value= 2e-102 complete sequence hit coverage= 100%, length is 282aa with 7 gaps, 1 stops, frame= Minus1 at 503011..503855 in the same strand
misc_feature no id HMMPfam hit to PF00665, Integrase core domain, score 9e-51 intersects 100%(161aa) with IS3_IS3_ORF2 e-value= 2e-102 complete sequence hit coverage= 100%, length is 282aa with 7 gaps, 1 stops, frame= Minus1 at 503011..503855 in the same strand
misc_feature no id Integrase core domain; Region: rve; cl01316 intersects 100%(117aa) with IS3_IS3_ORF2 e-value= 2e-102 complete sequence hit coverage= 100%, length is 282aa with 7 gaps, 1 stops, frame= Minus1 at 503011..503855 in the same strand
misc_feature no id putative helicase, pseudogene;This CDS has been interrupted by the insertion of a mobile element; similarity:fasta; with=UniProt:Q2ZSP4; Shewanella putrefaciens CN-32.; Helicase, C-terminal:Type III restriction enzyme, res subunit:DEAD/DEAH box helicase, N-terminal.; length=1020; id 38.596%; ungapped id 39.839%; E()=1.5e-90; 1026 aa overlap; query 13-1031; subject 15-1015 intersects 19.34%(275aa) with IS3_IS150_ORF2 e-value= 1.9e-74 fragment hit coverage= 73.52%, length is 276aa with 11 gaps, 0 stops, frame= Minus1 at 503017..503843 in the opposite strand

CDS YP_001970397.1 putative ISXac3 like transposase intersects 97.07%(275aa) with IS3_IS150_ORF2 e-value= 1.9e-74 fragment hit coverage= 73.52%, length is 276aa with 11 gaps, 0 stops, frame= Minus1 at 503017..503843 in the same strand
misc_feature no id putative transposase OrfB; Reviewed; Region: PHA02517 intersects 100%(263aa) with IS3_IS150_ORF2 e-value= 1.9e-74 fragment hit coverage= 73.52%, length is 276aa with 11 gaps, 0 stops, frame= Minus1 at 503017..503843 in the same strand
misc_feature no id HMMPfam hit to PF00665, Integrase core domain, score 9e-51 intersects 100%(161aa) with IS3_IS150_ORF2 e-value= 1.9e-74 fragment hit coverage= 73.52%, length is 276aa with 11 gaps, 0 stops, frame= Minus1 at 503017..503843 in the same strand
misc_feature no id Integrase core domain; Region: rve; cl01316 intersects 100%(117aa) with IS3_IS150_ORF2 e-value= 1.9e-74 fragment hit coverage= 73.52%, length is 276aa with 11 gaps, 0 stops, frame= Minus1 at 503017..503843 in the same strand
misc_feature no id putative helicase, pseudogene;This CDS has been interrupted by the insertion of a mobile element; similarity:fasta; with=UniProt:Q2ZSP4; Shewanella putrefaciens CN-32.; Helicase, C-terminal:Type III restriction enzyme, res subunit:DEAD/DEAH box helicase, N-terminal.; length=1020; id 38.596%; ungapped id 39.839%; E()=1.5e-90; 1026 aa overlap; query 13-1031; subject 15-1015 intersects 25.94%(369aa) with IS3_IS150_ORF2 e-value= 1.2e-45 complete sequence hit coverage= 100%, length is 370aa with 21 gaps, 4 stops, frame= Minus1 at 503017..504125 in the opposite strand
CDS YP_001970397.1 putative ISXac3 like transposase intersects 99.30%(282aa) with IS3_IS150_ORF2 e-value= 1.2e-45 complete sequence hit coverage= 100%, length is 370aa with 21 gaps, 4 stops, frame= Minus1 at 503017..504125 in the same strand
misc_feature no id putative transposase OrfB; Reviewed; Region: PHA02517 intersects 100%(263aa) with IS3_IS150_ORF2 e-value= 1.2e-45 complete sequence hit coverage= 100%, length is 370aa with 21 gaps, 4 stops, frame= Minus1 at 503017..504125 in the same strand
misc_feature no id HMMPfam hit to PF00665, Integrase core domain, score 9e-51 intersects 100%(161aa) with IS3_IS150_ORF2 e-value= 1.2e-45 complete sequence hit coverage= 100%, length is 370aa with 21 gaps, 4 stops, frame= Minus1 at 503017..504125 in the same strand
misc_feature no id Integrase core domain; Region: rve; cl01316 intersects 100%(117aa) with IS3_IS150_ORF2 e-value= 1.2e-45 complete sequence hit coverage= 100%, length is 370aa with 21 gaps, 4 stops, frame= Minus1 at 503017..504125 in the same strand
CDS YP_001970398.1 putative ISXac3 like transposase intersects 90.11%(82aa) with IS3_IS150_ORF2 e-value= 1.2e-45 complete sequence hit coverage= 100%, length is 370aa with 21 gaps, 4 stops, frame= Minus1 at 503017..504125 in the same strand
misc_feature no id HMMPfam hit to PF01527, Transposase, score 1.1e-21 intersects 91.03%(71aa) with IS3_IS150_ORF2 e-value= 1.2e-45 complete sequence hit coverage= 100%, length is 370aa with 21 gaps, 4 stops, frame= Minus1 at 503017..504125 in the same strand
misc_feature no id Helix-turn-helix domain of Hin and related proteins, a family of DNA-binding domains unique to bacteria and represented by the Hin protein of Salmonella. The basic HTH domain is a simple fold comprised of three core helices that form a right-handed...; Region: HTH_Hin_like; cl01116 intersects 91.30%(63aa) with IS3_IS150_ORF2 e-value= 1.2e-45 complete sequence hit coverage= 100%, length is 370aa with 21 gaps, 4 stops, frame= Minus1 at 503017..504125 in the same strand
misc_feature no id putative helicase, pseudogene;This CDS has been interrupted by the insertion of a mobile element; similarity:fasta; with=UniProt:Q2ZSP4; Shewanella putrefaciens CN-32.; Helicase, C-terminal:Type III restriction enzyme, res subunit:DEAD/DEAH box helicase, N-terminal.; length=1020; id 38.596%; ungapped id 39.839%; E()=1.5e-90; 1026 aa overlap; query 13-1031; subject 15-1015 intersects 20.53%(292aa) with IS3_IS150_ORF1 e-value= 5.1e-57 fragment hit coverage= 97.06%, length is 293aa with 7 gaps, 0 stops, frame= Minus1 at 503020..503897 in the opposite strand
CDS YP_001970397.1 putative ISXac3 like transposase intersects 98.94%(281aa) with IS3_IS150_ORF1 e-value= 5.1e-57 fragment hit coverage= 97.06%, length is 293aa with 7 gaps, 0 stops, frame= Minus1 at 503020..503897 in the same strand
misc_feature no id putative transposase OrfB; Reviewed; Region: PHA02517 intersects 100%(263aa) with IS3_IS150_ORF1 e-value= 5.1e-57 fragment hit coverage= 97.06%, length is 293aa with 7 gaps, 0 stops, frame= Minus1 at 503020..503897 in the same strand
misc_feature no id HMMPfam hit to PF00665, Integrase core domain, score 9e-51 intersects 100%(161aa) with IS3_IS150_ORF1 e-value= 5.1e-57 fragment hit coverage= 97.06%, length is 293aa with 7 gaps, 0 stops, frame= Minus1 at 503020..503897 in the same strand

misc_feature no id Integrase core domain; Region: rve; cl01316 intersects 100%(117aa) with IS3_IS150_ORF1 e-value= 5.1e-57 fragment hit coverage= 97.06%, length is 293aa with 7 gaps, 0 stops, frame= Minus1 at 503020..503897 in the same strand
CDS YP_001970398.1 putative ISXac3 like transposase intersects 6.59%(6aa) with IS3_IS150_ORF1 e-value= 5.1e-57 fragment hit coverage= 97.06%, length is 293aa with 7 gaps, 0 stops, frame= Minus1 at 503020..503897 in the same strand
misc_feature no id putative helicase, pseudogene;This CDS has been interrupted by the insertion of a mobile element; similarity:fasta; with=UniProt:Q2ZSP4; Shewanella putrefaciens CN-32.; Helicase, C-terminal:Type III restriction enzyme, res subunit:DEAD/DEAH box helicase, N-terminal.; length=1020; id 38.596%; ungapped id 39.839%; E()=1.5e-90; 1026 aa overlap; query 13-1031; subject 15-1015 intersects 21.16%(301aa) with IS3_IS150_ORF1 e-value= 1.4e-57 complete sequence hit coverage= 100%, length is 302aa with 7 gaps, 0 stops, frame= Minus1 at 503020..503924 in the opposite strand
CDS YP_001970397.1 putative ISXac3 like transposase intersects 98.94%(281aa) with IS3_IS150_ORF1 e-value= 1.4e-57 complete sequence hit coverage= 100%, length is 302aa with 7 gaps, 0 stops, frame= Minus1 at 503020..503924 in the same strand
misc_feature no id putative transposase OrfB; Reviewed; Region: PHA02517 intersects 100%(263aa) with IS3_IS150_ORF1 e-value= 1.4e-57 complete sequence hit coverage= 100%, length is 302aa with 7 gaps, 0 stops, frame= Minus1 at 503020..503924 in the same strand
misc_feature no id HMMPfam hit to PF00665, Integrase core domain, score 9e-51 intersects 100%(161aa) with IS3_IS150_ORF1 e-value= 1.4e-57 complete sequence hit coverage= 100%, length is 302aa with 7 gaps, 0 stops, frame= Minus1 at 503020..503924 in the same strand
misc_feature no id Integrase core domain; Region: rve; cl01316 intersects 100%(117aa) with IS3_IS150_ORF1 e-value= 1.4e-57 complete sequence hit coverage= 100%, length is 302aa with 7 gaps, 0 stops, frame= Minus1 at 503020..503924 in the same strand
CDS YP_001970398.1 putative ISXac3 like transposase intersects 16.48%(15aa) with IS3_IS150_ORF1 e-value= 1.4e-57 complete sequence hit coverage= 100%, length is 302aa with 7 gaps, 0 stops, frame= Minus1 at 503020..503924 in the same strand
misc_feature no id HMMPfam hit to PF01527, Transposase, score 1.1e-21 intersects 5.13%(4aa) with IS3_IS150_ORF1 e-value= 1.4e-57 complete sequence hit coverage= 100%, length is 302aa with 7 gaps, 0 stops, frame= Minus1 at 503020..503924 in the same strand
misc_feature no id putative helicase, pseudogene;This CDS has been interrupted by the insertion of a mobile element; similarity:fasta; with=UniProt:Q2ZSP4; Shewanella putrefaciens CN-32.; Helicase, C-terminal:Type III restriction enzyme, res subunit:DEAD/DEAH box helicase, N-terminal.; length=1020; id 38.596%; ungapped id 39.839%; E()=1.5e-90; 1026 aa overlap; query 13-1031; subject 15-1015 intersects 19.13%(272aa) with IS3_IS51_ORF2 e-value= 7.1e-152 fragment hit coverage= 90.22%, length is 273aa with 13 gaps, 0 stops, frame= Minus1 at 503023..503840 in the opposite strand
CDS YP_001970397.1 putative ISXac3 like transposase intersects 96.01%(272aa) with IS3_IS51_ORF2 e-value= 7.1e-152 fragment hit coverage= 90.22%, length is 273aa with 13 gaps, 0 stops, frame= Minus1 at 503023..503840 in the same strand
misc_feature no id putative transposase OrfB; Reviewed; Region: PHA02517 intersects 100%(263aa) with IS3_IS51_ORF2 e-value= 7.1e-152 fragment hit coverage= 90.22%, length is 273aa with 13 gaps, 0 stops, frame= Minus1 at 503023..503840 in the same strand
misc_feature no id HMMPfam hit to PF00665, Integrase core domain, score 9e-51 intersects 100%(161aa) with IS3_IS51_ORF2 e-value= 7.1e-152 fragment hit coverage= 90.22%, length is 273aa with 13 gaps, 0 stops, frame= Minus1 at 503023..503840 in the same strand
misc_feature no id Integrase core domain; Region: rve; cl01316 intersects 100%(117aa) with IS3_IS51_ORF2 e-value= 7.1e-152 fragment hit coverage= 90.22%, length is 273aa with 13 gaps, 0 stops, frame= Minus1 at 503023..503840 in the same strand
misc_feature no id putative helicase, pseudogene;This CDS has been interrupted by the insertion of a mobile element; similarity:fasta; with=UniProt:Q2ZSP4; Shewanella putrefaciens CN-32.; Helicase, C-terminal:Type III restriction enzyme, res subunit:DEAD/DEAH box helicase, N-terminal.; length=1020; id 38.596%; ungapped id 39.839%; E()=1.5e-90; 1026 aa overlap; query 13-1031; subject 15-1015 intersects 19.76%(281aa) with IS3_IS3_ORF1 e-value= 4.9e-95 complete sequence hit coverage= 100%, length is 282aa with 6 gaps, 0 stops, frame= Minus1 at 503023..503867 in the opposite strand
CDS YP_001970397.1 putative ISXac3 like transposase intersects 98.59%(280aa) with IS3_IS3_ORF1 e-value= 4.9e-95 complete sequence hit coverage= 100%, length is 282aa with 6 gaps, 0 stops, frame= Minus1 at 503023..503867 in the same strand

misc_feature no id putative transposase OrfB; Reviewed; Region: PHA02517 intersects 100%(263aa) with IS3_IS3_ORF1 e-value= 4.9e-95 complete sequence hit coverage= 100%, length is 282aa with 6 gaps, 0 stops, frame= Minus1 at 503023..503867 in the same strand
misc_feature no id HMMPfam hit to PF00665, Integrase core domain, score 9e-51 intersects 100%(161aa) with IS3_IS3_ORF1 e-value= 4.9e-95 complete sequence hit coverage= 100%, length is 282aa with 6 gaps, 0 stops, frame= Minus1 at 503023..503867 in the same strand
misc_feature no id Integrase core domain; Region: rve; cl01316 intersects 100%(117aa) with IS3_IS3_ORF1 e-value= 4.9e-95 complete sequence hit coverage= 100%, length is 282aa with 6 gaps, 0 stops, frame= Minus1 at 503023..503867 in the same strand
misc_feature no id putative helicase, pseudogene;This CDS has been interrupted by the insertion of a mobile element; similarity:fasta; with=UniProt:Q2ZSP4; Shewanella putrefaciens CN-32.; Helicase, C-terminal:Type III restriction enzyme, res subunit:DEAD/DEAH box helicase, N-terminal.; length=1020; id 38.596%; ungapped id 39.839%; E()=1.5e-90; 1026 aa overlap; query 13-1031; subject 15-1015 intersects 19.76%(281aa) with IS3_IS51_ORF1 e-value= 3.9e-110 fragment hit coverage= 96.42%, length is 282aa with 14 gaps, 0 stops, frame= Minus1 at 503026..503870 in the opposite strand
CDS YP_001970397.1 putative ISXac3 like transposase intersects 98.24%(279aa) with IS3_IS51_ORF1 e-value= 3.9e-110 fragment hit coverage= 96.42%, length is 282aa with 14 gaps, 0 stops, frame= Minus1 at 503026..503870 in the same strand
misc_feature no id putative transposase OrfB; Reviewed; Region: PHA02517 intersects 100%(263aa) with IS3_IS51_ORF1 e-value= 3.9e-110 fragment hit coverage= 96.42%, length is 282aa with 14 gaps, 0 stops, frame= Minus1 at 503026..503870 in the same strand
misc_feature no id HMMPfam hit to PF00665, Integrase core domain, score 9e-51 intersects 100%(161aa) with IS3_IS51_ORF1 e-value= 3.9e-110 fragment hit coverage= 96.42%, length is 282aa with 14 gaps, 0 stops, frame= Minus1 at 503026..503870 in the same strand
misc_feature no id Integrase core domain; Region: rve; cl01316 intersects 100%(117aa) with IS3_IS51_ORF1 e-value= 3.9e-110 fragment hit coverage= 96.42%, length is 282aa with 14 gaps, 0 stops, frame= Minus1 at 503026..503870 in the same strand
misc_feature no id putative helicase, pseudogene;This CDS has been interrupted by the insertion of a mobile element; similarity:fasta; with=UniProt:Q2ZSP4; Shewanella putrefaciens CN-32.; Helicase, C-terminal:Type III restriction enzyme, res subunit:DEAD/DEAH box helicase, N-terminal.; length=1020; id 38.596%; ungapped id 39.839%; E()=1.5e-90; 1026 aa overlap; query 13-1031; subject 15-1015 intersects 2.99%(42aa) with IS3_IS51_ORF1 e-value= 3.2e-08 fragment hit coverage= 14.66%, length is 43aa with 2 gaps, 1 stops, frame= Minus2 at 503862..503989 in the opposite strand
CDS YP_001970397.1 putative ISXac3 like transposase intersects 0.12%(0aa) with IS3_IS51_ORF1 e-value= 3.2e-08 fragment hit coverage= 14.66%, length is 43aa with 2 gaps, 1 stops, frame= Minus2 at 503862..503989 in the same strand
CDS YP_001970398.1 putative ISXac3 like transposase intersects 40.29%(36aa) with IS3_IS51_ORF1 e-value= 3.2e-08 fragment hit coverage= 14.66%, length is 43aa with 2 gaps, 1 stops, frame= Minus2 at 503862..503989 in the same strand
misc_feature no id HMMPfam hit to PF01527, Transposase, score 1.1e-21 intersects 32.91%(25aa) with IS3_IS51_ORF1 e-value= 3.2e-08 fragment hit coverage= 14.66%, length is 43aa with 2 gaps, 1 stops, frame= Minus2 at 503862..503989 in the same strand
misc_feature no id Helix-turn-helix domain of Hin and related proteins, a family of DNA-binding domains unique to bacteria and represented by the Hin protein of Salmonella. The basic HTH domain is a simple fold comprised of three core helices that form a right-handed...; Region: HTH_Hin_like; cl01116 intersects 25.60%(17aa) with IS3_IS51_ORF1 e-value= 3.2e-08 fragment hit coverage= 14.66%, length is 43aa with 2 gaps, 1 stops, frame= Minus2 at 503862..503989 in the same strand
Class A

6.
misc_feature no id putative helicase, pseudogene;This CDS has been interrupted by the insertion of a mobile element; similarity:fasta; with=UniProt:Q2ZSP4; Shewanella putrefaciens CN-32.; Helicase, C-terminal:Type III restriction enzyme, res subunit:DEAD/DEAH box helicase, N-terminal.; length=1020; id 38.596%; ungapped id 39.839%; E()=1.5e-90; 1026 aa overlap; query 13-1031; subject 15-1015 intersects 3.34%(47aa) with IS3_IS407_ORF1 e-value= 7.7e-06 fragment hit coverage= 55.81%, length is 48aa with 0 gaps, 0 stops, frame= Minus2 at 504000..504142 in the opposite strand

CDS YP_001970398.1 putative ISXac3 like transposase intersects 52.38%(47aa) with IS3_IS407_ORF1 e-value= 7.7e-06 fragment hit coverage= 55.81%, length is 48aa with 0 gaps, 0 stops, frame= Minus2 at 504000..504142 in the same strand
misc_feature no id HMMPfam hit to PF01527, Transposase, score 1.1e-21 intersects 61.11%(47aa) with IS3_IS407_ORF1 e-value= 7.7e-06 fragment hit coverage= 55.81%, length is 48aa with 0 gaps, 0 stops, frame= Minus2 at 504000..504142 in the same strand
misc_feature no id Helix-turn-helix domain of Hin and related proteins, a family of DNA-binding domains unique to bacteria and represented by the Hin protein of Salmonella. The basic HTH domain is a simple fold comprised of three core helices that form a right-handed...; Region: HTH_Hin_like; cl01116 intersects 69.08%(47aa) with IS3_IS407_ORF1 e-value= 7.7e-06 fragment hit coverage= 55.81%, length is 48aa with 0 gaps, 0 stops, frame= Minus2 at 504000..504142 in the same strand
Class A

7.
CDS YP_001970423.1 putative transposase intersects 98.31%(309aa) with IS481 e-value= 1.2e-93 complete sequence hit coverage= 100%, length is 314aa with 38 gaps, 1 stops, frame= Minus3 at 525887..526827 in the same strand
misc_feature no id HMMPfam hit to PF00665, Integrase core domain, score 4.9e-39 intersects 100%(178aa) with IS481 e-value= 1.2e-93 complete sequence hit coverage= 100%, length is 314aa with 38 gaps, 1 stops, frame= Minus3 at 525887..526827 in the same strand
misc_feature no id Transposase and inactivated derivatives [DNA replication, recombination, and repair]; Region: Tra5; COG2801 intersects 100%(227aa) with IS481 e-value= 1.2e-93 complete sequence hit coverage= 100%, length is 314aa with 38 gaps, 1 stops, frame= Minus3 at 525887..526827 in the same strand
misc_feature no id Integrase core domain; Region: rve; cl01316 intersects 100%(134aa) with IS481 e-value= 1.2e-93 complete sequence hit coverage= 100%, length is 314aa with 38 gaps, 1 stops, frame= Minus3 at 525887..526827 in the same strand
misc_feature no id Transposase and inactivated derivatives [DNA replication, recombination, and repair]; Region: COG3415 intersects 100%(100aa) with IS481 e-value= 1.2e-93 complete sequence hit coverage= 100%, length is 314aa with 38 gaps, 1 stops, frame= Minus3 at 525887..526827 in the same strand
CDS YP_001970423.1 putative transposase intersects 96.72%(304aa) with IS481 e-value= 6.1e-93 fragment hit coverage= 97.44%, length is 305aa with 38 gaps, 0 stops, frame= Minus3 at 525914..526827 in the same strand
misc_feature no id HMMPfam hit to PF00665, Integrase core domain, score 4.9e-39 intersects 100%(178aa) with IS481 e-value= 6.1e-93 fragment hit coverage= 97.44%, length is 305aa with 38 gaps, 0 stops, frame= Minus3 at 525914..526827 in the same strand
misc_feature no id Transposase and inactivated derivatives [DNA replication, recombination, and repair]; Region: Tra5; COG2801 intersects 100%(227aa) with IS481 e-value= 6.1e-93 fragment hit coverage= 97.44%, length is 305aa with 38 gaps, 0 stops, frame= Minus3 at 525914..526827 in the same strand
misc_feature no id Integrase core domain; Region: rve; cl01316 intersects 100%(134aa) with IS481 e-value= 6.1e-93 fragment hit coverage= 97.44%, length is 305aa with 38 gaps, 0 stops, frame= Minus3 at 525914..526827 in the same strand
misc_feature no id Transposase and inactivated derivatives [DNA replication, recombination, and repair]; Region: COG3415 intersects 100%(100aa) with IS481 e-value= 6.1e-93 fragment hit coverage= 97.44%, length is 305aa with 38 gaps, 0 stops, frame= Minus3 at 525914..526827 in the same strand
CDS YP_001970423.1 putative transposase intersects 28.15%(88aa) with IS3_IS150_ORF2 e-value= 1.8e-10 fragment hit coverage= 23.39%, length is 89aa with 2 gaps, 0 stops, frame= Minus3 at 525941..526206 in the same strand
misc_feature no id HMMPfam hit to PF00665, Integrase core domain, score 4.9e-39 intersects 49.81%(88aa) with IS3_IS150_ORF2 e-value= 1.8e-10 fragment hit coverage= 23.39%, length is 89aa with 2 gaps, 0 stops, frame= Minus3 at 525941..526206 in the same strand
misc_feature no id Transposase and inactivated derivatives [DNA replication, recombination, and repair]; Region: Tra5; COG2801 intersects 39.06%(88aa) with IS3_IS150_ORF2 e-value= 1.8e-10 fragment hit coverage= 23.39%, length is 89aa with 2 gaps, 0 stops, frame= Minus3 at 525941..526206 in the same strand
misc_feature no id Integrase core domain; Region: rve; cl01316 intersects 37.81%(50aa) with IS3_IS150_ORF2 e-value= 1.8e-10 fragment hit coverage= 23.39%, length is 89aa with 2 gaps, 0 stops, frame= Minus3 at 525941..526206 in the same strand

CDS YP_001970423.1 putative transposase intersects 7.20%(22aa) with IS3_IS407_ORF2 e-value= 4.1e-07 fragment hit coverage= 6.69%, length is 23aa with 0 gaps, 0 stops, frame= Minus3 at 525944..526011 in the same strand
misc_feature no id HMMPfam hit to PF00665, Integrase core domain, score 4.9e-39 intersects 12.73%(22aa) with IS3_IS407_ORF2 e-value= 4.1e-07 fragment hit coverage= 6.69%, length is 23aa with 0 gaps, 0 stops, frame= Minus3 at 525944..526011 in the same strand
misc_feature no id Transposase and inactivated derivatives [DNA replication, recombination, and repair]; Region: Tra5; COG2801 intersects 9.99%(22aa) with IS3_IS407_ORF2 e-value= 4.1e-07 fragment hit coverage= 6.69%, length is 23aa with 0 gaps, 0 stops, frame= Minus3 at 525944..526011 in the same strand
Class A

8.
CDS YP_001970461.1 putative transposase-related protein intersects 98.41%(310aa) with IS481 e-value= 1.3e-95 complete sequence hit coverage= 100%, length is 314aa with 38 gaps, 1 stops, frame= Plus1 at 572224..573165 in the same strand
misc_feature no id Transposase and inactivated derivatives [DNA replication, recombination, and repair]; Region: COG3415 intersects 100%(100aa) with IS481 e-value= 1.3e-95 complete sequence hit coverage= 100%, length is 314aa with 38 gaps, 1 stops, frame= Plus1 at 572224..573165 in the same strand
misc_feature no id Transposase and inactivated derivatives [DNA replication, recombination, and repair]; Region: Tra5; COG2801 intersects 100%(227aa) with IS481 e-value= 1.3e-95 complete sequence hit coverage= 100%, length is 314aa with 38 gaps, 1 stops, frame= Plus1 at 572224..573165 in the same strand
misc_feature no id HMMPfam hit to PF00665, Integrase core domain, score 4.9e-39 intersects 100%(178aa) with IS481 e-value= 1.3e-95 complete sequence hit coverage= 100%, length is 314aa with 38 gaps, 1 stops, frame= Plus1 at 572224..573165 in the same strand
misc_feature no id Integrase core domain; Region: rve; cl01316 intersects 100%(134aa) with IS481 e-value= 1.3e-95 complete sequence hit coverage= 100%, length is 314aa with 38 gaps, 1 stops, frame= Plus1 at 572224..573165 in the same strand
repeat_region no id no product intersects 25.32%(6aa) with IS481 e-value= 1.3e-95 complete sequence hit coverage= 100%, length is 314aa with 38 gaps, 1 stops, frame= Plus1 at 572224..573165 in the same strand
CDS YP_001970461.1 putative transposase-related protein intersects 28.25%(89aa) with IS3_IS150_ORF2 e-value= 1.8e-10 fragment hit coverage= 23.39%, length is 89aa with 2 gaps, 0 stops, frame= Plus1 at 572845..573111 in the same strand
misc_feature no id Transposase and inactivated derivatives [DNA replication, recombination, and repair]; Region: Tra5; COG2801 intersects 39.21%(89aa) with IS3_IS150_ORF2 e-value= 1.8e-10 fragment hit coverage= 23.39%, length is 89aa with 2 gaps, 0 stops, frame= Plus1 at 572845..573111 in the same strand
misc_feature no id HMMPfam hit to PF00665, Integrase core domain, score 4.9e-39 intersects 50%(89aa) with IS3_IS150_ORF2 e-value= 1.8e-10 fragment hit coverage= 23.39%, length is 89aa with 2 gaps, 0 stops, frame= Plus1 at 572845..573111 in the same strand
misc_feature no id Integrase core domain; Region: rve; cl01316 intersects 38.06%(51aa) with IS3_IS150_ORF2 e-value= 1.8e-10 fragment hit coverage= 23.39%, length is 89aa with 2 gaps, 0 stops, frame= Plus1 at 572845..573111 in the same strand
CDS YP_001970461.1 putative transposase-related protein intersects 7.30%(23aa) with IS3_IS407_ORF2 e-value= 4.1e-07 fragment hit coverage= 6.69%, length is 23aa with 0 gaps, 0 stops, frame= Plus1 at 573040..573108 in the same strand
misc_feature no id Transposase and inactivated derivatives [DNA replication, recombination, and repair]; Region: Tra5; COG2801 intersects 10.13%(23aa) with IS3_IS407_ORF2 e-value= 4.1e-07 fragment hit coverage= 6.69%, length is 23aa with 0 gaps, 0 stops, frame= Plus1 at 573040..573108 in the same strand
misc_feature no id HMMPfam hit to PF00665, Integrase core domain, score 4.9e-39 intersects 12.92%(23aa) with IS3_IS407_ORF2 e-value= 4.1e-07 fragment hit coverage= 6.69%, length is 23aa with 0 gaps, 0 stops, frame= Plus1 at 573040..573108 in the same strand
Class A

9.
repeat_region no id no product intersects 67.53%(305aa) with IS110 e-value= 3.4e-46 complete sequence hit coverage= 100%, length is 306aa with 110 gaps, 1 stops, frame= Minus2 at 686589..687505 in the opposite strand

CDS YP_001970563.1 putative transposase protein intersects 99.56%(303aa) with IS110 e-value= 3.4e-46 complete sequence hit coverage= 100%, length is 306aa with 110 gaps, 1 stops, frame= Minus2 at 686589..687505 in the same strand
misc_feature no id Transposase and inactivated derivatives [DNA replication, recombination, and repair]; Region: COG3547 intersects 100%(292aa) with IS110 e-value= 3.4e-46 complete sequence hit coverage= 100%, length is 306aa with 110 gaps, 1 stops, frame= Minus2 at 686589..687505 in the same strand
misc_feature no id Transposase IS116/IS110/IS902 family; Region: Transposase_20; pfam02371 intersects 100%(84aa) with IS110 e-value= 3.4e-46 complete sequence hit coverage= 100%, length is 306aa with 110 gaps, 1 stops, frame= Minus2 at 686589..687505 in the same strand
misc_feature no id HMMPfam hit to PF02371, Transposase IS116/IS110/IS902 family, score 4.3e-27 intersects 100%(108aa) with IS110 e-value= 3.4e-46 complete sequence hit coverage= 100%, length is 306aa with 110 gaps, 1 stops, frame= Minus2 at 686589..687505 in the same strand
misc_feature no id HMMPfam hit to PF01548, Transposase, score 1.8e-06 intersects 100%(98aa) with IS110 e-value= 3.4e-46 complete sequence hit coverage= 100%, length is 306aa with 110 gaps, 1 stops, frame= Minus2 at 686589..687505 in the same strand
misc_feature no id Transposase; Region: Transposase_9; pfam01548 intersects 100%(79aa) with IS110 e-value= 3.4e-46 complete sequence hit coverage= 100%, length is 306aa with 110 gaps, 1 stops, frame= Minus2 at 686589..687505 in the same strand
repeat_region no id no product intersects 65.10%(294aa) with IS110 e-value= 1e-48 fragment hit coverage= 96.88%, length is 295aa with 108 gaps, 0 stops, frame= Minus2 at 686622..687505 in the opposite strand
CDS YP_001970563.1 putative transposase protein intersects 96.61%(294aa) with IS110 e-value= 1e-48 fragment hit coverage= 96.88%, length is 295aa with 108 gaps, 0 stops, frame= Minus2 at 686622..687505 in the same strand
misc_feature no id Transposase and inactivated derivatives [DNA replication, recombination, and repair]; Region: COG3547 intersects 100%(292aa) with IS110 e-value= 1e-48 fragment hit coverage= 96.88%, length is 295aa with 108 gaps, 0 stops, frame= Minus2 at 686622..687505 in the same strand
misc_feature no id Transposase IS116/IS110/IS902 family; Region: Transposase_20; pfam02371 intersects 100%(84aa) with IS110 e-value= 1e-48 fragment hit coverage= 96.88%, length is 295aa with 108 gaps, 0 stops, frame= Minus2 at 686622..687505 in the same strand
misc_feature no id HMMPfam hit to PF02371, Transposase IS116/IS110/IS902 family, score 4.3e-27 intersects 100%(108aa) with IS110 e-value= 1e-48 fragment hit coverage= 96.88%, length is 295aa with 108 gaps, 0 stops, frame= Minus2 at 686622..687505 in the same strand
misc_feature no id HMMPfam hit to PF01548, Transposase, score 1.8e-06 intersects 100%(98aa) with IS110 e-value= 1e-48 fragment hit coverage= 96.88%, length is 295aa with 108 gaps, 0 stops, frame= Minus2 at 686622..687505 in the same strand
misc_feature no id Transposase; Region: Transposase_9; pfam01548 intersects 100%(79aa) with IS110 e-value= 1e-48 fragment hit coverage= 96.88%, length is 295aa with 108 gaps, 0 stops, frame= Minus2 at 686622..687505 in the same strand
Class A

10.
repeat_region no id no product intersects 67.53%(305aa) with IS110 e-value= 2.5e-43 complete sequence hit coverage= 100%, length is 306aa with 110 gaps, 1 stops, frame= Minus2 at 729681..730597 in the opposite strand
CDS YP_001970593.1 putative transposase intersects 99.56%(303aa) with IS110 e-value= 2.5e-43 complete sequence hit coverage= 100%, length is 306aa with 110 gaps, 1 stops, frame= Minus2 at 729681..730597 in the same strand
misc_feature no id Transposase and inactivated derivatives [DNA replication, recombination, and repair]; Region: COG3547 intersects 100%(292aa) with IS110 e-value= 2.5e-43 complete sequence hit coverage= 100%, length is 306aa with 110 gaps, 1 stops, frame= Minus2 at 729681..730597 in the same strand
misc_feature no id Transposase IS116/IS110/IS902 family; Region: Transposase_20; pfam02371 intersects 100%(84aa) with IS110 e-value= 2.5e-43 complete sequence hit coverage= 100%, length is 306aa with 110 gaps, 1 stops, frame= Minus2 at 729681..730597 in the same strand
misc_feature no id HMMPfam hit to PF02371, Transposase IS116/IS110/IS902 family, score 8.3e-25 intersects 100%(108aa) with IS110 e-value= 2.5e-43 complete sequence hit coverage= 100%, length is 306aa with 110 gaps, 1 stops, frame= Minus2 at 729681..730597 in the same strand

misc_feature no id HMMPfam hit to PF01548, Transposase, score 1.8e-06 intersects 100%(98aa) with IS110 e-value= 2.5e-43 complete sequence hit coverage= 100%, length is 306aa with 110 gaps, 1 stops, frame= Minus2 at 729681..730597 in the same strand
misc_feature no id Transposase; Region: Transposase_9; pfam01548 intersects 100%(79aa) with IS110 e-value= 2.5e-43 complete sequence hit coverage= 100%, length is 306aa with 110 gaps, 1 stops, frame= Minus2 at 729681..730597 in the same strand
repeat_region no id no product intersects 65.10%(294aa) with IS110 e-value= 5.3e-46 fragment hit coverage= 96.88%, length is 295aa with 108 gaps, 0 stops, frame= Minus2 at 729714..730597 in the opposite strand
CDS YP_001970593.1 putative transposase intersects 96.61%(294aa) with IS110 e-value= 5.3e-46 fragment hit coverage= 96.88%, length is 295aa with 108 gaps, 0 stops, frame= Minus2 at 729714..730597 in the same strand
misc_feature no id Transposase and inactivated derivatives [DNA replication, recombination, and repair]; Region: COG3547 intersects 100%(292aa) with IS110 e-value= 5.3e-46 fragment hit coverage= 96.88%, length is 295aa with 108 gaps, 0 stops, frame= Minus2 at 729714..730597 in the same strand
misc_feature no id Transposase IS116/IS110/IS902 family; Region: Transposase_20; pfam02371 intersects 100%(84aa) with IS110 e-value= 5.3e-46 fragment hit coverage= 96.88%, length is 295aa with 108 gaps, 0 stops, frame= Minus2 at 729714..730597 in the same strand
misc_feature no id HMMPfam hit to PF02371, Transposase IS116/IS110/IS902 family, score 8.3e-25 intersects 100%(108aa) with IS110 e-value= 5.3e-46 fragment hit coverage= 96.88%, length is 295aa with 108 gaps, 0 stops, frame= Minus2 at 729714..730597 in the same strand
misc_feature no id HMMPfam hit to PF01548, Transposase, score 1.8e-06 intersects 100%(98aa) with IS110 e-value= 5.3e-46 fragment hit coverage= 96.88%, length is 295aa with 108 gaps, 0 stops, frame= Minus2 at 729714..730597 in the same strand
misc_feature no id Transposase; Region: Transposase_9; pfam01548 intersects 100%(79aa) with IS110 e-value= 5.3e-46 fragment hit coverage= 96.88%, length is 295aa with 108 gaps, 0 stops, frame= Minus2 at 729714..730597 in the same strand
Class A

11.
CDS YP_001970697.1 putative ISPsy9 like transposase intersects 42.15%(73aa) with IS3_IS150_ORF2 e-value= 5e-121 complete sequence hit coverage= 100%, length is 350aa with 42 gaps, 3 stops, frame= Plus1 at 850483..851532 in the same strand
CDS YP_001970698.1 putative ISPsy9 like transposase integrase intersects 100%(278aa) with IS3_IS150_ORF2 e-value= 5e-121 complete sequence hit coverage= 100%, length is 350aa with 42 gaps, 3 stops, frame= Plus1 at 850483..851532 in the same strand
misc_feature no id putative transposase OrfB; Reviewed; Region: PHA02517 intersects 100%(251aa) with IS3_IS150_ORF2 e-value= 5e-121 complete sequence hit coverage= 100%, length is 350aa with 42 gaps, 3 stops, frame= Plus1 at 850483..851532 in the same strand
misc_feature no id Integrase core domain; Region: rve; cl01316 intersects 100%(118aa) with IS3_IS150_ORF2 e-value= 5e-121 complete sequence hit coverage= 100%, length is 350aa with 42 gaps, 3 stops, frame= Plus1 at 850483..851532 in the same strand
misc_feature no id HMMPfam hit to PF00665, Integrase core domain, score 1.9e-41 intersects 100%(160aa) with IS3_IS150_ORF2 e-value= 5e-121 complete sequence hit coverage= 100%, length is 350aa with 42 gaps, 3 stops, frame= Plus1 at 850483..851532 in the same strand
CDS YP_001970697.1 putative ISPsy9 like transposase intersects 15.13%(26aa) with IS3_IS150_ORF1 e-value= 3.2e-87 complete sequence hit coverage= 100%, length is 300aa with 9 gaps, 0 stops, frame= Plus1 at 850624..851523 in the same strand
CDS YP_001970698.1 putative ISPsy9 like transposase integrase intersects 98.92%(275aa) with IS3_IS150_ORF1 e-value= 3.2e-87 complete sequence hit coverage= 100%, length is 300aa with 9 gaps, 0 stops, frame= Plus1 at 850624..851523 in the same strand
misc_feature no id putative transposase OrfB; Reviewed; Region: PHA02517 intersects 100%(251aa) with IS3_IS150_ORF1 e-value= 3.2e-87 complete sequence hit coverage= 100%, length is 300aa with 9 gaps, 0 stops, frame= Plus1 at 850624..851523 in the same strand
misc_feature no id Integrase core domain; Region: rve; cl01316 intersects 100%(118aa) with IS3_IS150_ORF1 e-value= 3.2e-87 complete sequence hit coverage= 100%, length is 300aa with 9 gaps, 0 stops, frame= Plus1 at 850624..851523 in the same strand

misc_feature no id HMMPfam hit to PF00665, Integrase core domain, score 1.9e-41 intersects 100%(160aa) with IS3_IS150_ORF1 e-value= 3.2e-87 complete sequence hit coverage= 100%, length is 300aa with 9 gaps, 0 stops, frame= Plus1 at 850624..851523 in the same strand
CDS YP_001970697.1 putative ISPsy9 like transposase intersects 5.94%(10aa) with IS3_IS150_ORF2 e-value= 1.9e-151 fragment hit coverage= 76.61%, length is 287aa with 14 gaps, 1 stops, frame= Plus1 at 850672..851532 in the same strand
CDS YP_001970698.1 putative ISPsy9 like transposase integrase intersects 100%(278aa) with IS3_IS150_ORF2 e-value= 1.9e-151 fragment hit coverage= 76.61%, length is 287aa with 14 gaps, 1 stops, frame= Plus1 at 850672..851532 in the same strand
misc_feature no id putative transposase OrfB; Reviewed; Region: PHA02517 intersects 100%(251aa) with IS3_IS150_ORF2 e-value= 1.9e-151 fragment hit coverage= 76.61%, length is 287aa with 14 gaps, 1 stops, frame= Plus1 at 850672..851532 in the same strand
misc_feature no id Integrase core domain; Region: rve; cl01316 intersects 100%(118aa) with IS3_IS150_ORF2 e-value= 1.9e-151 fragment hit coverage= 76.61%, length is 287aa with 14 gaps, 1 stops, frame= Plus1 at 850672..851532 in the same strand
misc_feature no id HMMPfam hit to PF00665, Integrase core domain, score 1.9e-41 intersects 100%(160aa) with IS3_IS150_ORF2 e-value= 1.9e-151 fragment hit coverage= 76.61%, length is 287aa with 14 gaps, 1 stops, frame= Plus1 at 850672..851532 in the same strand
CDS YP_001970697.1 putative ISPsy9 like transposase intersects 4.21%(7aa) with IS3_IS51_ORF2e-value= 1.2e-17 complete sequence hit coverage= 100%, length is 292aa with 25 gaps, 1 stops, frame= Plus1 at 850681..851556 in the same strand
CDS YP_001970698.1 putative ISPsy9 like transposase integrase intersects 100%(278aa) with IS3_IS51_ORF2 e-value= 1.2e-17 complete sequence hit coverage= 100%, length is 292aa with 25 gaps, 1 stops, frame= Plus1 at 850681..851556 in the same strand
misc_feature no id putative transposase OrfB; Reviewed; Region: PHA02517 intersects 100%(251aa) with IS3_IS51_ORF2 e-value= 1.2e-17 complete sequence hit coverage= 100%, length is 292aa with 25 gaps, 1 stops, frame= Plus1 at 850681..851556 in the same strand
misc_feature no id Integrase core domain; Region: rve; cl01316 intersects 100%(118aa) with IS3_IS51_ORF2 e-value= 1.2e-17 complete sequence hit coverage= 100%, length is 292aa with 25 gaps, 1 stops, frame= Plus1 at 850681..851556 in the same strand
misc_feature no id HMMPfam hit to PF00665, Integrase core domain, score 1.9e-41 intersects 100%(160aa) with IS3_IS51_ORF2 e-value= 1.2e-17 complete sequence hit coverage= 100%, length is 292aa with 25 gaps, 1 stops, frame= Plus1 at 850681..851556 in the same strand
CDS YP_001970697.1 putative ISPsy9 like transposase intersects 4.21%(7aa) with IS3_IS51_ORF1e-value= 8.5e-39 complete sequence hit coverage= 100%, length is 291aa with 18 gaps, 1 stops, frame= Plus1 at 850681..851553 in the same strand
CDS YP_001970698.1 putative ISPsy9 like transposase integrase intersects 100%(278aa) with IS3_IS51_ORF1 e-value= 8.5e-39 complete sequence hit coverage= 100%, length is 291aa with 18 gaps, 1 stops, frame= Plus1 at 850681..851553 in the same strand
misc_feature no id putative transposase OrfB; Reviewed; Region: PHA02517 intersects 100%(251aa) with IS3_IS51_ORF1 e-value= 8.5e-39 complete sequence hit coverage= 100%, length is 291aa with 18 gaps, 1 stops, frame= Plus1 at 850681..851553 in the same strand
misc_feature no id Integrase core domain; Region: rve; cl01316 intersects 100%(118aa) with IS3_IS51_ORF1 e-value= 8.5e-39 complete sequence hit coverage= 100%, length is 291aa with 18 gaps, 1 stops, frame= Plus1 at 850681..851553 in the same strand
misc_feature no id HMMPfam hit to PF00665, Integrase core domain, score 1.9e-41 intersects 100%(160aa) with IS3_IS51_ORF1 e-value= 8.5e-39 complete sequence hit coverage= 100%, length is 291aa with 18 gaps, 1 stops, frame= Plus1 at 850681..851553 in the same strand
CDS YP_001970697.1 putative ISPsy9 like transposase intersects 3.64%(6aa) with IS3_IS3_ORF1 e-value= 4.6e-75 complete sequence hit coverage= 100%, length is 279aa with 8 gaps, 0 stops, frame= Plus1 at 850684..851520 in the same strand
CDS YP_001970698.1 putative ISPsy9 like transposase integrase intersects 98.56%(274aa) with IS3_IS3_ORF1 e-value= 4.6e-75 complete sequence hit coverage= 100%, length is 279aa with 8 gaps, 0 stops, frame= Plus1 at 850684..851520 in the same strand
misc_feature no id putative transposase OrfB; Reviewed; Region: PHA02517 intersects 100%(251aa) with IS3_IS3_ORF1 e-value= 4.6e-75 complete sequence hit coverage= 100%, length is 279aa with 8 gaps, 0 stops, frame= Plus1 at 850684..851520 in the same strand

misc_feature no id Integrase core domain; Region: rve; cl01316 intersects 100%(118aa) with IS3_IS3_ORF1 e-value= 4.6e-75 complete sequence hit coverage= 100%, length is 279aa with 8 gaps, 0 stops, frame= Plus1 at 850684..851520 in the same strand
misc_feature no id HMMPfam hit to PF00665, Integrase core domain, score 1.9e-41 intersects 100%(160aa) with IS3_IS3_ORF1 e-value= 4.6e-75 complete sequence hit coverage= 100%, length is 279aa with 8 gaps, 0 stops, frame= Plus1 at 850684..851520 in the same strand
CDS YP_001970697.1 putative ISPsy9 like transposase intersects 1.34%(2aa) with IS3_IS3_ORF2 e-value= 3.3e-64 complete sequence hit coverage= 100%, length is 279aa with 9 gaps, 1 stops, frame= Plus1 at 850696..851532 in the same strand
CDS YP_001970698.1 putative ISPsy9 like transposase integrase intersects 100%(278aa) with IS3_IS3_ORF2 e-value= 3.3e-64 complete sequence hit coverage= 100%, length is 279aa with 9 gaps, 1 stops, frame= Plus1 at 850696..851532 in the same strand
misc_feature no id putative transposase OrfB; Reviewed; Region: PHA02517 intersects 100%(251aa) with IS3_IS3_ORF2 e-value= 3.3e-64 complete sequence hit coverage= 100%, length is 279aa with 9 gaps, 1 stops, frame= Plus1 at 850696..851532 in the same strand
misc_feature no id Integrase core domain; Region: rve; cl01316 intersects 100%(118aa) with IS3_IS3_ORF2 e-value= 3.3e-64 complete sequence hit coverage= 100%, length is 279aa with 9 gaps, 1 stops, frame= Plus1 at 850696..851532 in the same strand
misc_feature no id HMMPfam hit to PF00665, Integrase core domain, score 1.9e-41 intersects 100%(160aa) with IS3_IS3_ORF2 e-value= 3.3e-64 complete sequence hit coverage= 100%, length is 279aa with 9 gaps, 1 stops, frame= Plus1 at 850696..851532 in the same strand
CDS YP_001970698.1 putative ISPsy9 like transposase integrase intersects 84.17%(234aa) with IS3_IS51_ORF1 e-value= 5e-45 fragment hit coverage= 79.80%, length is 234aa with 12 gaps, 0 stops, frame= Plus1 at 850804..851505 in the same strand
misc_feature no id putative transposase OrfB; Reviewed; Region: PHA02517 intersects 92.83%(233aa) with IS3_IS51_ORF1 e-value= 5e-45 fragment hit coverage= 79.80%, length is 234aa with 12 gaps, 0 stops, frame= Plus1 at 850804..851505 in the same strand
misc_feature no id Integrase core domain; Region: rve; cl01316 intersects 100%(118aa) with IS3_IS51_ORF1 e-value= 5e-45 fragment hit coverage= 79.80%, length is 234aa with 12 gaps, 0 stops, frame= Plus1 at 850804..851505 in the same strand
misc_feature no id HMMPfam hit to PF00665, Integrase core domain, score 1.9e-41 intersects 98.75%(158aa) with IS3_IS51_ORF1 e-value= 5e-45 fragment hit coverage= 79.80%, length is 234aa with 12 gaps, 0 stops, frame= Plus1 at 850804..851505 in the same strand
CDS YP_001970698.1 putative ISPsy9 like transposase integrase intersects 63.31%(176aa) with IS3_IS51_ORF2 e-value= 7.8e-32 fragment hit coverage= 57.41%, length is 176aa with 6 gaps, 0 stops, frame= Plus1 at 850855..851382 in the same strand
misc_feature no id putative transposase OrfB; Reviewed; Region: PHA02517 intersects 70.12%(176aa) with IS3_IS51_ORF2 e-value= 7.8e-32 fragment hit coverage= 57.41%, length is 176aa with 6 gaps, 0 stops, frame= Plus1 at 850855..851382 in the same strand
misc_feature no id Integrase core domain; Region: rve; cl01316 intersects 99.15%(117aa) with IS3_IS51_ORF2 e-value= 7.8e-32 fragment hit coverage= 57.41%, length is 176aa with 6 gaps, 0 stops, frame= Plus1 at 850855..851382 in the same strand
misc_feature no id HMMPfam hit to PF00665, Integrase core domain, score 1.9e-41 intersects 73.12%(117aa) with IS3_IS51_ORF2 e-value= 7.8e-32 fragment hit coverage= 57.41%, length is 176aa with 6 gaps, 0 stops, frame= Plus1 at 850855..851382 in the same strand
CDS YP_001970698.1 putative ISPsy9 like transposase integrase intersects 12.95%(36aa) with IS481 e-value= 3.8e-07 fragment hit coverage= 11.11%, length is 36aa with 3 gaps, 0 stops, frame= Plus1 at 851359..851466 in the same strand
misc_feature no id putative transposase OrfB; Reviewed; Region: PHA02517 intersects 14.34%(36aa) with IS481 e-value= 3.8e-07 fragment hit coverage= 11.11%, length is 36aa with 3 gaps, 0 stops, frame= Plus1 at 851359..851466 in the same strand
misc_feature no id Integrase core domain; Region: rve; cl01316 intersects 7.63%(9aa) with IS481 e-value= 3.8e-07 fragment hit coverage= 11.11%, length is 36aa with 3 gaps, 0 stops, frame= Plus1 at 851359..851466 in the same strand
misc_feature no id HMMPfam hit to PF00665, Integrase core domain, score 1.9e-41 intersects 22.50%(36aa) with IS481 e-value= 3.8e-07 fragment hit coverage= 11.11%, length is 36aa with 3 gaps, 0 stops, frame= Plus1 at 851359..851466 in the same strand

Class A

12.
CDS YP_001970725.1 putative ISPsy9 like transposase intersects 100%(278aa) with IS3_IS51_ORF2 e-value= 1.1e-16 complete sequence hit coverage= 100%, length is 292aa with 25 gaps, 1 stops, frame= Minus1 at 876160..877034 in the same strand
misc_feature no id HMMPfam hit to PF00665, Integrase core domain, score 7.3e-43 intersects 100%(160aa) with IS3_IS51_ORF2 e-value= 1.1e-16 complete sequence hit coverage= 100%, length is 292aa with 25 gaps, 1 stops, frame= Minus1 at 876160..877034 in the same strand
misc_feature no id putative transposase OrfB; Reviewed; Region: PHA02517 intersects 100%(251aa) with IS3_IS51_ORF2 e-value= 1.1e-16 complete sequence hit coverage= 100%, length is 292aa with 25 gaps, 1 stops, frame= Minus1 at 876160..877034 in the same strand
misc_feature no id Integrase core domain; Region: rve; cl01316 intersects 100%(118aa) with IS3_IS51_ORF2 e-value= 1.1e-16 complete sequence hit coverage= 100%, length is 292aa with 25 gaps, 1 stops, frame= Minus1 at 876160..877034 in the same strand
CDS YP_001970726.1 putative ISPsy9 like transposase intersects 4.02%(7aa) with IS3_IS51_ORF2e-value= 1.1e-16 complete sequence hit coverage= 100%, length is 292aa with 25 gaps, 1 stops, frame= Minus1 at 876160..877034 in the same strand
CDS YP_001970725.1 putative ISPsy9 like transposase intersects 100%(278aa) with IS3_IS51_ORF1 e-value= 3.8e-39 complete sequence hit coverage= 100%, length is 291aa with 18 gaps, 1 stops, frame= Minus1 at 876163..877034 in the same strand
misc_feature no id HMMPfam hit to PF00665, Integrase core domain, score 7.3e-43 intersects 100%(160aa) with IS3_IS51_ORF1 e-value= 3.8e-39 complete sequence hit coverage= 100%, length is 291aa with 18 gaps, 1 stops, frame= Minus1 at 876163..877034 in the same strand
misc_feature no id putative transposase OrfB; Reviewed; Region: PHA02517 intersects 100%(251aa) with IS3_IS51_ORF1 e-value= 3.8e-39 complete sequence hit coverage= 100%, length is 291aa with 18 gaps, 1 stops, frame= Minus1 at 876163..877034 in the same strand
misc_feature no id Integrase core domain; Region: rve; cl01316 intersects 100%(118aa) with IS3_IS51_ORF1 e-value= 3.8e-39 complete sequence hit coverage= 100%, length is 291aa with 18 gaps, 1 stops, frame= Minus1 at 876163..877034 in the same strand
CDS YP_001970726.1 putative ISPsy9 like transposase intersects 4.02%(7aa) with IS3_IS51_ORF1e-value= 3.8e-39 complete sequence hit coverage= 100%, length is 291aa with 18 gaps, 1 stops, frame= Minus1 at 876163..877034 in the same strand
CDS YP_001970725.1 putative ISPsy9 like transposase intersects 100%(278aa) with IS3_IS407_ORF2 e-value= 1.2e-08 complete sequence hit coverage= 100%, length is 362aa with 12 gaps, 3 stops, frame= Minus1 at 876166..877250 in the same strand
misc_feature no id HMMPfam hit to PF00665, Integrase core domain, score 7.3e-43 intersects 100%(160aa) with IS3_IS407_ORF2 e-value= 1.2e-08 complete sequence hit coverage= 100%, length is 362aa with 12 gaps, 3 stops, frame= Minus1 at 876166..877250 in the same strand
misc_feature no id putative transposase OrfB; Reviewed; Region: PHA02517 intersects 100%(251aa) with IS3_IS407_ORF2 e-value= 1.2e-08 complete sequence hit coverage= 100%, length is 362aa with 12 gaps, 3 stops, frame= Minus1 at 876166..877250 in the same strand
misc_feature no id Integrase core domain; Region: rve; cl01316 intersects 100%(118aa) with IS3_IS407_ORF2 e-value= 1.2e-08 complete sequence hit coverage= 100%, length is 362aa with 12 gaps, 3 stops, frame= Minus1 at 876166..877250 in the same strand
CDS YP_001970726.1 putative ISPsy9 like transposase intersects 45.40%(79aa) with IS3_IS407_ORF2 e-value= 1.2e-08 complete sequence hit coverage= 100%, length is 362aa with 12 gaps, 3 stops, frame= Minus1 at 876166..877250 in the same strand
misc_feature no id Transposase and inactivated derivatives [DNA replication, recombination, and repair]; Region: COG2963 intersects 5%(5aa) with IS3_IS407_ORF2 e-value= 1.2e-08 complete sequence hit coverage= 100%, length is 362aa with 12 gaps, 3 stops, frame= Minus1 at 876166..877250 in the same strand
CDS YP_001970725.1 putative ISPsy9 like transposase intersects 100%(278aa) with IS3_IS3_ORF2 e-value= 1.5e-64 complete sequence hit coverage= 100%, length is 279aa with 9 gaps, 1 stops, frame= Minus1 at 876184..877019 in the same strand
misc_feature no id HMMPfam hit to PF00665, Integrase core domain, score 7.3e-43 intersects 100%(160aa) with IS3_IS3_ORF2 e-value= 1.5e-64 complete sequence hit coverage= 100%, length is 279aa with 9 gaps, 1 stops, frame= Minus1 at 876184..877019 in the same strand

misc_feature	no id	putative transposase OrfB; Reviewed; Region: PHA02517 intersects 100%(251aa) with IS3_IS3_ORF2	e-value= 1.5e-64 complete sequence hit coverage= 100%, length is 279aa with 9 gaps, 1 stops, frame= Minus1 at 876184..877019 in the same strand
misc_feature	no id	Integrase core domain; Region: rve; cl01316 intersects 100%(118aa) with IS3_IS3_ORF2	e-value= 1.5e-64 complete sequence hit coverage= 100%, length is 279aa with 9 gaps, 1 stops, frame= Minus1 at 876184..877019 in the same strand
CDS	YP_001970726.1	putative ISPsy9 like transposase intersects 1.15%(2aa) with IS3_IS3_ORF2	e-value= 1.5e-64 complete sequence hit coverage= 100%, length is 279aa with 9 gaps, 1 stops, frame= Minus1 at 876184..877019 in the same strand
CDS	YP_001970725.1	putative ISPsy9 like transposase intersects 100%(278aa) with IS3_IS150_ORF2	e-value= 1.9e-152 fragment hit coverage= 76.61%, length is 287aa with 14 gaps, 1 stops, frame= Minus1 at 876184..877043 in the same strand
misc_feature	no id	HMMPfam hit to PF00665, Integrase core domain, score 7.3e-43 intersects 100%(160aa) with IS3_IS150_ORF2	e-value= 1.9e-152 fragment hit coverage= 76.61%, length is 287aa with 14 gaps, 1 stops, frame= Minus1 at 876184..877043 in the same strand
misc_feature	no id	putative transposase OrfB; Reviewed; Region: PHA02517 intersects 100%(251aa) with IS3_IS150_ORF2	e-value= 1.9e-152 fragment hit coverage= 76.61%, length is 287aa with 14 gaps, 1 stops, frame= Minus1 at 876184..877043 in the same strand
misc_feature	no id	Integrase core domain; Region: rve; cl01316 intersects 100%(118aa) with IS3_IS150_ORF2	e-value= 1.9e-152 fragment hit coverage= 76.61%, length is 287aa with 14 gaps, 1 stops, frame= Minus1 at 876184..877043 in the same strand
CDS	YP_001970726.1	putative ISPsy9 like transposase intersects 5.75%(10aa) with IS3_IS150_ORF2	e-value= 1.9e-152 fragment hit coverage= 76.61%, length is 287aa with 14 gaps, 1 stops, frame= Minus1 at 876184..877043 in the same strand
CDS	YP_001970725.1	putative ISPsy9 like transposase intersects 100%(278aa) with IS3_IS150_ORF2	e-value= 1.4e-122 complete sequence hit coverage= 100%, length is 354aa with 38 gaps, 3 stops, frame= Minus1 at 876184..877244 in the same strand
misc_feature	no id	HMMPfam hit to PF00665, Integrase core domain, score 7.3e-43 intersects 100%(160aa) with IS3_IS150_ORF2	e-value= 1.4e-122 complete sequence hit coverage= 100%, length is 354aa with 38 gaps, 3 stops, frame= Minus1 at 876184..877244 in the same strand
misc_feature	no id	putative transposase OrfB; Reviewed; Region: PHA02517 intersects 100%(251aa) with IS3_IS150_ORF2	e-value= 1.4e-122 complete sequence hit coverage= 100%, length is 354aa with 38 gaps, 3 stops, frame= Minus1 at 876184..877244 in the same strand
misc_feature	no id	Integrase core domain; Region: rve; cl01316 intersects 100%(118aa) with IS3_IS150_ORF2	e-value= 1.4e-122 complete sequence hit coverage= 100%, length is 354aa with 38 gaps, 3 stops, frame= Minus1 at 876184..877244 in the same strand
CDS	YP_001970726.1	putative ISPsy9 like transposase intersects 44.25%(77aa) with IS3_IS150_ORF2	e-value= 1.4e-122 complete sequence hit coverage= 100%, length is 354aa with 38 gaps, 3 stops, frame= Minus1 at 876184..877244 in the same strand
misc_feature	no id	Transposase and inactivated derivatives [DNA replication, recombination, and repair]; Region: COG2963 intersects 3%(3aa) with IS3_IS150_ORF2	e-value= 1.4e-122 complete sequence hit coverage= 100%, length is 354aa with 38 gaps, 3 stops, frame= Minus1 at 876184..877244 in the same strand
CDS	YP_001970725.1	putative ISPsy9 like transposase intersects 98.92%(275aa) with IS3_IS150_ORF1	e-value= 8e-89 complete sequence hit coverage= 100%, length is 300aa with 9 gaps, 0 stops, frame= Minus1 at 876193..877091 in the same strand
misc_feature	no id	HMMPfam hit to PF00665, Integrase core domain, score 7.3e-43 intersects 100%(160aa) with IS3_IS150_ORF1	e-value= 8e-89 complete sequence hit coverage= 100%, length is 300aa with 9 gaps, 0 stops, frame= Minus1 at 876193..877091 in the same strand
misc_feature	no id	putative transposase OrfB; Reviewed; Region: PHA02517 intersects 100%(251aa) with IS3_IS150_ORF1	e-value= 8e-89 complete sequence hit coverage= 100%, length is 300aa with 9 gaps, 0 stops, frame= Minus1 at 876193..877091 in the same strand
misc_feature	no id	Integrase core domain; Region: rve; cl01316 intersects 100%(118aa) with IS3_IS150_ORF1	e-value= 8e-89 complete sequence hit coverage= 100%, length is 300aa with 9 gaps, 0 stops, frame= Minus1 at 876193..877091 in the same strand
CDS	YP_001970726.1	putative ISPsy9 like transposase intersects 14.94%(26aa) with IS3_IS150_ORF1	e-value= 8e-89 complete sequence hit coverage= 100%, length is 300aa with 9 gaps, 0 stops, frame= Minus1 at 876193..877091 in the same strand

CDS YP_001970725.1 putative ISPsy9 like transposase intersects 98.56%(274aa) with IS3_IS3_ORF1 e-value= 2.5e-76 complete sequence hit coverage= 100%, length is 279aa with 8 gaps, 0 stops, frame= Minus1 at 876196..877031 in the same strand
misc_feature no id HMMPfam hit to PF00665, Integrase core domain, score 7.3e-43 intersects 100%(160aa) with IS3_IS3_ORF1 e-value= 2.5e-76 complete sequence hit coverage= 100%, length is 279aa with 8 gaps, 0 stops, frame= Minus1 at 876196..877031 in the same strand
misc_feature no id putative transposase OrfB; Reviewed; Region: PHA02517 intersects 100%(251aa) with IS3_IS3_ORF1 e-value= 2.5e-76 complete sequence hit coverage= 100%, length is 279aa with 8 gaps, 0 stops, frame= Minus1 at 876196..877031 in the same strand
misc_feature no id Integrase core domain; Region: rve; cl01316 intersects 100%(118aa) with IS3_IS3_ORF1 e-value= 2.5e-76 complete sequence hit coverage= 100%, length is 279aa with 8 gaps, 0 stops, frame= Minus1 at 876196..877031 in the same strand
CDS YP_001970726.1 putative ISPsy9 like transposase intersects 3.45%(6aa) with IS3_IS3_ORF1 e-value= 2.5e-76 complete sequence hit coverage= 100%, length is 279aa with 8 gaps, 0 stops, frame= Minus1 at 876196..877031 in the same strand
CDS YP_001970725.1 putative ISPsy9 like transposase intersects 84.05%(233aa) with IS3_IS51_ORF1 e-value= 2.3e-45 fragment hit coverage= 79.80%, length is 234aa with 12 gaps, 0 stops, frame= Minus1 at 876211..876911 in the same strand
misc_feature no id HMMPfam hit to PF00665, Integrase core domain, score 7.3e-43 intersects 98.75%(158aa) with IS3_IS51_ORF1 e-value= 2.3e-45 fragment hit coverage= 79.80%, length is 234aa with 12 gaps, 0 stops, frame= Minus1 at 876211..876911 in the same strand
misc_feature no id putative transposase OrfB; Reviewed; Region: PHA02517 intersects 92.70%(232aa) with IS3_IS51_ORF1 e-value= 2.3e-45 fragment hit coverage= 79.80%, length is 234aa with 12 gaps, 0 stops, frame= Minus1 at 876211..876911 in the same strand
misc_feature no id Integrase core domain; Region: rve; cl01316 intersects 100%(118aa) with IS3_IS51_ORF1 e-value= 2.3e-45 fragment hit coverage= 79.80%, length is 234aa with 12 gaps, 0 stops, frame= Minus1 at 876211..876911 in the same strand
CDS YP_001970725.1 putative ISPsy9 like transposase intersects 12.83%(35aa) with IS481 e-value= 1.2e-06 fragment hit coverage= 11.11%, length is 36aa with 3 gaps, 0 stops, frame= Minus1 at 876250..876356 in the same strand
misc_feature no id HMMPfam hit to PF00665, Integrase core domain, score 7.3e-43 intersects 22.29%(35aa) with IS481 e-value= 1.2e-06 fragment hit coverage= 11.11%, length is 36aa with 3 gaps, 0 stops, frame= Minus1 at 876250..876356 in the same strand
misc_feature no id putative transposase OrfB; Reviewed; Region: PHA02517 intersects 14.21%(35aa) with IS481 e-value= 1.2e-06 fragment hit coverage= 11.11%, length is 36aa with 3 gaps, 0 stops, frame= Minus1 at 876250..876356 in the same strand
misc_feature no id Integrase core domain; Region: rve; cl01316 intersects 7.34%(8aa) with IS481 e-value= 1.2e-06 fragment hit coverage= 11.11%, length is 36aa with 3 gaps, 0 stops, frame= Minus1 at 876250..876356 in the same strand
CDS YP_001970725.1 putative ISPsy9 like transposase intersects 63.19%(175aa) with IS3_IS51_ORF2 e-value= 5.2e-32 fragment hit coverage= 57.41%, length is 176aa with 6 gaps, 0 stops, frame= Minus1 at 876334..876860 in the same strand
misc_feature no id HMMPfam hit to PF00665, Integrase core domain, score 7.3e-43 intersects 73.12%(117aa) with IS3_IS51_ORF2 e-value= 5.2e-32 fragment hit coverage= 57.41%, length is 176aa with 6 gaps, 0 stops, frame= Minus1 at 876334..876860 in the same strand
misc_feature no id putative transposase OrfB; Reviewed; Region: PHA02517 intersects 69.99%(175aa) with IS3_IS51_ORF2 e-value= 5.2e-32 fragment hit coverage= 57.41%, length is 176aa with 6 gaps, 0 stops, frame= Minus1 at 876334..876860 in the same strand
misc_feature no id Integrase core domain; Region: rve; cl01316 intersects 99.15%(117aa) with IS3_IS51_ORF2 e-value= 5.2e-32 fragment hit coverage= 57.41%, length is 176aa with 6 gaps, 0 stops, frame= Minus1 at 876334..876860 in the same strand
Class A

13.
repeat_region no id no product intersects 68.23%(305aa) with IS110 e-value= 3.4e-46 complete sequence hit coverage= 100%, length is 306aa with 110 gaps, 1 stops, frame= Minus2 at 1142961..1143877 in the opposite strand

CDS YP_001970965.1 putative transposase intersects 99.56%(303aa) with IS110 e-value= 3.4e-46 complete sequence hit coverage= 100%, length is 306aa with 110 gaps, 1 stops, frame= Minus2 at 1142961..1143877 in the same strand
misc_feature no id Transposase and inactivated derivatives [DNA replication, recombination, and repair]; Region: COG3547 intersects 100%(292aa) with IS110 e-value= 3.4e-46 complete sequence hit coverage= 100%, length is 306aa with 110 gaps, 1 stops, frame= Minus2 at 1142961..1143877 in the same strand
misc_feature no id Transposase IS116/IS110/IS902 family; Region: Transposase_20; pfam02371 intersects 100%(84aa) with IS110 e-value= 3.4e-46 complete sequence hit coverage= 100%, length is 306aa with 110 gaps, 1 stops, frame= Minus2 at 1142961..1143877 in the same strand
misc_feature no id HMMPfam hit to PF02371, Transposase IS116/IS110/IS902 family, score 4.3e-27 intersects 100%(108aa) with IS110 e-value= 3.4e-46 complete sequence hit coverage= 100%, length is 306aa with 110 gaps, 1 stops, frame= Minus2 at 1142961..1143877 in the same strand
misc_feature no id HMMPfam hit to PF01548, Transposase, score 1.8e-06 intersects 100%(98aa) with IS110 e-value= 3.4e-46 complete sequence hit coverage= 100%, length is 306aa with 110 gaps, 1 stops, frame= Minus2 at 1142961..1143877 in the same strand
misc_feature no id Transposase; Region: Transposase_9; pfam01548 intersects 100%(79aa) with IS110 e-value= 3.4e-46 complete sequence hit coverage= 100%, length is 306aa with 110 gaps, 1 stops, frame= Minus2 at 1142961..1143877 in the same strand
repeat_region no id no product intersects 65.77%(294aa) with IS110 e-value= 1e-48 fragment hit coverage= 96.88%, length is 295aa with 108 gaps, 0 stops, frame= Minus2 at 1142994..1143877 in the opposite strand
CDS YP_001970965.1 putative transposase intersects 96.61%(294aa) with IS110 e-value= 1e-48 fragment hit coverage= 96.88%, length is 295aa with 108 gaps, 0 stops, frame= Minus2 at 1142994..1143877 in the same strand
misc_feature no id Transposase and inactivated derivatives [DNA replication, recombination, and repair]; Region: COG3547 intersects 100%(292aa) with IS110 e-value= 1e-48 fragment hit coverage= 96.88%, length is 295aa with 108 gaps, 0 stops, frame= Minus2 at 1142994..1143877 in the same strand
misc_feature no id Transposase IS116/IS110/IS902 family; Region: Transposase_20; pfam02371 intersects 100%(84aa) with IS110 e-value= 1e-48 fragment hit coverage= 96.88%, length is 295aa with 108 gaps, 0 stops, frame= Minus2 at 1142994..1143877 in the same strand
misc_feature no id HMMPfam hit to PF02371, Transposase IS116/IS110/IS902 family, score 4.3e-27 intersects 100%(108aa) with IS110 e-value= 1e-48 fragment hit coverage= 96.88%, length is 295aa with 108 gaps, 0 stops, frame= Minus2 at 1142994..1143877 in the same strand
misc_feature no id HMMPfam hit to PF01548, Transposase, score 1.8e-06 intersects 100%(98aa) with IS110 e-value= 1e-48 fragment hit coverage= 96.88%, length is 295aa with 108 gaps, 0 stops, frame= Minus2 at 1142994..1143877 in the same strand
misc_feature no id Transposase; Region: Transposase_9; pfam01548 intersects 100%(79aa) with IS110 e-value= 1e-48 fragment hit coverage= 96.88%, length is 295aa with 108 gaps, 0 stops, frame= Minus2 at 1142994..1143877 in the same strand
Class A

14.
CDS YP_001970973.1 hypothetical protein intersects 21.95%(36aa) with IS200_IS605 e-value= 5.7e-06 fragment hit coverage= 24.18%, length is 36aa with 1 gaps, 0 stops, frame= Plus1 at 1150204..1150311 in the same strand
misc_feature no id Transposase IS200 like; Region: Transposase_17; cl00848 intersects 32.73%(36aa) with IS200_IS605 e-value= 5.7e-06 fragment hit coverage= 24.18%, length is 36aa with 1 gaps, 0 stops, frame= Plus1 at 1150204..1150311 in the same strand
misc_feature no id HMMPfam hit to PF07605, Protein of unknown function (DUF1568), score 9.6e-11 intersects 70%(35aa) with IS200_IS605 e-value= 5.7e-06 fragment hit coverage= 24.18%, length is 36aa with 1 gaps, 0 stops, frame= Plus1 at 1150204..1150311 in the same strand
Class A

15.
CDS YP_001971182.1 putative transposase intersects 99.71%(347aa) with IS630 e-value= 2.1e-108 complete sequence hit coverage= 100%, length is 359aa with 18 gaps, 1 stops, frame= Plus3 at 1355496..1356572 in the same strand

misc_feature no id Transposase and inactivated derivatives [DNA replication, recombination, and repair]; Region: COG3415 intersects 100%(153aa) with IS630 e-value= 2.1e-108 complete sequence hit coverage= 100%, length is 359aa with 18 gaps, 1 stops, frame= Plus3 at 1355496..1356572 in the same strand
misc_feature no id Integrase core domain; Region: rve; cl01316 intersects 100%(109aa) with IS630 e-value= 2.1e-108 complete sequence hit coverage= 100%, length is 359aa with 18 gaps, 1 stops, frame= Plus3 at 1355496..1356572 in the same strand
CDS YP_001971183.1 hypothetical protein intersects 0.19%(0aa) with IS630 e-value= 2.1e-108 complete sequence hit coverage= 100%, length is 359aa with 18 gaps, 1 stops, frame= Plus3 at 1355496..1356572 in the same strand
CDS YP_001971182.1 putative transposase intersects 99.71%(347aa) with IS630 e-value= 7.9e-108 fragment hit coverage= 96.81%, length is 347aa with 18 gaps, 0 stops, frame= Plus3 at 1355532..1356572 in the same strand
misc_feature no id Transposase and inactivated derivatives [DNA replication, recombination, and repair]; Region: COG3415 intersects 100%(153aa) with IS630 e-value= 7.9e-108 fragment hit coverage= 96.81%, length is 347aa with 18 gaps, 0 stops, frame= Plus3 at 1355532..1356572 in the same strand
misc_feature no id Integrase core domain; Region: rve; cl01316 intersects 100%(109aa) with IS630 e-value= 7.9e-108 fragment hit coverage= 96.81%, length is 347aa with 18 gaps, 0 stops, frame= Plus3 at 1355532..1356572 in the same strand
CDS YP_001971183.1 hypothetical protein intersects 0.19%(0aa) with IS630 e-value= 7.9e-108 fragment hit coverage= 96.81%, length is 347aa with 18 gaps, 0 stops, frame= Plus3 at 1355532..1356572 in the same strand
Class A

16.
CDS YP_001971494.1 putative ISXac3 like transposase protein intersects 100%(284aa) with IS3_IS51_ORF2 e-value= 2.7e-134 complete sequence hit coverage= 100%, length is 303aa with 15 gaps, 0 stops, frame= Minus3 at 1722536..1723443 in the same strand
misc_feature no id putative transposase OrfB; Reviewed; Region: PHA02517 intersects 100%(263aa) with IS3_IS51_ORF2 e-value= 2.7e-134 complete sequence hit coverage= 100%, length is 303aa with 15 gaps, 0 stops, frame= Minus3 at 1722536..1723443 in the same strand
misc_feature no id HMMPfam hit to PF00665, Integrase core domain, score 9e-51 intersects 100%(161aa) with IS3_IS51_ORF2 e-value= 2.7e-134 complete sequence hit coverage= 100%, length is 303aa with 15 gaps, 0 stops, frame= Minus3 at 1722536..1723443 in the same strand
misc_feature no id Integrase core domain; Region: rve; cl01316 intersects 100%(117aa) with IS3_IS51_ORF2 e-value= 2.7e-134 complete sequence hit coverage= 100%, length is 303aa with 15 gaps, 0 stops, frame= Minus3 at 1722536..1723443 in the same strand
CDS YP_001971495.1 putative ISXac3 like transposase intersects 3.23%(3aa) with IS3_IS51_ORF2 e-value= 2.7e-134 complete sequence hit coverage= 100%, length is 303aa with 15 gaps, 0 stops, frame= Minus3 at 1722536..1723443 in the same strand
CDS YP_001971494.1 putative ISXac3 like transposase protein intersects 100%(284aa) with IS3_IS407_ORF2 e-value= 2.1e-06 complete sequence hit coverage= 100%, length is 357aa with 10 gaps, 4 stops, frame= Minus3 at 1722548..1723617 in the same strand
misc_feature no id putative transposase OrfB; Reviewed; Region: PHA02517 intersects 100%(263aa) with IS3_IS407_ORF2 e-value= 2.1e-06 complete sequence hit coverage= 100%, length is 357aa with 10 gaps, 4 stops, frame= Minus3 at 1722548..1723617 in the same strand
misc_feature no id HMMPfam hit to PF00665, Integrase core domain, score 9e-51 intersects 100%(161aa) with IS3_IS407_ORF2 e-value= 2.1e-06 complete sequence hit coverage= 100%, length is 357aa with 10 gaps, 4 stops, frame= Minus3 at 1722548..1723617 in the same strand
misc_feature no id Integrase core domain; Region: rve; cl01316 intersects 100%(117aa) with IS3_IS407_ORF2 e-value= 2.1e-06 complete sequence hit coverage= 100%, length is 357aa with 10 gaps, 4 stops, frame= Minus3 at 1722548..1723617 in the same strand
CDS YP_001971495.1 putative ISXac3 like transposase intersects 65.59%(61aa) with IS3_IS407_ORF2 e-value= 2.1e-06 complete sequence hit coverage= 100%, length is 357aa with 10 gaps, 4 stops, frame= Minus3 at 1722548..1723617 in the same strand
misc_feature no id HMMPfam hit to PF01527, Transposase, score 1.1e-21 intersects 64.10%(50aa) with IS3_IS407_ORF2 e-value= 2.1e-06 complete sequence hit coverage= 100%, length is 357aa with 10 gaps, 4 stops, frame= Minus3 at 1722548..1723617 in the same strand

misc_feature no id Helix-turn-helix domain of Hin and related proteins, a family of DNA-binding domains unique to bacteria and represented by the Hin protein of Salmonella. The basic HTH domain is a simple fold comprised of three core helices that form a right-handed...; Region: HTH_Hin_like; cl01116 intersects 60.87%(42aa) with IS3_IS407_ORF2 e-value= 2.1e-06 complete sequence hit coverage= 100%, length is 357aa with 10 gaps, 4 stops, frame= Minus3 at 1722548..1723617 in the same strand
CDS YP_001971494.1 putative ISXac3 like transposase protein intersects 100%(284aa) with IS3_IS51_ORF1 e-value= 3.7e-108 complete sequence hit coverage= 100%, length is 293aa with 14 gaps, 1 stops, frame= Minus3 at 1722548..1723425 in the same strand
misc_feature no id putative transposase OrfB; Reviewed; Region: PHA02517 intersects 100%(263aa) with IS3_IS51_ORF1 e-value= 3.7e-108 complete sequence hit coverage= 100%, length is 293aa with 14 gaps, 1 stops, frame= Minus3 at 1722548..1723425 in the same strand
misc_feature no id HMMPfam hit to PF00665, Integrase core domain, score 9e-51 intersects 100%(161aa) with IS3_IS51_ORF1 e-value= 3.7e-108 complete sequence hit coverage= 100%, length is 293aa with 14 gaps, 1 stops, frame= Minus3 at 1722548..1723425 in the same strand
misc_feature no id Integrase core domain; Region: rve; cl01316 intersects 100%(117aa) with IS3_IS51_ORF1 e-value= 3.7e-108 complete sequence hit coverage= 100%, length is 293aa with 14 gaps, 1 stops, frame= Minus3 at 1722548..1723425 in the same strand
CDS YP_001971494.1 putative ISXac3 like transposase protein intersects 100%(284aa) with IS3_IS2_ORF2 e-value= 1.4e-07 complete sequence hit coverage= 100%, length is 298aa with 24 gaps, 1 stops, frame= Minus3 at 1722563..1723455 in the same strand
misc_feature no id putative transposase OrfB; Reviewed; Region: PHA02517 intersects 100%(263aa) with IS3_IS2_ORF2 e-value= 1.4e-07 complete sequence hit coverage= 100%, length is 298aa with 24 gaps, 1 stops, frame= Minus3 at 1722563..1723455 in the same strand
misc_feature no id HMMPfam hit to PF00665, Integrase core domain, score 9e-51 intersects 100%(161aa) with IS3_IS2_ORF2 e-value= 1.4e-07 complete sequence hit coverage= 100%, length is 298aa with 24 gaps, 1 stops, frame= Minus3 at 1722563..1723455 in the same strand
misc_feature no id Integrase core domain; Region: rve; cl01316 intersects 100%(117aa) with IS3_IS2_ORF2 e-value= 1.4e-07 complete sequence hit coverage= 100%, length is 298aa with 24 gaps, 1 stops, frame= Minus3 at 1722563..1723455 in the same strand
CDS YP_001971495.1 putative ISXac3 like transposase intersects 7.53%(7aa) with IS3_IS2_ORF2 e-value= 1.4e-07 complete sequence hit coverage= 100%, length is 298aa with 24 gaps, 1 stops, frame= Minus3 at 1722563..1723455 in the same strand
CDS YP_001971494.1 putative ISXac3 like transposase protein intersects 99.18%(281aa) with IS3_IS3_ORF2 e-value= 2e-102 complete sequence hit coverage= 100%, length is 282aa with 7 gaps, 1 stops, frame= Minus3 at 1722566..1723410 in the same strand
misc_feature no id putative transposase OrfB; Reviewed; Region: PHA02517 intersects 100%(263aa) with IS3_IS3_ORF2 e-value= 2e-102 complete sequence hit coverage= 100%, length is 282aa with 7 gaps, 1 stops, frame= Minus3 at 1722566..1723410 in the same strand
misc_feature no id HMMPfam hit to PF00665, Integrase core domain, score 9e-51 intersects 100%(161aa) with IS3_IS3_ORF2 e-value= 2e-102 complete sequence hit coverage= 100%, length is 282aa with 7 gaps, 1 stops, frame= Minus3 at 1722566..1723410 in the same strand
misc_feature no id Integrase core domain; Region: rve; cl01316 intersects 100%(117aa) with IS3_IS3_ORF2 e-value= 2e-102 complete sequence hit coverage= 100%, length is 282aa with 7 gaps, 1 stops, frame= Minus3 at 1722566..1723410 in the same strand
CDS YP_001971494.1 putative ISXac3 like transposase protein intersects 97.07%(275aa) with IS3_IS150_ORF2 e-value= 1.9e-74 fragment hit coverage= 73.52%, length is 276aa with 11 gaps, 0 stops, frame= Minus3 at 1722572..1723398 in the same strand
misc_feature no id putative transposase OrfB; Reviewed; Region: PHA02517 intersects 100%(263aa) with IS3_IS150_ORF2 e-value= 1.9e-74 fragment hit coverage= 73.52%, length is 276aa with 11 gaps, 0 stops, frame= Minus3 at 1722572..1723398 in the same strand
misc_feature no id HMMPfam hit to PF00665, Integrase core domain, score 9e-51 intersects 100%(161aa) with IS3_IS150_ORF2 e-value= 1.9e-74 fragment hit coverage= 73.52%, length is 276aa with 11 gaps, 0 stops, frame= Minus3 at 1722572..1723398 in the same strand
misc_feature no id Integrase core domain; Region: rve; cl01316 intersects 100%(117aa) with IS3_IS150_ORF2 e-value= 1.9e-74 fragment hit coverage= 73.52%, length is 276aa with 11 gaps, 0 stops, frame= Minus3 at 1722572..1723398 in the same strand

CDS YP_001971494.1 putative ISXac3 like transposase protein intersects 99.30%(282aa) with IS3_IS150_ORF2 e-value= 1.2e-45 complete sequence hit coverage= 100%, length is 370aa with 21 gaps, 4 stops, frame= Minus3 at 1722572..1723680 in the same strand
misc_feature no id putative transposase OrfB; Reviewed; Region: PHA02517 intersects 100%(263aa) with IS3_IS150_ORF2 e-value= 1.2e-45 complete sequence hit coverage= 100%, length is 370aa with 21 gaps, 4 stops, frame= Minus3 at 1722572..1723680 in the same strand
misc_feature no id HMMPfam hit to PF00665, Integrase core domain, score 9e-51 intersects 100%(161aa) with IS3_IS150_ORF2 e-value= 1.2e-45 complete sequence hit coverage= 100%, length is 370aa with 21 gaps, 4 stops, frame= Minus3 at 1722572..1723680 in the same strand
misc_feature no id Integrase core domain; Region: rve; cl01316 intersects 100%(117aa) with IS3_IS150_ORF2 e-value= 1.2e-45 complete sequence hit coverage= 100%, length is 370aa with 21 gaps, 4 stops, frame= Minus3 at 1722572..1723680 in the same strand
CDS YP_001971495.1 putative ISXac3 like transposase intersects 88.17%(82aa) with IS3_IS150_ORF2 e-value= 1.2e-45 complete sequence hit coverage= 100%, length is 370aa with 21 gaps, 4 stops, frame= Minus3 at 1722572..1723680 in the same strand
misc_feature no id HMMPfam hit to PF01527, Transposase, score 1.1e-21 intersects 91.03%(71aa) with IS3_IS150_ORF2 e-value= 1.2e-45 complete sequence hit coverage= 100%, length is 370aa with 21 gaps, 4 stops, frame= Minus3 at 1722572..1723680 in the same strand
misc_feature no id Helix-turn-helix domain of Hin and related proteins, a family of DNA-binding domains unique to bacteria and represented by the Hin protein of Salmonella. The basic HTH domain is a simple fold comprised of three core helices that form a right-handed...; Region: HTH_Hin_like; cl01116 intersects 91.30%(63aa) with IS3_IS150_ORF2 e-value= 1.2e-45 complete sequence hit coverage= 100%, length is 370aa with 21 gaps, 4 stops, frame= Minus3 at 1722572..1723680 in the same strand
CDS YP_001971494.1 putative ISXac3 like transposase protein intersects 98.94%(281aa) with IS3_IS150_ORF1 e-value= 5.1e-57 fragment hit coverage= 97.06%, length is 293aa with 7 gaps, 0 stops, frame= Minus3 at 1722575..1723452 in the same strand
misc_feature no id putative transposase OrfB; Reviewed; Region: PHA02517 intersects 100%(263aa) with IS3_IS150_ORF1 e-value= 5.1e-57 fragment hit coverage= 97.06%, length is 293aa with 7 gaps, 0 stops, frame= Minus3 at 1722575..1723452 in the same strand
misc_feature no id HMMPfam hit to PF00665, Integrase core domain, score 9e-51 intersects 100%(161aa) with IS3_IS150_ORF1 e-value= 5.1e-57 fragment hit coverage= 97.06%, length is 293aa with 7 gaps, 0 stops, frame= Minus3 at 1722575..1723452 in the same strand
misc_feature no id Integrase core domain; Region: rve; cl01316 intersects 100%(117aa) with IS3_IS150_ORF1 e-value= 5.1e-57 fragment hit coverage= 97.06%, length is 293aa with 7 gaps, 0 stops, frame= Minus3 at 1722575..1723452 in the same strand
CDS YP_001971495.1 putative ISXac3 like transposase intersects 6.45%(6aa) with IS3_IS150_ORF1 e-value= 5.1e-57 fragment hit coverage= 97.06%, length is 293aa with 7 gaps, 0 stops, frame= Minus3 at 1722575..1723452 in the same strand
CDS YP_001971494.1 putative ISXac3 like transposase protein intersects 98.94%(281aa) with IS3_IS150_ORF1 e-value= 1.4e-57 complete sequence hit coverage= 100%, length is 302aa with 7 gaps, 0 stops, frame= Minus3 at 1722575..1723479 in the same strand
misc_feature no id putative transposase OrfB; Reviewed; Region: PHA02517 intersects 100%(263aa) with IS3_IS150_ORF1 e-value= 1.4e-57 complete sequence hit coverage= 100%, length is 302aa with 7 gaps, 0 stops, frame= Minus3 at 1722575..1723479 in the same strand
misc_feature no id HMMPfam hit to PF00665, Integrase core domain, score 9e-51 intersects 100%(161aa) with IS3_IS150_ORF1 e-value= 1.4e-57 complete sequence hit coverage= 100%, length is 302aa with 7 gaps, 0 stops, frame= Minus3 at 1722575..1723479 in the same strand
misc_feature no id Integrase core domain; Region: rve; cl01316 intersects 100%(117aa) with IS3_IS150_ORF1 e-value= 1.4e-57 complete sequence hit coverage= 100%, length is 302aa with 7 gaps, 0 stops, frame= Minus3 at 1722575..1723479 in the same strand
CDS YP_001971495.1 putative ISXac3 like transposase intersects 16.13%(15aa) with IS3_IS150_ORF1 e-value= 1.4e-57 complete sequence hit coverage= 100%, length is 302aa with 7 gaps, 0 stops, frame= Minus3 at 1722575..1723479 in the same strand
misc_feature no id HMMPfam hit to PF01527, Transposase, score 1.1e-21 intersects 5.13%(4aa) with IS3_IS150_ORF1 e-value= 1.4e-57 complete sequence hit coverage= 100%, length is 302aa with 7 gaps, 0 stops, frame= Minus3 at 1722575..1723479 in the same strand

CDS YP_001971494.1 putative ISXac3 like transposase protein intersects 98.59%(280aa) with IS3_IS3_ORF1 e-value= 4.9e-95 complete sequence hit coverage= 100%, length is 282aa with 6 gaps, 0 stops, frame= Minus3 at 1722578..1723422 in the same strand
misc_feature no id putative transposase OrfB; Reviewed; Region: PHA02517 intersects 100%(263aa) with IS3_IS3_ORF1 e-value= 4.9e-95 complete sequence hit coverage= 100%, length is 282aa with 6 gaps, 0 stops, frame= Minus3 at 1722578..1723422 in the same strand
misc_feature no id HMMPfam hit to PF00665, Integrase core domain, score 9e-51 intersects 100%(161aa) with IS3_IS3_ORF1 e-value= 4.9e-95 complete sequence hit coverage= 100%, length is 282aa with 6 gaps, 0 stops, frame= Minus3 at 1722578..1723422 in the same strand
misc_feature no id Integrase core domain; Region: rve; cl01316 intersects 100%(117aa) with IS3_IS3_ORF1 e-value= 4.9e-95 complete sequence hit coverage= 100%, length is 282aa with 6 gaps, 0 stops, frame= Minus3 at 1722578..1723422 in the same strand
CDS YP_001971494.1 putative ISXac3 like transposase protein intersects 96.01%(272aa) with IS3_IS51_ORF2 e-value= 7.1e-152 fragment hit coverage= 90.22%, length is 273aa with 13 gaps, 0 stops, frame= Minus3 at 1722578..1723395 in the same strand
misc_feature no id putative transposase OrfB; Reviewed; Region: PHA02517 intersects 100%(263aa) with IS3_IS51_ORF2 e-value= 7.1e-152 fragment hit coverage= 90.22%, length is 273aa with 13 gaps, 0 stops, frame= Minus3 at 1722578..1723395 in the same strand
misc_feature no id HMMPfam hit to PF00665, Integrase core domain, score 9e-51 intersects 100%(161aa) with IS3_IS51_ORF2 e-value= 7.1e-152 fragment hit coverage= 90.22%, length is 273aa with 13 gaps, 0 stops, frame= Minus3 at 1722578..1723395 in the same strand
misc_feature no id Integrase core domain; Region: rve; cl01316 intersects 100%(117aa) with IS3_IS51_ORF2 e-value= 7.1e-152 fragment hit coverage= 90.22%, length is 273aa with 13 gaps, 0 stops, frame= Minus3 at 1722578..1723395 in the same strand
CDS YP_001971494.1 putative ISXac3 like transposase protein intersects 98.24%(279aa) with IS3_IS51_ORF1 e-value= 3.9e-110 fragment hit coverage= 96.42%, length is 282aa with 14 gaps, 0 stops, frame= Minus3 at 1722581..1723425 in the same strand
misc_feature no id putative transposase OrfB; Reviewed; Region: PHA02517 intersects 100%(263aa) with IS3_IS51_ORF1 e-value= 3.9e-110 fragment hit coverage= 96.42%, length is 282aa with 14 gaps, 0 stops, frame= Minus3 at 1722581..1723425 in the same strand
misc_feature no id HMMPfam hit to PF00665, Integrase core domain, score 9e-51 intersects 100%(161aa) with IS3_IS51_ORF1 e-value= 3.9e-110 fragment hit coverage= 96.42%, length is 282aa with 14 gaps, 0 stops, frame= Minus3 at 1722581..1723425 in the same strand
misc_feature no id Integrase core domain; Region: rve; cl01316 intersects 100%(117aa) with IS3_IS51_ORF1 e-value= 3.9e-110 fragment hit coverage= 96.42%, length is 282aa with 14 gaps, 0 stops, frame= Minus3 at 1722581..1723425 in the same strand
CDS YP_001971494.1 putative ISXac3 like transposase protein intersects 0.12%(0aa) with IS3_IS51_ORF1 e-value= 3.2e-08 fragment hit coverage= 14.66%, length is 43aa with 2 gaps, 1 stops, frame= Minus1 at 1723417..1723544 in the same strand
CDS YP_001971495.1 putative ISXac3 like transposase intersects 39.43%(36aa) with IS3_IS51_ORF1 e-value= 3.2e-08 fragment hit coverage= 14.66%, length is 43aa with 2 gaps, 1 stops, frame= Minus1 at 1723417..1723544 in the same strand
misc_feature no id HMMPfam hit to PF01527, Transposase, score 1.1e-21 intersects 32.91%(25aa) with IS3_IS51_ORF1 e-value= 3.2e-08 fragment hit coverage= 14.66%, length is 43aa with 2 gaps, 1 stops, frame= Minus1 at 1723417..1723544 in the same strand
misc_feature no id Helix-turn-helix domain of Hin and related proteins, a family of DNA-binding domains unique to bacteria and represented by the Hin protein of Salmonella. The basic HTH domain is a simple fold comprised of three core helices that form a right-handed...; Region: HTH_Hin_like; cl01116 intersects 25.60%(17aa) with IS3_IS51_ORF1 e-value= 3.2e-08 fragment hit coverage= 14.66%, length is 43aa with 2 gaps, 1 stops, frame= Minus1 at 1723417..1723544 in the same strand
Class A

17.
CDS YP_001971495.1 putative ISXac3 like transposase intersects 51.25%(47aa) with IS3_IS407_ORF1 e-value= 7.7e-06 fragment hit coverage= 55.81%, length is 48aa with 0 gaps, 0 stops, frame= Minus1 at 1723555..1723697 in the same strand

misc_feature no id HMMPfam hit to PF01527, Transposase, score 1.1e-21 intersects 61.11%(47aa) with IS3_IS407_ORF1 e-value= 7.7e-06 fragment hit coverage= 55.81%, length is 48aa with 0 gaps, 0 stops, frame= Minus1 at 1723555..1723697 in the same strand
misc_feature no id Helix-turn-helix domain of Hin and related proteins, a family of DNA-binding domains unique to bacteria and represented by the Hin protein of Salmonella. The basic HTH domain is a simple fold comprised of three core helices that form a right-handed...; Region: HTH_Hin_like; cl01116 intersects 69.08%(47aa) with IS3_IS407_ORF1 e-value= 7.7e-06 fragment hit coverage= 55.81%, length is 48aa with 0 gaps, 0 stops, frame= Minus1 at 1723555..1723697 in the same strand
Class A

18.
repeat_region no id no product intersects 67.85%(306aa) with IS110 e-value= 3.4e-46 complete sequence hit coverage= 100%, length is 306aa with 110 gaps, 1 stops, frame= Plus1 at 1775491..1776408 in the opposite strand
CDS YP_001971560.1 putative transposase intersects 99.67%(304aa) with IS110 e-value= 3.4e-46 complete sequence hit coverage= 100%, length is 306aa with 110 gaps, 1 stops, frame= Plus1 at 1775491..1776408 in the same strand
misc_feature no id Transposase and inactivated derivatives [DNA replication, recombination, and repair]; Region: COG3547 intersects 100%(292aa) with IS110 e-value= 3.4e-46 complete sequence hit coverage= 100%, length is 306aa with 110 gaps, 1 stops, frame= Plus1 at 1775491..1776408 in the same strand
misc_feature no id Transposase; Region: Transposase_9; pfam01548 intersects 100%(79aa) with IS110 e-value= 3.4e-46 complete sequence hit coverage= 100%, length is 306aa with 110 gaps, 1 stops, frame= Plus1 at 1775491..1776408 in the same strand
misc_feature no id HMMPfam hit to PF01548, Transposase, score 1.8e-06 intersects 100%(98aa) with IS110 e-value= 3.4e-46 complete sequence hit coverage= 100%, length is 306aa with 110 gaps, 1 stops, frame= Plus1 at 1775491..1776408 in the same strand
misc_feature no id HMMPfam hit to PF02371, Transposase IS116/IS110/IS902 family, score 4.3e-27 intersects 100%(108aa) with IS110 e-value= 3.4e-46 complete sequence hit coverage= 100%, length is 306aa with 110 gaps, 1 stops, frame= Plus1 at 1775491..1776408 in the same strand
misc_feature no id Transposase IS116/IS110/IS902 family; Region: Transposase_20; pfam02371 intersects 100%(84aa) with IS110 e-value= 3.4e-46 complete sequence hit coverage= 100%, length is 306aa with 110 gaps, 1 stops, frame= Plus1 at 1775491..1776408 in the same strand
repeat_region no id no product intersects 65.41%(295aa) with IS110 e-value= 1e-48 fragment hit coverage= 96.88%, length is 295aa with 108 gaps, 0 stops, frame= Plus1 at 1775491..1776375 in the opposite strand
CDS YP_001971560.1 putative transposase intersects 96.72%(295aa) with IS110 e-value= 1e-48 fragment hit coverage= 96.88%, length is 295aa with 108 gaps, 0 stops, frame= Plus1 at 1775491..1776375 in the same strand
misc_feature no id Transposase and inactivated derivatives [DNA replication, recombination, and repair]; Region: COG3547 intersects 100%(292aa) with IS110 e-value= 1e-48 fragment hit coverage= 96.88%, length is 295aa with 108 gaps, 0 stops, frame= Plus1 at 1775491..1776375 in the same strand
misc_feature no id Transposase; Region: Transposase_9; pfam01548 intersects 100%(79aa) with IS110 e-value= 1e-48 fragment hit coverage= 96.88%, length is 295aa with 108 gaps, 0 stops, frame= Plus1 at 1775491..1776375 in the same strand
misc_feature no id HMMPfam hit to PF01548, Transposase, score 1.8e-06 intersects 100%(98aa) with IS110 e-value= 1e-48 fragment hit coverage= 96.88%, length is 295aa with 108 gaps, 0 stops, frame= Plus1 at 1775491..1776375 in the same strand
misc_feature no id HMMPfam hit to PF02371, Transposase IS116/IS110/IS902 family, score 4.3e-27 intersects 100%(108aa) with IS110 e-value= 1e-48 fragment hit coverage= 96.88%, length is 295aa with 108 gaps, 0 stops, frame= Plus1 at 1775491..1776375 in the same strand
misc_feature no id Transposase IS116/IS110/IS902 family; Region: Transposase_20; pfam02371 intersects 100%(84aa) with IS110 e-value= 1e-48 fragment hit coverage= 96.88%, length is 295aa with 108 gaps, 0 stops, frame= Plus1 at 1775491..1776375 in the same strand
Class A

19.
CDS YP_001971671.1 putative ISXac3 like transposase intersects 52.75%(48aa) with IS3_IS407_ORF1 e-value= 7.7e-06 fragment hit coverage= 55.81%, length is 48aa with 0 gaps, 0 stops, frame= Plus2 at 1896455..1896598 in the same strand

misc_feature no id HMMPfam hit to PF01527, Transposase, score 1.1e-21 intersects 61.54%(48aa) with IS3_IS407_ORF1 e-value= 7.7e-06 fragment hit coverage= 55.81%, length is 48aa with 0 gaps, 0 stops, frame= Plus2 at 1896455..1896598 in the same strand
misc_feature no id Helix-turn-helix domain of Hin and related proteins, a family of DNA-binding domains unique to bacteria and represented by the Hin protein of Salmonella. The basic HTH domain is a simple fold comprised of three core helices that form a right-handed...; Region: HTH_Hin_like; cl01116 intersects 69.57%(48aa) with IS3_IS407_ORF1 e-value= 7.7e-06 fragment hit coverage= 55.81%, length is 48aa with 0 gaps, 0 stops, frame= Plus2 at 1896455..1896598 in the same strand
CDS YP_001971671.1 putative ISXac3 like transposase intersects 90.48%(82aa) with IS3_IS150_ORF2 e-value= 1.2e-45 complete sequence hit coverage= 100%, length is 370aa with 21 gaps, 4 stops, frame= Plus1 at 1896472..1897581 in the same strand
misc_feature no id HMMPfam hit to PF01527, Transposase, score 1.1e-21 intersects 91.45%(71aa) with IS3_IS150_ORF2 e-value= 1.2e-45 complete sequence hit coverage= 100%, length is 370aa with 21 gaps, 4 stops, frame= Plus1 at 1896472..1897581 in the same strand
misc_feature no id Helix-turn-helix domain of Hin and related proteins, a family of DNA-binding domains unique to bacteria and represented by the Hin protein of Salmonella. The basic HTH domain is a simple fold comprised of three core helices that form a right-handed...; Region: HTH_Hin_like; cl01116 intersects 91.79%(63aa) with IS3_IS150_ORF2 e-value= 1.2e-45 complete sequence hit coverage= 100%, length is 370aa with 21 gaps, 4 stops, frame= Plus1 at 1896472..1897581 in the same strand
CDS YP_001971672.1 putative ISXac3 like transposase integrase intersects 99.30%(282aa) with IS3_IS150_ORF2 e-value= 1.2e-45 complete sequence hit coverage= 100%, length is 370aa with 21 gaps, 4 stops, frame= Plus1 at 1896472..1897581 in the same strand
misc_feature no id putative transposase OrfB; Reviewed; Region: PHA02517 intersects 100%(263aa) with IS3_IS150_ORF2 e-value= 1.2e-45 complete sequence hit coverage= 100%, length is 370aa with 21 gaps, 4 stops, frame= Plus1 at 1896472..1897581 in the same strand
misc_feature no id HMMPfam hit to PF00665, Integrase core domain, score 9e-51 intersects 100%(161aa) with IS3_IS150_ORF2 e-value= 1.2e-45 complete sequence hit coverage= 100%, length is 370aa with 21 gaps, 4 stops, frame= Plus1 at 1896472..1897581 in the same strand
misc_feature no id Integrase core domain; Region: rve; cl01316 intersects 100%(117aa) with IS3_IS150_ORF2 e-value= 1.2e-45 complete sequence hit coverage= 100%, length is 370aa with 21 gaps, 4 stops, frame= Plus1 at 1896472..1897581 in the same strand
Class A

20.
CDS YP_001971671.1 putative ISXac3 like transposase intersects 40.66%(37aa) with IS3_IS51_ORF1 e-value= 3.2e-08 fragment hit coverage= 14.66%, length is 43aa with 2 gaps, 1 stops, frame= Plus2 at 1896608..1896736 in the same strand
misc_feature no id HMMPfam hit to PF01527, Transposase, score 1.1e-21 intersects 33.33%(26aa) with IS3_IS51_ORF1 e-value= 3.2e-08 fragment hit coverage= 14.66%, length is 43aa with 2 gaps, 1 stops, frame= Plus2 at 1896608..1896736 in the same strand
misc_feature no id Helix-turn-helix domain of Hin and related proteins, a family of DNA-binding domains unique to bacteria and represented by the Hin protein of Salmonella. The basic HTH domain is a simple fold comprised of three core helices that form a right-handed...; Region: HTH_Hin_like; cl01116 intersects 26.09%(18aa) with IS3_IS51_ORF1 e-value= 3.2e-08 fragment hit coverage= 14.66%, length is 43aa with 2 gaps, 1 stops, frame= Plus2 at 1896608..1896736 in the same strand
CDS YP_001971672.1 putative ISXac3 like transposase integrase intersects 0.12%(0aa) with IS3_IS51_ORF1 e-value= 3.2e-08 fragment hit coverage= 14.66%, length is 43aa with 2 gaps, 1 stops, frame= Plus2 at 1896608..1896736 in the same strand
CDS YP_001971671.1 putative ISXac3 like transposase intersects 16.85%(15aa) with IS3_IS150_ORF1 e-value= 1.4e-57 complete sequence hit coverage= 100%, length is 302aa with 7 gaps, 0 stops, frame= Plus1 at 1896673..1897578 in the same strand
misc_feature no id HMMPfam hit to PF01527, Transposase, score 1.1e-21 intersects 5.56%(4aa) with IS3_IS150_ORF1 e-value= 1.4e-57 complete sequence hit coverage= 100%, length is 302aa with 7 gaps, 0 stops, frame= Plus1 at 1896673..1897578 in the same strand
CDS YP_001971672.1 putative ISXac3 like transposase integrase intersects 98.94%(281aa) with IS3_IS150_ORF1 e-value= 1.4e-57 complete sequence hit coverage= 100%, length is 302aa with 7 gaps, 0 stops, frame= Plus1 at 1896673..1897578 in the same strand

misc_feature no id putative transposase OrfB; Reviewed; Region: PHA02517 intersects 100%(263aa) with IS3_IS150_ORF1 e-value= 1.4e-57 complete sequence hit coverage= 100%, length is 302aa with 7 gaps, 0 stops, frame= Plus1 at 1896673..1897578 in the same strand
misc_feature no id HMMPfam hit to PF00665, Integrase core domain, score 9e-51 intersects 100%(161aa) with IS3_IS150_ORF1 e-value= 1.4e-57 complete sequence hit coverage= 100%, length is 302aa with 7 gaps, 0 stops, frame= Plus1 at 1896673..1897578 in the same strand
misc_feature no id Integrase core domain; Region: rve; cl01316 intersects 100%(117aa) with IS3_IS150_ORF1 e-value= 1.4e-57 complete sequence hit coverage= 100%, length is 302aa with 7 gaps, 0 stops, frame= Plus1 at 1896673..1897578 in the same strand
CDS YP_001971671.1 putative ISXac3 like transposase intersects 8.06%(7aa) with IS3_IS2_ORF2 e-value= 1.4e-07 complete sequence hit coverage= 100%, length is 298aa with 24 gaps, 1 stops, frame= Plus1 at 1896697..1897590 in the same strand
CDS YP_001971672.1 putative ISXac3 like transposase integrase intersects 100%(284aa) with IS3_IS2_ORF2 e-value= 1.4e-07 complete sequence hit coverage= 100%, length is 298aa with 24 gaps, 1 stops, frame= Plus1 at 1896697..1897590 in the same strand
misc_feature no id putative transposase OrfB; Reviewed; Region: PHA02517 intersects 100%(263aa) with IS3_IS2_ORF2 e-value= 1.4e-07 complete sequence hit coverage= 100%, length is 298aa with 24 gaps, 1 stops, frame= Plus1 at 1896697..1897590 in the same strand
misc_feature no id HMMPfam hit to PF00665, Integrase core domain, score 9e-51 intersects 100%(161aa) with IS3_IS2_ORF2 e-value= 1.4e-07 complete sequence hit coverage= 100%, length is 298aa with 24 gaps, 1 stops, frame= Plus1 at 1896697..1897590 in the same strand
misc_feature no id Integrase core domain; Region: rve; cl01316 intersects 100%(117aa) with IS3_IS2_ORF2 e-value= 1.4e-07 complete sequence hit coverage= 100%, length is 298aa with 24 gaps, 1 stops, frame= Plus1 at 1896697..1897590 in the same strand
CDS YP_001971671.1 putative ISXac3 like transposase intersects 6.96%(6aa) with IS3_IS150_ORF1 e-value= 5.1e-57 fragment hit coverage= 97.06%, length is 293aa with 7 gaps, 0 stops, frame= Plus1 at 1896700..1897578 in the same strand
CDS YP_001971672.1 putative ISXac3 like transposase integrase intersects 98.94%(281aa) with IS3_IS150_ORF1 e-value= 5.1e-57 fragment hit coverage= 97.06%, length is 293aa with 7 gaps, 0 stops, frame= Plus1 at 1896700..1897578 in the same strand
misc_feature no id putative transposase OrfB; Reviewed; Region: PHA02517 intersects 100%(263aa) with IS3_IS150_ORF1 e-value= 5.1e-57 fragment hit coverage= 97.06%, length is 293aa with 7 gaps, 0 stops, frame= Plus1 at 1896700..1897578 in the same strand
misc_feature no id HMMPfam hit to PF00665, Integrase core domain, score 9e-51 intersects 100%(161aa) with IS3_IS150_ORF1 e-value= 5.1e-57 fragment hit coverage= 97.06%, length is 293aa with 7 gaps, 0 stops, frame= Plus1 at 1896700..1897578 in the same strand
misc_feature no id Integrase core domain; Region: rve; cl01316 intersects 100%(117aa) with IS3_IS150_ORF1 e-value= 5.1e-57 fragment hit coverage= 97.06%, length is 293aa with 7 gaps, 0 stops, frame= Plus1 at 1896700..1897578 in the same strand
CDS YP_001971671.1 putative ISXac3 like transposase intersects 3.66%(3aa) with IS3_IS51_ORF2 e-value= 1.4e-134 complete sequence hit coverage= 100%, length is 303aa with 15 gaps, 0 stops, frame= Plus1 at 1896709..1897617 in the same strand
CDS YP_001971672.1 putative ISXac3 like transposase integrase intersects 100%(284aa) with IS3_IS51_ORF2 e-value= 1.4e-134 complete sequence hit coverage= 100%, length is 303aa with 15 gaps, 0 stops, frame= Plus1 at 1896709..1897617 in the same strand
misc_feature no id putative transposase OrfB; Reviewed; Region: PHA02517 intersects 100%(263aa) with IS3_IS51_ORF2 e-value= 1.4e-134 complete sequence hit coverage= 100%, length is 303aa with 15 gaps, 0 stops, frame= Plus1 at 1896709..1897617 in the same strand
misc_feature no id HMMPfam hit to PF00665, Integrase core domain, score 9e-51 intersects 100%(161aa) with IS3_IS51_ORF2 e-value= 1.4e-134 complete sequence hit coverage= 100%, length is 303aa with 15 gaps, 0 stops, frame= Plus1 at 1896709..1897617 in the same strand
misc_feature no id Integrase core domain; Region: rve; cl01316 intersects 100%(117aa) with IS3_IS51_ORF2 e-value= 1.4e-134 complete sequence hit coverage= 100%, length is 303aa with 15 gaps, 0 stops, frame= Plus1 at 1896709..1897617 in the same strand
CDS YP_001971672.1 putative ISXac3 like transposase integrase intersects 100%(284aa) with IS3_IS51_ORF1 e-value= 3.7e-108 complete sequence hit coverage= 100%, length is 293aa with 14 gaps, 1 stops, frame= Plus1 at 1896727..1897605 in the same strand

misc_feature no id putative transposase OrfB; Reviewed; Region: PHA02517 intersects 100%(263aa) with IS3_IS51_ORF1 e-value= 3.7e-108 complete sequence hit coverage= 100%, length is 293aa with 14 gaps, 1 stops, frame= Plus1 at 1896727..1897605 in the same strand
misc_feature no id HMMPfam hit to PF00665, Integrase core domain, score 9e-51 intersects 100%(161aa) with IS3_IS51_ORF1 e-value= 3.7e-108 complete sequence hit coverage= 100%, length is 293aa with 14 gaps, 1 stops, frame= Plus1 at 1896727..1897605 in the same strand
misc_feature no id Integrase core domain; Region: rve; cl01316 intersects 100%(117aa) with IS3_IS51_ORF1 e-value= 3.7e-108 complete sequence hit coverage= 100%, length is 293aa with 14 gaps, 1 stops, frame= Plus1 at 1896727..1897605 in the same strand
CDS YP_001971672.1 putative ISXac3 like transposase integrase intersects 98.24%(279aa) with IS3_IS51_ORF1 e-value= 3.9e-110 fragment hit coverage= 96.42%, length is 282aa with 14 gaps, 0 stops, frame= Plus1 at 1896727..1897572 in the same strand
misc_feature no id putative transposase OrfB; Reviewed; Region: PHA02517 intersects 100%(263aa) with IS3_IS51_ORF1 e-value= 3.9e-110 fragment hit coverage= 96.42%, length is 282aa with 14 gaps, 0 stops, frame= Plus1 at 1896727..1897572 in the same strand
misc_feature no id HMMPfam hit to PF00665, Integrase core domain, score 9e-51 intersects 100%(161aa) with IS3_IS51_ORF1 e-value= 3.9e-110 fragment hit coverage= 96.42%, length is 282aa with 14 gaps, 0 stops, frame= Plus1 at 1896727..1897572 in the same strand
misc_feature no id Integrase core domain; Region: rve; cl01316 intersects 100%(117aa) with IS3_IS51_ORF1 e-value= 3.9e-110 fragment hit coverage= 96.42%, length is 282aa with 14 gaps, 0 stops, frame= Plus1 at 1896727..1897572 in the same strand
CDS YP_001971672.1 putative ISXac3 like transposase integrase intersects 98.59%(280aa) with IS3_IS3_ORF1 e-value= 4.9e-95 complete sequence hit coverage= 100%, length is 282aa with 6 gaps, 0 stops, frame= Plus1 at 1896730..1897575 in the same strand
misc_feature no id putative transposase OrfB; Reviewed; Region: PHA02517 intersects 100%(263aa) with IS3_IS3_ORF1 e-value= 4.9e-95 complete sequence hit coverage= 100%, length is 282aa with 6 gaps, 0 stops, frame= Plus1 at 1896730..1897575 in the same strand
misc_feature no id HMMPfam hit to PF00665, Integrase core domain, score 9e-51 intersects 100%(161aa) with IS3_IS3_ORF1 e-value= 4.9e-95 complete sequence hit coverage= 100%, length is 282aa with 6 gaps, 0 stops, frame= Plus1 at 1896730..1897575 in the same strand
misc_feature no id Integrase core domain; Region: rve; cl01316 intersects 100%(117aa) with IS3_IS3_ORF1 e-value= 4.9e-95 complete sequence hit coverage= 100%, length is 282aa with 6 gaps, 0 stops, frame= Plus1 at 1896730..1897575 in the same strand
Class A

21.
CDS YP_001971672.1 putative ISXac3 like transposase integrase intersects 99.30%(282aa) with IS3_IS3_ORF2 e-value= 2e-102 complete sequence hit coverage= 100%, length is 282aa with 7 gaps, 1 stops, frame= Plus1 at 1896742..1897587 in the same strand
misc_feature no id putative transposase OrfB; Reviewed; Region: PHA02517 intersects 100%(263aa) with IS3_IS3_ORF2 e-value= 2e-102 complete sequence hit coverage= 100%, length is 282aa with 7 gaps, 1 stops, frame= Plus1 at 1896742..1897587 in the same strand
misc_feature no id HMMPfam hit to PF00665, Integrase core domain, score 9e-51 intersects 100%(161aa) with IS3_IS3_ORF2 e-value= 2e-102 complete sequence hit coverage= 100%, length is 282aa with 7 gaps, 1 stops, frame= Plus1 at 1896742..1897587 in the same strand
misc_feature no id Integrase core domain; Region: rve; cl01316 intersects 100%(117aa) with IS3_IS3_ORF2 e-value= 2e-102 complete sequence hit coverage= 100%, length is 282aa with 7 gaps, 1 stops, frame= Plus1 at 1896742..1897587 in the same strand
CDS YP_001971672.1 putative ISXac3 like transposase integrase intersects 97.18%(276aa) with IS3_IS150_ORF2 e-value= 1.9e-74 fragment hit coverage= 73.52%, length is 276aa with 11 gaps, 0 stops, frame= Plus1 at 1896754..1897581 in the same strand
misc_feature no id putative transposase OrfB; Reviewed; Region: PHA02517 intersects 100%(263aa) with IS3_IS150_ORF2 e-value= 1.9e-74 fragment hit coverage= 73.52%, length is 276aa with 11 gaps, 0 stops, frame= Plus1 at 1896754..1897581 in the same strand
misc_feature no id HMMPfam hit to PF00665, Integrase core domain, score 9e-51 intersects 100%(161aa) with IS3_IS150_ORF2 e-value= 1.9e-74 fragment hit coverage= 73.52%, length is 276aa with 11 gaps, 0 stops, frame= Plus1 at 1896754..1897581 in the same strand

misc_feature no id Integrase core domain; Region: rve; cl01316 intersects 100%(117aa) with IS3_IS150_ORF2 e-value= 1.9e-74 fragment hit coverage= 73.52%, length is 276aa with 11 gaps, 0 stops, frame= Plus1 at 1896754..1897581 in the same strand
CDS YP_001971672.1 putative ISXac3 like transposase integrase intersects 96.13%(273aa) with IS3_IS51_ORF2 e-value= 7.1e-152 fragment hit coverage= 90.22%, length is 273aa with 13 gaps, 0 stops, frame= Plus1 at 1896757..1897575 in the same strand
misc_feature no id putative transposase OrfB; Reviewed; Region: PHA02517 intersects 100%(263aa) with IS3_IS51_ORF2 e-value= 7.1e-152 fragment hit coverage= 90.22%, length is 273aa with 13 gaps, 0 stops, frame= Plus1 at 1896757..1897575 in the same strand
misc_feature no id HMMPfam hit to PF00665, Integrase core domain, score 9e-51 intersects 100%(161aa) with IS3_IS51_ORF2 e-value= 7.1e-152 fragment hit coverage= 90.22%, length is 273aa with 13 gaps, 0 stops, frame= Plus1 at 1896757..1897575 in the same strand
misc_feature no id Integrase core domain; Region: rve; cl01316 intersects 100%(117aa) with IS3_IS51_ORF2 e-value= 7.1e-152 fragment hit coverage= 90.22%, length is 273aa with 13 gaps, 0 stops, frame= Plus1 at 1896757..1897575 in the same strand
Class A

22.
CDS YP_001971706.1 putative transposase intersects 11.75%(43aa) with IS3_IS51_ORF2 e-value= 2.3e-08 fragment hit coverage= 12.93%, length is 43aa with 0 gaps, 0 stops, frame= Plus1 at 1925629..1925757 in the same strand
misc_feature no id Transposase and inactivated derivatives [DNA replication, recombination, and repair]; Region: Tra5; COG2801 intersects 18.22%(43aa) with IS3_IS51_ORF2 e-value= 2.3e-08 fragment hit coverage= 12.93%, length is 43aa with 0 gaps, 0 stops, frame= Plus1 at 1925629..1925757 in the same strand
misc_feature no id HMMPfam hit to PF00665, Integrase core domain, score 2.2e-26 intersects 25.60%(43aa) with IS3_IS51_ORF2 e-value= 2.3e-08 fragment hit coverage= 12.93%, length is 43aa with 0 gaps, 0 stops, frame= Plus1 at 1925629..1925757 in the same strand
misc_feature no id Integrase core domain; Region: rve; cl01316 intersects 34.15%(42aa) with IS3_IS51_ORF2 e-value= 2.3e-08 fragment hit coverage= 12.93%, length is 43aa with 0 gaps, 0 stops, frame= Plus1 at 1925629..1925757 in the same strand
CDS YP_001971706.1 putative transposase intersects 44.81%(164aa) with IS3_IS150_ORF2 e-value= 2.6e-08 fragment hit coverage= 42.16%, length is 164aa with 8 gaps, 0 stops, frame= Plus1 at 1925632..1926123 in the same strand
misc_feature no id Transposase and inactivated derivatives [DNA replication, recombination, and repair]; Region: Tra5; COG2801 intersects 69.49%(164aa) with IS3_IS150_ORF2 e-value= 2.6e-08 fragment hit coverage= 42.16%, length is 164aa with 8 gaps, 0 stops, frame= Plus1 at 1925632..1926123 in the same strand
misc_feature no id HMMPfam hit to PF00665, Integrase core domain, score 2.2e-26 intersects 97.62%(164aa) with IS3_IS150_ORF2 e-value= 2.6e-08 fragment hit coverage= 42.16%, length is 164aa with 8 gaps, 0 stops, frame= Plus1 at 1925632..1926123 in the same strand
misc_feature no id Integrase core domain; Region: rve; cl01316 intersects 100%(123aa) with IS3_IS150_ORF2 e-value= 2.6e-08 fragment hit coverage= 42.16%, length is 164aa with 8 gaps, 0 stops, frame= Plus1 at 1925632..1926123 in the same strand
repeat_region no id no product intersects 100%(2aa) with IS3_IS150_ORF2 e-value= 2.6e-08 fragment hit coverage= 42.16%, length is 164aa with 8 gaps, 0 stops, frame= Plus1 at 1925632..1926123 in the same strand
Class A

23.
CDS YP_001971818.1 RNA polymerase sigma factor intersects 17.17%(39aa) with IS21_ORF1 e-value= 2.4e-06 fragment hit coverage= 7.62%, length is 40aa with 0 gaps, 0 stops, frame= Minus2 at 2023779..2023897 in the same strand
misc_feature no id RNA polymerase sigma factor; Provisional; Region: PRK12513 intersects 21.21%(39aa) with IS21_ORF1 e-value= 2.4e-06 fragment hit coverage= 7.62%, length is 40aa with 0 gaps, 0 stops, frame= Minus2 at 2023779..2023897 in the same strand
misc_feature no id HMMPfam hit to PF04545, Sigma-70, region, score 1.6e-20 intersects 79.33%(39aa) with IS21_ORF1 e-value= 2.4e-06 fragment hit coverage= 7.62%, length is 40aa with 0 gaps, 0 stops, frame= Minus2 at 2023779..2023897 in the same strand

misc_feature no id Sigma70, region (SR) 4 refers to the most C-terminal of four conserved domains found in Escherichia coli (Ec) sigma70, the main housekeeping sigma, and related sigma-factors (SFs). A SF is a dissociable subunit of RNA polymerase, it directs bacterial...; Region: Sigma70_r4; cd06171 intersects 72.12%(39aa) with IS21_ORF1 e-value= 2.4e-06 fragment hit coverage= 7.62%, length is 40aa with 0 gaps, 0 stops, frame= Minus2 at 2023779..2023897 in the same strand
misc_feature no id HMMPfam hit to PF08281, Sigma-70, region, score 2.6e-12 intersects 73.46%(39aa) with IS21_ORF1 e-value= 2.4e-06 fragment hit coverage= 7.62%, length is 40aa with 0 gaps, 0 stops, frame= Minus2 at 2023779..2023897 in the same strand
misc_feature no id DNA binding residues intersects 100%(1aa) with IS21_ORF1 e-value= 2.4e-06 fragment hit coverage= 7.62%, length is 40aa with 0 gaps, 0 stops, frame= Minus2 at 2023779..2023897 in the same strand
Class B

24.
repeat_region no id no product intersects 65.31%(295aa) with IS110 e-value= 1e-48 fragment hit coverage= 96.88%, length is 295aa with 108 gaps, 0 stops, frame= Plus1 at 2293057..2293941 in the opposite strand
CDS YP_001972049.1 putative transposase intersects 96.72%(295aa) with IS110 e-value= 1e-48 fragment hit coverage= 96.88%, length is 295aa with 108 gaps, 0 stops, frame= Plus1 at 2293057..2293941 in the same strand
misc_feature no id Transposase and inactivated derivatives [DNA replication, recombination, and repair]; Region: COG3547 intersects 100%(292aa) with IS110 e-value= 1e-48 fragment hit coverage= 96.88%, length is 295aa with 108 gaps, 0 stops, frame= Plus1 at 2293057..2293941 in the same strand
misc_feature no id HMMPfam hit to PF01548, Transposase, score 1.8e-06 intersects 100%(98aa) with IS110 e-value= 1e-48 fragment hit coverage= 96.88%, length is 295aa with 108 gaps, 0 stops, frame= Plus1 at 2293057..2293941 in the same strand
misc_feature no id Transposase; Region: Transposase_9; pfam01548 intersects 100%(79aa) with IS110 e-value= 1e-48 fragment hit coverage= 96.88%, length is 295aa with 108 gaps, 0 stops, frame= Plus1 at 2293057..2293941 in the same strand
misc_feature no id HMMPfam hit to PF02371, Transposase IS116/IS110/IS902 family, score 4.3e-27 intersects 100%(108aa) with IS110 e-value= 1e-48 fragment hit coverage= 96.88%, length is 295aa with 108 gaps, 0 stops, frame= Plus1 at 2293057..2293941 in the same strand
misc_feature no id Transposase IS116/IS110/IS902 family; Region: Transposase_20; pfam02371 intersects 100%(84aa) with IS110 e-value= 1e-48 fragment hit coverage= 96.88%, length is 295aa with 108 gaps, 0 stops, frame= Plus1 at 2293057..2293941 in the same strand
repeat_region no id no product intersects 67.75%(306aa) with IS110 e-value= 3.4e-46 complete sequence hit coverage= 100%, length is 306aa with 110 gaps, 1 stops, frame= Plus1 at 2293057..2293974 in the opposite strand
CDS YP_001972049.1 putative transposase intersects 99.67%(304aa) with IS110 e-value= 3.4e-46 complete sequence hit coverage= 100%, length is 306aa with 110 gaps, 1 stops, frame= Plus1 at 2293057..2293974 in the same strand
misc_feature no id Transposase and inactivated derivatives [DNA replication, recombination, and repair]; Region: COG3547 intersects 100%(292aa) with IS110 e-value= 3.4e-46 complete sequence hit coverage= 100%, length is 306aa with 110 gaps, 1 stops, frame= Plus1 at 2293057..2293974 in the same strand
misc_feature no id HMMPfam hit to PF01548, Transposase, score 1.8e-06 intersects 100%(98aa) with IS110 e-value= 3.4e-46 complete sequence hit coverage= 100%, length is 306aa with 110 gaps, 1 stops, frame= Plus1 at 2293057..2293974 in the same strand
misc_feature no id Transposase; Region: Transposase_9; pfam01548 intersects 100%(79aa) with IS110 e-value= 3.4e-46 complete sequence hit coverage= 100%, length is 306aa with 110 gaps, 1 stops, frame= Plus1 at 2293057..2293974 in the same strand
misc_feature no id HMMPfam hit to PF02371, Transposase IS116/IS110/IS902 family, score 4.3e-27 intersects 100%(108aa) with IS110 e-value= 3.4e-46 complete sequence hit coverage= 100%, length is 306aa with 110 gaps, 1 stops, frame= Plus1 at 2293057..2293974 in the same strand
misc_feature no id Transposase IS116/IS110/IS902 family; Region: Transposase_20; pfam02371 intersects 100%(84aa) with IS110 e-value= 3.4e-46 complete sequence hit coverage= 100%, length is 306aa with 110 gaps, 1 stops, frame= Plus1 at 2293057..2293974 in the same strand
Class A

25.

CDS YP_001972086.1 putative ISPsy9 like transposase intersects 42.15%(73aa) with IS3_IS150_ORF2 e-value= 5e-121 complete sequence hit coverage= 100%, length is 350aa with 42 gaps, 3 stops, frame= Plus3 at 2334351..2335400 in the same strand
CDS YP_001972087.1 putative ISPsy9 like transposase for insertion sequence intersects 100%(278aa) with IS3_IS150_ORF2 e-value= 5e-121 complete sequence hit coverage= 100%, length is 350aa with 42 gaps, 3 stops, frame= Plus3 at 2334351..2335400 in the same strand
misc_feature no id putative transposase OrfB; Reviewed; Region: PHA02517 intersects 100%(251aa) with IS3_IS150_ORF2 e-value= 5e-121 complete sequence hit coverage= 100%, length is 350aa with 42 gaps, 3 stops, frame= Plus3 at 2334351..2335400 in the same strand
misc_feature no id HMMPfam hit to PF00665, Integrase core domain, score 1.9e-41 intersects 100%(160aa) with IS3_IS150_ORF2 e-value= 5e-121 complete sequence hit coverage= 100%, length is 350aa with 42 gaps, 3 stops, frame= Plus3 at 2334351..2335400 in the same strand
misc_feature no id Integrase core domain; Region: rve; cl01316 intersects 100%(118aa) with IS3_IS150_ORF2 e-value= 5e-121 complete sequence hit coverage= 100%, length is 350aa with 42 gaps, 3 stops, frame= Plus3 at 2334351..2335400 in the same strand
CDS YP_001972086.1 putative ISPsy9 like transposase intersects 39.85%(69aa) with IS3_IS407_ORF2 e-value= 9.5e-09 complete sequence hit coverage= 100%, length is 352aa with 19 gaps, 3 stops, frame= Plus3 at 2334363..2335418 in the same strand
CDS YP_001972087.1 putative ISPsy9 like transposase for insertion sequence intersects 100%(278aa) with IS3_IS407_ORF2 e-value= 9.5e-09 complete sequence hit coverage= 100%, length is 352aa with 19 gaps, 3 stops, frame= Plus3 at 2334363..2335418 in the same strand
misc_feature no id putative transposase OrfB; Reviewed; Region: PHA02517 intersects 100%(251aa) with IS3_IS407_ORF2 e-value= 9.5e-09 complete sequence hit coverage= 100%, length is 352aa with 19 gaps, 3 stops, frame= Plus3 at 2334363..2335418 in the same strand
misc_feature no id HMMPfam hit to PF00665, Integrase core domain, score 1.9e-41 intersects 100%(160aa) with IS3_IS407_ORF2 e-value= 9.5e-09 complete sequence hit coverage= 100%, length is 352aa with 19 gaps, 3 stops, frame= Plus3 at 2334363..2335418 in the same strand
misc_feature no id Integrase core domain; Region: rve; cl01316 intersects 100%(118aa) with IS3_IS407_ORF2 e-value= 9.5e-09 complete sequence hit coverage= 100%, length is 352aa with 19 gaps, 3 stops, frame= Plus3 at 2334363..2335418 in the same strand
CDS YP_001972086.1 putative ISPsy9 like transposase intersects 15.13%(26aa) with IS3_IS150_ORF1 e-value= 3.2e-87 complete sequence hit coverage= 100%, length is 300aa with 9 gaps, 0 stops, frame= Plus3 at 2334492..2335391 in the same strand
CDS YP_001972087.1 putative ISPsy9 like transposase for insertion sequence intersects 98.92%(275aa) with IS3_IS150_ORF1 e-value= 3.2e-87 complete sequence hit coverage= 100%, length is 300aa with 9 gaps, 0 stops, frame= Plus3 at 2334492..2335391 in the same strand
misc_feature no id putative transposase OrfB; Reviewed; Region: PHA02517 intersects 100%(251aa) with IS3_IS150_ORF1 e-value= 3.2e-87 complete sequence hit coverage= 100%, length is 300aa with 9 gaps, 0 stops, frame= Plus3 at 2334492..2335391 in the same strand
misc_feature no id HMMPfam hit to PF00665, Integrase core domain, score 1.9e-41 intersects 100%(160aa) with IS3_IS150_ORF1 e-value= 3.2e-87 complete sequence hit coverage= 100%, length is 300aa with 9 gaps, 0 stops, frame= Plus3 at 2334492..2335391 in the same strand
misc_feature no id Integrase core domain; Region: rve; cl01316 intersects 100%(118aa) with IS3_IS150_ORF1 e-value= 3.2e-87 complete sequence hit coverage= 100%, length is 300aa with 9 gaps, 0 stops, frame= Plus3 at 2334492..2335391 in the same strand
CDS YP_001972086.1 putative ISPsy9 like transposase intersects 13.41%(23aa) with IS481 e-value= 5e-06 complete sequence hit coverage= 100%, length is 312aa with 46 gaps, 1 stops, frame= Plus3 at 2334501..2335436 in the same strand
CDS YP_001972087.1 putative ISPsy9 like transposase for insertion sequence intersects 100%(278aa) with IS481 e-value= 5e-06 complete sequence hit coverage= 100%, length is 312aa with 46 gaps, 1 stops, frame= Plus3 at 2334501..2335436 in the same strand
misc_feature no id putative transposase OrfB; Reviewed; Region: PHA02517 intersects 100%(251aa) with IS481 e-value= 5e-06 complete sequence hit coverage= 100%, length is 312aa with 46 gaps, 1 stops, frame= Plus3 at 2334501..2335436 in the same strand
misc_feature no id HMMPfam hit to PF00665, Integrase core domain, score 1.9e-41 intersects 100%(160aa) with IS481 e-value= 5e-06 complete sequence hit coverage= 100%, length is 312aa with 46 gaps, 1 stops, frame= Plus3 at 2334501..2335436 in the same strand

misc_feature no id Integrase core domain; Region: rve; cl01316 intersects 100%(118aa) with IS481 e-value= 5e-06 complete sequence hit coverage= 100%, length is 312aa with 46 gaps, 1 stops, frame= Plus3 at 2334501..2335436 in the same strand
repeat_region no id no product intersects 9.09%(2aa) with IS481 e-value= 5e-06 complete sequence hit coverage= 100%, length is 312aa with 46 gaps, 1 stops, frame= Plus3 at 2334501..2335436 in the same strand
CDS YP_001972086.1 putative ISPsy9 like transposase intersects 5.94%(10aa) with IS3_IS150_ORF2 e-value= 1.9e-151 fragment hit coverage= 76.61%, length is 287aa with 14 gaps, 1 stops, frame= Plus3 at 2334540..2335400 in the same strand
CDS YP_001972087.1 putative ISPsy9 like transposase for insertion sequence intersects 100%(278aa) with IS3_IS150_ORF2 e-value= 1.9e-151 fragment hit coverage= 76.61%, length is 287aa with 14 gaps, 1 stops, frame= Plus3 at 2334540..2335400 in the same strand
misc_feature no id putative transposase OrfB; Reviewed; Region: PHA02517 intersects 100%(251aa) with IS3_IS150_ORF2 e-value= 1.9e-151 fragment hit coverage= 76.61%, length is 287aa with 14 gaps, 1 stops, frame= Plus3 at 2334540..2335400 in the same strand
misc_feature no id HMMPfam hit to PF00665, Integrase core domain, score 1.9e-41 intersects 100%(160aa) with IS3_IS150_ORF2 e-value= 1.9e-151 fragment hit coverage= 76.61%, length is 287aa with 14 gaps, 1 stops, frame= Plus3 at 2334540..2335400 in the same strand
misc_feature no id Integrase core domain; Region: rve; cl01316 intersects 100%(118aa) with IS3_IS150_ORF2 e-value= 1.9e-151 fragment hit coverage= 76.61%, length is 287aa with 14 gaps, 1 stops, frame= Plus3 at 2334540..2335400 in the same strand
CDS YP_001972086.1 putative ISPsy9 like transposase intersects 4.21%(7aa) with IS3_IS51_ORF1e-value= 8.5e-39 complete sequence hit coverage= 100%, length is 291aa with 18 gaps, 1 stops, frame= Plus3 at 2334549..2335421 in the same strand
CDS YP_001972087.1 putative ISPsy9 like transposase for insertion sequence intersects 100%(278aa) with IS3_IS51_ORF1 e-value= 8.5e-39 complete sequence hit coverage= 100%, length is 291aa with 18 gaps, 1 stops, frame= Plus3 at 2334549..2335421 in the same strand
misc_feature no id putative transposase OrfB; Reviewed; Region: PHA02517 intersects 100%(251aa) with IS3_IS51_ORF1 e-value= 8.5e-39 complete sequence hit coverage= 100%, length is 291aa with 18 gaps, 1 stops, frame= Plus3 at 2334549..2335421 in the same strand
misc_feature no id HMMPfam hit to PF00665, Integrase core domain, score 1.9e-41 intersects 100%(160aa) with IS3_IS51_ORF1 e-value= 8.5e-39 complete sequence hit coverage= 100%, length is 291aa with 18 gaps, 1 stops, frame= Plus3 at 2334549..2335421 in the same strand
misc_feature no id Integrase core domain; Region: rve; cl01316 intersects 100%(118aa) with IS3_IS51_ORF1 e-value= 8.5e-39 complete sequence hit coverage= 100%, length is 291aa with 18 gaps, 1 stops, frame= Plus3 at 2334549..2335421 in the same strand
CDS YP_001972086.1 putative ISPsy9 like transposase intersects 4.21%(7aa) with IS3_IS51_ORF2e-value= 1.2e-17 complete sequence hit coverage= 100%, length is 292aa with 25 gaps, 1 stops, frame= Plus3 at 2334549..2335424 in the same strand
CDS YP_001972087.1 putative ISPsy9 like transposase for insertion sequence intersects 100%(278aa) with IS3_IS51_ORF2 e-value= 1.2e-17 complete sequence hit coverage= 100%, length is 292aa with 25 gaps, 1 stops, frame= Plus3 at 2334549..2335424 in the same strand
misc_feature no id putative transposase OrfB; Reviewed; Region: PHA02517 intersects 100%(251aa) with IS3_IS51_ORF2 e-value= 1.2e-17 complete sequence hit coverage= 100%, length is 292aa with 25 gaps, 1 stops, frame= Plus3 at 2334549..2335424 in the same strand
misc_feature no id HMMPfam hit to PF00665, Integrase core domain, score 1.9e-41 intersects 100%(160aa) with IS3_IS51_ORF2 e-value= 1.2e-17 complete sequence hit coverage= 100%, length is 292aa with 25 gaps, 1 stops, frame= Plus3 at 2334549..2335424 in the same strand
misc_feature no id Integrase core domain; Region: rve; cl01316 intersects 100%(118aa) with IS3_IS51_ORF2 e-value= 1.2e-17 complete sequence hit coverage= 100%, length is 292aa with 25 gaps, 1 stops, frame= Plus3 at 2334549..2335424 in the same strand
CDS YP_001972086.1 putative ISPsy9 like transposase intersects 3.64%(6aa) with IS3_IS3_ORF1 e-value= 4.6e-75 complete sequence hit coverage= 100%, length is 279aa with 8 gaps, 0 stops, frame= Plus3 at 2334552..2335388 in the same strand
CDS YP_001972087.1 putative ISPsy9 like transposase for insertion sequence intersects 98.56%(274aa) with IS3_IS3_ORF1 e-value= 4.6e-75 complete sequence hit coverage= 100%, length is 279aa with 8 gaps, 0 stops, frame= Plus3 at 2334552..2335388 in the same strand

misc_feature	no id	putative transposase OrfB; Reviewed; Region: PHA02517 intersects 100%(251aa) with IS3_IS3_ORF1	e-value= 4.6e-75 complete sequence hit coverage= 100%, length is 279aa with 8 gaps, 0 stops, frame= Plus3 at 2334552..2335388 in the same strand
misc_feature	no id	HMMPfam hit to PF00665, Integrase core domain, score 1.9e-41 intersects 100%(160aa) with IS3_IS3_ORF1	e-value= 4.6e-75 complete sequence hit coverage= 100%, length is 279aa with 8 gaps, 0 stops, frame= Plus3 at 2334552..2335388 in the same strand
misc_feature	no id	Integrase core domain; Region: rve; cl01316 intersects 100%(118aa) with IS3_IS3_ORF1	e-value= 4.6e-75 complete sequence hit coverage= 100%, length is 279aa with 8 gaps, 0 stops, frame= Plus3 at 2334552..2335388 in the same strand
CDS	YP_001972086.1		putative ISPsy9 like transposase intersects 1.34%(2aa) with IS3_IS3_ORF2	e-value= 3.3e-64 complete sequence hit coverage= 100%, length is 279aa with 9 gaps, 1 stops, frame= Plus3 at 2334564..2335400 in the same strand
CDS	YP_001972087.1		putative ISPsy9 like transposase for insertion sequence intersects 100%(278aa) with IS3_IS3_ORF2	e-value= 3.3e-64 complete sequence hit coverage= 100%, length is 279aa with 9 gaps, 1 stops, frame= Plus3 at 2334564..2335400 in the same strand
misc_feature	no id	putative transposase OrfB; Reviewed; Region: PHA02517 intersects 100%(251aa) with IS3_IS3_ORF2	e-value= 3.3e-64 complete sequence hit coverage= 100%, length is 279aa with 9 gaps, 1 stops, frame= Plus3 at 2334564..2335400 in the same strand
misc_feature	no id	HMMPfam hit to PF00665, Integrase core domain, score 1.9e-41 intersects 100%(160aa) with IS3_IS3_ORF2	e-value= 3.3e-64 complete sequence hit coverage= 100%, length is 279aa with 9 gaps, 1 stops, frame= Plus3 at 2334564..2335400 in the same strand
misc_feature	no id	Integrase core domain; Region: rve; cl01316 intersects 100%(118aa) with IS3_IS3_ORF2	e-value= 3.3e-64 complete sequence hit coverage= 100%, length is 279aa with 9 gaps, 1 stops, frame= Plus3 at 2334564..2335400 in the same strand
CDS	YP_001972087.1		putative ISPsy9 like transposase for insertion sequence intersects 84.17%(234aa) with IS3_IS51_ORF1	e-value= 5e-45 fragment hit coverage= 79.80%, length is 234aa with 12 gaps, 0 stops, frame= Plus3 at 2334672..2335373 in the same strand
misc_feature	no id	putative transposase OrfB; Reviewed; Region: PHA02517 intersects 92.83%(233aa) with IS3_IS51_ORF1	e-value= 5e-45 fragment hit coverage= 79.80%, length is 234aa with 12 gaps, 0 stops, frame= Plus3 at 2334672..2335373 in the same strand
misc_feature	no id	HMMPfam hit to PF00665, Integrase core domain, score 1.9e-41 intersects 98.75%(158aa) with IS3_IS51_ORF1	e-value= 5e-45 fragment hit coverage= 79.80%, length is 234aa with 12 gaps, 0 stops, frame= Plus3 at 2334672..2335373 in the same strand
misc_feature	no id	Integrase core domain; Region: rve; cl01316 intersects 100%(118aa) with IS3_IS51_ORF1	e-value= 5e-45 fragment hit coverage= 79.80%, length is 234aa with 12 gaps, 0 stops, frame= Plus3 at 2334672..2335373 in the same strand
CDS	YP_001972087.1		putative ISPsy9 like transposase for insertion sequence intersects 63.31%(176aa) with IS3_IS51_ORF2	e-value= 7.8e-32 fragment hit coverage= 57.41%, length is 176aa with 6 gaps, 0 stops, frame= Plus3 at 2334723..2335250 in the same strand
misc_feature	no id	putative transposase OrfB; Reviewed; Region: PHA02517 intersects 70.12%(176aa) with IS3_IS51_ORF2	e-value= 7.8e-32 fragment hit coverage= 57.41%, length is 176aa with 6 gaps, 0 stops, frame= Plus3 at 2334723..2335250 in the same strand
misc_feature	no id	HMMPfam hit to PF00665, Integrase core domain, score 1.9e-41 intersects 73.12%(117aa) with IS3_IS51_ORF2	e-value= 7.8e-32 fragment hit coverage= 57.41%, length is 176aa with 6 gaps, 0 stops, frame= Plus3 at 2334723..2335250 in the same strand
misc_feature	no id	Integrase core domain; Region: rve; cl01316 intersects 99.15%(117aa) with IS3_IS51_ORF2	e-value= 7.8e-32 fragment hit coverage= 57.41%, length is 176aa with 6 gaps, 0 stops, frame= Plus3 at 2334723..2335250 in the same strand
CDS	YP_001972087.1		putative ISPsy9 like transposase for insertion sequence intersects 12.95%(36aa) with IS481	e-value= 3.8e-07 fragment hit coverage= 11.11%, length is 36aa with 3 gaps, 0 stops, frame= Plus3 at 2335227..2335334 in the same strand
misc_feature	no id	putative transposase OrfB; Reviewed; Region: PHA02517 intersects 14.34%(36aa) with IS481	e-value= 3.8e-07 fragment hit coverage= 11.11%, length is 36aa with 3 gaps, 0 stops, frame= Plus3 at 2335227..2335334 in the same strand
misc_feature	no id	HMMPfam hit to PF00665, Integrase core domain, score 1.9e-41 intersects 22.50%(36aa) with IS481	e-value= 3.8e-07 fragment hit coverage= 11.11%, length is 36aa with 3 gaps, 0 stops, frame= Plus3 at 2335227..2335334 in the same strand

misc_feature no id Integrase core domain; Region: rve; cl01316 intersects 7.63%(9aa) with IS481 e-value= 3.8e-07 fragment hit coverage= 11.11%, length is 36aa with 3 gaps, 0 stops, frame= Plus3 at 2335227..2335334 in the same strand
Class A

26.
repeat_region no id no product intersects 80.76%(296aa) with IS110 e-value= 1.7e-49 complete sequence hit coverage= 100%, length is 313aa with 108 gaps, 1 stops, frame= Plus2 at 2371592..2372530 in the opposite strand
CDS YP_001972121.1 putative transposase intersects 99.67%(304aa) with IS110 e-value= 1.7e-49 complete sequence hit coverage= 100%, length is 313aa with 108 gaps, 1 stops, frame= Plus2 at 2371592..2372530 in the same strand
misc_feature no id Transposase and inactivated derivatives [DNA replication, recombination, and repair]; Region: COG3547 intersects 100%(290aa) with IS110 e-value= 1.7e-49 complete sequence hit coverage= 100%, length is 313aa with 108 gaps, 1 stops, frame= Plus2 at 2371592..2372530 in the same strand
misc_feature no id Transposase; Region: Transposase_9; pfam01548 intersects 100%(85aa) with IS110 e-value= 1.7e-49 complete sequence hit coverage= 100%, length is 313aa with 108 gaps, 1 stops, frame= Plus2 at 2371592..2372530 in the same strand
misc_feature no id HMMPfam hit to PF01548, Transposase, score 2.6e-10 intersects 100%(99aa) with IS110 e-value= 1.7e-49 complete sequence hit coverage= 100%, length is 313aa with 108 gaps, 1 stops, frame= Plus2 at 2371592..2372530 in the same strand
misc_feature no id HMMPfam hit to PF02371, Transposase IS116/IS110/IS902 family, score 2e-24 intersects 100%(108aa) with IS110 e-value= 1.7e-49 complete sequence hit coverage= 100%, length is 313aa with 108 gaps, 1 stops, frame= Plus2 at 2371592..2372530 in the same strand
misc_feature no id Transposase IS116/IS110/IS902 family; Region: Transposase_20; pfam02371 intersects 100%(79aa) with IS110 e-value= 1.7e-49 complete sequence hit coverage= 100%, length is 313aa with 108 gaps, 1 stops, frame= Plus2 at 2371592..2372530 in the same strand
CDS YP_001972122.1 hypothetical protein intersects 5.18%(5aa) with IS110 e-value= 1.7e-49 complete sequence hit coverage= 100%, length is 313aa with 108 gaps, 1 stops, frame= Plus2 at 2371592..2372530 in the opposite strand
repeat_region no id no product intersects 79.49%(292aa) with IS110 e-value= 1.4e-51 fragment hit coverage= 96.15%, length is 292aa with 108 gaps, 0 stops, frame= Plus2 at 2371592..2372467 in the opposite strand
CDS YP_001972121.1 putative transposase intersects 95.74%(292aa) with IS110 e-value= 1.4e-51 fragment hit coverage= 96.15%, length is 292aa with 108 gaps, 0 stops, frame= Plus2 at 2371592..2372467 in the same strand
misc_feature no id Transposase and inactivated derivatives [DNA replication, recombination, and repair]; Region: COG3547 intersects 100%(290aa) with IS110 e-value= 1.4e-51 fragment hit coverage= 96.15%, length is 292aa with 108 gaps, 0 stops, frame= Plus2 at 2371592..2372467 in the same strand
misc_feature no id Transposase; Region: Transposase_9; pfam01548 intersects 100%(85aa) with IS110 e-value= 1.4e-51 fragment hit coverage= 96.15%, length is 292aa with 108 gaps, 0 stops, frame= Plus2 at 2371592..2372467 in the same strand
misc_feature no id HMMPfam hit to PF01548, Transposase, score 2.6e-10 intersects 100%(99aa) with IS110 e-value= 1.4e-51 fragment hit coverage= 96.15%, length is 292aa with 108 gaps, 0 stops, frame= Plus2 at 2371592..2372467 in the same strand
misc_feature no id HMMPfam hit to PF02371, Transposase IS116/IS110/IS902 family, score 2e-24 intersects 100%(108aa) with IS110 e-value= 1.4e-51 fragment hit coverage= 96.15%, length is 292aa with 108 gaps, 0 stops, frame= Plus2 at 2371592..2372467 in the same strand
misc_feature no id Transposase IS116/IS110/IS902 family; Region: Transposase_20; pfam02371 intersects 100%(79aa) with IS110 e-value= 1.4e-51 fragment hit coverage= 96.15%, length is 292aa with 108 gaps, 0 stops, frame= Plus2 at 2371592..2372467 in the same strand
Class A

27.
CDS YP_001972142.1 hypothetical protein intersects 4.46%(6aa) with IS110 e-value= 6.5e-50 complete sequence hit coverage= 100%, length is 313aa with 108 gaps, 2 stops, frame= Minus1 at 2394142..2395079 in the same strand

CDS YP_001972143.1 putative transposase intersects 99.56%(303aa) with IS110 e-value= 6.5e-50 complete sequence hit coverage= 100%, length is 313aa with 108 gaps, 2 stops, frame= Minus1 at 2394142..2395079 in the same strand
repeat_region no id no product intersects 80.67%(296aa) with IS110 e-value= 6.5e-50 complete sequence hit coverage= 100%, length is 313aa with 108 gaps, 2 stops, frame= Minus1 at 2394142..2395079 in the opposite strand
misc_feature no id Transposase and inactivated derivatives [DNA replication, recombination, and repair]; Region: COG3547 intersects 100%(290aa) with IS110 e-value= 6.5e-50 complete sequence hit coverage= 100%, length is 313aa with 108 gaps, 2 stops, frame= Minus1 at 2394142..2395079 in the same strand
misc_feature no id Transposase IS116/IS110/IS902 family; Region: Transposase_20; pfam02371 intersects 100%(79aa) with IS110 e-value= 6.5e-50 complete sequence hit coverage= 100%, length is 313aa with 108 gaps, 2 stops, frame= Minus1 at 2394142..2395079 in the same strand
misc_feature no id HMMPfam hit to PF02371, Transposase IS116/IS110/IS902 family, score 2e-24 intersects 100%(108aa) with IS110 e-value= 6.5e-50 complete sequence hit coverage= 100%, length is 313aa with 108 gaps, 2 stops, frame= Minus1 at 2394142..2395079 in the same strand
misc_feature no id HMMPfam hit to PF01548, Transposase, score 2.6e-10 intersects 100%(99aa) with IS110 e-value= 6.5e-50 complete sequence hit coverage= 100%, length is 313aa with 108 gaps, 2 stops, frame= Minus1 at 2394142..2395079 in the same strand
misc_feature no id Transposase; Region: Transposase_9; pfam01548 intersects 100%(85aa) with IS110 e-value= 6.5e-50 complete sequence hit coverage= 100%, length is 313aa with 108 gaps, 2 stops, frame= Minus1 at 2394142..2395079 in the same strand
CDS YP_001972143.1 putative transposase intersects 95.63%(291aa) with IS110 e-value= 1.4e-51 fragment hit coverage= 96.15%, length is 292aa with 108 gaps, 0 stops, frame= Minus1 at 2394205..2395079 in the same strand
repeat_region no id no product intersects 79.40%(291aa) with IS110 e-value= 1.4e-51 fragment hit coverage= 96.15%, length is 292aa with 108 gaps, 0 stops, frame= Minus1 at 2394205..2395079 in the opposite strand
misc_feature no id Transposase and inactivated derivatives [DNA replication, recombination, and repair]; Region: COG3547 intersects 100%(290aa) with IS110 e-value= 1.4e-51 fragment hit coverage= 96.15%, length is 292aa with 108 gaps, 0 stops, frame= Minus1 at 2394205..2395079 in the same strand
misc_feature no id Transposase IS116/IS110/IS902 family; Region: Transposase_20; pfam02371 intersects 100%(79aa) with IS110 e-value= 1.4e-51 fragment hit coverage= 96.15%, length is 292aa with 108 gaps, 0 stops, frame= Minus1 at 2394205..2395079 in the same strand
misc_feature no id HMMPfam hit to PF02371, Transposase IS116/IS110/IS902 family, score 2e-24 intersects 100%(108aa) with IS110 e-value= 1.4e-51 fragment hit coverage= 96.15%, length is 292aa with 108 gaps, 0 stops, frame= Minus1 at 2394205..2395079 in the same strand
misc_feature no id HMMPfam hit to PF01548, Transposase, score 2.6e-10 intersects 100%(99aa) with IS110 e-value= 1.4e-51 fragment hit coverage= 96.15%, length is 292aa with 108 gaps, 0 stops, frame= Minus1 at 2394205..2395079 in the same strand
misc_feature no id Transposase; Region: Transposase_9; pfam01548 intersects 100%(85aa) with IS110 e-value= 1.4e-51 fragment hit coverage= 96.15%, length is 292aa with 108 gaps, 0 stops, frame= Minus1 at 2394205..2395079 in the same strand
Class A

28.
CDS YP_001972151.1 putative transposase intersects 98.31%(309aa) with IS481 e-value= 1.1e-95 complete sequence hit coverage= 100%, length is 314aa with 38 gaps, 1 stops, frame= Minus3 at 2402087..2403027 in the same strand
misc_feature no id HMMPfam hit to PF00665, Integrase core domain, score 4.9e-39 intersects 100%(178aa) with IS481 e-value= 1.1e-95 complete sequence hit coverage= 100%, length is 314aa with 38 gaps, 1 stops, frame= Minus3 at 2402087..2403027 in the same strand
misc_feature no id Transposase and inactivated derivatives [DNA replication, recombination, and repair]; Region: Tra5; COG2801 intersects 100%(227aa) with IS481 e-value= 1.1e-95 complete sequence hit coverage= 100%, length is 314aa with 38 gaps, 1 stops, frame= Minus3 at 2402087..2403027 in the same strand
misc_feature no id Integrase core domain; Region: rve; cl01316 intersects 100%(134aa) with IS481 e-value= 1.1e-95 complete sequence hit coverage= 100%, length is 314aa with 38 gaps, 1 stops, frame= Minus3 at 2402087..2403027 in the same strand

misc_feature no id Transposase and inactivated derivatives [DNA replication, recombination, and repair]; Region: COG3415 intersects 100%(100aa) with IS481 e-value= 1.1e-95 complete sequence hit coverage= 100%, length is 314aa with 38 gaps, 1 stops, frame= Minus3 at 2402087..2403027 in the same strand
CDS YP_001972151.1 putative transposase intersects 28.15%(88aa) with IS3_IS150_ORF2 e-value= 1.8e-10 fragment hit coverage= 23.39%, length is 89aa with 2 gaps, 0 stops, frame= Minus3 at 2402141..2402406 in the same strand
misc_feature no id HMMPfam hit to PF00665, Integrase core domain, score 4.9e-39 intersects 49.81%(88aa) with IS3_IS150_ORF2 e-value= 1.8e-10 fragment hit coverage= 23.39%, length is 89aa with 2 gaps, 0 stops, frame= Minus3 at 2402141..2402406 in the same strand
misc_feature no id Transposase and inactivated derivatives [DNA replication, recombination, and repair]; Region: Tra5; COG2801 intersects 39.06%(88aa) with IS3_IS150_ORF2 e-value= 1.8e-10 fragment hit coverage= 23.39%, length is 89aa with 2 gaps, 0 stops, frame= Minus3 at 2402141..2402406 in the same strand
misc_feature no id Integrase core domain; Region: rve; cl01316 intersects 37.81%(50aa) with IS3_IS150_ORF2 e-value= 1.8e-10 fragment hit coverage= 23.39%, length is 89aa with 2 gaps, 0 stops, frame= Minus3 at 2402141..2402406 in the same strand
CDS YP_001972151.1 putative transposase intersects 7.20%(22aa) with IS3_IS407_ORF2 e-value= 4.1e-07 fragment hit coverage= 6.69%, length is 23aa with 0 gaps, 0 stops, frame= Minus3 at 2402144..2402211 in the same strand
misc_feature no id HMMPfam hit to PF00665, Integrase core domain, score 4.9e-39 intersects 12.73%(22aa) with IS3_IS407_ORF2 e-value= 4.1e-07 fragment hit coverage= 6.69%, length is 23aa with 0 gaps, 0 stops, frame= Minus3 at 2402144..2402211 in the same strand
misc_feature no id Transposase and inactivated derivatives [DNA replication, recombination, and repair]; Region: Tra5; COG2801 intersects 9.99%(22aa) with IS3_IS407_ORF2 e-value= 4.1e-07 fragment hit coverage= 6.69%, length is 23aa with 0 gaps, 0 stops, frame= Minus3 at 2402144..2402211 in the same strand
Class A

29.
CDS YP_001972195.1 putative transposase intersects 100%(284aa) with IS3_IS51_ORF2 e-value= 9.2e-135 complete sequence hit coverage= 100%, length is 301aa with 16 gaps, 1 stops, frame= Minus1 at 2442025..2442926 in the same strand
misc_feature no id putative transposase OrfB; Reviewed; Region: PHA02517 intersects 100%(262aa) with IS3_IS51_ORF2 e-value= 9.2e-135 complete sequence hit coverage= 100%, length is 301aa with 16 gaps, 1 stops, frame= Minus1 at 2442025..2442926 in the same strand
misc_feature no id HMMPfam hit to PF00665, Integrase core domain, score 6.6e-51 intersects 100%(161aa) with IS3_IS51_ORF2 e-value= 9.2e-135 complete sequence hit coverage= 100%, length is 301aa with 16 gaps, 1 stops, frame= Minus1 at 2442025..2442926 in the same strand
misc_feature no id Integrase core domain; Region: rve; cl01316 intersects 100%(116aa) with IS3_IS51_ORF2 e-value= 9.2e-135 complete sequence hit coverage= 100%, length is 301aa with 16 gaps, 1 stops, frame= Minus1 at 2442025..2442926 in the same strand
CDS YP_001972195.1 putative transposase intersects 100%(284aa) with IS3_IS51_ORF1 e-value= 1.3e-108 complete sequence hit coverage= 100%, length is 291aa with 16 gaps, 1 stops, frame= Minus1 at 2442037..2442908 in the same strand
misc_feature no id putative transposase OrfB; Reviewed; Region: PHA02517 intersects 100%(262aa) with IS3_IS51_ORF1 e-value= 1.3e-108 complete sequence hit coverage= 100%, length is 291aa with 16 gaps, 1 stops, frame= Minus1 at 2442037..2442908 in the same strand
misc_feature no id HMMPfam hit to PF00665, Integrase core domain, score 6.6e-51 intersects 100%(161aa) with IS3_IS51_ORF1 e-value= 1.3e-108 complete sequence hit coverage= 100%, length is 291aa with 16 gaps, 1 stops, frame= Minus1 at 2442037..2442908 in the same strand
misc_feature no id Integrase core domain; Region: rve; cl01316 intersects 100%(116aa) with IS3_IS51_ORF1 e-value= 1.3e-108 complete sequence hit coverage= 100%, length is 291aa with 16 gaps, 1 stops, frame= Minus1 at 2442037..2442908 in the same strand
CDS YP_001972195.1 putative transposase intersects 95.66%(271aa) with IS3_IS51_ORF2 e-value= 7.1e-151 fragment hit coverage= 90.22%, length is 272aa with 14 gaps, 0 stops, frame= Minus1 at 2442052..2442866 in the same strand
misc_feature no id putative transposase OrfB; Reviewed; Region: PHA02517 intersects 100%(262aa) with IS3_IS51_ORF2 e-value= 7.1e-151 fragment hit coverage= 90.22%, length is 272aa with 14 gaps, 0 stops, frame= Minus1 at 2442052..2442866 in the same strand

misc_feature no id HMMPfam hit to PF00665, Integrase core domain, score 6.6e-51 intersects 100%(161aa) with IS3_IS51_ORF2 e-value= 7.1e-151 fragment hit coverage= 90.22%, length is 272aa with 14 gaps, 0 stops, frame= Minus1 at 2442052..2442866 in the same strand
misc_feature no id Integrase core domain; Region: rve; cl01316 intersects 100%(116aa) with IS3_IS51_ORF2 e-value= 7.1e-151 fragment hit coverage= 90.22%, length is 272aa with 14 gaps, 0 stops, frame= Minus1 at 2442052..2442866 in the same strand
CDS YP_001972195.1 putative transposase intersects 98.83%(280aa) with IS3_IS3_ORF2 e-value= 7.8e-108 complete sequence hit coverage= 100%, length is 281aa with 8 gaps, 0 stops, frame= Minus1 at 2442052..2442893 in the same strand
misc_feature no id putative transposase OrfB; Reviewed; Region: PHA02517 intersects 100%(262aa) with IS3_IS3_ORF2 e-value= 7.8e-108 complete sequence hit coverage= 100%, length is 281aa with 8 gaps, 0 stops, frame= Minus1 at 2442052..2442893 in the same strand
misc_feature no id HMMPfam hit to PF00665, Integrase core domain, score 6.6e-51 intersects 100%(161aa) with IS3_IS3_ORF2 e-value= 7.8e-108 complete sequence hit coverage= 100%, length is 281aa with 8 gaps, 0 stops, frame= Minus1 at 2442052..2442893 in the same strand
misc_feature no id Integrase core domain; Region: rve; cl01316 intersects 100%(116aa) with IS3_IS3_ORF2 e-value= 7.8e-108 complete sequence hit coverage= 100%, length is 281aa with 8 gaps, 0 stops, frame= Minus1 at 2442052..2442893 in the same strand
CDS YP_001972195.1 putative transposase intersects 96.71%(274aa) with IS3_IS150_ORF2 e-value= 3.9e-68 fragment hit coverage= 73.78%, length is 275aa with 15 gaps, 0 stops, frame= Minus1 at 2442061..2442884 in the same strand
misc_feature no id putative transposase OrfB; Reviewed; Region: PHA02517 intersects 100%(262aa) with IS3_IS150_ORF2 e-value= 3.9e-68 fragment hit coverage= 73.78%, length is 275aa with 15 gaps, 0 stops, frame= Minus1 at 2442061..2442884 in the same strand
misc_feature no id HMMPfam hit to PF00665, Integrase core domain, score 6.6e-51 intersects 100%(161aa) with IS3_IS150_ORF2 e-value= 3.9e-68 fragment hit coverage= 73.78%, length is 275aa with 15 gaps, 0 stops, frame= Minus1 at 2442061..2442884 in the same strand
misc_feature no id Integrase core domain; Region: rve; cl01316 intersects 100%(116aa) with IS3_IS150_ORF2 e-value= 3.9e-68 fragment hit coverage= 73.78%, length is 275aa with 15 gaps, 0 stops, frame= Minus1 at 2442061..2442884 in the same strand
CDS YP_001972195.1 putative transposase intersects 98.59%(280aa) with IS3_IS150_ORF2 e-value= 1.2e-42 complete sequence hit coverage= 100%, length is 378aa with 23 gaps, 2 stops, frame= Minus1 at 2442061..2443193 in the same strand
misc_feature no id putative transposase OrfB; Reviewed; Region: PHA02517 intersects 100%(262aa) with IS3_IS150_ORF2 e-value= 1.2e-42 complete sequence hit coverage= 100%, length is 378aa with 23 gaps, 2 stops, frame= Minus1 at 2442061..2443193 in the same strand
misc_feature no id HMMPfam hit to PF00665, Integrase core domain, score 6.6e-51 intersects 100%(161aa) with IS3_IS150_ORF2 e-value= 1.2e-42 complete sequence hit coverage= 100%, length is 378aa with 23 gaps, 2 stops, frame= Minus1 at 2442061..2443193 in the same strand
misc_feature no id Integrase core domain; Region: rve; cl01316 intersects 100%(116aa) with IS3_IS150_ORF2 e-value= 1.2e-42 complete sequence hit coverage= 100%, length is 378aa with 23 gaps, 2 stops, frame= Minus1 at 2442061..2443193 in the same strand
CDS YP_001972196.1 putative MerR-family transcriptional regulator intersects 3.92%(5aa) with IS3_IS150_ORF2 e-value= 1.2e-42 complete sequence hit coverage= 100%, length is 378aa with 23 gaps, 2 stops, frame= Minus1 at 2442061..2443193 in the same strand
misc_feature no id putative transcriptional regulator MerR; Provisional; Region: PRK13752 intersects 2.49%(3aa) with IS3_IS150_ORF2 e-value= 1.2e-42 complete sequence hit coverage= 100%, length is 378aa with 23 gaps, 2 stops, frame= Minus1 at 2442061..2443193 in the same strand
misc_feature no id Helix-Turn-Helix DNA binding domain of the MerR1 transcription regulator; Region: HTH_MerR1; cd04783 intersects 1.85%(2aa) with IS3_IS150_ORF2 e-value= 1.2e-42 complete sequence hit coverage= 100%, length is 378aa with 23 gaps, 2 stops, frame= Minus1 at 2442061..2443193 in the same strand
CDS YP_001972195.1 putative transposase intersects 98.24%(279aa) with IS3_IS150_ORF1 e-value= 2.9e-46 complete sequence hit coverage= 100%, length is 302aa with 9 gaps, 0 stops, frame= Minus1 at 2442064..2442968 in the same strand
misc_feature no id putative transposase OrfB; Reviewed; Region: PHA02517 intersects 100%(262aa) with IS3_IS150_ORF1 e-value= 2.9e-46 complete sequence hit coverage= 100%, length is 302aa with 9 gaps, 0 stops, frame= Minus1 at 2442064..2442968 in the same strand

misc_feature no id HMMPfam hit to PF00665, Integrase core domain, score 6.6e-51 intersects 100%(161aa) with IS3_IS150_ORF1 e-value= 2.9e-46 complete sequence hit coverage= 100%, length is 302aa with 9 gaps, 0 stops, frame= Minus1 at 2442064..2442968 in the same strand
misc_feature no id Integrase core domain; Region: rve; cl01316 intersects 100%(116aa) with IS3_IS150_ORF1 e-value= 2.9e-46 complete sequence hit coverage= 100%, length is 302aa with 9 gaps, 0 stops, frame= Minus1 at 2442064..2442968 in the same strand
CDS YP_001972195.1 putative transposase intersects 98.24%(279aa) with IS3_IS3_ORF1 e-value= 2.7e-94 complete sequence hit coverage= 100%, length is 281aa with 9 gaps, 0 stops, frame= Minus1 at 2442064..2442905 in the same strand
misc_feature no id putative transposase OrfB; Reviewed; Region: PHA02517 intersects 100%(262aa) with IS3_IS3_ORF1 e-value= 2.7e-94 complete sequence hit coverage= 100%, length is 281aa with 9 gaps, 0 stops, frame= Minus1 at 2442064..2442905 in the same strand
misc_feature no id HMMPfam hit to PF00665, Integrase core domain, score 6.6e-51 intersects 100%(161aa) with IS3_IS3_ORF1 e-value= 2.7e-94 complete sequence hit coverage= 100%, length is 281aa with 9 gaps, 0 stops, frame= Minus1 at 2442064..2442905 in the same strand
misc_feature no id Integrase core domain; Region: rve; cl01316 intersects 100%(116aa) with IS3_IS3_ORF1 e-value= 2.7e-94 complete sequence hit coverage= 100%, length is 281aa with 9 gaps, 0 stops, frame= Minus1 at 2442064..2442905 in the same strand
CDS YP_001972195.1 putative transposase intersects 78.76%(223aa) with IS3_IS150_ORF1 e-value= 2.3e-51 fragment hit coverage= 76.14%, length is 224aa with 9 gaps, 0 stops, frame= Minus1 at 2442064..2442734 in the same strand
misc_feature no id putative transposase OrfB; Reviewed; Region: PHA02517 intersects 84.99%(222aa) with IS3_IS150_ORF1 e-value= 2.3e-51 fragment hit coverage= 76.14%, length is 224aa with 9 gaps, 0 stops, frame= Minus1 at 2442064..2442734 in the same strand
misc_feature no id HMMPfam hit to PF00665, Integrase core domain, score 6.6e-51 intersects 100%(161aa) with IS3_IS150_ORF1 e-value= 2.3e-51 fragment hit coverage= 76.14%, length is 224aa with 9 gaps, 0 stops, frame= Minus1 at 2442064..2442734 in the same strand
misc_feature no id Integrase core domain; Region: rve; cl01316 intersects 100%(116aa) with IS3_IS150_ORF1 e-value= 2.3e-51 fragment hit coverage= 76.14%, length is 224aa with 9 gaps, 0 stops, frame= Minus1 at 2442064..2442734 in the same strand
Class A

30.
CDS YP_001972200.1 putative transposase intersects 88.07%(393aa) with ISL3 e-value= 1.1e-43 complete sequence hit coverage= 100%, length is 394aa with 60 gaps, 0 stops, frame= Minus2 at 2446239..2447419 in the same strand
misc_feature no id Transposase and inactivated derivatives [DNA replication, recombination, and repair]; Region: COG3464 intersects 100%(348aa) with ISL3 e-value= 1.1e-43 complete sequence hit coverage= 100%, length is 394aa with 60 gaps, 0 stops, frame= Minus2 at 2446239..2447419 in the same strand
misc_feature no id HMMPfam hit to PF01610, Transposase, score 1.1e-33 intersects 100%(96aa) with ISL3 e-value= 1.1e-43 complete sequence hit coverage= 100%, length is 394aa with 60 gaps, 0 stops, frame= Minus2 at 2446239..2447419 in the same strand
CDS YP_001972200.1 putative transposase intersects 78%(348aa) with ISL3 e-value= 5.3e-50 fragment hit coverage= 80.49%, length is 349aa with 16 gaps, 0 stops, frame= Minus2 at 2446284..2447329 in the same strand
misc_feature no id Transposase and inactivated derivatives [DNA replication, recombination, and repair]; Region: COG3464 intersects 99.90%(347aa) with ISL3 e-value= 5.3e-50 fragment hit coverage= 80.49%, length is 349aa with 16 gaps, 0 stops, frame= Minus2 at 2446284..2447329 in the same strand
misc_feature no id HMMPfam hit to PF01610, Transposase, score 1.1e-33 intersects 100%(96aa) with ISL3 e-value= 5.3e-50 fragment hit coverage= 80.49%, length is 349aa with 16 gaps, 0 stops, frame= Minus2 at 2446284..2447329 in the same strand
Class A

31.
CDS YP_001972201.1 putative ISXac3 like transposase for insertion sequence element intersects 37.18%(38aa) with IS3_IS407_ORF1 e-value= 7e-06 fragment hit coverage= 45.35%, length is 39aa with 0 gaps, 0 stops, frame= Minus2 at 2447712..2447827 in the same strand

misc_feature	no id	HMMPfam hit to PF01527, Transposase, score 1.6e-22 intersects 46.03%(38aa) with IS3_IS407_ORF1	e-value= 7e-06 fragment hit coverage= 45.35%, length is 39aa with 0 gaps, 0 stops, frame= Minus2 at 2447712..2447827 in the same strand
misc_feature	no id	Helix-turn-helix domain of Hin and related proteins, a family of DNA-binding domains unique to bacteria and represented by the Hin protein of Salmonella. The basic HTH domain is a simple fold comprised of three core helices that form a right-handed...; Region: HTH_Hin_like; cl01116 intersects 50.68%(37aa) with IS3_IS407_ORF1	e-value= 7e-06 fragment hit coverage= 45.35%, length is 39aa with 0 gaps, 0 stops, frame= Minus2 at 2447712..2447827 in the same strand
Class A

32.
CDS	YP_001972202.1	putative insertion sequence protein intersects 92.55%(87aa) with IS3_IS150_ORF1	e-value= 1.8e-12 fragment hit coverage= 35.29%, length is 90aa with 18 gaps, 1 stops, frame= Plus2 at 2448206..2448475 in the same strand
misc_feature	no id	HMMPfam hit to PF01527, Transposase, score 7.8e-25 intersects 100%(78aa) with IS3_IS150_ORF1	e-value= 1.8e-12 fragment hit coverage= 35.29%, length is 90aa with 18 gaps, 1 stops, frame= Plus2 at 2448206..2448475 in the same strand
misc_feature	no id	Helix-turn-helix domain of Hin and related proteins, a family of DNA-binding domains unique to bacteria and represented by the Hin protein of Salmonella. The basic HTH domain is a simple fold comprised of three core helices that form a right-handed...; Region: HTH_Hin_like; cl01116 intersects 100%(70aa) with IS3_IS150_ORF1	e-value= 1.8e-12 fragment hit coverage= 35.29%, length is 90aa with 18 gaps, 1 stops, frame= Plus2 at 2448206..2448475 in the same strand
CDS	YP_001972203.1	putative insertion element hypothetical protein intersects 1.88%(5aa) with IS3_IS150_ORF1	e-value= 1.8e-12 fragment hit coverage= 35.29%, length is 90aa with 18 gaps, 1 stops, frame= Plus2 at 2448206..2448475 in the same strand
CDS	YP_001972202.1	putative insertion sequence protein intersects 91.49%(86aa) with IS3_IS407_ORF1	e-value= 8.1e-47 complete sequence hit coverage= 100%, length is 86aa with 0 gaps, 0 stops, frame= Plus2 at 2448206..2448463 in the same strand
misc_feature	no id	HMMPfam hit to PF01527, Transposase, score 7.8e-25 intersects 100%(78aa) with IS3_IS407_ORF1	e-value= 8.1e-47 complete sequence hit coverage= 100%, length is 86aa with 0 gaps, 0 stops, frame= Plus2 at 2448206..2448463 in the same strand
misc_feature	no id	Helix-turn-helix domain of Hin and related proteins, a family of DNA-binding domains unique to bacteria and represented by the Hin protein of Salmonella. The basic HTH domain is a simple fold comprised of three core helices that form a right-handed...; Region: HTH_Hin_like; cl01116 intersects 100%(70aa) with IS3_IS407_ORF1	e-value= 8.1e-47 complete sequence hit coverage= 100%, length is 86aa with 0 gaps, 0 stops, frame= Plus2 at 2448206..2448463 in the same strand
CDS	YP_001972203.1	putative insertion element hypothetical protein intersects 0.47%(1aa) with IS3_IS407_ORF1	e-value= 8.1e-47 complete sequence hit coverage= 100%, length is 86aa with 0 gaps, 0 stops, frame= Plus2 at 2448206..2448463 in the same strand
CDS	YP_001972202.1	putative insertion sequence protein intersects 66.31%(62aa) with IS3_IS407_ORF2	e-value= 5.4e-203 complete sequence hit coverage= 100%, length is 342aa with 2 gaps, 0 stops, frame= Plus1 at 2448280..2449305 in the same strand
misc_feature	no id	HMMPfam hit to PF01527, Transposase, score 7.8e-25 intersects 68.38%(53aa) with IS3_IS407_ORF2	e-value= 5.4e-203 complete sequence hit coverage= 100%, length is 342aa with 2 gaps, 0 stops, frame= Plus1 at 2448280..2449305 in the same strand
misc_feature	no id	Helix-turn-helix domain of Hin and related proteins, a family of DNA-binding domains unique to bacteria and represented by the Hin protein of Salmonella. The basic HTH domain is a simple fold comprised of three core helices that form a right-handed...; Region: HTH_Hin_like; cl01116 intersects 64.76%(45aa) with IS3_IS407_ORF2	e-value= 5.4e-203 complete sequence hit coverage= 100%, length is 342aa with 2 gaps, 0 stops, frame= Plus1 at 2448280..2449305 in the same strand
CDS	YP_001972203.1	putative insertion element hypothetical protein intersects 99.65%(282aa) with IS3_IS407_ORF2	e-value= 5.4e-203 complete sequence hit coverage= 100%, length is 342aa with 2 gaps, 0 stops, frame= Plus1 at 2448280..2449305 in the same strand
misc_feature	no id	Transposase and inactivated derivatives [DNA replication, recombination, and repair]; Region: Tra5; COG2801 intersects 100%(240aa) with IS3_IS407_ORF2	e-value= 5.4e-203 complete sequence hit coverage= 100%, length is 342aa with 2 gaps, 0 stops, frame= Plus1 at 2448280..2449305 in the same strand

misc_feature no id HMMPfam hit to PF00665, Integrase core domain, score 2.7e-39 intersects 100%(159aa) with IS3_IS407_ORF2 e-value= 5.4e-203 complete sequence hit coverage= 100%, length is 342aa with 2 gaps, 0 stops, frame= Plus1 at 2448280..2449305 in the same strand
misc_feature no id Integrase core domain; Region: rve; cl01316 intersects 100%(117aa) with IS3_IS407_ORF2 e-value= 5.4e-203 complete sequence hit coverage= 100%, length is 342aa with 2 gaps, 0 stops, frame= Plus1 at 2448280..2449305 in the same strand
CDS YP_001972202.1 putative insertion sequence protein intersects 16.31%(15aa) with IS3_IS150_ORF1 e-value= 6.2e-15 complete sequence hit coverage= 100%, length is 286aa with 21 gaps, 0 stops, frame= Plus1 at 2448421..2449278 in the same strand
misc_feature no id HMMPfam hit to PF01527, Transposase, score 7.8e-25 intersects 8.12%(6aa) with IS3_IS150_ORF1 e-value= 6.2e-15 complete sequence hit coverage= 100%, length is 286aa with 21 gaps, 0 stops, frame= Plus1 at 2448421..2449278 in the same strand
CDS YP_001972203.1 putative insertion element hypothetical protein intersects 96.47%(273aa) with IS3_IS150_ORF1 e-value= 6.2e-15 complete sequence hit coverage= 100%, length is 286aa with 21 gaps, 0 stops, frame= Plus1 at 2448421..2449278 in the same strand
misc_feature no id Transposase and inactivated derivatives [DNA replication, recombination, and repair]; Region: Tra5; COG2801 intersects 99.58%(239aa) with IS3_IS150_ORF1 e-value= 6.2e-15 complete sequence hit coverage= 100%, length is 286aa with 21 gaps, 0 stops, frame= Plus1 at 2448421..2449278 in the same strand
misc_feature no id HMMPfam hit to PF00665, Integrase core domain, score 2.7e-39 intersects 100%(159aa) with IS3_IS150_ORF1 e-value= 6.2e-15 complete sequence hit coverage= 100%, length is 286aa with 21 gaps, 0 stops, frame= Plus1 at 2448421..2449278 in the same strand
misc_feature no id Integrase core domain; Region: rve; cl01316 intersects 100%(117aa) with IS3_IS150_ORF1 e-value= 6.2e-15 complete sequence hit coverage= 100%, length is 286aa with 21 gaps, 0 stops, frame= Plus1 at 2448421..2449278 in the same strand
CDS YP_001972202.1 putative insertion sequence protein intersects 2.48%(2aa) with IS3_IS407_ORF1 e-value= 8.5e-17 complete sequence hit coverage= 100%, length is 82aa with 4 gaps, 0 stops, frame= Plus1 at 2448460..2448705 in the same strand
CDS YP_001972203.1 putative insertion element hypothetical protein intersects 28.98%(82aa) with IS3_IS407_ORF1 e-value= 8.5e-17 complete sequence hit coverage= 100%, length is 82aa with 4 gaps, 0 stops, frame= Plus1 at 2448460..2448705 in the same strand
misc_feature no id Transposase and inactivated derivatives [DNA replication, recombination, and repair]; Region: Tra5; COG2801 intersects 20%(48aa) with IS3_IS407_ORF1 e-value= 8.5e-17 complete sequence hit coverage= 100%, length is 82aa with 4 gaps, 0 stops, frame= Plus1 at 2448460..2448705 in the same strand
Class A

33.
CDS YP_001972203.1 putative insertion element hypothetical protein intersects 93.64%(265aa) with IS3_IS3_ORF1 e-value= 8.7e-09 complete sequence hit coverage= 100%, length is 265aa with 24 gaps, 0 stops, frame= Plus1 at 2448481..2449275 in the same strand
misc_feature no id Transposase and inactivated derivatives [DNA replication, recombination, and repair]; Region: Tra5; COG2801 intersects 99.17%(238aa) with IS3_IS3_ORF1 e-value= 8.7e-09 complete sequence hit coverage= 100%, length is 265aa with 24 gaps, 0 stops, frame= Plus1 at 2448481..2449275 in the same strand
misc_feature no id HMMPfam hit to PF00665, Integrase core domain, score 2.7e-39 intersects 100%(159aa) with IS3_IS3_ORF1 e-value= 8.7e-09 complete sequence hit coverage= 100%, length is 265aa with 24 gaps, 0 stops, frame= Plus1 at 2448481..2449275 in the same strand
misc_feature no id Integrase core domain; Region: rve; cl01316 intersects 100%(117aa) with IS3_IS3_ORF1 e-value= 8.7e-09 complete sequence hit coverage= 100%, length is 265aa with 24 gaps, 0 stops, frame= Plus1 at 2448481..2449275 in the same strand
CDS YP_001972203.1 putative insertion element hypothetical protein intersects 91.52%(259aa) with IS3_IS150_ORF1 e-value= 4.2e-17 fragment hit coverage= 91.50%, length is 259aa with 21 gaps, 0 stops, frame= Plus1 at 2448502..2449278 in the same strand
misc_feature no id Transposase and inactivated derivatives [DNA replication, recombination, and repair]; Region: Tra5; COG2801 intersects 99.58%(239aa) with IS3_IS150_ORF1 e-value= 4.2e-17 fragment hit coverage= 91.50%, length is 259aa with 21 gaps, 0 stops, frame= Plus1 at 2448502..2449278 in the same strand
misc_feature no id HMMPfam hit to PF00665, Integrase core domain, score 2.7e-39 intersects 100%(159aa) with IS3_IS150_ORF1 e-value= 4.2e-17 fragment hit coverage= 91.50%, length is 259aa with 21 gaps, 0 stops, frame= Plus1 at 2448502..2449278 in the same strand

misc_feature no id Integrase core domain; Region: rve; cl01316 intersects 100%(117aa) with IS3_IS150_ORF1 e-value= 4.2e-17 fragment hit coverage= 91.50%, length is 259aa with 21 gaps, 0 stops, frame= Plus1 at 2448502..2449278 in the same strand
CDS YP_001972203.1 putative insertion element hypothetical protein intersects 24.03%(68aa) with IS3_IS150_ORF2 e-value= 2.9e-08 fragment hit coverage= 17.99%, length is 68aa with 2 gaps, 0 stops, frame= Plus1 at 2449078..2449281 in the same strand
misc_feature no id Transposase and inactivated derivatives [DNA replication, recombination, and repair]; Region: Tra5; COG2801 intersects 28.33%(68aa) with IS3_IS150_ORF2 e-value= 2.9e-08 fragment hit coverage= 17.99%, length is 68aa with 2 gaps, 0 stops, frame= Plus1 at 2449078..2449281 in the same strand
misc_feature no id HMMPfam hit to PF00665, Integrase core domain, score 2.7e-39 intersects 39.62%(63aa) with IS3_IS150_ORF2 e-value= 2.9e-08 fragment hit coverage= 17.99%, length is 68aa with 2 gaps, 0 stops, frame= Plus1 at 2449078..2449281 in the same strand
misc_feature no id Integrase core domain; Region: rve; cl01316 intersects 17.95%(21aa) with IS3_IS150_ORF2 e-value= 2.9e-08 fragment hit coverage= 17.99%, length is 68aa with 2 gaps, 0 stops, frame= Plus1 at 2449078..2449281 in the same strand
CDS YP_001972203.1 putative insertion element hypothetical protein intersects 21.20%(60aa) with IS3_IS3_ORF2 e-value= 6.9e-07 fragment hit coverage= 21.18%, length is 60aa with 1 gaps, 0 stops, frame= Plus1 at 2449081..2449260 in the same strand
misc_feature no id Transposase and inactivated derivatives [DNA replication, recombination, and repair]; Region: Tra5; COG2801 intersects 25%(60aa) with IS3_IS3_ORF2 e-value= 6.9e-07 fragment hit coverage= 21.18%, length is 60aa with 1 gaps, 0 stops, frame= Plus1 at 2449081..2449260 in the same strand
misc_feature no id HMMPfam hit to PF00665, Integrase core domain, score 2.7e-39 intersects 37.74%(60aa) with IS3_IS3_ORF2 e-value= 6.9e-07 fragment hit coverage= 21.18%, length is 60aa with 1 gaps, 0 stops, frame= Plus1 at 2449081..2449260 in the same strand
misc_feature no id Integrase core domain; Region: rve; cl01316 intersects 17.09%(20aa) with IS3_IS3_ORF2 e-value= 6.9e-07 fragment hit coverage= 21.18%, length is 60aa with 1 gaps, 0 stops, frame= Plus1 at 2449081..2449260 in the same strand
CDS YP_001972203.1 putative insertion element hypothetical protein intersects 21.20%(60aa) with IS3_IS3_ORF1 e-value= 2.9e-08 fragment hit coverage= 22.03%, length is 60aa with 3 gaps, 0 stops, frame= Plus1 at 2449096..2449275 in the same strand
misc_feature no id Transposase and inactivated derivatives [DNA replication, recombination, and repair]; Region: Tra5; COG2801 intersects 25%(60aa) with IS3_IS3_ORF1 e-value= 2.9e-08 fragment hit coverage= 22.03%, length is 60aa with 3 gaps, 0 stops, frame= Plus1 at 2449096..2449275 in the same strand
misc_feature no id HMMPfam hit to PF00665, Integrase core domain, score 2.7e-39 intersects 35.85%(57aa) with IS3_IS3_ORF1 e-value= 2.9e-08 fragment hit coverage= 22.03%, length is 60aa with 3 gaps, 0 stops, frame= Plus1 at 2449096..2449275 in the same strand
misc_feature no id Integrase core domain; Region: rve; cl01316 intersects 12.82%(15aa) with IS3_IS3_ORF1 e-value= 2.9e-08 fragment hit coverage= 22.03%, length is 60aa with 3 gaps, 0 stops, frame= Plus1 at 2449096..2449275 in the same strand
Class A

34.
CDS YP_001972246.1 putative transposon Tn5044/Tn3926 transposase intersects 29.06%(290aa) with Tn3 e-value= 1.5e-20 fragment hit coverage= 29.48%, length is 290aa with 7 gaps, 0 stops, frame= Plus1 at 2494939..2495808 in the same strand
misc_feature no id HMMPfam hit to PF01526, Transposase, score 1.3e-66 intersects 29.74%(290aa) with Tn3 e-value= 1.5e-20 fragment hit coverage= 29.48%, length is 290aa with 7 gaps, 0 stops, frame= Plus1 at 2494939..2495808 in the same strand
misc_feature no id Transposase; Region: Transposase_7; pfam01526 intersects 31.15%(290aa) with Tn3 e-value= 1.5e-20 fragment hit coverage= 29.48%, length is 290aa with 7 gaps, 0 stops, frame= Plus1 at 2494939..2495808 in the same strand
misc_feature no id Transposase and inactivated derivatives, TnpA family [DNA replication, recombination, and repair]; Region: COG4644 intersects 87.34%(276aa) with Tn3 e-value= 1.5e-20 fragment hit coverage= 29.48%, length is 290aa with 7 gaps, 0 stops, frame= Plus1 at 2494939..2495808 in the same strand
Class A

35.

CDS YP_001972257.1 putative transposase (pseudogene) intersects 100%(287aa) with IS3_IS51_ORF2 e-value= 7.4e-58 complete sequence hit coverage= 100%, length is 301aa with 20 gaps, 1 stops, frame= Minus2 at 2507577..2508478 in the same strand
misc_feature no id HMMPfam hit to PF00665, Integrase core domain, score 3.3e-32 intersects 100%(161aa) with IS3_IS51_ORF2 e-value= 7.4e-58 complete sequence hit coverage= 100%, length is 301aa with 20 gaps, 1 stops, frame= Minus2 at 2507577..2508478 in the same strand
misc_feature no id putative transposase OrfB; Reviewed; Region: PHA02517 intersects 100%(250aa) with IS3_IS51_ORF2 e-value= 7.4e-58 complete sequence hit coverage= 100%, length is 301aa with 20 gaps, 1 stops, frame= Minus2 at 2507577..2508478 in the same strand
misc_feature no id Integrase core domain; Region: rve; cl01316 intersects 100%(117aa) with IS3_IS51_ORF2 e-value= 7.4e-58 complete sequence hit coverage= 100%, length is 301aa with 20 gaps, 1 stops, frame= Minus2 at 2507577..2508478 in the same strand
CDS YP_001972258.1 putative ISXac3 like transposase intersects 5.21%(5aa) with IS3_IS51_ORF2 e-value= 7.4e-58 complete sequence hit coverage= 100%, length is 301aa with 20 gaps, 1 stops, frame= Minus2 at 2507577..2508478 in the same strand
CDS YP_001972257.1 putative transposase (pseudogene) intersects 99.30%(285aa) with IS3_IS51_ORF1 e-value= 7.6e-67 complete sequence hit coverage= 100%, length is 287aa with 21 gaps, 0 stops, frame= Minus2 at 2507601..2508460 in the same strand
misc_feature no id HMMPfam hit to PF00665, Integrase core domain, score 3.3e-32 intersects 100%(161aa) with IS3_IS51_ORF1 e-value= 7.6e-67 complete sequence hit coverage= 100%, length is 287aa with 21 gaps, 0 stops, frame= Minus2 at 2507601..2508460 in the same strand
misc_feature no id putative transposase OrfB; Reviewed; Region: PHA02517 intersects 100%(250aa) with IS3_IS51_ORF1 e-value= 7.6e-67 complete sequence hit coverage= 100%, length is 287aa with 21 gaps, 0 stops, frame= Minus2 at 2507601..2508460 in the same strand
misc_feature no id Integrase core domain; Region: rve; cl01316 intersects 100%(117aa) with IS3_IS51_ORF1 e-value= 7.6e-67 complete sequence hit coverage= 100%, length is 287aa with 21 gaps, 0 stops, frame= Minus2 at 2507601..2508460 in the same strand
CDS YP_001972257.1 putative transposase (pseudogene) intersects 97.56%(280aa) with IS3_IS150_ORF1 e-value= 1.5e-33 complete sequence hit coverage= 100%, length is 300aa with 10 gaps, 0 stops, frame= Minus2 at 2507616..2508514 in the same strand
misc_feature no id HMMPfam hit to PF00665, Integrase core domain, score 3.3e-32 intersects 100%(161aa) with IS3_IS150_ORF1 e-value= 1.5e-33 complete sequence hit coverage= 100%, length is 300aa with 10 gaps, 0 stops, frame= Minus2 at 2507616..2508514 in the same strand
misc_feature no id putative transposase OrfB; Reviewed; Region: PHA02517 intersects 100%(250aa) with IS3_IS150_ORF1 e-value= 1.5e-33 complete sequence hit coverage= 100%, length is 300aa with 10 gaps, 0 stops, frame= Minus2 at 2507616..2508514 in the same strand
misc_feature no id Integrase core domain; Region: rve; cl01316 intersects 100%(117aa) with IS3_IS150_ORF1 e-value= 1.5e-33 complete sequence hit coverage= 100%, length is 300aa with 10 gaps, 0 stops, frame= Minus2 at 2507616..2508514 in the same strand
CDS YP_001972258.1 putative ISXac3 like transposase intersects 17.71%(17aa) with IS3_IS150_ORF1 e-value= 1.5e-33 complete sequence hit coverage= 100%, length is 300aa with 10 gaps, 0 stops, frame= Minus2 at 2507616..2508514 in the same strand
misc_feature no id HMMPfam hit to PF01527, Transposase, score 1.1e-20 intersects 5%(4aa) with IS3_IS150_ORF1 e-value= 1.5e-33 complete sequence hit coverage= 100%, length is 300aa with 10 gaps, 0 stops, frame= Minus2 at 2507616..2508514 in the same strand
CDS YP_001972257.1 putative transposase (pseudogene) intersects 96.52%(277aa) with IS3_IS3_ORF1 e-value= 2.4e-72 complete sequence hit coverage= 100%, length is 278aa with 10 gaps, 0 stops, frame= Minus2 at 2507625..2508457 in the same strand
misc_feature no id HMMPfam hit to PF00665, Integrase core domain, score 3.3e-32 intersects 98.76%(159aa) with IS3_IS3_ORF1 e-value= 2.4e-72 complete sequence hit coverage= 100%, length is 278aa with 10 gaps, 0 stops, frame= Minus2 at 2507625..2508457 in the same strand
misc_feature no id putative transposase OrfB; Reviewed; Region: PHA02517 intersects 100%(250aa) with IS3_IS3_ORF1 e-value= 2.4e-72 complete sequence hit coverage= 100%, length is 278aa with 10 gaps, 0 stops, frame= Minus2 at 2507625..2508457 in the same strand
misc_feature no id Integrase core domain; Region: rve; cl01316 intersects 100%(117aa) with IS3_IS3_ORF1 e-value= 2.4e-72 complete sequence hit coverage= 100%, length is 278aa with 10 gaps, 0 stops, frame= Minus2 at 2507625..2508457 in the same strand

CDS YP_001972257.1 putative transposase (pseudogene) intersects 95.01%(272aa) with IS3_IS3_ORF2 e-value= 2.6e-66 complete sequence hit coverage= 100%, length is 273aa with 19 gaps, 0 stops, frame= Minus2 at 2507628..2508445 in the same strand
misc_feature no id HMMPfam hit to PF00665, Integrase core domain, score 3.3e-32 intersects 98.14%(158aa) with IS3_IS3_ORF2 e-value= 2.6e-66 complete sequence hit coverage= 100%, length is 273aa with 19 gaps, 0 stops, frame= Minus2 at 2507628..2508445 in the same strand
misc_feature no id putative transposase OrfB; Reviewed; Region: PHA02517 intersects 100%(250aa) with IS3_IS3_ORF2 e-value= 2.6e-66 complete sequence hit coverage= 100%, length is 273aa with 19 gaps, 0 stops, frame= Minus2 at 2507628..2508445 in the same strand
misc_feature no id Integrase core domain; Region: rve; cl01316 intersects 100%(117aa) with IS3_IS3_ORF2 e-value= 2.6e-66 complete sequence hit coverage= 100%, length is 273aa with 19 gaps, 0 stops, frame= Minus2 at 2507628..2508445 in the same strand
CDS YP_001972257.1 putative transposase (pseudogene) intersects 94.43%(271aa) with IS3_IS150_ORF2 e-value= 1.6e-25 complete sequence hit coverage= 100%, length is 360aa with 39 gaps, 1 stops, frame= Minus2 at 2507643..2508721 in the same strand
misc_feature no id HMMPfam hit to PF00665, Integrase core domain, score 3.3e-32 intersects 95.03%(153aa) with IS3_IS150_ORF2 e-value= 1.6e-25 complete sequence hit coverage= 100%, length is 360aa with 39 gaps, 1 stops, frame= Minus2 at 2507643..2508721 in the same strand
misc_feature no id putative transposase OrfB; Reviewed; Region: PHA02517 intersects 100%(250aa) with IS3_IS150_ORF2 e-value= 1.6e-25 complete sequence hit coverage= 100%, length is 360aa with 39 gaps, 1 stops, frame= Minus2 at 2507643..2508721 in the same strand
misc_feature no id Integrase core domain; Region: rve; cl01316 intersects 100%(117aa) with IS3_IS150_ORF2 e-value= 1.6e-25 complete sequence hit coverage= 100%, length is 360aa with 39 gaps, 1 stops, frame= Minus2 at 2507643..2508721 in the same strand
CDS YP_001972258.1 putative ISXac3 like transposase intersects 89.58%(86aa) with IS3_IS150_ORF2 e-value= 1.6e-25 complete sequence hit coverage= 100%, length is 360aa with 39 gaps, 1 stops, frame= Minus2 at 2507643..2508721 in the same strand
misc_feature no id HMMPfam hit to PF01527, Transposase, score 1.1e-20 intersects 91.25%(73aa) with IS3_IS150_ORF2 e-value= 1.6e-25 complete sequence hit coverage= 100%, length is 360aa with 39 gaps, 1 stops, frame= Minus2 at 2507643..2508721 in the same strand
misc_feature no id Helix-turn-helix domain of Hin and related proteins, a family of DNA-binding domains unique to bacteria and represented by the Hin protein of Salmonella. The basic HTH domain is a simple fold comprised of three core helices that form a right-handed...; Region: HTH_Hin_like; cl01116 intersects 86.67%(65aa) with IS3_IS150_ORF2 e-value= 1.6e-25 complete sequence hit coverage= 100%, length is 360aa with 39 gaps, 1 stops, frame= Minus2 at 2507643..2508721 in the same strand
CDS YP_001972257.1 putative transposase (pseudogene) intersects 92.57%(265aa) with IS3_IS3_ORF2 e-value= 2.2e-69 fragment hit coverage= 93.75%, length is 266aa with 8 gaps, 0 stops, frame= Minus2 at 2507649..2508445 in the same strand
misc_feature no id HMMPfam hit to PF00665, Integrase core domain, score 3.3e-32 intersects 93.79%(151aa) with IS3_IS3_ORF2 e-value= 2.2e-69 fragment hit coverage= 93.75%, length is 266aa with 8 gaps, 0 stops, frame= Minus2 at 2507649..2508445 in the same strand
misc_feature no id putative transposase OrfB; Reviewed; Region: PHA02517 intersects 100%(250aa) with IS3_IS3_ORF2 e-value= 2.2e-69 fragment hit coverage= 93.75%, length is 266aa with 8 gaps, 0 stops, frame= Minus2 at 2507649..2508445 in the same strand
misc_feature no id Integrase core domain; Region: rve; cl01316 intersects 100%(117aa) with IS3_IS3_ORF2 e-value= 2.2e-69 fragment hit coverage= 93.75%, length is 266aa with 8 gaps, 0 stops, frame= Minus2 at 2507649..2508445 in the same strand
CDS YP_001972257.1 putative transposase (pseudogene) intersects 91.99%(264aa) with IS3_IS3_ORF1 e-value= 4e-75 fragment hit coverage= 94.06%, length is 265aa with 6 gaps, 0 stops, frame= Minus2 at 2507664..2508457 in the same strand
misc_feature no id HMMPfam hit to PF00665, Integrase core domain, score 3.3e-32 intersects 90.68%(146aa) with IS3_IS3_ORF1 e-value= 4e-75 fragment hit coverage= 94.06%, length is 265aa with 6 gaps, 0 stops, frame= Minus2 at 2507664..2508457 in the same strand
misc_feature no id putative transposase OrfB; Reviewed; Region: PHA02517 intersects 98.80%(247aa) with IS3_IS3_ORF1 e-value= 4e-75 fragment hit coverage= 94.06%, length is 265aa with 6 gaps, 0 stops, frame= Minus2 at 2507664..2508457 in the same strand

misc_feature no id Integrase core domain; Region: rve; cl01316 intersects 100%(117aa) with IS3_IS3_ORF1 e-value= 4e-75 fragment hit coverage= 94.06%, length is 265aa with 6 gaps, 0 stops, frame= Minus2 at 2507664..2508457 in the same strand
CDS YP_001972257.1 putative transposase (pseudogene) intersects 91.64%(263aa) with IS3_IS51_ORF1 e-value= 1.7e-73 fragment hit coverage= 90.88%, length is 265aa with 15 gaps, 0 stops, frame= Minus2 at 2507667..2508460 in the same strand
misc_feature no id HMMPfam hit to PF00665, Integrase core domain, score 3.3e-32 intersects 90.06%(145aa) with IS3_IS51_ORF1 e-value= 1.7e-73 fragment hit coverage= 90.88%, length is 265aa with 15 gaps, 0 stops, frame= Minus2 at 2507667..2508460 in the same strand
misc_feature no id putative transposase OrfB; Reviewed; Region: PHA02517 intersects 98.40%(246aa) with IS3_IS51_ORF1 e-value= 1.7e-73 fragment hit coverage= 90.88%, length is 265aa with 15 gaps, 0 stops, frame= Minus2 at 2507667..2508460 in the same strand
misc_feature no id Integrase core domain; Region: rve; cl01316 intersects 100%(117aa) with IS3_IS51_ORF1 e-value= 1.7e-73 fragment hit coverage= 90.88%, length is 265aa with 15 gaps, 0 stops, frame= Minus2 at 2507667..2508460 in the same strand
CDS YP_001972257.1 putative transposase (pseudogene) intersects 87.69%(251aa) with IS3_IS51_ORF2 e-value= 1.5e-73 fragment hit coverage= 83.28%, length is 252aa with 15 gaps, 0 stops, frame= Minus2 at 2507670..2508424 in the same strand
misc_feature no id HMMPfam hit to PF00665, Integrase core domain, score 3.3e-32 intersects 89.44%(144aa) with IS3_IS51_ORF2 e-value= 1.5e-73 fragment hit coverage= 83.28%, length is 252aa with 15 gaps, 0 stops, frame= Minus2 at 2507670..2508424 in the same strand
misc_feature no id putative transposase OrfB; Reviewed; Region: PHA02517 intersects 98%(245aa) with IS3_IS51_ORF2 e-value= 1.5e-73 fragment hit coverage= 83.28%, length is 252aa with 15 gaps, 0 stops, frame= Minus2 at 2507670..2508424 in the same strand
misc_feature no id Integrase core domain; Region: rve; cl01316 intersects 100%(117aa) with IS3_IS51_ORF2 e-value= 1.5e-73 fragment hit coverage= 83.28%, length is 252aa with 15 gaps, 0 stops, frame= Minus2 at 2507670..2508424 in the same strand
CDS YP_001972257.1 putative transposase (pseudogene) intersects 83.86%(240aa) with IS3_IS150_ORF1 e-value= 2.4e-37 fragment hit coverage= 80.39%, length is 241aa with 7 gaps, 0 stops, frame= Minus2 at 2507673..2508394 in the same strand
misc_feature no id HMMPfam hit to PF00665, Integrase core domain, score 3.3e-32 intersects 88.82%(143aa) with IS3_IS150_ORF1 e-value= 2.4e-37 fragment hit coverage= 80.39%, length is 241aa with 7 gaps, 0 stops, frame= Minus2 at 2507673..2508394 in the same strand
misc_feature no id putative transposase OrfB; Reviewed; Region: PHA02517 intersects 96.27%(240aa) with IS3_IS150_ORF1 e-value= 2.4e-37 fragment hit coverage= 80.39%, length is 241aa with 7 gaps, 0 stops, frame= Minus2 at 2507673..2508394 in the same strand
misc_feature no id Integrase core domain; Region: rve; cl01316 intersects 100%(117aa) with IS3_IS150_ORF1 e-value= 2.4e-37 fragment hit coverage= 80.39%, length is 241aa with 7 gaps, 0 stops, frame= Minus2 at 2507673..2508394 in the same strand
CDS YP_001972257.1 putative transposase (pseudogene) intersects 89.55%(257aa) with IS3_IS150_ORF2 e-value= 1.7e-58 fragment hit coverage= 68.89%, length is 258aa with 16 gaps, 0 stops, frame= Minus2 at 2507685..2508457 in the same strand
misc_feature no id HMMPfam hit to PF00665, Integrase core domain, score 3.3e-32 intersects 86.34%(139aa) with IS3_IS150_ORF2 e-value= 1.7e-58 fragment hit coverage= 68.89%, length is 258aa with 16 gaps, 0 stops, frame= Minus2 at 2507685..2508457 in the same strand
misc_feature no id putative transposase OrfB; Reviewed; Region: PHA02517 intersects 96%(240aa) with IS3_IS150_ORF2 e-value= 1.7e-58 fragment hit coverage= 68.89%, length is 258aa with 16 gaps, 0 stops, frame= Minus2 at 2507685..2508457 in the same strand
misc_feature no id Integrase core domain; Region: rve; cl01316 intersects 100%(117aa) with IS3_IS150_ORF2 e-value= 1.7e-58 fragment hit coverage= 68.89%, length is 258aa with 16 gaps, 0 stops, frame= Minus2 at 2507685..2508457 in the same strand
Class A

36.
CDS YP_001972267.1 putative ISXac3 like element protein intersects 100%(284aa) with IS3_IS51_ORF2 e-value= 4.4e-135 complete sequence hit coverage= 100%, length is 306aa with 15 gaps, 0 stops, frame= Minus1 at 2519842..2520758 in the same strand

misc_feature	no id	putative transposase OrfB; Reviewed; Region: PHA02517 intersects 100%(263aa) with IS3_IS51_ORF2 e-value= 4.4e-135 complete sequence hit coverage= 100%, length is 306aa with 15 gaps, 0 stops, frame= Minus1 at 2519842..2520758 in the same strand
misc_feature	no id	HMMPfam hit to PF00665, Integrase core domain, score 9e-51 intersects 100%(161aa) with IS3_IS51_ORF2 e-value= 4.4e-135 complete sequence hit coverage= 100%, length is 306aa with 15 gaps, 0 stops, frame= Minus1 at 2519842..2520758 in the same strand
misc_feature	no id	Integrase core domain; Region: rve; cl01316 intersects 100%(117aa) with IS3_IS51_ORF2 e-value= 4.4e-135 complete sequence hit coverage= 100%, length is 306aa with 15 gaps, 0 stops, frame= Minus1 at 2519842..2520758 in the same strand
CDS	YP_001972268.1	putative ISXac3 like transposase family protein intersects 3.30%(3aa) with IS3_IS51_ORF2 e-value= 4.4e-135 complete sequence hit coverage= 100%, length is 306aa with 15 gaps, 0 stops, frame= Minus1 at 2519842..2520758 in the same strand
CDS	YP_001972267.1	putative ISXac3 like element protein intersects 100%(284aa) with IS481	e-value= 4.4e-07 complete sequence hit coverage= 100%, length is 315aa with 40 gaps, 1 stops, frame= Minus1 at 2519854..2520797 in the same strand
misc_feature	no id	putative transposase OrfB; Reviewed; Region: PHA02517 intersects 100%(263aa) with IS481 e-value= 4.4e-07 complete sequence hit coverage= 100%, length is 315aa with 40 gaps, 1 stops, frame= Minus1 at 2519854..2520797 in the same strand
misc_feature	no id	HMMPfam hit to PF00665, Integrase core domain, score 9e-51 intersects 100%(161aa) with IS481	e-value= 4.4e-07 complete sequence hit coverage= 100%, length is 315aa with 40 gaps, 1 stops, frame= Minus1 at 2519854..2520797 in the same strand
misc_feature	no id	Integrase core domain; Region: rve; cl01316 intersects 100%(117aa) with IS481	e-value= 4.4e-07 complete sequence hit coverage= 100%, length is 315aa with 40 gaps, 1 stops, frame= Minus1 at 2519854..2520797 in the same strand
CDS	YP_001972268.1	putative ISXac3 like transposase family protein intersects 17.58%(16aa) with IS481 e-value= 4.4e-07 complete sequence hit coverage= 100%, length is 315aa with 40 gaps, 1 stops, frame= Minus1 at 2519854..2520797 in the same strand
misc_feature	no id	HMMPfam hit to PF01527, Transposase, score 1.1e-21 intersects 6.41%(5aa) with IS481 e-value= 4.4e-07 complete sequence hit coverage= 100%, length is 315aa with 40 gaps, 1 stops, frame= Minus1 at 2519854..2520797 in the same strand
CDS	YP_001972267.1	putative ISXac3 like element protein intersects 100%(284aa) with IS3_IS51_ORF1 e-value= 3.7e-108 complete sequence hit coverage= 100%, length is 293aa with 14 gaps, 1 stops, frame= Minus1 at 2519863..2520740 in the same strand
misc_feature	no id	putative transposase OrfB; Reviewed; Region: PHA02517 intersects 100%(263aa) with IS3_IS51_ORF1 e-value= 3.7e-108 complete sequence hit coverage= 100%, length is 293aa with 14 gaps, 1 stops, frame= Minus1 at 2519863..2520740 in the same strand
misc_feature	no id	HMMPfam hit to PF00665, Integrase core domain, score 9e-51 intersects 100%(161aa) with IS3_IS51_ORF1 e-value= 3.7e-108 complete sequence hit coverage= 100%, length is 293aa with 14 gaps, 1 stops, frame= Minus1 at 2519863..2520740 in the same strand
misc_feature	no id	Integrase core domain; Region: rve; cl01316 intersects 100%(117aa) with IS3_IS51_ORF1 e-value= 3.7e-108 complete sequence hit coverage= 100%, length is 293aa with 14 gaps, 1 stops, frame= Minus1 at 2519863..2520740 in the same strand
CDS	YP_001972267.1	putative ISXac3 like element protein intersects 100%(284aa) with IS3_IS2_ORF2 e-value= 1.4e-07 complete sequence hit coverage= 100%, length is 298aa with 24 gaps, 1 stops, frame= Minus1 at 2519878..2520770 in the same strand
misc_feature	no id	putative transposase OrfB; Reviewed; Region: PHA02517 intersects 100%(263aa) with IS3_IS2_ORF2	e-value= 1.4e-07 complete sequence hit coverage= 100%, length is 298aa with 24 gaps, 1 stops, frame= Minus1 at 2519878..2520770 in the same strand
misc_feature	no id	HMMPfam hit to PF00665, Integrase core domain, score 9e-51 intersects 100%(161aa) with IS3_IS2_ORF2	e-value= 1.4e-07 complete sequence hit coverage= 100%, length is 298aa with 24 gaps, 1 stops, frame= Minus1 at 2519878..2520770 in the same strand
misc_feature	no id	Integrase core domain; Region: rve; cl01316 intersects 100%(117aa) with IS3_IS2_ORF2 e-value= 1.4e-07 complete sequence hit coverage= 100%, length is 298aa with 24 gaps, 1 stops, frame= Minus1 at 2519878..2520770 in the same strand
CDS	YP_001972268.1	putative ISXac3 like transposase family protein intersects 7.69%(7aa) with IS3_IS2_ORF2	e-value= 1.4e-07 complete sequence hit coverage= 100%, length is 298aa with 24 gaps, 1 stops, frame= Minus1 at 2519878..2520770 in the same strand

CDS YP_001972267.1 putative ISXac3 like element protein intersects 99.18%(281aa) with IS3_IS3_ORF2 e-value= 2e-102 complete sequence hit coverage= 100%, length is 282aa with 7 gaps, 1 stops, frame= Minus1 at 2519881..2520725 in the same strand
misc_feature no id putative transposase OrfB; Reviewed; Region: PHA02517 intersects 100%(263aa) with IS3_IS3_ORF2 e-value= 2e-102 complete sequence hit coverage= 100%, length is 282aa with 7 gaps, 1 stops, frame= Minus1 at 2519881..2520725 in the same strand
misc_feature no id HMMPfam hit to PF00665, Integrase core domain, score 9e-51 intersects 100%(161aa) with IS3_IS3_ORF2 e-value= 2e-102 complete sequence hit coverage= 100%, length is 282aa with 7 gaps, 1 stops, frame= Minus1 at 2519881..2520725 in the same strand
misc_feature no id Integrase core domain; Region: rve; cl01316 intersects 100%(117aa) with IS3_IS3_ORF2 e-value= 2e-102 complete sequence hit coverage= 100%, length is 282aa with 7 gaps, 1 stops, frame= Minus1 at 2519881..2520725 in the same strand
CDS YP_001972267.1 putative ISXac3 like element protein intersects 97.07%(275aa) with IS3_IS150_ORF2 e-value= 1.9e-74 fragment hit coverage= 73.52%, length is 276aa with 11 gaps, 0 stops, frame= Minus1 at 2519887..2520713 in the same strand
misc_feature no id putative transposase OrfB; Reviewed; Region: PHA02517 intersects 100%(263aa) with IS3_IS150_ORF2 e-value= 1.9e-74 fragment hit coverage= 73.52%, length is 276aa with 11 gaps, 0 stops, frame= Minus1 at 2519887..2520713 in the same strand
misc_feature no id HMMPfam hit to PF00665, Integrase core domain, score 9e-51 intersects 100%(161aa) with IS3_IS150_ORF2 e-value= 1.9e-74 fragment hit coverage= 73.52%, length is 276aa with 11 gaps, 0 stops, frame= Minus1 at 2519887..2520713 in the same strand
misc_feature no id Integrase core domain; Region: rve; cl01316 intersects 100%(117aa) with IS3_IS150_ORF2 e-value= 1.9e-74 fragment hit coverage= 73.52%, length is 276aa with 11 gaps, 0 stops, frame= Minus1 at 2519887..2520713 in the same strand
CDS YP_001972267.1 putative ISXac3 like element protein intersects 99.30%(282aa) with IS3_IS150_ORF2 e-value= 1.2e-45 complete sequence hit coverage= 100%, length is 370aa with 21 gaps, 4 stops, frame= Minus1 at 2519887..2520995 in the same strand
misc_feature no id putative transposase OrfB; Reviewed; Region: PHA02517 intersects 100%(263aa) with IS3_IS150_ORF2 e-value= 1.2e-45 complete sequence hit coverage= 100%, length is 370aa with 21 gaps, 4 stops, frame= Minus1 at 2519887..2520995 in the same strand
misc_feature no id HMMPfam hit to PF00665, Integrase core domain, score 9e-51 intersects 100%(161aa) with IS3_IS150_ORF2 e-value= 1.2e-45 complete sequence hit coverage= 100%, length is 370aa with 21 gaps, 4 stops, frame= Minus1 at 2519887..2520995 in the same strand
misc_feature no id Integrase core domain; Region: rve; cl01316 intersects 100%(117aa) with IS3_IS150_ORF2 e-value= 1.2e-45 complete sequence hit coverage= 100%, length is 370aa with 21 gaps, 4 stops, frame= Minus1 at 2519887..2520995 in the same strand
CDS YP_001972268.1 putative ISXac3 like transposase family protein intersects 90.11%(82aa) with IS3_IS150_ORF2 e-value= 1.2e-45 complete sequence hit coverage= 100%, length is 370aa with 21 gaps, 4 stops, frame= Minus1 at 2519887..2520995 in the same strand
misc_feature no id HMMPfam hit to PF01527, Transposase, score 1.1e-21 intersects 91.03%(71aa) with IS3_IS150_ORF2 e-value= 1.2e-45 complete sequence hit coverage= 100%, length is 370aa with 21 gaps, 4 stops, frame= Minus1 at 2519887..2520995 in the same strand
misc_feature no id Helix-turn-helix domain of Hin and related proteins, a family of DNA-binding domains unique to bacteria and represented by the Hin protein of Salmonella. The basic HTH domain is a simple fold comprised of three core helices that form a right-handed...; Region: HTH_Hin_like; cl01116 intersects 91.30%(63aa) with IS3_IS150_ORF2 e-value= 1.2e-45 complete sequence hit coverage= 100%, length is 370aa with 21 gaps, 4 stops, frame= Minus1 at 2519887..2520995 in the same strand
CDS YP_001972267.1 putative ISXac3 like element protein intersects 98.94%(281aa) with IS3_IS150_ORF1 e-value= 5.1e-57 fragment hit coverage= 97.06%, length is 293aa with 7 gaps, 0 stops, frame= Minus1 at 2519890..2520767 in the same strand
misc_feature no id putative transposase OrfB; Reviewed; Region: PHA02517 intersects 100%(263aa) with IS3_IS150_ORF1 e-value= 5.1e-57 fragment hit coverage= 97.06%, length is 293aa with 7 gaps, 0 stops, frame= Minus1 at 2519890..2520767 in the same strand
misc_feature no id HMMPfam hit to PF00665, Integrase core domain, score 9e-51 intersects 100%(161aa) with IS3_IS150_ORF1 e-value= 5.1e-57 fragment hit coverage= 97.06%, length is 293aa with 7 gaps, 0 stops, frame= Minus1 at 2519890..2520767 in the same strand

misc_feature no id Integrase core domain; Region: rve; cl01316 intersects 100%(117aa) with IS3_IS150_ORF1 e-value= 5.1e-57 fragment hit coverage= 97.06%, length is 293aa with 7 gaps, 0 stops, frame= Minus1 at 2519890..2520767 in the same strand
CDS YP_001972268.1 putative ISXac3 like transposase family protein intersects 6.59%(6aa) with IS3_IS150_ORF1 e-value= 5.1e-57 fragment hit coverage= 97.06%, length is 293aa with 7 gaps, 0 stops, frame= Minus1 at 2519890..2520767 in the same strand
CDS YP_001972267.1 putative ISXac3 like element protein intersects 98.94%(281aa) with IS3_IS150_ORF1 e-value= 1.4e-57 complete sequence hit coverage= 100%, length is 302aa with 7 gaps, 0 stops, frame= Minus1 at 2519890..2520794 in the same strand
misc_feature no id putative transposase OrfB; Reviewed; Region: PHA02517 intersects 100%(263aa) with IS3_IS150_ORF1 e-value= 1.4e-57 complete sequence hit coverage= 100%, length is 302aa with 7 gaps, 0 stops, frame= Minus1 at 2519890..2520794 in the same strand
misc_feature no id HMMPfam hit to PF00665, Integrase core domain, score 9e-51 intersects 100%(161aa) with IS3_IS150_ORF1 e-value= 1.4e-57 complete sequence hit coverage= 100%, length is 302aa with 7 gaps, 0 stops, frame= Minus1 at 2519890..2520794 in the same strand
misc_feature no id Integrase core domain; Region: rve; cl01316 intersects 100%(117aa) with IS3_IS150_ORF1 e-value= 1.4e-57 complete sequence hit coverage= 100%, length is 302aa with 7 gaps, 0 stops, frame= Minus1 at 2519890..2520794 in the same strand
CDS YP_001972268.1 putative ISXac3 like transposase family protein intersects 16.48%(15aa) with IS3_IS150_ORF1 e-value= 1.4e-57 complete sequence hit coverage= 100%, length is 302aa with 7 gaps, 0 stops, frame= Minus1 at 2519890..2520794 in the same strand
misc_feature no id HMMPfam hit to PF01527, Transposase, score 1.1e-21 intersects 5.13%(4aa) with IS3_IS150_ORF1 e-value= 1.4e-57 complete sequence hit coverage= 100%, length is 302aa with 7 gaps, 0 stops, frame= Minus1 at 2519890..2520794 in the same strand
CDS YP_001972267.1 putative ISXac3 like element protein intersects 98.59%(280aa) with IS3_IS3_ORF1 e-value= 4.9e-95 complete sequence hit coverage= 100%, length is 282aa with 6 gaps, 0 stops, frame= Minus1 at 2519893..2520737 in the same strand
misc_feature no id putative transposase OrfB; Reviewed; Region: PHA02517 intersects 100%(263aa) with IS3_IS3_ORF1 e-value= 4.9e-95 complete sequence hit coverage= 100%, length is 282aa with 6 gaps, 0 stops, frame= Minus1 at 2519893..2520737 in the same strand
misc_feature no id HMMPfam hit to PF00665, Integrase core domain, score 9e-51 intersects 100%(161aa) with IS3_IS3_ORF1 e-value= 4.9e-95 complete sequence hit coverage= 100%, length is 282aa with 6 gaps, 0 stops, frame= Minus1 at 2519893..2520737 in the same strand
misc_feature no id Integrase core domain; Region: rve; cl01316 intersects 100%(117aa) with IS3_IS3_ORF1 e-value= 4.9e-95 complete sequence hit coverage= 100%, length is 282aa with 6 gaps, 0 stops, frame= Minus1 at 2519893..2520737 in the same strand
CDS YP_001972267.1 putative ISXac3 like element protein intersects 96.01%(272aa) with IS3_IS51_ORF2 e-value= 7.1e-152 fragment hit coverage= 90.22%, length is 273aa with 13 gaps, 0 stops, frame= Minus1 at 2519893..2520710 in the same strand
misc_feature no id putative transposase OrfB; Reviewed; Region: PHA02517 intersects 100%(263aa) with IS3_IS51_ORF2 e-value= 7.1e-152 fragment hit coverage= 90.22%, length is 273aa with 13 gaps, 0 stops, frame= Minus1 at 2519893..2520710 in the same strand
misc_feature no id HMMPfam hit to PF00665, Integrase core domain, score 9e-51 intersects 100%(161aa) with IS3_IS51_ORF2 e-value= 7.1e-152 fragment hit coverage= 90.22%, length is 273aa with 13 gaps, 0 stops, frame= Minus1 at 2519893..2520710 in the same strand
misc_feature no id Integrase core domain; Region: rve; cl01316 intersects 100%(117aa) with IS3_IS51_ORF2 e-value= 7.1e-152 fragment hit coverage= 90.22%, length is 273aa with 13 gaps, 0 stops, frame= Minus1 at 2519893..2520710 in the same strand
CDS YP_001972267.1 putative ISXac3 like element protein intersects 98.24%(279aa) with IS3_IS51_ORF1 e-value= 3.9e-110 fragment hit coverage= 96.42%, length is 282aa with 14 gaps, 0 stops, frame= Minus1 at 2519896..2520740 in the same strand
misc_feature no id putative transposase OrfB; Reviewed; Region: PHA02517 intersects 100%(263aa) with IS3_IS51_ORF1 e-value= 3.9e-110 fragment hit coverage= 96.42%, length is 282aa with 14 gaps, 0 stops, frame= Minus1 at 2519896..2520740 in the same strand
misc_feature no id HMMPfam hit to PF00665, Integrase core domain, score 9e-51 intersects 100%(161aa) with IS3_IS51_ORF1 e-value= 3.9e-110 fragment hit coverage= 96.42%, length is 282aa with 14 gaps, 0 stops, frame= Minus1 at 2519896..2520740 in the same strand

misc_feature no id Integrase core domain; Region: rve; cl01316 intersects 100%(117aa) with IS3_IS51_ORF1 e-value= 3.9e-110 fragment hit coverage= 96.42%, length is 282aa with 14 gaps, 0 stops, frame= Minus1 at 2519896..2520740 in the same strand
CDS YP_001972267.1 putative ISXac3 like element protein intersects 0.12%(0aa) with IS3_IS51_ORF1 e-value= 3.2e-08 fragment hit coverage= 14.66%, length is 43aa with 2 gaps, 1 stops, frame= Minus2 at 2520732..2520859 in the same strand
CDS YP_001972268.1 putative ISXac3 like transposase family protein intersects 40.29%(36aa) with IS3_IS51_ORF1 e-value= 3.2e-08 fragment hit coverage= 14.66%, length is 43aa with 2 gaps, 1 stops, frame= Minus2 at 2520732..2520859 in the same strand
misc_feature no id HMMPfam hit to PF01527, Transposase, score 1.1e-21 intersects 32.91%(25aa) with IS3_IS51_ORF1 e-value= 3.2e-08 fragment hit coverage= 14.66%, length is 43aa with 2 gaps, 1 stops, frame= Minus2 at 2520732..2520859 in the same strand
misc_feature no id Helix-turn-helix domain of Hin and related proteins, a family of DNA-binding domains unique to bacteria and represented by the Hin protein of Salmonella. The basic HTH domain is a simple fold comprised of three core helices that form a right-handed...; Region: HTH_Hin_like; cl01116 intersects 25.60%(17aa) with IS3_IS51_ORF1 e-value= 3.2e-08 fragment hit coverage= 14.66%, length is 43aa with 2 gaps, 1 stops, frame= Minus2 at 2520732..2520859 in the same strand
Class A

37.
CDS YP_001972268.1 putative ISXac3 like transposase family protein intersects 52.38%(47aa) with IS3_IS407_ORF1 e-value= 7.7e-06 fragment hit coverage= 55.81%, length is 48aa with 0 gaps, 0 stops, frame= Minus2 at 2520870..2521012 in the same strand
misc_feature no id HMMPfam hit to PF01527, Transposase, score 1.1e-21 intersects 61.11%(47aa) with IS3_IS407_ORF1 e-value= 7.7e-06 fragment hit coverage= 55.81%, length is 48aa with 0 gaps, 0 stops, frame= Minus2 at 2520870..2521012 in the same strand
misc_feature no id Helix-turn-helix domain of Hin and related proteins, a family of DNA-binding domains unique to bacteria and represented by the Hin protein of Salmonella. The basic HTH domain is a simple fold comprised of three core helices that form a right-handed...; Region: HTH_Hin_like; cl01116 intersects 69.08%(47aa) with IS3_IS407_ORF1 e-value= 7.7e-06 fragment hit coverage= 55.81%, length is 48aa with 0 gaps, 0 stops, frame= Minus2 at 2520870..2521012 in the same strand
Class A

38.
repeat_region no id no product intersects 83.65%(296aa) with IS110 e-value= 3.1e-48 complete sequence hit coverage= 100%, length is 303aa with 113 gaps, 0 stops, frame= Plus2 at 2756306..2757214 in the opposite strand
CDS YP_001972500.1 putative transposase intersects 99.34%(303aa) with IS110 e-value= 3.1e-48 complete sequence hit coverage= 100%, length is 303aa with 113 gaps, 0 stops, frame= Plus2 at 2756306..2757214 in the same strand
misc_feature no id Transposase and inactivated derivatives [DNA replication, recombination, and repair]; Region: COG3547 intersects 100%(290aa) with IS110 e-value= 3.1e-48 complete sequence hit coverage= 100%, length is 303aa with 113 gaps, 0 stops, frame= Plus2 at 2756306..2757214 in the same strand
misc_feature no id Transposase; Region: Transposase_9; pfam01548 intersects 100%(85aa) with IS110 e-value= 3.1e-48 complete sequence hit coverage= 100%, length is 303aa with 113 gaps, 0 stops, frame= Plus2 at 2756306..2757214 in the same strand
misc_feature no id HMMPfam hit to PF01548, Transposase, score 2.6e-10 intersects 100%(99aa) with IS110 e-value= 3.1e-48 complete sequence hit coverage= 100%, length is 303aa with 113 gaps, 0 stops, frame= Plus2 at 2756306..2757214 in the same strand
misc_feature no id HMMPfam hit to PF02371, Transposase IS116/IS110/IS902 family, score 2e-24 intersects 100%(108aa) with IS110 e-value= 3.1e-48 complete sequence hit coverage= 100%, length is 303aa with 113 gaps, 0 stops, frame= Plus2 at 2756306..2757214 in the same strand
misc_feature no id Transposase IS116/IS110/IS902 family; Region: Transposase_20; pfam02371 intersects 100%(79aa) with IS110 e-value= 3.1e-48 complete sequence hit coverage= 100%, length is 303aa with 113 gaps, 0 stops, frame= Plus2 at 2756306..2757214 in the same strand
repeat_region no id no product intersects 82.33%(292aa) with IS110 e-value= 1.4e-51 fragment hit coverage= 96.15%, length is 292aa with 108 gaps, 0 stops, frame= Plus2 at 2756306..2757181 in the opposite strand

CDS YP_001972500.1 putative transposase intersects 95.74%(292aa) with IS110 e-value= 1.4e-51 fragment hit coverage= 96.15%, length is 292aa with 108 gaps, 0 stops, frame= Plus2 at 2756306..2757181 in the same strand
misc_feature no id Transposase and inactivated derivatives [DNA replication, recombination, and repair]; Region: COG3547 intersects 100%(290aa) with IS110 e-value= 1.4e-51 fragment hit coverage= 96.15%, length is 292aa with 108 gaps, 0 stops, frame= Plus2 at 2756306..2757181 in the same strand
misc_feature no id Transposase; Region: Transposase_9; pfam01548 intersects 100%(85aa) with IS110 e-value= 1.4e-51 fragment hit coverage= 96.15%, length is 292aa with 108 gaps, 0 stops, frame= Plus2 at 2756306..2757181 in the same strand
misc_feature no id HMMPfam hit to PF01548, Transposase, score 2.6e-10 intersects 100%(99aa) with IS110 e-value= 1.4e-51 fragment hit coverage= 96.15%, length is 292aa with 108 gaps, 0 stops, frame= Plus2 at 2756306..2757181 in the same strand
misc_feature no id HMMPfam hit to PF02371, Transposase IS116/IS110/IS902 family, score 2e-24 intersects 100%(108aa) with IS110 e-value= 1.4e-51 fragment hit coverage= 96.15%, length is 292aa with 108 gaps, 0 stops, frame= Plus2 at 2756306..2757181 in the same strand
misc_feature no id Transposase IS116/IS110/IS902 family; Region: Transposase_20; pfam02371 intersects 100%(79aa) with IS110 e-value= 1.4e-51 fragment hit coverage= 96.15%, length is 292aa with 108 gaps, 0 stops, frame= Plus2 at 2756306..2757181 in the same strand
Class A

39.
misc_feature no id Helix-turn-helix domain of Hin and related proteins, a family of DNA-binding domains unique to bacteria and represented by the Hin protein of Salmonella. The basic HTH domain is a simple fold comprised of three core helices that form a right-handed...; Region: HTH_Hin_like; cl01116 intersects 100%(75aa) with IS3_IS150_ORF2 e-value= 7.4e-24 complete sequence hit coverage= 100%, length is 376aa with 32 gaps, 1 stops, frame= Plus2 at 3087821..3088948 in the same strand
CDS YP_001972786.1 putative ISXac3 like transposase intersects 100%(96aa) with IS3_IS150_ORF2 e-value= 7.4e-24 complete sequence hit coverage= 100%, length is 376aa with 32 gaps, 1 stops, frame= Plus2 at 3087821..3088948 in the same strand
misc_feature no id HMMPfam hit to PF01527, Transposase, score 1.4e-19 intersects 100%(80aa) with IS3_IS150_ORF2 e-value= 7.4e-24 complete sequence hit coverage= 100%, length is 376aa with 32 gaps, 1 stops, frame= Plus2 at 3087821..3088948 in the same strand
CDS YP_001972787.1 putative ISXac3 like transposase intersects 94.16%(258aa) with IS3_IS150_ORF2 e-value= 7.4e-24 complete sequence hit coverage= 100%, length is 376aa with 32 gaps, 1 stops, frame= Plus2 at 3087821..3088948 in the same strand
misc_feature no id putative transposase OrfB; Reviewed; Region: PHA02517 intersects 100%(250aa) with IS3_IS150_ORF2 e-value= 7.4e-24 complete sequence hit coverage= 100%, length is 376aa with 32 gaps, 1 stops, frame= Plus2 at 3087821..3088948 in the same strand
misc_feature no id HMMPfam hit to PF00665, Integrase core domain, score 7.9e-27 intersects 94.44%(153aa) with IS3_IS150_ORF2 e-value= 7.4e-24 complete sequence hit coverage= 100%, length is 376aa with 32 gaps, 1 stops, frame= Plus2 at 3087821..3088948 in the same strand
misc_feature no id Integrase core domain; Region: rve; cl01316 intersects 100%(116aa) with IS3_IS150_ORF2 e-value= 7.4e-24 complete sequence hit coverage= 100%, length is 376aa with 32 gaps, 1 stops, frame= Plus2 at 3087821..3088948 in the same strand
CDS YP_001972786.1 putative ISXac3 like transposase intersects 18.06%(17aa) with IS3_IS150_ORF1 e-value= 6e-28 complete sequence hit coverage= 100%, length is 300aa with 11 gaps, 0 stops, frame= Plus2 at 3088076..3088975 in the same strand
misc_feature no id HMMPfam hit to PF01527, Transposase, score 1.4e-19 intersects 5.42%(4aa) with IS3_IS150_ORF1 e-value= 6e-28 complete sequence hit coverage= 100%, length is 300aa with 11 gaps, 0 stops, frame= Plus2 at 3088076..3088975 in the same strand
CDS YP_001972787.1 putative ISXac3 like transposase intersects 97.45%(267aa) with IS3_IS150_ORF1 e-value= 6e-28 complete sequence hit coverage= 100%, length is 300aa with 11 gaps, 0 stops, frame= Plus2 at 3088076..3088975 in the same strand
misc_feature no id putative transposase OrfB; Reviewed; Region: PHA02517 intersects 100%(250aa) with IS3_IS150_ORF1 e-value= 6e-28 complete sequence hit coverage= 100%, length is 300aa with 11 gaps, 0 stops, frame= Plus2 at 3088076..3088975 in the same strand

misc_feature no id HMMPfam hit to PF00665, Integrase core domain, score 7.9e-27 intersects 100%(162aa) with IS3_IS150_ORF1 e-value= 6e-28 complete sequence hit coverage= 100%, length is 300aa with 11 gaps, 0 stops, frame= Plus2 at 3088076..3088975 in the same strand
misc_feature no id Integrase core domain; Region: rve; cl01316 intersects 100%(116aa) with IS3_IS150_ORF1 e-value= 6e-28 complete sequence hit coverage= 100%, length is 300aa with 11 gaps, 0 stops, frame= Plus2 at 3088076..3088975 in the same strand
misc_feature no id putative integrase, pseudogene;Similar to N-terminus to codon 110 of Xanthomonas campestris pv. vesicatoria (strain 85-10). phage-related integrase. UniProt:Q3BUY5 (EMBL:AM039952 (453 aa) fasta scores: E()=1e-18, 58.095% id in 105 aa; similarity:fasta; with=UniProt:Q3BUY5 (EMBL:AM039952;); Xanthomonas campestris pv. vesicatoria (strain 85-10).; Phage-related integrase.; length=453; id 58.095%; ungapped id 58.095%; E()=1e-18; 105 aa overlap; query 1-105; subject 1-105 intersects 6.83%(8aa) with IS3_IS150_ORF1 e-value= 6e-28 complete sequence hit coverage= 100%, length is 300aa with 11 gaps, 0 stops, frame= Plus2 at 3088076..3088975 in the opposite strand
CDS YP_001972786.1 putative ISXac3 like transposase intersects 5.56%(5aa) with IS3_IS51_ORF2 e-value= 9.2e-49 complete sequence hit coverage= 100%, length is 304aa with 17 gaps, 2 stops, frame= Plus2 at 3088112..3089023 in the same strand
CDS YP_001972787.1 putative ISXac3 like transposase intersects 100%(274aa) with IS3_IS51_ORF2 e-value= 9.2e-49 complete sequence hit coverage= 100%, length is 304aa with 17 gaps, 2 stops, frame= Plus2 at 3088112..3089023 in the same strand
misc_feature no id putative transposase OrfB; Reviewed; Region: PHA02517 intersects 100%(250aa) with IS3_IS51_ORF2 e-value= 9.2e-49 complete sequence hit coverage= 100%, length is 304aa with 17 gaps, 2 stops, frame= Plus2 at 3088112..3089023 in the same strand
misc_feature no id HMMPfam hit to PF00665, Integrase core domain, score 7.9e-27 intersects 100%(162aa) with IS3_IS51_ORF2 e-value= 9.2e-49 complete sequence hit coverage= 100%, length is 304aa with 17 gaps, 2 stops, frame= Plus2 at 3088112..3089023 in the same strand
misc_feature no id Integrase core domain; Region: rve; cl01316 intersects 100%(116aa) with IS3_IS51_ORF2 e-value= 9.2e-49 complete sequence hit coverage= 100%, length is 304aa with 17 gaps, 2 stops, frame= Plus2 at 3088112..3089023 in the same strand
misc_feature no id putative integrase, pseudogene;Similar to N-terminus to codon 110 of Xanthomonas campestris pv. vesicatoria (strain 85-10). phage-related integrase. UniProt:Q3BUY5 (EMBL:AM039952 (453 aa) fasta scores: E()=1e-18, 58.095% id in 105 aa; similarity:fasta; with=UniProt:Q3BUY5 (EMBL:AM039952;); Xanthomonas campestris pv. vesicatoria (strain 85-10).; Phage-related integrase.; length=453; id 58.095%; ungapped id 58.095%; E()=1e-18; 105 aa overlap; query 1-105; subject 1-105 intersects 19.95%(24aa) with IS3_IS51_ORF2 e-value= 9.2e-49 complete sequence hit coverage= 100%, length is 304aa with 17 gaps, 2 stops, frame= Plus2 at 3088112..3089023 in the opposite strand
CDS YP_001972787.1 putative ISXac3 like transposase intersects 89.42%(245aa) with IS3_IS51_ORF1 e-value= 3.8e-62 fragment hit coverage= 88.93%, length is 260aa with 14 gaps, 0 stops, frame= Plus2 at 3088130..3088909 in the same strand
misc_feature no id putative transposase OrfB; Reviewed; Region: PHA02517 intersects 96.40%(241aa) with IS3_IS51_ORF1 e-value= 3.8e-62 fragment hit coverage= 88.93%, length is 260aa with 14 gaps, 0 stops, frame= Plus2 at 3088130..3088909 in the same strand
misc_feature no id HMMPfam hit to PF00665, Integrase core domain, score 7.9e-27 intersects 86.42%(140aa) with IS3_IS51_ORF1 e-value= 3.8e-62 fragment hit coverage= 88.93%, length is 260aa with 14 gaps, 0 stops, frame= Plus2 at 3088130..3088909 in the same strand
misc_feature no id Integrase core domain; Region: rve; cl01316 intersects 100%(116aa) with IS3_IS51_ORF1 e-value= 3.8e-62 fragment hit coverage= 88.93%, length is 260aa with 14 gaps, 0 stops, frame= Plus2 at 3088130..3088909 in the same strand
CDS YP_001972787.1 putative ISXac3 like transposase intersects 100%(274aa) with IS3_IS51_ORF1 e-value= 1.3e-54 complete sequence hit coverage= 100%, length is 293aa with 17 gaps, 1 stops, frame= Plus2 at 3088130..3089008 in the same strand
misc_feature no id putative transposase OrfB; Reviewed; Region: PHA02517 intersects 100%(250aa) with IS3_IS51_ORF1 e-value= 1.3e-54 complete sequence hit coverage= 100%, length is 293aa with 17 gaps, 1 stops, frame= Plus2 at 3088130..3089008 in the same strand
misc_feature no id HMMPfam hit to PF00665, Integrase core domain, score 7.9e-27 intersects 100%(162aa) with IS3_IS51_ORF1 e-value= 1.3e-54 complete sequence hit coverage= 100%, length is 293aa with 17 gaps, 1 stops, frame= Plus2 at 3088130..3089008 in the same strand

misc_feature no id Integrase core domain; Region: rve; cl01316 intersects 100%(116aa) with IS3_IS51_ORF1 e-value= 1.3e-54 complete sequence hit coverage= 100%, length is 293aa with 17 gaps, 1 stops, frame= Plus2 at 3088130..3089008 in the same strand
misc_feature no id putative integrase, pseudogene;Similar to N-terminus to codon 110 of Xanthomonas campestris pv. vesicatoria (strain 85-10). phage-related integrase. UniProt:Q3BUY5 (EMBL:AM039952 (453 aa) fasta scores: E()=1e-18, 58.095% id in 105 aa; similarity:fasta; with=UniProt:Q3BUY5 (EMBL:AM039952;); Xanthomonas campestris pv. vesicatoria (strain 85-10).; Phage-related integrase.; length=453; id 58.095%; ungapped id 58.095%; E()=1e-18; 105 aa overlap; query 1-105; subject 1-105 intersects 15.85%(19aa) with IS3_IS51_ORF1 e-value= 1.3e-54 complete sequence hit coverage= 100%, length is 293aa with 17 gaps, 1 stops, frame= Plus2 at 3088130..3089008 in the opposite strand
CDS YP_001972787.1 putative ISXac3 like transposase intersects 96.35%(264aa) with IS3_IS3_ORF1 e-value= 1.3e-67 complete sequence hit coverage= 100%, length is 278aa with 10 gaps, 0 stops, frame= Plus2 at 3088133..3088966 in the same strand
misc_feature no id putative transposase OrfB; Reviewed; Region: PHA02517 intersects 100%(250aa) with IS3_IS3_ORF1 e-value= 1.3e-67 complete sequence hit coverage= 100%, length is 278aa with 10 gaps, 0 stops, frame= Plus2 at 3088133..3088966 in the same strand
misc_feature no id HMMPfam hit to PF00665, Integrase core domain, score 7.9e-27 intersects 98.15%(159aa) with IS3_IS3_ORF1 e-value= 1.3e-67 complete sequence hit coverage= 100%, length is 278aa with 10 gaps, 0 stops, frame= Plus2 at 3088133..3088966 in the same strand
misc_feature no id Integrase core domain; Region: rve; cl01316 intersects 100%(116aa) with IS3_IS3_ORF1 e-value= 1.3e-67 complete sequence hit coverage= 100%, length is 278aa with 10 gaps, 0 stops, frame= Plus2 at 3088133..3088966 in the same strand
misc_feature no id putative integrase, pseudogene;Similar to N-terminus to codon 110 of Xanthomonas campestris pv. vesicatoria (strain 85-10). phage-related integrase. UniProt:Q3BUY5 (EMBL:AM039952 (453 aa) fasta scores: E()=1e-18, 58.095% id in 105 aa; similarity:fasta; with=UniProt:Q3BUY5 (EMBL:AM039952;); Xanthomonas campestris pv. vesicatoria (strain 85-10).; Phage-related integrase.; length=453; id 58.095%; ungapped id 58.095%; E()=1e-18; 105 aa overlap; query 1-105; subject 1-105 intersects 4.37%(5aa) with IS3_IS3_ORF1 e-value= 1.3e-67 complete sequence hit coverage= 100%, length is 278aa with 10 gaps, 0 stops, frame= Plus2 at 3088133..3088966 in the opposite strand
CDS YP_001972787.1 putative ISXac3 like transposase intersects 89.05%(244aa) with IS3_IS3_ORF1 e-value= 8e-71 fragment hit coverage= 91.26%, length is 258aa with 5 gaps, 0 stops, frame= Plus2 at 3088133..3088906 in the same strand
misc_feature no id putative transposase OrfB; Reviewed; Region: PHA02517 intersects 96%(240aa) with IS3_IS3_ORF1 e-value= 8e-71 fragment hit coverage= 91.26%, length is 258aa with 5 gaps, 0 stops, frame= Plus2 at 3088133..3088906 in the same strand
misc_feature no id HMMPfam hit to PF00665, Integrase core domain, score 7.9e-27 intersects 85.80%(139aa) with IS3_IS3_ORF1 e-value= 8e-71 fragment hit coverage= 91.26%, length is 258aa with 5 gaps, 0 stops, frame= Plus2 at 3088133..3088906 in the same strand
misc_feature no id Integrase core domain; Region: rve; cl01316 intersects 100%(116aa) with IS3_IS3_ORF1 e-value= 8e-71 fragment hit coverage= 91.26%, length is 258aa with 5 gaps, 0 stops, frame= Plus2 at 3088133..3088906 in the same strand
CDS YP_001972787.1 putative ISXac3 like transposase intersects 89.05%(244aa) with IS3_IS3_ORF2 e-value= 4.4e-65 fragment hit coverage= 89.93%, length is 254aa with 8 gaps, 0 stops, frame= Plus2 at 3088145..3088906 in the same strand
misc_feature no id putative transposase OrfB; Reviewed; Region: PHA02517 intersects 96%(240aa) with IS3_IS3_ORF2 e-value= 4.4e-65 fragment hit coverage= 89.93%, length is 254aa with 8 gaps, 0 stops, frame= Plus2 at 3088145..3088906 in the same strand
misc_feature no id HMMPfam hit to PF00665, Integrase core domain, score 7.9e-27 intersects 85.80%(139aa) with IS3_IS3_ORF2 e-value= 4.4e-65 fragment hit coverage= 89.93%, length is 254aa with 8 gaps, 0 stops, frame= Plus2 at 3088145..3088906 in the same strand
misc_feature no id Integrase core domain; Region: rve; cl01316 intersects 100%(116aa) with IS3_IS3_ORF2 e-value= 4.4e-65 fragment hit coverage= 89.93%, length is 254aa with 8 gaps, 0 stops, frame= Plus2 at 3088145..3088906 in the same strand
CDS YP_001972787.1 putative ISXac3 like transposase intersects 95.99%(263aa) with IS3_IS3_ORF2 e-value= 9.6e-61 complete sequence hit coverage= 100%, length is 273aa with 19 gaps, 0 stops, frame= Plus2 at 3088145..3088963 in the same strand

misc_feature no id putative transposase OrfB; Reviewed; Region: PHA02517 intersects 100%(250aa) with IS3_IS3_ORF2 e-value= 9.6e-61 complete sequence hit coverage= 100%, length is 273aa with 19 gaps, 0 stops, frame= Plus2 at 3088145..3088963 in the same strand
misc_feature no id HMMPfam hit to PF00665, Integrase core domain, score 7.9e-27 intersects 97.53%(158aa) with IS3_IS3_ORF2 e-value= 9.6e-61 complete sequence hit coverage= 100%, length is 273aa with 19 gaps, 0 stops, frame= Plus2 at 3088145..3088963 in the same strand
misc_feature no id Integrase core domain; Region: rve; cl01316 intersects 100%(116aa) with IS3_IS3_ORF2 e-value= 9.6e-61 complete sequence hit coverage= 100%, length is 273aa with 19 gaps, 0 stops, frame= Plus2 at 3088145..3088963 in the same strand
misc_feature no id putative integrase, pseudogene;Similar to N-terminus to codon 110 of Xanthomonas campestris pv. vesicatoria (strain 85-10). phage-related integrase. UniProt:Q3BUY5 (EMBL:AM039952 (453 aa) fasta scores: E()=1e-18, 58.095% id in 105 aa; similarity:fasta; with=UniProt:Q3BUY5 (EMBL:AM039952;); Xanthomonas campestris pv. vesicatoria (strain 85-10).; Phage-related integrase.; length=453; id 58.095%; ungapped id 58.095%; E()=1e-18; 105 aa overlap; query 1-105; subject 1-105 intersects 3.55%(4aa) with IS3_IS3_ORF2 e-value= 9.6e-61 complete sequence hit coverage= 100%, length is 273aa with 19 gaps, 0 stops, frame= Plus2 at 3088145..3088963 in the opposite strand
CDS YP_001972787.1 putative ISXac3 like transposase intersects 90.88%(249aa) with IS3_IS51_ORF2 e-value= 3.9e-65 fragment hit coverage= 83.28%, length is 252aa with 15 gaps, 0 stops, frame= Plus2 at 3088166..3088921 in the same strand
misc_feature no id putative transposase OrfB; Reviewed; Region: PHA02517 intersects 98%(245aa) with IS3_IS51_ORF2 e-value= 3.9e-65 fragment hit coverage= 83.28%, length is 252aa with 15 gaps, 0 stops, frame= Plus2 at 3088166..3088921 in the same strand
misc_feature no id HMMPfam hit to PF00665, Integrase core domain, score 7.9e-27 intersects 88.89%(144aa) with IS3_IS51_ORF2 e-value= 3.9e-65 fragment hit coverage= 83.28%, length is 252aa with 15 gaps, 0 stops, frame= Plus2 at 3088166..3088921 in the same strand
misc_feature no id Integrase core domain; Region: rve; cl01316 intersects 100%(116aa) with IS3_IS51_ORF2 e-value= 3.9e-65 fragment hit coverage= 83.28%, length is 252aa with 15 gaps, 0 stops, frame= Plus2 at 3088166..3088921 in the same strand
CDS YP_001972787.1 putative ISXac3 like transposase intersects 87.96%(241aa) with IS3_IS150_ORF1 e-value= 7.6e-35 fragment hit coverage= 80.39%, length is 241aa with 8 gaps, 0 stops, frame= Plus2 at 3088184..3088906 in the same strand
misc_feature no id putative transposase OrfB; Reviewed; Region: PHA02517 intersects 96%(240aa) with IS3_IS150_ORF1 e-value= 7.6e-35 fragment hit coverage= 80.39%, length is 241aa with 8 gaps, 0 stops, frame= Plus2 at 3088184..3088906 in the same strand
misc_feature no id HMMPfam hit to PF00665, Integrase core domain, score 7.9e-27 intersects 85.80%(139aa) with IS3_IS150_ORF1 e-value= 7.6e-35 fragment hit coverage= 80.39%, length is 241aa with 8 gaps, 0 stops, frame= Plus2 at 3088184..3088906 in the same strand
misc_feature no id Integrase core domain; Region: rve; cl01316 intersects 100%(116aa) with IS3_IS150_ORF1 e-value= 7.6e-35 fragment hit coverage= 80.39%, length is 241aa with 8 gaps, 0 stops, frame= Plus2 at 3088184..3088906 in the same strand
Class A

40.
CDS YP_001972824.1 putative transposase intersects 100%(287aa) with IS3_IS51_ORF2 e-value= 2.2e-57 complete sequence hit coverage= 100%, length is 304aa with 20 gaps, 1 stops, frame= Minus3 at 3122558..3123468 in the same strand
misc_feature no id HMMPfam hit to PF00665, Integrase core domain, score 3.3e-32 intersects 100%(161aa) with IS3_IS51_ORF2 e-value= 2.2e-57 complete sequence hit coverage= 100%, length is 304aa with 20 gaps, 1 stops, frame= Minus3 at 3122558..3123468 in the same strand
misc_feature no id putative transposase OrfB; Reviewed; Region: PHA02517 intersects 100%(250aa) with IS3_IS51_ORF2 e-value= 2.2e-57 complete sequence hit coverage= 100%, length is 304aa with 20 gaps, 1 stops, frame= Minus3 at 3122558..3123468 in the same strand
misc_feature no id Integrase core domain; Region: rve; cl01316 intersects 100%(117aa) with IS3_IS51_ORF2 e-value= 2.2e-57 complete sequence hit coverage= 100%, length is 304aa with 20 gaps, 1 stops, frame= Minus3 at 3122558..3123468 in the same strand
misc_feature no id putative transposase, pseudogene;Similar to Xanthomonas campestris pv. vesicatoria (strain 85-10) isxac3 transposase. UniProt:Q3BYS7 (95 aa) fasta scores: E()=2.5e-28, 90.426% id in 94 aa; similarity:fasta;

with=UniProt:Q3BYS7; Xanthomonas campestris pv. vesicatoria (strain 85-10).; ISxac3 transposase.; length=95; id 90.426%; ungapped id 91.398%; E()=2.5e-28; 94 aa overlap; query 1-93; subject 1-94 intersects 5.28%(5aa) with IS3_IS51_ORF2 e-value= 2.2e-57 complete sequence hit coverage= 100%, length is 304aa with 20 gaps, 1 stops, frame= Minus3 at 3122558..3123468 in the same strand

CDS YP_001972824.1 putative transposase intersects 99.30%(285aa) with IS3_IS51_ORF1 e-value= 7.6e-67 complete sequence hit coverage= 100%, length is 287aa with 21 gaps, 0 stops, frame= Minus3 at 3122591..3123450 in the same strand

misc_feature no id HMMPfam hit to PF00665, Integrase core domain, score 3.3e-32 intersects 100%(161aa) with IS3_IS51_ORF1 e-value= 7.6e-67 complete sequence hit coverage= 100%, length is 287aa with 21 gaps, 0 stops, frame= Minus3 at 3122591..3123450 in the same strand

misc_feature no id putative transposase OrfB; Reviewed; Region: PHA02517 intersects 100%(250aa) with IS3_IS51_ORF1 e-value= 7.6e-67 complete sequence hit coverage= 100%, length is 287aa with 21 gaps, 0 stops, frame= Minus3 at 3122591..3123450 in the same strand

misc_feature no id Integrase core domain; Region: rve; cl01316 intersects 100%(117aa) with IS3_IS51_ORF1 e-value= 7.6e-67 complete sequence hit coverage= 100%, length is 287aa with 21 gaps, 0 stops, frame= Minus3 at 3122591..3123450 in the same strand

CDS YP_001972824.1 putative transposase intersects 97.56%(280aa) with IS3_IS150_ORF1 e-value= 1.5e-33 complete sequence hit coverage= 100%, length is 300aa with 10 gaps, 0 stops, frame= Minus3 at 3122606..3123504 in the same strand

misc_feature no id HMMPfam hit to PF00665, Integrase core domain, score 3.3e-32 intersects 100%(161aa) with IS3_IS150_ORF1 e-value= 1.5e-33 complete sequence hit coverage= 100%, length is 300aa with 10 gaps, 0 stops, frame= Minus3 at 3122606..3123504 in the same strand

misc_feature no id putative transposase OrfB; Reviewed; Region: PHA02517 intersects 100%(250aa) with IS3_IS150_ORF1 e-value= 1.5e-33 complete sequence hit coverage= 100%, length is 300aa with 10 gaps, 0 stops, frame= Minus3 at 3122606..3123504 in the same strand

misc_feature no id Integrase core domain; Region: rve; cl01316 intersects 100%(117aa) with IS3_IS150_ORF1 e-value= 1.5e-33 complete sequence hit coverage= 100%, length is 300aa with 10 gaps, 0 stops, frame= Minus3 at 3122606..3123504 in the same strand

misc_feature no id putative transposase, pseudogene;Similar to Xanthomonas campestris pv. vesicatoria (strain 85-10) isxac3 transposase. UniProt:Q3BYS7 (95 aa) fasta scores: E()=2.5e-28, 90.426% id in 94 aa; similarity:fasta; with=UniProt:Q3BYS7; Xanthomonas campestris pv. vesicatoria (strain 85-10).; ISxac3 transposase.; length=95; id 90.426%; ungapped id 91.398%; E()=2.5e-28; 94 aa overlap; query 1-93; subject 1-94 intersects 17.96%(17aa) with IS3_IS150_ORF1 e-value= 1.5e-33 complete sequence hit coverage= 100%, length is 300aa with 10 gaps, 0 stops, frame= Minus3 at 3122606..3123504 in the same strand

CDS YP_001972824.1 putative transposase intersects 96.52%(277aa) with IS3_IS3_ORF1 e-value= 2.4e-72 complete sequence hit coverage= 100%, length is 278aa with 10 gaps, 0 stops, frame= Minus3 at 3122615..3123447 in the same strand

misc_feature no id HMMPfam hit to PF00665, Integrase core domain, score 3.3e-32 intersects 98.76%(159aa) with IS3_IS3_ORF1 e-value= 2.4e-72 complete sequence hit coverage= 100%, length is 278aa with 10 gaps, 0 stops, frame= Minus3 at 3122615..3123447 in the same strand

misc_feature no id putative transposase OrfB; Reviewed; Region: PHA02517 intersects 100%(250aa) with IS3_IS3_ORF1 e-value= 2.4e-72 complete sequence hit coverage= 100%, length is 278aa with 10 gaps, 0 stops, frame= Minus3 at 3122615..3123447 in the same strand

misc_feature no id Integrase core domain; Region: rve; cl01316 intersects 100%(117aa) with IS3_IS3_ORF1 e-value= 2.4e-72 complete sequence hit coverage= 100%, length is 278aa with 10 gaps, 0 stops, frame= Minus3 at 3122615..3123447 in the same strand

CDS YP_001972824.1 putative transposase intersects 95.01%(272aa) with IS3_IS3_ORF2 e-value= 2.6e-66 complete sequence hit coverage= 100%, length is 273aa with 19 gaps, 0 stops, frame= Minus3 at 3122618..3123435 in the same strand

misc_feature no id HMMPfam hit to PF00665, Integrase core domain, score 3.3e-32 intersects 98.14%(158aa) with IS3_IS3_ORF2 e-value= 2.6e-66 complete sequence hit coverage= 100%, length is 273aa with 19 gaps, 0 stops, frame= Minus3 at 3122618..3123435 in the same strand

misc_feature no id putative transposase OrfB; Reviewed; Region: PHA02517 intersects 100%(250aa) with IS3_IS3_ORF2 e-value= 2.6e-66 complete sequence hit coverage= 100%, length is 273aa with 19 gaps, 0 stops, frame= Minus3 at 3122618..3123435 in the same strand

misc_feature no id Integrase core domain; Region: rve; cl01316 intersects 100%(117aa) with IS3_IS3_ORF2 e-value= 2.6e-66 complete sequence hit coverage= 100%, length is 273aa with 19 gaps, 0 stops, frame= Minus3 at 3122618..3123435 in the same strand
CDS YP_001972824.1 putative transposase intersects 94.43%(271aa) with IS3_IS150_ORF2 e-value= 8.1e-26 complete sequence hit coverage= 100%, length is 359aa with 40 gaps, 0 stops, frame= Minus3 at 3122633..3123708 in the same strand
misc_feature no id HMMPfam hit to PF00665, Integrase core domain, score 3.3e-32 intersects 95.03%(153aa) with IS3_IS150_ORF2 e-value= 8.1e-26 complete sequence hit coverage= 100%, length is 359aa with 40 gaps, 0 stops, frame= Minus3 at 3122633..3123708 in the same strand
misc_feature no id putative transposase OrfB; Reviewed; Region: PHA02517 intersects 100%(250aa) with IS3_IS150_ORF2 e-value= 8.1e-26 complete sequence hit coverage= 100%, length is 359aa with 40 gaps, 0 stops, frame= Minus3 at 3122633..3123708 in the same strand
misc_feature no id Integrase core domain; Region: rve; cl01316 intersects 100%(117aa) with IS3_IS150_ORF2 e-value= 8.1e-26 complete sequence hit coverage= 100%, length is 359aa with 40 gaps, 0 stops, frame= Minus3 at 3122633..3123708 in the same strand
misc_feature no id putative transposase, pseudogene;Similar to Xanthomonas campestris pv. vesicatoria (strain 85-10) isxac3 transposase. UniProt:Q3BYS7 (95 aa) fasta scores: E()=2.5e-28, 90.426% id in 94 aa; similarity:fasta; with=UniProt:Q3BYS7; Xanthomonas campestris pv. vesicatoria (strain 85-10).; ISxac3 transposase.; length=95; id 90.426%; ungapped id 91.398%; E()=2.5e-28; 94 aa overlap; query 1-93; subject 1-94 intersects 89.79%(85aa) with IS3_IS150_ORF2 e-value= 8.1e-26 complete sequence hit coverage= 100%, length is 359aa with 40 gaps, 0 stops, frame= Minus3 at 3122633..3123708 in the same strand
CDS YP_001972824.1 putative transposase intersects 92.57%(265aa) with IS3_IS3_ORF2 e-value= 2.2e-69 fragment hit coverage= 93.75%, length is 266aa with 8 gaps, 0 stops, frame= Minus3 at 3122639..3123435 in the same strand
misc_feature no id HMMPfam hit to PF00665, Integrase core domain, score 3.3e-32 intersects 93.79%(151aa) with IS3_IS3_ORF2 e-value= 2.2e-69 fragment hit coverage= 93.75%, length is 266aa with 8 gaps, 0 stops, frame= Minus3 at 3122639..3123435 in the same strand
misc_feature no id putative transposase OrfB; Reviewed; Region: PHA02517 intersects 100%(250aa) with IS3_IS3_ORF2 e-value= 2.2e-69 fragment hit coverage= 93.75%, length is 266aa with 8 gaps, 0 stops, frame= Minus3 at 3122639..3123435 in the same strand
misc_feature no id Integrase core domain; Region: rve; cl01316 intersects 100%(117aa) with IS3_IS3_ORF2 e-value= 2.2e-69 fragment hit coverage= 93.75%, length is 266aa with 8 gaps, 0 stops, frame= Minus3 at 3122639..3123435 in the same strand
CDS YP_001972824.1 putative transposase intersects 91.99%(264aa) with IS3_IS3_ORF1 e-value= 4e-75 fragment hit coverage= 94.06%, length is 265aa with 6 gaps, 0 stops, frame= Minus3 at 3122654..3123447 in the same strand
misc_feature no id HMMPfam hit to PF00665, Integrase core domain, score 3.3e-32 intersects 90.68%(146aa) with IS3_IS3_ORF1 e-value= 4e-75 fragment hit coverage= 94.06%, length is 265aa with 6 gaps, 0 stops, frame= Minus3 at 3122654..3123447 in the same strand
misc_feature no id putative transposase OrfB; Reviewed; Region: PHA02517 intersects 98.80%(247aa) with IS3_IS3_ORF1 e-value= 4e-75 fragment hit coverage= 94.06%, length is 265aa with 6 gaps, 0 stops, frame= Minus3 at 3122654..3123447 in the same strand
misc_feature no id Integrase core domain; Region: rve; cl01316 intersects 100%(117aa) with IS3_IS3_ORF1 e-value= 4e-75 fragment hit coverage= 94.06%, length is 265aa with 6 gaps, 0 stops, frame= Minus3 at 3122654..3123447 in the same strand
CDS YP_001972824.1 putative transposase intersects 91.64%(263aa) with IS3_IS51_ORF1 e-value= 1.7e-73 fragment hit coverage= 90.88%, length is 265aa with 15 gaps, 0 stops, frame= Minus3 at 3122657..3123450 in the same strand
misc_feature no id HMMPfam hit to PF00665, Integrase core domain, score 3.3e-32 intersects 90.06%(145aa) with IS3_IS51_ORF1 e-value= 1.7e-73 fragment hit coverage= 90.88%, length is 265aa with 15 gaps, 0 stops, frame= Minus3 at 3122657..3123450 in the same strand
misc_feature no id putative transposase OrfB; Reviewed; Region: PHA02517 intersects 98.40%(246aa) with IS3_IS51_ORF1 e-value= 1.7e-73 fragment hit coverage= 90.88%, length is 265aa with 15 gaps, 0 stops, frame= Minus3 at 3122657..3123450 in the same strand
misc_feature no id Integrase core domain; Region: rve; cl01316 intersects 100%(117aa) with IS3_IS51_ORF1 e-value= 1.7e-73 fragment hit coverage= 90.88%, length is 265aa with 15 gaps, 0 stops, frame= Minus3 at 3122657..3123450 in the same strand

CDS YP_001972824.1 putative transposase intersects 87.69%(251aa) with IS3_IS51_ORF2 e-value= 1.5e-73 fragment hit coverage= 83.28%, length is 252aa with 15 gaps, 0 stops, frame= Minus3 at 3122660..3123414 in the same strand
misc_feature no id HMMPfam hit to PF00665, Integrase core domain, score 3.3e-32 intersects 89.44%(144aa) with IS3_IS51_ORF2 e-value= 1.5e-73 fragment hit coverage= 83.28%, length is 252aa with 15 gaps, 0 stops, frame= Minus3 at 3122660..3123414 in the same strand
misc_feature no id putative transposase OrfB; Reviewed; Region: PHA02517 intersects 98%(245aa) with IS3_IS51_ORF2 e-value= 1.5e-73 fragment hit coverage= 83.28%, length is 252aa with 15 gaps, 0 stops, frame= Minus3 at 3122660..3123414 in the same strand
misc_feature no id Integrase core domain; Region: rve; cl01316 intersects 100%(117aa) with IS3_IS51_ORF2 e-value= 1.5e-73 fragment hit coverage= 83.28%, length is 252aa with 15 gaps, 0 stops, frame= Minus3 at 3122660..3123414 in the same strand
CDS YP_001972824.1 putative transposase intersects 83.86%(240aa) with IS3_IS150_ORF1 e-value= 2.4e-37 fragment hit coverage= 80.39%, length is 241aa with 7 gaps, 0 stops, frame= Minus3 at 3122663..3123384 in the same strand
misc_feature no id HMMPfam hit to PF00665, Integrase core domain, score 3.3e-32 intersects 88.82%(143aa) with IS3_IS150_ORF1 e-value= 2.4e-37 fragment hit coverage= 80.39%, length is 241aa with 7 gaps, 0 stops, frame= Minus3 at 3122663..3123384 in the same strand
misc_feature no id putative transposase OrfB; Reviewed; Region: PHA02517 intersects 96.27%(240aa) with IS3_IS150_ORF1 e-value= 2.4e-37 fragment hit coverage= 80.39%, length is 241aa with 7 gaps, 0 stops, frame= Minus3 at 3122663..3123384 in the same strand
misc_feature no id Integrase core domain; Region: rve; cl01316 intersects 100%(117aa) with IS3_IS150_ORF1 e-value= 2.4e-37 fragment hit coverage= 80.39%, length is 241aa with 7 gaps, 0 stops, frame= Minus3 at 3122663..3123384 in the same strand
CDS YP_001972824.1 putative transposase intersects 89.55%(257aa) with IS3_IS150_ORF2 e-value= 1.7e-58 fragment hit coverage= 68.89%, length is 258aa with 16 gaps, 0 stops, frame= Minus3 at 3122675..3123447 in the same strand
misc_feature no id HMMPfam hit to PF00665, Integrase core domain, score 3.3e-32 intersects 86.34%(139aa) with IS3_IS150_ORF2 e-value= 1.7e-58 fragment hit coverage= 68.89%, length is 258aa with 16 gaps, 0 stops, frame= Minus3 at 3122675..3123447 in the same strand
misc_feature no id putative transposase OrfB; Reviewed; Region: PHA02517 intersects 96%(240aa) with IS3_IS150_ORF2 e-value= 1.7e-58 fragment hit coverage= 68.89%, length is 258aa with 16 gaps, 0 stops, frame= Minus3 at 3122675..3123447 in the same strand
misc_feature no id Integrase core domain; Region: rve; cl01316 intersects 100%(117aa) with IS3_IS150_ORF2 e-value= 1.7e-58 fragment hit coverage= 68.89%, length is 258aa with 16 gaps, 0 stops, frame= Minus3 at 3122675..3123447 in the same strand
Class A

41.
CDS YP_001973254.1 putative ISPsy9, transposase orfa intersects 45.59%(79aa) with IS3_IS407_ORF2 e-value= 7.1e-09 complete sequence hit coverage= 100%, length is 362aa with 12 gaps, 3 stops, frame= Plus2 at 3595427..3596512 in the same strand
misc_feature no id Transposase and inactivated derivatives [DNA replication, recombination, and repair]; Region: COG2963 intersects 5.33%(5aa) with IS3_IS407_ORF2 e-value= 7.1e-09 complete sequence hit coverage= 100%, length is 362aa with 12 gaps, 3 stops, frame= Plus2 at 3595427..3596512 in the same strand
CDS YP_001973255.1 putative ISPys9 like transposase intersects 100%(278aa) with IS3_IS407_ORF2 e-value= 7.1e-09 complete sequence hit coverage= 100%, length is 362aa with 12 gaps, 3 stops, frame= Plus2 at 3595427..3596512 in the same strand
misc_feature no id putative transposase OrfB; Reviewed; Region: PHA02517 intersects 100%(251aa) with IS3_IS407_ORF2 e-value= 7.1e-09 complete sequence hit coverage= 100%, length is 362aa with 12 gaps, 3 stops, frame= Plus2 at 3595427..3596512 in the same strand
misc_feature no id HMMPfam hit to PF00665, Integrase core domain, score 4.2e-43 intersects 100%(160aa) with IS3_IS407_ORF2 e-value= 7.1e-09 complete sequence hit coverage= 100%, length is 362aa with 12 gaps, 3 stops, frame= Plus2 at 3595427..3596512 in the same strand
misc_feature no id Integrase core domain; Region: rve; cl01316 intersects 100%(118aa) with IS3_IS407_ORF2 e-value= 7.1e-09 complete sequence hit coverage= 100%, length is 362aa with 12 gaps, 3 stops, frame= Plus2 at 3595427..3596512 in the same strand

CDS YP_001973254.1 putative ISPsy9, transposase orfa intersects 44.44%(77aa) with IS3_IS150_ORF2 e-value= 2.1e-122 complete sequence hit coverage= 100%, length is 354aa with 38 gaps, 3 stops, frame= Plus2 at 3595433..3596494 in the same strand
misc_feature no id Transposase and inactivated derivatives [DNA replication, recombination, and repair]; Region: COG2963 intersects 3.33%(3aa) with IS3_IS150_ORF2 e-value= 2.1e-122 complete sequence hit coverage= 100%, length is 354aa with 38 gaps, 3 stops, frame= Plus2 at 3595433..3596494 in the same strand
CDS YP_001973255.1 putative ISPys9 like transposase intersects 100%(278aa) with IS3_IS150_ORF2 e-value= 2.1e-122 complete sequence hit coverage= 100%, length is 354aa with 38 gaps, 3 stops, frame= Plus2 at 3595433..3596494 in the same strand
misc_feature no id putative transposase OrfB; Reviewed; Region: PHA02517 intersects 100%(251aa) with IS3_IS150_ORF2 e-value= 2.1e-122 complete sequence hit coverage= 100%, length is 354aa with 38 gaps, 3 stops, frame= Plus2 at 3595433..3596494 in the same strand
misc_feature no id HMMPfam hit to PF00665, Integrase core domain, score 4.2e-43 intersects 100%(160aa) with IS3_IS150_ORF2 e-value= 2.1e-122 complete sequence hit coverage= 100%, length is 354aa with 38 gaps, 3 stops, frame= Plus2 at 3595433..3596494 in the same strand
misc_feature no id Integrase core domain; Region: rve; cl01316 intersects 100%(118aa) with IS3_IS150_ORF2 e-value= 2.1e-122 complete sequence hit coverage= 100%, length is 354aa with 38 gaps, 3 stops, frame= Plus2 at 3595433..3596494 in the same strand
CDS YP_001973254.1 putative ISPsy9, transposase orfa intersects 15.13%(26aa) with IS3_IS150_ORF1 e-value= 4.9e-88 complete sequence hit coverage= 100%, length is 300aa with 9 gaps, 0 stops, frame= Plus2 at 3595586..3596485 in the same strand
CDS YP_001973255.1 putative ISPys9 like transposase intersects 98.92%(275aa) with IS3_IS150_ORF1 e-value= 4.9e-88 complete sequence hit coverage= 100%, length is 300aa with 9 gaps, 0 stops, frame= Plus2 at 3595586..3596485 in the same strand
misc_feature no id putative transposase OrfB; Reviewed; Region: PHA02517 intersects 100%(251aa) with IS3_IS150_ORF1 e-value= 4.9e-88 complete sequence hit coverage= 100%, length is 300aa with 9 gaps, 0 stops, frame= Plus2 at 3595586..3596485 in the same strand
misc_feature no id HMMPfam hit to PF00665, Integrase core domain, score 4.2e-43 intersects 100%(160aa) with IS3_IS150_ORF1 e-value= 4.9e-88 complete sequence hit coverage= 100%, length is 300aa with 9 gaps, 0 stops, frame= Plus2 at 3595586..3596485 in the same strand
misc_feature no id Integrase core domain; Region: rve; cl01316 intersects 100%(118aa) with IS3_IS150_ORF1 e-value= 4.9e-88 complete sequence hit coverage= 100%, length is 300aa with 9 gaps, 0 stops, frame= Plus2 at 3595586..3596485 in the same strand
CDS YP_001973254.1 putative ISPsy9, transposase orfa intersects 13.41%(23aa) with IS481 e-value= 4.9e-06 complete sequence hit coverage= 100%, length is 312aa with 46 gaps, 1 stops, frame= Plus2 at 3595595..3596530 in the same strand
CDS YP_001973255.1 putative ISPys9 like transposase intersects 100%(278aa) with IS481 e-value= 4.9e-06 complete sequence hit coverage= 100%, length is 312aa with 46 gaps, 1 stops, frame= Plus2 at 3595595..3596530 in the same strand
misc_feature no id putative transposase OrfB; Reviewed; Region: PHA02517 intersects 100%(251aa) with IS481 e-value= 4.9e-06 complete sequence hit coverage= 100%, length is 312aa with 46 gaps, 1 stops, frame= Plus2 at 3595595..3596530 in the same strand
misc_feature no id HMMPfam hit to PF00665, Integrase core domain, score 4.2e-43 intersects 100%(160aa) with IS481 e-value= 4.9e-06 complete sequence hit coverage= 100%, length is 312aa with 46 gaps, 1 stops, frame= Plus2 at 3595595..3596530 in the same strand
misc_feature no id Integrase core domain; Region: rve; cl01316 intersects 100%(118aa) with IS481 e-value= 4.9e-06 complete sequence hit coverage= 100%, length is 312aa with 46 gaps, 1 stops, frame= Plus2 at 3595595..3596530 in the same strand
CDS YP_001973256.1 hypothetical protein intersects 3.81%(5aa) with IS481 e-value= 4.9e-06 complete sequence hit coverage= 100%, length is 312aa with 46 gaps, 1 stops, frame= Plus2 at 3595595..3596530 in the opposite strand
repeat_region no id no product intersects 2.08%(2aa) with IS481 e-value= 4.9e-06 complete sequence hit coverage= 100%, length is 312aa with 46 gaps, 1 stops, frame= Plus2 at 3595595..3596530 in the opposite strand
CDS YP_001973254.1 putative ISPsy9, transposase orfa intersects 5.94%(10aa) with IS3_IS150_ORF2 e-value= 2.4e-152 fragment hit coverage= 76.61%, length is 287aa with 14 gaps, 1 stops, frame= Plus2 at 3595634..3596494 in the same strand

CDS YP_001973255.1 putative ISPys9 like transposase intersects 100%(278aa) with IS3_IS150_ORF2 e-value= 2.4e-152 fragment hit coverage= 76.61%, length is 287aa with 14 gaps, 1 stops, frame= Plus2 at 3595634..3596494 in the same strand
misc_feature no id putative transposase OrfB; Reviewed; Region: PHA02517 intersects 100%(251aa) with IS3_IS150_ORF2 e-value= 2.4e-152 fragment hit coverage= 76.61%, length is 287aa with 14 gaps, 1 stops, frame= Plus2 at 3595634..3596494 in the same strand
misc_feature no id HMMPfam hit to PF00665, Integrase core domain, score 4.2e-43 intersects 100%(160aa) with IS3_IS150_ORF2 e-value= 2.4e-152 fragment hit coverage= 76.61%, length is 287aa with 14 gaps, 1 stops, frame= Plus2 at 3595634..3596494 in the same strand
misc_feature no id Integrase core domain; Region: rve; cl01316 intersects 100%(118aa) with IS3_IS150_ORF2 e-value= 2.4e-152 fragment hit coverage= 76.61%, length is 287aa with 14 gaps, 1 stops, frame= Plus2 at 3595634..3596494 in the same strand
CDS YP_001973254.1 putative ISPsy9, transposase orfa intersects 4.21%(7aa) with IS3_IS51_ORF1 e-value= 8.4e-39 complete sequence hit coverage= 100%, length is 291aa with 18 gaps, 1 stops, frame= Plus2 at 3595643..3596515 in the same strand
CDS YP_001973255.1 putative ISPys9 like transposase intersects 100%(278aa) with IS3_IS51_ORF1 e-value= 8.4e-39 complete sequence hit coverage= 100%, length is 291aa with 18 gaps, 1 stops, frame= Plus2 at 3595643..3596515 in the same strand
misc_feature no id putative transposase OrfB; Reviewed; Region: PHA02517 intersects 100%(251aa) with IS3_IS51_ORF1 e-value= 8.4e-39 complete sequence hit coverage= 100%, length is 291aa with 18 gaps, 1 stops, frame= Plus2 at 3595643..3596515 in the same strand
misc_feature no id HMMPfam hit to PF00665, Integrase core domain, score 4.2e-43 intersects 100%(160aa) with IS3_IS51_ORF1 e-value= 8.4e-39 complete sequence hit coverage= 100%, length is 291aa with 18 gaps, 1 stops, frame= Plus2 at 3595643..3596515 in the same strand
misc_feature no id Integrase core domain; Region: rve; cl01316 intersects 100%(118aa) with IS3_IS51_ORF1 e-value= 8.4e-39 complete sequence hit coverage= 100%, length is 291aa with 18 gaps, 1 stops, frame= Plus2 at 3595643..3596515 in the same strand
CDS YP_001973256.1 hypothetical protein intersects 0.24%(0aa) with IS3_IS51_ORF1 e-value= 8.4e-39 complete sequence hit coverage= 100%, length is 291aa with 18 gaps, 1 stops, frame= Plus2 at 3595643..3596515 in the opposite strand
CDS YP_001973254.1 putative ISPsy9, transposase orfa intersects 4.21%(7aa) with IS3_IS51_ORF2 e-value= 7.9e-18 complete sequence hit coverage= 100%, length is 292aa with 25 gaps, 1 stops, frame= Plus2 at 3595643..3596518 in the same strand
CDS YP_001973255.1 putative ISPys9 like transposase intersects 100%(278aa) with IS3_IS51_ORF2 e-value= 7.9e-18 complete sequence hit coverage= 100%, length is 292aa with 25 gaps, 1 stops, frame= Plus2 at 3595643..3596518 in the same strand
misc_feature no id putative transposase OrfB; Reviewed; Region: PHA02517 intersects 100%(251aa) with IS3_IS51_ORF2 e-value= 7.9e-18 complete sequence hit coverage= 100%, length is 292aa with 25 gaps, 1 stops, frame= Plus2 at 3595643..3596518 in the same strand
misc_feature no id HMMPfam hit to PF00665, Integrase core domain, score 4.2e-43 intersects 100%(160aa) with IS3_IS51_ORF2 e-value= 7.9e-18 complete sequence hit coverage= 100%, length is 292aa with 25 gaps, 1 stops, frame= Plus2 at 3595643..3596518 in the same strand
misc_feature no id Integrase core domain; Region: rve; cl01316 intersects 100%(118aa) with IS3_IS51_ORF2 e-value= 7.9e-18 complete sequence hit coverage= 100%, length is 292aa with 25 gaps, 1 stops, frame= Plus2 at 3595643..3596518 in the same strand
CDS YP_001973256.1 hypothetical protein intersects 0.95%(1aa) with IS3_IS51_ORF2 e-value= 7.9e-18 complete sequence hit coverage= 100%, length is 292aa with 25 gaps, 1 stops, frame= Plus2 at 3595643..3596518 in the opposite strand
CDS YP_001973254.1 putative ISPsy9, transposase orfa intersects 3.64%(6aa) with IS3_IS3_ORF1 e-value= 5.9e-76 complete sequence hit coverage= 100%, length is 279aa with 8 gaps, 0 stops, frame= Plus2 at 3595646..3596482 in the same strand
CDS YP_001973255.1 putative ISPys9 like transposase intersects 98.56%(274aa) with IS3_IS3_ORF1 e-value= 5.9e-76 complete sequence hit coverage= 100%, length is 279aa with 8 gaps, 0 stops, frame= Plus2 at 3595646..3596482 in the same strand
misc_feature no id putative transposase OrfB; Reviewed; Region: PHA02517 intersects 100%(251aa) with IS3_IS3_ORF1 e-value= 5.9e-76 complete sequence hit coverage= 100%, length is 279aa with 8 gaps, 0 stops, frame= Plus2 at 3595646..3596482 in the same strand

misc_feature no id HMMPfam hit to PF00665, Integrase core domain, score 4.2e-43 intersects 100%(160aa) with IS3_IS3_ORF1 e-value= 5.9e-76 complete sequence hit coverage= 100%, length is 279aa with 8 gaps, 0 stops, frame= Plus2 at 3595646..3596482 in the same strand
misc_feature no id Integrase core domain; Region: rve; cl01316 intersects 100%(118aa) with IS3_IS3_ORF1 e-value= 5.9e-76 complete sequence hit coverage= 100%, length is 279aa with 8 gaps, 0 stops, frame= Plus2 at 3595646..3596482 in the same strand
CDS YP_001973254.1 putative ISPsy9, transposase orfa intersects 1.34%(2aa) with IS3_IS3_ORF2 e-value= 1.1e-64 complete sequence hit coverage= 100%, length is 279aa with 9 gaps, 1 stops, frame= Plus2 at 3595658..3596494 in the same strand
CDS YP_001973255.1 putative ISPys9 like transposase intersects 100%(278aa) with IS3_IS3_ORF2 e-value= 1.1e-64 complete sequence hit coverage= 100%, length is 279aa with 9 gaps, 1 stops, frame= Plus2 at 3595658..3596494 in the same strand
misc_feature no id putative transposase OrfB; Reviewed; Region: PHA02517 intersects 100%(251aa) with IS3_IS3_ORF2 e-value= 1.1e-64 complete sequence hit coverage= 100%, length is 279aa with 9 gaps, 1 stops, frame= Plus2 at 3595658..3596494 in the same strand
misc_feature no id HMMPfam hit to PF00665, Integrase core domain, score 4.2e-43 intersects 100%(160aa) with IS3_IS3_ORF2 e-value= 1.1e-64 complete sequence hit coverage= 100%, length is 279aa with 9 gaps, 1 stops, frame= Plus2 at 3595658..3596494 in the same strand
misc_feature no id Integrase core domain; Region: rve; cl01316 intersects 100%(118aa) with IS3_IS3_ORF2 e-value= 1.1e-64 complete sequence hit coverage= 100%, length is 279aa with 9 gaps, 1 stops, frame= Plus2 at 3595658..3596494 in the same strand
CDS YP_001973255.1 putative ISPys9 like transposase intersects 84.17%(234aa) with IS3_IS51_ORF1 e-value= 4.9e-45 fragment hit coverage= 79.80%, length is 234aa with 12 gaps, 0 stops, frame= Plus2 at 3595766..3596467 in the same strand
misc_feature no id putative transposase OrfB; Reviewed; Region: PHA02517 intersects 92.83%(233aa) with IS3_IS51_ORF1 e-value= 4.9e-45 fragment hit coverage= 79.80%, length is 234aa with 12 gaps, 0 stops, frame= Plus2 at 3595766..3596467 in the same strand
misc_feature no id HMMPfam hit to PF00665, Integrase core domain, score 4.2e-43 intersects 98.75%(158aa) with IS3_IS51_ORF1 e-value= 4.9e-45 fragment hit coverage= 79.80%, length is 234aa with 12 gaps, 0 stops, frame= Plus2 at 3595766..3596467 in the same strand
misc_feature no id Integrase core domain; Region: rve; cl01316 intersects 100%(118aa) with IS3_IS51_ORF1 e-value= 4.9e-45 fragment hit coverage= 79.80%, length is 234aa with 12 gaps, 0 stops, frame= Plus2 at 3595766..3596467 in the same strand
CDS YP_001973255.1 putative ISPys9 like transposase intersects 63.31%(176aa) with IS3_IS51_ORF2 e-value= 5.2e-32 fragment hit coverage= 57.41%, length is 176aa with 6 gaps, 0 stops, frame= Plus2 at 3595817..3596344 in the same strand
misc_feature no id putative transposase OrfB; Reviewed; Region: PHA02517 intersects 70.12%(176aa) with IS3_IS51_ORF2 e-value= 5.2e-32 fragment hit coverage= 57.41%, length is 176aa with 6 gaps, 0 stops, frame= Plus2 at 3595817..3596344 in the same strand
misc_feature no id HMMPfam hit to PF00665, Integrase core domain, score 4.2e-43 intersects 73.12%(117aa) with IS3_IS51_ORF2 e-value= 5.2e-32 fragment hit coverage= 57.41%, length is 176aa with 6 gaps, 0 stops, frame= Plus2 at 3595817..3596344 in the same strand
misc_feature no id Integrase core domain; Region: rve; cl01316 intersects 99.15%(117aa) with IS3_IS51_ORF2 e-value= 5.2e-32 fragment hit coverage= 57.41%, length is 176aa with 6 gaps, 0 stops, frame= Plus2 at 3595817..3596344 in the same strand
CDS YP_001973255.1 putative ISPys9 like transposase intersects 12.95%(36aa) with IS481 e-value= 3.8e-07 fragment hit coverage= 11.11%, length is 36aa with 3 gaps, 0 stops, frame= Plus2 at 3596321..3596428 in the same strand
misc_feature no id putative transposase OrfB; Reviewed; Region: PHA02517 intersects 14.34%(36aa) with IS481 e-value= 3.8e-07 fragment hit coverage= 11.11%, length is 36aa with 3 gaps, 0 stops, frame= Plus2 at 3596321..3596428 in the same strand
misc_feature no id HMMPfam hit to PF00665, Integrase core domain, score 4.2e-43 intersects 22.50%(36aa) with IS481 e-value= 3.8e-07 fragment hit coverage= 11.11%, length is 36aa with 3 gaps, 0 stops, frame= Plus2 at 3596321..3596428 in the same strand
misc_feature no id Integrase core domain; Region: rve; cl01316 intersects 7.63%(9aa) with IS481 e-value= 3.8e-07 fragment hit coverage= 11.11%, length is 36aa with 3 gaps, 0 stops, frame= Plus2 at 3596321..3596428 in the same strand

Class A

42.
repeat_region no id no product intersects 67.60%(306aa) with IS110 e-value= 3.4e-46 complete sequence hit coverage= 100%, length is 306aa with 110 gaps, 1 stops, frame= Plus1 at 3623575..3624492 in the opposite strand
CDS YP_001973280.1 putative transposase intersects 99.67%(304aa) with IS110 e-value= 3.4e-46 complete sequence hit coverage= 100%, length is 306aa with 110 gaps, 1 stops, frame= Plus1 at 3623575..3624492 in the same strand
misc_feature no id Transposase and inactivated derivatives [DNA replication, recombination, and repair]; Region: COG3547 intersects 100%(292aa) with IS110 e-value= 3.4e-46 complete sequence hit coverage= 100%, length is 306aa with 110 gaps, 1 stops, frame= Plus1 at 3623575..3624492 in the same strand
misc_feature no id Transposase; Region: Transposase_9; pfam01548 intersects 100%(79aa) with IS110 e-value= 3.4e-46 complete sequence hit coverage= 100%, length is 306aa with 110 gaps, 1 stops, frame= Plus1 at 3623575..3624492 in the same strand
misc_feature no id HMMPfam hit to PF01548, Transposase, score 1.8e-06 intersects 100%(98aa) with IS110 e-value= 3.4e-46 complete sequence hit coverage= 100%, length is 306aa with 110 gaps, 1 stops, frame= Plus1 at 3623575..3624492 in the same strand
misc_feature no id HMMPfam hit to PF02371, Transposase IS116/IS110/IS902 family, score 4.3e-27 intersects 100%(108aa) with IS110 e-value= 3.4e-46 complete sequence hit coverage= 100%, length is 306aa with 110 gaps, 1 stops, frame= Plus1 at 3623575..3624492 in the same strand
misc_feature no id Transposase IS116/IS110/IS902 family; Region: Transposase_20; pfam02371 intersects 100%(84aa) with IS110 e-value= 3.4e-46 complete sequence hit coverage= 100%, length is 306aa with 110 gaps, 1 stops, frame= Plus1 at 3623575..3624492 in the same strand
repeat_region no id no product intersects 65.17%(295aa) with IS110 e-value= 1e-48 fragment hit coverage= 96.88%, length is 295aa with 108 gaps, 0 stops, frame= Plus1 at 3623575..3624459 in the opposite strand
CDS YP_001973280.1 putative transposase intersects 96.72%(295aa) with IS110 e-value= 1e-48 fragment hit coverage= 96.88%, length is 295aa with 108 gaps, 0 stops, frame= Plus1 at 3623575..3624459 in the same strand
misc_feature no id Transposase and inactivated derivatives [DNA replication, recombination, and repair]; Region: COG3547 intersects 100%(292aa) with IS110 e-value= 1e-48 fragment hit coverage= 96.88%, length is 295aa with 108 gaps, 0 stops, frame= Plus1 at 3623575..3624459 in the same strand
misc_feature no id Transposase; Region: Transposase_9; pfam01548 intersects 100%(79aa) with IS110 e-value= 1e-48 fragment hit coverage= 96.88%, length is 295aa with 108 gaps, 0 stops, frame= Plus1 at 3623575..3624459 in the same strand
misc_feature no id HMMPfam hit to PF01548, Transposase, score 1.8e-06 intersects 100%(98aa) with IS110 e-value= 1e-48 fragment hit coverage= 96.88%, length is 295aa with 108 gaps, 0 stops, frame= Plus1 at 3623575..3624459 in the same strand
misc_feature no id HMMPfam hit to PF02371, Transposase IS116/IS110/IS902 family, score 4.3e-27 intersects 100%(108aa) with IS110 e-value= 1e-48 fragment hit coverage= 96.88%, length is 295aa with 108 gaps, 0 stops, frame= Plus1 at 3623575..3624459 in the same strand
misc_feature no id Transposase IS116/IS110/IS902 family; Region: Transposase_20; pfam02371 intersects 100%(84aa) with IS110 e-value= 1e-48 fragment hit coverage= 96.88%, length is 295aa with 108 gaps, 0 stops, frame= Plus1 at 3623575..3624459 in the same strand
Class A

43.
CDS YP_001973591.1 putative RNA polymerase ECF sigma factor intersects 30.34%(50aa) with IS21_ORF1 e-value= 5e-06 fragment hit coverage= 11.62%, length is 53aa with 8 gaps, 1 stops, frame= Minus1 at 3988837..3988994 in the same strand
misc_feature no id HMMPfam hit to PF04545, Sigma-70, region, score 4.6e-10 intersects 89.33%(44aa) with IS21_ORF1 e-value= 5e-06 fragment hit coverage= 11.62%, length is 53aa with 8 gaps, 1 stops, frame= Minus1 at 3988837..3988994 in the same strand
misc_feature no id RNA polymerase sigma factor, sigma-70 family; Region: sigma70-ECF; TIGR02937 intersects 28.17%(43aa) with IS21_ORF1 e-value= 5e-06 fragment hit coverage= 11.62%, length is 53aa with 8 gaps, 1 stops, frame= Minus1 at 3988837..3988994 in the same strand
misc_feature no id HMMPfam hit to PF08281, Sigma-70, region, score 9.3e-17 intersects 79.01%(42aa) with IS21_ORF1 e-value= 5e-06 fragment hit coverage= 11.62%, length is 53aa with 8 gaps, 1 stops, frame= Minus1 at 3988837..3988994 in the same strand

Class B

44.
repeat_region no id no product intersects 25%(6aa) with IS481e-value= 6.4e-95 complete sequence hit coverage= 100%, length is 314aa with 38 gaps, 1 stops, frame= Minus2 at 4036800..4037740 in the opposite strand
CDS YP_001973628.1 putative transposase intersects 98.31%(309aa) with IS481 e-value= 6.4e-95 complete sequence hit coverage= 100%, length is 314aa with 38 gaps, 1 stops, frame= Minus2 at 4036800..4037740 in the same strand
misc_feature no id HMMPfam hit to PF00665, Integrase core domain, score 4.9e-39 intersects 100%(178aa) with IS481 e-value= 6.4e-95 complete sequence hit coverage= 100%, length is 314aa with 38 gaps, 1 stops, frame= Minus2 at 4036800..4037740 in the same strand
misc_feature no id Transposase and inactivated derivatives [DNA replication, recombination, and repair]; Region: Tra5; COG2801 intersects 100%(227aa) with IS481 e-value= 6.4e-95 complete sequence hit coverage= 100%, length is 314aa with 38 gaps, 1 stops, frame= Minus2 at 4036800..4037740 in the same strand
misc_feature no id Integrase core domain; Region: rve; cl01316 intersects 100%(134aa) with IS481 e-value= 6.4e-95 complete sequence hit coverage= 100%, length is 314aa with 38 gaps, 1 stops, frame= Minus2 at 4036800..4037740 in the same strand
misc_feature no id Transposase and inactivated derivatives [DNA replication, recombination, and repair]; Region: COG3415 intersects 100%(100aa) with IS481 e-value= 6.4e-95 complete sequence hit coverage= 100%, length is 314aa with 38 gaps, 1 stops, frame= Minus2 at 4036800..4037740 in the same strand
CDS YP_001973628.1 putative transposase intersects 28.15%(88aa) with IS3_IS150_ORF2 e-value= 1.8e-10 fragment hit coverage= 23.39%, length is 89aa with 2 gaps, 0 stops, frame= Minus2 at 4036854..4037119 in the same strand
misc_feature no id HMMPfam hit to PF00665, Integrase core domain, score 4.9e-39 intersects 49.81%(88aa) with IS3_IS150_ORF2 e-value= 1.8e-10 fragment hit coverage= 23.39%, length is 89aa with 2 gaps, 0 stops, frame= Minus2 at 4036854..4037119 in the same strand
misc_feature no id Transposase and inactivated derivatives [DNA replication, recombination, and repair]; Region: Tra5; COG2801 intersects 39.06%(88aa) with IS3_IS150_ORF2 e-value= 1.8e-10 fragment hit coverage= 23.39%, length is 89aa with 2 gaps, 0 stops, frame= Minus2 at 4036854..4037119 in the same strand
misc_feature no id Integrase core domain; Region: rve; cl01316 intersects 37.81%(50aa) with IS3_IS150_ORF2 e-value= 1.8e-10 fragment hit coverage= 23.39%, length is 89aa with 2 gaps, 0 stops, frame= Minus2 at 4036854..4037119 in the same strand
CDS YP_001973628.1 putative transposase intersects 7.20%(22aa) with IS3_IS407_ORF2 e-value= 4.1e-07 fragment hit coverage= 6.69%, length is 23aa with 0 gaps, 0 stops, frame= Minus2 at 4036857..4036924 in the same strand
misc_feature no id HMMPfam hit to PF00665, Integrase core domain, score 4.9e-39 intersects 12.73%(22aa) with IS3_IS407_ORF2 e-value= 4.1e-07 fragment hit coverage= 6.69%, length is 23aa with 0 gaps, 0 stops, frame= Minus2 at 4036857..4036924 in the same strand
misc_feature no id Transposase and inactivated derivatives [DNA replication, recombination, and repair]; Region: Tra5; COG2801 intersects 9.99%(22aa) with IS3_IS407_ORF2 e-value= 4.1e-07 fragment hit coverage= 6.69%, length is 23aa with 0 gaps, 0 stops, frame= Minus2 at 4036857..4036924 in the same strand
Class A

45.
CDS YP_001973922.1 putative transmembrane phosphatase intersects 1.65%(7aa) with IS3_IS2_ORF2 e-value= 9.6e-06 fragment hit coverage= 9.27%, length is 28aa with 0 gaps, 0 stops, frame= Plus2 at 4365179..4365262 in the opposite strand
CDS YP_001973923.1 putative hydrolase intersects 3.77%(22aa) with IS3_IS2_ORF2 e-value= 9.6e-06 fragment hit coverage= 9.27%, length is 28aa with 0 gaps, 0 stops, frame= Plus2 at 4365179..4365262 in the opposite strand
misc_feature no id S-adenosylmethionine-dependent methyltransferases (SAM or AdoMet-MTase), class I; AdoMet-MTases are enzymes that use S-adenosyl-L-methionine (SAM or AdoMet) as a substrate for methyltransfer, creating the product S-adenosyl-L-homocysteine (AdoHcy)...; Region: AdoMet_MTases; cl12011 intersects 6.11%(19aa) with IS3_IS2_ORF2e-value= 9.6e-06 fragment hit coverage= 9.27%, length is 28aa with 0 gaps, 0 stops, frame= Plus2 at 4365179..4365262 in the opposite strand
Class B

46.
CDS YP_001974031.1 putative transposase intersects 98.31%(309aa) with IS481 e-value= 1.5e-93 complete sequence hit coverage= 100%, length is 314aa with 38 gaps, 1 stops, frame= Minus2 at 4481847..4482787 in the same strand
misc_feature no id HMMPfam hit to PF00665, Integrase core domain, score 8.2e-34 intersects 100%(178aa) with IS481 e-value= 1.5e-93 complete sequence hit coverage= 100%, length is 314aa with 38 gaps, 1 stops, frame= Minus2 at 4481847..4482787 in the same strand
misc_feature no id Integrase core domain; Region: rve; cl01316 intersects 100%(132aa) with IS481 e-value= 1.5e-93 complete sequence hit coverage= 100%, length is 314aa with 38 gaps, 1 stops, frame= Minus2 at 4481847..4482787 in the same strand
misc_feature no id Transposase and inactivated derivatives [DNA replication, recombination, and repair]; Region: COG3415 intersects 100%(100aa) with IS481 e-value= 1.5e-93 complete sequence hit coverage= 100%, length is 314aa with 38 gaps, 1 stops, frame= Minus2 at 4481847..4482787 in the same strand
CDS YP_001974031.1 putative transposase intersects 96.72%(304aa) with IS481 e-value= 5.6e-93 fragment hit coverage= 97.44%, length is 305aa with 38 gaps, 0 stops, frame= Minus2 at 4481874..4482787 in the same strand
misc_feature no id HMMPfam hit to PF00665, Integrase core domain, score 8.2e-34 intersects 100%(178aa) with IS481 e-value= 5.6e-93 fragment hit coverage= 97.44%, length is 305aa with 38 gaps, 0 stops, frame= Minus2 at 4481874..4482787 in the same strand
misc_feature no id Integrase core domain; Region: rve; cl01316 intersects 100%(132aa) with IS481 e-value= 5.6e-93 fragment hit coverage= 97.44%, length is 305aa with 38 gaps, 0 stops, frame= Minus2 at 4481874..4482787 in the same strand
misc_feature no id Transposase and inactivated derivatives [DNA replication, recombination, and repair]; Region: COG3415 intersects 100%(100aa) with IS481 e-value= 5.6e-93 fragment hit coverage= 97.44%, length is 305aa with 38 gaps, 0 stops, frame= Minus2 at 4481874..4482787 in the same strand
CDS YP_001974031.1 putative transposase intersects 24.66%(77aa) with IS3_IS150_ORF2 e-value= 2.5e-08 fragment hit coverage= 20.57%, length is 78aa with 2 gaps, 0 stops, frame= Minus2 at 4481934..4482166 in the same strand
misc_feature no id HMMPfam hit to PF00665, Integrase core domain, score 8.2e-34 intersects 43.63%(77aa) with IS3_IS150_ORF2 e-value= 2.5e-08 fragment hit coverage= 20.57%, length is 78aa with 2 gaps, 0 stops, frame= Minus2 at 4481934..4482166 in the same strand
misc_feature no id Integrase core domain; Region: rve; cl01316 intersects 38.38%(50aa) with IS3_IS150_ORF2 e-value= 2.5e-08 fragment hit coverage= 20.57%, length is 78aa with 2 gaps, 0 stops, frame= Minus2 at 4481934..4482166 in the same strand
CDS YP_001974031.1 putative transposase intersects 28.47%(89aa) with IS630 e-value= 8.1e-07 fragment hit coverage= 28.72%, length is 90aa with 18 gaps, 0 stops, frame= Minus2 at 4482465..4482733 in the same strand
misc_feature no id Transposase and inactivated derivatives [DNA replication, recombination, and repair]; Region: COG3415 intersects 89.67%(89aa) with IS630 e-value= 8.1e-07 fragment hit coverage= 28.72%, length is 90aa with 18 gaps, 0 stops, frame= Minus2 at 4482465..4482733 in the same strand
Class A

47.
misc_feature no id putative transposase, pseudogene;Similar to codons 30 to 68 of Xanthomonas oryzae pv. oryzae putative transposase. putative transposase. UniProt:Q5GXG6 (161 aa) fasta scores: E()=4.3e-10, 78.947% id in 38 aa; similarity:fasta; with=UniProt:Q5GXG6; Xanthomonas oryzae pv. oryzae.; Putative transposase. Putative transposase.; length=161; id 78.947%; ungapped id 78.947%; E()=4.3e-10; 38 aa overlap; query 1-38; subject 30-67 intersects 100%(39aa) with IS5_IS5 e-value= 2.5e-06 complete sequence hit coverage= 100%, length is 279aa with 155 gaps, 5 stops, frame= Plus3 at 4666605..4667441 in the same strand
CDS YP_001974180.1 putative phage-related protein intersects 100%(40aa) with IS5_IS5 e-value= 2.5e-06 complete sequence hit coverage= 100%, length is 279aa with 155 gaps, 5 stops, frame= Plus3 at 4666605..4667441 in the same strand
misc_feature no id putative transposase, pseudogene;Similar to codons 30 to 68 of Xanthomonas oryzae pv. oryzae putative transposase. putative transposase. UniProt:Q5GXG6 (161 aa) fasta scores: E()=4.3e-10, 78.947% id in 38 aa; similarity:fasta; with=UniProt:Q5GXG6; Xanthomonas oryzae pv. oryzae.; Putative transposase. Putative transposase.; length=161; id 78.947%; ungapped id 78.947%; E()=4.3e-10; 38 aa overlap; query 1-38; subject 30-67

intersects 100%(39aa) with IS5_IS5 e-value= 2.4e-24 fragment hit coverage= 16.82%, length is 70aa with 1 gaps, 2 stops, frame= Plus3 at 4666605..4666814 in the same strand
Class A

Not Found: 850181..850426 1 HMMPfam hit to PF01527, Transposase, score 0.00031
Not Found: 2334049..2334348 1 Transposase and inactivated derivatives [DNA replication, recombination, and repair]; Region: COG2963
Not Found: 2510883..2511005 -1 Mu transposase, C-terminal; Region: Mu-transpos_C; pfam09299
Not Found: 2849539..2849859 -1 Transposase IS200 like; Region: Transposase_17; cl00848
Not Found: 3595143..3595388 1 HMMPfam hit to PF01527, Transposase, score 0.00054
Not Found: 4167747..4167902 -1 putative transposase, pseudogene;Similar to N-terminus of Leifsonia xyli subsp. xyli Tnp transposase, islxx4. UniProt:Q6AEV8 (121 aa) fasta scores: E()=8.3, 40.909% id in 44 aa; similarity:fasta; with=UniProt:Q6AEV8; Leifsonia xyli subsp. xyli.; tnp; Transposase, ISlxx4.; length=121; id 40.909%; ungapped id 46.154%; E()=8.3; 44 aa overlap; query 12-50; subject 10-53

Pred: 4665734..4665888 -1 IS5_IS5 e-value= 3.8e-15 fragment hit coverage= 12.56%, length is 52aa with 1 gaps, 1 stops, frame= Minus3
